# Ni-catalyzed benzylic *β*-C(sp^3^)–H bond activation of formamides

**DOI:** 10.1038/s41467-022-35541-6

**Published:** 2022-12-22

**Authors:** Rong-Hua Wang, Wei-Wei Xu, Hongli Wu, Yue Li, Jiang-Fei Li, Tao Zhang, Genping Huang, Mengchun Ye

**Affiliations:** 1 State Key Laboratory and Institute of Elemento-Organic Chemistry, College of Chemistry, Nankai University, Tianjin, 300071 China; 2 Department of Chemistry, School of Science and Tianjin Key Laboratory of Molecular Optoelectronic Sciences, Tianjin University, Tianjin, 300072 China; 3 Haihe Laboratory of Sustainable Chemical Transformations, Tianjin, 300192 China

**Keywords:** Catalyst synthesis, Homogeneous catalysis, Synthetic chemistry methodology

## Abstract

The development of transition metal-catalyzed *β*-C–H bond activation via highly-strained 4-membered metallacycles has been a formidable task. So far, only scarce examples have been reported to undergo *β*-C–H bond activation via 4-membered metallacycles, and all of them rely on precious metals. In contrast, earth-abundant and inexpensive 3d transition metal-catalyzed *β*-C–H bond activation via 4-membered metallacycles still remains an elusive challenge. Herein, we report a phosphine oxide-ligated Ni−Al bimetallic catalyst to activate secondary benzylic C(sp^3^)–H bonds of formamides via 4-membered nickelacycles, providing a series of *α,β*-unsaturated *γ*-lactams in up to 97% yield.

## Abstract (web summary)

The development of 3d-metal-catalyzed β-C–H bond activation via 4-membered metallacycles remains an elusive challenge. Here, the authors report a Ni-Al bimetal-catalyzed β-C(sp3)–H bond activation of formamides via 4-membered nickelacycles.

## Introduction

**Fig. 1:**
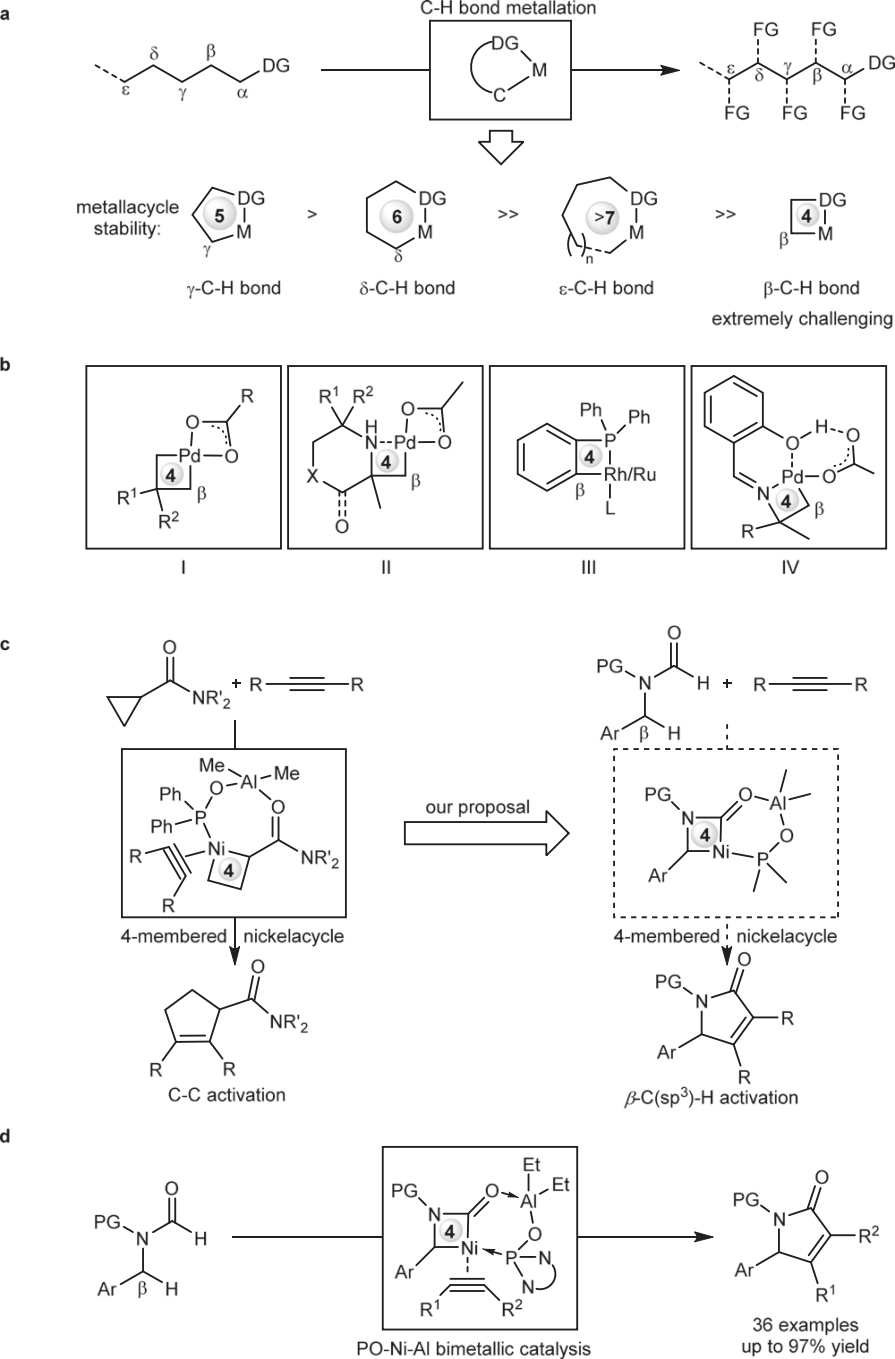
Transition metal-catalyzed C–H activation via cyclometallation. **a** The activation of C–H bonds at different position to the coordinating atom of directing groups is faced with varying levels of difficulty. *γ*-C–H bonds are the most easily activated via 5-membered metallacycles, while the activation of proximate *β*-C–H bonds via highly-strained 4-membered metallacycles is extremely challenging. **b** Only scarce examples on *β*-C–H bond activation via 4-membered metallacycles have been reported, and all of them rely on precious metals such as Pd, Rh, and Ru. **c** Proposed secondary *β*-C(sp^3^)–H bond activation: PO-ligated Ni–Al bimetal-catalyzed C–H bond activation via 4-membered nickelacycle. **d** 3d-Transition metal-catalyzed secondary C(sp^3^)–H bond activation via 4-membered nickelacycle (this work). DG Directing group. M Metal catalyst. FG Functional group. PG Protecting group.

Cyclometallation represents one of the most efficient pathways for transition metal-catalyzed C–H activation, and remarkable progress has been achieved by the combination of a diverse range of directing groups (DG) and transition metals during the past several decades^1–4^. However, most examples proceed through stable 5-membered metallacycles, allowing the activation of C−H bonds at the position of *γ* to a coordinating atom (Fig. 1a). In contrast, the activation of non-*γ*-C–H bonds via larger or smaller-size metallacycles is faced with great challenges owing to unfavorable entropic effect or ring strain^5^. Among various non-*γ*-C–H bonds, proximate *β*-C–H bonds are in general the most difficult to activate owing to the need of forming highly-strained 4-membered metallacycles. So far, only scarce examples have been achieved for the activation of proximate *β*-C–H bonds via 4-membered metallacycles. For example, the incorporation of palladium into molecules via alkene carbopalladation or 1,4-palladium shift of aryl halides proves to be an efficient strategy for the activation of *β*-C(sp^3^)–H bonds via 4-membered palladacycles (Fig. 1b, I)^6–13^. Besides, the use of amines or phosphines as monodentate directing groups with proximate sterically-hindered groups is another elegant strategy for the activation of *β*-C(sp^3^)–H or *β*-C(sp^2^)–H bonds via 4-membered palladacycles, rhodacycles or ruthenacycles (Fig. 1b, II and III)^14–18^. Most recently, a hydroxyl-containing bidentate directing group is devised to activate *β*-C(sp^3^)–H activation via 4-membered palladacycles, allowing various tertiary amines to be used as substrates (Fig. 1b, IV)^19^. Despite great progress, all these methods have to rely on precious metals such as Pd, Rh, and Ru, while the use of earth-abundant and inexpensive 3d-transition metal as catalysts to activate *β*-C–H bonds via 4-membered metallacycles still remains an elusive challenge^20–33^. Although larger-sized metallacycles such as 7- or 10-membered nickelacycles have been devised to enable *β*-C–H bond activation, these strategies often suffered reduced reactivity and limited scope of C–H bonds, for example, only more reactive *β*-C(sp^2^)–H bonds are compatible^34–36^. Thereby, the development of 3d metal-catalyzed unreactive *β*-C(sp^3^)−H bond activation via 4-membered metallacycles are highly desirable. Inspired by our previous work on C–C bond activation of cyclopropanes^37^, in which a phosphine oxide (PO)-ligated Ni–Al bimetallic catalyst can form a bicyclic structure to stabilize a 4-membered nickelacycle intermediate (Fig. 1c, left), we envisioned that the PO–Ni–Al catalytic system may also be used to activate proximate *β*-C(sp^3^)–H bond of formamides via a similar 4-membered nickelacycle intermediate (Fig. 1c, right)^38–48^, thus providing a 3d-metal-catalyzed [3 + 2] cycloaddition of two C–H bonds^49–54^.

Here, we show a 3d metal-catalyzed unreactive benzylic secondary *β*-C(sp^3^)–H bond activation via 4-membered nickelacycles, providing a series of *α,β*-unsaturated *γ*-lactams in up to 97% yield through a [3 + 2] cycloaddition of two C–H bonds of formamides with alkynes (Fig. 1d). In the reaction, a phosphine oxide (PO)-ligated Ni–Al bimetallic catalyst plays a crucial role in controlling catalytic reactivity and site-selectivity via the formation of a well-stabilized bicyclic nickelacycle. Preliminary experimental evidence and density functional theory (DFT) calculations reveal that the formyl C–H activation proceeds via ligand-to-ligand H transfer pathway, and the alkyne insertion into 4-membered nickelacycles is a turnover-limiting step of the reaction.

## Results

### Reaction optimization

*N*-benzylformamides (**1a**) and oct-4-yne (**2a**) were selected as model substrates on the basis of the following considerations: (1) formamides have proved to be good model substrates for acyl group-directed Ni-catalyzed *γ*-C–H bond activation^55–59^; (2) to eliminate the interference of more reactive *γ*- or *δ*-C(sp^3^)–H bonds, benzyl group containing only two secondary *β*-C(sp^3^)–H bonds was selected as a N-substituent; (3) a removable bulky 2,4,4-trimethylpentan-2-yl (TP) group was selected as another N-substituent to promote *β*-C(sp^3^)–H bond activation by steric repulsion and prevent from hydrocarbamoylation side reaction. Through systematic examination on various PO ligands, Al-based Lewis acids and other reaction parameters (Table 1), the optimal condition was eventually achieved: 10 mol% Ni(cod)_2_, 10 mol% *tert*-butyl diamine-derived PO (^*t*^Bu-DAPO), and 40 mol% AlEt_3_ at 120 °C. Under which, the desired product **3a** can be obtained in 81% yield (entry 1), proving that Ni–Al bimetallic catalyst can effectively promote the formation of 4-membered nickelacycles. Control experiments revealed that the combination of Ni(cod)_2_, ^*t*^Bu-DAPO, and AlEt_3_ was crucial, and the removal of any of them would inactivate the reaction (entries 2 and 3). Other Ni sources (entries 4 and 5) and traditional ligands such as phosphines (entries 6–8) and N-heterocyclic carbenes (entries 9 and 10) led to poor results. In addition, the reaction was rather sensitive to the structure of PO ligands, for example, other PO ligands gave either no products or low yields (entries 11–16), meaning an extremely narrow range of ligands. The superior reactivity of ^*t*^Bu-DAPO may be ascribed to the presence of two bulky *tert*-butyl groups that would affect the cone angle of phosphine ligand. Notably, the replacement of AlEt_3_ by other Lewis acids also resulted in low yields (entries 17–20), suggesting that the reactivity was also highly dependent on the structure and the acidity of Al-based Lewis acids.

**Table 1:** Reaction optimization


Entry		Deviation from the standard conditions	Yield (3a, %)
1		None	81
2		w/o Ni(cod)_2_ or AlEt_3_	0
3		w/o ^*t*^Bu-DAPO	0
4	Ni(cod)_2_ replaced by	NiBr_2_‧diglyme	0
5		NiBr_2_‧diglyme/Zn (50 mol%)	14
6	^*t*^Bu-DAPO replaced by	PPh_3_	0
7		PCy_3_	0
8		BINAP	0
9		IPr	0
10		IMes	0
11		Ph_2_P(O)H	0
12		**L**_**1**_	Trace
13		**L**_**2**_	12
14		**L**_**3**_	0
15		**L**_**4**_	0
16		**L**_**5**_	0
17	AlEt_3_ replaced by	AlMe_3_	45
18		AlEt_2_Cl	0
19		BPh_3_	0
20		ZnMe_2_	0


Reaction conditions: **1a** (0.2 mmol), **2a** (0.6 mmol), toluene (1.0 mL) under N_2_ for 2 h; yield was determined by ^1^H NMR using DMF as the internal standard. BINAP = 2,2’-bis(diphenylphosphino)-1,1’-binaphthalene, IPr = 1,3-bis(2,6-diisopropylphenyl)-2,3-dihydro-1*H*-imidazole. IMes = 1,3-dimesityl-2,3-dihydro-1*H*-imidazole.

### Scope of formamides and alkynes

**Fig. 2:**
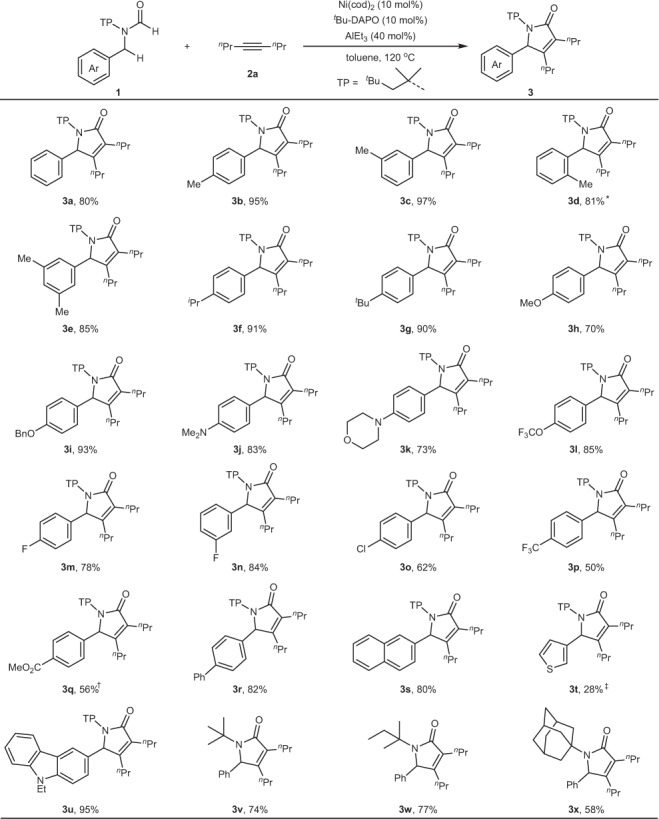
Scope of formamides. Reaction conditions: **1** (0.2 mmol), **2a** (0.6 mmol), toluene (1.0 mL) under N_2_ for 2 h; yield of isolated products. *AlEt_3_ (60 mol%). ^†^AlEt_3_ (80 mol%), 4 h. ^‡^AlEt_3_ (100 mol%). ^*n*^Pr = *n*-propyl. ^*t*^Bu = *tert*-butyl.

With the optimized reaction conditions established, we first investigated the scope of benzylformamides **1** (Fig. 2). Various electron-donating alkyl groups such as methyl group (**3b** to **3e**), isopropyl group (**3f**) and *tert*-butyl group (**3g**) at different positions of the aromatic ring were in general well compatible with the reaction, providing good to high yield. Different from alkyl substituents, electron-rich methoxy group (**3h**) gave a significantly decreased yield, which was attributed to the instability of C–O bond when the oxygen atom coordinated to Al-Lewis acid. To inhibit the coordination of oxygen atoms, bulkier benzyloxy group (**3i**) was examined and the yield was improved to 93%. In addition, due to similar coordination to Al-Lewis acid, electron-rich alkylamino groups also resulted in a little lower yield (**3j** and **3k**). Comparing with electron-donating groups, various electron-withdrawing groups such as CF_3_O (**3l**), F (**3m** and **3n**), Cl (**3o**), CF_3_ (**3p**), and carboxylate group (**3q**) were also well tolerated with the reaction, yet with slightly reduced yields, suggesting that the benzylic *β*-C(sp^3^)–H bond activation would proceed via an electrophilic metalation pathway. Except thiophenyl group with a low yield (**3t**), other (hetero)aryl groups such as biphenyl group (**3r**), naphthyl group (**3s**), and carbazolyl group (**3u**) were well compatible, providing the corresponding products in 80−95% yield. Notably, the presence of carboxylate (**3q**) and thiophenyl group (**3t**) resulted in the need of an excess of AlEt_3_, because these groups had relatively strong coordination with AlEt_3_. Besides aryl groups, general alkyl groups were also tested, however, these groups would result in undesired *γ*- or *δ*-C(sp^3^)−H activation (*vide infra*), further confirming that *β*-C(sp^3^)–H bonds are much less reactive than *γ*- or *δ*-C(sp^3^)–H bonds. In addition, other protecting groups such as *tert*-butyl (**3v**), *tert*-amyl (**3w**), and adamantly (**3x**) were also effective, providing the corresponding products in 58–77% yield, but these less bulky groups would result in partial activation of *γ*-C–H bonds of protecting groups via 5-membered nickelacycles.

**Fig. 3:**
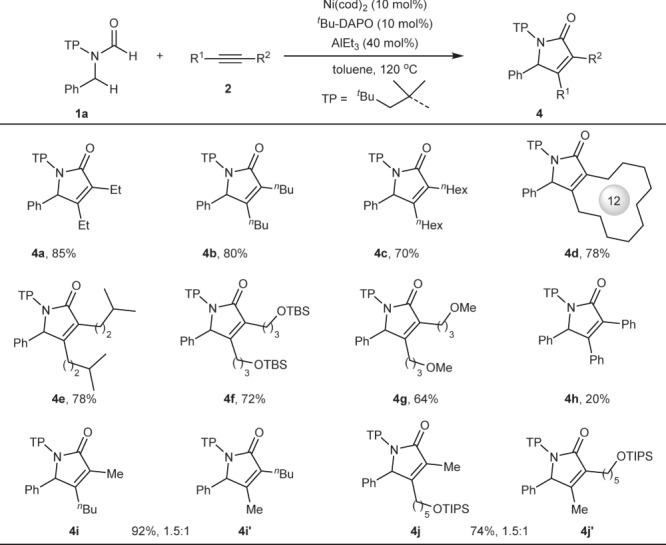
Scope of alkynes. Reaction conditions: **1a** (0.2 mmol), **2** (0.6 mmol), toluene (1.0 mL) under N_2_ for 2 h; yield of isolated products. ^*n*^Hex = *n*-hexyl. TBS = *tert*-butyldimethylsilyl. TIPS = triisopropylsilyl. ^*n*^Bu = *n*-butyl.

Next, the scope of alkynes was investigated under the optimal conditions (Fig. 3). Linear alkyl alkynes including ethyl (**4a**), *n*-butyl (**4b**), *n*-hexyl (**4c**) substituted alkynes and even cyclic alkyl alkynes (**4d**) were well tolerated, providing the corresponding products in 70−85% yield. In addition, alkynes with branched alkyl group (**4e**) or functional groups (**4f** and **4g**) also proved to be suitable substrates, affording the desired products in 64–78% yield. However, diphenyl alkynes or aryl alkyl alkynes were not very active in the reaction, delivering the desired product in low yield (**4h** in 20% yield). In these cases, insoluble precipitation was formed very quickly, resulting in a shutdown of the reaction with most of formamides recovered. We reasoned that insoluble precipitation could be complex of nickel with diaryl alkynes, for example, π-complex, or 5-membered nickelacycle that are generated by nickel with two alkynes^60,61^. In addition, non-symmetrical alkynes were also compatible, providing the corresponding products in 92% (**4i**/**4i′**) and 74% (**4j**/**4j′**) yield, respectively, with similar regioisomer ratio of 1.5:1, while further increasing steric bias between two substituents of alkynes would result in no reaction.

### Reaction utility

**Fig. 4:**
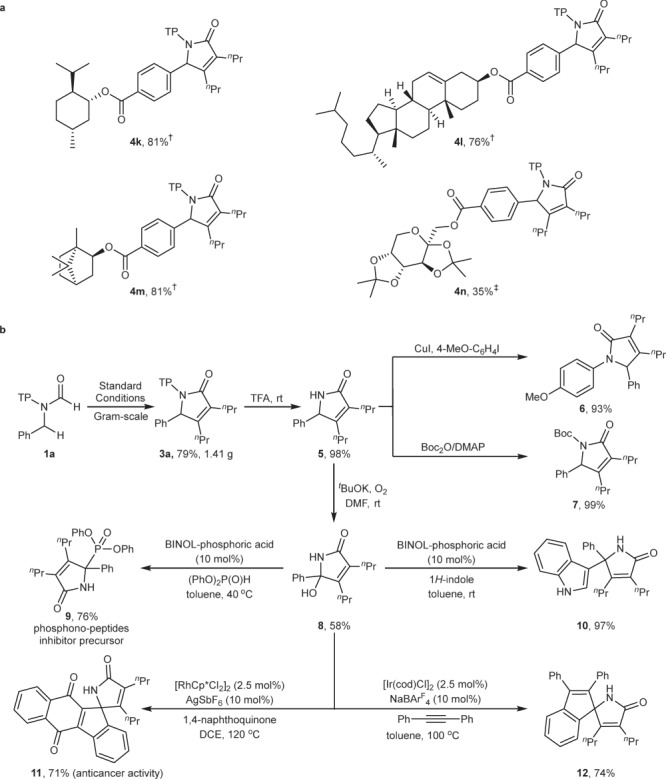
Synthetic utility. **a** Reaction of substrates bearing bioactive structural motifs under the optimal conditions with varying amounts of AlEt_3_. ^†^AlEt_3_ (80 mol%). ^‡^AlEt_3_ (100 mol%). **b** Gram-scale reaction and product transformations. TFA = trifluoroacetic acid. DMEDA = *N,N’*-dimethyl-1,2-ethanediamine. Boc_2_O = di(*tert*-butyl)carbonate. DMAP = 4-dimethylamino pyridine. BINOL = (*rac*)−1,1’-bi(2-naphthol). NaBAr^F^_4_ = sodium tetrakis(perfluorophenyl)borate.

To demonstrate the utility of the current method, a range of substrates containing bioactive structural motifs such as menthol (**4k**), cholesterol (**4l**), borneol (**4m**) and diacetonefructose (**4n**) have been examined, achieving 35–81% yield under the optimal conditions with varying amounts of AlEt_3_, owing to strong coordination of functional groups with AlEt_3_ (Fig. 4a). Then, a gram-scale reaction of **1a** and **2a** was conducted under the standard conditions, and product **3a** can be obtained without a significant loss of yield (Fig. 4b).

Upon treatment with trifluoroacetic acid under mild conditions, 2,4,4-trimethylpentan-2-yl (TP) protecting group can be easily removed, affording pyrrolidone **5** in 98% yield. Further treated with aryl iodide and (Boc)_2_O, compound **5** can be easily transformed into 4-methoxylphenyl-pyrrolidone **6** and Boc-pyrrolidone **7** in 93 and 99% yield, respectively. Under oxidative conditions, the benzylic C−H bond of compound **5** can be facilely oxidized, providing a versatile synthetic precursor, aminal **8**, in 58% yield. Aminal **8** underwent in situ dehydration to generate the corresponding imine, which was then attacked by diphenyl phosphonate and indole, providing tetra-substituted carbon-containing compound **9** in 76% yield and **10** in 97% yield, respectively^62^. In addition, the imine can play a directing group to facilitate Rh or Ir-catalyzed C−H activation, and subsequent cyclization with 1,4-dihydroquinone and alkyne afforded spiro compound **11** and **12**^63^.

### Mechanistic investigation

**Fig. 5:**
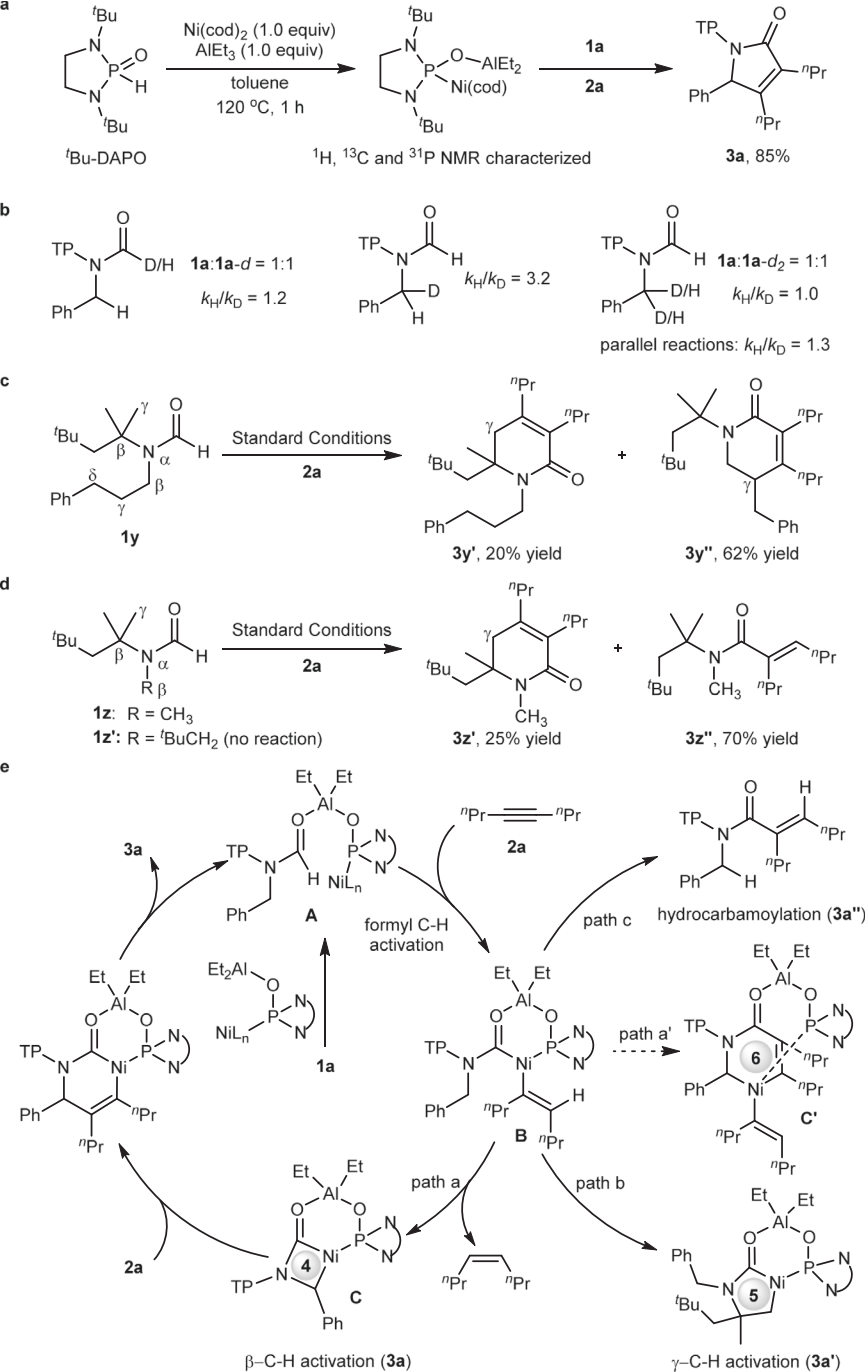
Mechanistic experiments. **a** ^*t*^Bu-DAPO–Ni–Al complex preparation and reactivity. **b** Kinetic isotope effect of formyl C–H bond (left), and intra- (middle) and intermolecular (right) kinetic isotope effect of benzylic C–H bond. **c** 3-Phenylpropyl group instead of benzyl group led to complete *γ*-C(sp^3^)–H bond activation, suggesting that *γ*-C(sp^3^)–H bond were more reactive than *β*-C(sp^3^)–H bonds in the reaction. **d** Methyl group and ^*t*^BuCH_2_ group instead of benzyl group led to no *β*-C(sp^3^)–H bond activation, suggesting the presence of benzyl group is critical to the reactivity. **e** Proposed mechanism.

To gain more insights into the mechanism, relevant mechanistic experiments were conducted. Firstly, ^*t*^Bu-DAPO-ligated Ni–Al bimetallic catalyst was prepared and characterized by ^1^H, ^13^C, and ^31^P NMR (Fig. 5a). When the complex was treated with substrates **1a** and **2a**, 85% yield of product **3a** can be obtained, suggesting ^*t*^Bu-DAPO–Ni–Al can catalyze the cycloaddition reaction. Secondly, deuterium-labeling experiment disclosed low isotope effect for formyl C–H bond (*k*_H_/*k*_D_ = 1.2) (Fig. 5b, left). Further intra- and intermolecular competitive experiments on the benzylic C–H bond generated the corresponding *k*_H_/*k*_D_ values, 3.2 and 1.0, respectively (Fig. 5b, middle and right). In addition, parallel reactions also revealed a low isotope effect for benzylic C–H bond (*k*_H_/*k*_D_ = 1.3). These results indicated that neither the formyl C–H bond cleavage nor the benzylic C–H bond cleavage would be involved into the turnover-limiting step, while high *k*_*H*_/*k*_*D*_ (3.2) meant that benzylic C−H cleavage could be an irreversible step. Thirdly, we also measured the rate order with regards to alkynes and achieved a first-order reaction for alkynes (see Supplementary Information, note 6), meaning that in turnover-limiting step, only one alkyne was involved, which suggested that the reaction occurred via a 4-membered nickelacycle without bearing an alkyne, otherwise, it would be a second-order reaction for alkynes when the reaction proceeds via a 6‐membered nickelacycle. Fourthly, 3-phenylpropyl group instead of benzyl group of **1a** resulted in complete *γ*-C(sp^3^)–H bond activation (Fig. 5c), providing the corresponding products **3y′** in 20% yield and **3y″** in 62% yield, without observation of *β*-C(sp^3^)–H bond activation. This result further confirmed that the activation of *β*-C(sp^3^)–H bond was more difficult than that of common *γ*-C(sp^3^)–H bond. In addition, simple methyl group instead of benzyl group of **1a** led to *γ*-C(sp^3^)–H bond activation product **3z′** in 25% yield and hydrocarbamoylation product **3z″** in 70% yield (Fig. 5d), while ^*t*^BuCH_2_ group instead of benzyl group gave no reaction. These results suggested that the presence of phenyl group was critical to *β*-C(sp^3^)–H bond activation. On the basis of these mechanistic experiments, possible reaction mechanisms were proposed in Fig. 5e. Substrate **1a** coordinates to the bimetallic catalyst, and then formyl C–H activation occurs to produce intermediate **B** that undergoes benzylic C–H activation to generate 4-membered nickelacycle **C** (path a). Subsequent alkyne insertion and reductive elimination furnish the desired product **3a** and regenerate the bimetallic catalyst. The intermediate **B** may undergo *γ*-C(sp^3^)–H bond activation to form more stable 5-membered nickelacycle (path b), which finally results in side product **3a′**. In addition, direct reductive elimination of the intermediate **B** would provide hydrocarbamoylation product **3a″** (path c). However, the pathway (path a′), wherein the intermediate **B** undergoes migratory insertion with an alkyne first, followed by benzylic C–H activation, to form a six-membered nickelacycle, would be excluded.

**Fig. 6:**
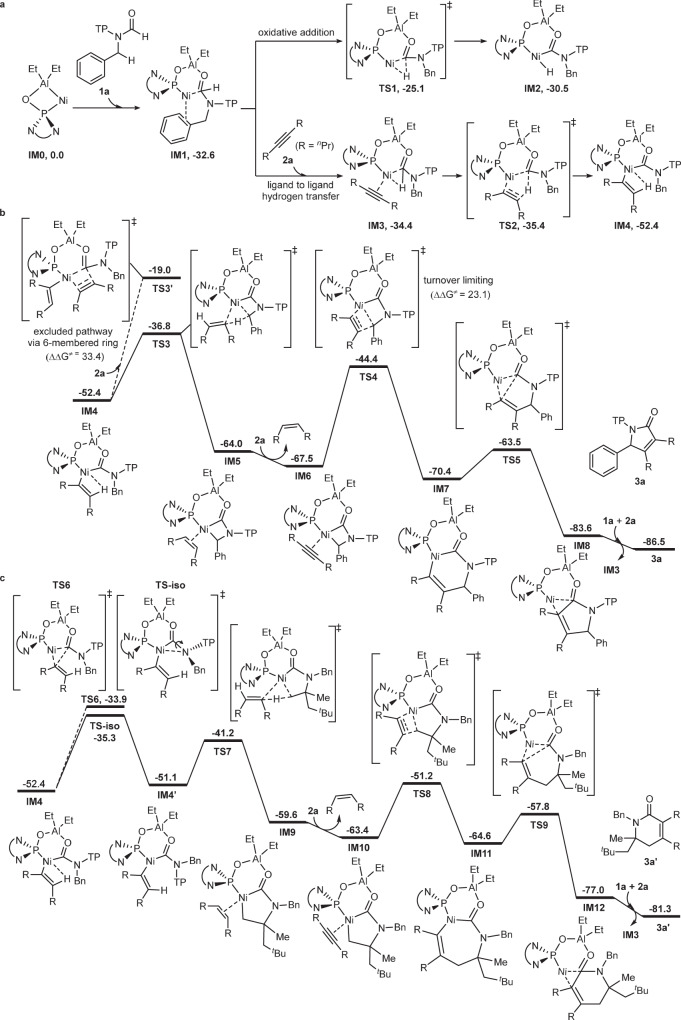
DFT calculations. The computations were performed at the B3LYP-D3(BJ)/def2- TZVPP-SMD_(toluene)_//B3LYP-D3(BJ)/def2-SVP level of theory. **a** Comparison between oxidative addition and ligand-to-ligand H transfer for formyl C–H bond activation, and the result shows that the latter is a preferred pathway. **b** Product-forming pathway via 4-membered nickelacycle. **c** Calculation on the activation Gibbs energy of *γ*-C(sp^3^)–H bond of TP group, indicating 17.1 kcal/mol (**TS-iso**), which is a little higher than the activation Gibbs energy of benzylic *β*-C(sp^3^)–H bond (15.6 kcal/mol, **TS3**).

Further DFT calculation shows that ligand-to-ligand H transfer pathway^64,65^ in the formyl C–H activation step is a more favorable process than oxidative addition pathway (Fig. 6a), which is in accordance with the observed kinetic isotope effect of formyl C–H bond.

The resulting intermediate **IM4** undergoes σ-bond metathesis, a ligand exchange, and a turnover-limiting alkyne insertion via **TS4** with an activation Gibbs energy of 23.1 kcal/mol (Fig. 6b), which is also in accordance with the observed kinetic isotope effect of benzylic C–H bond. Notably, DFT calculation shows that intermediate **IM4** would not undergo alkyne insertion before the step of benzylic C–H bond activation, otherwise an unstable 8-membered ring intermediate would be formed, resulting in a high energy barrier (33.4 kcal/mol, **TS3’**). In addition, as shown in Fig. 6c, DFT calculation indicates that the activation Gibbs energy of *γ*-C(sp^3^)–H bond of TP group is 17.1 kcal/mol (**TS-iso**), which is a little higher than the activation Gibbs energy of *β*-C(sp^3^)–H bond (15.6 kcal/mol, **TS3**). This result is also in accordance with the observed selective activation of benzylic *β*-C(sp^3^)–H bond over *γ*-C(sp^3^)–H bonds of TP group.

In summary, we have developed a Ni-catalyzed benzylic *β*-C(sp^3^)–H bond activation of formamides with alkynes via highly-strained 4-membered nickelacycle, providing a series of *α,β*-unsaturated *γ*-lactams in up to 97% yield. Bulky protecting group can be easily removed for further elaboration of products. In addition, the formed *γ*-lactams not only widely exist in bioactive molecules, but also proved to be versatile synthetic precursors. Mechanistic experiments and DFT calculations showed that phosphine oxide-ligated Ni–Al bimetallic catalyst along with removable bulky N-protecting group play critical roles in controlling good efficiency and good selectivity. This method should find wider applications in other 3d metal-involved C–H metalation via 4-membered metallacycles.

## Methods

### General procedure benzylic *β*-C(sp^3^)–H bond

To a 15 mL oven-dried tube were added ^*t*^Bu-DAPO (4.4 mg, 10 mol%), Ni(cod)_2_ (5.5 mg, 10 mol%), dry degassed toluene (1.0 mL), benzyl formamide (0.2 mmol), alkyne (0.6 mmol) and AlEt_3_ (1.0 M in toluene, 80 μL, 40 mol%) sequentially in an N_2_-filled glove-box. The tube was sealed and removed out of the glove-box. After heated at 120 °C in a preheated dry block heater for 2 h, the mixture was cooled to r.t., quenched with 0.1 mL H_2_O, filtered through a short plug of silica gel (DCM as the eluent) and concentrated in vacuo to afford the crude product. Further purification by flash column chromatography on silica gel (eluting with EtOAc/n-hexane) gave the pure product.

## Supplementary information

Supplementary Information

Description of Additional Supplementary Files

Supplementary Data 1

## Data Availability

The authors declare that the data supporting the findings of this study are available within the article and its Supplementary Information file. For the experimental procedures, computational details, additional computational results and data of NMR see Supplementary Methods in Supplementary Information file. For computed energies and cartesian coordinates of the stationary points see Supplementary Data 1. The X-ray crystallographic coordinates for structures reported in this study have been deposited at the Cambridge Crystallographic Data Centre (CCDC), under deposition number CCDC 2089375. These data can be obtained free of charge from The Cambridge Crystallographic Data Centre via https://www.ccdc.cam.ac.uk/MyStructures/. Data is available from the corresponding authors upon request.
