# Supplementary material for: Ni-catalyzed benzylic β-C(sp3)–H bond activation of formamides
Source: Nat Commun. 2022 Dec 22;13:7892. doi: 10.1038/s41467-022-35541-6 (PMC9780214; doi:10.1038/s41467-022-35541-6)
Supplement: Supplementary file 1 — Supplementary Information [file 41467_2022_35541_MOESM1_ESM.pdf]

Supplementary Information

Ni-catalyzed Benzylic β-C(sp^3)−H Bond Activation of Formamides

Wang et al.

Table of Contents

Supplementary Methods 2

General Information 2

Supplementary Note 1 2

Substrate Preparation 2

Supplementary Note 2 13

Reaction Optimization 13

Supplementary Note 3 17

General Procedure for [3+2] Annulation 17

Supplementary Note 4 33

Crystal Structure Information of 3k 33

Supplementary Note 5 34

Gram-scale reaction and product transformation 34

Supplementary Note 6 38

Mechanistic experiments 38

Supplementary Note 7 48

DFT Calculations 48

Supplementary Figures 50

Supplementary references 136

Supplementary Methods

General Information

Unless stated otherwise, all reactions were conducted under N_2 atmosphere. All solvents were received from commercial sources without further purification. Melting points were measured on X-4B microscope melting point apparatus and uncorrected. Thin-layer chromatography (TLC) was performed by UV absorbance (254 nm). 200–300 mesh silica gel was used for column chromatography separation. NMR spectra were recorded on Bruker AV 400 spectrometer at 400 MHz (^{1}H NMR), 100 MHz (^{13}C NMR), 376 MHz (^{19}F NMR) and 162 MHz (^{31}P NMR). Proton and carbon chemical shifts are reported relative to the solvent used as an internal reference ($CDCl_3$: δ_H = 7.26 ppm; δ_C = 77.16 ppm). All coupling constants (*J* values) were reported in Hertz (Hz). Multiplicities are reported as follows: singlet (s), doublet (d), doublet of doublets (dd), triplet (t), triplet of doublets (td), quartet (q), and multiplet (m). High resolution mass spectra (HRMS) were recorded on an Agilent 6520 Q-TOF LC/MS with Electron Spray Ionization (ESI) resource. Chiral high-performance liquid chromatography (HPLC) analysis was performed using an Agilent 1260 with commercial ChiralPak 4.6 × 250 mm columns. Infrared spectra (IR) were obtained on a JASCO FT/IR-4000 spectrometer, and the absorptions have been reported in reciprocal centimeters with the following relative intensities: s (strong), m (medium), or w (weak). Optical rotations were determined by a Rudolph Autopol VI polarimeter. Single crystal X-ray diffraction data were collected on Rigaku Saturn70 diffractometer. Commercially available reagents were used as received. Non-commercially available substrates were synthesized following reported protocols. Phosphines oxides ligands were prepared according to the reported procedure in literature[1-5].

Supplementary Note 1

Substrate Preparation

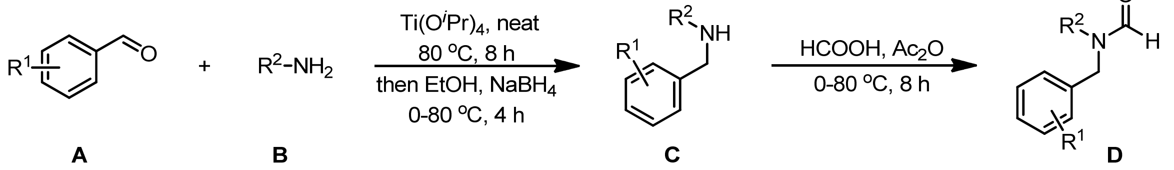

A solution of aldehyde **A** (5.0 mmol), amine **B** (5.5 mmol) and Ti(O^{i}Pr)$_4$ (3.0 mL) was stirred for 8 hours at 80 °C under nitrogen atmosphere. After cooled to 0 °C, the mixture was diluted with anhydrous ethanol (40 mL), to which sodium borohydride (567.4 mg, 15 mmol) was added portion wise over 10 mins. The resulting mixture was stirred for an additional 4 hours at 80 °C, cooled to room temperature, and quenched with 2.0 M sodium hydroxide. The white suspension was filtered and washed with ethyl acetate. The filtrate was extracted with ethyl acetate and the combined organic

extracts were washed with brine, dried over anhydrous magnesium sulphate and concentrated in vacuo. The crude product was directly used for next step without further purification.

To a mixture of formic acid (1.9 mL, 50 mmol), acetic anhydride (4.7 mL, 50 mmol) was added crude amine **C** at 0 °C, and the resulting mixture was heated to 80 °C and stirred for an additional 8 hours. After cooled to room temperature, the solution was concentrated under reduced pressure, and the residue was purified by column chromatography on silica gel.

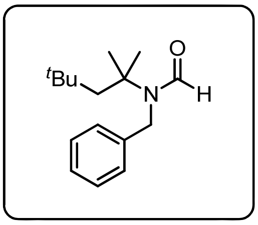

***N*-Benzyl-*N*-(2,4,4-trimethylpentan-2-yl)formamide (1a)**
White solid (54% yield for two steps). m.p. 45-47 °C. Rf 0.51 (SiO_2, hexane/EtOAc = 6/1). **^{1}H NMR** (400 MHz, $CDCl_3$) δ 8.68 (s, 1H), 7.30 – 7.26 (m, 2H), 7.22 – 7.18 (m, 3H), 4.60 (s, 2H), 1.66 (s, 2H), 1.38 (s, 6H), 0.99 (s, 9H). **^{13}C NMR** (100 MHz, $CDCl_3$) δ 163.4, 138.9, 128.5, 127.0, 126.8, 59.7, 53.4, 44.8, 31.8, 31.5, 30.4. **HRMS (ESI)** *m/z:* $[M+H]^+$ Calcd. for $C_{16}H_{26}NO$ 248.2009; Found 248.2007.

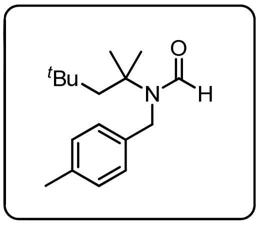

***N*-(4-Methylbenzyl)-*N*-(2,4,4-trimethylpentan-2-yl)formamide (1b)**
White solid (43% yield for two steps). m.p. 59-61 °C. Rf 0.51 (SiO_2, hexane/EtOAc = 6/1).**^{1}H NMR** (400 MHz, $CDCl_3$) δ 8.66 (s, 1H), 7.23 – 6.94 (m, 4H), 4.55 (s, 2H), 2.30 (s, 3H), 1.65 (s, 2H), 1.37 (s, 6H), 0.98 (s, 9H). **^{13}C NMR** (100 MHz, $CDCl_3$) δ 163.1, 136.3, 136.0, 129.2, 127.1, 59.6, 53.4, 44.6, 31.8, 31.6, 30.5, 21.2. **HRMS (ESI)** *m/z:* $[M+H]^+$ Calcd. for $C_{17}H_{28}NO$ 262.2165; Found 262.2163.

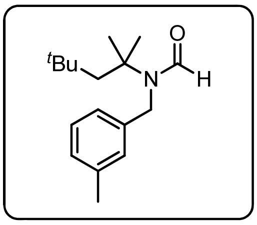

***N*-(3-Methylbenzyl)-*N*-(2,4,4-trimethylpentan-2-yl)formamide (1c)**
White solid (63% yield for two steps). m.p. 77-79 °C. Rf 0.50 (SiO_2, hexane/EtOAc = 6/1). **^{1}H NMR** (400 MHz, $CDCl_3$) δ 8.68 (s, 1H), 7.16 (t, J = 7.5 Hz, 1H), 7.06 – 6.96 (m, 3H), 4.57 (s, 2H), 2.31 (s, 3H), 1.65 (s, 2H), 1.38 (s, 6H), 0.98 (s, 9H). **^{13}C NMR** (100 MHz, $CDCl_3$) δ 163.3, 138.8, 138.1,

128.4, 127.7, 127.6, 124.1, 59.7, 53.4, 44.8, 31.8, 31.6, 30.4, 21.6. **HRMS (ESI)** *m/z:* $[M+H]^+$ Calcd. for $C_{17}H_{28}NO$ 262.2165; Found 262.2163.

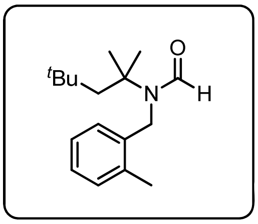

N-(2-Methylbenzyl)-N-(2,4,4-trimethylpentan-2-yl)formamide (1d)

White solid (47% yield for two steps). m.p. 71-73 °C. Rf 0.50 (SiO_2, hexane/EtOAc = 6/1). **^{1}H NMR** (400 MHz, $CDCl_3$) δ 8.74 (s, 1H), 7.18 – 7.08 (m, 3H), 7.00 (d, J = 6.4 Hz, 1H), 4.50 (s, 2H), 2.33 (s, 3H), 1.67 (s, 2H), 1.41 (s, 6H), 1.02 (s, 9H). **^{13}C NMR** (100 MHz, $CDCl_3$) δ 163.3, 135.7, 134.2, 130.2, 126.5, 126.1, 125.7, 59.5, 53.7, 42.7, 31.9, 31.6, 29.9, 19.3. **HRMS (ESI)** *m/z:* $[M+H]^+$ Calcd. for $C_{17}H_{28}NO$ 262.2165; Found 262.2162.

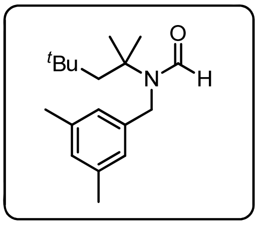

N-(3,5-Dimethylbenzyl)-N-(2,4,4-trimethylpentan-2-yl)formamide (1e)

Yollow solid (48% yield for two steps). m.p. 105-107 °C. Rf 0.51 (SiO_2, hexane/EtOAc = 6/1). **^{1}H NMR** (400 MHz, $CDCl_3$) δ 8.67 (s, 1H), 6.8 – 6.81 (m, 3H), 4.53 (s, 2H), 2.27 (s, 6H), 1.65 (s, 2H), 1.39 (s, 6H), 0.99 (s, 9H). **^{13}C NMR** (100 MHz, $CDCl_3$) δ 163.1, 138.9, 138.0, 128.5, 124.8, 59.6, 53.5, 44.7, 31.9, 31.6, 30.5, 21.5. **HRMS (ESI)** *m/z:* $[M+H]^+$ Calcd. for $C_{18}H_{30}NO$ 276.2322; Found 276.2320.

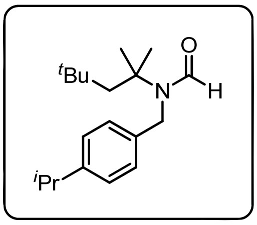

N-(4-Isopropylbenzyl)-N-(2,4,4-trimethylpentan-2-yl)formamide (1f)

White solid (79% yield for two steps). m.p. 71-73 °C. Rf 0.49 (SiO_2, hexane/EtOAc = 6/1).**^{1}H NMR** (400 MHz, $CDCl_3$) δ 8.66 (s, 1H), 7.13 (s, 4H), 4.56 (s, 2H), 2.86 (dt, J = 13.8, 6.9 Hz, 1H), 1.64 (s, 2H), 1.38 (s, 6H), 1.21 (d, J = 6.9 Hz, 6H), 0.98 (s, 9H). **^{13}C NMR** (100 MHz, $CDCl_3$) δ 163.0, 147.3, 136.2, 127.0, 126.5, 59.5, 53.4, 44.5, 33.8, 31.8, 31.6, 30.4, 24.1. **HRMS (ESI)** *m/z:* $[M+H]^+$ Calcd. for $C_{19}H_{32}NO$ 290.2478; Found 290.2475.

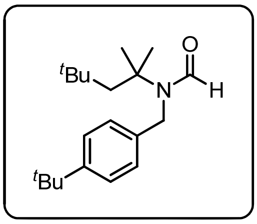

N-(4-(*tert*-Butyl)benzyl)-N-(2,4,4-trimethylpentan-2-yl)formamide (1g)

White solid (79% yield for two steps). m.p. 98-100 °C. Rf 0.51 (SiO_2, hexane/EtOAc = 6/1). **^{1}H NMR** (400 MHz, $CDCl_3$) δ 8.66 (s, 1H), 7.29 (d, J = 8.3 Hz, 2H), 7.14 (d, J = 8.2 Hz, 2H), 4.56 (s, 2H), 1.65 (s, 2H), 1.39 (s, 6H), 1.28 (s, 9H), 0.98 (s, 9H). **^{13}C NMR** (100 MHz, $CDCl_3$) δ 163.0, 149.6, 135.8, 126.7, 125.4, 59.5, 53.4, 44.5, 34.5, 31.8, 31.6, 31.5, 30.4. **HRMS (ESI)** *m/z:* $[M+H]^+$ Calcd. for $C_{20}H_{34}NO$ 304.2635; Found 304.2630.

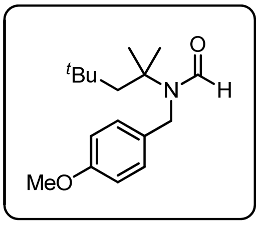

N-(4-Methoxybenzyl)-N-(2,4,4-trimethylpentan-2-yl)formamide (1h)

White solid (78% yield for two steps). m.p. 53-55 °C. Rf 0.43 (SiO_2, hexane/EtOAc = 6/1). **^{1}H NMR** (400 MHz, $CDCl_3$) δ 8.65 (s, 1H), 7.15 (d, J = 8.5 Hz, 2H), 6.81 (d, J = 8.6 Hz, 2H), 4.53 (s, 2H), 3.76 (s, 3H), 1.64 (s, 2H), 1.37 (s, 7H), 0.97 (s, 9H). **^{13}C NMR** (100 MHz, $CDCl_3$) δ 163.3, 158.5, 131.1, 128.5, 113.9, 59.7, 55.3, 53.4, 44.2, 31.8, 31.6, 30.4. **HRMS (ESI)** *m/z:* $[M+H]^+$ Calcd. for $C_{17}H_{28}NO_2$ 278.2115; Found 278.2113.

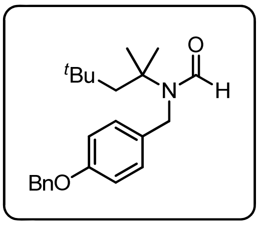

N-(4-(Benzyloxy)benzyl)-N-(2,4,4-trimethylpentan-2-yl)formamide (1i)

White solid (51% yield for two steps). m.p. 118-120 °C. Rf 0.41 (SiO_2, hexane/EtOAc = 6/1). **^{1}H NMR** (400 MHz, $CDCl_3$) δ 8.65 (s, 1H), 7.43-7.30 (m, 5H), 7.16 (d, J = 8.4 Hz, 2H), 6.90 (d, J = 8.5 Hz, 2H), 5.03 (s, 2H), 4.54 (s, 2H), 1.65 (s, 2H), 1.38 (s, 6H), 0.98 (s, 9H). **^{13}C NMR** (100 MHz, $CDCl_3$) δ 163.1, 157.7, 137.2, 131.5, 128.7, 128.4, 128.0, 127.6, 114.8, 70.1, 59.6, 53.4, 44.2, 31.8, 31.6, 30.5. **HRMS (ESI)** *m/z:* $[M+H]^+$ Calcd. for $C_{23}H_{32}NO_2$ 354.2428; Found 354.2425.

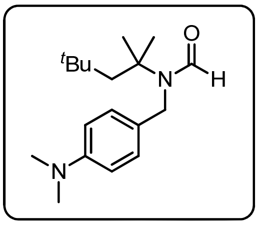

N-(4-(Dimethylamino)benzyl)-N-(2,4,4-trimethylpentan-2-yl)formamide (1j)
White solid (34% yield for two steps). m.p. 68-70 °C. Rf 0.41 (SiO_2, hexane/EtOAc = 6/1). **^{1}H NMR** (400 MHz, $CDCl_3$) δ 8.64 (s, 1H), 7.11 (d, J = 8.4 Hz, 2H), 6.66 (d, J = 8.3 Hz, 2H), 4.51 (s, 2H), 2.90 (s, 6H), 1.65 (s, 2H), 1.37 (s, 7H), 0.98 (s, 9H). **^{13}C NMR** (100 MHz, $CDCl_3$) δ 163.2, 149.7, 128.3, 127.1, 112.8, 59.5, 53.5, 44.2, 40.8, 31.8, 31.6, 30.5. **HRMS (ESI)** *m/z:* $[M+H]^+$ Calcd. for $C_{18}H_{31}N_2O$ 291.2431; Found 291.2427.

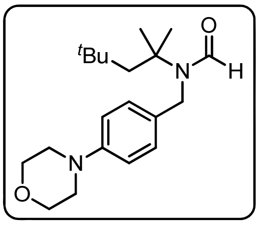

N-(4-Morpholinobenzyl)-N-(2,4,4-trimethylpentan-2-yl)formamide (1k)
White solid (65% yield for two steps). m.p. 86-88 °C. Rf 0.38 (SiO_2, hexane/EtOAc = 6/1). **^{1}H NMR** (400 MHz, $CDCl_3$) δ 8.64 (s, 1H), 7.12 (d, J = 8.6 Hz, 2H), 6.81 (d, J = 8.7 Hz, 2H), 4.50 (s, 2H), 3.88 – 3.76 (m, 4H), 3.16 – 2.95 (m, 4H), 1.63 (s, 2H), 1.35 (s, 6H), 0.96 (s, 9H). **^{13}C NMR** (100 MHz, $CDCl_3$) δ 163.3, 150.1, 130.4, 128.2, 115.7, 66.9, 59.6, 53.3, 49.5, 44.2, 31.7, 31.5, 30.4. **HRMS (ESI)** *m/z:* $[M+H]^+$ Calcd. for $C_{20}H_{33}N_2O_2$ 333.2537; Found 333.2534.

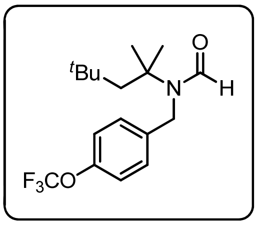

N-(4-(Trifluoromethoxy)benzyl)-N-(2,4,4-trimethylpentan-2-yl)formamide (1l)
White solid (86% yield for two steps). m.p. 57-59 °C. Rf 0.42 (SiO_2, hexane/EtOAc = 6/1). **^{1}H NMR** (400 MHz, $CDCl_3$) δ 8.66 (s, 1H), 7.25 (d, J = 8.9 Hz, 2H), 7.13 (d, J = 8.0 Hz, 2H), 4.58 (s, 2H), 1.65 (s, 2H), 1.39 (s, 6H), 0.99 (s, 9H). **^{13}C NMR** (100 MHz, $CDCl_3$) δ 163.1, 148.2, 137.8, 128.5, 121.1, 120.6 (q, J = 256 Hz), 59.7, 53.4, 44.3, 31.9, 31.6, 30.4. **^{19}F NMR** (376 MHz, $CDCl_3$) δ -63.5. **HRMS (ESI)** *m/z:* $[M+H]^+$ Calcd. for $C_{17}H_{25}F_3NO_2$ 332.1832; Found 332.1828.

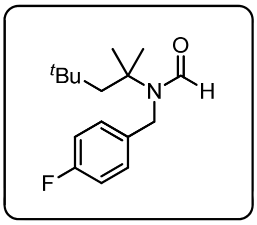

N-(4-Fluorobenzyl)-N-(2,4,4-trimethylpentan-2-yl)formamide (1m)
White solid (86% yield for two steps). m.p. 67-69 °C. Rf 0.48 (SiO_2, hexane/EtOAc = 6/1). **^{1}H NMR** (400 MHz, $CDCl_3$) δ 8.65 (s, 1H), 7.23 – 7.11 (m, 2H), 6.97 (t, J = 7.7 Hz, 2H), 4.55 (s, 2H), 1.65 (s, 2H), 1.37 (s, 6H), 0.98 (s, 9H). **^{13}C NMR** (100 MHz, $CDCl_3$) δ 163.1, 161.8 (d, J = 243.0 Hz), 134.8 (d, J = 3.0 Hz), 128.7 (d, 8.0 Hz), 115.3 (d, J = 22.0 Hz), 59.6, 53.2, 44.1, 31.8, 31.5, 30.4. **^{19}F NMR**

(376 MHz, $CDCl_3$) δ -116.3. **HRMS (ESI)** *m/z:* $[M+H]^+$ Calcd. for $C_{16}H_{25}FNO$ 266.1915; Found 266.1912.

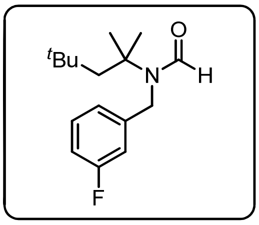

***N*-(3-Fluorobenzyl)-*N*-(2,4,4-trimethylpentan-2-yl)formamide (1n)**

White solid (81% yield for two steps). m.p. 44-46 °C. Rf 0.48 (SiO_2, hexane/EtOAc = 6/1). **^{1}H NMR** (400 MHz, $CDCl_3$) δ 8.66 (s, 1H), 7.23 (td, J = 7.9, 6.0 Hz, 1H), 6.98 (d, J = 7.7 Hz, 1H), 6.94 – 6.85 (m, 2H), 4.56 (s, 2H), 1.63 (s, 2H), 1.37 (s, 7H), 0.97 (s, 9H). **^{13}C NMR** (100 MHz, $CDCl_3$) δ 163.1, 163.0 (d, J = 245.0 Hz), 141.7 (d, J = 7.0 Hz), 130.0 (d, J = 8.0 Hz), 122.6 (d, J = 3.0 Hz), 114.1 (d, J = 22.0 Hz), 113.7 (d, J = 21.0 Hz), 59.7, 53.3, 44.4, 31.8, 31.5, 30.3. **^{19}F NMR** (376 MHz, $CDCl_3$) δ -118.7. **HRMS (ESI)** *m/z:* $[M+H]^+$ Calcd. for $C_{16}H_{25}FNO$ 266.1915; Found 266.1912.

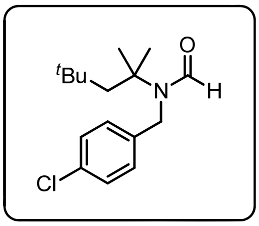

***N*-(4-Chlorobenzyl)-*N*-(2,4,4-trimethylpentan-2-yl)formamide (1o)**

White solid (67% yield for two steps). m.p. 65-67 °C. Rf 0.43 (SiO_2, hexane/EtOAc = 6/1). **^{1}H NMR** (400 MHz, $CDCl_3$) δ 8.65 (s, 1H), 7.29 – 7.21 (m, 2H), 7.16 – 7.14 (m, 2H), 4.54 (s, 2H), 1.63 (s, 2H), 1.36 (s, 6H), 0.97 (s, 9H). **^{13}C NMR** (100 MHz, $CDCl_3$) δ 163.2, 137.6, 132.6, 128.6, 128.6, 59.7, 53.3, 44.3, 31.8, 31.5, 30.4. **HRMS (ESI)** *m/z:* $[M+H]^+$ Calcd. for $C_{16}H_{25}ClNO$ 282.1619; Found 282.1615.

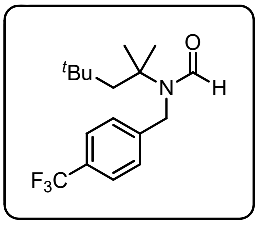

***N*-(4-(Trifluoromethyl)benzyl)-*N*-(2,4,4-trimethylpentan-2-yl)formamide (1p)**

Yellowish brown solid (73% yield for two steps). m.p. 78-80 °C. Rf 0.44 (SiO_2, hexane/EtOAc = 6/1). **^{1}H NMR** (400 MHz, $CDCl_3$) δ 8.68 (s, 1H), 7.54 (d, J = 7.8 Hz, 2H), 7.32 (d, J = 7.7 Hz, 2H), 4.62 (s, 2H), 1.65 (s, 3H), 1.38 (s, 6H), 0.99 (s, 9H). **^{13}C NMR** (100 MHz, $CDCl_3$) δ 163.1, 143.1, 129.2 (q, J = 32 Hz), 127.3, 125.5 (q, J = 3.8 Hz), 124.3 (q, J = 270 Hz), 59.7, 53.2, 44.6, 31.8, 31.5, 30.3. **^{19}F NMR** (376 MHz, $CDCl_3$) δ -68.0. **HRMS (ESI)** *m/z:* $[M+H]^+$ Calcd. for $C_{17}H_{25}F_3NO$ 316.1883; Found 316.1881.

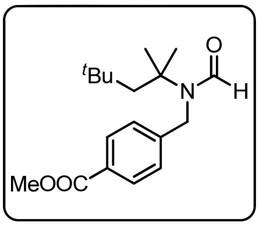

Methyl 4-((*N*-(2,4,4-trimethylpentan-2-yl)formamido)methyl)benzoate (1q)
White solid (36% yield for two steps). m.p. 88-90 °C. Rf 0.42 (SiO_2, hexane/EtOAc = 6/1). **^{1}H NMR** (400 MHz, $CDCl_3$) δ 8.66 (s, 1H), 7.94 (d, J = 8.3 Hz, 2H), 7.29 – 7.24 (m, 2H), 4.61 (s, 2H), 3.87 (s, 3H), 1.63 (s, 2H), 1.35 (s, 6H), 0.96 (s, 9H). **^{13}C NMR** (100 MHz, $CDCl_3$) δ 167.0, 163.1, 144.3, 129.9, 128.8, 127.0, 59.7, 53.2, 52.1, 44.7, 31.8, 31.5, 30.3. **HRMS (ESI)** *m/z:* $[M+H]^+$ Calcd. for $C_{18}H_{28}NO_3$ 306.2064; Found 306.2060.

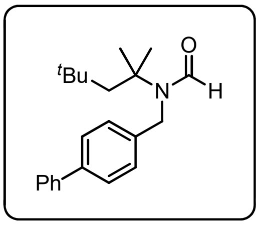

***N*-([1,1'-Biphenyl]-4-ylmethyl)-*N*-(2,4,4-trimethylpentan-2-yl)formamide (1r)**
White solid (67% yield for two steps). m.p. 121-123 °C. Rf 0.51 (SiO_2, hexane/EtOAc = 6/1). **^{1}H NMR** (400 MHz, $CDCl_3$) δ 8.71 (s, 1H), 7.61 – 7.55 (m, 2H), 7.53 – 7.51 (m, 2H), 7.44 – 7.41 (m, 2H), 7.35 – 7.32 (m, 1H), 7.31 – 7.28 (m, 2H), 4.64 (s, 2H), 1.68 (s, 2H), 1.42 (s, 6H), 1.01 (s, 9H). **^{13}C NMR** (100 MHz, $CDCl_3$) δ 163.1, 141.0, 139.7, 138.1, 128.8, 127.5, 127.2, 127.1, 59.6, 53.4, 44.6, 31.9, 31.6, 30.5. **HRMS (ESI)** *m/z:* $[M+H]^+$ Calcd. for $C_{22}H_{30}NO$ 324.2322; Found 324.2319.

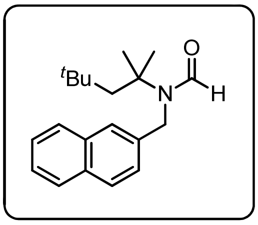

***N*-(Naphthalen-2-ylmethyl)-*N*-(2,4,4-trimethylpentan-2-yl)formamide (1s)**
White solid (45% yield for two steps). m.p. 114-116 °C. Rf 0.50 (SiO_2, hexane/EtOAc = 6/1). **^{1}H NMR** (400 MHz, $CDCl_3$) δ 8.76 (s, 1H), 7.81 – 7.77 (m, 3H), 7.65 (s, 1H), 7.49 – 7.42 (m, 2H), 7.40 – 7.38 (m, 1H), 4.77 (s, 2H), 1.70 (s, 2H), 1.40 (s, 6H), 1.02 (s, 9H). **^{13}C NMR** (100 MHz, $CDCl_3$) δ 163.2, 136.7, 133.5, 132.6, 128.3, 127.8, 127.7, 126.1, 125.7, 125.6, 125.6, 59.7, 53.4, 45.0, 31.9, 31.6, 30.5. **HRMS (ESI)** *m/z:* $[M+H]^+$ Calcd. for $C_{20}H_{28}NO$ 298.2165; Found 298.2164.

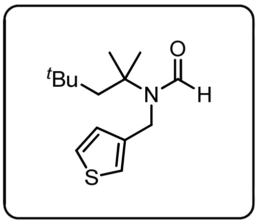

***N*-(Thiophen-3-ylmethyl)-*N*-(2,4,4-trimethylpentan-2-yl)formamide (1t)**
Light yellow oil (47% yield for two steps). Rf 0.35 (SiO_2, hexane/EtOAc = 6/1). **^{1}H NMR** (400 MHz, $CDCl_3$) δ 8.59 (s, 1H), 7.22 (s, 1H), 7.09 (s, 1H), 7.02 (d, J = 4.7 Hz, 1H), 4.54 (s, 2H), 1.64 (s, 2H), 1.40 (s, 6H), 0.96 (s, 9H). **^{13}C NMR** (100 MHz, $CDCl_3$) δ 162.9, 140.0, 127.9, 125.6, 122.0, 59.6, 53.2, 40.4, 31.8, 31.6, 30.4. **HRMS (ESI)** *m/z:* $[M+H]^+$ Calcd. for $C_{14}H_{24}NOS$ 254.1573; Found 254.1572.

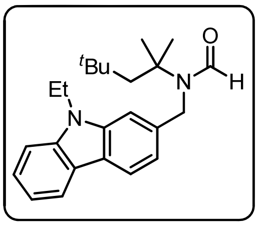

***N*-((9-Ethyl-9H-carbazol-2-yl)methyl)-*N*-(2,4,4-trimethylpentan-2-yl)formamide (1u)**
White solid (24% yield for two steps). m.p. 143-145 °C. Rf 0.34 (SiO_2, hexane/EtOAc = 6/1). **^{1}H NMR** (400 MHz, $CDCl_3$) δ 8.76 (s, 1H), 8.09 (d, J = 7.7 Hz, 1H), 7.97 (s, 1H), 7.49 – 7.43 (m, 1H), 7.39 (d, J = 8.2 Hz, 2H), 7.32 (d, J = 8.4 Hz, 1H), 7.24 – 7.19 (m, 1H), 4.81 (s, 2H), 4.34 (q, J = 7.2 Hz, 2H), 1.72 (s, 2H), 1.45 – 1.38 (m, 9H), 1.02 (s, 9H). **^{13}C NMR** (100 MHz, $CDCl_3$) δ 163.5, 140.3, 139.1, 129.7, 125.7, 125.4, 123.0, 122.8, 120.6, 119.2, 118.8, 108.5, 108.4, 59.8, 53.5, 45.0, 37.7, 31.9, 31.6, 30.6, 13.9. **HRMS (ESI)** *m/z:* $[M+H]^+$ Calcd. for $C_{24}H_{33}N_2O$ 365.2587; Found 365.2586.

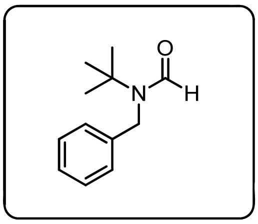

***N*-Benzyl-*N*-(*tert*-butyl)formamide[6] (1v)**
Colorless oil (90% yield, one step synthesis from commercially available *N*-(*tert*-butyl)benzylamine). Rf 0.50 (SiO_2, hexane/EtOAc = 6/1). **^{1}H NMR** (400 MHz, $CDCl_3$) δ 8.70 (s, 1H), 7.36 – 7.26 (m, 1H), 7.25 – 7.14 (m, 2H), 4.64 (s, 2H), 1.35 (s, 9H). **^{13}C NMR** (100 MHz, $CDCl_3$) δ 162.7, 138.9, 128.5, 126.9, 126.8, 56.1, 44.1, 30.1.

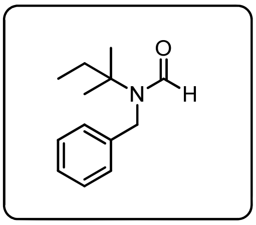

***N*-Benzyl-*N*-(*tert*-pentyl)formamide (1w)**
Colorless oil (87% yield for two steps). Rf 0.49 (SiO_2, hexane/EtOAc = 6/1). **^{1}H NMR** (400 MHz, $CDCl_3$) δ 8.60 (s, 1H), 7.33 – 7.25 (m, 2H), 7.25 – 7.17 (m, 3H), 4.59 (s, 2H), 1.64 (q, J = 7.4 Hz, 2H), 1.29 (s, 6H), 0.81 (t, J = 7.4 Hz, 3H). **^{13}C NMR** (100 MHz, $CDCl_3$) δ 163.2, 138.9, 128.5, 127.1, 126.9, 58.9, 44.2, 34.0, 27.8, 8.3. **HRMS (ESI)** *m/z:* $[M+H]^+$ Calcd. for $C_{13}H_{20}NO$ 206.1539; Found 206.1541.

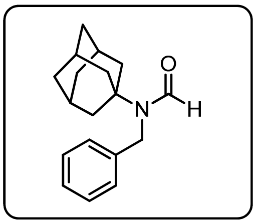

***N*-(Adamantan-1-yl)-*N*-benzylformamide (1x)**
White solid (72% yield for two steps). m.p. 94-96 °C. Rf 0.50 (SiO_2, hexane/EtOAc = 6/1). **^{1}H NMR** (400 MHz, $CDCl_3$) δ 8.70 (s, 1H), 7.35 – 7.23 (m, 2H), 7.24 – 7.12 (m, 3H), 4.64 (s, 2H), 2.16 – 2.07 (m, 3H), 1.96 – 1.86 (m, 6H), 1.75 – 1.52 (m, 6H). **^{13}C NMR** (100 MHz, $CDCl_3$) δ 162.2, 139.2, 128.5, 126.8, 126.7, 56.9, 43.0, 42.6, 36.0, 29.6. **HRMS (ESI)** *m/z:* $[M+H]^+$ Calcd. for $C_{18}H_{25}NO$ 270.1852; Found 270.1851.

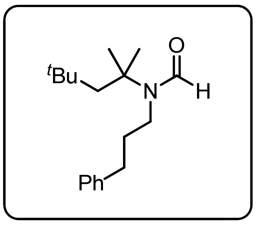

***N*-(3-Phenylpropyl)-*N*-(2,4,4-trimethylpentan-2-yl)formamide (1y)**
Light yellow oil (34% yield for two steps). Rf 0.51 (SiO_2, hexane/EtOAc = 6/1). **^{1}H NMR** (400 MHz, $CDCl_3$) δ 8.41 (s, 1H), 7.31 – 7.26 (m, 2H), 7.22 – 7.16 (m, 3H), 3.28 – 3.22 (m, 2H), 2.65 (t, J = 7.8 Hz, 2H), 1.96 – 1.87 (m, 2H), 1.52 (s, 2H), 1.39 (s, 6H), 0.93 (s, 9H). **^{13}C NMR** (100 MHz, $CDCl_3$) δ 162.5, 141.7, 128.5, 128.5, 126.0, 59.1, 52.6, 42.0, 33.8, 31.8, 31.5, 30.9, 30.0. **HRMS (ESI)** *m/z:* $[M+Na]^+$ Calcd. for $C_{18}H_{29}NONa$ 298.2141; Found 298.2145.

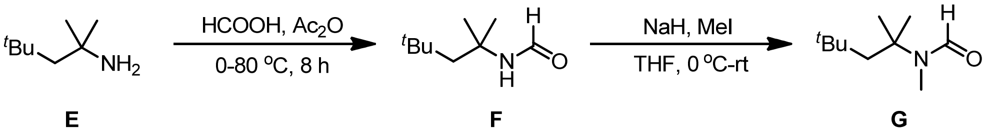

A mixture of formic acid (1.9 mL, 50 mmol) and acetic anhydride (4.7 mL, 50 mmol) was stirred for 30 mins at room temperature. To the solution was added 1,1,3,3-tetramethylbutylamine **E** at 0 °C, and the resulting mixture was heated to 80 °C and stirred for an additional 8 hours. After cooling to room temperature, the mixture was concentrated under reduced pressure, and the residue was purified by column chromatography on silica gel.

To a solution of amide **F** in dry THF was added NaH (3.0 equiv, 60% dispersion in mineral oil) and MeI (1.5 equiv) at 0 °C under N_2, and the resulting mixture was stirred at room temperature until the complete consumption of amide **F** by TLC analysis. The reaction mixture was quenched by saturated aqueous NH_4Cl and extracted with EtOAc. The combined organic phases were washed with brine, dried over anhydrous $MgSO_4$, filtered and evaporated to afford a crude product, which was further purified by column chromatography on silica gel.

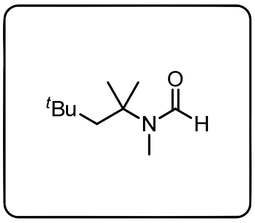

N-Methyl-N-(2,4,4-trimethylpentan-2-yl)formamide (1z)

Light yellow oil (30% yield over two steps). Rf 0.48 (SiO_2, hexane/EtOAc = 6/1). **^{1}H NMR** (400 MHz, $CDCl_3$) δ 8.33 (s, 1H), 2.77 (s, 3H), 1.52 (s, 2H), 1.37 (s, 6H), 0.91 (s, 9H). **^{13}C NMR** (100 MHz, $CDCl_3$) δ 161.8, 58.4, 51.1, 31.6, 31.2, 29.5, 26.8. **HRMS (ESI)** *m/z:* $[M+Na]^+$ Calcd. for $C_{10}H_{21}NONa$ 194.1515; Found 194.1520.

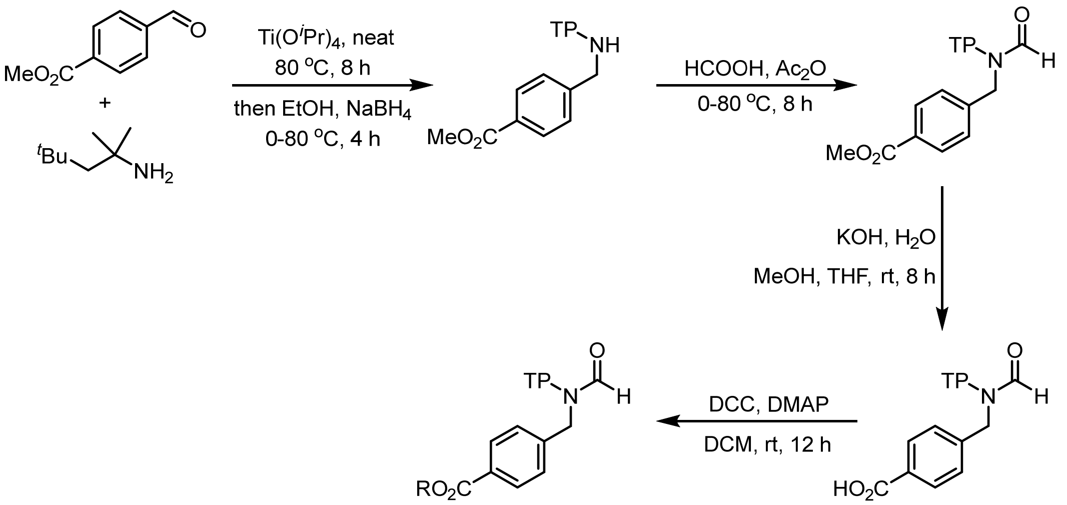

A solution of methyl 4-formylbenzoate (25.0 mmol), 2,3,3-trimethylbutan-2-amine (27.5 mmol) and $Ti(O^iPr)_4$ (15.0 mL) was stirred for 8 hours at 80 °C under nitrogen atmosphere. After cooled to 0 °C, the mixture was diluted with anhydrous ethanol (200 mL), to which sodium borohydride (2.8 g, 75 mmol) was added portionwise over 10 min. The resulting mixture was stirred for an additional 4 hours at 80 °C, cooled to room temperature, and quenched with 2.0 M sodium hydroxide. The white suspension was filtered and washed with ethyl acetate. The filtrate was extracted with ethyl acetate and the combined organic extracts were washed with brine, dried over anhydrous magnesium sulphate and concentrated in vacuo. The crude product was directly used for next step without further purification.

To a mixture of formic acid (9.5 mL, 250 mmol) and acetic anhydride (23.5 mL, 250 mmol) was added crude amine at 0 °C, and the resulting mixture was heated to 80 °C and stirred for an additional 8 hours. After cooled to room temperature, the solution was concentrated under reduced pressure, and the residue was purified by column chromatography on silica gel.

The ester (1.8 g, 6.0 mmol) was dissolved in THF (70 mL) and MeOH (50 mL). The resulting solution was then mixed with a solution of KOH (4 g, 72.0 mmol) in H_2O (80 mL). The reaction mixture was stirred at room temperature overnight. After all the volatiles were removed in vacuo, it was diluted with H_2O and acidified with 1M HCl solution until pH = 2. The precipitates were collected via centrifugation, washed with H_2O, and dried in air. The crude product was directly used for next step without further purification.

To a flame-dried 100 mL flask were added crude acid (291 mg, 1.0 mmol), alcohol (1.0 mmol) and

anhydrous DCM. Then DCC (206 mg, 1.0 mmol) and DMAP (122 mg, 1.0 mmol) were added at 30 °C and the resulting mixture was stirred vigorously for 12 hours. The solution was concentrated under reduced pressure, and the crude product was purified by silica gel flash column chromatography (*n*-hexane/EtOAc) to give the desired ester.

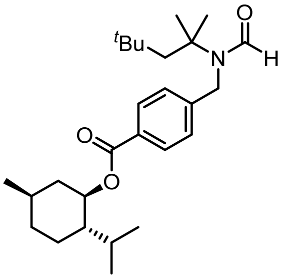

(1*R*,2*S*,5*R*)-2-Isopropyl-5-methylcyclohexyl 4-((*N*-(2,4,4-trimethylpentan-2-yl)formamido)methyl)benzoate (1k′)

White solid (78% yield). m.p. 100-102 °C. Rf 0.43 (SiO_2, hexane/EtOAc = 6/1).^{1}**H NMR** (400 MHz, $CDCl_3$) δ 8.69 (s, 1H), 8.00 – 7.93 (m, 2H), 7.28 (d, J = 8.6 Hz, 3H), 4.91 (td, J = 10.9, 4.4 Hz, 1H), 4.66 – 4.61 (m, 2H), 2.11 (m, J = 11.9, 4.2, 3.2 Hz, 1H), 1.96 (m, J = 13.9, 7.1, 2.3 Hz, 1H), 1.77 – 1.69 (m, 2H), 1.66 (s, 2H), 1.54 (ddt, J = 13.9, 10.3, 3.9 Hz, 2H), 1.39 (d, J = 4.2 Hz, 6H), 1.31 (d, J = 19.8 Hz, 1H), 1.19 – 1.05 (m, 2H), 1.00 (s, 9H), 0.92 (dd, J = 6.8, 5.2 Hz, 6H), 0.78 (d, J = 6.9 Hz, 3H). ^{13}**C NMR** (100 MHz, $CDCl_3$) δ 166.0, 163.0, 144.1, 129.9, 129.5, 126.8, 77.4, 77.1, 76.8, 74.8, 59.6, 53.3, 47.3, 44.8, 41.1, 34.4, 31.9, 31.6, 31.6, 30.4, 30.4, 26.6, 23.8, 22.2, 20.9, 16.6. **HRMS (ESI)** *m/z:* $[M+Na]^+$ Calcd. for $C_{27}H_{43}NO_3$ 452.3135; Found 452.3138.

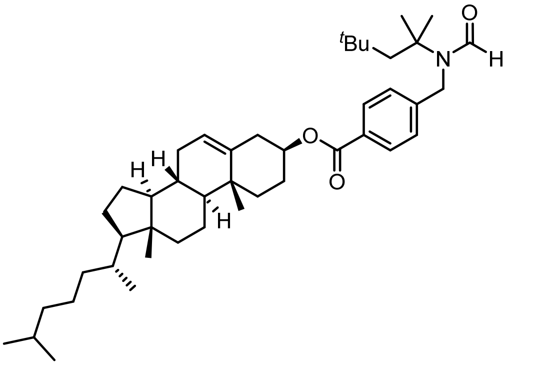

(3*S*,8*S*,9*S*,10*R*,13*R*,14*S*,17*R*)-10,13-Dimethyl-17-((*R*)-6-methylheptan-2-yl)-2,3,4,7,8,9,10,11,12,13,14,15,16,17-tetradecahydro-1*H*-cyclopenta[*a*]phenanthren-3-yl 4-((*N*-(2,4,4-trimethylpentan-2-yl)formamido)methyl)benzoate (1l′)

Brown solid (73% yield). m.p. 194-195 °C. Rf 0.42 (SiO_2, hexane/EtOAc = 6/1).^{1}**H NMR** (400 MHz, $CDCl_3$) δ 8.68 (s, 1H), 7.96 (d, J = 8.4 Hz, 2H), 7.27 (d, J = 9.2 Hz, 3H), 5.41 (dd, J = 5.0, 1.9 Hz, 1H), 4.83 (dtd, J = 12.1, 8.4, 4.4 Hz, 1H), 4.63 (s, 2H), 2.51 – 2.41 (m, 2H), 2.08 – 1.94 (m, 3H), 1.90 (dt, J = 13.3, 3.4 Hz, 1H), 1.87 – 1.75 (m, 1H), 1.75 – 1.67 (m, 1H), 1.65 (s, 2H), 1.63 – 1.40 (m, 7H), 1.37 (s, 6H), 1.35 – 1.08 (m, 11H), 1.06 (s, 4H), 1.04 – 1.01 (m, 2H), 0.99 (s, 9H), 0.92 (d, J = 6.5 Hz, 3H), 0.86 (dd, J = 6.6, 1.8 Hz, 6H), 0.68 (s, 3H). ^{13}**C NMR** (100 MHz, $CDCl_3$) δ 165.9, 163.1, 144.2, 139.8, 129.9, 129.6, 126.9, 122.9, 77.5, 77.4, 77.2, 76.8, 74.6, 59.7, 56.8, 56.3, 53.4, 50.2, 44.8, 42.5, 39.9, 39.6, 38.4, 37.2, 36.8, 36.3, 35.9, 32.1, 32.0, 31.9, 31.7, 31.6, 30.4, 28.4, 28.2, 28.0, 24.4, 24.0, 23.0, 22.7, 21.2, 19.5, 18.9, 12.0. **HRMS (ESI)** *m/z:* $[M+H]^+$ Calcd. for $C_{44}H_{69}NO_3$

660.5350; Found 660.5355.

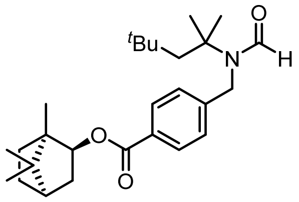

(1*R*,2*S*,4*R*)-1,7,7-Trimethylbicyclo[2.2.1]heptan-2-yl 4-((*N*-(2,4,4-trimethylpentan-2-yl)formamido)methyl)benzoate (1m′)

White solid (68% yield). m.p. 123-125 °C. Rf 0.44 (SiO_2, hexane/EtOAc = 6/1).^{1}**H NMR** (400 MHz, $CDCl_3$) δ 8.71 (s, 1H), 8.01 (d, J = 8.3 Hz, 2H), 7.31 (d, J = 8.1 Hz, 2H), 5.16 – 5.08 (m, 1H), 4.66 (s, 2H), 2.48 (ddt, J = 13.8, 9.9, 4.0 Hz, 1H), 2.14 (ddd, J = 13.2, 9.4, 4.4 Hz, 1H), 1.81 (dq, J = 8.0, 4.2 Hz, 1H), 1.75 (t, J = 4.5 Hz, 1H), 1.68 (s, 2H), 1.41 (s, 6H), 1.36 – 1.26 (m, 2H), 1.12 (dd, J = 13.8, 3.5 Hz, 1H), 1.02 (s, 9H), 0.98 (s, 3H), 0.93 (d, J = 4.5 Hz, 6H). ^{13}**C NMR** (100 MHz, $CDCl_3$) δ 166.7, 163.0, 144.2, 129.8, 129.6, 126.9, 80.5, 77.5, 77.2, 76.8, 59.7, 53.3, 49.2, 48.0, 45.1, 44.8, 37.0, 31.8, 31.6, 30.4, 28.2, 27.5, 19.8, 19.0, 13.7. **HRMS (ESI)** *m/z:* $[M+Na]^+$ Calcd. for $C_{27}H_{41}NO_3$ 450.2979; Found 450.2983.

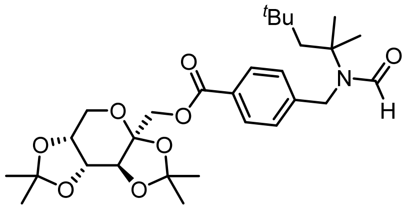

((3*aS*,5*aR*,8*aR*,8*bS*)-2,2,7,7-tetramethyltetrahydro-3a*H*-bis([1,3]dioxolo)[4,5-b:4',5'-d]pyran-3a-yl)methyl 4-((*N*-(2,4,4-trimethylpentan-2-yl)formamido)methyl)benzoate (1n′)

White solid (43% yield). m.p. 99-100 °C. Rf 0.38 (SiO_2, hexane/EtOAc = 6/1).^{1}**H NMR** (400 MHz, $CDCl_3$) δ 8.68 (s, 1H), 7.99 (d, J = 8.3 Hz, 2H), 7.28 (d, J = 8.0 Hz, 2H), 4.68 – 4.60 (m, 4H), 4.45 (d, J = 2.6 Hz, 1H), 4.31 (d, J = 11.8 Hz, 1H), 4.25 (dd, J = 7.9, 1.7 Hz, 1H), 3.95 (dd, J = 13.0, 1.9 Hz, 1H), 3.79 (d, J = 13.0 Hz, 1H), 1.65 (s, 2H), 1.54 (s, 3H), 1.45 (s, 3H), 1.36 (s, 9H), 1.34 (s, 3H), 0.98 (s, 9H). ^{13}**C NMR** (100 MHz, $CDCl_3$) δ 165.8, 163.1, 144.6, 130.1, 128.6, 127.0, 109.2, 108.9, 101.7, 77.5, 77.2, 76.9, 70.9, 70.6, 70.2, 65.3, 61.4, 59.7, 53.2, 44.7, 31.8, 31.5, 30.3, 26.6, 26.0, 25.6, 24.1. **HRMS (ESI)** *m/z:* $[M+Na]^+$ Calcd. for $C_{30}H_{45}NO_7$ 556.2881; Found 556.2993.

Supplementary Note 2

Reaction Optimization

Supplementary Table 1. Comparison of protecting groups

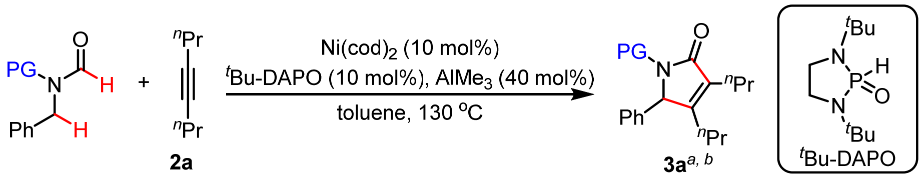

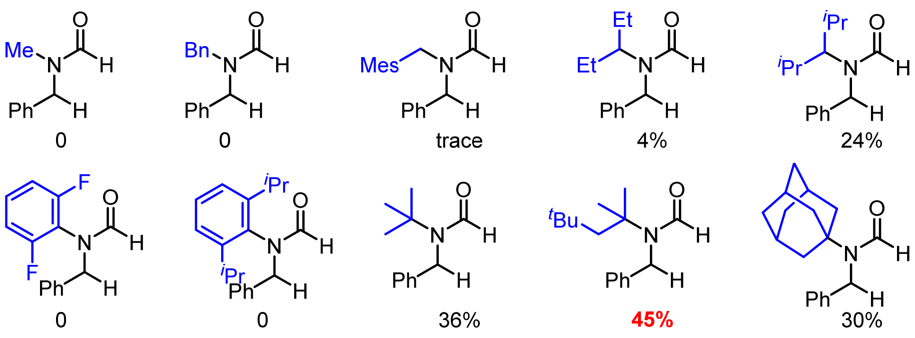

[a] Reaction conditions: formamide (0.2 mmol), **2a** (0.6 mmol), toluene (1.0 mL), N_2, 12 h. [b] Yields were determined by ^{1}H NMR analysis with CH_2Br_2 as the internal standard.

Supplementary Table 2. Lewis acid effects

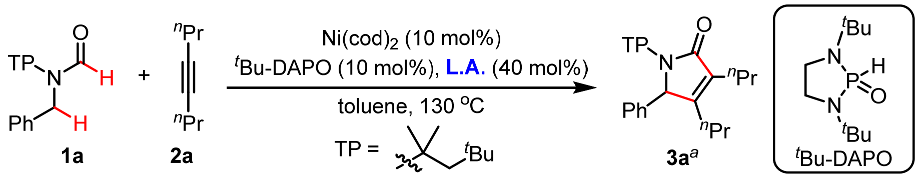

entry	L.A. (40 mol%)	yield (%)[b]
1	$AlMe_3$	45
2	**$AlEt_3$**	**81**
3	Al^iBu_3	78
4	$AlMe_2Cl$	0
5	$MgBu_2$	16
6	$ZnMe_2$	0
7	BPh_3	0

[a]Reaction conditions: **1a** (0.2 mmol), **2a** (0.6 mmol), toluene (1.0 mL), N_2, 12 h. [b]Determined by ^{1}H NMR analysis with DMF as the internal standard.

Supplementary Table 3. Reaction time and Lewis acid loading effects

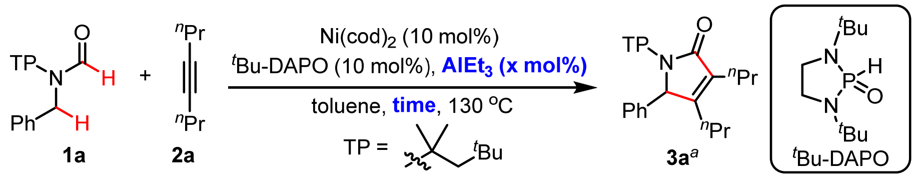

entry	time (h)	$AlEt_3$ (x mol%)	yield (%)[b]
1	12	40	81
2	5	40	81
3	**2**	**40**	**81**
4	2	20	24
5	2	60	80

[a]Reaction conditions: **1a** (0.2 mmol), **2a** (0.6 mmol), toluene (1.0 mL), N_2. [b]Determined by ^{1}H NMR analysis with DMF as the internal standard.

Supplementary Table 4. Temperature effects

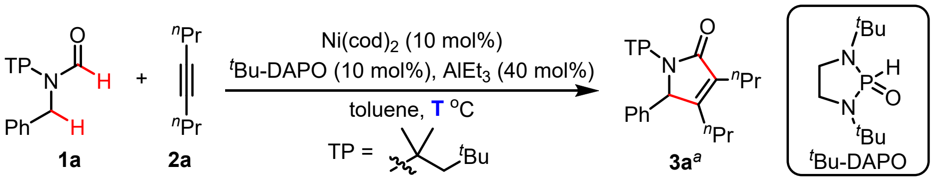

entry	T (°C)	yield (%)[b]
1	130	81
2	**120**	**81**
3	110	52
4	100	22
5	90	12
6	80	4

[a]Reaction conditions: **1a** (0.2 mmol), **2a** (0.6 mmol), toluene (1.0 mL), N_2, 2 h. [b]Determined by ^1H NMR analysis with DMF as the internal standard.

Supplementary Table 5. Solvent effects

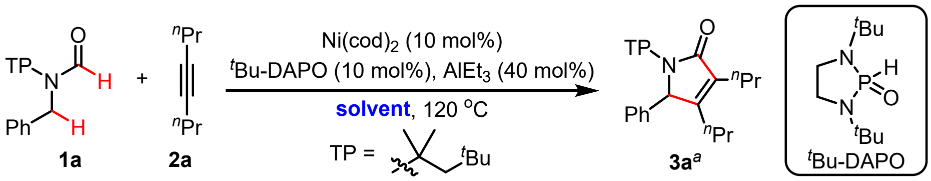

entry	solvent (1.0 mL)	yield (%)[b]
1	**toluene**	**81**
2	xylene	76
3	mesitylene	58
4	dioxane	64
5	hexane	64

[a]Reaction conditions: **1a** (0.2 mmol), **2a** (0.6 mmol), toluene (1.0 mL), N_2, 2 h. [b]Determined by ^1H NMR analysis with DMF as the internal standard.

Supplementary Table 6. Alkyne loading effects

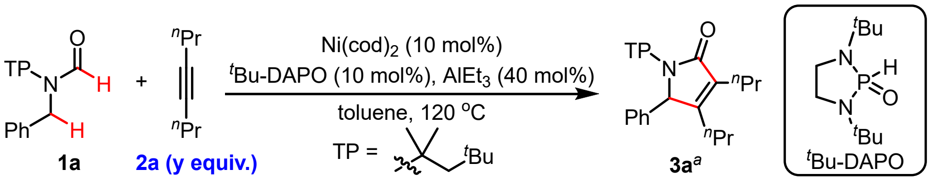

entry	2a (y equiv.)	yield (%)[b]
1	3.4	80
2	**3.0**	**81**
3	2.6	58
4	2.2	28

[a]Reaction conditions: **1a** (0.2 mmol), **2a** (0.6 mmol), toluene (1.0 mL), N_2, 2 h. [b]Determined by ^1H NMR analysis with DMF as the internal standard.

Supplementary Table 7. Ligand effects

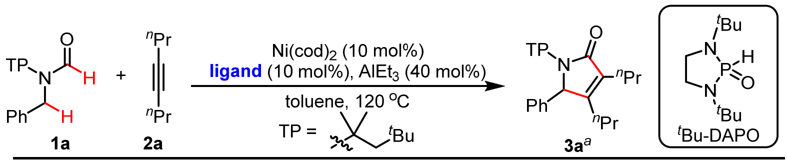

entry	**ligand**	yield (%)[b]
1	PPh_3	0
2	PCy_3	0
3	BINAP	0
4	IPr	0
5	IMes	0
6	$Ph_2P(O)H$	0
7	***t*Bu-DAPO**	**81**

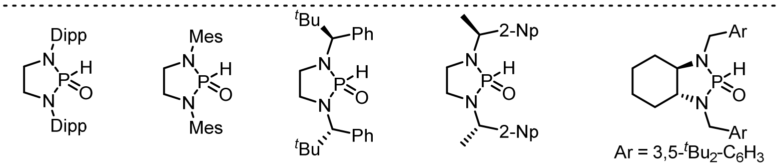

trace | **L1**, trace | **L2**, 12% | 0 | Ar = 3,5-^{t}Bu$_2$-C$_6$H$_3$ **L3**, 0

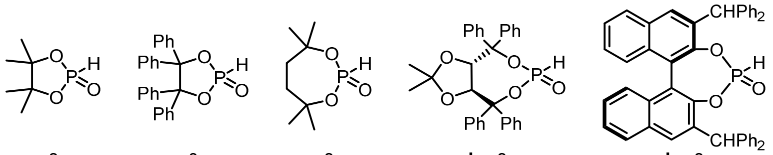

0 | 0 | 0 | **L4**, 0 | **L5**, 0

[a]Reaction conditions: **1a** (0.2 mmol), **2a** (0.6 mmol), toluene (1.0 mL), N_2, 2 h. [b]Determined by ^{1}H NMR analysis with DMF as the internal standard.

Supplementary Table 8. Control experiment

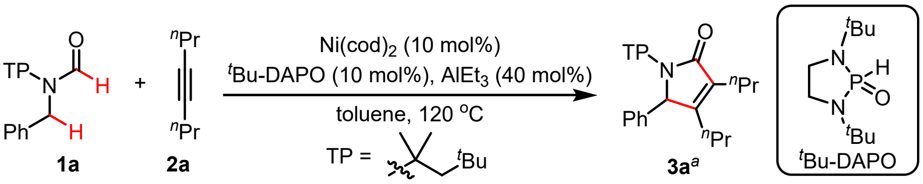

entry	*deviation from standard cnditions*	yield (%)[b]
1	**none**	**81**
2	w/o $Ni(cod)_2$	0
3	w/o $AlEt_3$	0
4	w/o ^{t}Bu-DAPO	0
5	$NiBr_2$·diglyme instead of $Ni(cod)_2$	0
6	$NiBr_2$·diglyme with Zn (50 mol%) instead of $Ni(cod)_2$	14

[a]Reaction conditions: **1a** (0.2 mmol), **2a** (0.6 mmol), toluene (1.0 mL), N_2, 2 h. [b]Determined by ^{1}H NMR analysis with DMF as the internal standard.

Supplementary Note 3

General Procedure for [3+2] Annulation

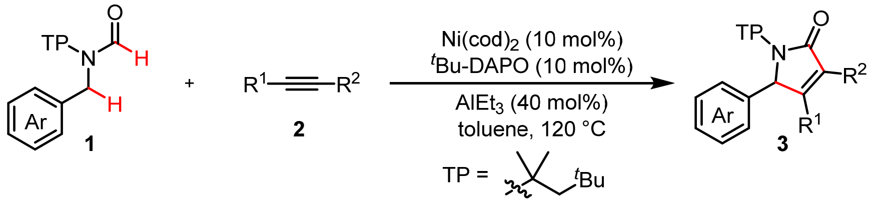

To a 15 mL oven-dried tube were added ^{t}Bu-DAPO (4.4 mg, 10 mol%), Ni(cod)$_2$ (5.5 mg, 10 mol%), dry degassed toluene (1.0 mL), benzyl formamide (0.2 mmol), alkyne (0.6 mmol) and AlEt$_3$ (1.0 M in toluene, 80 μL, 40 mol%) sequentially in an N_2-filled glove-box. The tube was sealed and removed out of the glove-box. After heated at 120 °C in a preheated dry block heater for 2 h, the mixture was cooled to r.t., quenched with 0.1 mL H_2O, filtered through a short plug of silica gel (DCM as the eluent) and concentrated in vacuo to afford a crude product. Further purification by flash column chromatography on silica gel (eluting with EtOAc/*n*-hexane) gave the pure product.

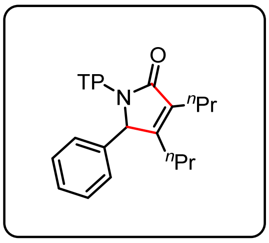

5-Phenyl-3,4-dipropyl-1-(2,4,4-trimethylpentan-2-yl)-1*H*-pyrrol-2(5*H*)-one (3a)
Colourless oil (56.9 mg, 80% yield). Rf 0.29 (SiO_2, hexane/EtOAc = 40/1). **^{1}H NMR** (400 MHz, $CDCl_3$) δ 7.42 – 7.19 (m, 4H), 7.01 (s, 1H), 4.92 (s, 1H), 2.85 (d, J = 14.8 Hz, 1H), 2.23 – 2.18 (m, 2H), 2.18 – 2.09 (m, 1H), 1.68 – 1.43 (m, 4H), 1.42 (s, 3H), 1.34 – 1.22 (m, 1H), 1.19 (d, J = 14.8 Hz, 1H), 1.14 (s, 3H), 0.95 – 0.88 (m, 12H), 0.85 (t, J = 7.3 Hz, 3H). **^{13}C NMR** (100 MHz, $CDCl_3$) δ 173.8, 154.1, 139.8, 133.1, 128.7, 127.8, 125.4, 67.2, 59.0, 50.2, 31.6, 31.4, 30.8, 28.3, 28.1, 25.8, 22.2, 14.3, 14.1. **HRMS (ESI)** *m/z:* $[M+H]^+$ Calcd. for $C_{18}H_{15}F_4N_2$ 356.2948; Found 356.2952. **IR** (neat, cm^{-1}): 1664, 1660, 1465, 1454, 1379, 1364, 1238, 1223, 1169, 1110, 763, 701.

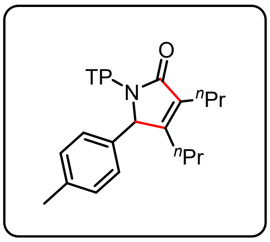

3,4-Dipropyl-5-(*p*-tolyl)-1-(2,4,4-trimethylpentan-2-yl)-1*H*-pyrrol-2(5*H*)-one (3b)
Colourless oil (70.2 mg, 95% yield). Rf 0.29 (SiO_2, hexane/EtOAc = 40/1). **^{1}H NMR** (400 MHz, $CDCl_3$) δ 7.20 – 7.05 (m, 3H), 6.95 – 6.75 (m, 1H), 4.88 (s, 1H), 2.85 (d, J = 14.8 Hz, 1H), 2.32 (s, 3H), 2.22 – 2.09 (m, 3H), 1.64 – 1.43 (m, 4H), 1.40 (s, 3H), 1.32 – 1.22 (m, 1H), 1.18 – 1.12 (m, 4H), 0.92 – 0.88 (m, 12H), 0.83 (t, J = 7.3 Hz, 3H). **^{13}C NMR** (100 MHz, $CDCl_3$) δ 173.7, 154.2, 137.4,

136.6, 132.9, 129.3, 125.2, 66.9, 58.9, 50.2, 31.5, 31.4, 30.8, 28.2, 28.1, 25.8, 22.2, 21.2, 14.2, 14.1. **HRMS (ESI)** *m/z:* $[M+H]^+$ Calcd. for $C_{25}H_{40}NO$ 370.3104; Found 370.3103. **IR** (neat, cm^{-1}): 2871, 1682, 1678, 1674, 1671, 1667, 1652, 1511, 1464, 1456, 1388, 1378, 1364, 1352, 1328, 1259, 1237, 1223, 1169, 1107, 815, 765.

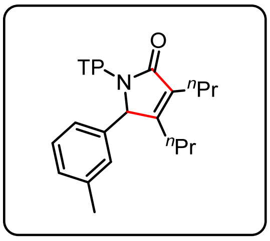

3,4-Dipropyl-5-(*m*-tolyl)-1-(2,4,4-trimethylpentan-2-yl)-1*H*-pyrrol-2(5*H*)-one (3c)
Colourless oil (71.6 mg, 97% yield). Rf 0.28 (SiO_2, hexane/EtOAc = 40/1). **^{1}H NMR** (400 MHz, $CDCl_3$) δ 7.24 – 7.12 (m, 1H), 7.05 – 7.03 (m, 2H), 6.84 – 6.72 (m, 1H), 4.87 (s, 1H), 2.84 (d, J = 14.8 Hz, 1H), 2.39 – 2.08 (m, 6H), 1.66 – 1.43 (m, 4H), 1.40 (s, 3H), 1.31 – 1.22 (m, 1H), 1.17 (d, J = 14.8 Hz, 1H), 1.13 (s, 3H), 0.94 – 0.87 (m, 12H), 0.84 (t, J = 7.4 Hz, 3H). **^{13}C NMR** (100 MHz, $CDCl_3$) δ 173.8, 154.2, 139.7, 138.5, 133.0, 129.4, 128.5, 125.8, 122.5, 67.2, 59.0, 50.3, 31.6, 31.4, 30.7, 28.2, 28.1, 25.9, 22.2, 22.1, 21.5, 14.2, 14.1. **HRMS (ESI)** *m/z:* $[M+H]^+$ Calcd. for $C_{25}H_{40}NO$ 370.3104; Found 370.3109. **IR** (neat, cm^{-1}): 2935, 2871, 1673, 1669, 1665, 1607, 1484, 1464, 1379, 1363, 1326, 1260, 1250, 1235, 1223, 1182, 1167, 1129, 1111, 1092, 782, 763, 750, 708.

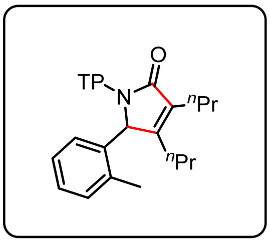

3,4-Dipropyl-5-(*o*-tolyl)-1-(2,4,4-trimethylpentan-2-yl)-1*H*-pyrrol-2(5*H*)-one (3d)
Light yellow oil (59.8 mg, 81% yield). Rf 0.29 (SiO_2, hexane/EtOAc = 40/1). **^{1}H NMR** (400 MHz, $CDCl_3$) δ 7.19 – 7.05 (m, 3H), 6.96 – 6.90 (m, 1H), 5.24 (s, 1H), 2.86 (d, J = 14.8 Hz, 1H), 2.47 (s, 3H), 2.20 (t, J = 7.7 Hz, 2H), 2.16 – 2.08 (m, 1H), 1.68 – 1.47 (m, 4H), 1.40 (s, 3H), 1.31 – 1.21 (m, 1H), 1.16 (d, J = 14.9 Hz, 1H), 1.10 (s, 3H), 0.95 – 0.90 (m, 12H), 0.85 (t, J = 7.3 Hz, 3H). **^{13}C NMR** (100 MHz, $CDCl_3$) δ 174.0, 154.6, 137.5, 134.7, 133.3, 130.9, 127.4, 126.4, 125.9, 62.4, 58.6, 50.2, 31.6, 31.5, 30.4, 28.2, 27.0, 26.0, 22.9, 22.1, 19.6, 14.3, 14.2. **HRMS** (ESI) calcd. for $C_{25}H_{40}NO$ ($[M+H]^+$) 370.3104, Found. 370.3108. **IR** (neat, cm^{-1}): 2989, 2955, 2932, 2905, 2870, 1668, 1629, 1486, 1463, 1454, 1394, 1387, 1362, 1251, 1238, 1222, 1168, 764.

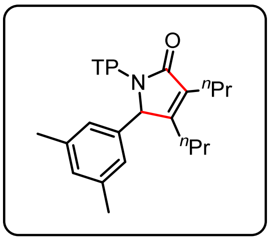

5-(3,5-Dimethylphenyl)-3,4-dipropyl-1-(2,4,4-trimethylpentan-2-yl)-1*H*-pyrrol-2(5*H*)-one (3e)
White solid (65.2 mg, 85% yield). m.p. 95-97 °C. Rf 0.29 (SiO_2, hexane/EtOAc = 40/1). **^{1}H NMR** (400 MHz, $CDCl_3$) δ 6.87 – 6.85 (m, 2H), 6.62 – 6.56 (m, 1H), 4.84 (s, 1H), 2.85 (d, J = 14.8 Hz, 1H), 2.42 – 2.25 (m, 3H), 2.27 – 2.06 (m, 6H), 1.67 – 1.44 (m, 4H), 1.41 (s, 3H), 1.37 – 1.22 (m, 1H),

1.19 – 1.15 (m, 4H), 0.95 – 0.88 (m, 12H), 0.85 (t, *J* = 7.4 Hz, 3H). **^{13}C NMR** (100 MHz, $CDCl_3$) *δ* 173.9, 154.3, 139.6, 132.9, 129.3, 126.5, 123.7, 122.9, 67.2, 59.0, 50.2, 31.6, 31.4, 30.8, 28.2, 28.1, 25.9, 22.2, 22.2, 21.4, 14.2, 14.1, 1.1. **HRMS (ESI)** *m/z:* $[M+H]^+$ Calcd. for $C_{26}H_{42}NO$ 384.3261; Found 384.3265. **IR** (neat, cm^{-1}): 3002, 2959, 2937, 2871, 1655, 1466, 1396, 1384, 1377, 1363, 1255, 1238, 1222, 1181, 1176, 1169, 1153, 1111, 1107, 802, 474, 471.

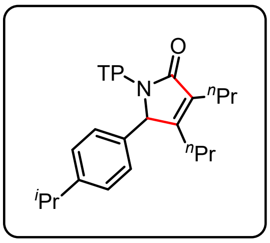

5-(4-Isopropylphenyl)-3,4-dipropyl-1-(2,4,4-trimethylpentan-2-yl)-1*H*-pyrrol-2(5*H*)-one (3f)
Colourless oil (72.3 mg, 91% yield). Rf 0.30 (SiO_2, hexane/EtOAc = 40/1). **^{1}H NMR** (400 MHz, $CDCl_3$) *δ* 7.18 – 6.89 (m, 4H), 4.89 (s, 1H), 2.94 – 2.80 (m, 2H), 2.24 – 2.08 (m, 3H), 1.67 – 1.59 (m, 1H), 1.56 – 1.45 (m, 3H), 1.41 (s, 3H), 1.31 – 1.27 (m, 1H), 1.23 (d, *J* = 6.9 Hz, 6H), 1.20 – 1.13 (m, 4H), 0.95 – 0.89 (m, 12H), 0.84 (t, *J* = 7.3 Hz, 3H). **^{13}C NMR** (100 MHz, $CDCl_3$) *δ* 173.8, 154.2, 148.4, 136.8, 132.8, 67.0, 58.9, 50.2, 33.8, 31.6, 31.4, 30.8, 28.2, 28.1, 25.8, 24.1, 24.0, 22.2, 14.3, 14.0. **HRMS (ESI)** *m/z:* $[M+H]^+$ Calcd. for $C_{27}H_{44}NO$ 398.3417; Found 398.3417. **IR** (neat, cm^{-1}): 2935, 2871, 1678, 1675, 1464, 1420, 1389, 1378, 1364, 1260, 1237, 1222, 1168.

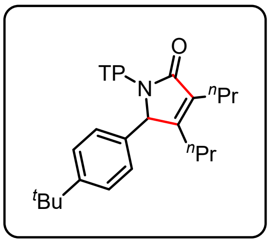

5-(4-(*tert*-Butyl)phenyl)-3,4-dipropyl-1-(2,4,4-trimethylpentan-2-yl)-1*H*-pyrrol-2(5*H*)-one (3g)
Colourless oil (74.0 mg, 90% yield). Rf 0.27 (SiO_2, hexane/EtOAc = 40/1). **^{1}H NMR** (400 MHz, $CDCl_3$) *δ* 7.32 – 7.26 (m, 2H), 7.16 – 6.82 (m, 2H), 4.89 (s, 1H), 2.84 (d, *J* = 14.8 Hz, 1H), 2.24 – 2.08 (m, 3H), 1.67 – 1.46 (m, 4H), 1.42 (s, 3H), 1.32 – 1.26 (m, 10H), 1.18 (d, *J* = 14.8 Hz, 1H), 1.14 (s, 3H), 0.96 – 0.88 (m, 12H), 0.85 (t, *J* = 7.3 Hz, 3H). **^{13}C NMR** (100 MHz, $CDCl_3$) *δ* 173.8, 154.2, 150.7, 136.4, 132.9, 128.2, 125.7, 66.9, 58.9, 50.2, 34.6, 31.6, 31.5, 31.4, 30.8, 28.2, 28.1, 25.8, 22.2, 14.3, 14.0. **HRMS (ESI)** *m/z:* $[M+H]^+$ Calcd. for $C_{28}H_{46}NO$ 412.3574; Found 412.3578. **IR** (neat, cm^{-1}): 2959, 2906, 2871, 1682, 1678, 1464, 1388, 1378, 1364, 1353, 1267, 1237, 1223, 1167, 1110.

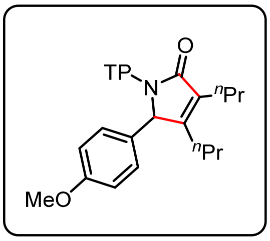

5-(4-Methoxyphenyl)-3,4-dipropyl-1-(2,4,4-trimethylpentan-2-yl)-1*H*-pyrrol-2(5*H*)-one (3h)
Colourless oil (53.9 mg, 70% yield). Rf 0.23 (SiO_2, hexane/EtOAc = 40/1). **^{1}H NMR** (400 MHz, $CDCl_3$) *δ* 7.25 – 7.05 (m, 1H), 7.03 – 6.77 (m, 3H), 4.86 (s, 1H), 3.79 (s, 3H), 2.84 (d, *J* = 14.8 Hz, 1H), 2.24 – 2.07 (m, 3H), 1.68 – 1.58 (m, 1H), 1.58 – 1.42 (m, 3H), 1.39 (s, 3H), 1.30 – 1.22 (m, 1H),

1.20 – 1.11 (m, 4H), 0.93 – 0.87 (m, 12H), 0.83 (t, J = 7.3 Hz, 3H). **^{13}C NMR** (100 MHz, $CDCl_3$) δ 173.7, 159.2, 154.2, 132.9, 131.5, 66.6, 58.9, 55.4, 50.3, 31.6, 31.4, 30.8, 28.2, 28.1, 25.8, 22.2, 22.1, 14.2, 14.1. **HRMS (ESI)** *m/z:* $[M+H]^+$ Calcd. for $C_{25}H_{40}NO_2$ 386.3054; Found 386.3057. **IR** (neat, cm^{-1}): 2935, 2908, 2871, 1678, 1674, 1671, 1610, 1510, 1464, 1378, 1364, 1303, 1252, 1225, 1171, 1106, 1036.

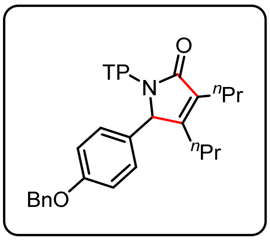

5-(4-(Benzyloxy)phenyl)-3,4-dipropyl-1-(2,4,4-trimethylpentan-2-yl)-1*H*-pyrrol-2(5*H*)-one (3i)
Colourless oil (85.8 mg, 93% yield). Rf 0.21 (SiO_2, hexane/EtOAc = 40/1). **^{1}H NMR** (400 MHz, $CDCl_3$) δ 7.44 – 7.31 (m, 5H), 7.23 – 7.05 (m, 1H), 6.97 – 6.87 (m, 3H), 5.04 (s, 2H), 4.88 (s, 1H), 2.85 (d, J = 14.8 Hz, 1H), 2.24 – 2.10 (m, 3H), 1.68 – 1.61 (m, 1H), 1.57 – 1.44 (m, 3H), 1.40 (s, 3H), 1.31 – 1.22 (m, 1H), 1.20 – 1.14 (m, 4H), 0.95 – 0.88 (m, 12H), 0.85 (t, J = 7.3 Hz, 3H). **^{13}C NMR** (100 MHz, $CDCl_3$) δ 173.8, 158.4, 154.5, 136.9, 132.8, 131.6, 128.7, 128.1, 127.7, 115.7, 114.5, 70.2, 66.7, 59.0, 50.2, 31.6, 31.4, 30.8, 28.2, 28.1, 25.8, 22.2, 22.1, 14.2, 14.1. **HRMS (ESI)** *m/z:* $[M+H]^+$ Calcd. for $C_{31}H_{44}NO_2$ 462.3367; Found 462.3365. **IR** (neat, cm^{-1}): 3032, 2959, 2935, 2871, 1674, 1671, 1609, 1585, 1509, 1464, 1455, 1388, 1379, 1364, 1328, 1312, 1300, 1240, 1224, 1171, 1107, 1025, 842, 825, 736, 696.

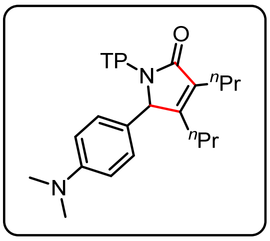

5-(4-(Dimethylamino)phenyl)-3,4-dipropyl-1-(2,4,4-trimethylpentan-2-yl)-1*H*-pyrrol-2(5*H*)-one (3j)
Light yellow oil (66.1 mg, 83% yield). Rf 0.21 (SiO_2, hexane/EtOAc = 40/1). **^{1}H NMR** (400 MHz, $CDCl_3$) δ 7.10 – 6.80 (m, 2H), 6.67 – 6.61 (m, 2H), 4.83 (s, 1H), 2.94 (s, 6H), 2.87 (d, J = 14.7 Hz, 1H), 2.22 – 2.08 (m, 3H), 1.69 – 1.62 (m, 1H), 1.58 – 1.44 (m, 3H), 1.40 (s, 3H), 1.33 – 1.24 (m, 1H), 1.19 – 1.14 (m, 4H), 0.93 – 0.89 (m, 12H), 0.84 (t, J = 7.3 Hz, 3H). **^{13}C NMR** (100 MHz, $CDCl_3$) δ 173.7, 154.6, 150.0, 132.4, 126.6, 66.8, 58.8, 50.2, 40.6, 31.6, 31.4, 30.8, 28.1, 25.8, 22.2, 22.1, 14.2, 14.1. **HRMS (ESI)** *m/z:* $[M+H]^+$ Calcd. for $C_{26}H_{43}N_2O$ 399.3370; Found 399.3373. **IR** (neat, cm^{-1}): 3465, 3458, 3453, 3444, 3436, 3429, 3424, 3349, 3196, 3062, 2960, 2934, 2871, 1682, 1678, 1674, 1667, 1652, 1608, 1582, 1514, 1463, 1455, 1412, 1378, 1364, 1343, 1307, 1273, 1259, 1236, 1223, 1168, 1110, 1059, 1017, 978, 846.

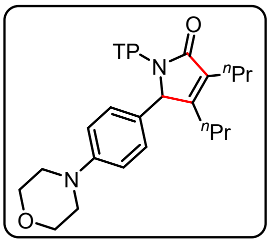

5-(4-Morpholinophenyl)-3,4-dipropyl-1-(2,4,4-trimethylpentan-2-yl)-1*H*-pyrrol-2(5*H*)-one (3k)
Light yellow solid (64.3 mg, 73% yield). m.p. 105-107 °C. Rf 0.22 (SiO_2, hexane/EtOAc = 40/1). **^{1}H NMR** (400 MHz, $CDCl_3$) δ 7.15 – 6.88 (m, 2H), 6.87 – 6.79 (m, 2H), 4.86 (s, 1H), 3.90 – 3.78 (m, 4H), 3.20 – 3.08 (m, 4H), 2.85 (d, J = 14.8 Hz, 1H), 2.23 – 2.09 (m, 3H), 1.68 – 1.60 (m, 1H), 1.56 – 1.45 (m, 3H), 1.40 (s, 3H), 1.31 – 1.25 (m, 1H), 1.20 – 1.13 (m, 4H), 0.94 – 0.88 (m, 12H), 0.85 (t, J = 7.3 Hz, 3H). **^{13}C NMR** (100 MHz, $CDCl_3$) δ 173.7, 154.3, 150.7, 132.7, 130.5, 67.0, 66.6, 58.9, 50.2, 49.2, 31.6, 31.4, 30.8, 28.2, 28.1, 25.8, 22.2, 22.1, 14.2, 14.1. **HRMS (ESI)** *m/z:* $[M+H]^+$ Calcd. for $C_{28}H_{45}N_2O_2$ 441.3476; Found 441.3479. **IR** (neat, cm^{-1}): 2983, 2959, 2933, 2905, 2869, 2841, 1676, 1659, 1610, 1518, 1486, 1477, 1463, 1448, 1396, 1387, 1375, 1362, 1343, 1332, 1271, 1259, 1239, 1223, 1179, 1120, 1109, 1092, 1067, 1050, 927, 809.

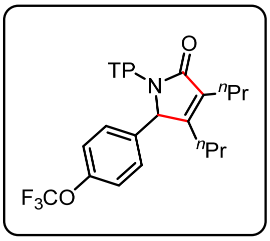

3,4-Dipropyl-5-(4-(trifluoromethoxy)phenyl)-1-(2,4,4-trimethylpentan-2-yl)-1*H*-pyrrol-2(5*H*)-one (3l)
White solid (74.7 mg, 85%). m.p. 76-78 °C. Rf 0.21 (SiO_2, hexane/EtOAc = 40/1). **^{1}H NMR** (400 MHz, CDCl3) δ 7.33 – 7.21 (m, 1H), 7.20 – 7.10 (m, 2H), 7.10 – 6.90 (m, 1H), 4.93 (s, 1H), 2.81 (d, J = 14.8 Hz, 1H), 2.49 – 2.36 (m, 3H), 1.66 – 1.42 (m, 4H), 1.40 (s, 3H), 1.27 (m, 1H), 1.19 (d, J = 14.8 Hz, 1H), 1.13 (s, 3H), 0.94 – 0.87 (m, 12H), 0.84 (t, J = 7.3 Hz, 3H). **^{13}C NMR** (100 MHz, CDCl3) δ 173.7, 153.7, 148.7, 138.7, 133.6, 129.5, 127.0, 120.5 (q, J = 261 Hz), 66.4, 59.2, 50.3, 31.6, 31.4, 30.9, 28.3, 28.1, 25.9, 22.2, 22.1, 14.3, 14.0. **^{19}F NMR** (376 MHz, $CDCl_3$) δ -63.4. **HRMS (ESI)** *m/z:* $[M+H]^+$ Calcd. for $C_{25}H_{37}F_3NO_2$ 440.2771; Found 440.2771. **IR** (neat, cm^{-1}): 2964, 2938, 2874, 1659, 1507, 1466, 1455, 1390, 1382, 1362, 1267, 1222, 1200, 1159, 1103.

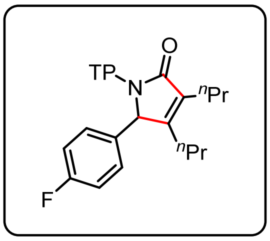

5-(4-Fluorophenyl)-3,4-dipropyl-1-(2,4,4-trimethylpentan-2-yl)-1*H*-pyrrol-2(5*H*)-one (3m)
Colourless oil (58.2 mg, 78% yield). Rf 0.28 (SiO_2, hexane/EtOAc = 40/1). **^{1}H NMR** (400 MHz, $CDCl_3$) δ 7.28 – 7.10 (m, 1H), 7.05 – 6.87 (m, 3H), 4.89 (s, 1H), 2.81 (d, J = 14.8 Hz, 1H), 2.20 – 2.08 (m, 3H), 1.65 – 1.55 (m, 1H), 1.54 – 1.42 (m, 3H), 1.37 (s, 3H), 1.29 – 1.20 (m, 1H), 1.17 (d, J = 14.8 Hz, 1H), 1.12 (s, 3H), 0.96 – 0.85 (m, 12H), 0.82 (t, J = 7.3 Hz, 3H). **^{13}C NMR** (100 MHz, $CDCl_3$) δ 173.6, 162.2 (d, J = 245.0 Hz), 153.9, 135.5 (d, J = 3.0 Hz), 133.3, 66.4, 59.0, 50.2, 31.5, 31.4, 30.8, 28.2, 28.0, 25.8, 22.1, 14.2, 14.0. **^{19}F NMR** (376 MHz, $CDCl_3$) δ -114.4. **HRMS (ESI)** *m/z:* $[M+H]^+$ Calcd. for $C_{24}H_{37}FNO$ 374.2854; Found 374.2851. **IR** (neat, cm^{-1}): 2957, 2907, 2872, 1673, 1655, 1617, 1602, 1506, 1487, 1469, 1456, 1441, 1417, 1390, 1383, 1375, 1365, 1353, 1328, 1319, 1310, 1295, 1277, 1260, 1238, 1222, 1197, 1177, 1156, 1128, 1112, 1093, 1061, 1033, 1015, 998, 977, 891, 878, 869, 854, 841, 829, 816, 804, 795, 773, 763, 734, 642, 635, 623, 570, 538, 529.

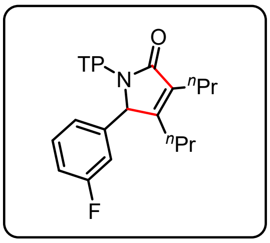

(3-Fluorophenyl)-3,4-dipropyl-1-(2,4,4-trimethylpentan-2-yl)-1*H*-pyrrol-2(5*H*)-one (3n)

Colourless oil (62.7 mg, 84% yield). Rf 0.28 (SiO_2, hexane/EtOAc = 40/1). **^{1}H NMR** (400 MHz, $CDCl_3$) δ 7.35 – 7.18 (m, 1H), 7.10 – 6.90 (m, 2H), 6.86 – 6.63 (m, 1H), 4.90 (s, 1H), 2.81 (d, J = 14.8 Hz, 1H), 2.23 – 2.09 (m, 3H), 1.70 – 1.44 (m, 4H), 1.42 (s, 3H), 1.31 – 1.23 (m, 1H), 1.20 (d, J = 12.0 Hz, 1H), 1.16 – 1.12 (m, 3H), 0.93 – 0.88 (m, 12H), 0.84 (t, J = 7.3 Hz, 3H). **^{13}C NMR** (100 MHz, $CDCl_3$) δ 173.7, 153.6, 142.8, 133.6, 130.4 (d, J = 5.0 Hz), 66.7, 59.1, 50.2, 31.6, 31.4, 30.8, 28.3, 28.1, 25.9, 22.2, 22.1, 14.3, 14.1. **^{19}F NMR** (376 MHz, $CDCl_3$) δ -117.3, -118.3. **HRMS (ESI)** *m/z:* $[M+H]^+$ Calcd. for $C_{24}H_{37}FNO$ 374.2854; Found 374.2853. **IR** (neat, cm^{-1}): 2958, 2934, 2871, 1682, 1678, 1674, 1667, 1659, 1655, 1651, 1613, 1590, 1486, 1464, 1454, 1451, 1389, 1381, 1364, 1327, 1300, 1275, 1251, 1239, 1225, 1186, 1168, 1138, 1124, 1111, 1092, 1074, 1001, 950, 930, 882, 801, 787, 756, 702.

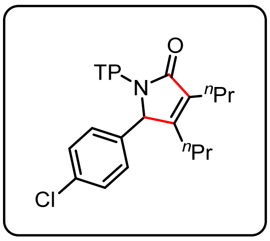

5-(4-Chlorophenyl)-3,4-dipropyl-1-(2,4,4-trimethylpentan-2-yl)-1*H*-pyrrol-2(5*H*)-one (3o)

Colourless oil (48.3 mg, 62% yield). Rf 0.29 (SiO_2, hexane/EtOAc = 40/1). **^{1}H NMR** (400 MHz, $CDCl_3$) δ 7.35 – 7.26 (m, 2H), 7.25 – 7.10 (m, 1H), 7.05 – 6.85 (m, 1H), 4.89 (s, 1H), 2.82 (d, J = 14.8 Hz, 1H), 2.19 (t, J = 7.8 Hz, 2H), 2.17 – 2.11 (m, 1H), 1.63 – 1.46 (m, 4H), 1.40 (s, 3H), 1.30 – 1.24 (m, 1H), 1.19 (d, J = 14.8 Hz, 1H), 1.14 (s, 3H), 0.93 – 0.89 (m, 12H), 0.85 (t, J = 7.3 Hz, 3H). **^{13}C NMR** (100 MHz, $CDCl_3$) δ 173.7, 153.7, 138.6, 133.5, 133.5, 129.0, 127.0, 66.5, 59.1, 50.2, 31.6, 31.5, 30.9, 28.3, 28.1, 25.8, 22.2, 22.1, 14.3, 14.1. **HRMS (ESI)** *m/z:* $[M+H]^+$ Calcd. for $C_{24}H_{37}ClNO$ 390.2558; Found 390.2560. **IR** (neat, cm^{-1}): 2958, 2934, 2905, 2871, 1673, 1666, 1658, 1489, 1464, 1455, 1377, 1364, 1353, 1259, 1238, 1221, 1170, 1111, 1101, 1089, 1015, 841, 821, 801.

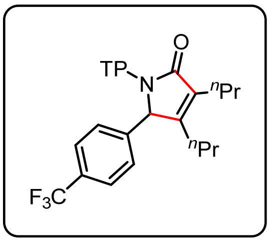

3,4-Dipropyl-5-(4-(trifluoromethyl)phenyl)-1-(2,4,4-trimethylpentan-2-yl)-1*H*-pyrrol-2(5*H*)-one (3p)

Colourless oil (42.3 mg, 50% yield). Rf 0.29 (SiO_2, hexane/EtOAc = 40/1). **^{1}H NMR** (400 MHz, $CDCl_3$) δ 7.63 – 7.52 (m, 2H), 7.30 – 7.45 (m, 1H), 7.25 – 7.05 (m, 1H), 4.98 (s, 1H), 2.82 (d, J = 14.8 Hz, 1H), 2.24 – 2.12 (m, 3H), 1.62 – 1.45 (m, 4H), 1.42 (s, 3H), 1.34 – 1.24 (m, 1H), 1.21 (d, J = 14.8 Hz, 1H), 1.12 (s, 3H), 0.94 – 0.88 (m, 12H), 0.86 (t, J = 7.3 Hz, 3H). **^{13}C NMR** (100 MHz,

$CDCl_3$) δ 173.8, 153.4, 144.4, 133.9, 130.2 (q, *J* = 32 Hz), 128.6, 125.9 (q, *J* = 3.8 Hz), 124.1 (q, *J* = 270 Hz), 66.6, 59.2, 50.2, 31.6, 31.4, 30.9, 28.3, 28.1, 25.8, 22.3, 22.1, 14.2, 14.0. **^{19}F NMR** (376 MHz, $CDCl_3$) δ -68.0. **HRMS (ESI)** *m/z:* $[M+H]^+$ Calcd. for $C_{25}H_{37}F_3NO$ 424.2822; Found 424.2826. **IR** (neat, cm^{-1}): 2960, 2936, 2872, 1678, 1674, 1671, 1617, 1465, 1419, 1377, 1364, 1324, 1237, 1223, 1164, 1129, 1106, 1066.

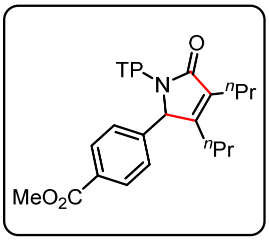

Methyl 4-(5-oxo-3,4-dipropyl-1-(2,4,4-trimethylpentan-2-yl)-2,5-dihydro-1*H*-pyrrol-2-yl)benzoate (3q)
White solid (46.3 mg, 56% yield). Rf 0.17 (SiO_2, hexane/EtOAc = 40/1). m.p. 91-93 °C. **^{1}H NMR** (400 MHz, $CDCl_3$) δ 8.07 – 7.90 (m, 2H), 7.40 – 7.30 (m, 1H), 7.15 – 7.03 (m, 1H), 4.97 (s, 1H), 3.90 (s, 3H), 2.81 (d, *J* = 14.8 Hz, 1H), 2.24 – 2.10 (m, 3H), 1.62 – 1.44 (m, 4H), 1.41 (s, 3H), 1.29 – 1.22 (m, 1H), 1.19 (d, *J* = 14.8 Hz, 1H), 1.11 (s, 3H), 0.93 – 0.87 (m, 12H), 0.83 (t, *J* = 7.3 Hz, 3H). **^{13}C NMR** (100 MHz, $CDCl_3$) δ 173.7, 166.8, 153.5, 145.5, 133.8, 130.2, 129.8, 66.8, 59.2, 52.3, 50.2, 31.6, 31.4, 30.8, 28.3, 28.1, 25.9, 22.2, 22.1, 14.2, 14.1. **HRMS (ESI)** *m/z:* $[M+H]^+$ Calcd. for $C_{26}H_{40}NO_3$ 414.3003; Found 414.3005. **IR** (neat, cm^{-1}): 3003, 2963, 2937, 2872, 1724, 1660, 1608, 1466, 1448 1437, 1417, 1389, 1378, 1362, 1279, 1258, 1238, 1222, 1191, 1176, 1112, 1102, 1018.

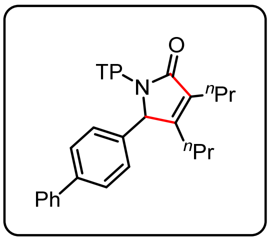

5-([1,1'-Biphenyl]-4-yl)-3,4-dipropyl-1-(2,4,4-trimethylpentan-2-yl)-1*H*-pyrrol-2(5*H*)-one (3r)
Colourless oil (70.7 mg, 82% yield). Rf 0.29 (SiO_2, hexane/EtOAc = 40/1). **^{1}H NMR** (400 MHz, $CDCl_3$) δ 7.62 – 7.52 (m, 4H), 7.45 – 7.42 (m, 2H), 7.36 – 7.32 (m, 2H), 7.14 – 7.02 (m, 1H), 4.97 (s, 1H), 2.88 (d, *J* = 14.8 Hz, 1H), 2.24 – 2.14 (m, 3H), 1.71 – 1.64 (m, 1H), 1.62 – 1.48 (m, 3H), 1.45 (s, 3H), 1.39 – 1.25 (m, 1H), 1.23 – 1.18 (m, 4H), 0.96 – 0.90 (m, 12H), 0.87 (t, *J* = 7.3 Hz, 3H). **^{13}C NMR** (100 MHz, $CDCl_3$) δ 173.7, 154.0, 140.6, 140.5, 138.8, 133.2, 128.9, 127.5, 127.2, 127.0, 125.8, 66.9, 59.0, 50.2, 31.6, 31.4, 30.8, 28.3, 28.1, 25.8, 22.2, 22.1, 14.3, 14.1. **HRMS (ESI)** *m/z:* $[M+H]^+$ Calcd. for $C_{30}H_{42}NO$ 432.3261; Found 432.3261. **IR** (neat, cm^{-1}): ν =3249, 3238, 3228, 3058, 3029, 2958, 2935, 2907, 2871, 1678, 1674, 1651, 1609, 1601, 1485, 1464, 1455, 1388, 1377, 1364, 1352, 1328, 1314, 1291, 1272, 1257, 1236, 1222, 1168, 1129, 1108, 1091, 1076, 1007,847, 828, 763, 736, 697.

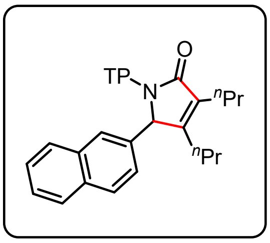

5-(Naphthalen-2-yl)-3,4-dipropyl-1-(2,4,4-trimethylpentan-2-yl)-1*H*-pyrrol-2(5*H*)-one (3s)
White solid (64.8 mg, 80% yield). m.p. 101-103 °C. Rf 0.29 (SiO_2, hexane/EtOAc = 40/1). **^{1}H NMR** (400 MHz, $CDCl_3$) δ 7.90 – 7.65 (m, 4H), 7.50 – 7.05 (m, 3H), 5.11 (s, 1H), 2.89 (d, J = 14.8 Hz, 1H), 2.24 – 2.23 (m, 2H), 2.21 – 2.08 (m, 1H), 1.67 – 1.47 (m, 4H), 1.42 (s, 3H), 1.38 – 1.25 (m, 1H), 1.22 – 1.13 (m, 4H), 0.95 – 0.92 (m, 12H), 0.84 (t, J = 7.1 Hz, 3H). **^{13}C NMR** (100 MHz, $CDCl_3$) δ 173.8, 153.6, 137.5, 133.8, 133.5, 133.2, 128.8, 127.9, 127.6, 127.3, 126.5, 126.2, 123.2, 67.3, 59.2, 50.2, 31.6, 31.5, 30.9, 28.3, 28.0, 25.9, 22.2, 1.2. **HRMS (ESI)** *m/z:* $[M+H]^+$ Calcd. for $C_{28}H_{40}NO$ 406.3104; Found 406.3105. **IR** (neat, cm^{-1}): 2958, 2908, 2868, 1678, 1674 1670, 1667, 1473, 1463, 1455, 1375, 1362, 1350, 1341, 1261, 1236, 1219, 1164, 1107, 826, 754, 482.

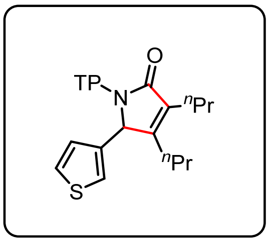

3,4-Dipropyl-5-(thiophen-3-yl)-1-(2,4,4-trimethylpentan-2-yl)-1*H*-pyrrol-2(5*H*)-one (3t)
Colourless oil (20.2 mg, 28% yield). Rf 0.18 (SiO_2, hexane/EtOAc = 40/1). **^{1}H NMR** (400 MHz, $CDCl_3$) δ 7.25 – 7.23 (m, 1H), 7.12 – 7.07 (m, 1H), 6.79 –6.74 (m, 1H), 5.07 (s, 1H), 2.81 (d, J = 14.8 Hz, 1H), 2.22 – 2.13 (m, 3H), 1.74 – 1.66 (m, 1H), 1.57 – 1.45 (m, 3H), 1.41 (s, 3H), 1.30 – 1.25 (m, 1H), 1.23 – 1.18 (m, 4H), 0.93 – 0.88 (m, 12H), 0.85 (t, J = 7.3 Hz, 3H). **^{13}C NMR** (100 MHz, $CDCl_3$) δ 173.4, 153.1, 141.0, 133.3, 126.4, 125.8, 121.9, 62.7, 59.0, 50.2, 31.6, 31.4, 30.7, 28.2, 27.9, 25.9, 22.2, 22.1, 14.3, 14.1. **HRMS (ESI)** *m/z:* $[M+H]^+$ Calcd. for $C_{22}H_{36}NOS$ 362.2512; Found 362.2513. **IR** (neat, cm^{-1}): 2959, 2934, 2871, 1678, 1674, 1671, 1667, 1464, 1456, 1390, 1376, 1364, 1350, 1253, 1237, 1224, 1180, 1166, 1109, 832, 777.

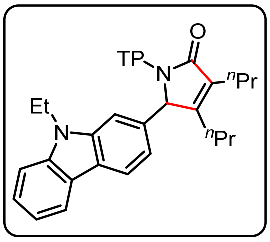

5-(9-Ethyl-9H-carbazol-2-yl)-3,4-dipropyl-1-(2,4,4-trimethylpentan-2-yl)-1*H*-pyrrol-2(5*H*)-one (3u)
White solid (89.7 mg, 95%). m.p. 107-109 °C. Rotamer mixtures. Rf 0.20 (SiO_2, hexane/EtOAc = 40/1). **^{1}H NMR** (400 MHz, $CDCl_3$) δ 8.13 (d, J = 7.7 Hz, 0.5H), 7.99 (d, J = 7.7 Hz, 0.5H), 7.97 (s, 0.5H), 7.72 (s, 0.5H), 7.51 – 7.29 (m, 4H), 7.29 – 7.15 (m, 1H), 7.06 (d, J = 8.4 Hz, 0.5H), 5.14 (s, 0.5H), 5.10 (s, 0.5H), 4.35 (q, J = 7.2 Hz, 2H), 2.92 (d, J = 14.7 Hz, 1H), 2.37 – 2.21 (m, 2H), 2.21 – 1.99 (m, 1H), 1.74 – 1.49 (m, 4H), 1.48 – 1.40 (m, 6H), 1.39 – 1.27 (m, 1H), 1.24 – 1.13 (m, 4H), 1.00 – 0.91 (m, 12H), 0.84 (t, J = 7.2 Hz, 3H). **^{13}C NMR** (100 MHz, $CDCl_3$) δ 174.0, 173.8, 155.2, 154.7, 140.5, 140.2, 139.7, 139.5, 132.7, 132.4, 129.7, 129.7, 126.4, 126.2, 125.9, 125.5, 123.7, 123.1, 122.7, 122.6, 120.7, 120.5, 120.4, 119.1, 119.0, 117.2, 109.2, 108.8, 108.6, 108.1, 67.7, 67.5, 59.1, 50.3, 37.8, 31.8, 31.6, 31.5, 31.0, 30.9, 28.4, 28.3, 28.2, 28.1, 25.9, 22.3, 22.2, 14.3, 14.1, 14.0, 13.9. **HRMS (ESI)** *m/z:* $[M+H]^+$ Calcd. for $C_{32}H_{45}N_2O$ 473.3526; Found 473.3528. **IR** (neat, cm^{-1}): 2959, 2933, 2903, 2870, 1663, 1600, 1484, 1473, 1462, 1383, 1376, 1363, 1348, 1330, 1232, 1164, 1150, 748.

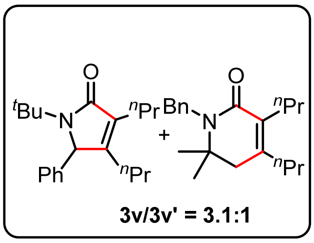

1-(*tert*-Butyl)-5-phenyl-3,4-dipropyl-1*H*-pyrrol-2(5*H*)-one (3v)

Colourless oil (inseparable mixture of **3v** and **3v′** (3.1:1, determined by ^{1}H NMR, 58.6 mg, 98% yield). Rf 0.29 (SiO_2, hexane/EtOAc = 40/1). **^{1}H NMR** (400 MHz, $CDCl_3$) δ 7.34 – 7.23 (m, 4.74H), 7.22 – 6.95 (m, 2.03H), 4.94 (s, 1H), 4.66 (s, 0.66H), 2.42 – 2.36 (m, 0.65H), 2.28 – 2.09 (m, 4.61H), 1.68 – 1.61 (m, 1H), 1.57 – 1.50 (m, 2H), 1.50 – 1.43 (m, 2H), 1.33 (s, 9H), 1.28 – 1.22 (m, 1H), 1.17 (s, 2H), 0.99 – 0.88 (m, 5.31H), 0.84 (t, J = 7.3 Hz, 3H). **^{13}C NMR** (100 MHz, $CDCl_3$) δ 173.8, 154.3, 143.9, 140.7, 139.9, 132.7, 128.9, 128.4, 127.8, 127.3, 126.6, 66.4, 55.3, 55.2, 44.9, 43.4, 35.9, 28.9, 28.7, 28.3, 26.7, 25.8, 23.3, 22.2, 22.2, 20.8, 14.5, 14.4, 14.2. **HRMS (ESI)** *m/z:* $[M+H]^+$ Calcd. for $C_{21}H_{32}NO$ 300.2322; Found 300.2320. **IR** (neat, cm^{-1}): 2960, 2931, 2871, 1678, 1620, 1463, 1455, 1402, 1393, 1377, 1366, 1238, 1218, 702.

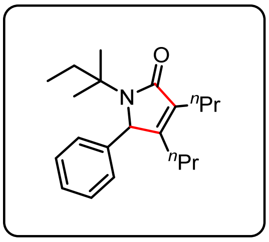

1-(*tert*-Pentyl)-5-phenyl-3,4-dipropyl-1*H*-pyrrol-2(5*H*)-one (3w)

Colourless oil (48.2 mg, 77% yield). Rf 0.28 (SiO_2, hexane/EtOAc = 40/1). **^{1}H NMR** (400 MHz, $CDCl_3$) δ 7.43 – 7.22 (m, 3H), 7.23 – 6.89 (m, 2H), 4.91 (s, 1H), 2.39 – 2.06 (m, 4H), 1.74 – 1.49 (m, 4H), 1.49 – 1.37 (m, 1H), 1.34 – 1.19 (m, 4H), 1.12 (s, 3H), 0.91 (t, J = 7.4 Hz, 3H), 0.83 (t, J = 7.3 Hz, 3H), 0.74 (t, J = 7.4 Hz, 3H). **^{13}C NMR** (100 MHz, $CDCl_3$) δ 173.8, 154.3, 139.9, 132.6, 128.8, 127.8, 67.2, 58.4, 32.5, 28.2, 27.3, 25.8, 25.7, 22.2, 22.1, 14.2, 14.1, 8.8. **HRMS (ESI)** *m/z:* $[M+H]^+$ Calcd. for $C_{21}H_{32}NO$ 314.2478; Found 314.2476. **IR** (neat, cm^{-1}): 3237, 3226, 3105, 3087, 3063, 3029, 2961, 2933, 2872, 1682, 1678, 1674, 1667, 1662, 1659, 1651, 1601, 1494, 1462, 1455, 1388, 1378 , 1365, 1352, 1337, 1318, 1302, 1291, 1243, 1199, 1185, 1142, 1115, 1090, 1076, 1029, 767, 739.

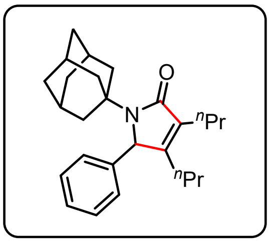

1-(Adamantan-1-yl)-5-phenyl-3,4-dipropyl-1*H*-pyrrol-2(5*H*)-one (3x)

White solid (43.8 mg, 58%). m.p. 80-82 °C. Rf 0.28 (SiO_2, hexane/EtOAc = 40/1). **^{1}H NMR** (400 MHz, $CDCl_3$) δ 7.35 – 7.23 (m, 3H), 7.22 – 6.92 (m, 2H), 4.99 (s, 1H), 2.29 – 2.02 (m, 9H), 1.96 (s, 3H), 1.67 – 1.47 (m, 9H), 1.47 – 1.37 (m, 1H), 1.29 – 1.17 (m, 1H), 0.90 (t, J = 7.4 Hz, 3H), 0.83 (t, J = 7.3 Hz, 3H). **^{13}C NMR** (100 MHz, $CDCl_3$) δ 173.9, 154.5, 140.2, 132.6, 128.8, 127.7, 65.5, 56.8, 40.3, 36.4, 29.8, 28.3, 25.7, 22.2, 14.2. **HRMS (ESI)** *m/z:* $[M+H]^+$ Calcd. for $C_{26}H_{36}NO$ 378.2791;

Found 378.2793. **IR** (neat): 2960, 2909, 2866, 2859, 2851, 1662, 1658, 1655, 1453, 1376, 1360, 1339, 1308, 1208, 1184, 1119, 700.

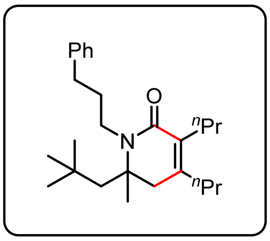

6-Methyl-6-neopentyl-1-(3-phenylpropyl)-3,4-dipropyl-5,6-dihydropyridin-2(1*H*)-one (3y′)
Colourless oil (15.3 mg, 20% yield). Rf 0.28 (SiO_2, hexane/EtOAc = 40/1). **^{1}H NMR** (400 MHz, $CDCl_3$) δ 7.29 – 7.25 (m, 2H), 7.23 – 7.14 (m, 3H), 3.71 – 3.62 (m, 1H), 3.02 – 2.92 (m, 1H), 2.76 – 2.66 (m, 1H), 2.64 – 2.54 (m, 1H), 2.52 – 2.43 (m, 1H), 2.38 – 2.23 (m, 3H), 2.23 – 1.99 (m, 3H), 1.78 – 1.68 (m, 1H), 1.63 (t, *J* = 7.3 Hz, 2H), 1.48 – 1.36 (m, 4H), 1.33 (s, 3H), 0.96 – 0.89 (m, 15H). **^{13}C NMR** (100 MHz, $CDCl_3$) δ 166.2, 143.0, 142.2, 129.9, 128.5, 128.4, 125.8, 59.0, 49.0, 42.5, 41.8, 36.0, 33.9, 32.2, 31.7, 31.2, 28.6, 27.8, 23.2, 20.7, 14.5, 14.3. **HRMS (ESI)** *m/z:* $[M+Na]^+$ Calcd. for $C_{26}H_{41}NONa$ 406.3080; Found 406.3082.

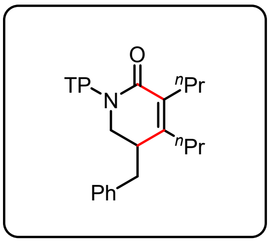

5-Benzyl-3,4-dipropyl-1-(2,4,4-trimethylpentan-2-yl)-5,6-dihydropyridin-2(1*H*)-one (3y″)
Colourless oil (47.6 mg, 62% yield). Rf 0.29 (SiO_2, hexane/EtOAc = 40/1). **^{1}H NMR** (400 MHz, $CDCl_3$) δ 7.34 – 7.28 (m, 2H), 7.25 – 7.17 (m, 3H), 3.32 (dd, *J* = 12.7, 1.6 Hz, 1H), 3.15 (dd, *J* = 12.6, 4.0 Hz, 1H), 2.77 (dd, *J* = 14.0, 4.1 Hz, 1H), 2.67 (d, *J* = 14.7 Hz, 1H), 2.59 (dd, *J* = 14.0, 10.5 Hz, 1H), 2.45 – 2.30 (m, 3H), 2.20 – 2.10 (m, 1H), 1.94 – 1.84 (m, 1H), 1.58 (s, 3H), 1.54 – 1.37 (m, 5H), 1.22 (s, 3H), 0.99 – 0.91 (m, 15H). **^{13}C NMR** (100 MHz, $CDCl_3$) δ 166.6, 148.5, 140.0, 132.7, 129.0, 128.7, 126.5, 60.4, 50.0, 44.8, 39.8, 36.3, 33.9, 31.7, 31.7, 31.1, 29.3, 23.3, 22.4, 14.5, 14.5. **HRMS (ESI)** *m/z:* $[M+Na]^+$ Calcd. for $C_{26}H_{41}NONa$ 406.3080; Found 406.3085.

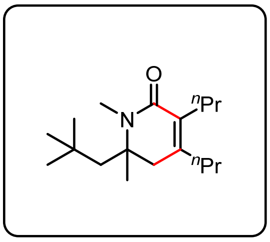

1,6-Dimethyl-6-neopentyl-3,4-dipropyl-5,6-dihydropyridin-2(1*H*)-one (3z′)
Colourless oil (11.2 mg, 20% yield). Rf 0.27 (SiO_2, hexane/EtOAc = 40/1). **^{1}H NMR** (400 MHz, $CDCl_3$) δ 2.89 (s, 3H), 2.49 – 2.40 (m, 1H), 2.36 – 2.27 (m, 3H), 2.25 – 2.16 (m, 1H), 2.09 – 2.00 (m, 1H), 1.64 (d, *J* = 14.7 Hz, 1H), 1.53 – 1.36 (m, 5H), 1.36 (s, 3H), 0.98 – 0.88 (m, 15H). **^{13}C NMR** (100 MHz, $CDCl_3$) δ 166.2, 143.1, 129.6, 58.1, 47.9, 41.0, 36.0, 31.7, 31.2, 28.7, 28.0, 27.5, 23.2, 20.7, 14.4, 14.3. **HRMS (ESI)** *m/z:* $[M+H]^+$ Calcd. for $C_{18}H_{33}NONa$ 302.2454; Found 302.2458.

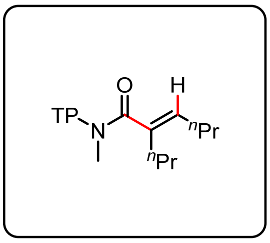

(*E*)-*N*-Methyl-2-propyl-*N*-(2,4,4-trimethylpentan-2-yl)hex-2-enamide (3z″)
Colourless oil (39.2 mg, 70% yield). Rf 0.27 (SiO_2, hexane/EtOAc = 35/1). **^{1}H NMR** (400 MHz, $CDCl_3$) δ 5.42 (t, J = 7.3 Hz, 1H), 2.94 (s, 3H), 2.25 – 2.19 (m, 2H), 2.08 – 2.02 (m, 2H), 2.00 (s, 2H), 1.45 (s, 6H), 1.44 – 1.37 (m, 4H), 0.99 (s, 9H), 0.94 – 0.89 (m, 6H). **^{13}C NMR** (100 MHz, $CDCl_3$) δ 174.7, 139.4, 129.9, 60.5, 49.8, 35.0, 31.7, 30.9, 29.7, 29.2, 22.6, 21.9, 14.4, 14.1. **HRMS (ESI)** *m/z:* $[M+Na]^+$ Calcd. for $C_{18}H_{35}NONa$ 304.2611; Found 304.2615.

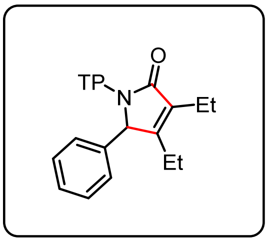

3,4-Diethyl-5-phenyl-1-(2,4,4-trimethylpentan-2-yl)-1*H*-pyrrol-2(5*H*)-one (4a)
Colourless oil (55.6 mg, 85% yield). Rf 0.27 (SiO_2, hexane/EtOAc = 40/1). **^{1}H NMR** (400 MHz, $CDCl_3$) δ 7.30 – 7.26 (m, 4H), 7.10 – 6.95 (m, 1H), 4.93 (s, 1H), 2.82 (d, J = 14.8 Hz, 1H), 2.33 – 2.15 (m, 3H), 1.71–1.64 (m, 1H), 1.42 (s, 3H), 1.21 (d, J = 14.8 Hz, 1H), 1.15 (s, 3H), 1.07 (t, J = 7.5 Hz, 3H), 0.94 – 0.88 (m, 12H). **^{13}C NMR** (100 MHz, $CDCl_3$) δ 173.7, 155.0, 139.6, 133.9, 128.8, 127.8, 125.5, 67.0, 59.0, 50.3, 31.6, 31.4, 30.7, 28.3, 19.3, 16.9, 13.8, 13.7. **HRMS (ESI)** *m/z:* $[M+H]^+$ Calcd. for $C_{22}H_{34}NO$ 328.2635; Found 328.2634. **IR** (neat, cm^{-1}): 2962, 2936, 2904, 2873, 1677, 1674, 1667, 1663, 1652, 1635, 1616, 1601, 1484, 1462, 1455, 1388, 1364, 1333, 1264, 1254, 1225, 1185, 1170, 1129, 1109, 1076, 1059, 780, 760, 750, 702.

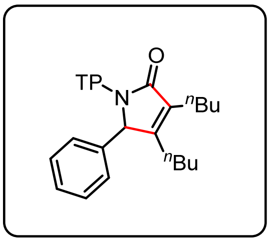

3,4-Dibutyl-5-phenyl-1-(2,4,4-trimethylpentan-2-yl)-1*H*-pyrrol-2(5*H*)-one (4b)
Colourless oil (61.3 mg, 80% yield). Rf 0.28 (SiO_2, hexane/EtOAc = 40/1). **^{1}H NMR** (400 MHz, $CDCl_3$) δ 7.30 – 7.20 (m, 4H), 7.10 – 6.90 (m, 1H), 4.91 (s, 1H), 2.83 (d, J = 14.8 Hz, 1H), 2.26 – 2.12 (m, 3H), 1.69 – 1.55 (m, 2H), 1.51 – 1.44 (m, 2H), 1.41 (s, 3H), 1.36 – 1.30 (m, 2H), 1.27 – 1.17 (m, 4H), 1.14 (s, 3H), 0.93 – 0.81 (m, 15H). **^{13}C NMR** (100 MHz, $CDCl_3$) δ 173.8, 154.1, 139.8, 133.0, 128.7, 127.7, 125.4, 67.2, 59.0, 50.2, 31.6, 31.4, 31.1, 31.1, 30.8, 28.2, 25.8, 23.6, 22.9, 22.6, 14.0, 13.9. **HRMS (ESI)** *m/z:* $[M+H]^+$ Calcd. for $C_{26}H_{42}NO$ 384.3261; Found 384.3261. **IR** (neat, cm^{-1}): 2956, 2933, 2872, 2861, 1678, 1466, 1454, 1388, 1378, 1364, 1228, 1167, 702.

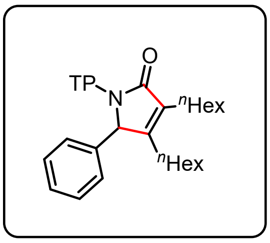

3,4-Dihexyl-5-phenyl-1-(2,4,4-trimethylpentan-2-yl)-1*H*-pyrrol-2(5*H*)-one (4c)

Colourless oil (61.5 mg, 70% yield). Rf 0.29 (SiO_2, hexane/EtOAc = 40/1). **^{1}H NMR** (400 MHz, $CDCl_3$) δ 7.35 – 7.20 (m, 4H), 7.10 – 6.92 (m, 1H), 4.91 (s, 1H), 2.84 (d, J = 14.8 Hz, 1H), 2.28 – 2.10 (m, 3H), 1.69 – 1.56 (m, 1H), 1.55 – 1.45 (m, 2H), 1.42 (s, 3H), 1.35 – 1.17 (m, 15H), 1.14 (s, 3H), 0.91 (s, 9H), 0.89 – 0.84 (m, 6H). **^{13}C NMR** (100 MHz, $CDCl_3$) δ 173.8, 154.1, 139.8, 133.1, 128.8, 127.7, 67.2, 59.0, 50.2, 31.8, 31.6, 31.4, 30.8, 29.5, 29.1, 28.9, 28.8, 28.3, 26.1, 23.9, 22.8, 22.6, 14.2, 14.1. **HRMS (ESI)** *m/z:* $[M+H]^+$ Calcd. for $C_{30}H_{50}NO$ 440.3887; Found 440.3886. **IR** (neat, cm^{-1}): 2954, 2929, 2870, 2858, 1682, 1679, 1466, 1455, 1387, 1379, 1364, 1227, 702.

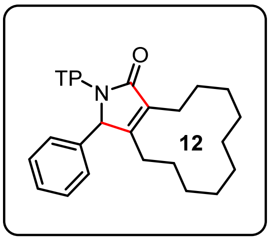

3-Phenyl-2-(2,4,4-trimethylpentan-2-yl)-2,3,4,5,6,7,8,9,10,11,12,13-dodecahydro-1*H*-cyclododeca[c]pyrrol-1-one (4d)

Colourless oil (63.9 mg, 78% yield). Rf 0.27 (SiO_2, hexane/EtOAc = 40/1). **^{1}H NMR** (400 MHz, $CDCl_3$) δ 7.36 – 7.20 (m, 4H), 7.05 – 6.94 (m, 1H), 4.96 (s, 1H), 2.93 (d, J = 14.8 Hz, 1H), 2.45 – 2.30 (m, 2H), 2.25 – 2.15 (m, 1H), 1.86 – 1.72 (m, 1H), 1.71 – 1.52 (m, 4H), 1.52 – 1.40 (m, 6H), 1.39 – 1.33 (m, 3H), 1.30 – 1.25 (m, 4H), 1.23 – 1.16 (m, 2H), 1.16 – 1.06 (m, 4H), 0.92 (s, 9H). **^{13}C NMR** (100 MHz, $CDCl_3$) δ 173.8, 155.0, 139.9, 132.4, 128.7, 127.8, 125.4, 67.0, 58.8, 50.1, 31.6, 31.4, 31.0, 28.2, 26.1, 25.8, 25.4, 25.4, 24.2, 23.5, 23.0, 22.5, 21.6, 21.2. **HRMS (ESI)** *m/z:* $[M+H]^+$ Calcd. for $C_{28}H_{44}NO$ 410.3417; Found 410.3419. **IR** (neat, cm^{-1}): 3085, 3061, 3027, 2929, 2862, 1722, 1682, 1678, 1673, 1667, 1661, 1652, 1600, 1469, 1454, 1417, 1379, 1364, 1351, 1337, 1301, 1287, 1276, 1259, 1247, 1226, 1203, 1170, 1153, 1145, 1136, 1126, 1116, 1095, 1075, 1028, 1001, 975, 970, 922, 910, 839, 808, 770, 757, 730, 701, 663, 652, 645, 638, 607, 515.

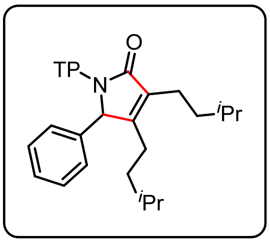

3,4-Diisopentyl-5-phenyl-1-(2,4,4-trimethylpentan-2-yl)-1*H*-pyrrol-2(5*H*)-one (4e)

Colourless oil (64.2 mg, 78% yield). Rf 0.29 (SiO_2, hexane/EtOAc = 40/1). **^{1}H NMR** (400 MHz, $CDCl_3$) δ 7.36 – 7.22 (m, 4H), 7.15 – 6.93 (m, 1H), 4.93 (s, 1H), 2.83 (d, J = 14.8 Hz, 1H), 2.27 – 2.13 (m, 3H), 1.66 – 1.57 (m, 2H), 1.50 – 1.43 (m, 4H), 1.42 – 1.34 (m, 2H), 1.33 – 1.28 (m, 1H), 1.22 (d, J = 14.8 Hz, 1H), 1.16 (s, 3H), 1.13 – 1.05 (m, 1H), 0.97 – 0.92 (m, 15H), 0.86 (d, J = 6.6 Hz, 3H), 0.83 (d, J = 6.6 Hz, 3H). **^{13}C NMR** (100 MHz, $CDCl_3$) δ 173.8, 154.2, 139.8, 133.1, 128.8,

127.8, 125.4, 67.3, 59.0, 50.3, 38.1, 38.0, 31.6, 31.4, 30.7, 28.3, 28.1, 24.1, 22.6, 22.6, 22.2, 21.8. **HRMS (ESI)** *m/z:* $[M+H]^+$ Calcd. for $C_{28}H_{46}NO$ 412.3574; Found 412.3575. **IR** (neat, cm^{-1}): 2956, 2934, 2868, 1674, 1666, 1655, 1617, 1482, 1467, 1455, 1393, 1379, 1366, 1259, 1227, 1214, 1204, 1179, 698.

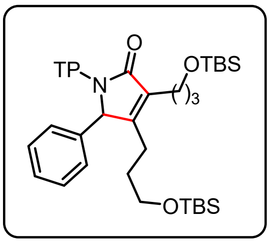

3,4-Bis(3-((*tert*-butyldimethylsilyl)oxy)propyl)-5-phenyl-1-(2,4,4-trimethylpentan-2-yl)-1*H*-pyrrol-2(5*H*)-one (4f)

White solid (88.6 mg, 72% yield). m.p. 89-91 °C. Rf 0.21 (SiO_2, hexane/EtOAc = 40/1). **^{1}H NMR** (400 MHz, $CDCl_3$) δ 7.35 – 7.22 (m, 4H), 7.15– 6.92 (m, 1H), 4.94 (s, 1H), 3.66 – 3.58 (m, 2H), 3.59 – 3.42 (m, 2H), 2.83 (d, J = 14.7 Hz, 1H), 2.36 – 2.18 (m, 3H), 1.81 – 1.56 (m, 4H), 1.48 – 1.37 (m, 4H), 1.22 (d, J = 14.8 Hz, 1H), 1.16 (s, 3H), 0.92 (s, 9H), 0.90 – 0.86 (m, 18H), 0.08 – 0.00 (m, 12H). **^{13}C NMR** (100 MHz, $CDCl_3$) δ 173.6, 154.1, 139.6, 132.8, 128.9, 127.8, 67.4, 62.9, 62.5, 59.1, 50.3, 32.1, 31.9, 31.6, 31.5, 30.7, 28.2, 26.1, 26.1, 22.5, 20.1, 18.4, 18.4, -5.1, -5.2. **HRMS (ESI)** *m/z:* $[M+H]^+$ Calcd. for $C_{36}H_{66}NO_3Si_2$ 616.4576; Found 616.4573. **IR** (neat, cm^{-1}): 2954, 2929, 2892, 2858, 1652, 1487, 1471, 1463, 1456, 1448, 1384, 1361, 1255, 1227, 1198, 1168, 1111, 1097, 958, 836, 813, 775, 754, 703.

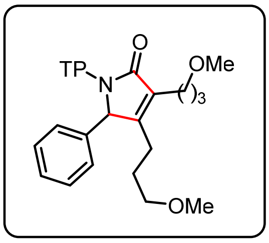

3,4-Bis(3-methoxypropyl)-5-phenyl-1-(2,4,4-trimethylpentan-2-yl)-1*H*-pyrrol-2(5*H*)-one (4g)

Colourless oil (53.2 mg, 64% yield). Rf 0.20 (SiO_2, hexane/EtOAc = 40/1). **^{1}H NMR** (400 MHz, $CDCl_3$) δ 7.38 – 7.20 (m, 4H), 7.10 – 69 (m, 1H), 4.95 (s, 1H), 3.42 – 3.35 (m, 2H), 3.34 – 3.19 (m, 8H), 2.84 (d, J = 14.8 Hz, 1H), 2.34 – 2.23 (m, 3H), 1.85 – 1.75 (m, 2H), 1.74– 1.63 (m, 2H), 1.58 – 1.45 (m, 1H), 1.41 (s, 3H), 1.20 (d, J = 14.9 Hz, 1H), 1.14 (s, 3H), 0.91 (s, 9H). **^{13}C NMR** (100 MHz, $CDCl_3$) δ 173.5, 153.9, 139.5, 132.8, 128.9, 127.9, 125.3, 72.3, 71.8, 67.3, 59.1, 58.6, 58.6, 50.2, 31.6, 31.4, 30.7, 28.7, 28.5, 28.2, 22.5, 20.3. **HRMS (ESI)** *m/z:* $[M+H]^+$ Calcd. for $C_{26}H_{42}NO_3$ 416.3159; Found 416.3160. **IR** (neat, cm^{-1}): 2951, 2926, 2895, 2870, 1675, 1454, 1388, 1380, 1364, 1227, 1119.

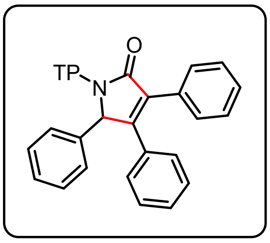

3,4,5-Triphenyl-1-(2,4,4-trimethylpentan-2-yl)-1*H*-pyrrol-2(5*H*)-one (4h)

White solid (16.9 mg, 20%). m.p. 102-104 °C. Rf 0.23 (SiO_2, hexane/EtOAc = 40/1). **^{1}H NMR** (400

MHz, $CDCl_3$) δ 7.42 – 7.37 (m, 2H), 7.26 – 7.08 (m, 11H), 6.94 – 6.89 (m, 2H), 5.51 (s, 1H), 2.88 (d, J = 14.8 Hz, 1H), 1.52 (s, 3H), 1.34 (d, J = 14.8 Hz, 1H), 1.30 (s, 3H), 0.99 (s, 9H). **^{13}C NMR** (100 MHz, $CDCl_3$) δ 171.59, 153.36, 138.51, 133.17, 132.71, 131.65, 131.55, 129.82, 128.80, 128.66, 128.46, 128.30, 128.16, 127.92, 126.70, 77.48, 77.16, 76.84, 67.95, 59.92, 50.31, 31.72, 31.55, 30.30, 28.28. **HRMS (ESI)** *m/z:* $[M+H]^+$ Calcd. for $C_{30}H_{34}NO$ 424.2635; Found 424.2640. **IR** (neat, cm^{-1}): 2961, 2953, 1678, 1454, 1441, 1389, 1365, 1218, 1207, 1196, 1178, 794, 786, 744, 696.

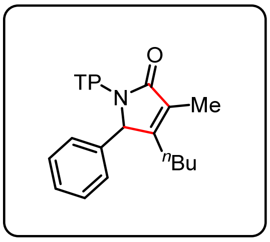

4-Butyl-3-methyl-5-phenyl-1-(2,4,4-trimethylpentan-2-yl)-1*H*-pyrrol-2(5*H*)-one (4i)

Colourless oil (37.5 mg, 55% yield). Rf 0.27 (SiO_2, hexane/EtOAc = 40/1). **^{1}H NMR** (400 MHz, $CDCl_3$) δ 7.32 – 7.11 (m, 4H), 7.10 – 6.85 (m, 1H), 4.72 (s, 1H), 2.71 (d, J = 14.7 Hz, 1H), 2.22 – 2.04 (m, 2H), 1.49 (s, 3H), 1.45 – 1.32 (m, 5H), 1.28 – 2.21 (m, 2H), 1.17 (d, J = 14.8 Hz, 1H), 1.07 (s, 3H), 0.87 – 0.78 (m, 12H). **^{13}C NMR** (100 MHz, $CDCl_3$) δ 173.8, 149.8, 139.9, 133.0, 129.0, 127.8, 125.2, 69.1, 59.1, 50.4, 31.6, 31.5, 30.8, 30.7, 28.3, 23.5, 22.8, 14.0, 12.0. **HRMS (ESI)** *m/z:* $[M+H]^+$ Calcd. for $C_{23}H_{36}NO$ 342.2791; Found 342.2789. **IR** (neat, cm^{-1}): 2955, 2942, 2872, 2858, 1662, 1655, 1486, 1474, 1465, 1454, 1391, 1366, 1362, 1261, 1249, 1227, 1178, 1170, 1132, 1108, 1074, 1029, 763, 755, 703.

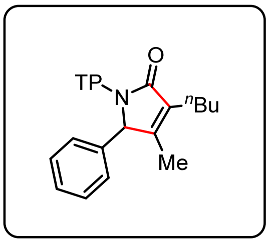

3-Butyl-4-methyl-5-phenyl-1-(2,4,4-trimethylpentan-2-yl)-1*H*-pyrrol-2(5*H*)-one (4i′)

Colourless oil (25.3 mg, 37% yield). Rf 0.26 (SiO_2, hexane/EtOAc = 40/1). **^{1}H NMR** (400 MHz, $CDCl_3$) δ 7.37 – 7.17 (m, 5H), 7.14 – 6.88 (m, 1H), 4.91 (s, 1H), 2.79 (d, J = 14.8 Hz, 1H), 2.18 – 2.10 (m, 1H), 1.78 (s, 3H), 1.72 – 1.59 (m, 2H), 1.42 (s, 3H), 1.39 – 1.30 (m, 1H), 1.29 – 1.16 (m, 3H), 1.15 (s, 3H), 0.90 (s, 3H), 0.83 (t, J = 7.0 Hz, 3H). **^{13}C NMR** (100 MHz, $CDCl_3$) δ 174.2, 154.1, 139.7, 128.8, 128.7, 127.8, 67.6, 59.2, 50.4, 31.6, 31.4, 30.7, 30.6, 28.2, 26.0, 22.5, 13.9, 8.8. **HRMS (ESI)** *m/z:* $[M+H]^+$ Calcd. for $C_{23}H_{36}NO$ 342.2791; Found 342.2792. **IR** (neat, cm^{-1}): 2961, 2933, 2889, 2871, 1681, 1664, 1653, 1617, 1476, 1467, 1455, 1397, 1389, 1376, 1362, 1227, 1187, 762, 755, 702.

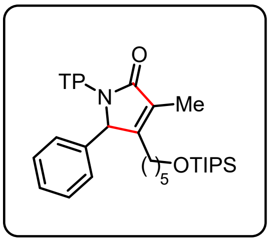

3-Methyl-5-phenyl-4-(5-((triisopropylsilyl)oxy)pentyl)-1-(2,4,4-trimethylpentan-2-yl)-1*H*-pyrrol-2(5*H*)-one (4j)

Colourless oil (46.4 mg, 44% yield). Rf 0.24 (SiO_2, hexane/EtOAc = 40/1). **^{1}H NMR** (400 MHz, $CDCl_3$) δ 7.39 – 7.18 (m, 4H), 7.17 – 6.85 (m, 1H), 4.81 (s, 1H), 3.66 (t, J = 6.6 Hz, 2H), 2.80 (d, J = 14.7 Hz, 1H), 2.35 – 2.12 (m, 2H), 1.63 – 1.47 (m, 8H), 1.43 (s, 3H), 1.41 – 1.30 (m, 2H), 1.25 (d, J = 14.8 Hz, 1H), 1.16 (s, 3H), 1.11 – 1.01 (m, 20H), 0.92 (s, 9H). **^{13}C NMR** (100 MHz, $CDCl_3$) δ 173.7, 149.9, 139.9, 132.9, 128.8, 127.8, 125.2, 69.1, 63.6, 59.0, 50.4, 33.0, 31.6, 31.5, 30.7, 28.5, 28.3, 26.0, 23.7, 18.2, 12.1, 12.0. **HRMS (ESI)** *m/z:* $[M+H]^+$ Calcd. for $C_{33}H_{58}NO_2Si$ 528.4231; Found 528.4226. **IR** (neat, cm^{-1}): 2942, 2892, 2865, 1682, 1679, 1674, 1463, 1455, 1390, 1365, 1334, 1259, 1248, 1227, 1172, 1154, 1105, 1073, 1029, 1013, 996, 882, 791, 782, 762, 701, 680, 658, 637.

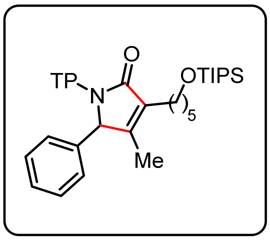

4-Methyl-5-phenyl-3-(5-((triisopropylsilyl)oxy)pentyl)-1-(2,4,4-trimethylpentan-2-yl)-1*H*-pyrrol-2(5*H*)-one (4j′)

Colourless oil (31.6 mg, 30% yield). Rf 0.23 (SiO_2, hexane/EtOAc = 40/1). **^{1}H NMR** (400 MHz, $CDCl_3$) δ 7.37 – 7.21 (m, 4H), 7.13 – 6.89 (m, 1H), 4.91 (s, 1H), 3.61 (t, J = 6.5 Hz, 2H), 2.80 (d, J = 14.8 Hz, 1H), 2.21 – 2.08 (m, 1H), 1.79 (s, 3H), 1.75 – 1.65 (m, 1H), 1.52 – 1.35 (m, 7H), 1.31 – 1.19 (m, 4H), 1.15 (s, 3H), 1.11 – 1.01 (m, 20H), 0.91 (s, 9H). **^{13}C NMR** (100 MHz, $CDCl_3$) δ 174.2, 154.0, 139.6, 128.8, 128.4, 127.8, 125.4, 67.6, 63.3, 59.2, 50.3, 32.8, 31.6, 31.4, 30.6, 28.5, 28.2, 26.3, 25.8, 18.2, 12.1, 8.8. **HRMS (ESI)** *m/z:* $[M+H]^+$ Calcd. for $C_{33}H_{58}NO_2Si$ 528.4231; Found 528.4226. **IR** (neat, cm^{-1}): 3306, 3062, 3029, 2941, 2891, 2864, 1726, 1682, 1678, 1674, 1667, 1662, 1659, 1655, 1651, 1602, 1495, 1463, 1455, 1389, 1373, 1364, 1290, 1247, 1226, 1173, 1107, 1073, 1029, 1012, 996, 986, 919, 882, 801, 785, 757, 719, 703.9019, 680, 657, 640.

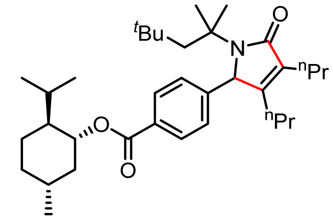

(1*R*,2*S*,5*R*)-2-isopropyl-5-methylcyclohexyl 4-(5-oxo-3,4-dipropyl-1-(2,4,4-trimethylpentan-2-yl)-2,5-dihydro-1*H*-pyrrol-2-yl)benzoate (4k)

White solid (87.1 mg, 81% yield). m.p. 81-82 °C. Rf 0.17 (SiO_2, hexane/EtOAc = 40/1). **^{1}H NMR** (400 MHz, $CDCl_3$) δ 7.97 (d, J = 20.4 Hz, 2H), 7.34 (s, 1H), 7.16 – 7.00 (m, 1H), 4.99 – 4.96 (m, 1H), 4.91 (s, 1H), 2.83 (d, J = 14.8 Hz, 1H), 2.20 (t, J = 7.7 Hz, 2H), 2.16 – 2.07 (m, 2H), 2.02 – 1.90 (m, 1H), 1.77 – 1.68 (m, 3H), 1.62 – 1.44 (m, 6H), 1.41 (d, J = 2.7 Hz, 3H), 1.30 (d, J = 19.8 Hz, 2H), 1.26 – 1.22 (m, 1H), 1.19 (d, J = 14.8 Hz, 1H), 1.15 – 1.08 (m, 4H), 0.93 (d, J = 2.9 Hz, 4H), 0.90 (s, 13H), 0.85 (t, J = 7.3 Hz, 4H), 0.79 (dd, J = 7.0, 2.2 Hz, 3H). **^{13}C NMR** (100 MHz, $CDCl_3$) δ 166.0, 163.1, 144.1, 129.9, 129.6, 126.9, 77.5, 77.2, 76.8, 74.9, 59.7, 53.4, 47.4, 44.8, 41.1, 34.5, 31.9, 31.6, 30.4, 30.4, 26.6, 23.8, 22.2, 20.9, 16.7. **HRMS (ESI)** *m/z:* $[M+Na]^+$ Calcd. for $C_{35}H_{55}NO_3$ 560.4074; Found 560.4080. 2957.834. **IR** (neat, cm^{-1}): 2933, 2871, 1714, 1681, 1609,

1464, 1456, 1415, 1387, 1377, 1365, 1351, 1326, 1307, 1285, 1274, 1222, 1174, 1114, 1101, 1082, 1038, 1018, 982, 962, 917, 768, 733, 709.

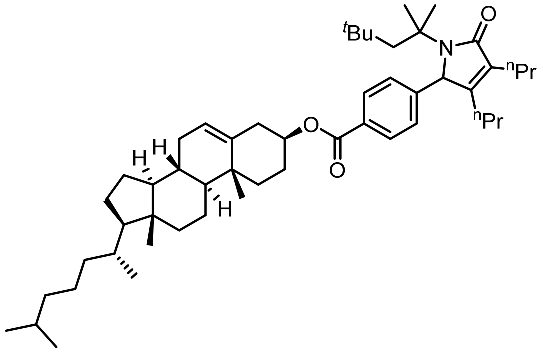

(3*S*,8*S*,9*S*,10*R*,13*R*,14*S*,17*R*)-10,13-Dimethyl-17-((*R*)-6-methylheptan-2-yl)-2,3,4,7,8,9,10,11,12,13,14,15,16,17-tetradecahydro-1*H*-cyclopenta[*a*]phenanthren-3-yl 4-(5-oxo-3,4-dipropyl-1-(2,4,4-trimethylpentan-2-yl)-2,5-dihydro-1*H*-pyrrol-2-yl)benzoate (4l)

Brown solid (116.7 mg, 76% yield). m.p. 134-135 °C. Rf 0.18 (SiO_2, hexane/EtOAc = 40/1). **^{1}H NMR** (400 MHz, $CDCl_3$) 7.98 (d, J = 18.4 Hz, 2H), 7.41 – 7.28 (m, 1H), 7.17 – 7.00 (m, 1H), 5.41 (d, J = 5.0 Hz, 1H), 4.97 (s, 1H), 4.84 (dtd, J = 12.2, 8.3, 4.5 Hz, 1H), 2.82 (d, J = 14.8 Hz, 1H), 2.50 – 2.41 (m, 2H), 2.26 – 2.17 (m, 2H), 2.16 – 2.09 (m, 1H), 2.04 – 1.94 (m, 3H), 1.91 (dt, J = 13.5, 3.5 Hz, 1H), 1.86 – 1.65 (m, 3H), 1.63 – 1.43 (m, 10H), 1.40 (s, 4H), 1.33 (s, 3H), 1.27 (s, 2H), 1.25 (s, 2H), 1.20 (d, J = 5.7 Hz, 2H), 1.17 (d, J = 4.3 Hz, 2H), 1.11 (d, J = 4.3 Hz, 5H), 1.05 (s, 3H), 1.03 – 0.95 (m, 3H), 0.92 (s, 3H), 0.90 (s, 12H), 0.87 – 0.81 (m, 9H), 0.68 (s, 3H). **^{13}C NMR** (100 MHz, $CDCl_3$) δ 173.7, 165.7, 153.5, 145.2, 139.7, 133.8, 130.5, 130.2, 123.0, 77.5, 77.2, 76.8, 74.9, 66.8, 59.2, 56.8, 56.3, 50.2, 50.2, 42.4, 39.9, 39.6, 38.3, 37.2, 36.8, 36.3, 35.9, 32.1, 32.0, 31.6, 31.4, 30.8, 28.4, 28.3, 28.1, 28.1, 28.0, 25.8, 24.4, 23.9, 22.9, 22.7, 22.2, 22.1, 21.2, 19.5, 18.8, 14.2, 14.1, 12.0. **HRMS (ESI)** *m/z:* $[M+H]^+$ Calcd. for $C_{52}H_{81}NO_3$ 768.6289; Found 768.6293. **IR** (neat, cm^{-1}): 2933, 1713, 1659, 1464, 1381, 1273, 1116, 771.

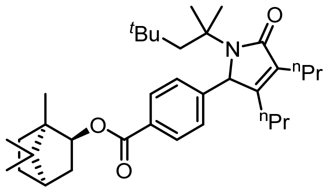

(1*R*,2*S*,4*R*)-1,7,7-Trimethylbicyclo[2.2.1]heptan-2-yl 4-(5-oxo-3,4-dipropyl-1-(2,4,4-trimethylpentan-2-yl)-2,5-dihydro-1*H*-pyrrol-2-yl)benzoate (4m)

White solid (86.8 mg, 81% yield). m.p. 93-94 °C. Rf 0.17 (SiO_2, hexane/EtOAc = 40/1). **^{1}H NMR** (400 MHz, $CDCl_3$) δ 8.12 – 7.89 (m, 2H), 7.43 – 7.29 (m, 1H), 7.17 – 7.02 (m, 1H), 5.10 (dd, J = 9.9, 3.1 Hz, 1H), 4.97 (s, 1H), 2.82 (d, J = 14.8 Hz, 1H), 2.46 (td, J = 9.9, 4.6 Hz, 1H), 2.20 (t, J = 7.6 Hz, 2H), 2.13 (dt, J = 12.6, 3.2 Hz, 1H), 1.80 (td, J = 8.1, 4.0 Hz, 1H), 1.73 (t, J = 4.5 Hz, 1H), 1.63 – 1.44 (m, 5H), 1.41 (s, 3H), 1.38 – 1.16 (m, 7H), 1.13 (s, 3H), 0.96 (s, 3H), 0.92 (s, 1H), 0.90 (s, 15H), 0.85 (t, J = 7.3 Hz, 4H). **^{13}C NMR** (100 MHz, $CDCl_3$) 173.7, 166.5, 153.5, 145.3, 133.8, 130.6, 130.1, 80.8, 77.5, 77.2, 76.8, 66.8, 59.2, 50.2, 49.2, 48.0, 45.1, 37.0, 37.0, 31.6, 31.4, 30.8, 28.3, 28.2, 28.1, 27.5, 25.8, 22.2, 22.1, 19.8, 19.0, 14.2, 14.1, 13.8. **HRMS (ESI)** *m/z:* $[M+Na]^+$ Calcd. for $C_{35}H_{53}NO_3$ 558.3918; Found 558.3919. **IR** (neat, cm^{-1}): 2956, 2871, 1715, 1678, 1674,

1666, 1658, 1619, 1609, 1465, 1454, 1416, 1379, 1364, 1326, 1307, 1300, 1281, 1273, 1237, 1223, 1175, 1115, 1101, 1082, 1044, 1032, 1019, 988, 978, 940, 917, 888, 878, 862, 844, 830, 813, 806, 769, 732, 710, 646.

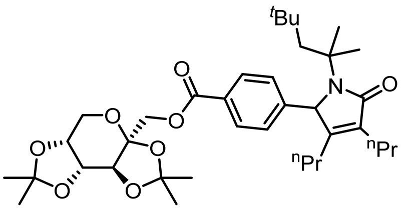

((3*aS*,5*aR*,8*aR*,8*bS*)-2,2,7,7-Tetramethyltetrahydro-3a*H*-bis([1,3]dioxolo)[4,5-b:4',5'-d]pyran-3a-yl)methyl 4-(5-oxo-3,4-dipropyl-1-(2,4,4-trimethylpentan-2-yl)-2,5-dihydro-1*H*-pyrrol-2-yl) benzoate (4n)

White solid (44.9 mg, 35% yield). m.p. 67-69 °C. Rf 0.12 (SiO_2, hexane/EtOAc = 40/1). **^{1}H NMR** (400 MHz, $CDCl_3$) δ8.03 (d, *J* = 17.2 Hz, 2H), 7.44 – 7.28 (m, 1H), 7.16 – 7.01 (m, 1H), 4.96 (s, 1H), 4.68 – 4.56 (m, 2H), 4.44 (s, 1H), 4.33 (d, *J* = 11.8 Hz, 1H), 4.25 (d, *J* = 7.9 Hz, 1H), 3.95 (dd, *J* = 13.1, 1.9 Hz, 1H), 3.80 (d, *J* = 13.0 Hz, 1H), 2.80 (dd, *J* = 14.9, 1.8 Hz, 1H), 2.28 – 2.07 (m, 3H), 1.67 (s, 2H), 1.59 (dd, *J* = 8.9, 5.4 Hz, 1H), 1.55 (s, 4H), 1.53 – 1.46 (m, 2H), 1.43 (d, *J* = 2.2 Hz, 3H), 1.41 – 1.35 (m, 6H), 1.33 (s, 3H), 1.29 – 1.16 (m, 3H), 1.11 (s, 3H), 0.91 (d, *J* = 7.3 Hz, 3H), 0.89 (s, 7H), 0.83 (t, *J* = 7.3 Hz, 3H). **^{13}C NMR** (100 MHz, $CDCl_3$) δ 173.7, 165.7, 153.4, 145.7, 133.9, 130.4, 129.6, 129.6, 109.3, 109.0, 101.8, 77.5, 77.2, 76.8, 70.9, 70.8, 70.2, 66.9, 65.9, 65.9, 61.5, 59.2, 50.2, 31.6, 31.4, 30.8, 30.7, 28.3, 28.1, 26.6, 26.0, 26.0, 25.9, 25.6, 25.6, 24.1, 22.2, 22.1, 14.2, 14.1. **HRMS (ESI)** *m/z:* $[M+Na]^+$ Calcd. for $C_{38}H_{57}NO_7$ 664.3820; Found 664.3900. **IR** (neat, cm^{-1}): 2930, 1725, 1678, 1454, 1374, 1250, 1206, 1164, 1101, 1070, 1019, 889, 758, 710, 1285, 521.

Supplementary Note 4

Crystal Structure Information of 3k

A solution of pure **3k** (50 mg) in DCM (2 mL) in a 5 mL oven-dried glass sample bottle, was sealed with perforated paper. After the solvent was slowly evaporated at room temperature, crystals were obtained. Single crystal X-ray diffraction data were collected on Rigaku Saturn70 diffractometer. The crystal structure has been deposited at the Cambridge Crystallographic Data Centre (CCDC)：Deposition Number 2089375

Compound Name: 5-(4-Morpholinophenyl)-3,4-dipropyl-1-(2,4,4-trimethylpentan-2-yl)-1H-pyrrol-2(5*H*)-one

Chemical Formula: $C_{28}H_{44}N_2O_2$

Data Block Name: data_r20200519c

Unit Cell Parameters: a 9.7732(4) b 14.8171(7) c 18.3308(6) P212121

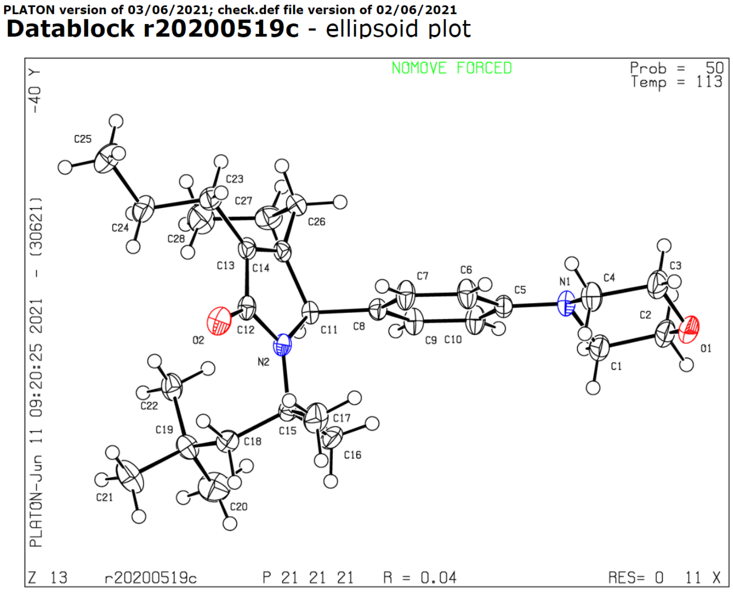

Supplementary Figure 1. Crystal structure information of compound **3k**.

Supplementary Note 5

Gram-scale reaction and product transformation

Gram-scale reaction

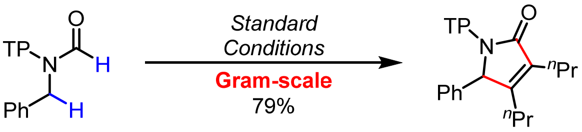

1a, 5.0 mmol **3a**, 1.41g

To a 100 mL oven-dried tube was added ^{t}Bu-DAPO (109.1 mg, 10 mol%), $Ni(cod)_2$ (137.5 mg, 10 mol%), dry degassed toluene (25.0 mL), **1a** (1.236 g, 5.0 mmol), alkyne (1.651 g, 15.0 mmol) and $AlEt_3$ (1.0 M in toluene, 2.0 mL, 40 mol%) sequentially in an N_2-filled glove-box. The tube was sealed and removed out of the glove-box. After heated at 120 °C in a dry block heater for 8 h, the mixture was cooled to r.t., filtered through a short plug of silica gel (DCM as the eluent) and concentrated in vacuo to afford a crude product. Further purification by flash column chromatography on silica gel (eluting with EtOAc/*n*-hexane) gave pure product **3a** as a pale yellow oil (79% yield, 1.41 g).

Removal of protecting group

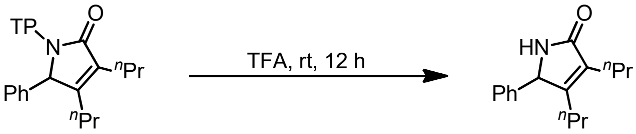

3a **5**, 98%

To a round-bottomed flask was added TFA (3.0 mL) and **3a** (142.1 mg, 0.4 mmol) at 0 ºC under

aIR atmosphere. The reaction mixture was then stirred at room temperature for additional 12 h, after which time the solvent was removed under reduced pressure. The mixture was diluted with saturated $NaHCO_3$ (aq), extracted with EtOAc (10 mL x 3). The combined organic layers were washed by brine, dried over $MgSO_4$, filtered and concentrated in vacuo. The crude product was subjected to silica gel chromatography (Hexane/EtOAc) to afford the desired product as a white solid (95.3 mg). m.p. 120-122 °C. **^{1}H NMR** (400 MHz, $CDCl_3$) δ 7.38 – 7.30 (m, 3H), 7.19 – 7.15 (m, 2H), 6.35 (s, 1H), 4.94 (s, 1H), 2.34 – 2.18 (m, 3H), 1.93 – 1.79 (m, 1H), 1.64 – 1.53 (m, 2H), 1.50 – 1.40 (m, 1H), 1.36 – 1.27 (m, 1H), 0.95 (t, J = 7.3 Hz, 3H), 0.87 (t, J = 7.3 Hz, 3H). **^{13}C NMR** (100 MHz, $CDCl_3$) δ 175.3, 157.5, 137.6, 132.1, 129.0, 128.4, 127.2, 62.6, 28.7, 25.6, 22.2, 22.2, 14.2, 14.1. **HRMS (ESI)** *m/z:* $[M+H]^+$ Calcd. for $C_{16}H_{22}NO$ 244.1696; Found 244.1696.

C-N Formation of γ-lactam 5 and 1-iodo-4-methoxybenzene

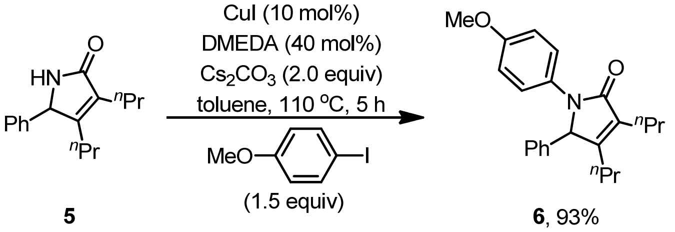

To a 15 mL oven-dried tube was added CuI (11.8 mg, 0.062 mmol, 0.1 equiv), Cs_2CO_3 (404.0 mg, 1.24 mmol, 2.0 equiv), compound **5** (150.0 mg, 0.62 mmol, 1.0 equiv), 1-iodo-4-methoxybenzene (217.6 mg, 0.93 mmol, 1.5 equiv), dry degassed toluene (2.0 mL) and DMEDA (21.9 mg, 0.25 mmol, 0.4 equiv) sequentially in an N_2-filled glove-box. The tube was then sealed and removed out of the glove-box. After heated at 110 °C in a preheated dry block heater for additional 5 h, the mixture was cooled to r.t., filtered through a short plug of silica gel and concentrated in vacuo to afford the crude product, which was purified by flash column chromatography on silica gel (eluting with EtOAc/Hexane) to afford a pure product as a colorless oil (201.4 mg). **^{1}H NMR** (400 MHz, $CDCl_3$) δ 7.41 – 7.35 (m, 2H), 7.29 – 7.19 (m, 3H), 7.16 – 7.10 (m, 2H), 6.77 – 6.71 (m, 2H), 5.35 (s, 1H), 3.67 (s, 3H), 2.40 – 2.26 (m, 3H), 1.93 – 1.84 (m, 1H), 1.68 – 1.58 (m, 2H), 1.56 – 1.46 (m, 1H), 1.40 – 1.28 (m, 1H), 0.97 (t, J = 7.4 Hz, 3H), 0.89 (t, J = 7.3 Hz, 3H). **^{13}C NMR** (100 MHz, $CDCl_3$) δ 171.4, 156.2, 154.7, 136.6, 132.4, 131.1, 129.0, 128.3, 127.2, 123.0, 113.9, 67.4, 55.3, 28.5, 25.9, 22.1, 22.1, 14.2, 14.2. **HRMS (ESI)** *m/z:* $[M+Na]^+$ Calcd. for $C_{23}H_{27}NO_2Na$ 372.1934; Found 372.1937.

Synthesis of *N*-Boc-substituted γ-lactam

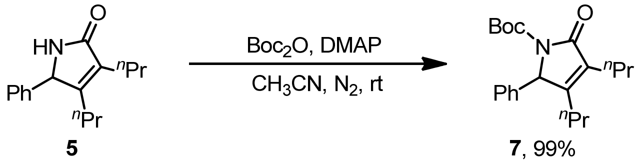

To a solution of compound **5a** (97.3 mg, 0.4 mmol, 1.0 equiv) in dry CH_3CN (5.0 mL) were added Boc_2O (130.8 mg, 0.6 mmol, 1.5 equiv) and DMAP (4.9 mg, 0.04 mmol, 0.1 equiv) under N_2 at room temperature. After stirring for 6 hours, the reaction mixture was concentrated under reduced pressure. The residue was purified by column chromatography eluting with (EtOAc/Hexane) on silica gel to afford *N*-Boc-substituted γ-lactam **7** as a colorless oil (136.0 mg). **^{1}H NMR** (400 MHz,

$CDCl_3$) δ 7.36 – 7.27 (m, 3H), 7.19 – 7.13 (m, 2H), 5.25 (s, 1H), 2.35 – 2.22 (m, 3H), 1.87 – 1.77 (m, 1H), 1.63 – 1.45 (m, 3H), 1.38 – 1.30 (m, 1H), 1.28 (s, 9H), 0.94 (t, J = 7.4 Hz, 3H), 0.88 (t, J = 7.4 Hz, 3H). **^{13}C NMR** (100 MHz, $CDCl_3$) δ 170.7, 157.6, 149.1, 137.3, 132.0, 128.7, 128.3, 126.9, 82.4, 65.6, 28.6, 27.9, 25.5, 21.9, 21.6, 14.1, 14.1. **HRMS (ESI)** *m/z:* $[M+Na]^+$ Calcd. for $C_{21}H_{29}NO_3Na$ 366.2040; Found 366.2044.

Oxidation of γ-lactam 5 and related transformation

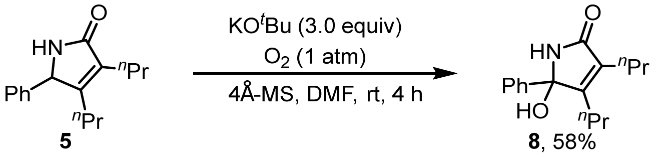

To a 25 mL three-necked round bottom flask equipped with a magnetic stir bar was added freshly activated 4Å-molecular sieves (145.8 mg), compound **5** (48.6 mg, 0.2 mmol, 1.0 equiv) and *t*-BuOK (67.3 mg, 0.6 mmol, 3.0 equiv). The flask was then purged with O_2 and fitted with a balloon of O_2 for the duration of the reaction. Dry DMF (2.0 mL) was then added via syringe and the mixture was allowed to stir vigorously at room temperature for additional 4 hours. The mixture was transferred to a separatory funnel and diluted with EtOAc. The resulting mixture was washed with aqueous ammonium chloride to remove DMF. The organic phase was dried over anhydrous sodium sulfate, filtered and concentrated to give a crude product, which was purified by silica gel chromatography (Hexane/EtOAc) to give the desired product as a white solid (30.1 mg). m.p. 98-100 °C. **^{1}H NMR** (400 MHz, $CDCl_3$) δ 7.46 – 7.40 (m, 2H), 7.34 – 7.27 (m, 3H), 6.60 (s, 1H), 4.12 (s, 1H), 2.19 – 2.10 (m, 3H), 2.03 – 1.94 (m, 1H), 1.54 – 1.44 (m, 2H), 1.38 – 1.28 (m, 1H), 1.16 – 1.04 (m, 1H), 0.91 (t, J = 7.4 Hz, 3H), 0.78 (t, J = 7.3 Hz, 3H). **^{13}C NMR** (100 MHz, $CDCl_3$) δ 174.3, 159.4, 138.9, 131.2, 128.6, 128.4, 125.8, 89.3, 27.7, 25.5, 22.0, 21.8, 14.7, 14.3. **HRMS (ESI)** *m/z:* $[M+Na]^+$ Calcd. for $C_{16}H_{21}NO_2Na$ 282.1465; Found 282.1468.

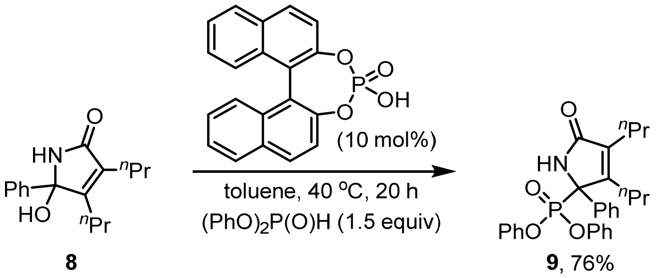

To a dry Schlenk tube were added compound **8** (51.8 mg, 0.2 mmol, 1.0 equiv), diphenyl phosphite (70.0 mg, 0.3 mmol, 1.5 equiv), 1,1'-Binaphthyl-2,2'-diyl hydrogenphosphate (7.0 mg, 0.02 mmol, 0.1 equiv) and dry toluene (1.0 mL) under nitrogen atmosphere. After the mixture was stirred at 40 °C for 20 hours, all volatiles were evaporated under reduced pressure, and the residue was purified by flash chromatography (Hexane/EtOAc) to afford the desired product as a white solid (72.2 mg). m.p. 124-126 °C. **^{1}H NMR** (400 MHz, $CDCl_3$) δ 7.91 – 7.83 (m, 2H), 7.59 – 7.47 (m, 1H), 7.39 – 7.31 (m, 3H), 7.26 – 7.16 (m, 4H), 7.15 – 7.05 (m, 4H), 7.04 – 6.98 (m, 2H), 2.58 – 2.48 (m, 1H), 2.37 – 2.25 (m, 1H), 2.23 – 2.10 (m, 2H), 1.56 – 1.41 (m, 2H), 1.23 – 1.11 (m, 1H), 0.89 (t, J = 7.4 Hz, 3H), 0.82 – 0.73 (m, 1H), 0.70 (t, J = 6.8 Hz, 3H). **^{13}C NMR** (100 MHz, $CDCl_3$) δ 174.8, 155.1 (d, J = 4.9 Hz), 151.0 (d, J = 9.2 Hz), 150.2 (d, J = 10.4 Hz), 135.6 (d, J = 7.6 Hz), 134.3 (d, J = 2.2 Hz), 129.7, 129.0, 128.8, 126.7 (d, J = 6.7 Hz), 125.5, 125.3, 120.7 (d, J = 4.1 Hz), 120.2 (d, J = 4.4 Hz), 69.5 (d, J = 152.7 Hz), 29.2, 26.5, 22.3, 21.5 (d, J = 2.5 Hz), 14.5, 14.4. **^{31}P NMR** (162 MHz, $CDCl_3$) δ 10.63. **HRMS (ESI)** *m/z:* $[M+Na]^+$ Calcd. for $C_{28}H_{30}NO_4PNa$ 498.1805; Found

498.1810.

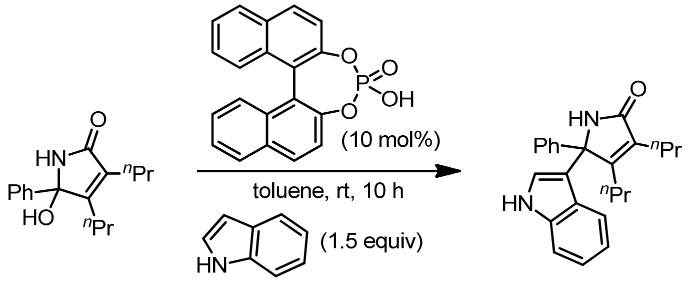

8 **10**, 97%

To a dry Schlenk tube were added compound **8** (51.8 mg, 0.2 mmol, 1.0 equiv), 1H-indole (35.2 mg, 0.3 mmol, 1.5 equiv), 1,1'-Binaphthyl-2,2'-diyl hydrogenphosphate (7.0 mg, 0.02 mmol, 0.1 equiv) and dry toluene (1.0 mL) at room temperature under nitrogen atmosphere. After the mixture was stirred for 10 hours, all volatiles were evaporated under reduced pressure, and the residue was purified by flash chromatography (Hexane/EtOAc) to afford the desired product as a white solid (69.5 mg). m.p. 230-232 °C. **^{1}H NMR** (400 MHz, $CDCl_3$) δ 8.67 (s, 1H), 7.39 – 7.35 (m, 2H), 7.30 – 7.28 (m, 2H), 7.18 – 7.11 (m, 1H), 7.01 – 6.90 (m, 3H), 6.40 (s, 1H), 2.46 – 2.24 (m, 4H), 1.72 – 1.57 (m, 2H), 1.01 – 0.89 (m, 4H), 0.66 – 0.54 (m, 4H). **^{13}C NMR** (100 MHz, $CDCl_3$) δ 174.6, 160.7, 140.1, 136.9, 132.0, 128.6, 128.0, 127.3, 126.0, 123.0, 122.5, 120.4, 120.0, 116.1, 111.7, 68.8, 30.0, 26.3, 22.5, 21.9, 14.7, 14.4. **HRMS (ESI)** *m/z:* $[M+Na]^+$ Calcd. for $C_{24}H_{26}N_2ONa$ 381.1937; Found 381.1940.

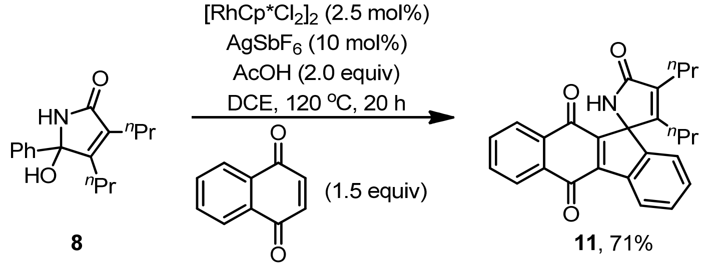

8 **11**, 71%

To a 15 mL oven-dried tube were added $[RhCp^*Cl_2]_2$ (3.1 mg, 0.005 mmol, 0.025 equiv), silver hexafluoroantimonate (6.8 mg, 0.02 mmol, 0.1 equiv), compound **8** (51.8 mg, 0.2 mmol, 1.0 equiv), 1,4-naphthoquinone (47.4 mg, 0.3 mmol, 1.5 equiv), AcOH (24.0 mg, 0.4 mmol, 2.0 equiv) and dry degassed DCE (1.0 mL) in an N_2-filled glove-box. The tube was then sealed and removed out of the glove-box. After heated at 120 °C in a preheated dry block heater for additional 20 h, the mixture was cooled to r.t., filtered through a short plug of silica gel and concentrated in vacuo to afford a crude product, which was purified by flash column chromatography on silica gel (eluting with EtOAc/Hexane) to afford a pure product as a yellow solid (56.2 mg). m.p. 230-232 °C. **^{1}H NMR** (400 MHz, $CDCl_3$) δ 8.31 (d, J = 7.2 Hz, 1H), 8.17 – 8.09 (m, 1H), 8.08 – 7.98 (m, 1H), 7.78 – 7.68 (m, 2H), 7.46 – 7.38 (m, 2H), 7.22 (d, J = 7.1 Hz, 1H), 6.18 (s, 1H), 2.45 – 2.34 (m, 2H), 1.81 – 1.73 (m, 2H), 1.72 – 1.62 (m, 2H), 1.01 (t, J = 7.3 Hz, 3H), 0.97 – 0.92 (m, 1H), 0.91 – 0.80 (m, 1H), 0.62 (t, J = 7.2 Hz, 3H). **^{13}C NMR** (100 MHz, $CDCl_3$) δ 183.4, 180.5, 175.4, 153.2, 146.5, 146.5, 145.4, 136.7, 135.4, 134.4, 133.7, 133.3, 132.9, 130.8, 130.1, 126.6, 126.6, 126.6, 123.2, 74.3, 28.2, 26.6, 22.3, 21.9, 14.5, 14.2. **HRMS (ESI)** *m/z:* $[M+Na]^+$ Calcd. for $C_{26}H_{23}NO_3Na$ 420.1570; Found 420.1575.

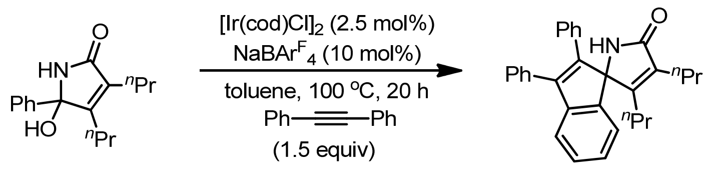

8 **12**, 74%

To a 15 mL oven-dried tube was added $[Ir(cod)Cl]_2$ (3.6 mg, 0.005 mmol, 0.025 equiv), sodium tetrakis[3,5-bis(trifluoromethyl)phenyl]borate (17.8 mg, 0.02 mmol, 0.1 equiv), compound **8** (51.8 mg, 0.2 mmol, 1.0 equiv), biphenylacetylene (53.4 mg, 0.3 mmol, 1.5 equiv) and dry degassed toluene (1.0 mL) in an N_2-filled glove-box. The tube was then sealed and removed out of the glove-box. After heated at 100 °C in a preheated dry block heater for additional 20 h, the mixture was cooled to r.t., filtered through a short plug of silica gel and concentrated in vacuo to afford the crude product, which was purified by flash column chromatography on silica gel (eluting with EtOAc/Hexane) to afford a pure product as a reddish solid (62.0 mg). m.p. 171-173 ºC. **^{1}H NMR** (400 MHz, $CDCl_3$) δ 7.40 – 7.29 (m, 7H), 7.24 – 7.18 (m, 1H), 7.17 – 7.07 (m, 6H), 5.89 (s, 1H), 2.40 – 2.25 (m, J = 13.4, 6.7 Hz, 2H), 2.02 – 1.85 (m, 2H), 1.58 – 1.44 (m, J = 13.8, 7.0 Hz, 2H), 1.18 – 0.99 (m, 2H), 0.83 (t, J = 7.3 Hz, 3H), 0.70 (t, J = 7.3 Hz, 3H). **^{13}C NMR** (100 MHz, $CDCl_3$) δ 175.3, 155.8, 144.0, 143.2, 143.1, 142.0, 134.7, 134.4, 133.8, 129.3, 128.9, 128.8, 128.8, 128.2, 128.1, 127.8, 126.8, 122.4, 121.3, 28.3, 26.4, 22.0, 21.8, 14.8, 14.0. **HRMS (ESI)** *m/z:* $[M+Na]^+$ Calcd. for $C_{30}H_{29}NONa$ 442.2141; Found 442.2145.

Supplementary Note 6

Mechanistic experiments

NMR experiments

1) Detection of PO–Al Complex

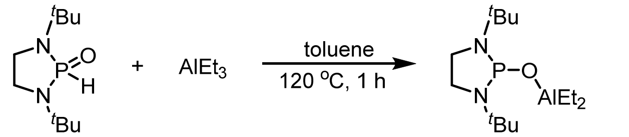

0.1 mmol

$AlEt_3$	**PO-Al complex** (^{31}P NMR, δ ppm)
0	6.90
1.0 eq	**96.91**
$Ni(cod)_2$ (1.0 eq) instead of $AlEt_3$	6.86 (*black nickel precipitation*)

To a 15 mL oven dried Schlenk tube was added ^{t}Bu-DAPO (21.8 mg, 0.1 mmol), toluene (0.5 mL) and $AlEt_3$ (100 µL, 1.0 M in hexane, 0.1 mmol) at room temperature under N_2 atmosphere. After stirred for 1h at 120 ºC, the mixture was cooled to r.t. and all volatiles were evaporated under reduced pressure. The crude sample were dissolved, diluted with C_6D_6 and transferred into the NMR tube, sealed and monitored by nuclear magnetic resonance spectrometer (^{31}P NMR, ^{1}H NMR, ^{13}C NMR). The ^{31}P NMR suggested that $Ni(cod)_2$ failed to shift the equilibrium toward to the trivalent phosphinous species and the catalyst decomposed, whereas $AlEt_3$ can promote the process to form a new phosphorus species (δ 96.91 ppm). The PO–Al–complex also was characterized by ^{1}H NMR

and ^{13}C NMR.

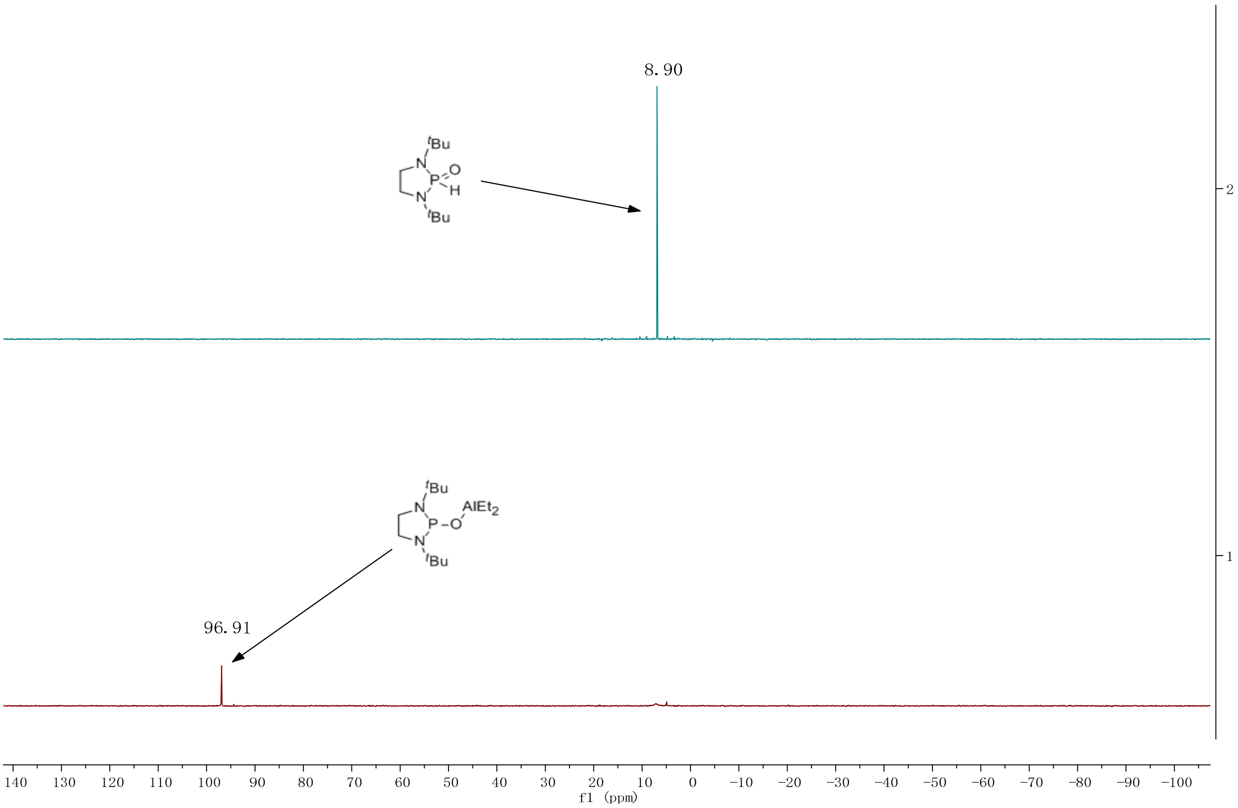

Supplementary Figure 2. ^{31}P NMR spectrum comparison between **PO** and **PO-Al** complex.

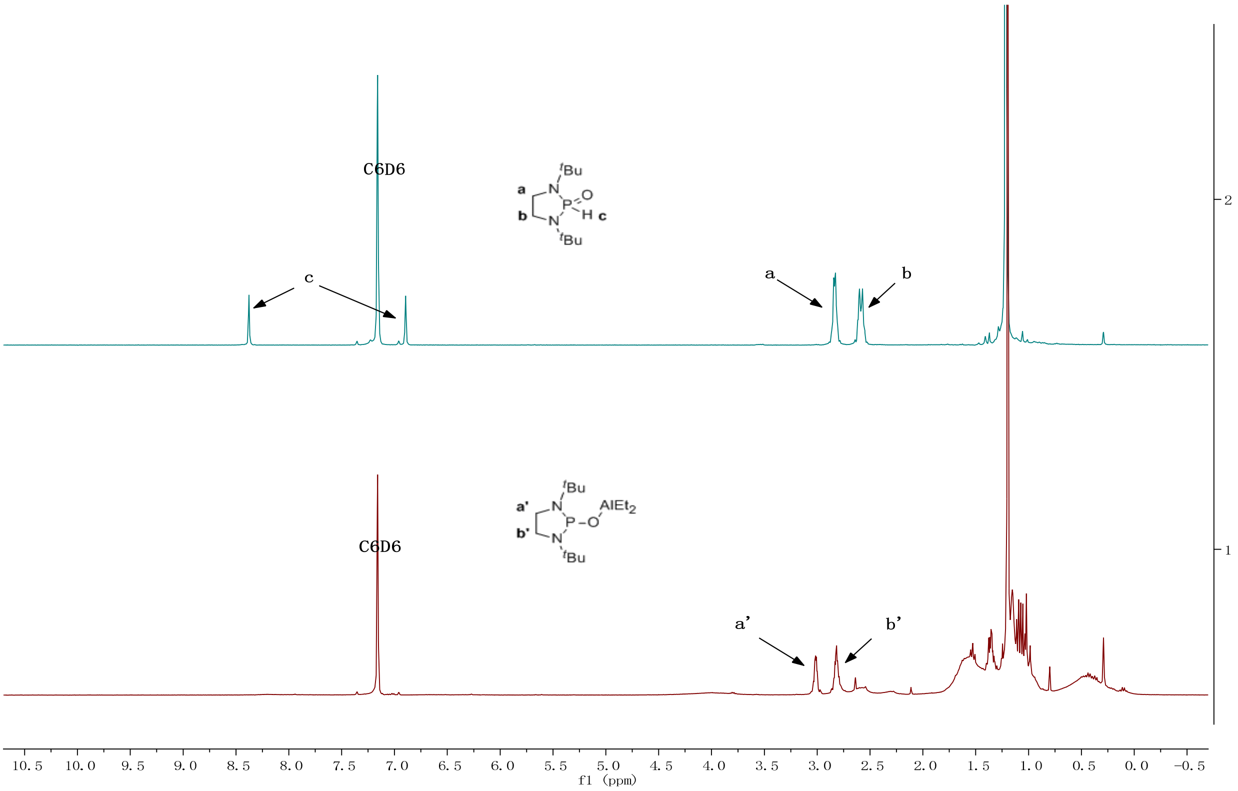

Supplementary Figure 3. ^{1}H NMR spectrum comparison between **PO** and **PO-Al** complex.

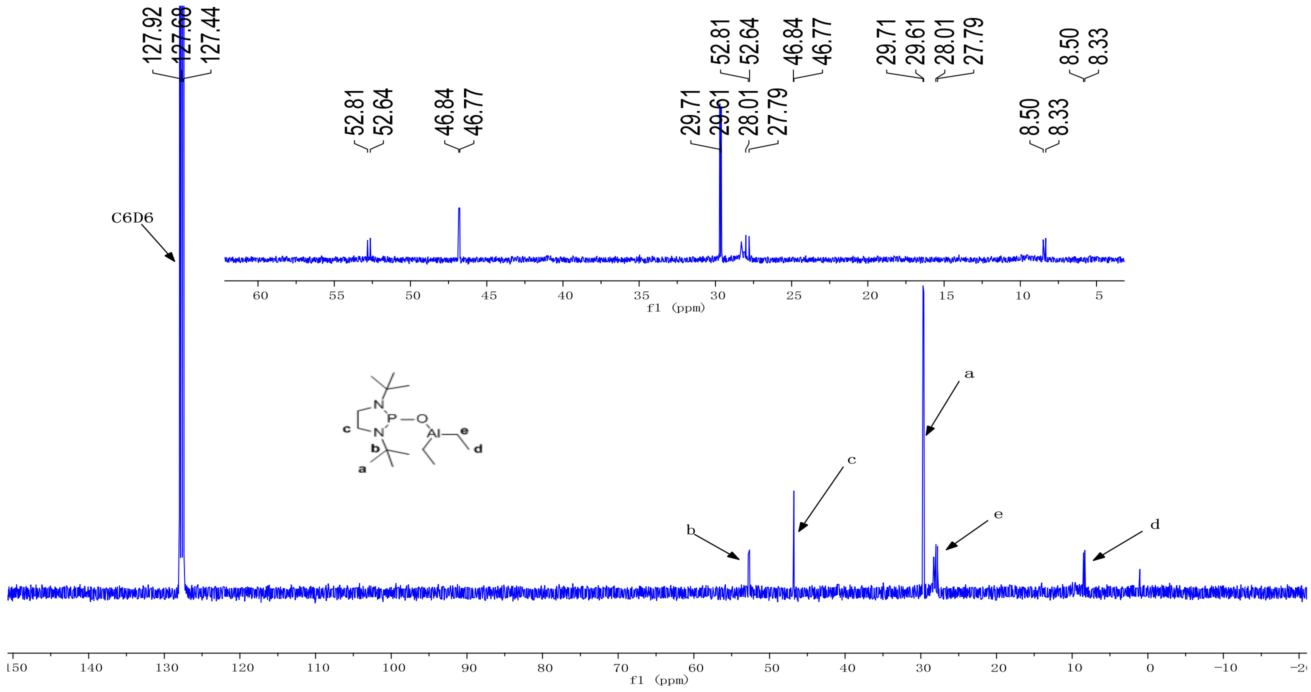

Supplementary Figure 4. ^{13}C NMR spectrum comparison between **PO** and **PO-Al** complex.

2) Detection of PO-Ni-Al Complex

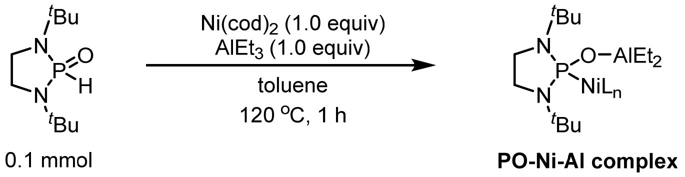

Addition sequence	^{31}P NMR (δ ppm)
$Ni(cod)_2$, 1 h, then $AlEt_3$, 1 h	96.91 (*black nickel precipitation*)
$AlEt_3$, 1 h, then $Ni(cod)_2$, 1 h	96.91 (*black nickel precipitation*)
$Ni(cod)_2$ and $AlEt_3$ for 1 h	96.13

To a 15 mL oven dried Schlenk tube was added $Ni(cod)_2$ (27.5 mg, 0.1 mmol), ^{t}Bu-DAPO (21.8 mg, 0.1 mmol), toluene (0.5 mL) and $AlEt_3$ (100 μL, 1.0 M in hexane, 0.1 mmol) at room temperature under N_2 atmosphere. After stirred for 1h at 120 °C, the mixture was cooled to r.t., all volatiles were evaporated under reduced pressure. The crude sample were dissolved, diluted with C_6D_6 and transferred into the NMR tube, sealed and monitored by NMR (^{31}P NMR, ^{1}H NMR, ^{13}C NMR). It is worth mentioning that the addition sequence of $Ni(cod)_2$ and $AlEt_3$ is pivotal for the formation of PO–Ni–Al complex. When $Ni(cod)_2$ was added first and heated for 1 h, the catalyst will decompose and only PO–Al complex (δ 96.91 ppm) was detected. After the PO–Al complex was prepared in advance, addition of $Ni(cod)_2$ also failed to generate the desired PO–Ni–Al complex, which probably was ascribed to formation of the stable the PO–Al complex dimer and P–O bond easily cleavage in the presence of $Ni(cod)_2$. However, both are simultaneously added and heated with PO ligand, the new phosphorus species (δ 96.13 ppm) was detected in a homogeneous solution.

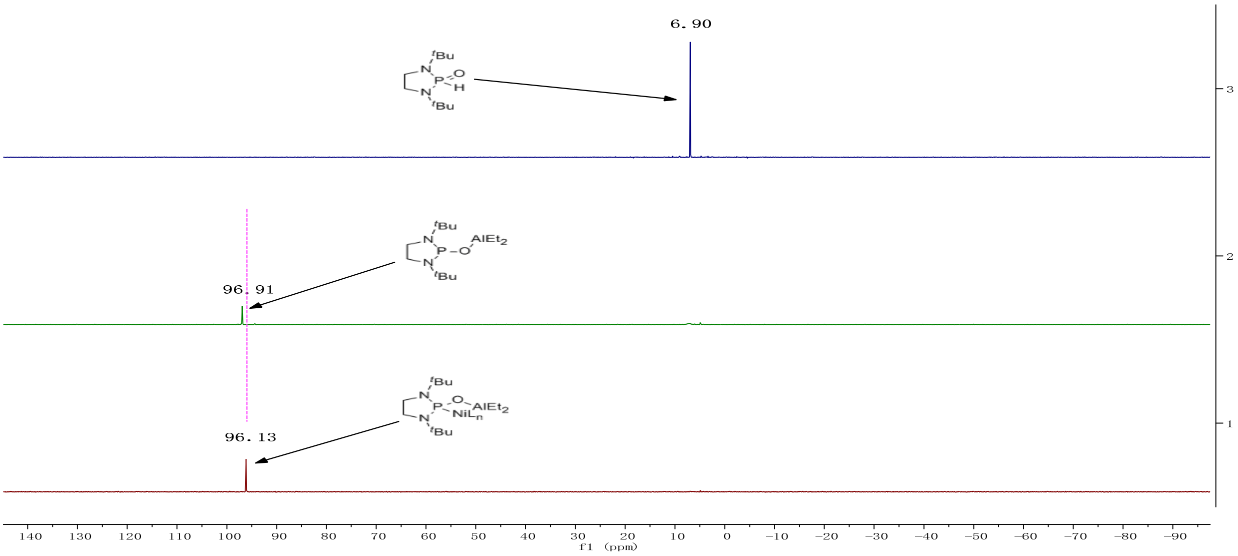

Supplementary Figure 5. ^{31}P NMR spectrum comparison between **PO** and **PO-Al** complex.

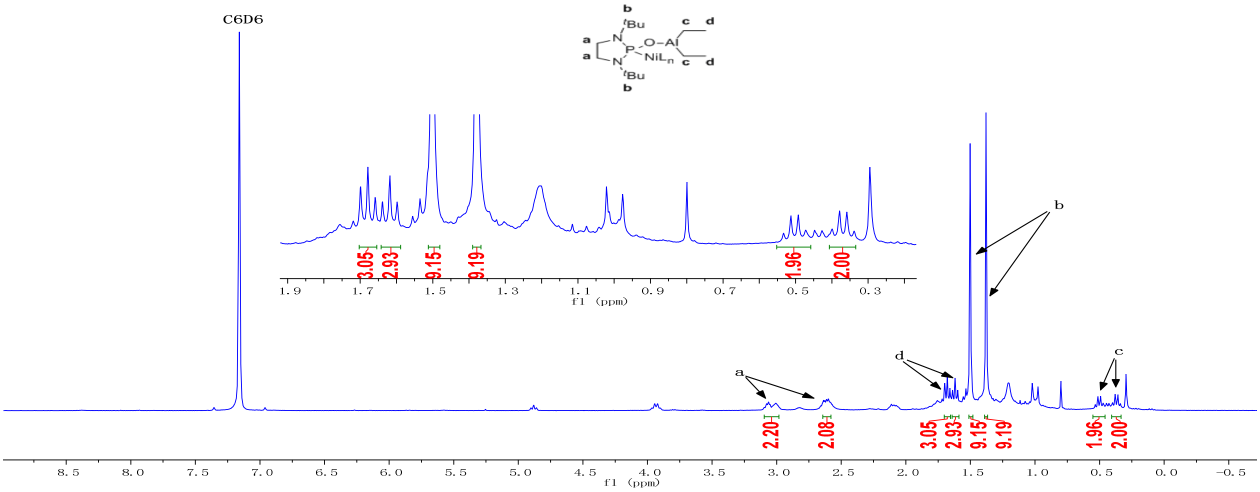

Supplementary Figure 6. ^{1}H NMR spectrum of **PO-Al** complex.

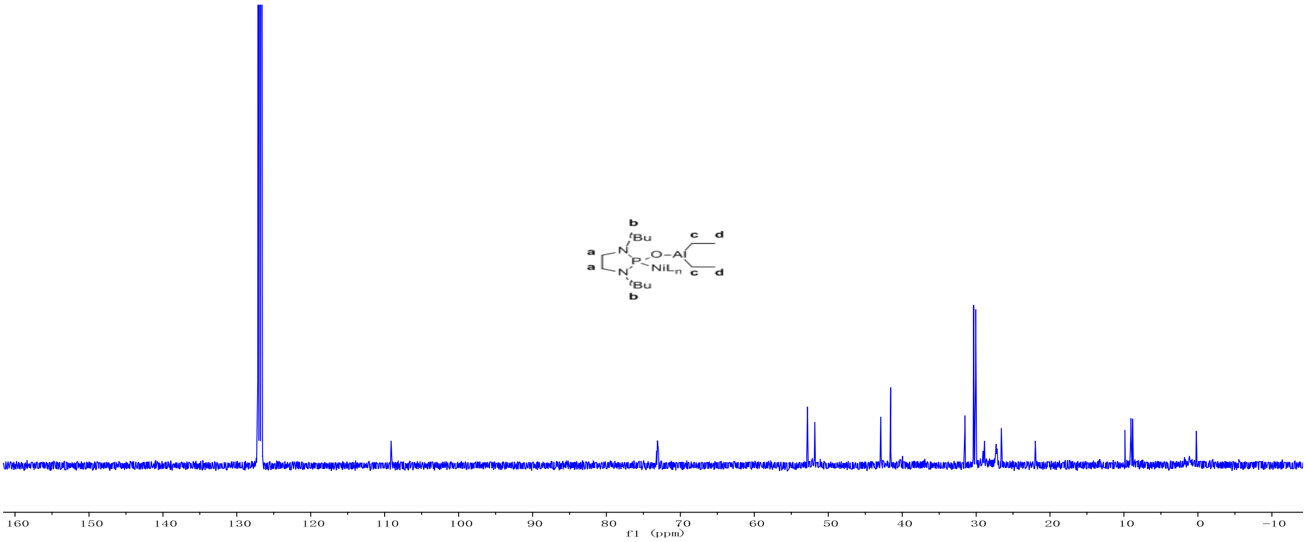

Supplementary Figure 7. ^{13}C NMR spectrum of **PO-Al** complex.

3) Reactivity of PO–Ni–Al Complex

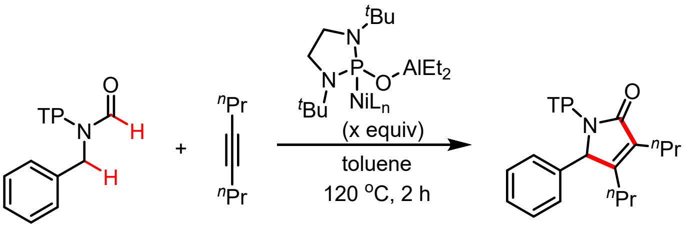

1a, 0.2 mmol **2a**, 0.6 mmol **3a**

x (equiv)	yield of **3a** (%)
0.1	6
0.1 $AlEt_3$ (30 mol%)	40
1.0	85

To a 15 mL oven dried tube was added **1a** (49.4 mg, 0.2 mmol), toluene (1.0 mL) and oct-4-yne (**2a**) (88 μL, 0.6 mmol) sequentially in an N_2-filled glove-box. The freshly prepared PO-Ni-Al-complex (0.5 mL toluene solution) was then added. The tube was sealed and removed out of the glove-box. After heated at 120 °C in a preheated dry block heater for 2 h, the mixture was cooled to r.t., quenched with 0.1 mL H_2O, filtered through a short plug of silica gel (DCM as the eluent) and concentrated in vacuo to afford a crude product. The yield was determined by ^{1}H NMR analysis of the crude sample using DMF as the internal standard.

Deuterated-substrate preparation

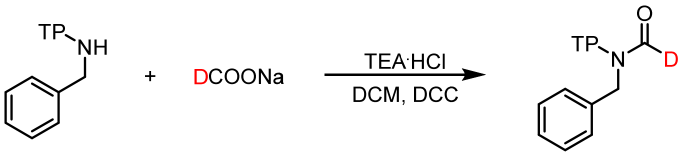

1a-*d*

According to the reported literature,[2] a mixture of *N*-benzyl-2,4,4-trimethylpentan-2-amine (439 mg, 2.0 mmol), sodium formate-*d* (218 mg, 1.5 equiv) and Et_3N·HCl (435 mg, 1.5 equiv) was stirred in DCM (3 mL) at room temperature for 30 min. Then DCC (651 mg, 1.5 equiv) was added. The reaction mixture was stirred for 24 h at room temperature. The residue was filtered through a pad of celite and washed with EtOAc (30 mL). The filtrate was concentrated and purified by column chromatography on silica gel to afford the desired product as a white solid (260 mg, 52% yield, > 99% D). **^{1}H NMR** (400 MHz, $CDCl_3$) δ 7.33 – 7.26 (m, 2H), 7.25 – 7.18 (m, 3H), 4.60 (s, 2H), 1.66 (s, 2H), 1.38 (s, 6H), 0.99 (s, 9H).

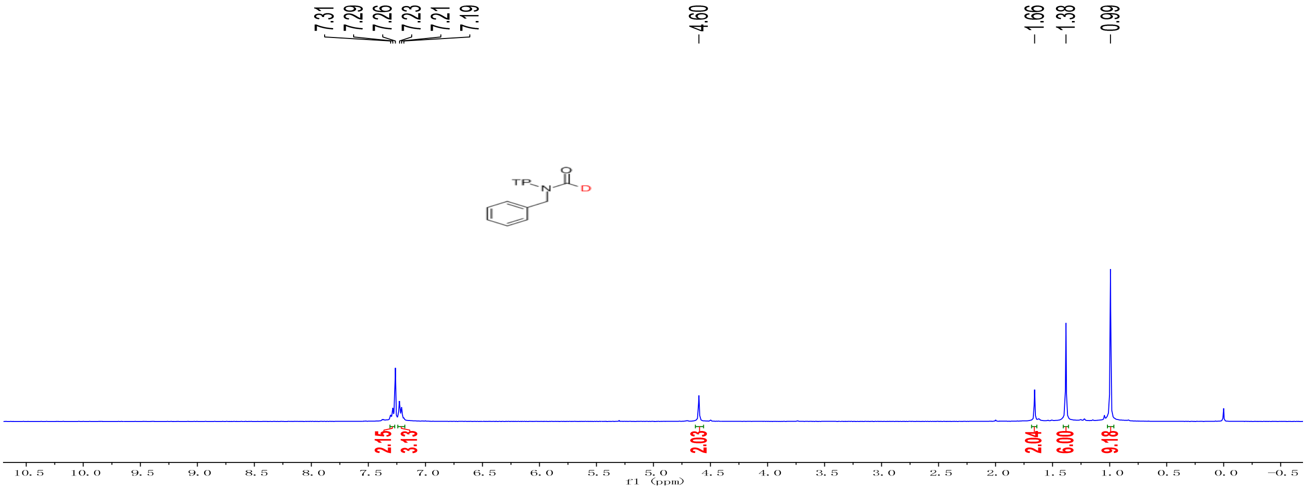

Supplementary Figure 8. ^{1}H NMR of compound **1a-*d***.

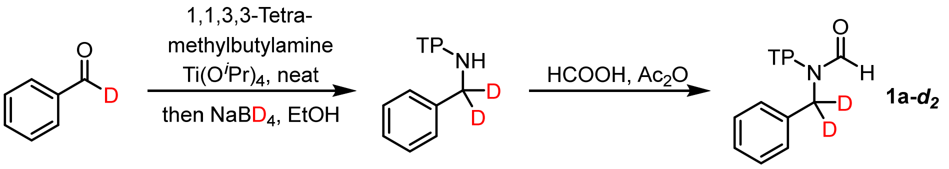

A solution of benzaldehyde-*d* (5.0 mmol, 1.0 equiv), amine (1.1 equiv) and Ti(O^{i}Pr)$_4$ (2.0 equiv) was stirred neat for 8 hours at 80 °C under nitrogen. After cooled to 0 °C, the mixture was diluted with absolute ethanol (dilute to 0.25 M), and then sodium borohydride-d_4 (3.0 equiv) was added portion wise over 10 mins. The resulting mixture was stirred for an additional 4 hours at 80 °C, cooled to room temperature and poured into aqueous sodium hydroxide (2.0 M). The resulting white suspension was filtered and washed with ethyl acetate. The filtrate was extracted with ethyl acetate and the combined organic extracts were washed with brine, dried over magnesium sulphate and concentrated in vacuo. The crude product was directly used for next step without further purification.

A mixture of formic acid (50 mmol, 1.9 mL), acetic anhydride (50 mmol, 4.7 mL) was stirred for 30 mins at room temperature. To the solution was added crude amine at 0 °C, and the resulting mixture was heated to 80 °C and stirred for an additional 8 hours. After cooling to room temperature, the solution was concentrated under reduced pressure and purified by column chromatography on silica gel, providing the desired product as white solid (1.05 g, 84% yield, 98% D). **^{1}H NMR** (400 MHz, $CDCl_3$) δ 8.68 (s, 1H), 7.33 – 7.26 (m, 2H), 7.25 – 7.18 (m, 3H), 4.60 (s, 0.04H), 1.66 (s, 2H), 1.38 (s, 6H), 0.99 (s, 9H).

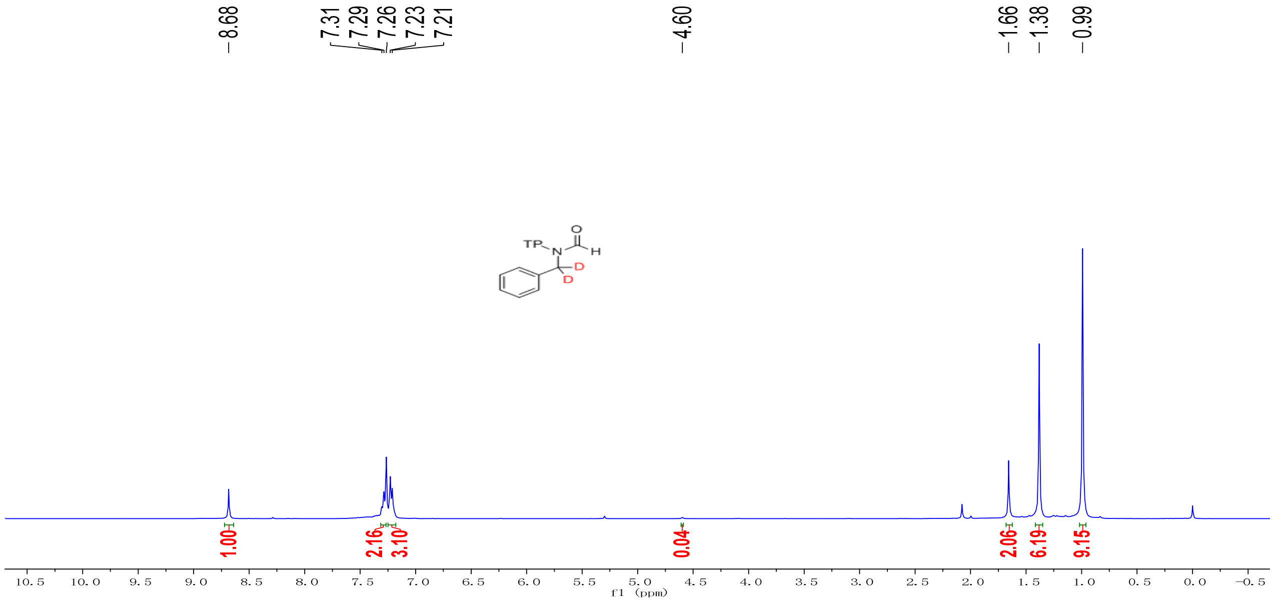

Supplementary Figure 9. ^{1}H NMR of compound **1a-*d*$_2$**.

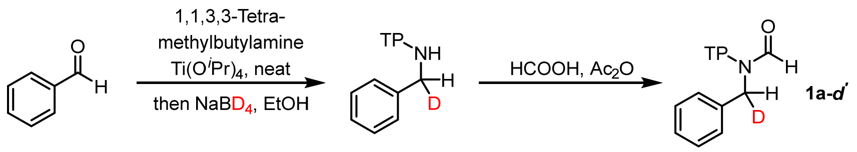

The synthesis method is the same as **1a-*d*$_2$**, afforded the desired product **1a-*d′*** as white solid (88% yield, >99% D). **^{1}H NMR** (400 MHz, $CDCl_3$) δ 8.68 (s, 1H), 7.33 – 7.26 (m, 2H), 7.25 – 7.18 (m, 3H), 4.60 (s, 1H), 1.66 (s, 2H), 1.38 (s, 6H), 0.99 (s, 9H).

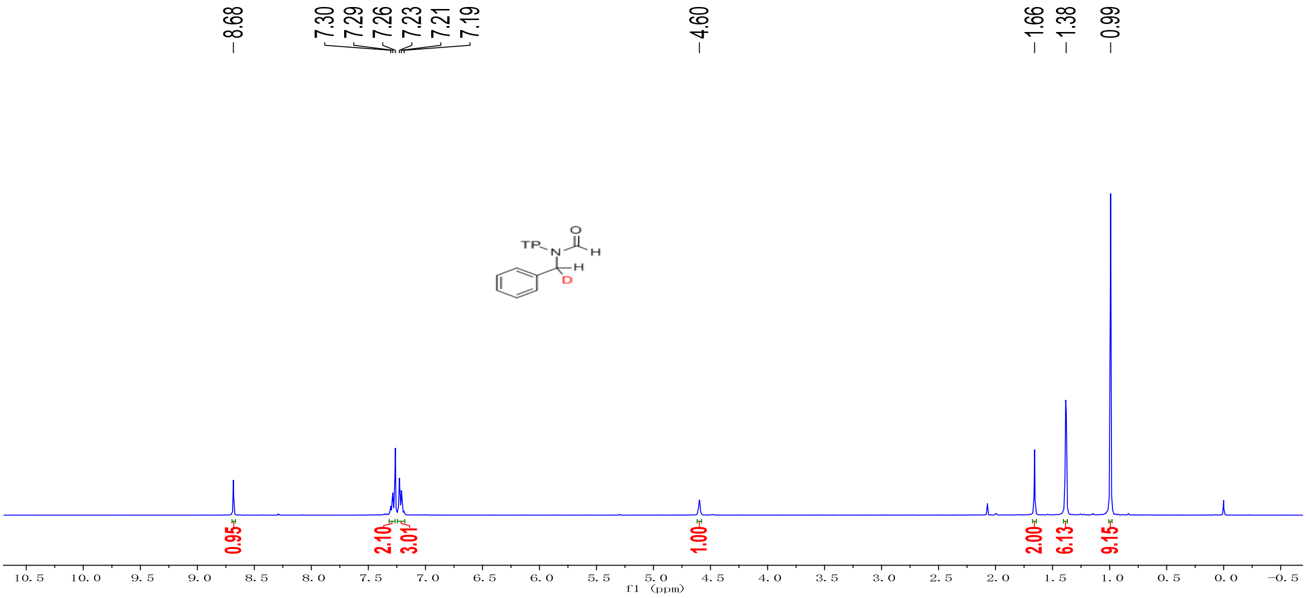

Supplementary Figure 10. ^{1}H NMR of compound **1a-*d'***.

Parallel reactions for KIE determination of carbonylic C–H of formamide

Parallel reactions were set up following the general procedure at 110 °C by using **1a** and **1a-*d*** as substrate respectively. Aliquots were taken at 15 minute intervals for the first 75 minutes. Product yield was determined by ^{1}H NMR using DMF as an internal standard. Data points represent the average of two runs.

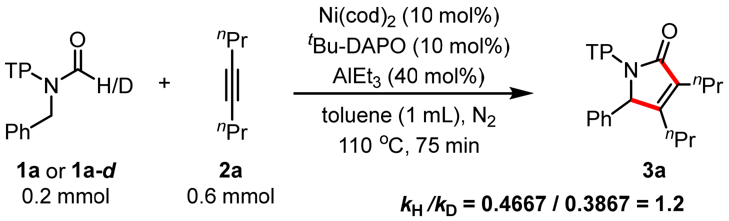

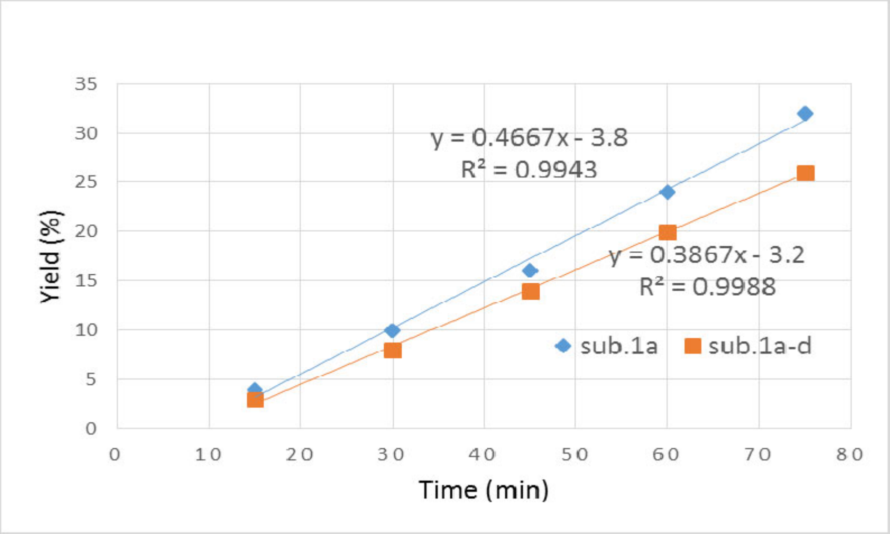

Supplementary Figure 11. KIE determination via parallel reactions of **1a** and **1a**-*d*.

Intramolecular competitive reaction

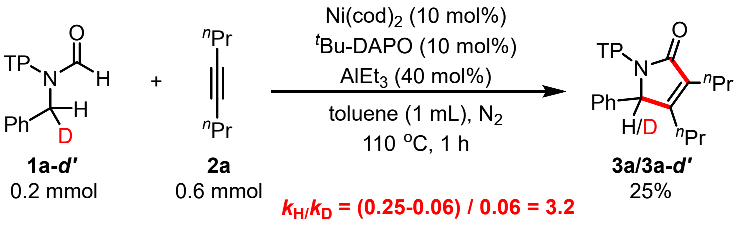

To a 15 mL oven dried tube was added PO ligand ^{t}Bu-DAPO (4.4 mg, 10 mol%), Ni(cod)$_2$ (5.5 mg, 10 mol%), dry degassed toluene (1.0 mL), **1a**-*d'* (49.6 mg, 0.2 mmol), oct-4-yne (88 μL, 0.6 mmol) and AlEt$_3$ (80 μL, 40 mol%) sequentially in an N$_2$-filled glove-box. The tube was sealed and removed out of the glove-box. After heated at 110 °C in a preheated dry block heater for 1 h, the mixture was cooled to r.t. and quenched with 0.1 mL of H$_2$O, then filtered through a short plug of silica gel (DCM as the eluent) and concentrated in vacuo. The yield was determined by ^{1}H NMR analysis of crude sample using 1,4-dinitrobenzene (16.8 mg, 0.1 mmol) as the internal standard. The k_H/k_D was obtained by calculation of the ratio of two products.

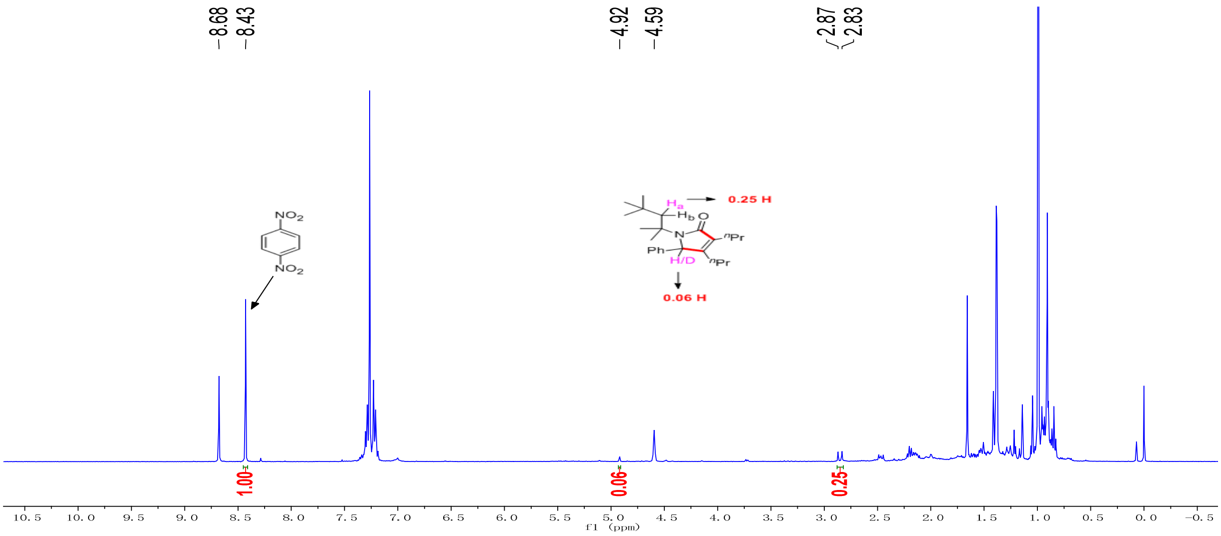

Supplementary Figure 12. KIE determination via intramolecular competitive reaction of **1a**-*d'*.

Intermolecular competitive reaction

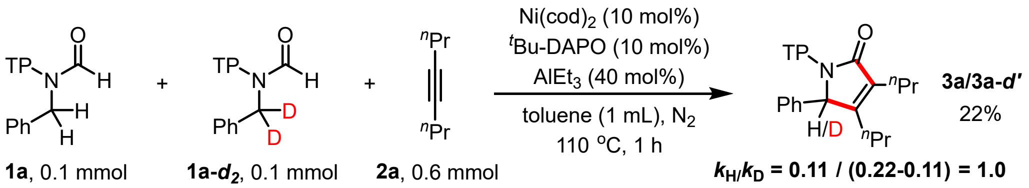

To a 15 mL oven dried tube was added PO ligand ^{t}Bu-DAPO (4.4 mg, 10 mol%), Ni(cod)$_2$ (5.5 mg, 10 mol%), dry degassed toluene (1.0 mL), **1a** (24.7 mg, 0.1 mmol), **1a**-*d*$_2$ (24.9 mg, 0.1 mmol), AlEt$_3$ (80 μL, 40 mol%) and oct-4-yne (88 μL, 0.6 mmol) and AlEt$_3$ (80 μL, 40 mol%) sequentially in an N$_2$-filled glove-box. The tube was sealed and removed out of the glove-box. After heated at 110 °C in a preheated dry block heater for 1 h, the mixture was cooled to r.t. and quenched with 0.1 mL of H$_2$O, then filtered through a short plug of silica gel (DCM as the eluent) and concentrated in vacuo. The yield was determined by ^{1}H NMR analysis of crude product using 1,4-dinitrobenzene (16.8 mg,

0.1 mmol) as the internal standard. The k_H/k_D was obtained by calculating the ratio of two products.

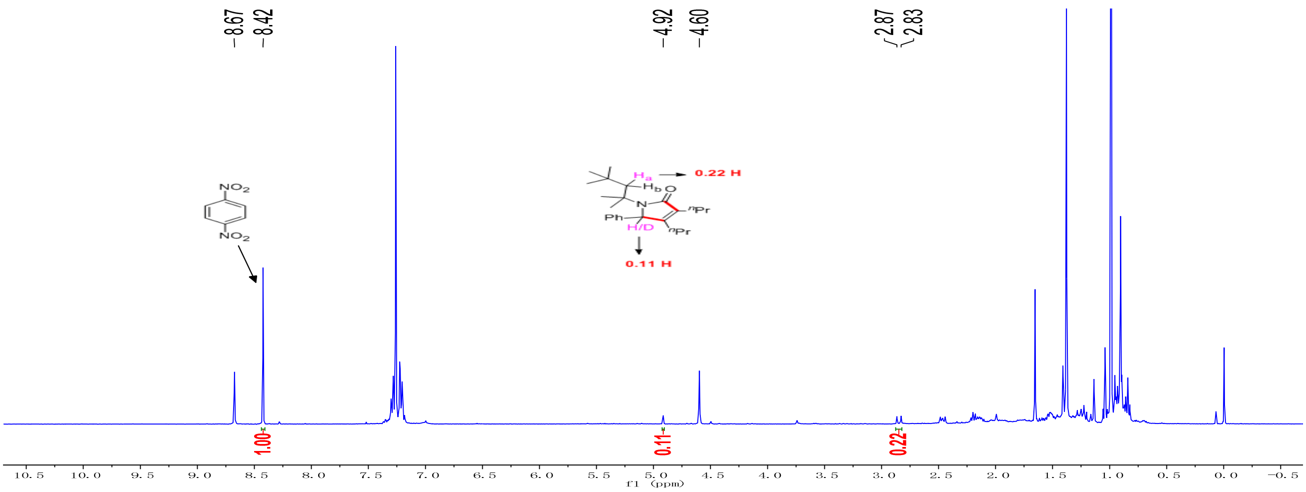

Supplementary Figure 13. KIE determination via competitive reaction of .**1a** and **1a**-d_2.

Parallel reactions for KIE determination of C–H of formamide

Parallel reactions were set up following the general procedure at 110 °C by using **1a** and **1a-*d*** as substrate respectively. Aliquots were taken at intervals for the first 60 minutes. Product yield was determined by ^{1}H NMR using DMF as an internal standard. Data points represent the average of two runs.

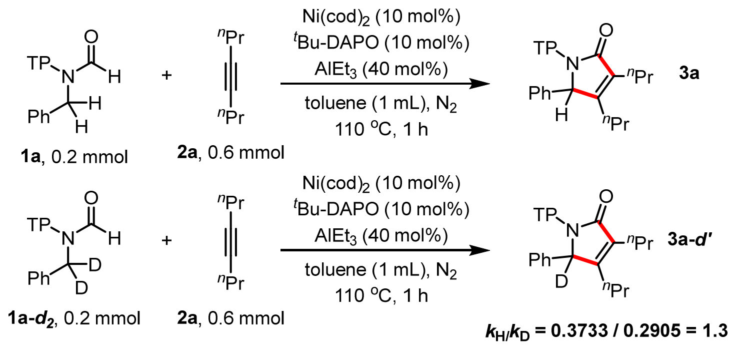

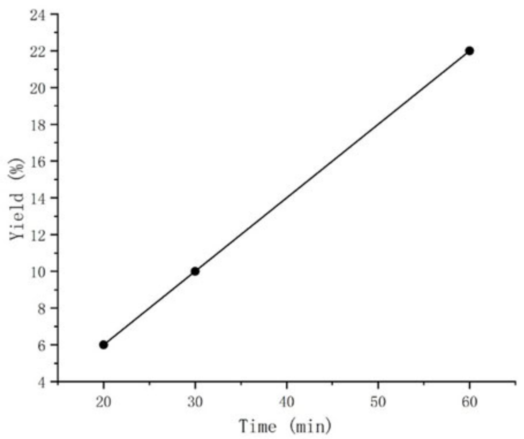

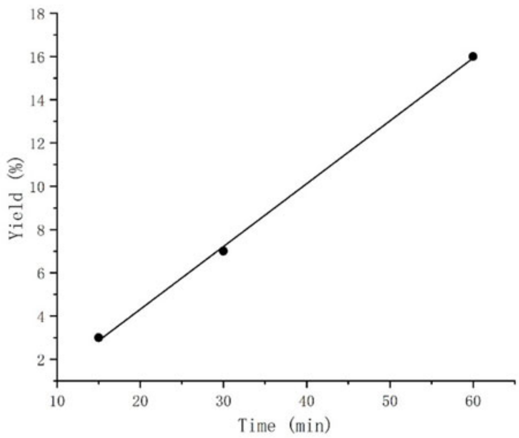

reactions of 1a: $y = 0.3733x + 0.5$; $R^2 = 0.9987$. reactions of 1a-d_2: $y = 0.2905x - 1.5$; $R^2 = 0.9992$.

Supplementary Figure 14. KIE determination via parallel reactions of **1a** and **1a**-*d*.

Kinetic studies: measure reaction order of oct-4-yne

Parallel reactions were set up following the general procedure at 110 °C by using **1a** as substrate. Aliquots were taken at intervals. Product yield was determined by ^{1}H NMR using DMF as an internal standard. Data points represent the average of two runs.

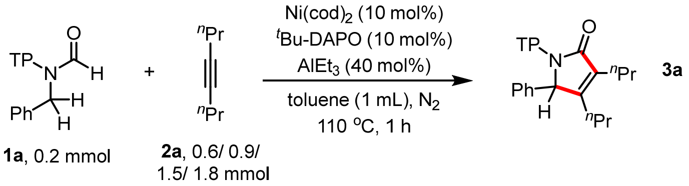

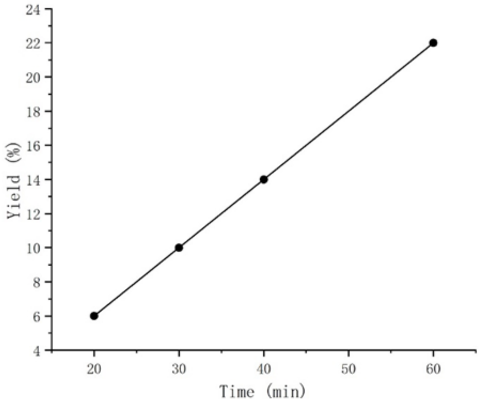

3 equiv of oct-4-yne: $y = 0.4x - 2$, $R^2 = 1$

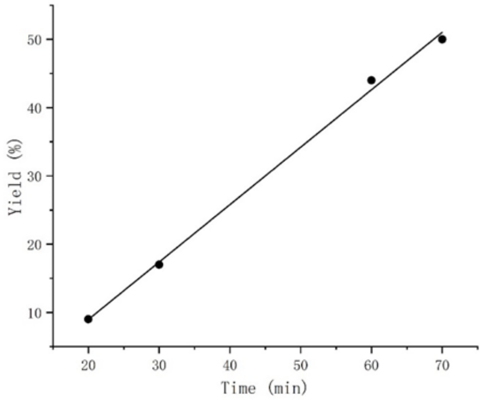

4.5 equiv of oct-4-yne: $y = 0.8412x - 7.8529$, $R^2 = 0.9974$

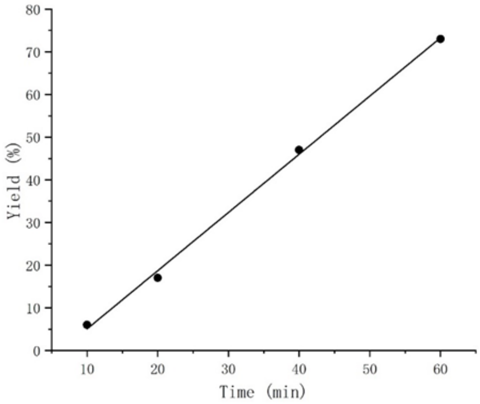

7.5 equiv of oct-4-yne: $y = 1.3644x - 8.5932$; $R^2 = 0.9982$

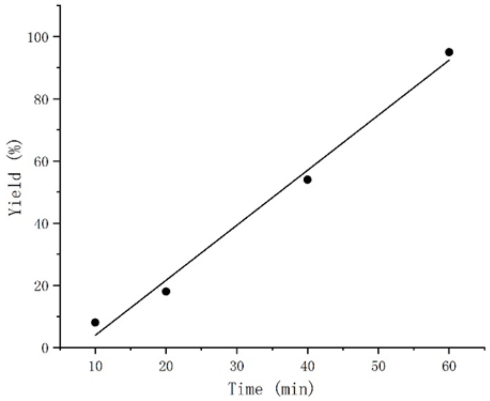

9 equiv of oct-4-yne: $y = 1.7711x - 13.8136$; $R^2 = 0.9854$

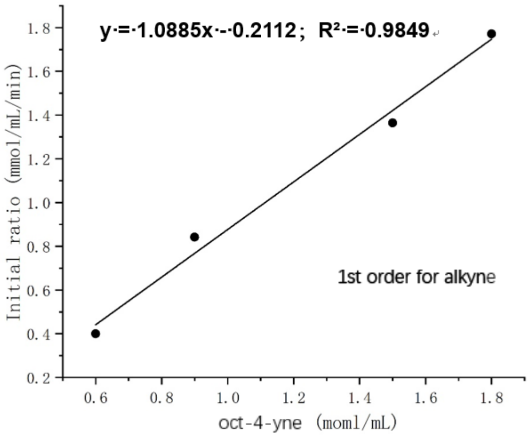

Supplementary Figure 15. Reaction rate order measure of oct-4-yne.

Electronic effect

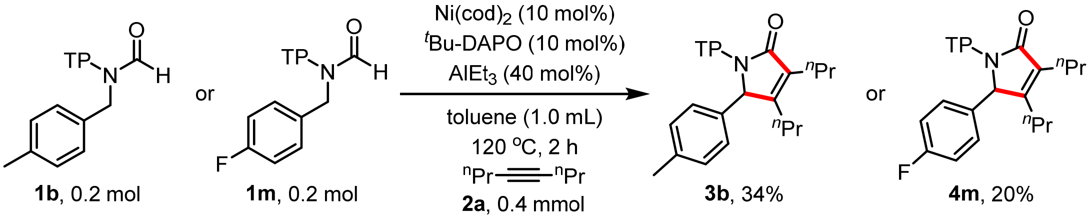

To a 15 mL oven-dried tube was added PO ligand ^{t}Bu-DAPO (4.4 mg, 10 mol%), Ni(cod)$_2$ (5.5 mg, 10 mol%), dry degassed toluene (1.0 mL), **1b** (52.2 mg, 0.2 mmol), **1m** (53.0 mg, 0.2 mmol), oct-4-yne (58 µL, 0.4 mmol) and $AlEt_3$ (1.0 M in toluene, 80 µL, 40 mol%) sequentially in an N_2-filled glove-box. The tube was sealed and removed out of the glove-box. After heated at 120 °C in a dry block heater for 2 h, the mixture was cooled to r.t., quenched with 0.1 mL H_2O, filtered through a short plug of silica gel (DCM as the eluent) and concentrated in vacuo to afford the crude product. The yield was determined by ^{1}H NMR analysis of crude product using DMF as the internal standard.

Alkene determination

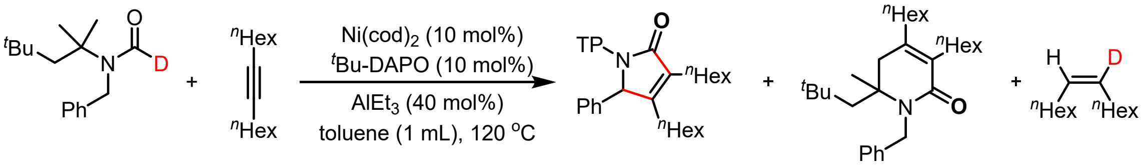

To a 15 mL oven-dried tube was added PO ligand ^{t}Bu-DAPO (4.4 mg, 10 mol%), Ni(cod)$_2$ (5.5 mg, 10 mol%), dry degassed toluene (1.0 mL), **1a** (52.2 mg, 0.2 mmol), tetradec-7-yne (0.6 mmol) and $AlEt_3$ (1.0 M in toluene, 80 µL, 40 mol%) sequentially in an N_2-filled glove-box. The tube was sealed and removed out of the glove-box. After heated at 120 °C in a dry block heater for 2 h, the mixture was cooled to r.t., quenched with 0.1 mL H_2O, filtered through a short plug of silica gel (DCM as the eluent) and concentrated in vacuo to afford the crude product. The yield was determined by ^{1}H NMR analysis and GC analysis of crude product.

Supplementary Note 7

DFT Calculations

Computational Details

All the calculations were performed at the B3LYP-D3(BJ)[7–9] level of theory using Gaussian 09 package[10]. The B3LYP-D3(BJ) functional has been demonstrated to provide reliable results for the Ni-Al co-catalyzed C-H activation reactions[11]. The geometry optimizations were carried out with the basis set of def2-SVP[12]. Frequencies were computed analytically at the same level of theory to

confirm whether the structures are minima (no imaginary frequencies) or transition states (only one imaginary frequency). Selected transition-state structures were confirmed to connect the correct reactants and products by intrinsic reaction coordinate (IRC) calculations.[13,14] To obtain better accuracy, energies for the optimized geometries were recalculated using the solution-phase single-point calculations with a larger basis set of def2-TZVPP[12,15]. Solvation effects (solvent = toluene, ε = 2.374) were taken into account by performing single-point calculations with the SMD model.[16] The final free energies reported in the article are the large basis set single-point energies corrected by gas-phase Gibbs free energy correction (at 298.15 K).

Alternative pathway for the first formyl C–H activation

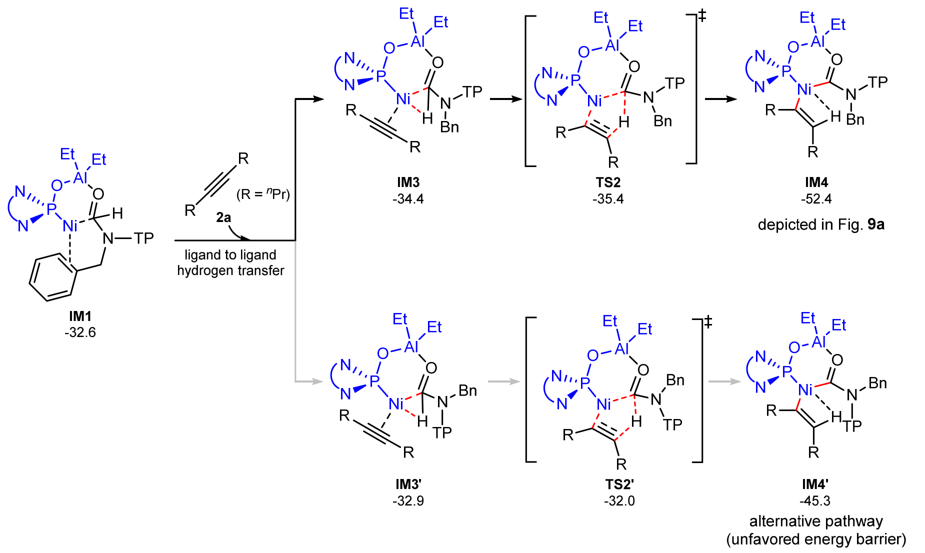

Supplementary Figure 16. Comparison of two possible pathways for formyl C–H bond activation.

Selective activation of benzylic *β*-C(sp^3)–H bond over *γ*-C(sp^3)–H bonds of TP group

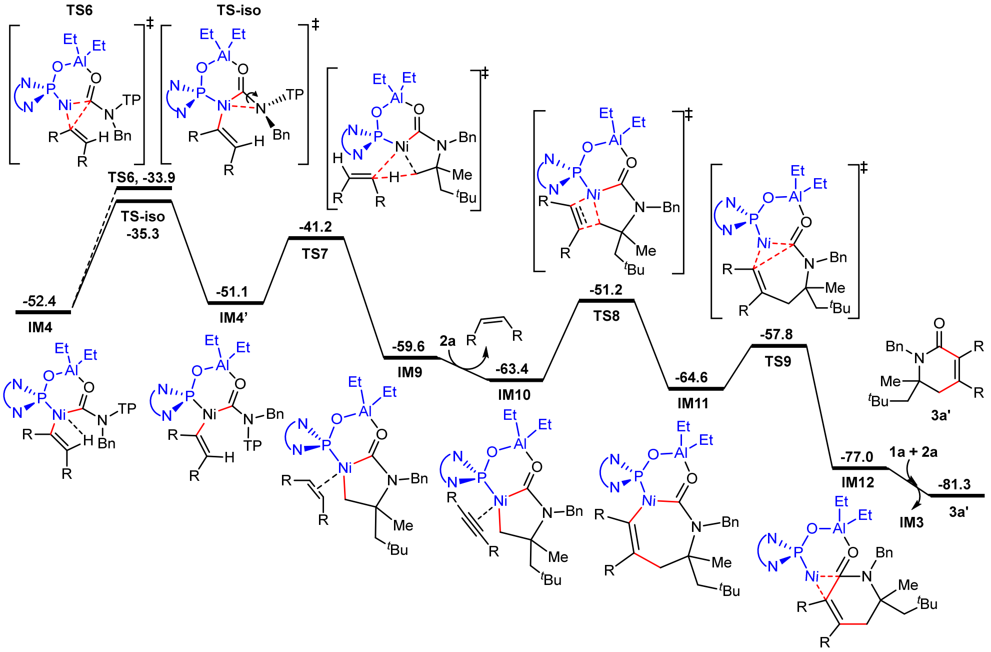

Supplementary Figure 17. Selective activation of benzylic C–H group overriding TP group is attributed to high rotation energy barrier (**TS-iso**, activation Gibbs energy of 17.1 kcal/mol).

Supplementary Figures

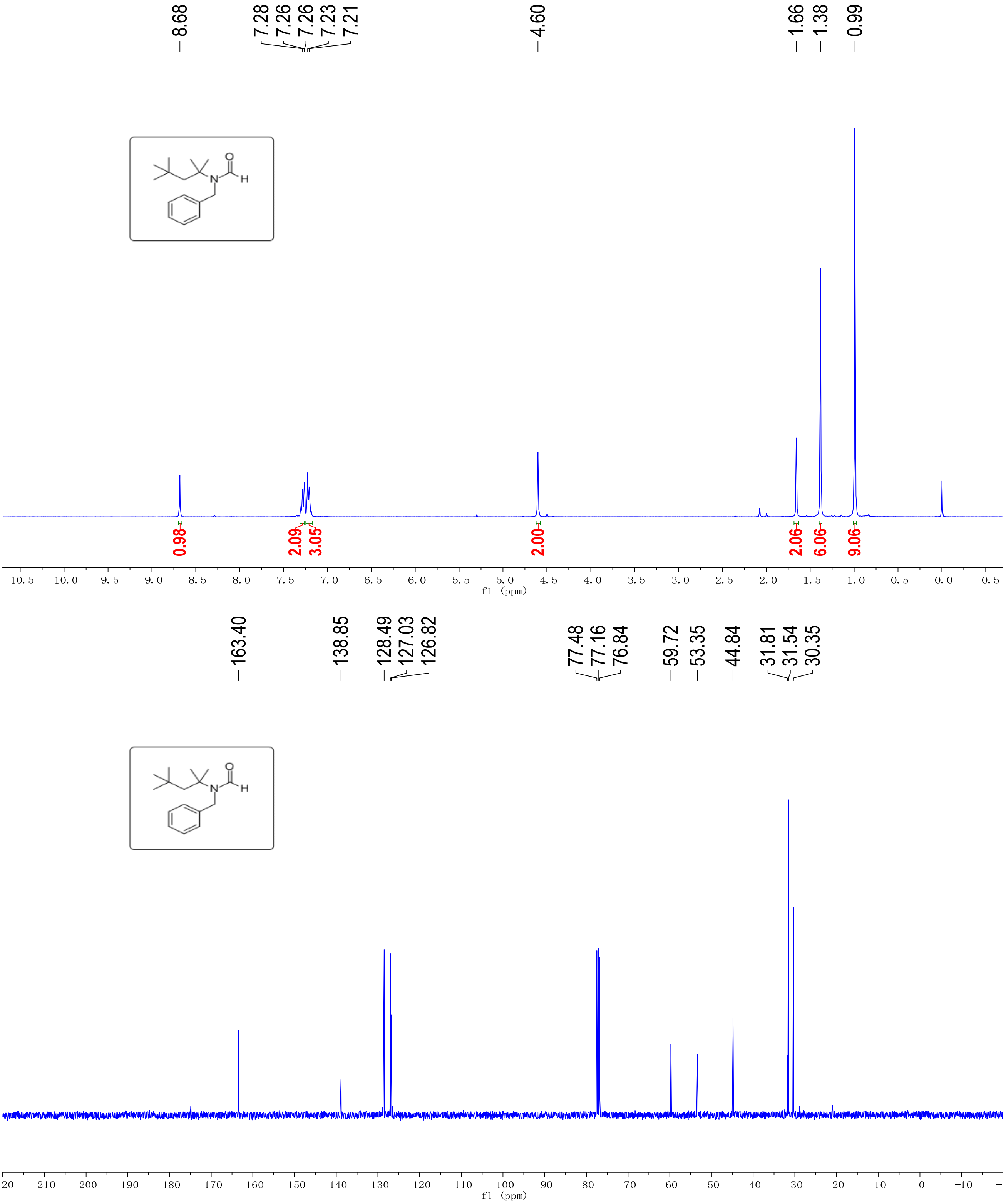

Supplementary Figure 18. ^{1}H and ^{13}C NMR spectra of compound **4** in $CDCl_3$

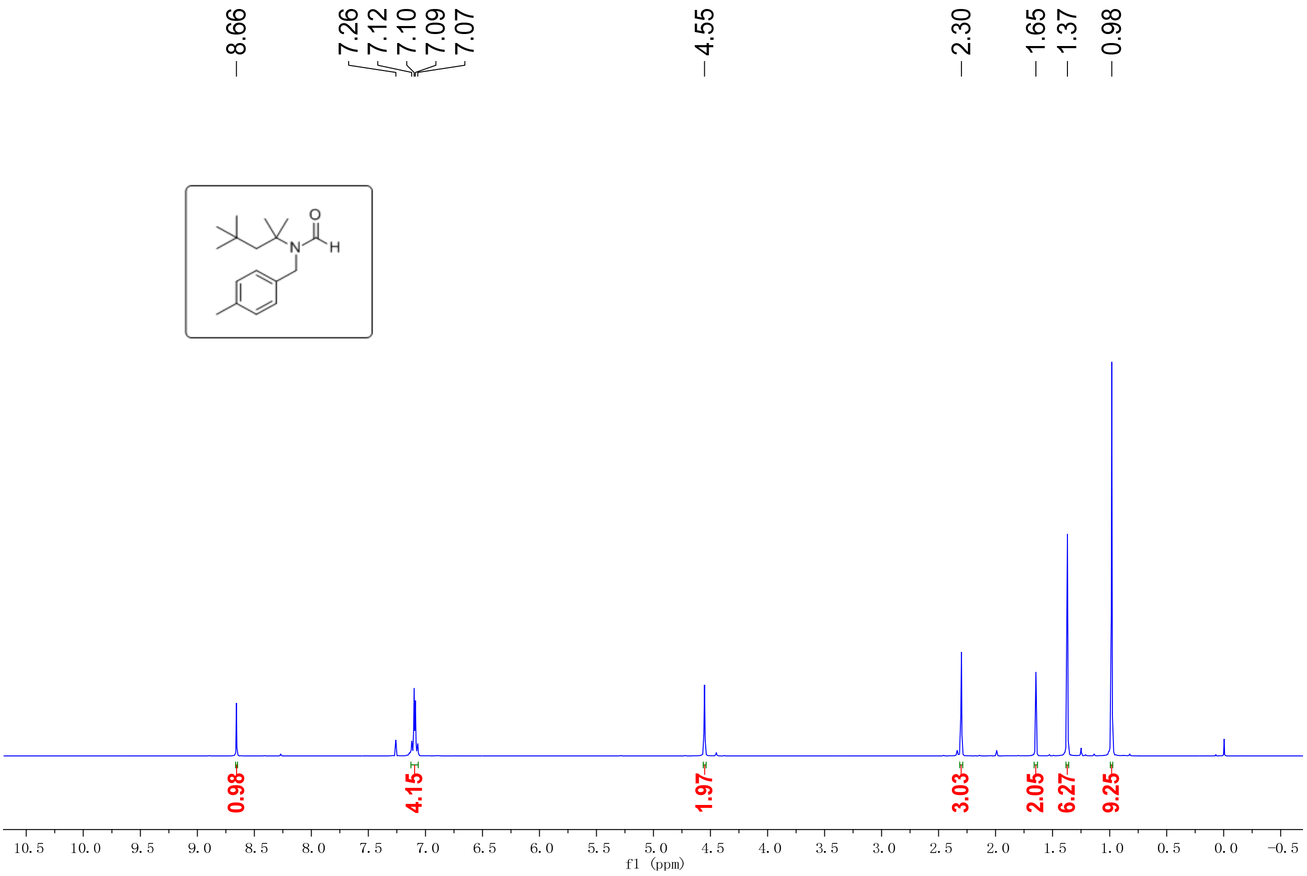

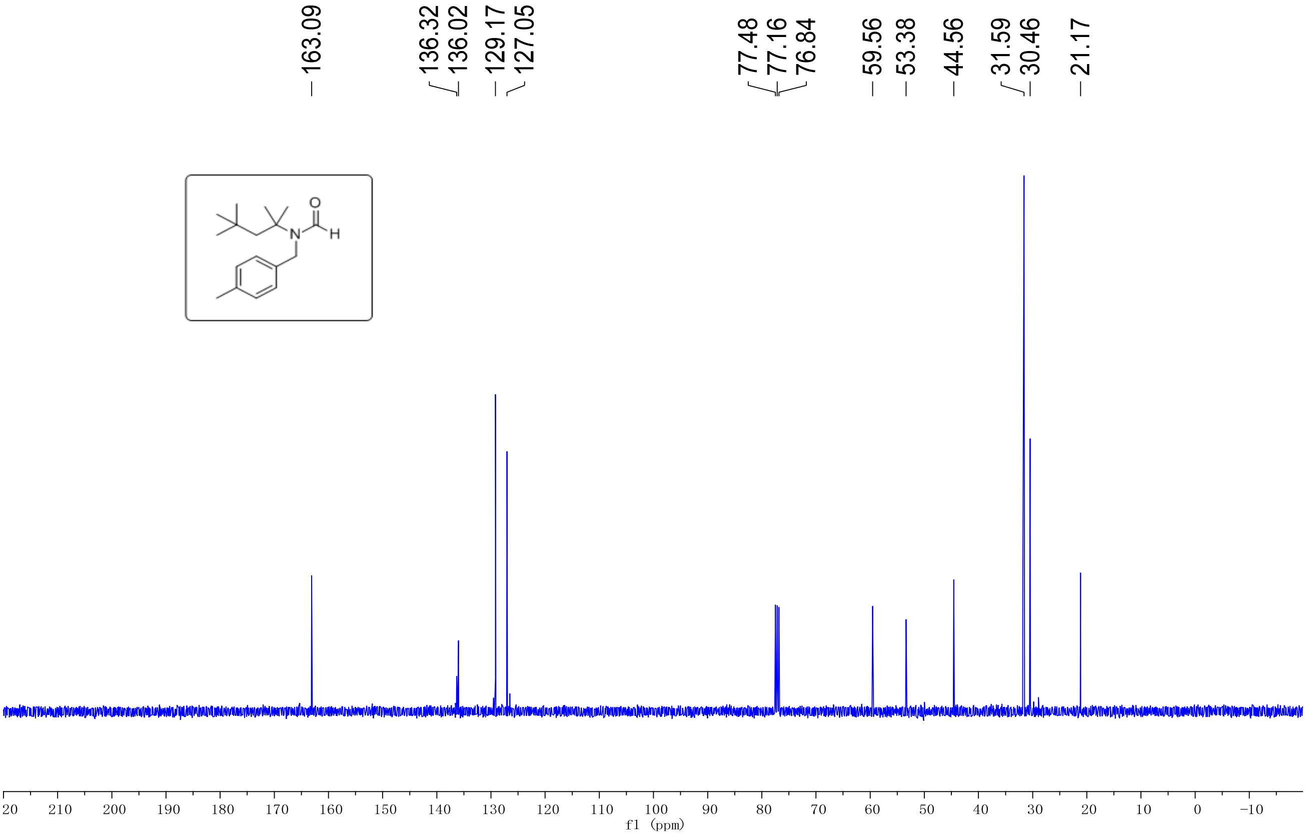

Supplementary Figure 19. ^{1}H and ^{13}C NMR spectra of compound **1b** in $CDCl_3$

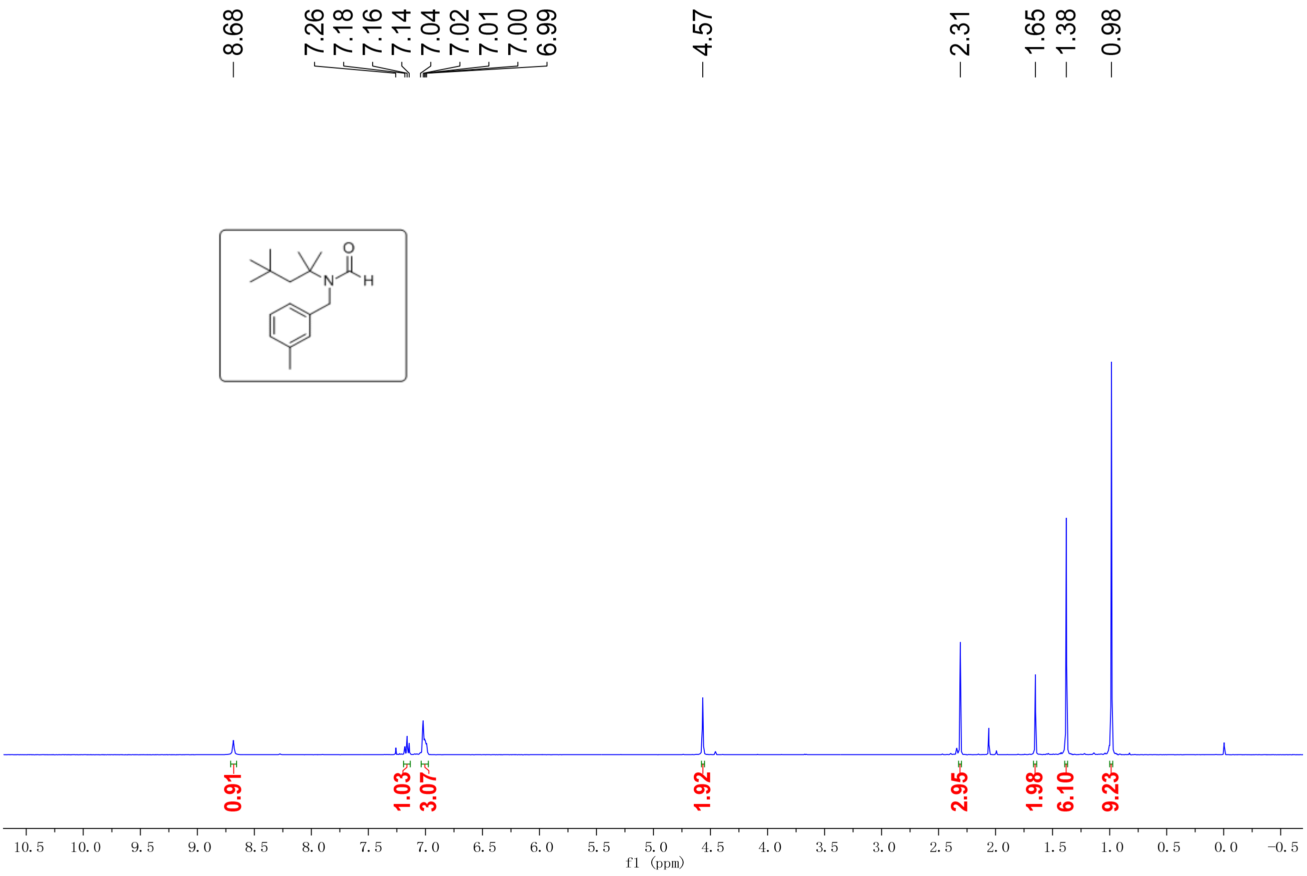

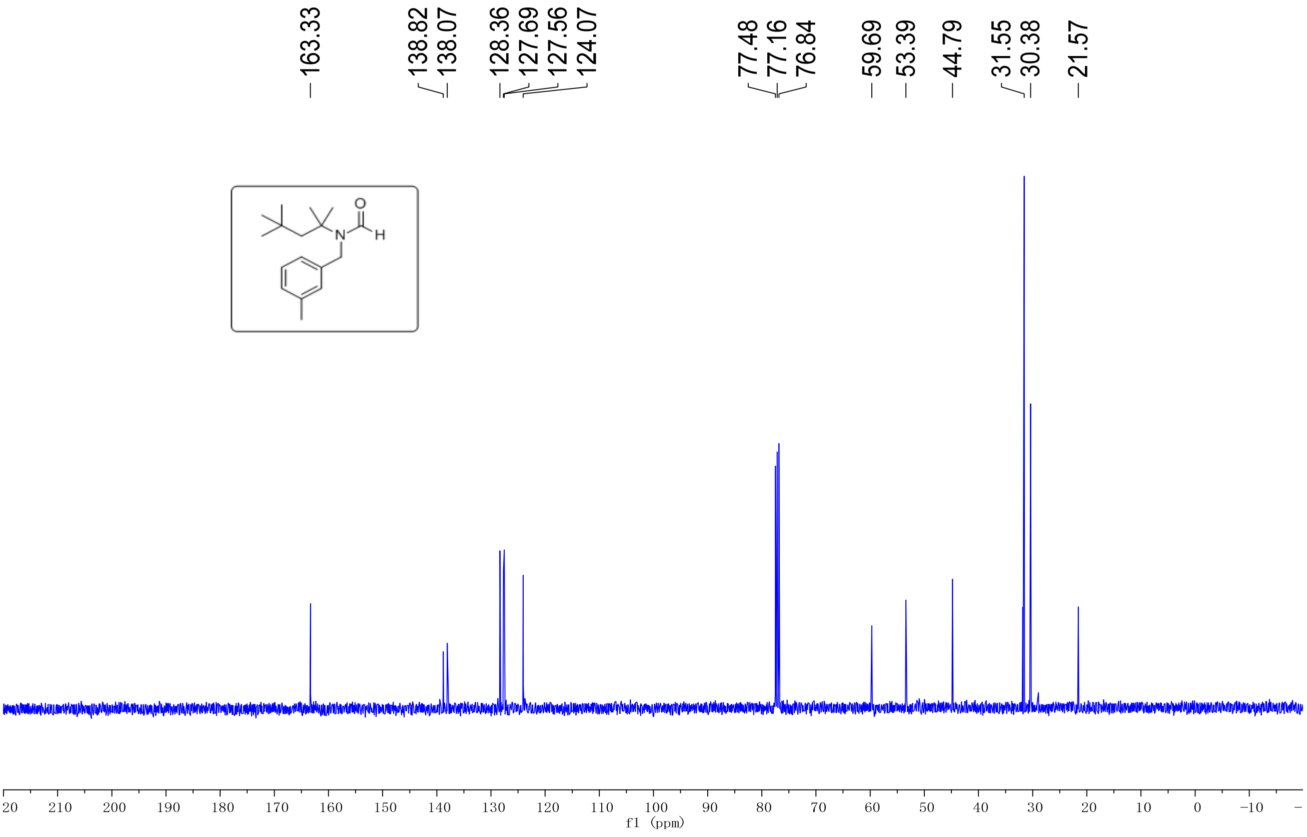

Supplementary Figure 20. ^{1}H and ^{13}C NMR spectra of compound **1c** in $CDCl_3$

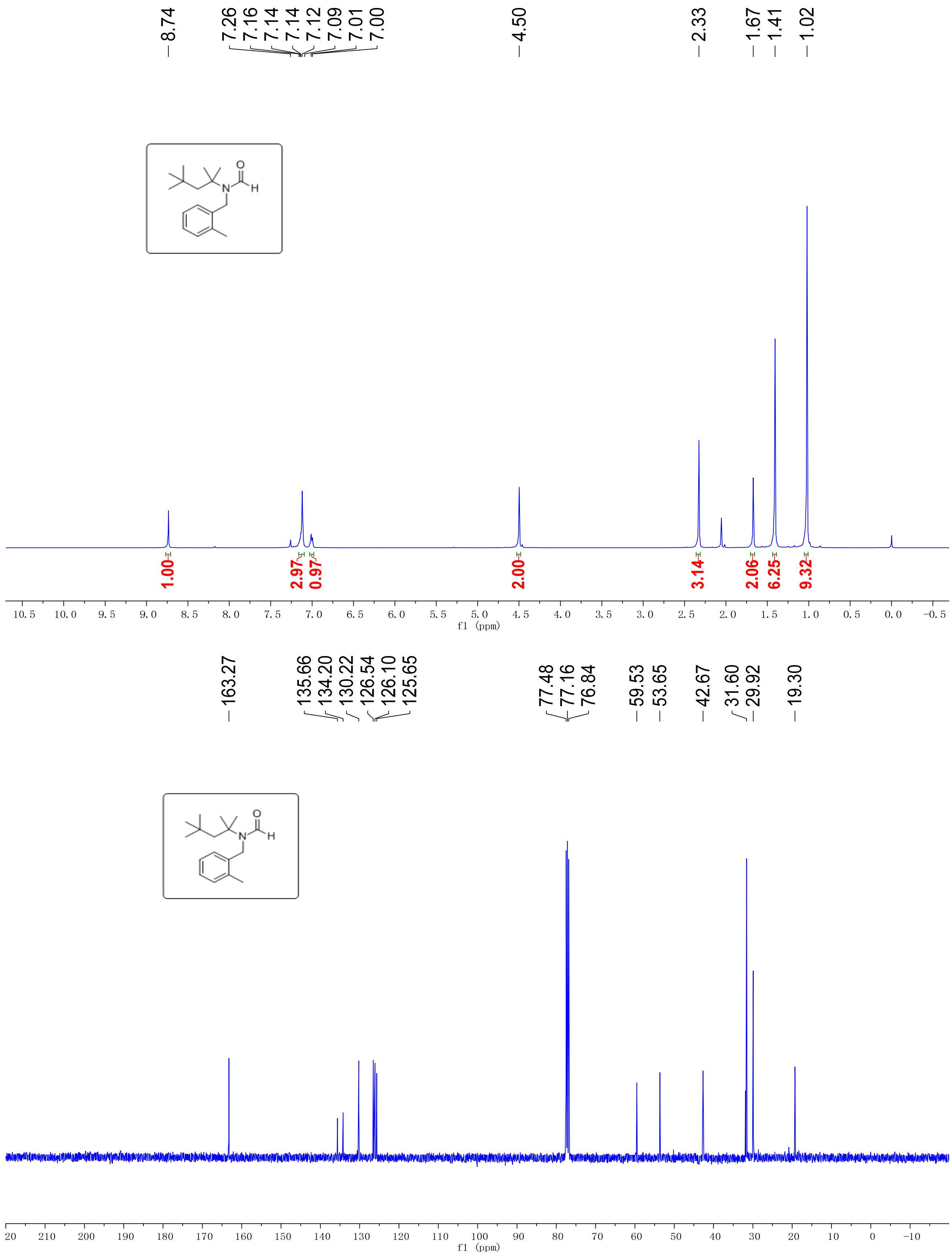

Supplementary Figure 21. ^{1}H and ^{13}C NMR spectra of compound **1d** in $CDCl_3$

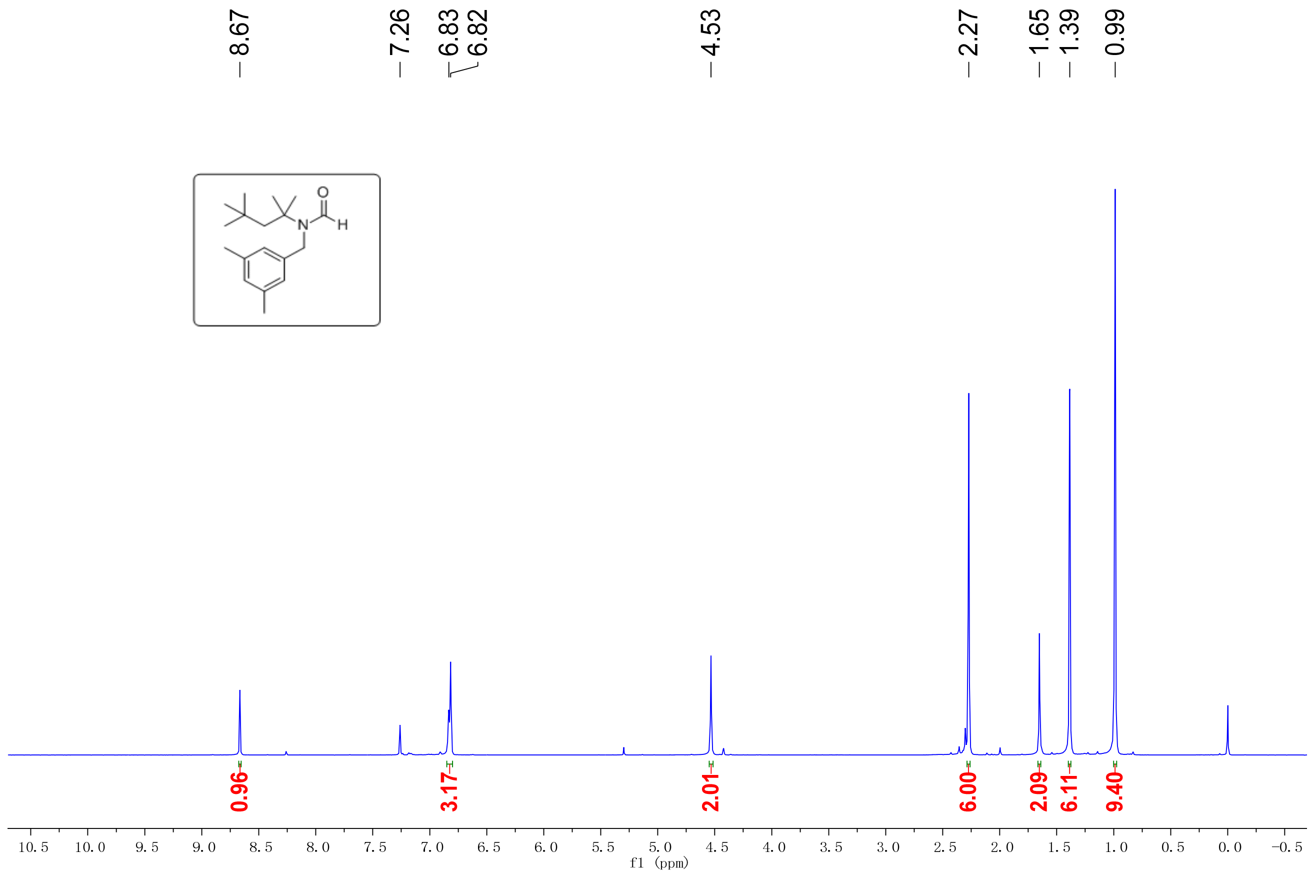

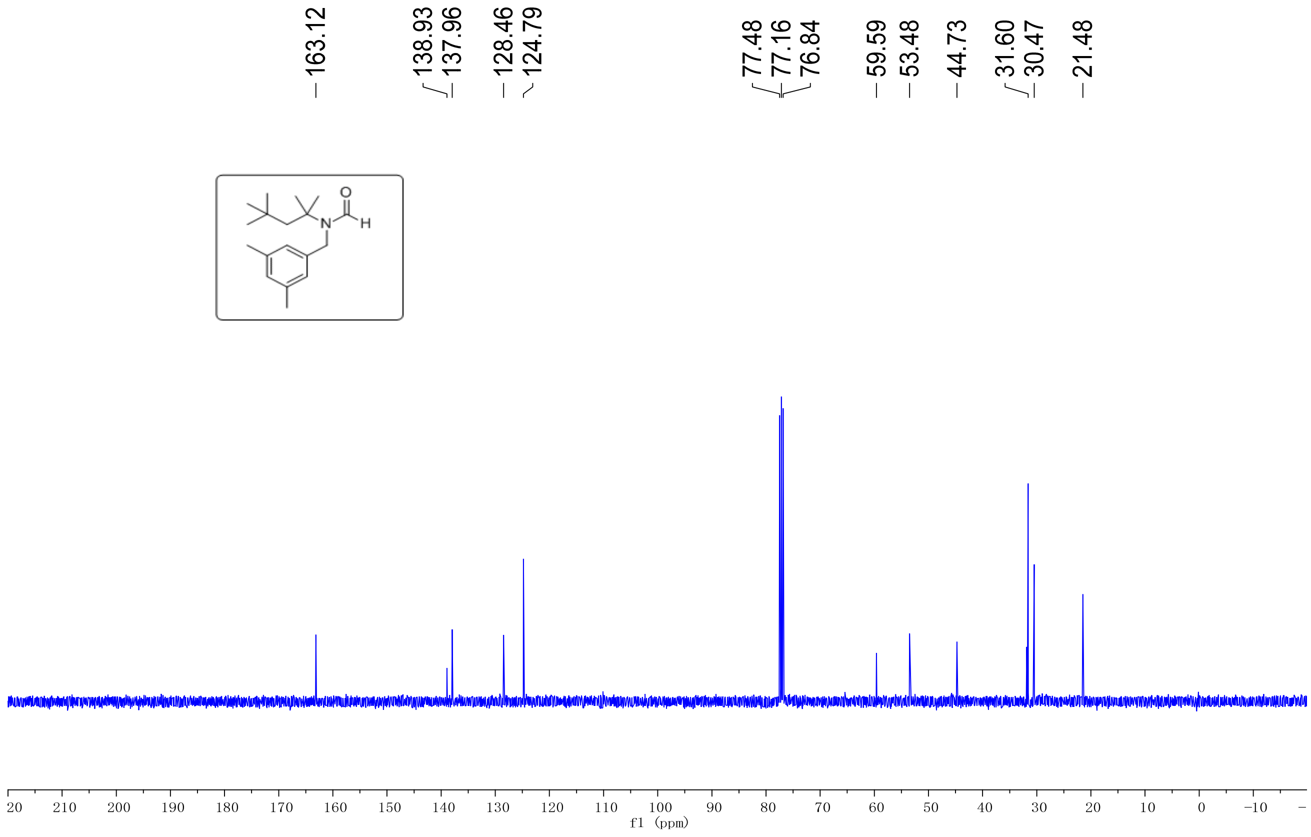

Supplementary Figure 22. ^{1}H and ^{13}C NMR spectra of compound **1e** in $CDCl_3$

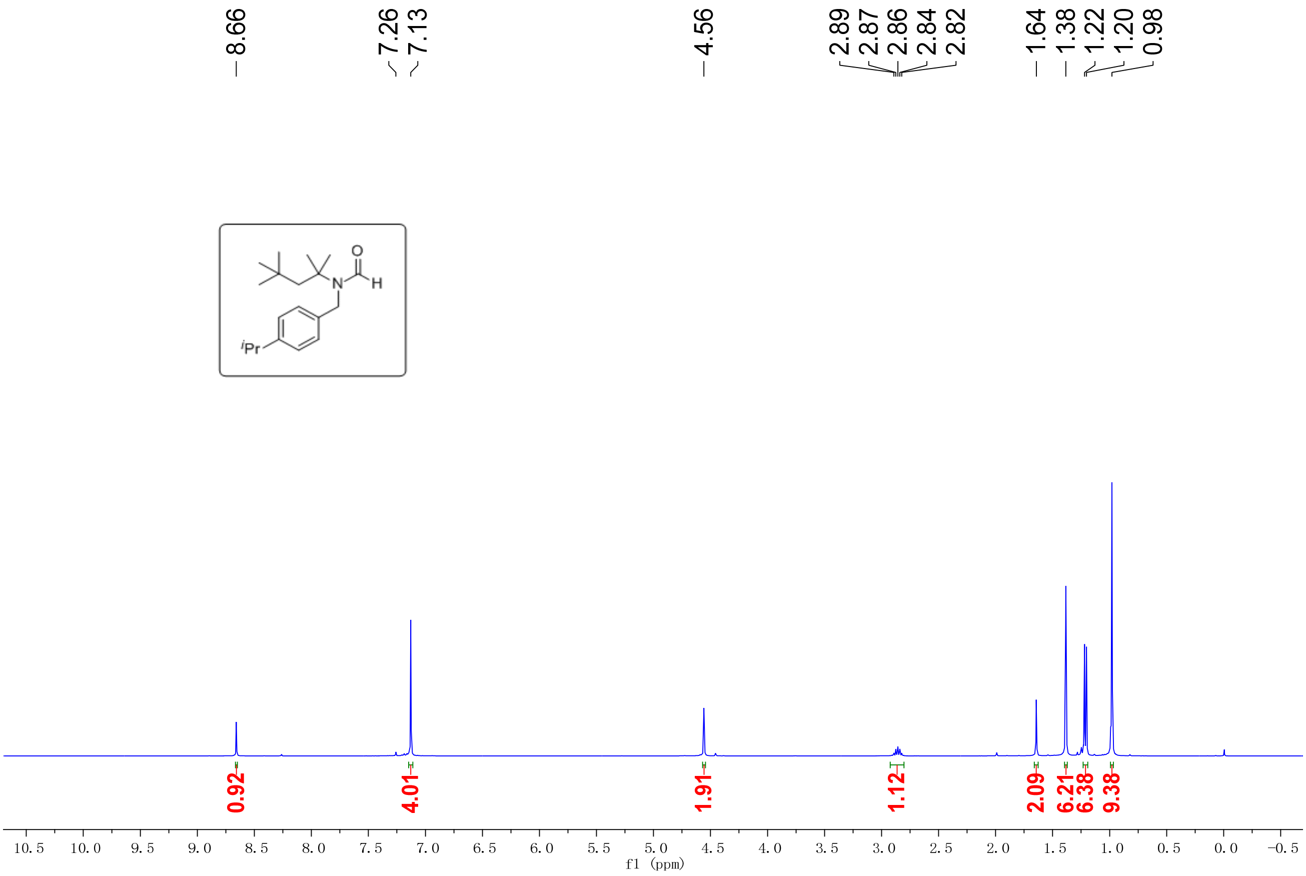

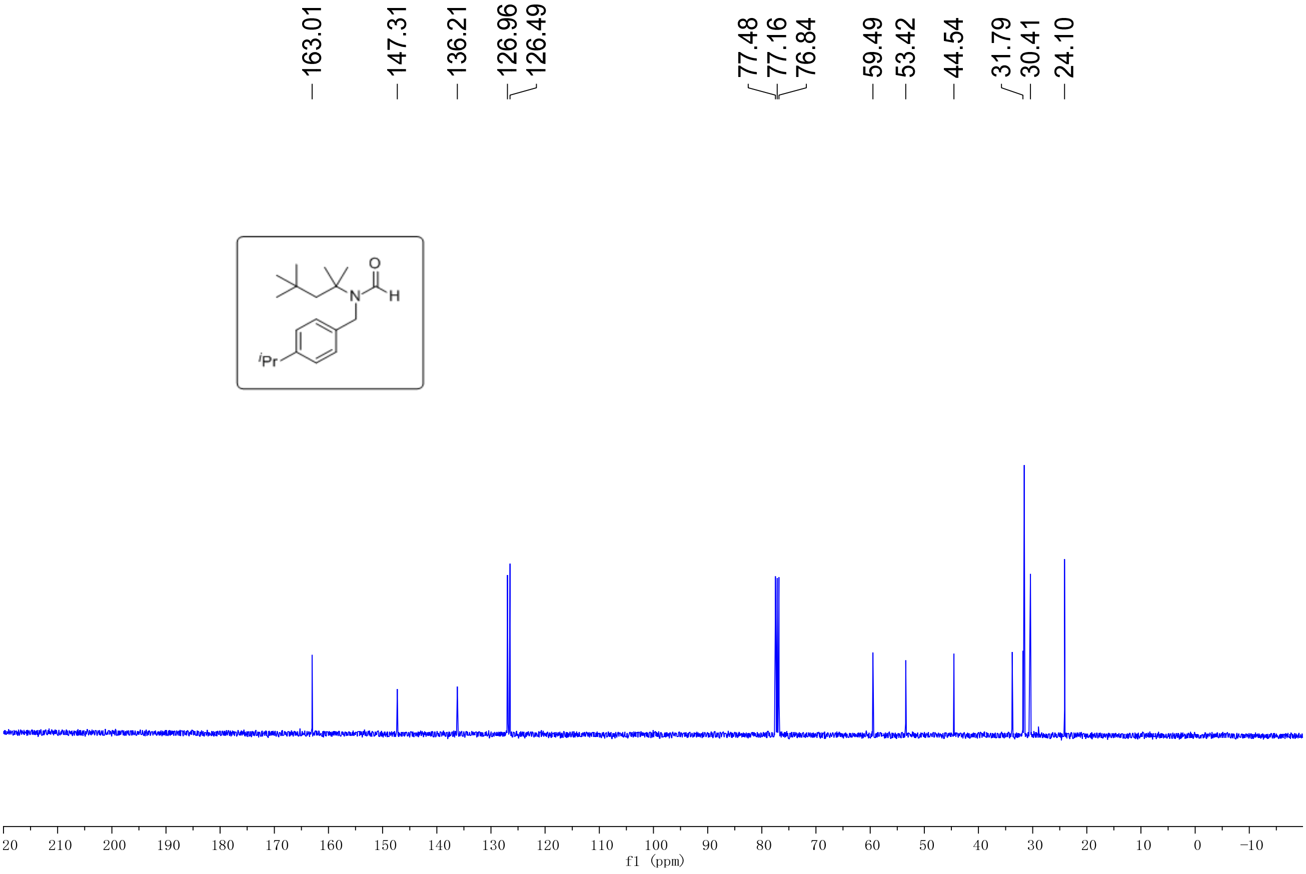

Supplementary Figure 23. ^{1}H and ^{13}C NMR spectra of compound **1f** in $CDCl_3$

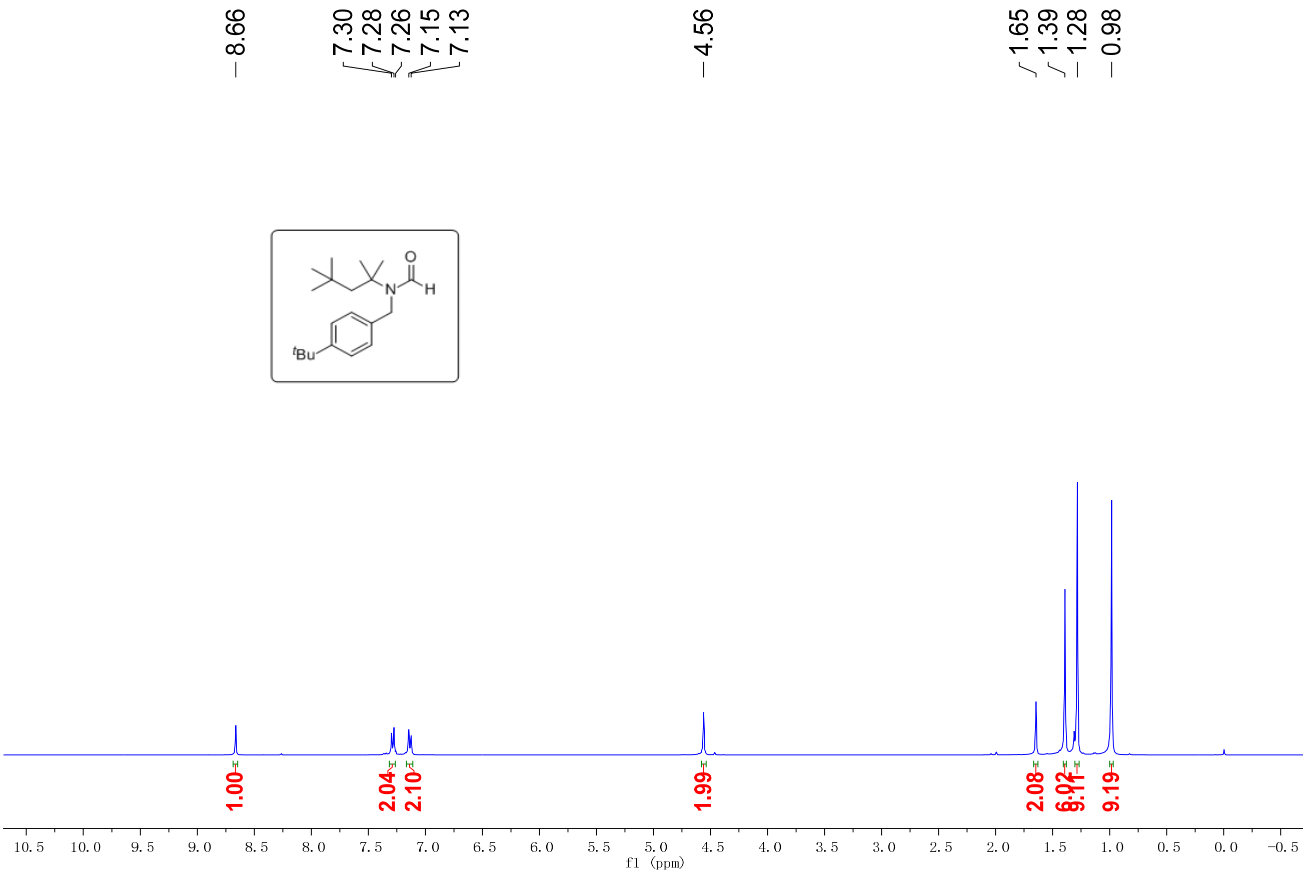

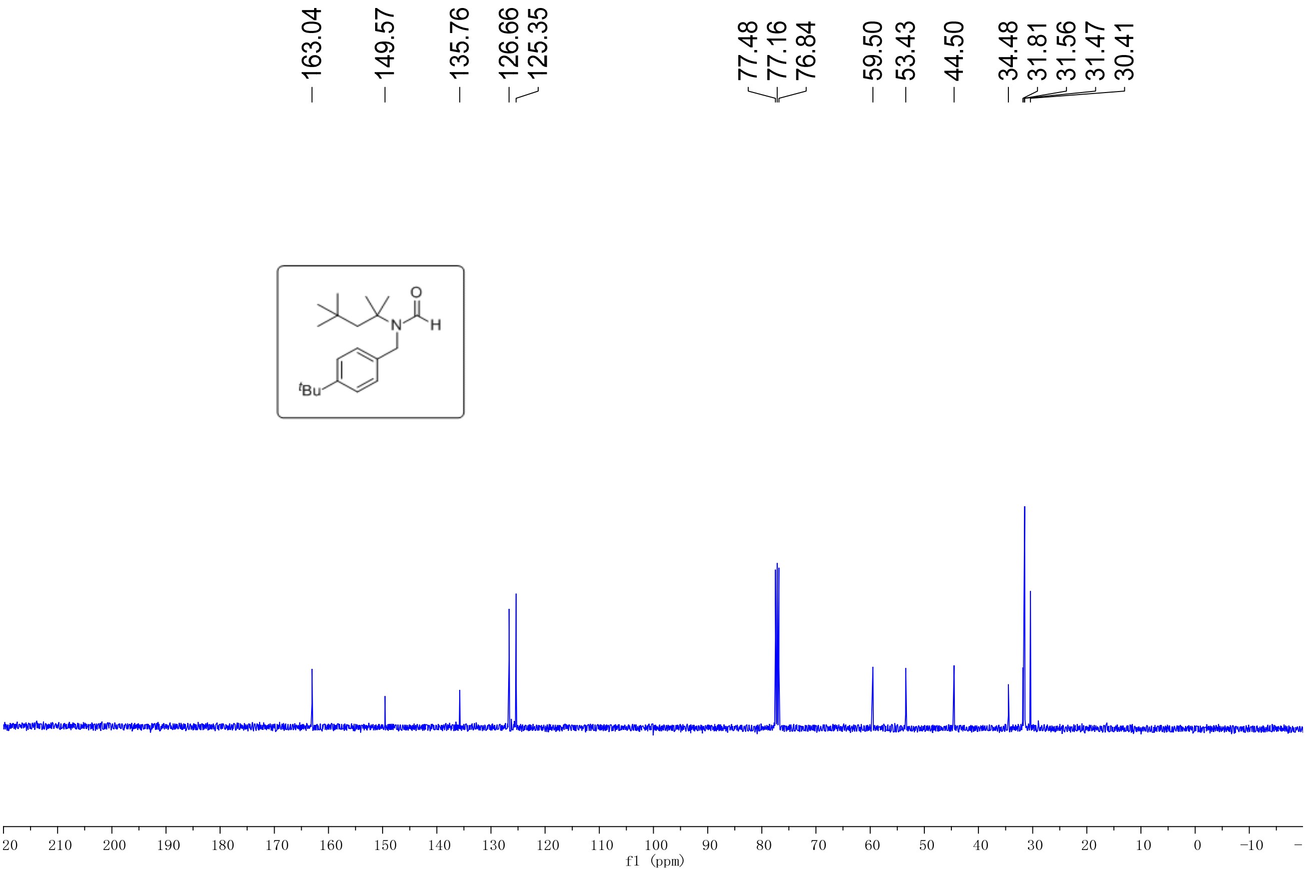

Supplementary Figure 24. ^{1}H and ^{13}C NMR spectra of compound **1g** in $CDCl_3$

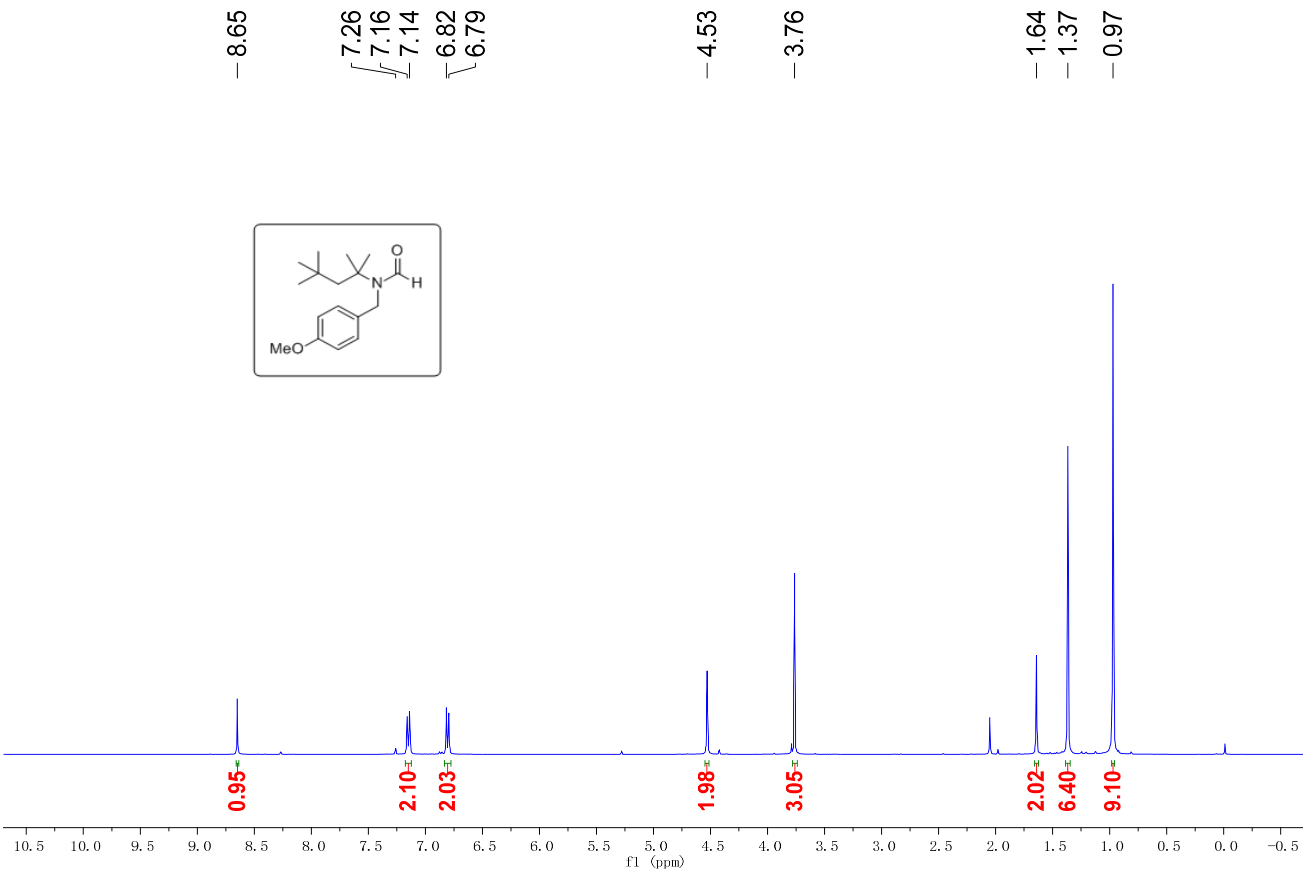

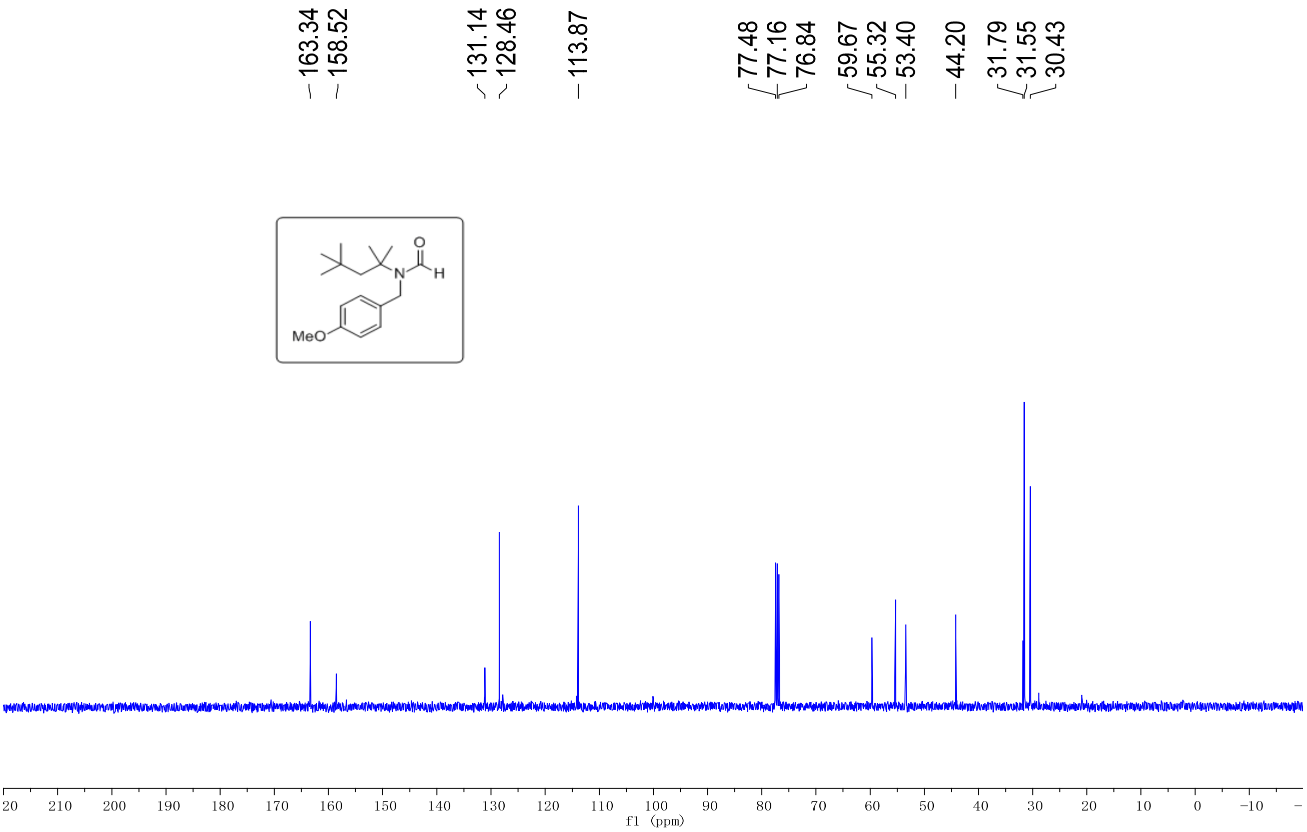

Supplementary Figure 25. ^{1}H and ^{13}C NMR spectra of compound **1h** in $CDCl_3$

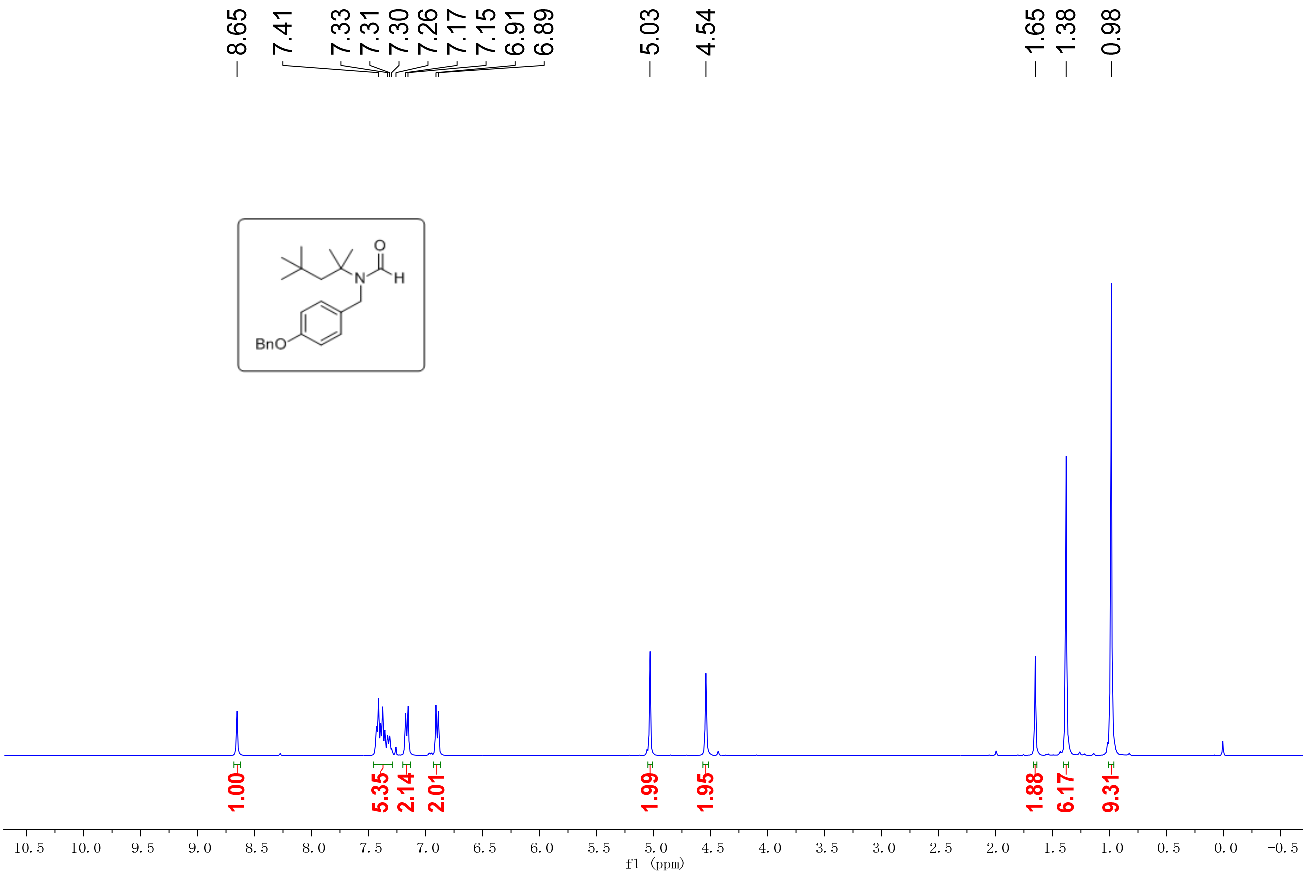

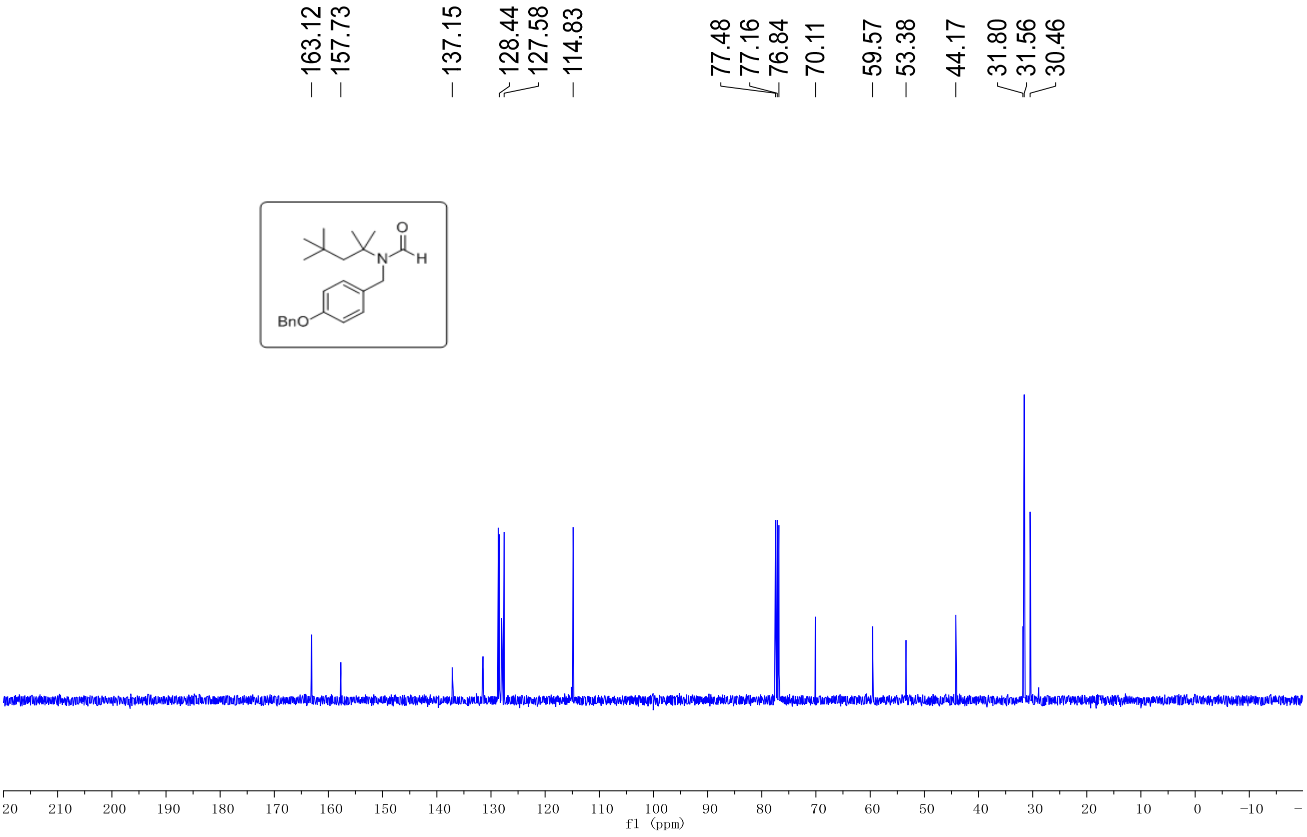

Supplementary Figure 26. ^{1}H and ^{13}C NMR spectra of compound **1i** in $CDCl_3$

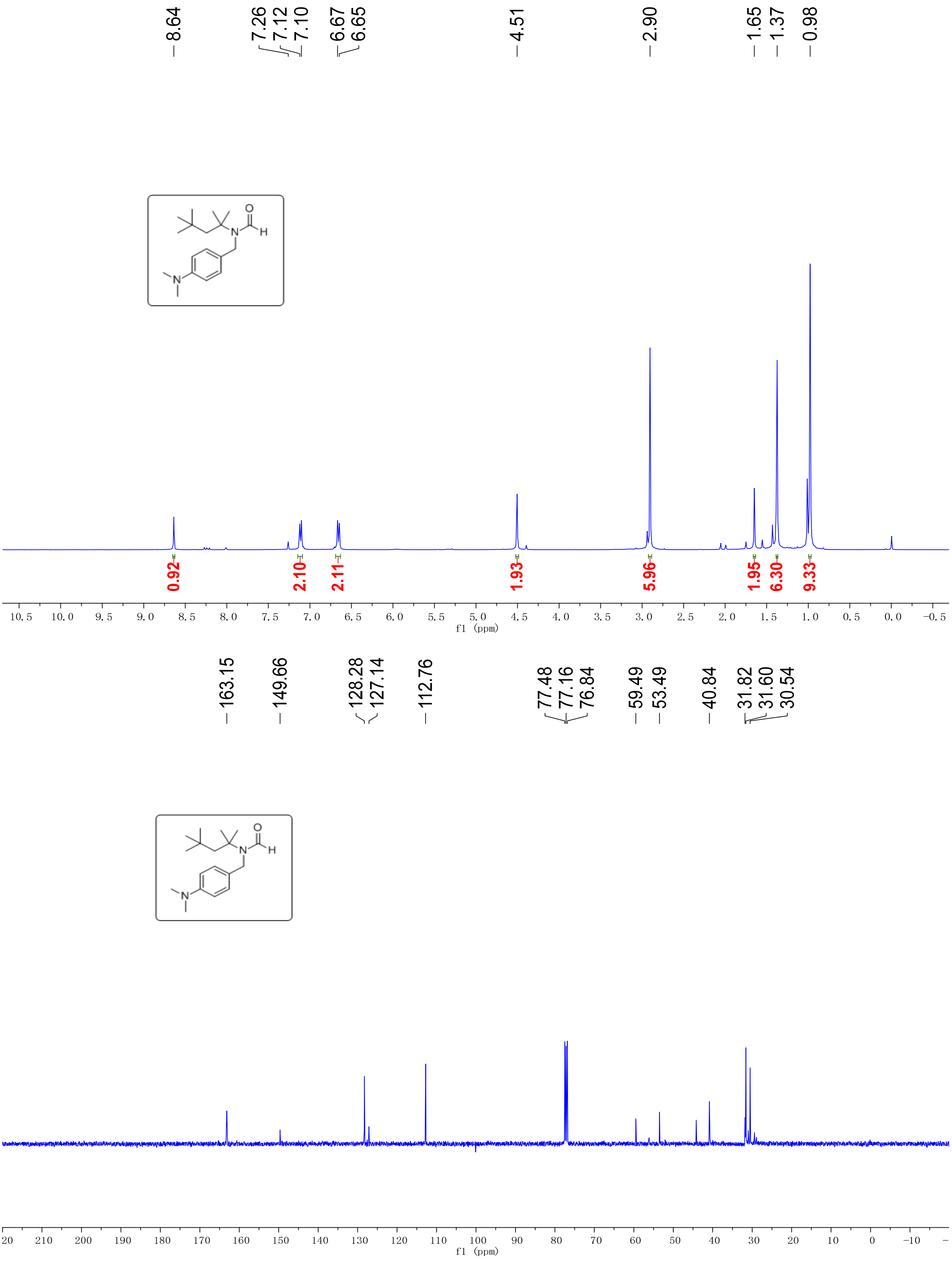

Supplementary Figure 27. ^{1}H and ^{13}C NMR spectra of compound **1j** in $CDCl_3$

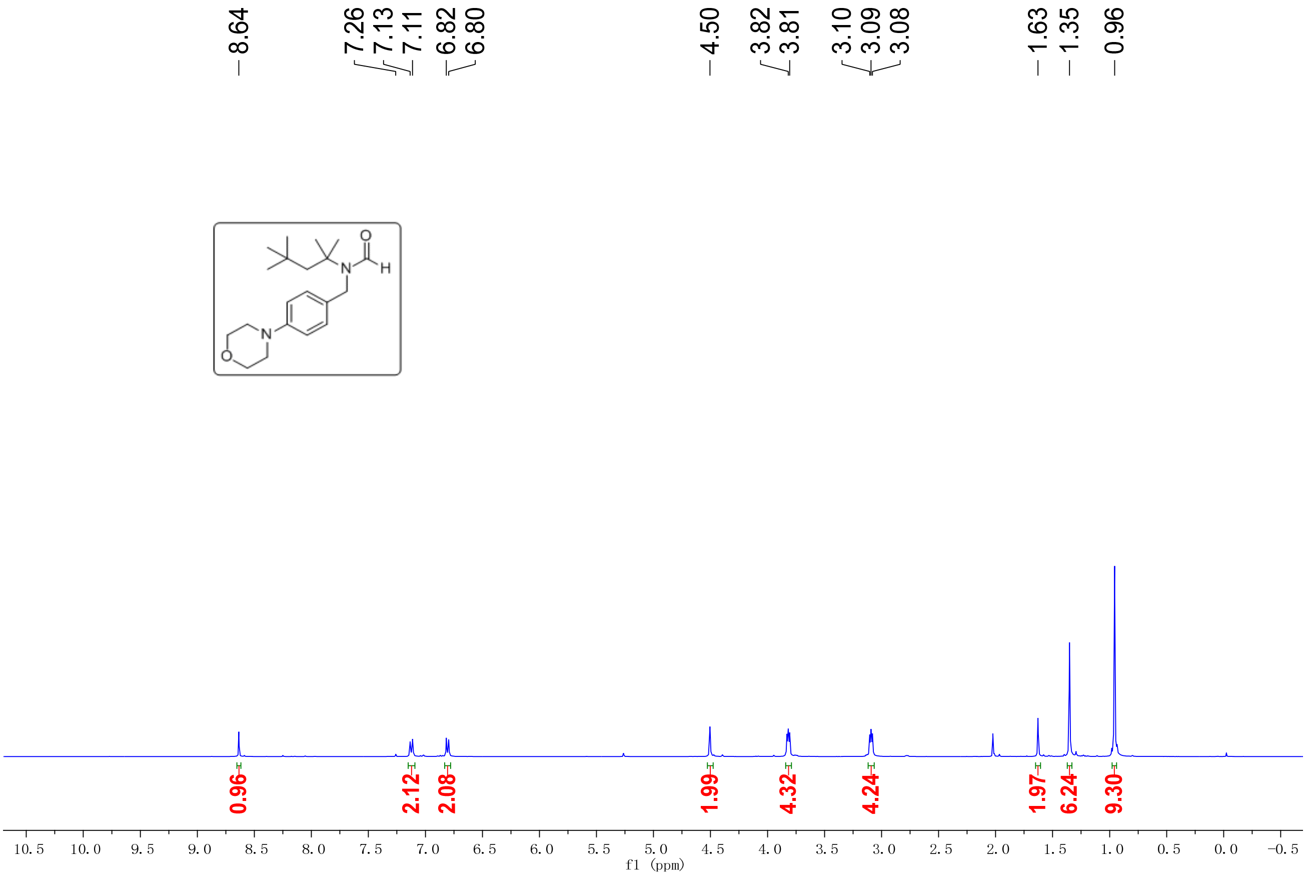

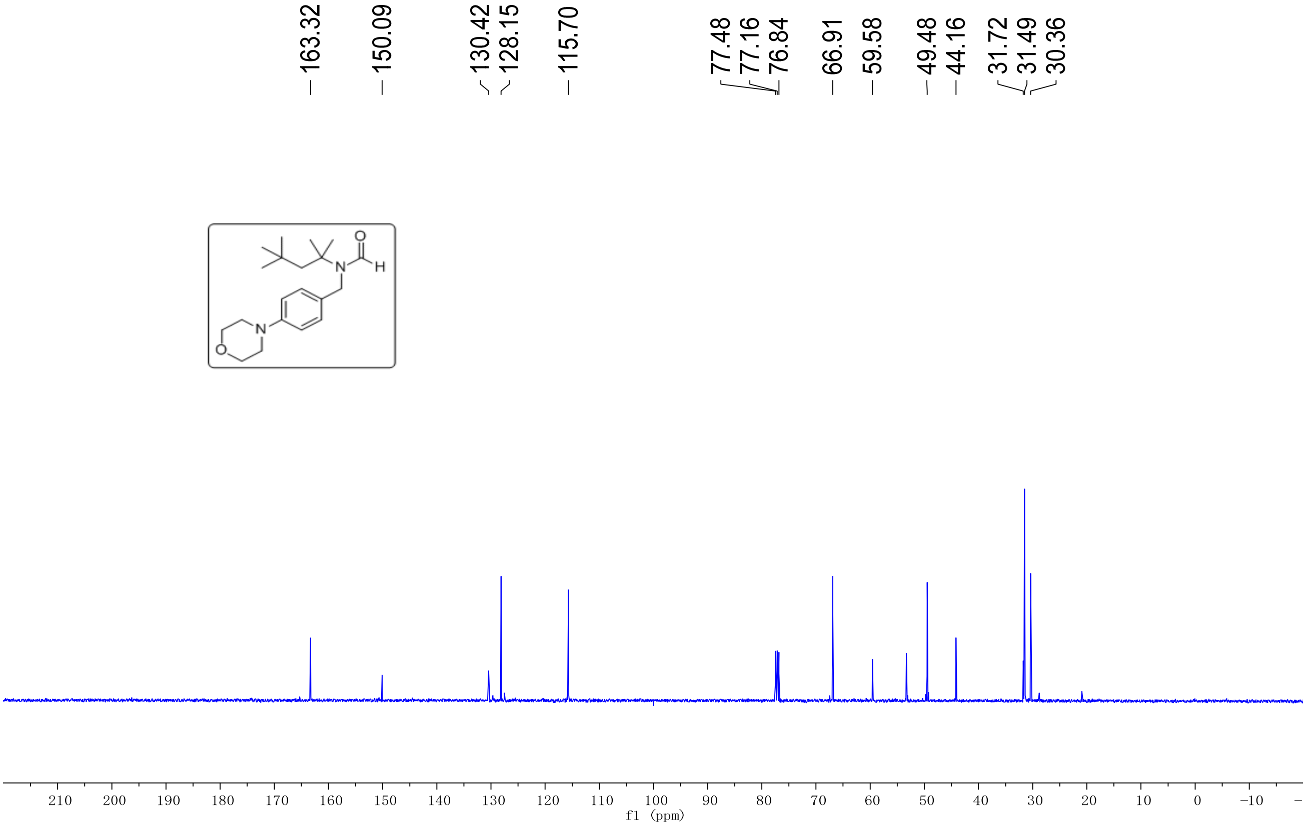

Supplementary Figure 28. ^{1}H and ^{13}C NMR spectra of compound **1k** in $CDCl_3$

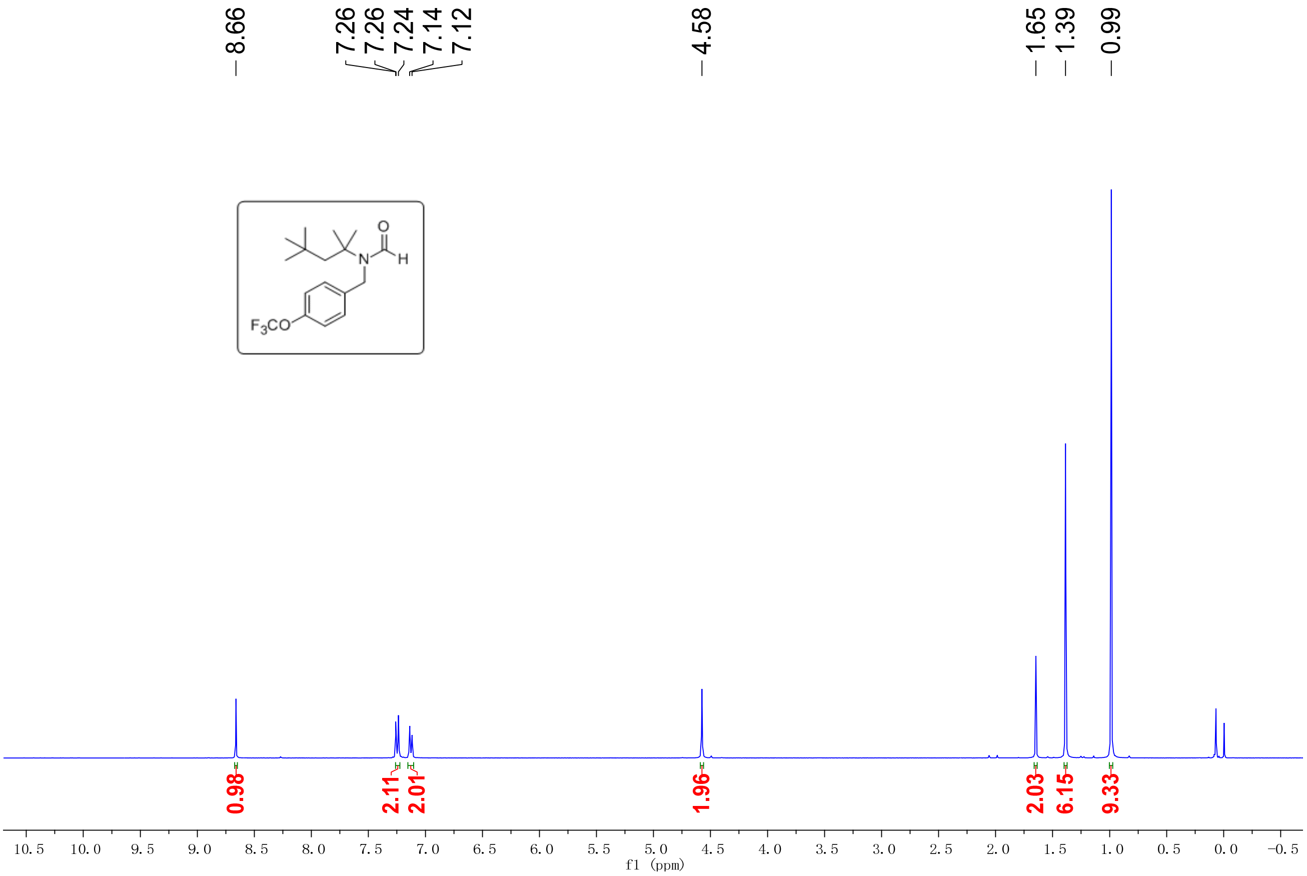

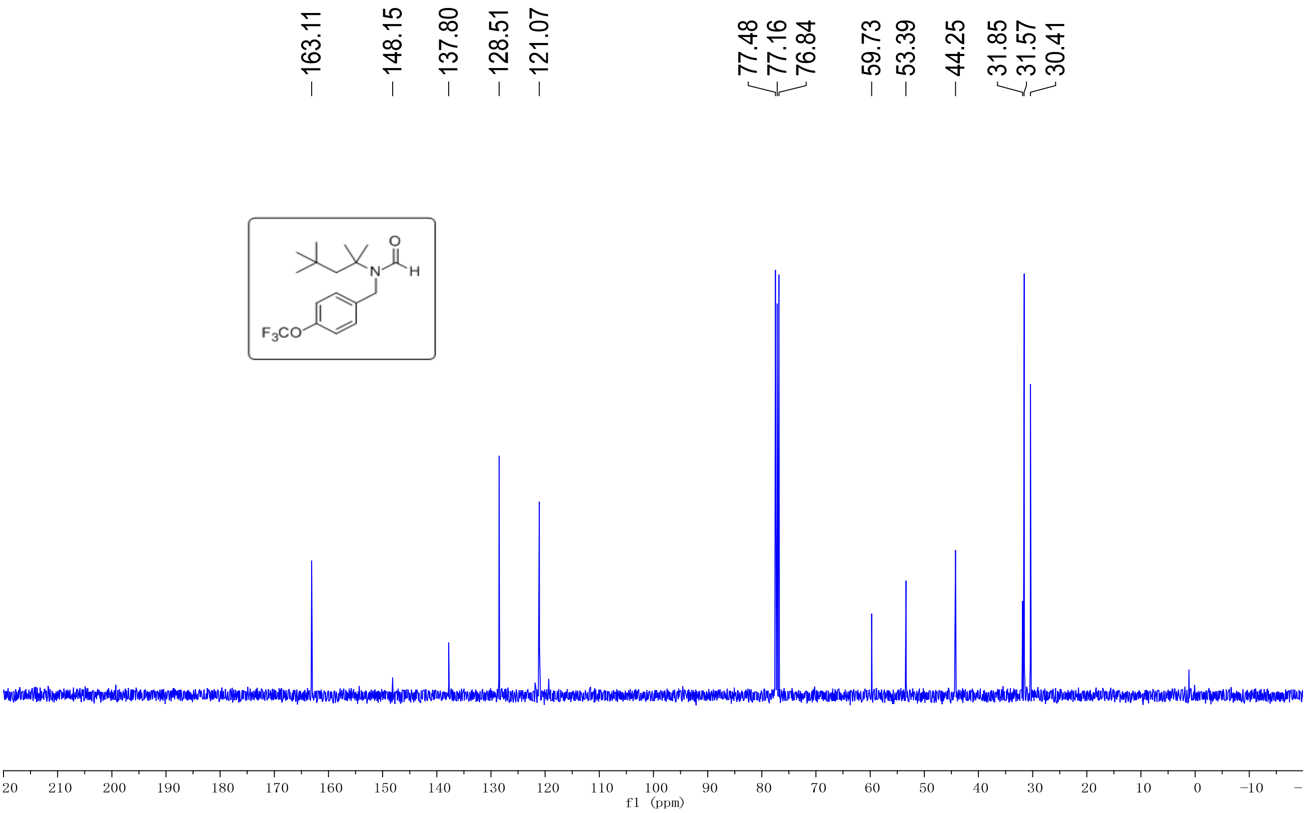

Supplementary Figure 29. ^{1}H and ^{13}C NMR spectra of compound **1l** in $CDCl_3$

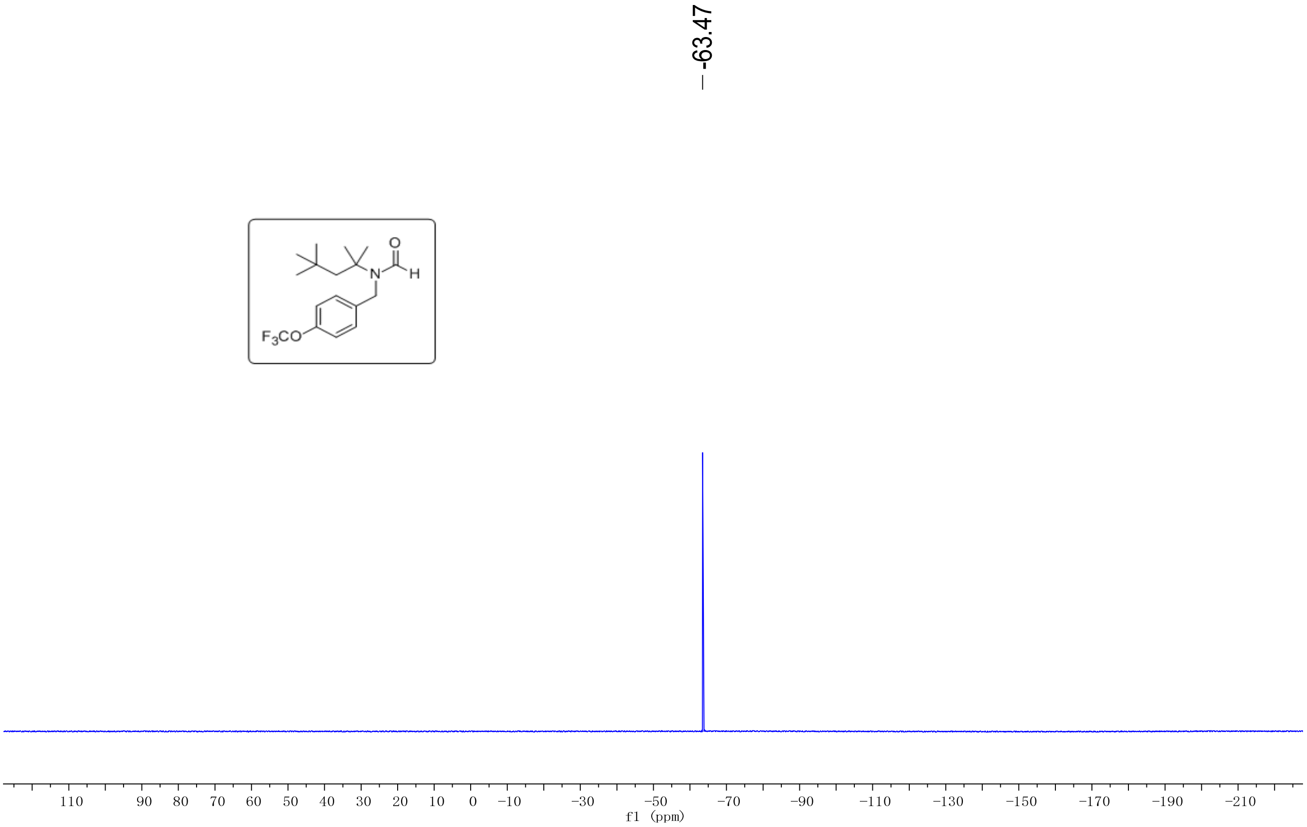

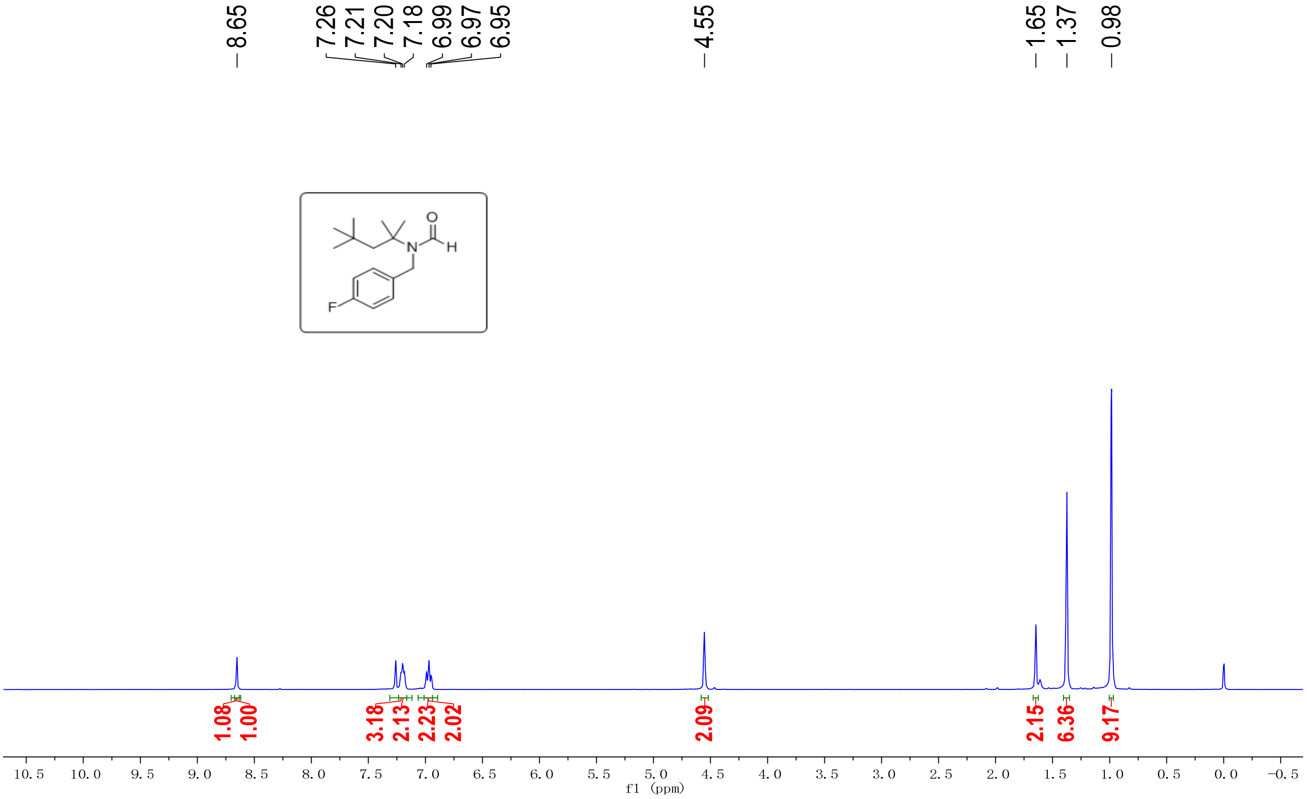

Supplementary Figure 30. ^{19}F (**1l**) and ^{1}H NMR (**1m**) spectra in $CDCl_3$

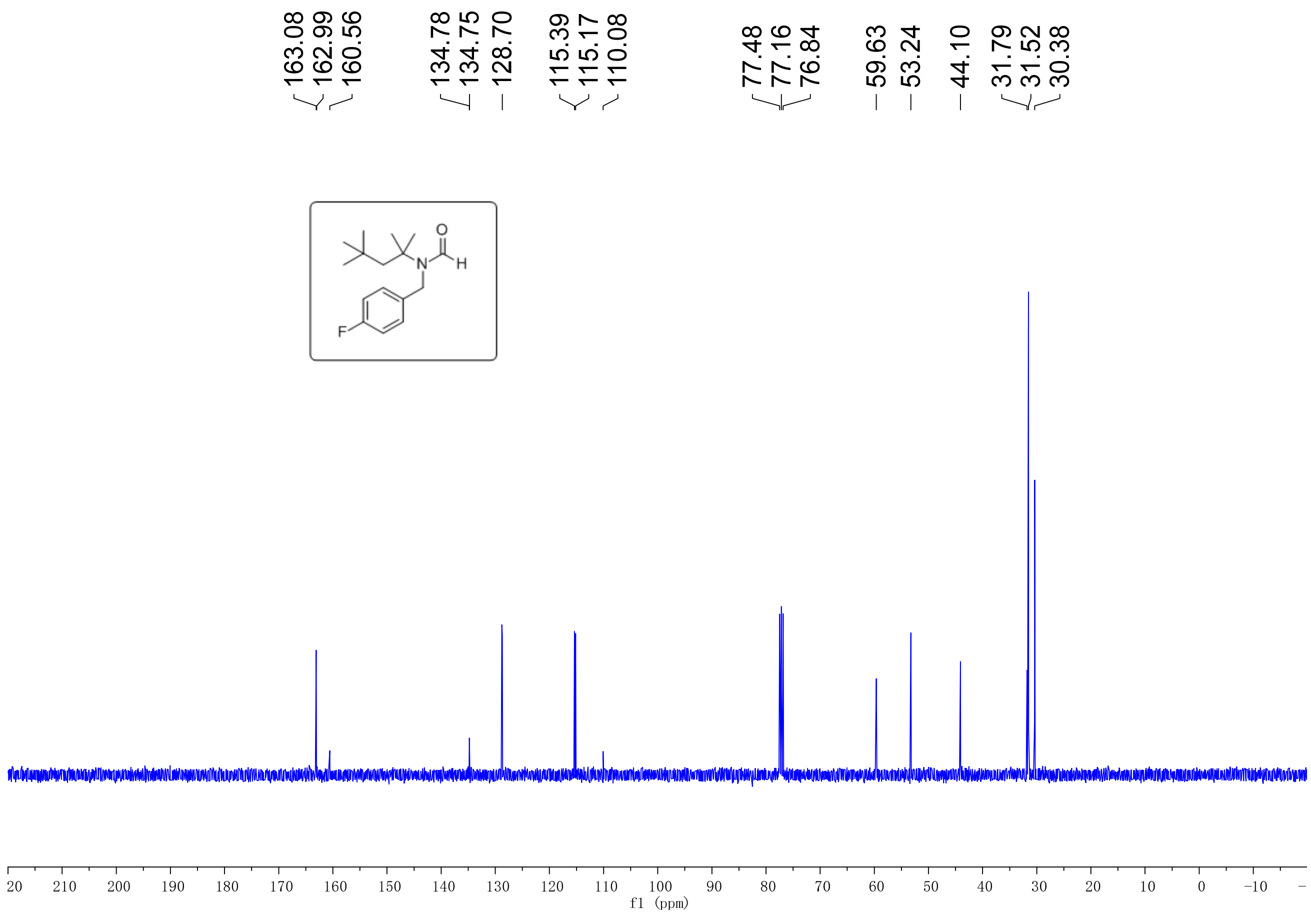

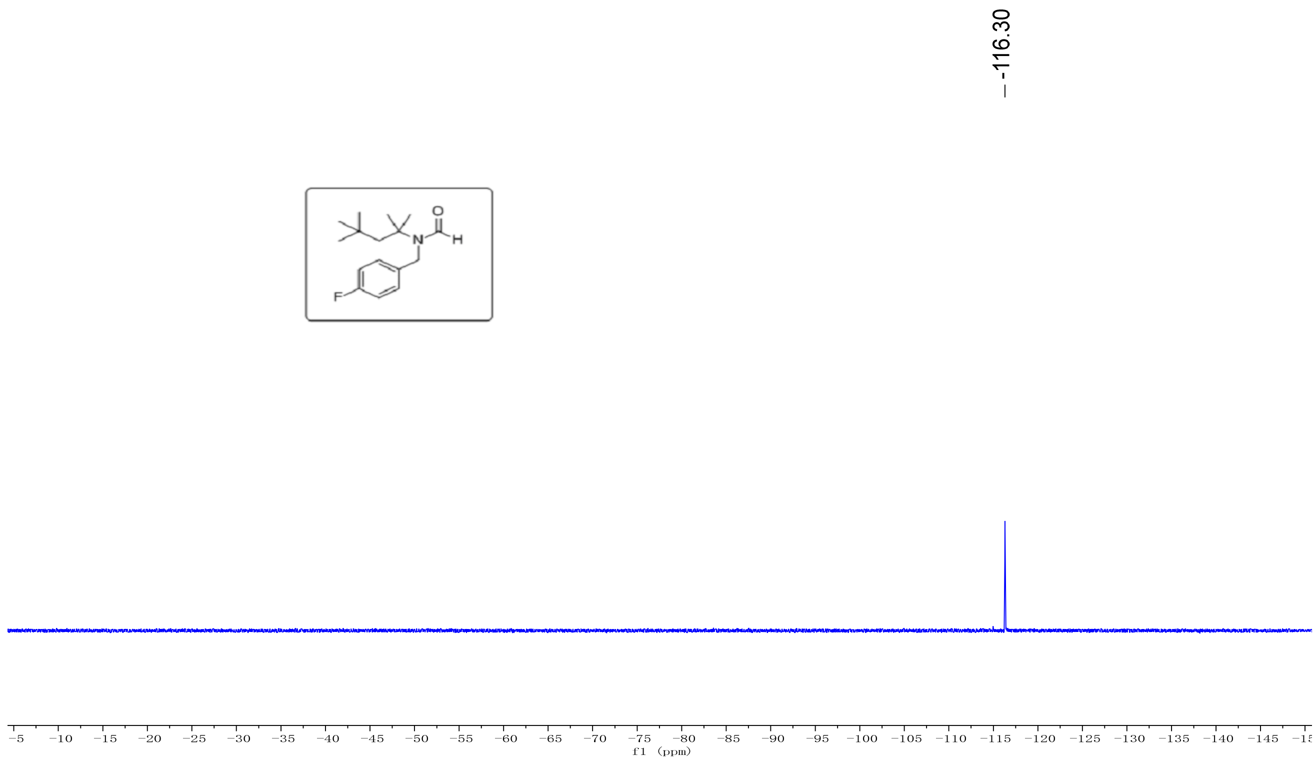

Supplementary Figure 31. ^{13}C and ^{19}F NMR spectra of compound **1m** in $CDCl_3$

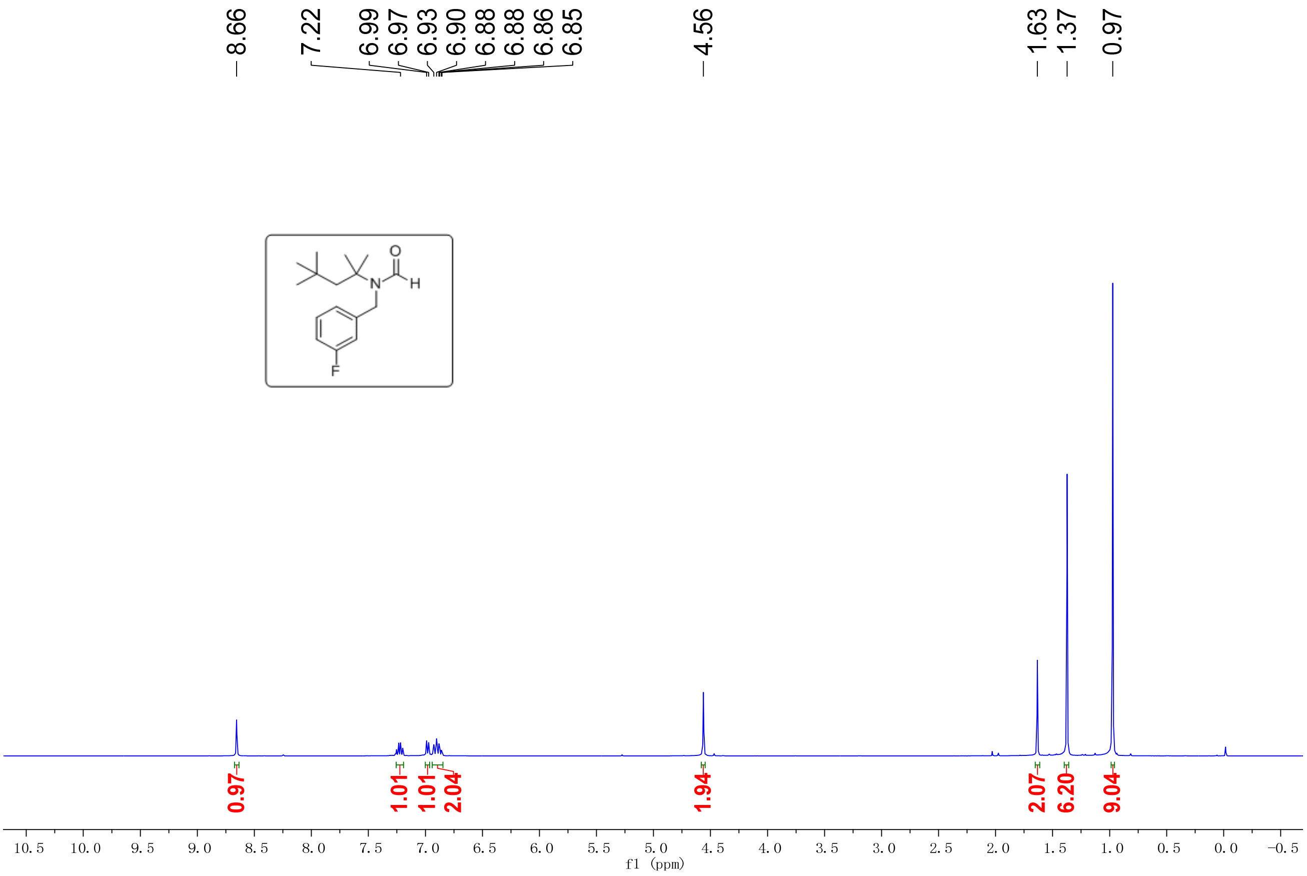

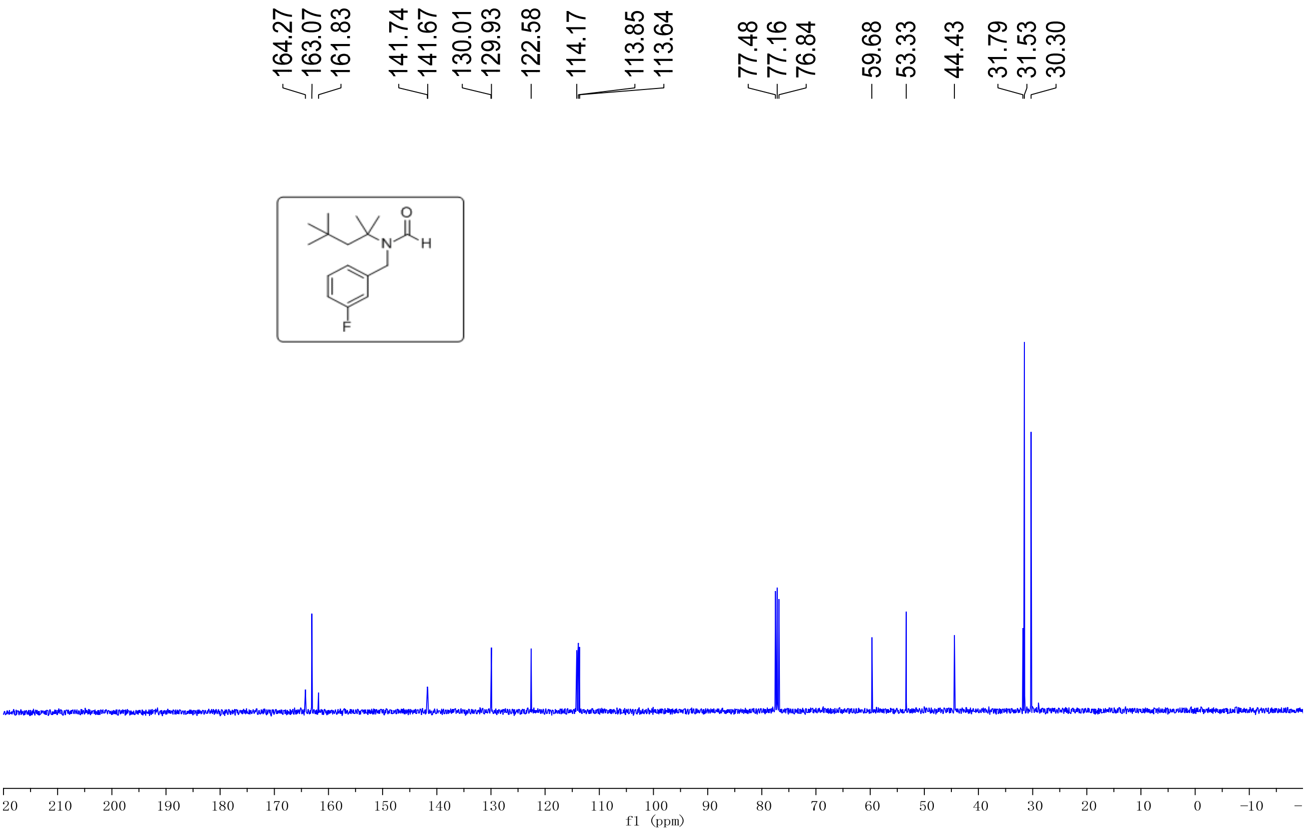

Supplementary Figure 32. ^{1}H and ^{13}C NMR spectra of compound **1n** in $CDCl_3$

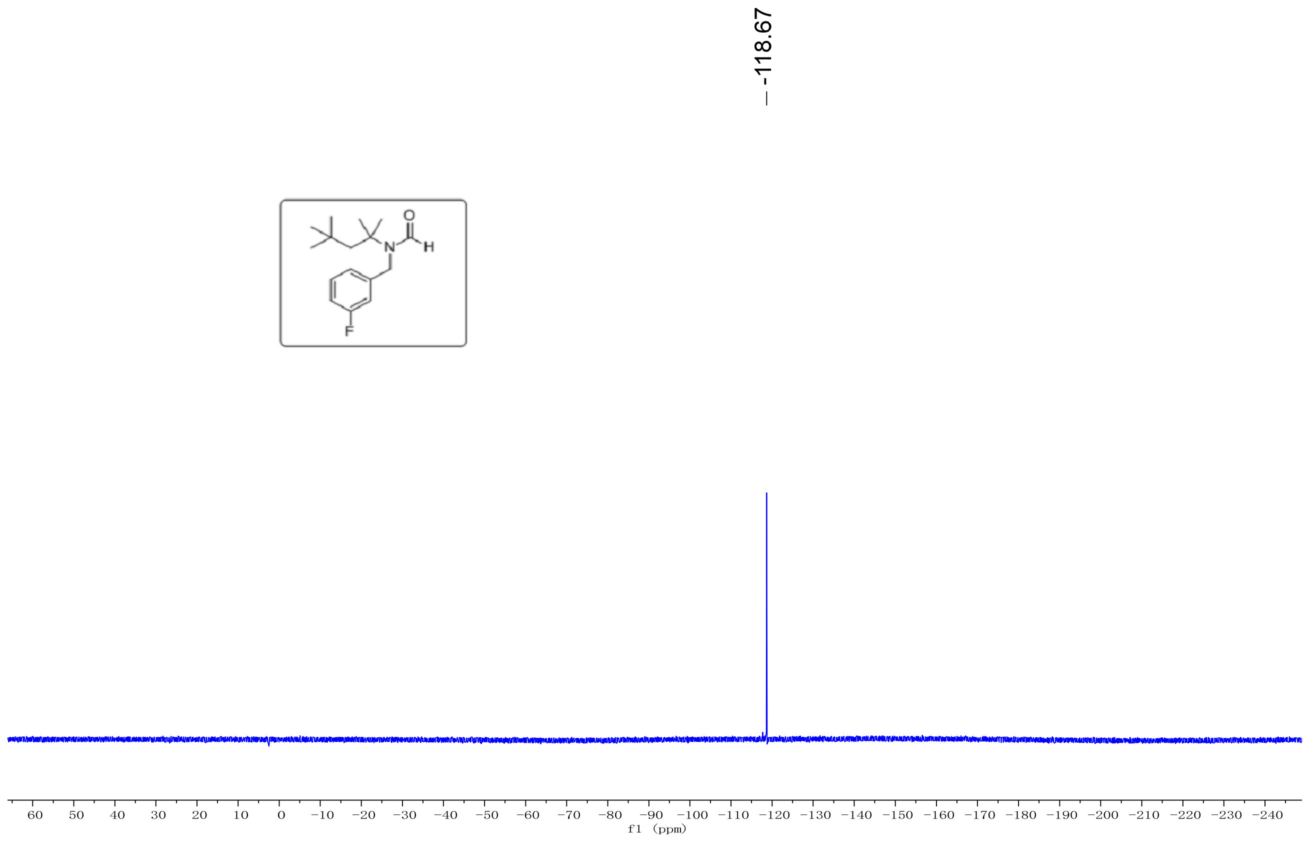

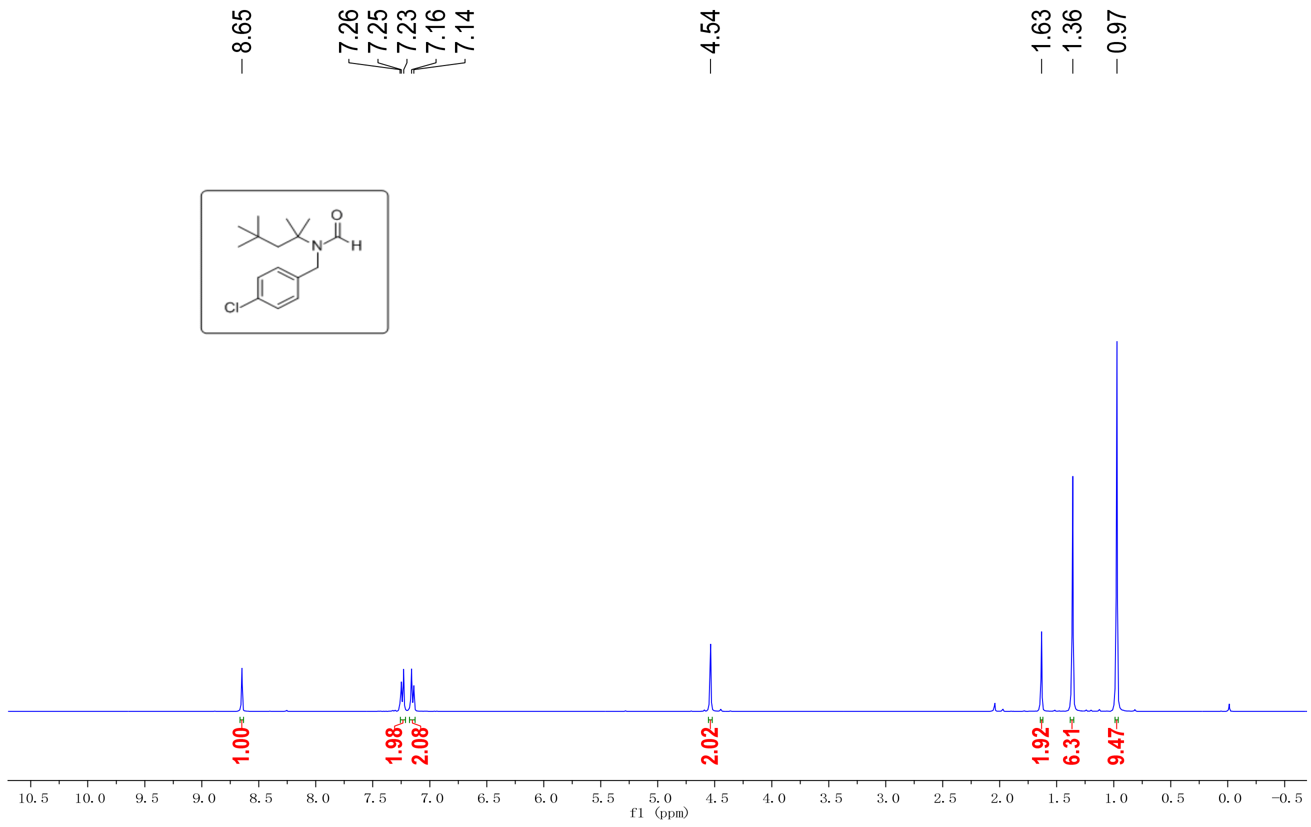

Supplementary Figure 33. ^{19}F (**1n**) and ^{1}H (**1o**) NMR spectra in $CDCl_3$

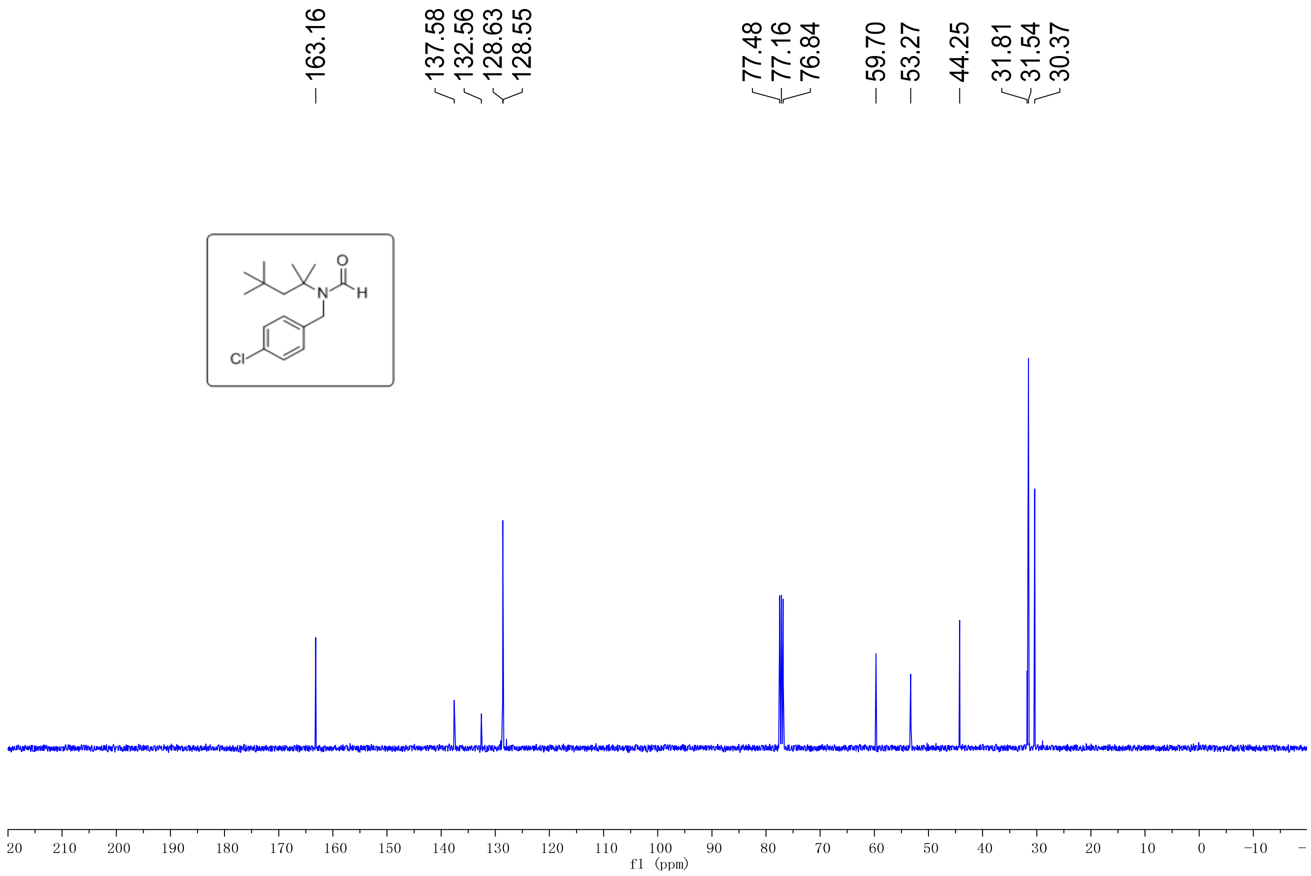

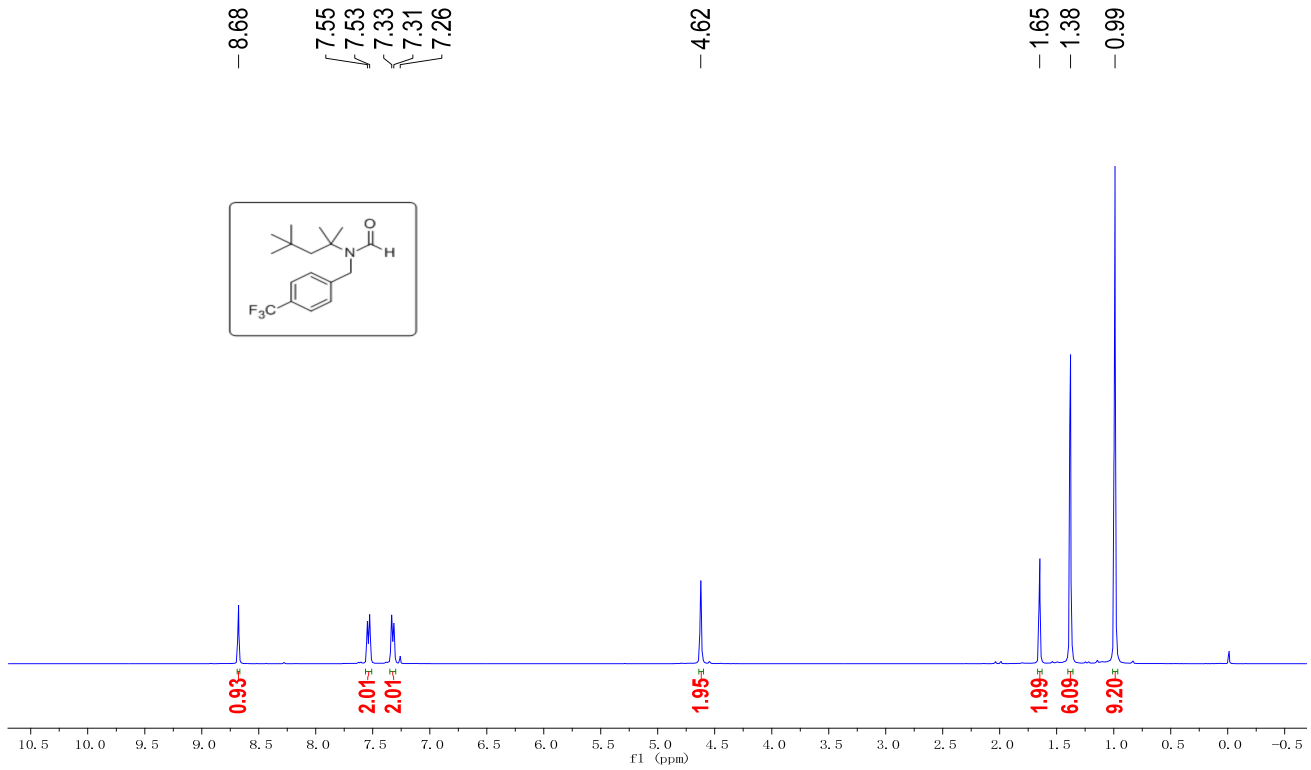

Supplementary Figure 34. ^{13}C (**1o**) and ^{1}H NMR(**1p**) spectra in $CDCl_3$

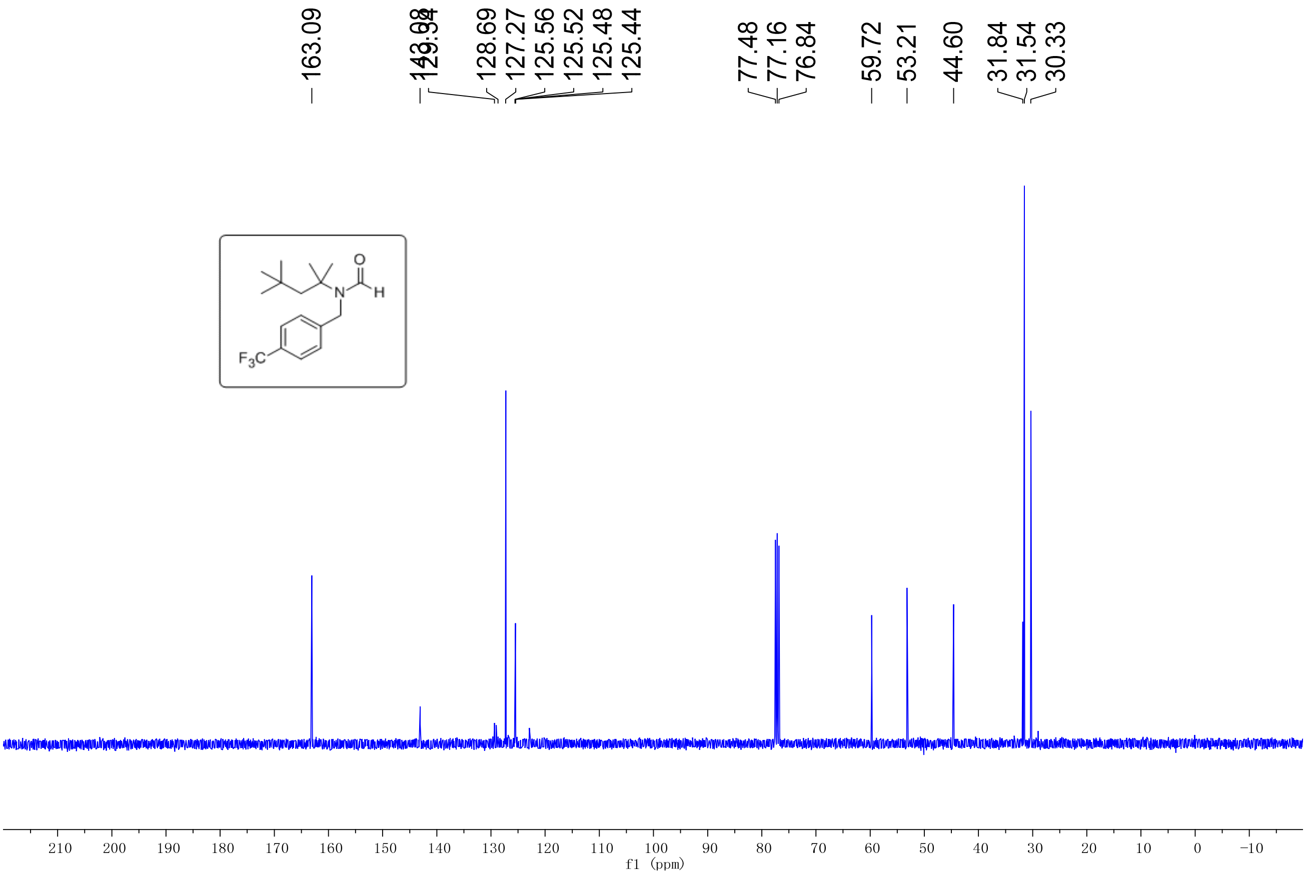

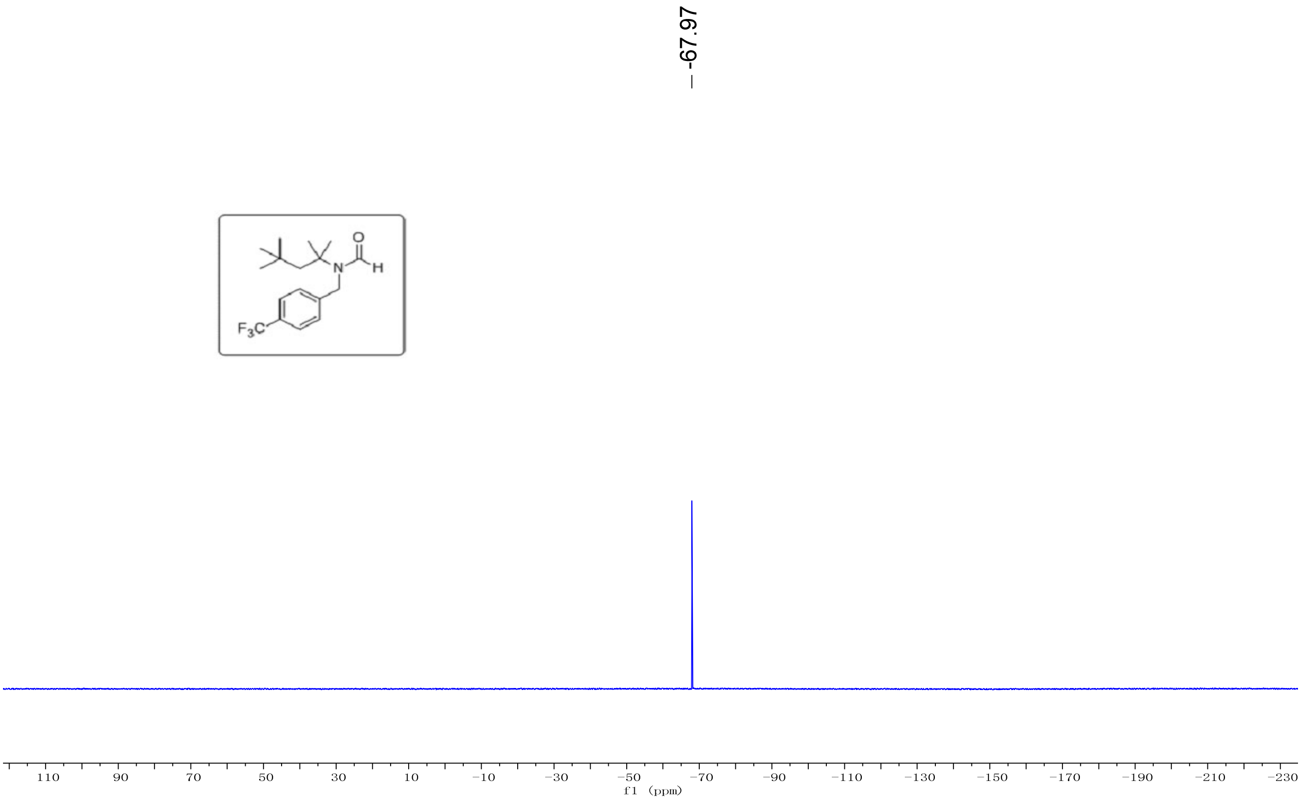

Supplementary Figure 35. ^{13}C and ^{19}F NMR spectra of compound **1p** in $CDCl_3$

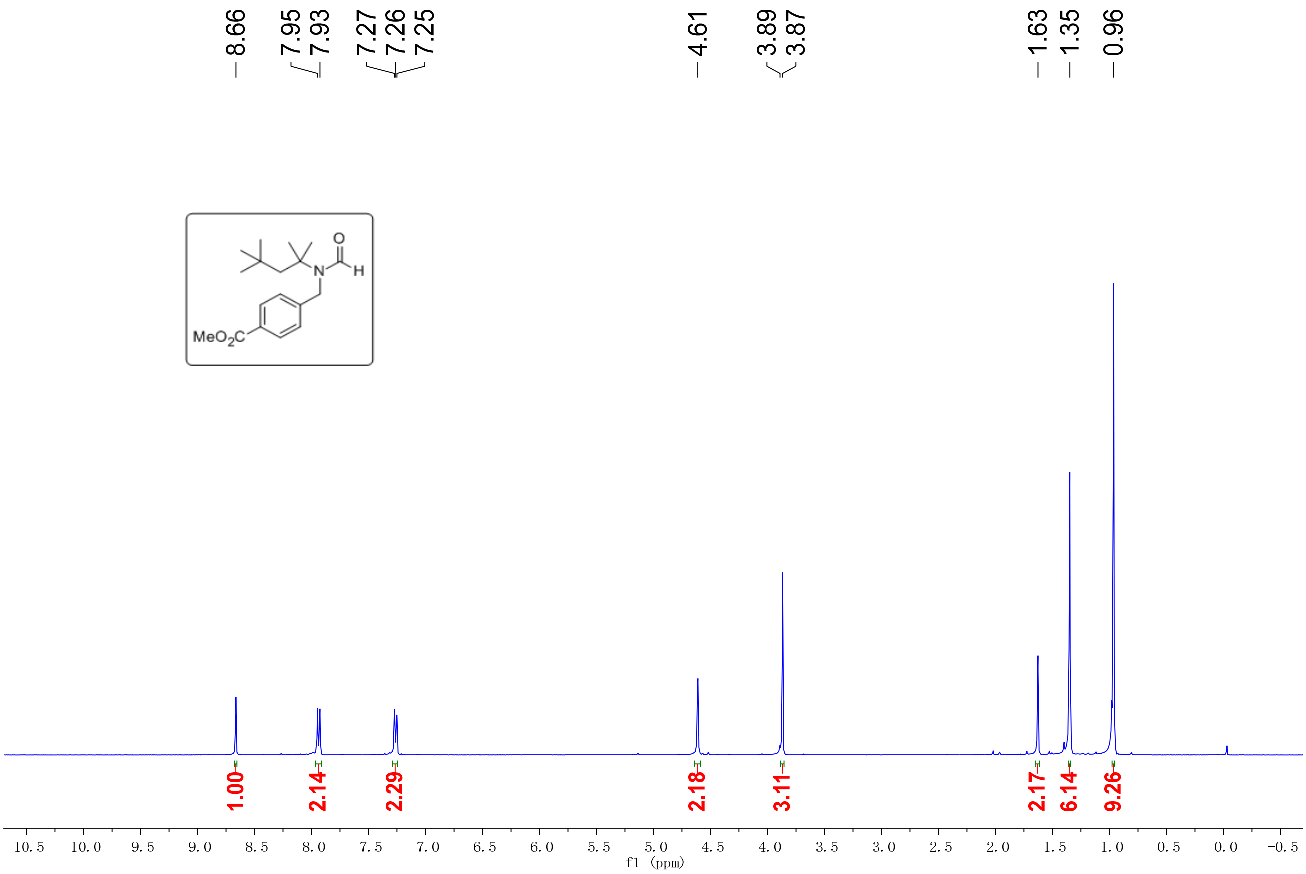

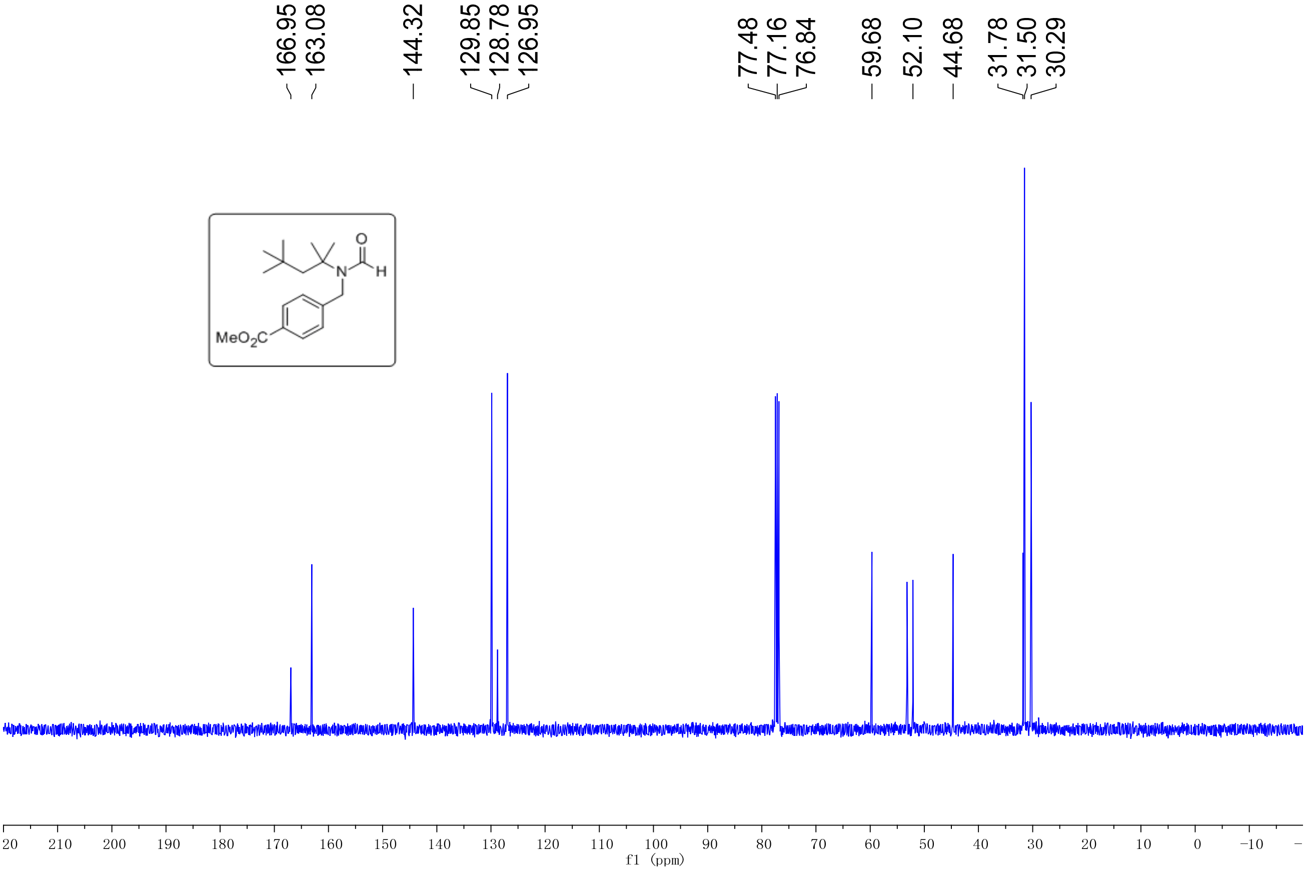

Supplementary Figure 36. ^{1}H and ^{13}C NMR spectra of compound **1q** in $CDCl_3$

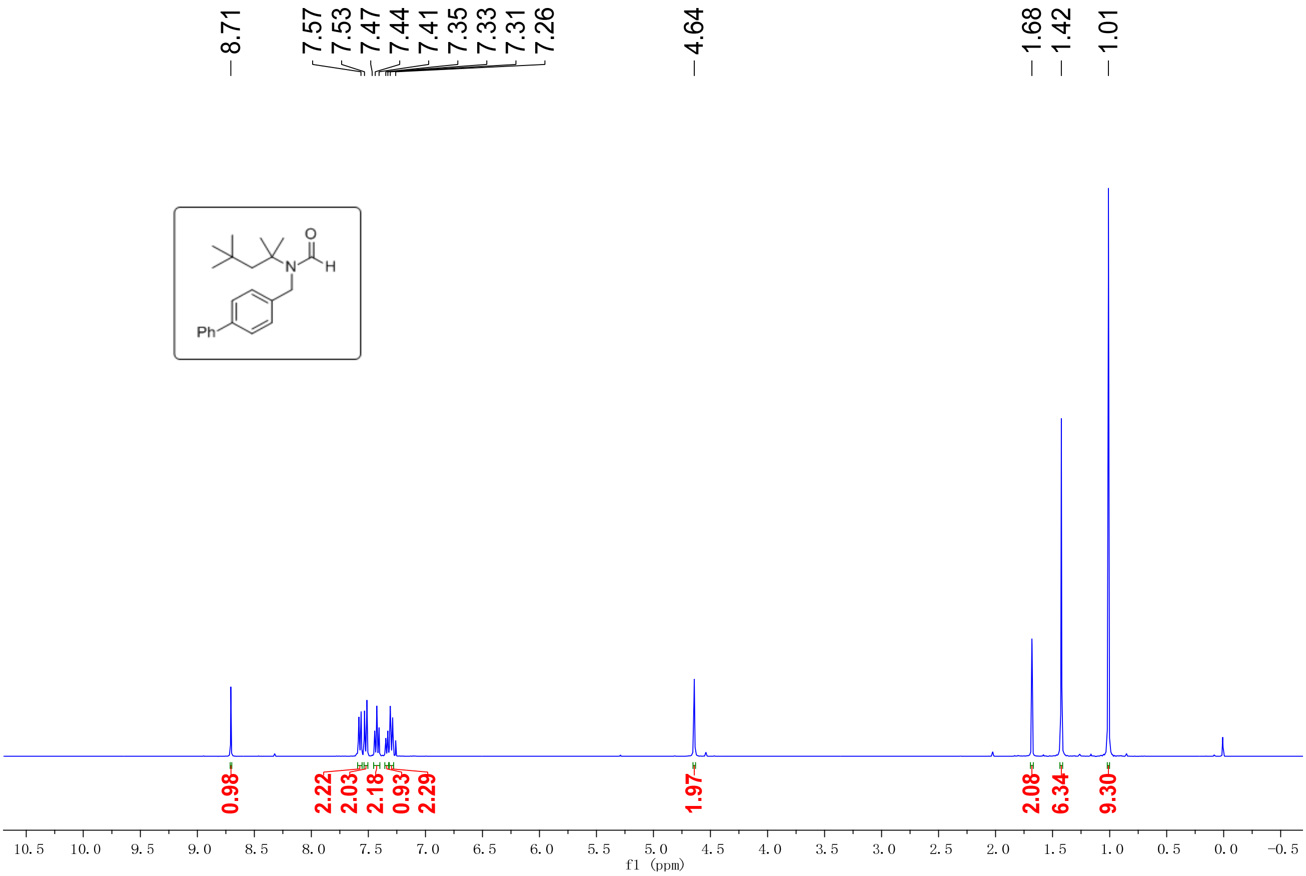

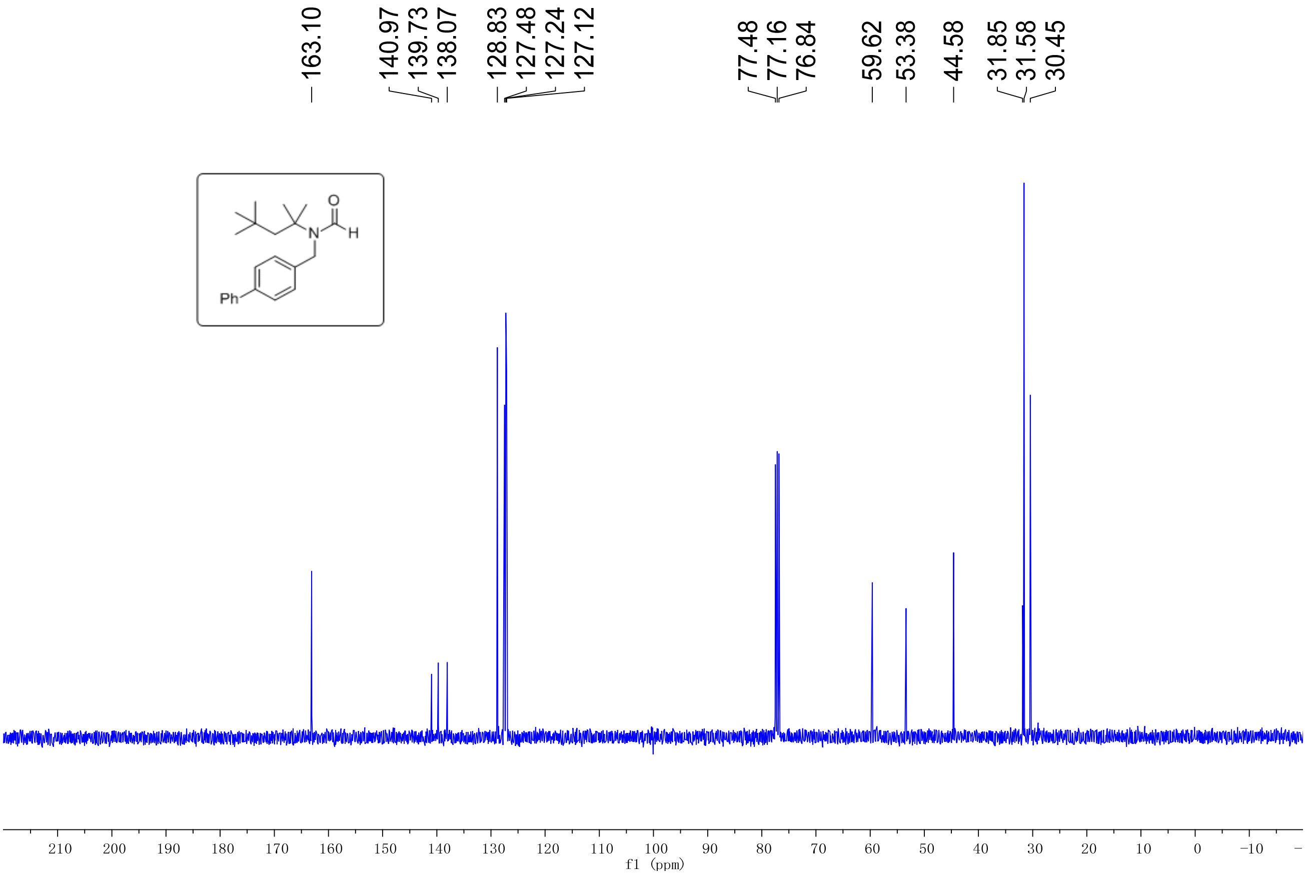

Supplementary Figure 37. ^{1}H and ^{13}C NMR spectra of compound **1r** in $CDCl_3$

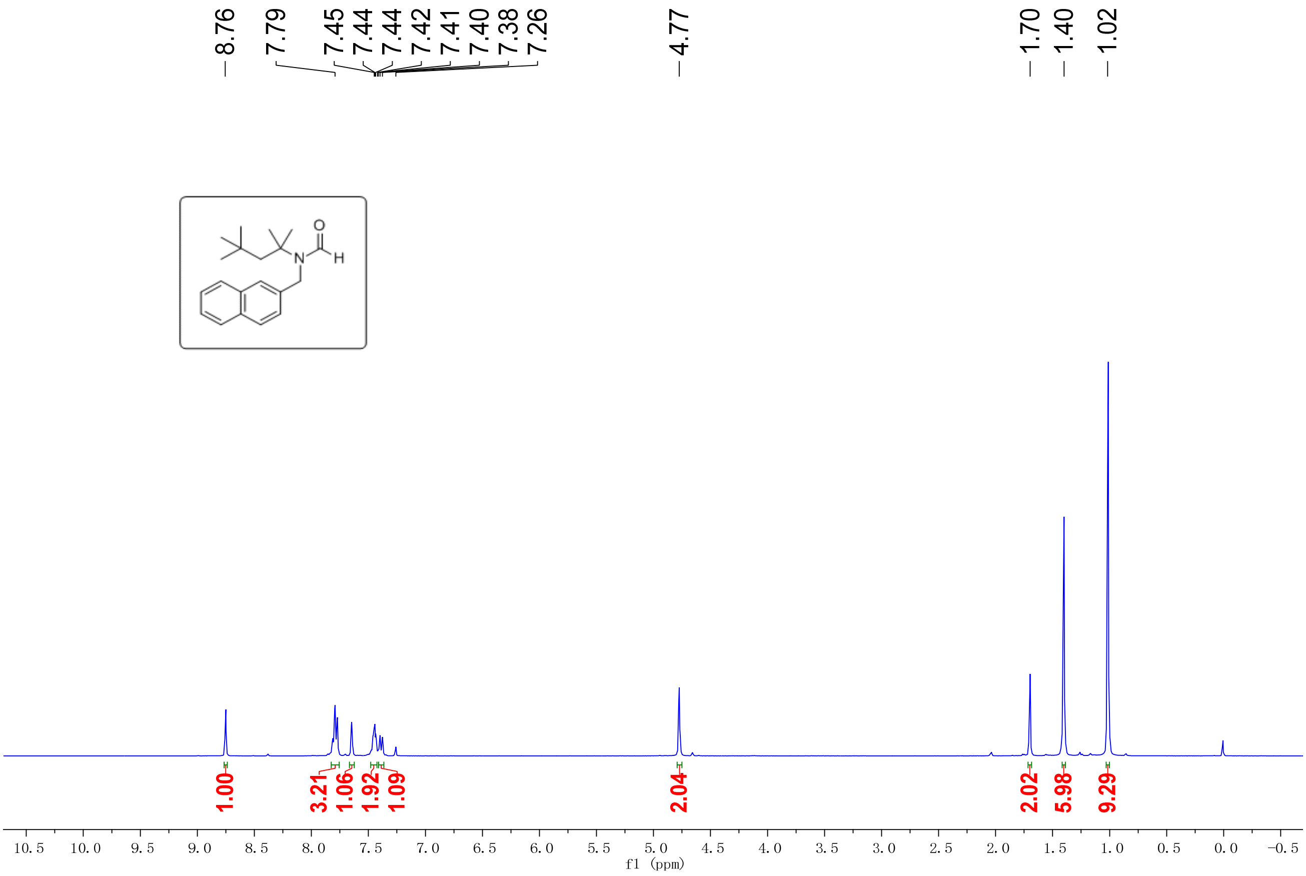

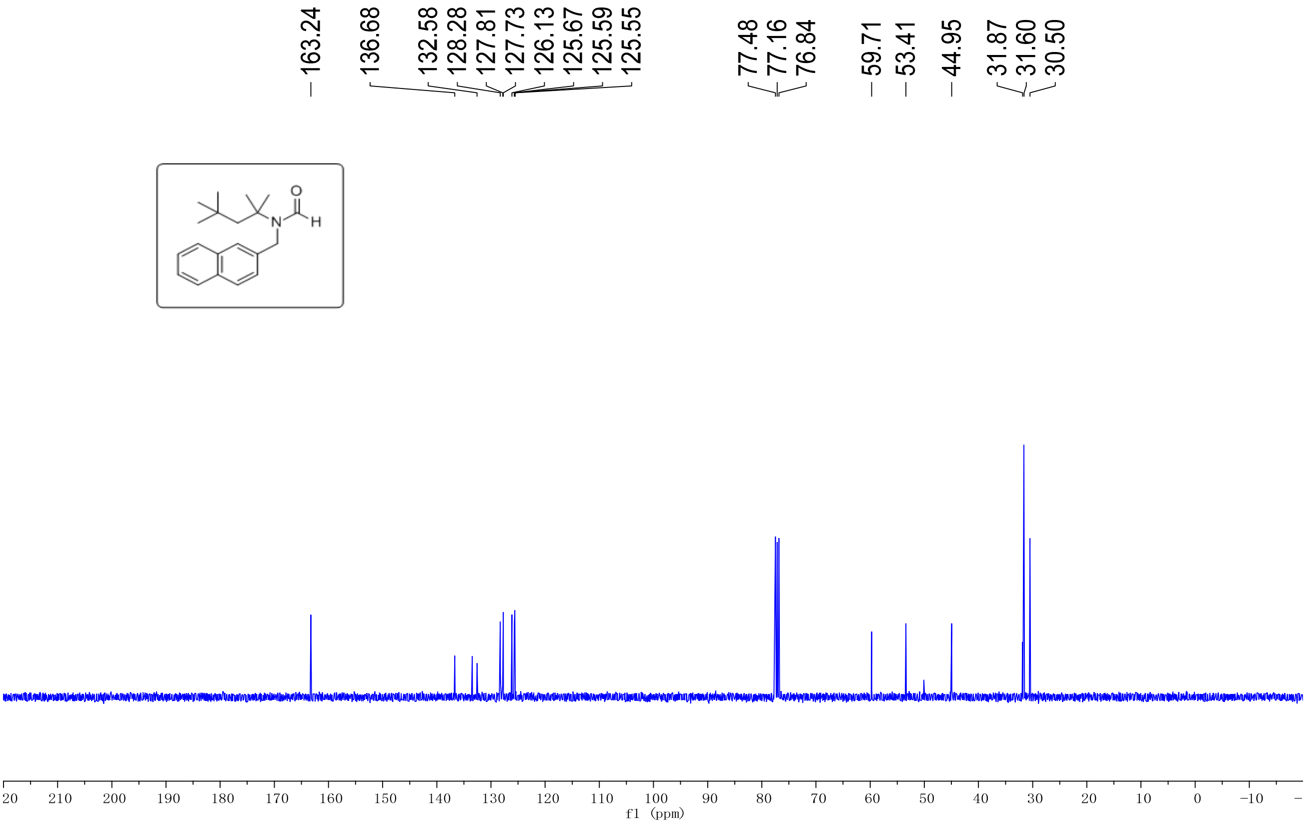

Supplementary Figure 38. ^{1}H and ^{13}C NMR spectra of compound **1s** in $CDCl_3$

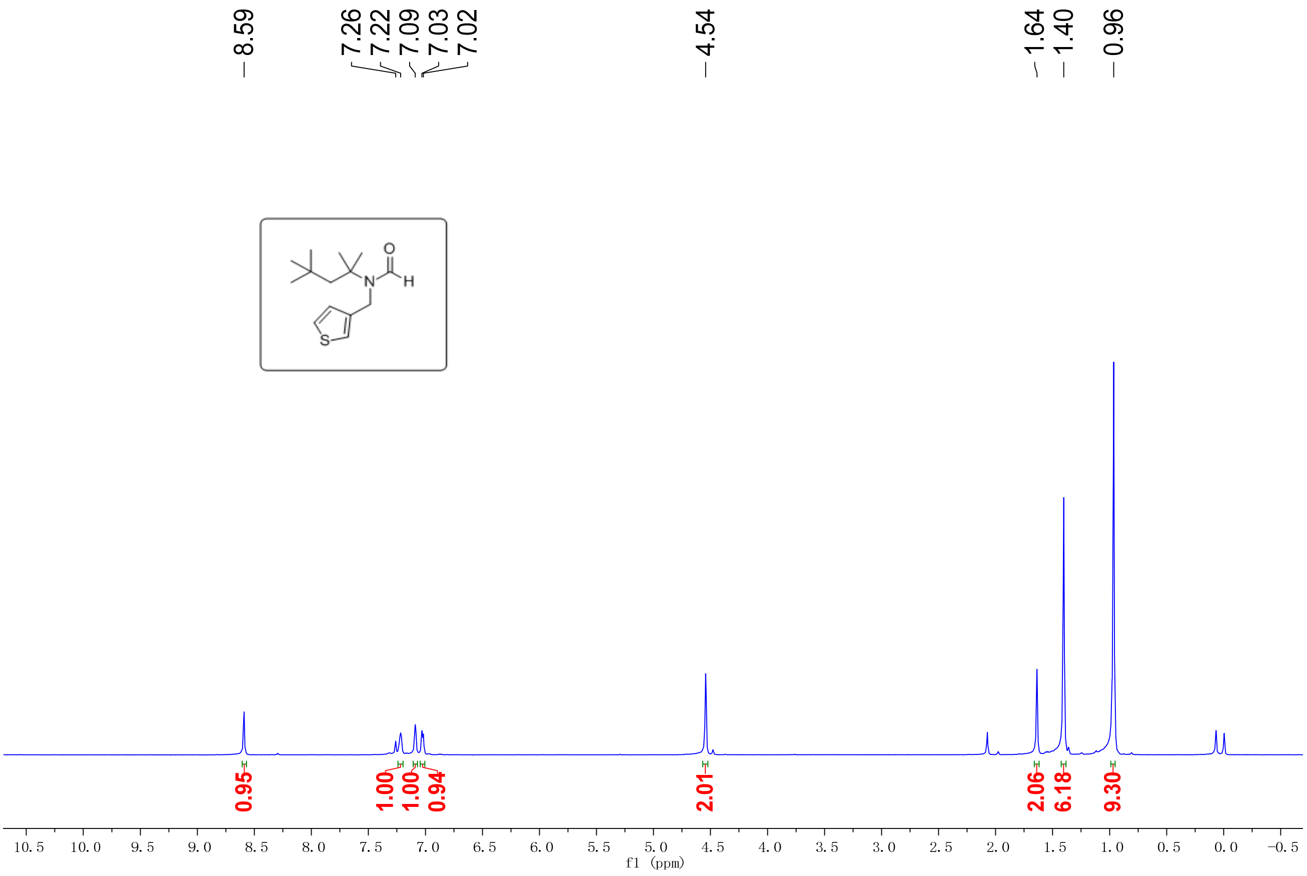

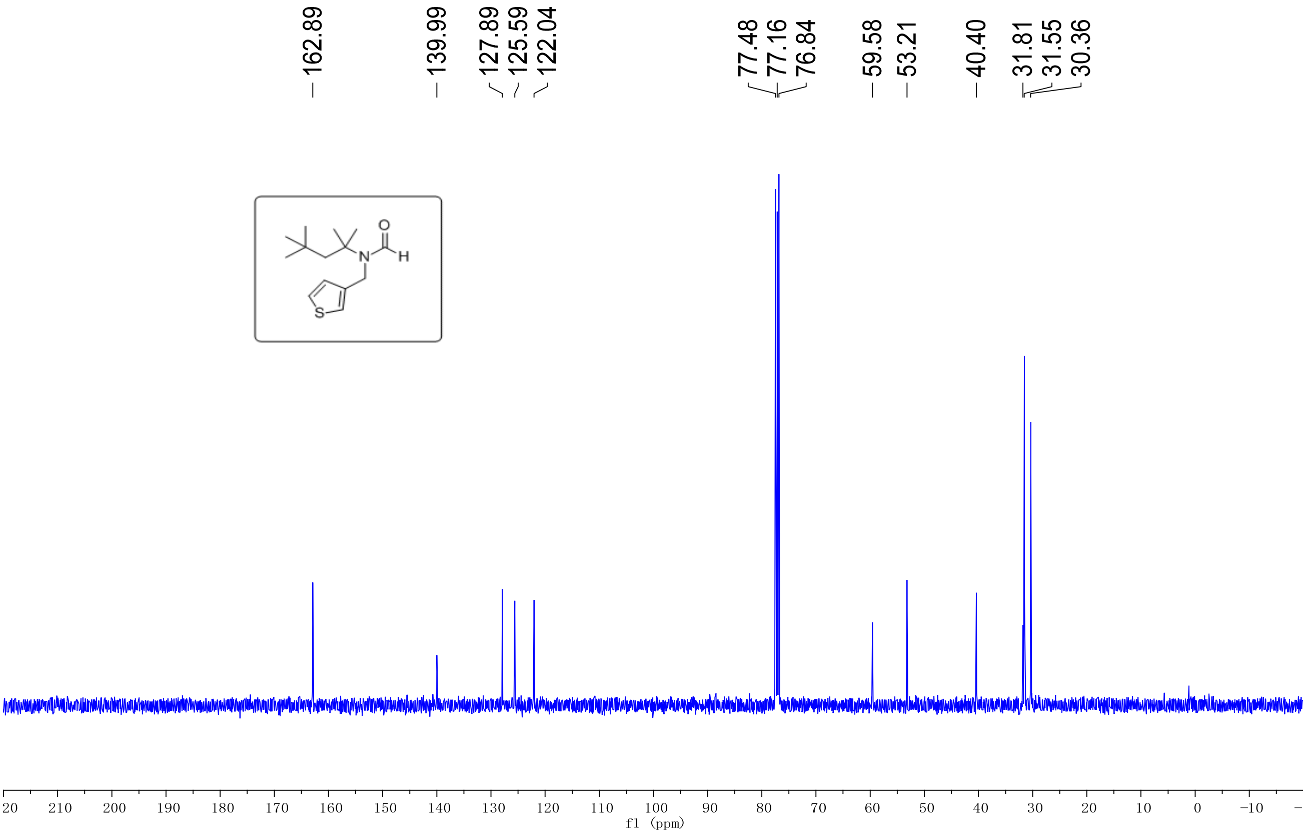

Supplementary Figure 39. ^{1}H and ^{13}C NMR spectra of compound **1t** in $CDCl_3$

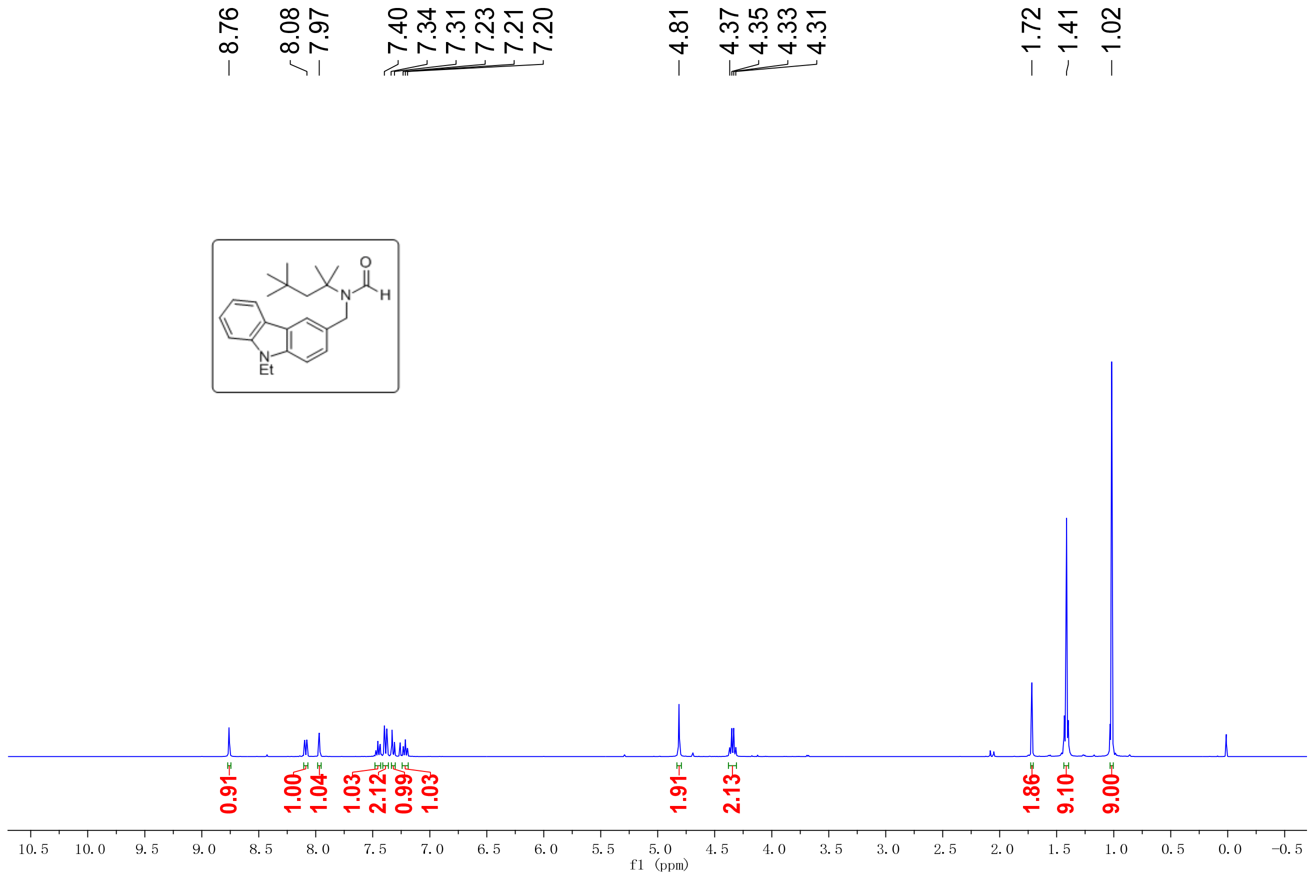

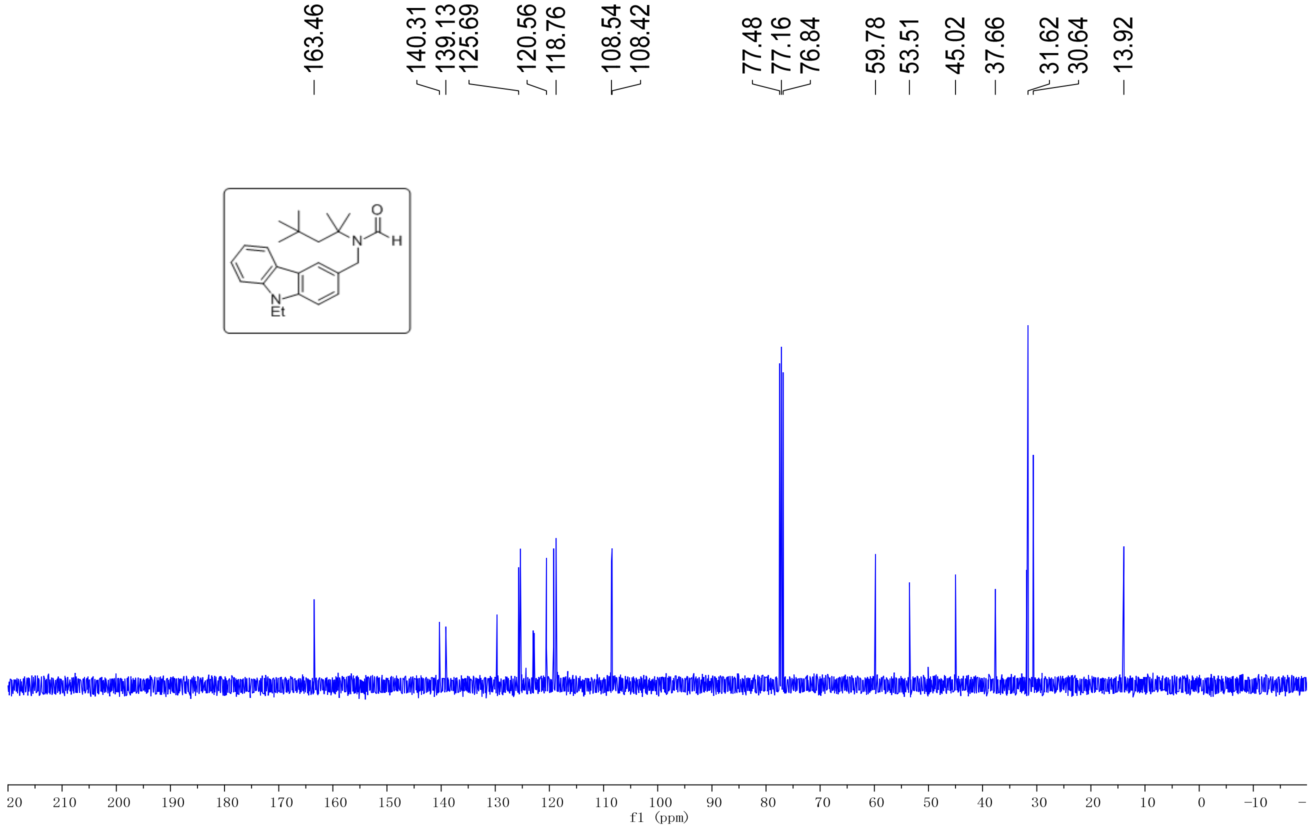

Supplementary Figure 40. ^{1}H and ^{13}C NMR spectra of compound **1u** in $CDCl_3$

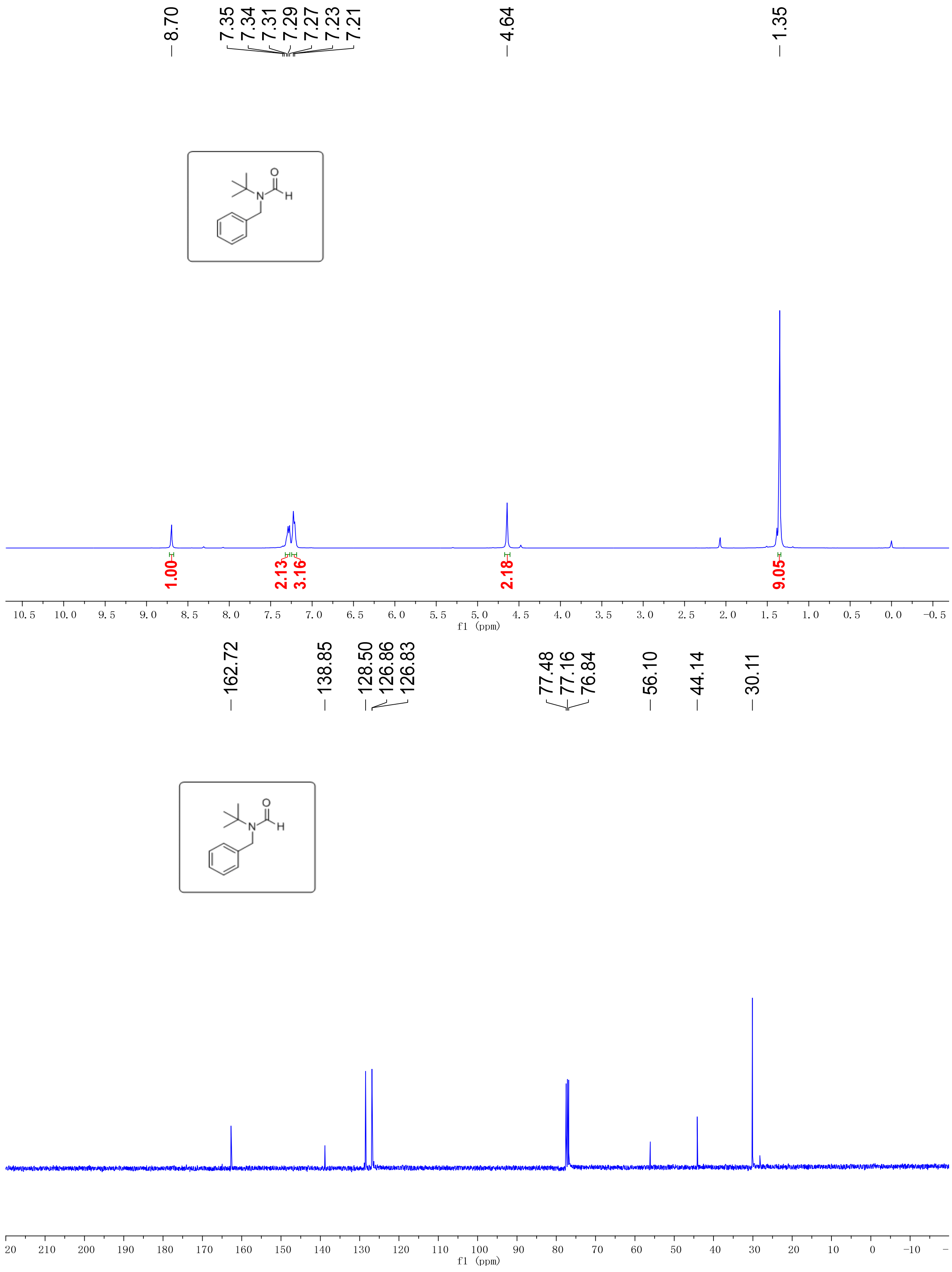

Supplementary Figure 41. ^{1}H and ^{13}C NMR spectra of compound **1v** in $CDCl_3$

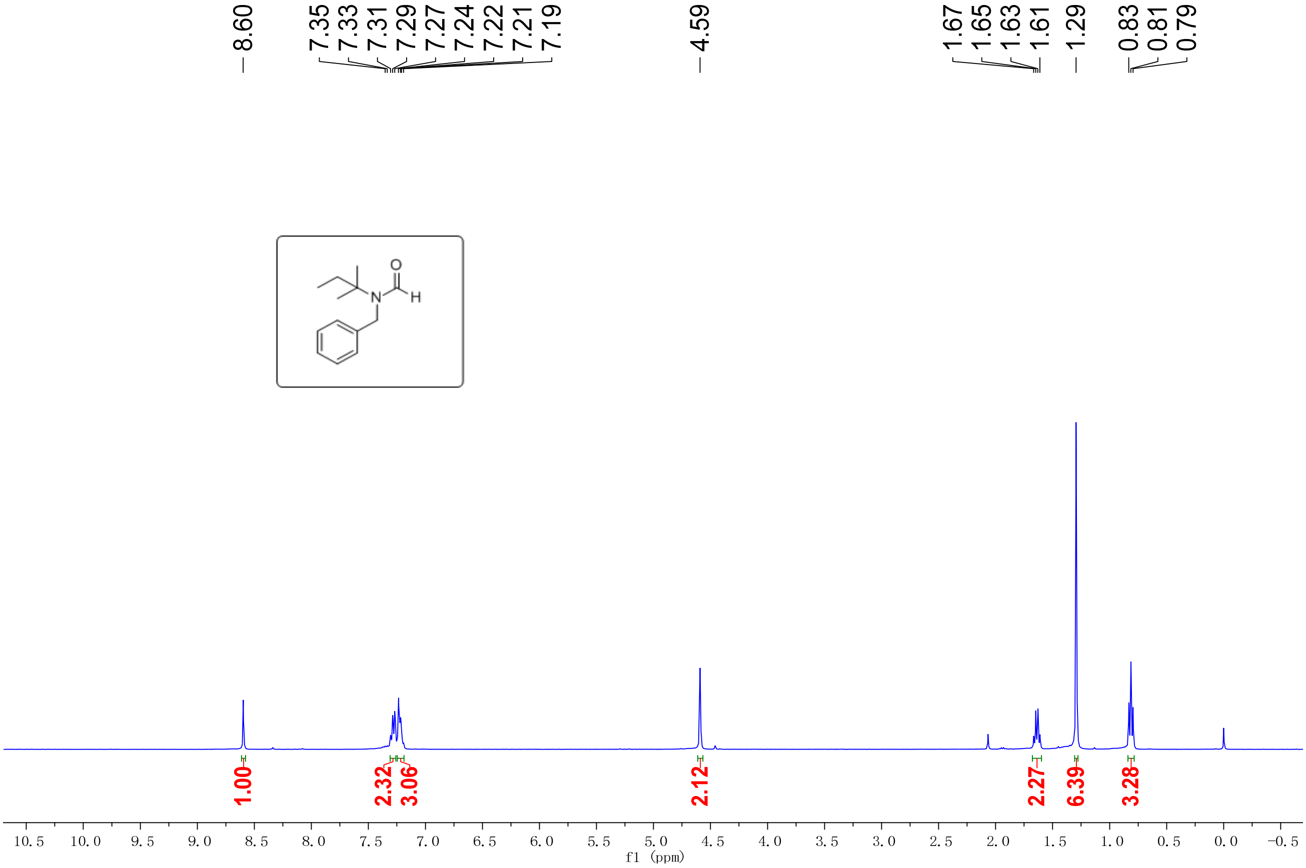

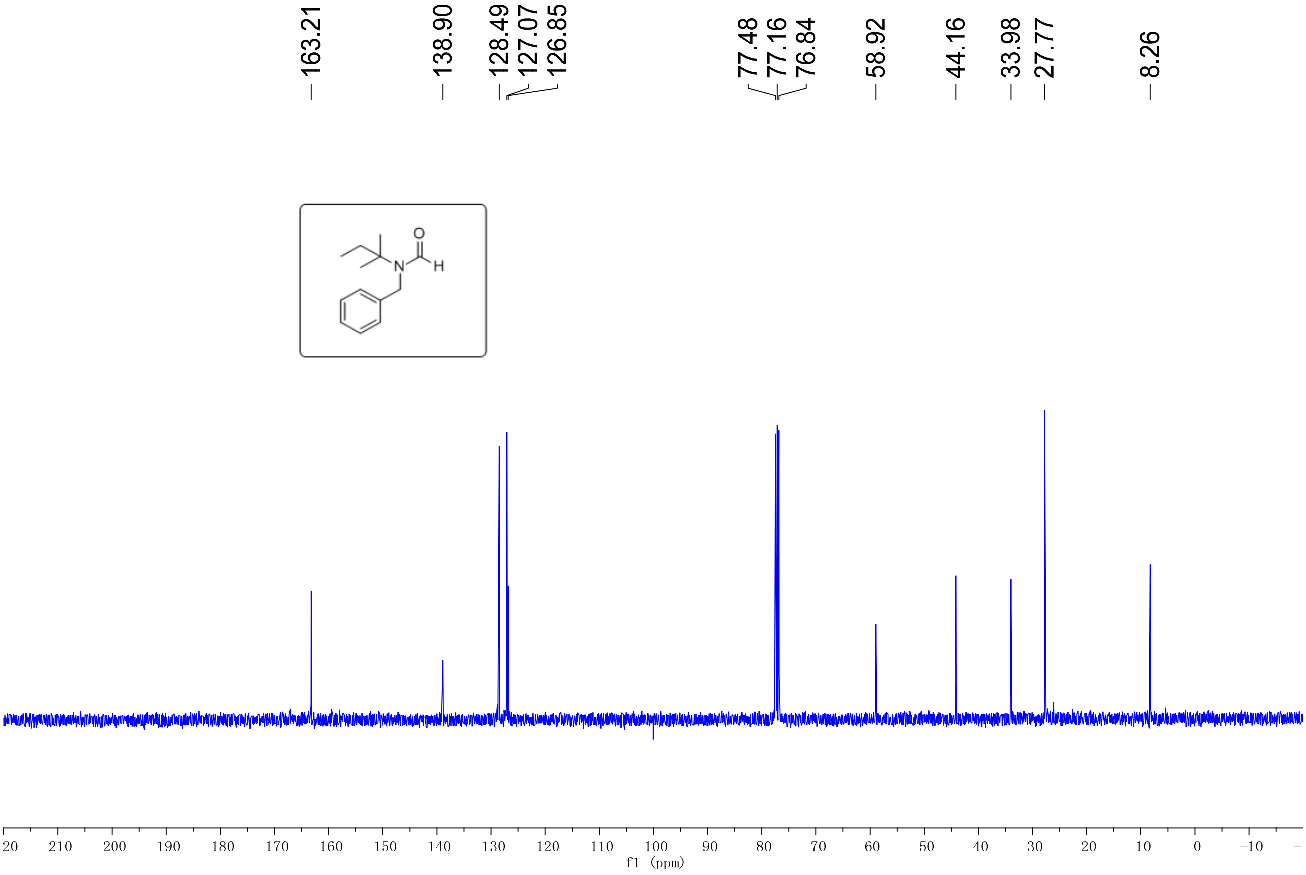

Supplementary Figure 42. ^{1}H and ^{13}C NMR spectra of compound **1w** in $CDCl_3$

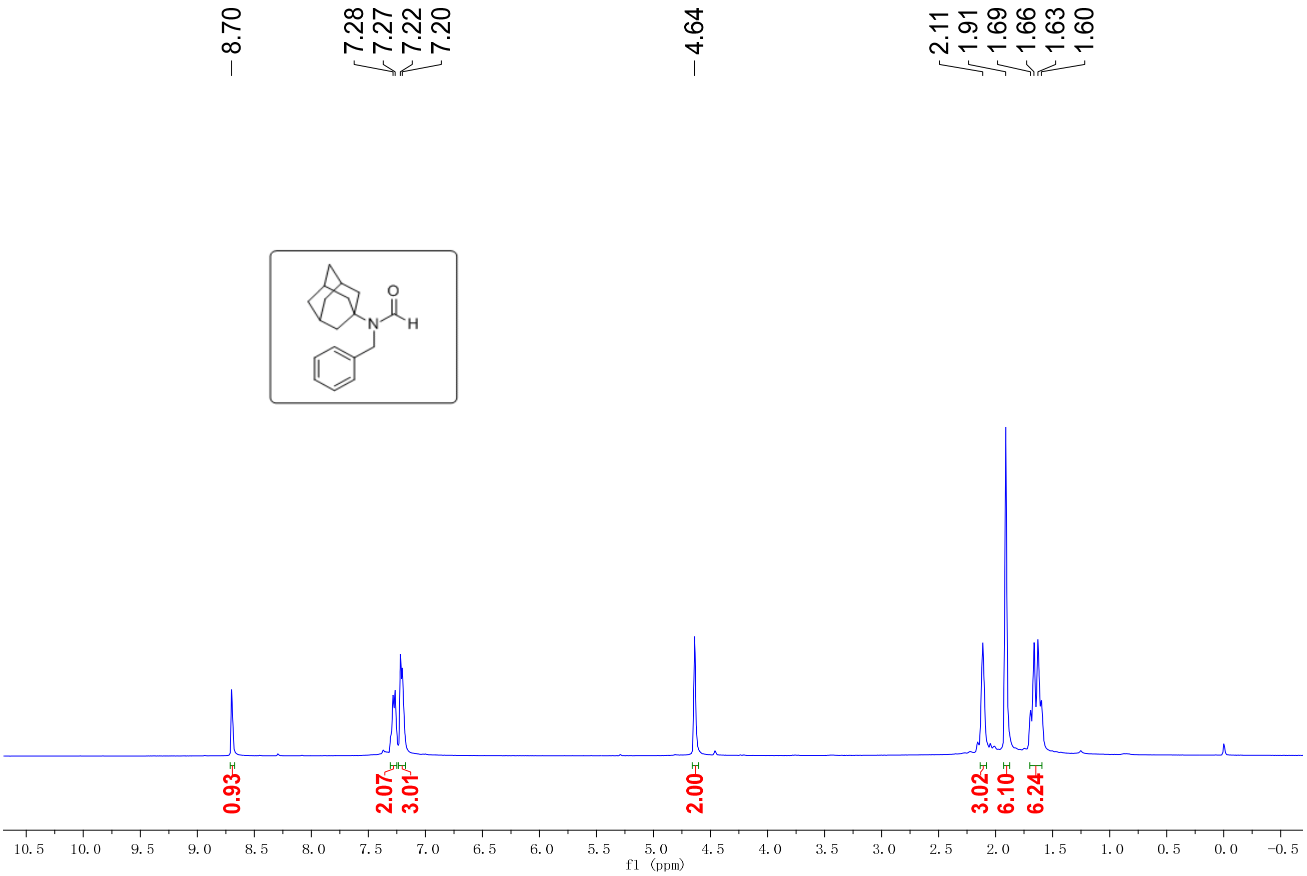

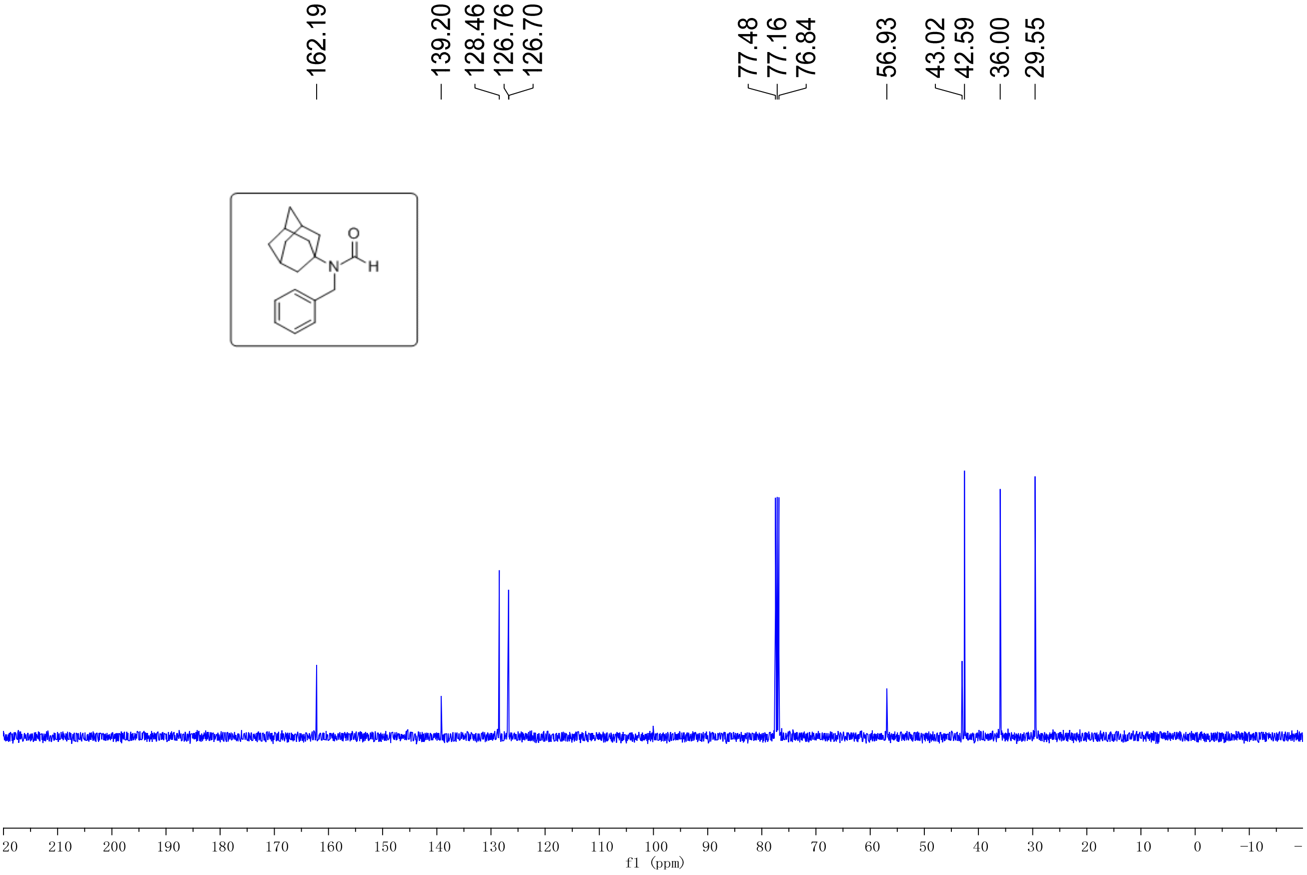

Supplementary Figure 43. ^{1}H and ^{13}C NMR spectra of compound **1x** in $CDCl_3$

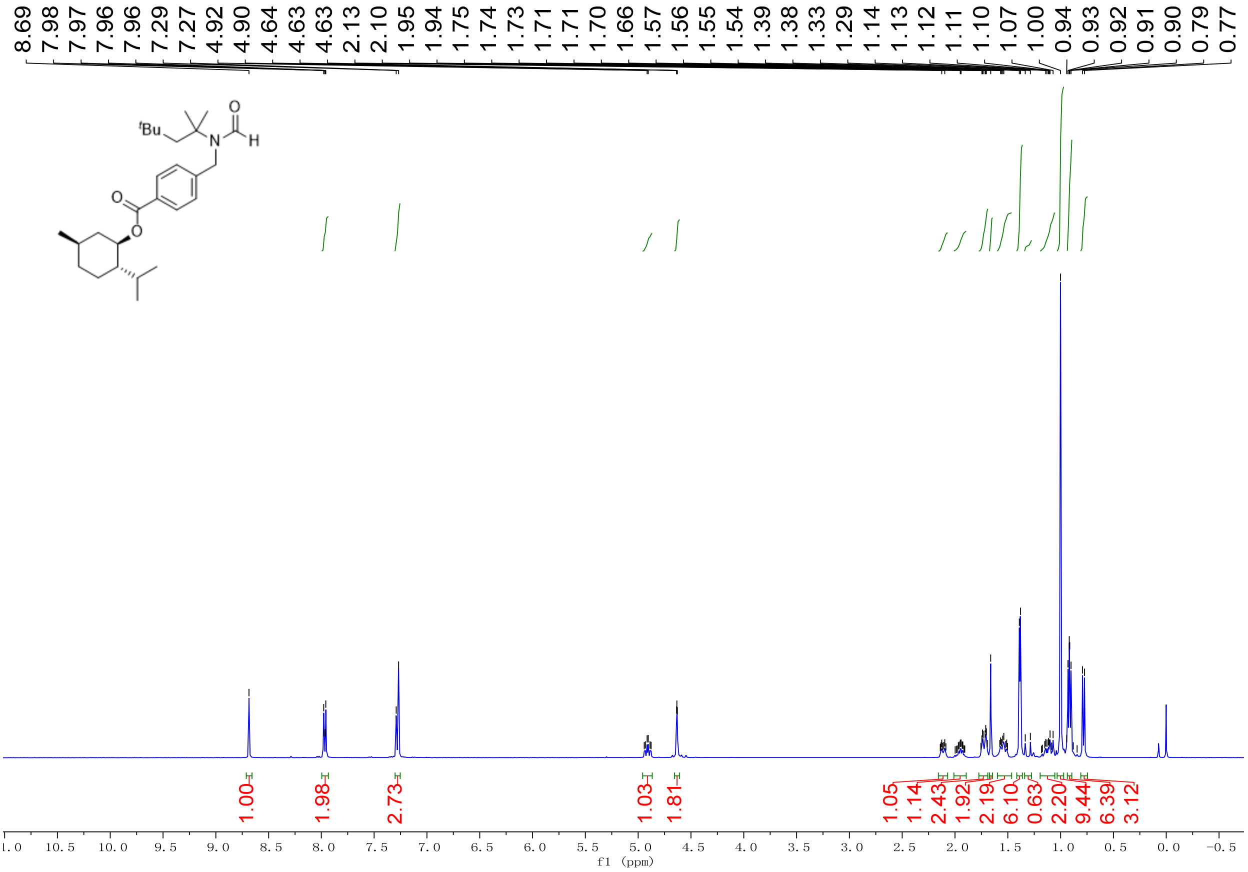

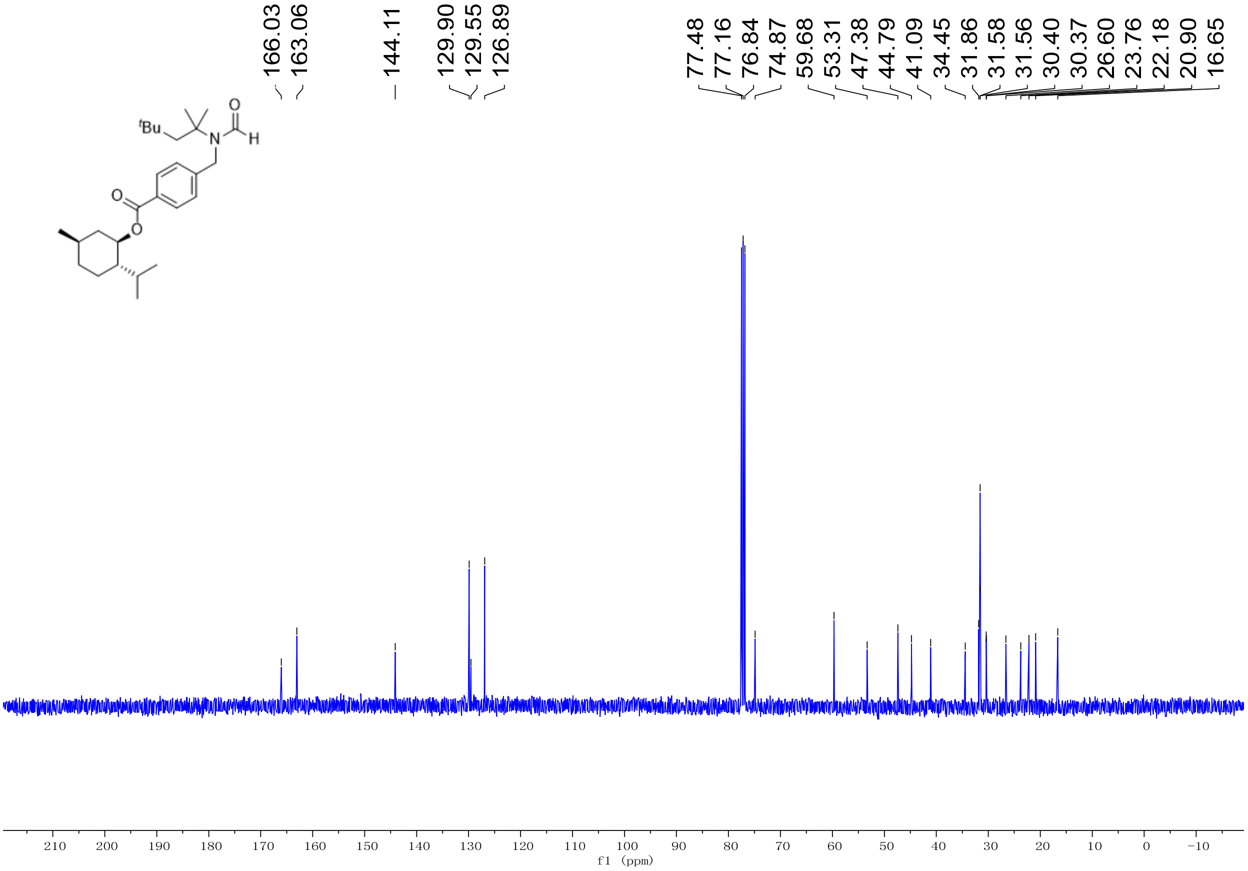

Supplementary Figure 44. ^{1}H and ^{13}C NMR spectra of compound **1k′** in $CDCl_3$

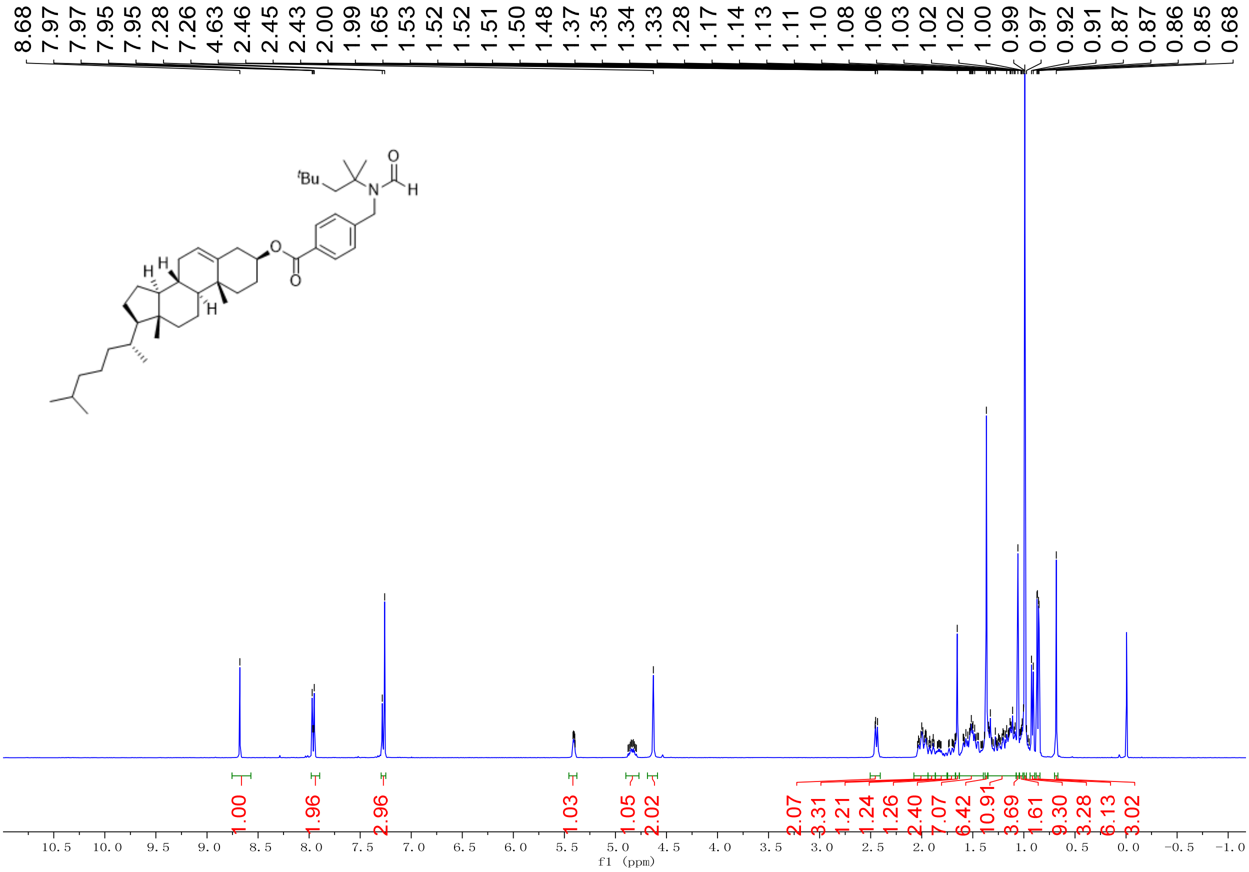

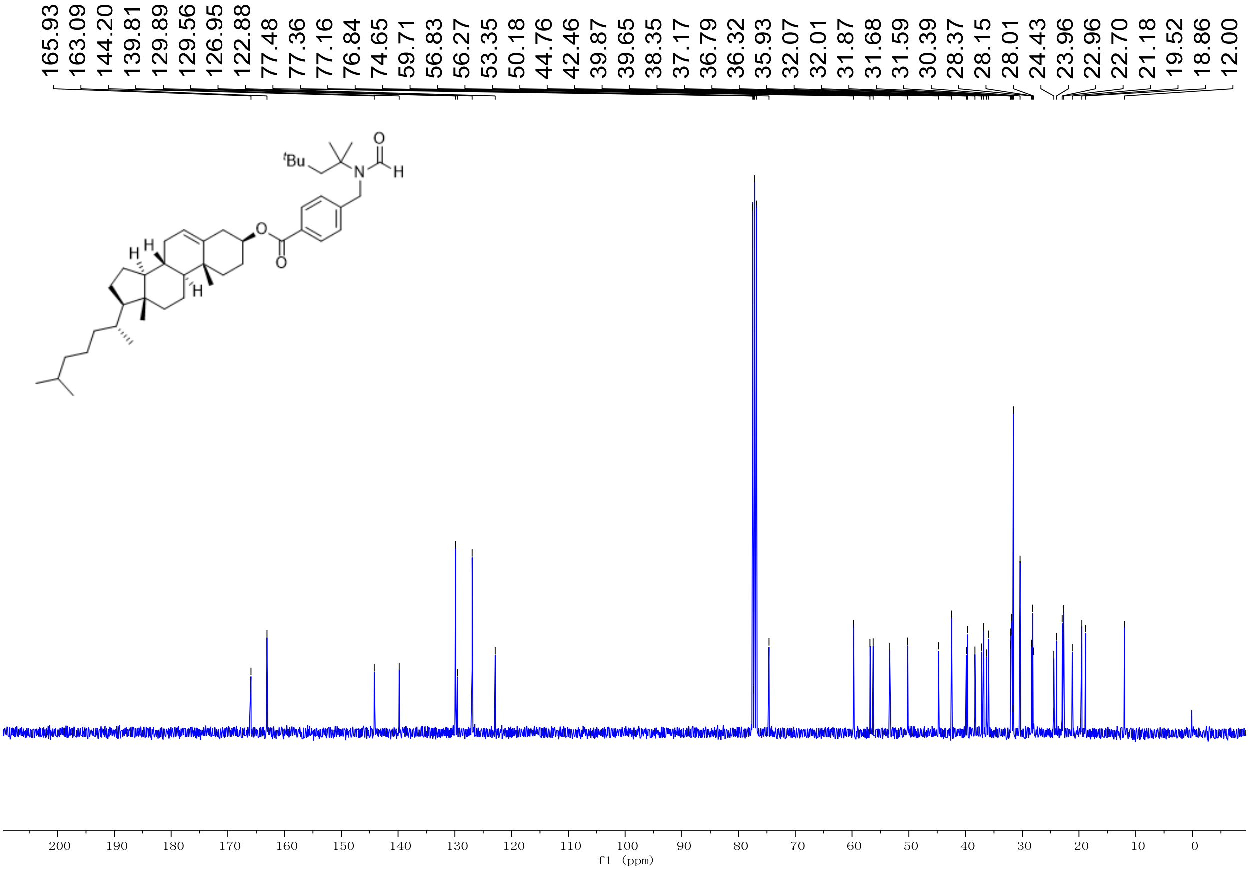

Supplementary Figure 45. ^{1}H and ^{13}C NMR spectra of compound **1l′** in $CDCl_3$

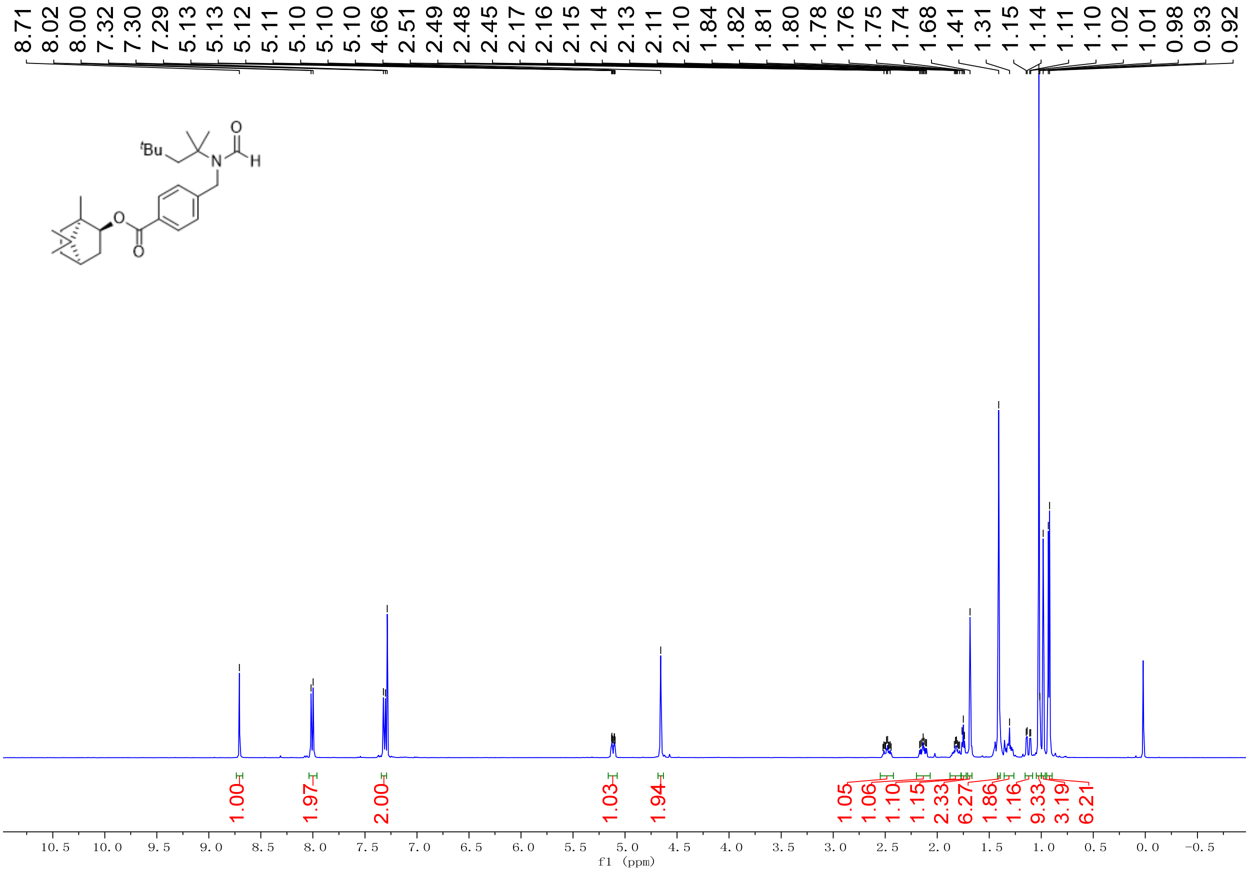

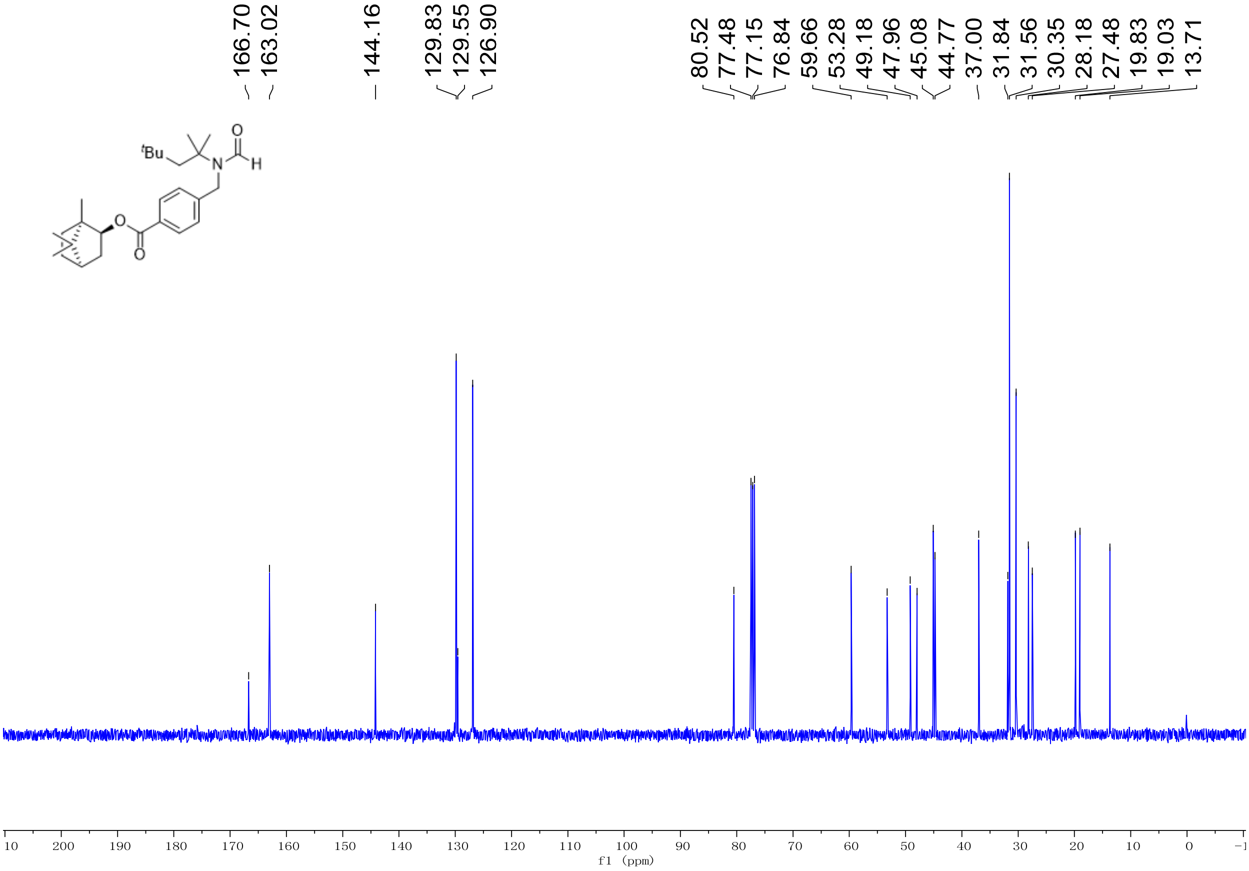

Supplementary Figure 46. ^{1}H and ^{13}C NMR spectra of compound **1m′** in $CDCl_3$

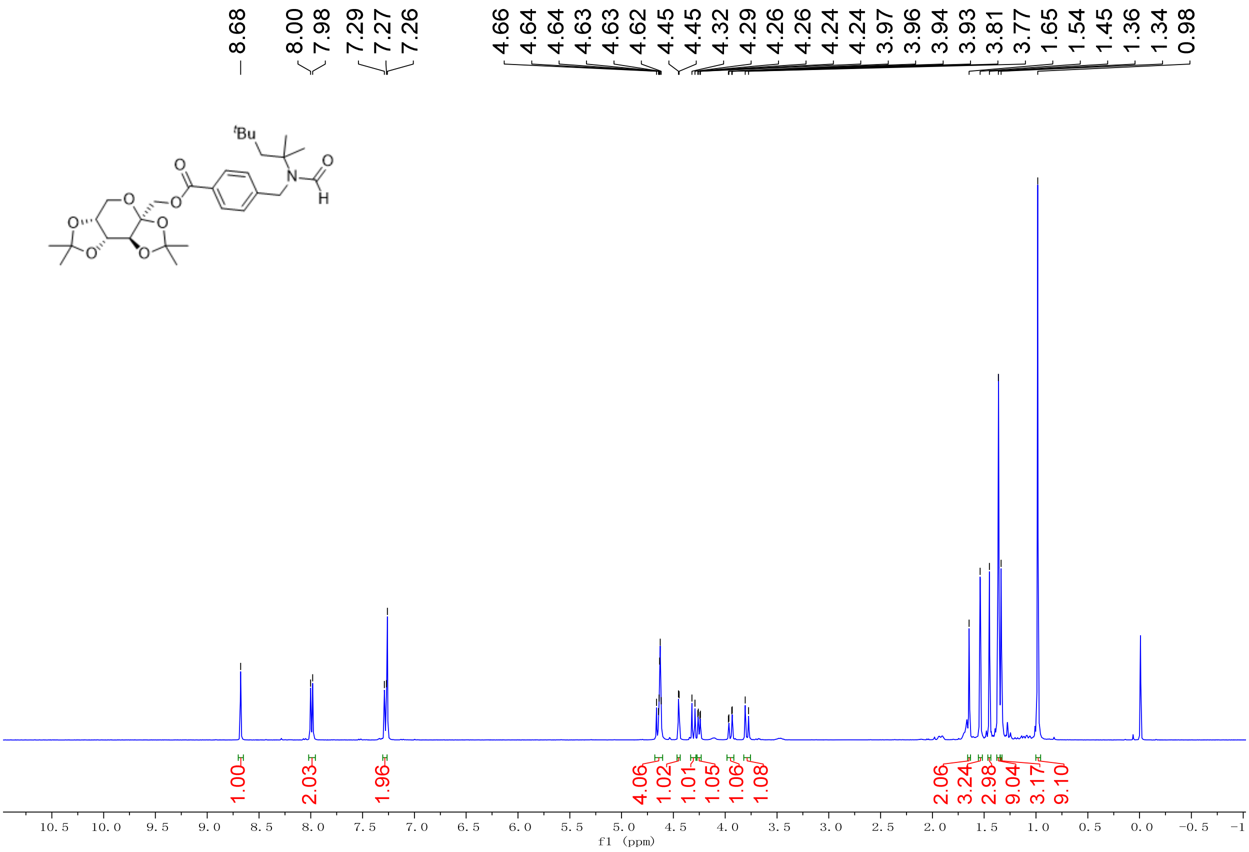

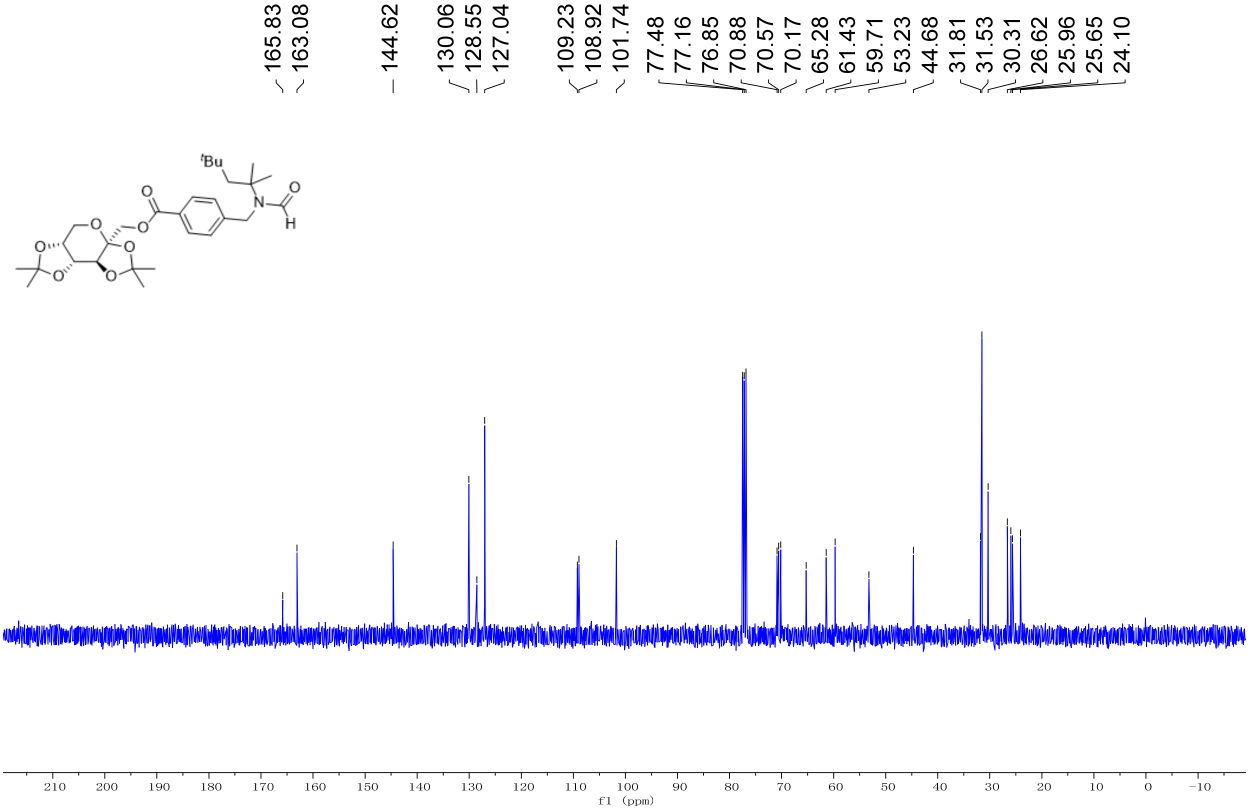

Supplementary Figure 47. ^{1}H and ^{13}C NMR spectra of compound **1n′** in $CDCl_3$

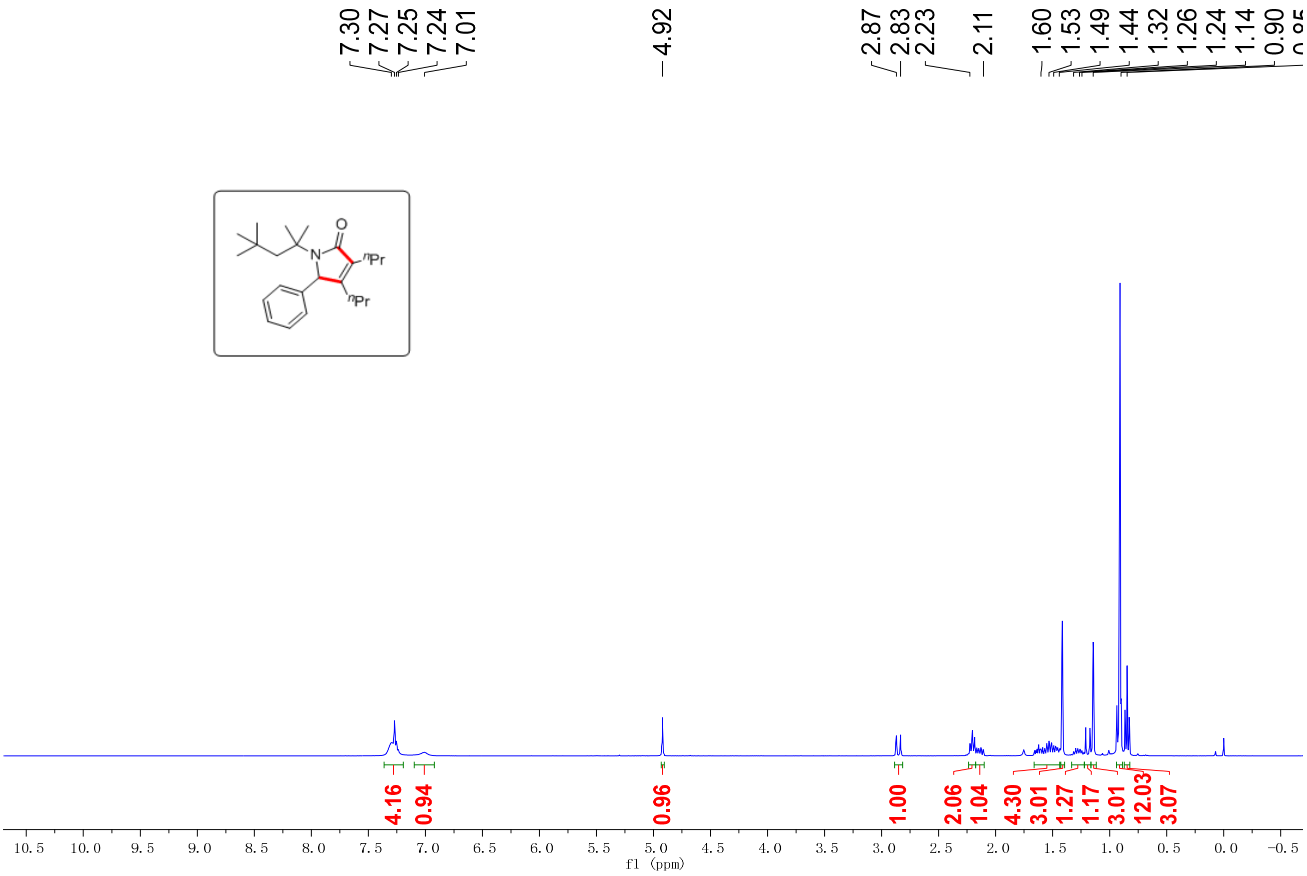

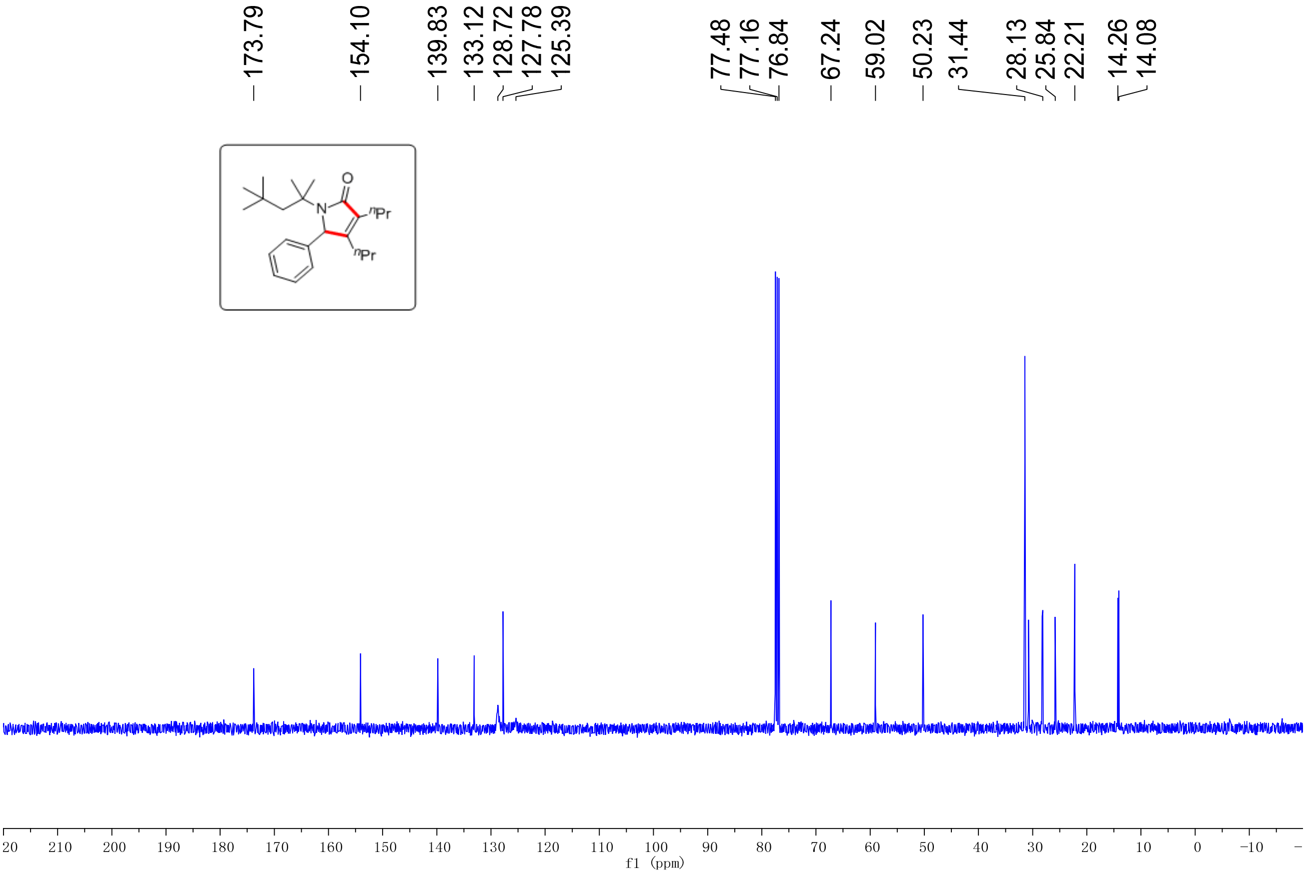

Supplementary Figure 48. ^{1}H and ^{13}C NMR spectra of compound **3a** in $CDCl_3$

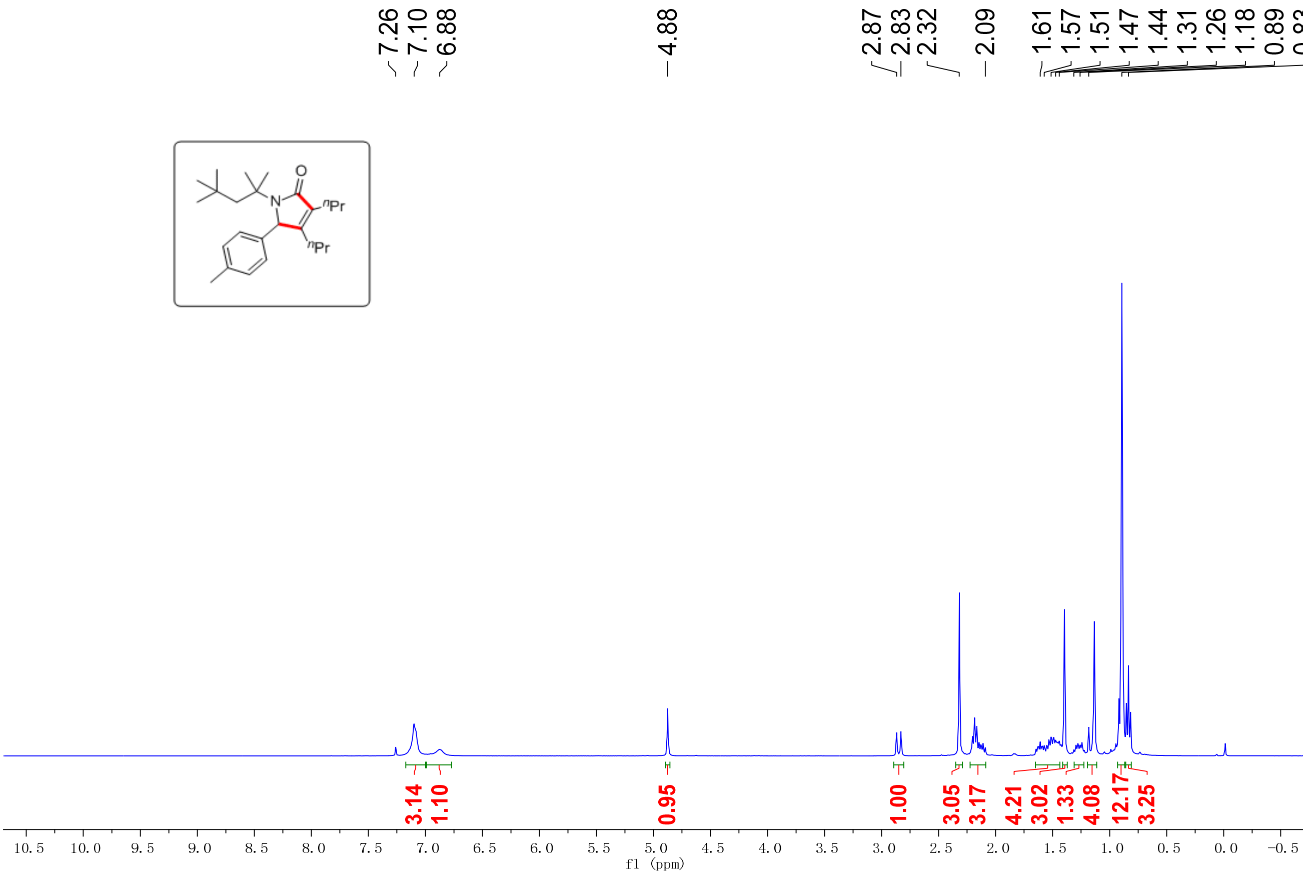

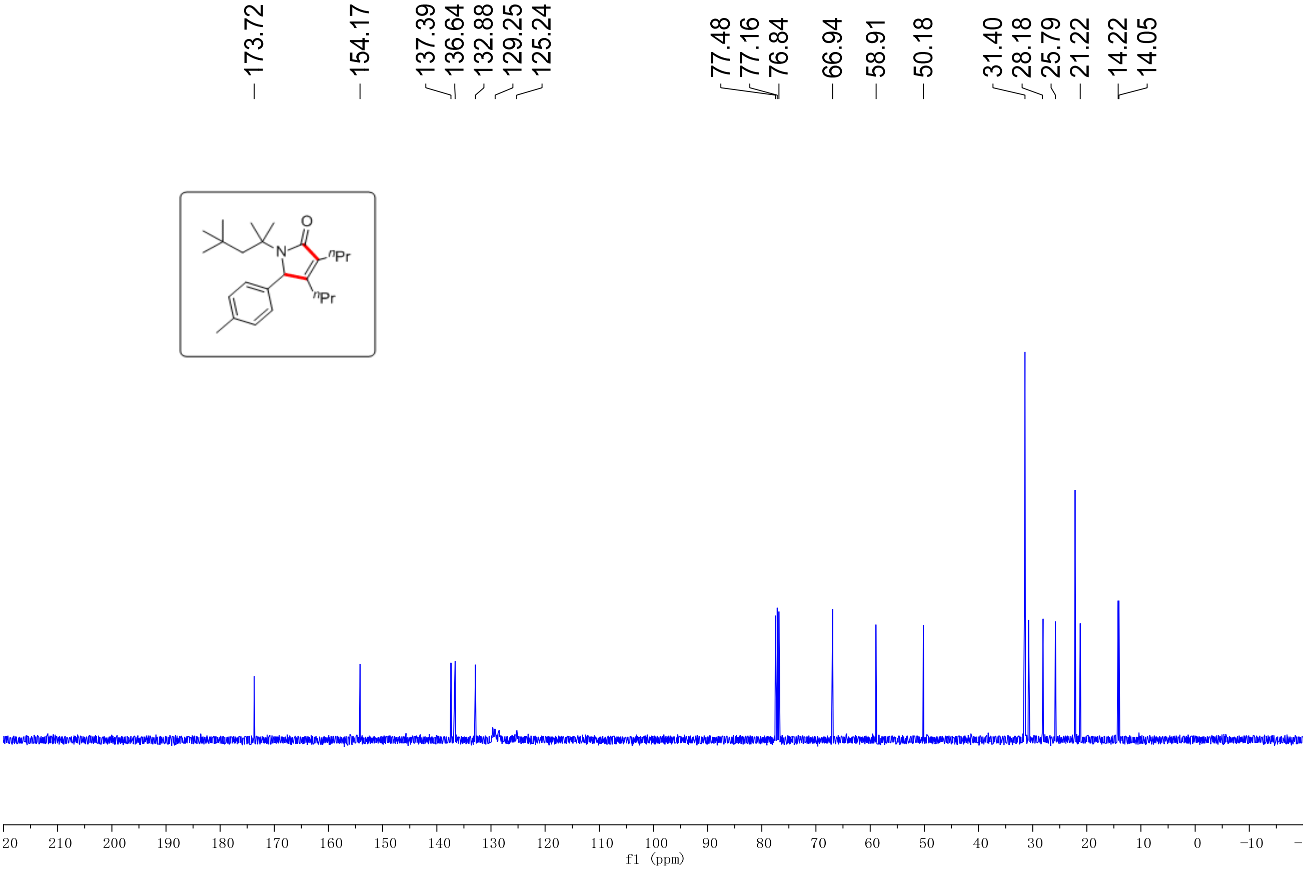

Supplementary Figure 49. ^{1}H and ^{13}C NMR spectra of compound **3b** in $CDCl_3$

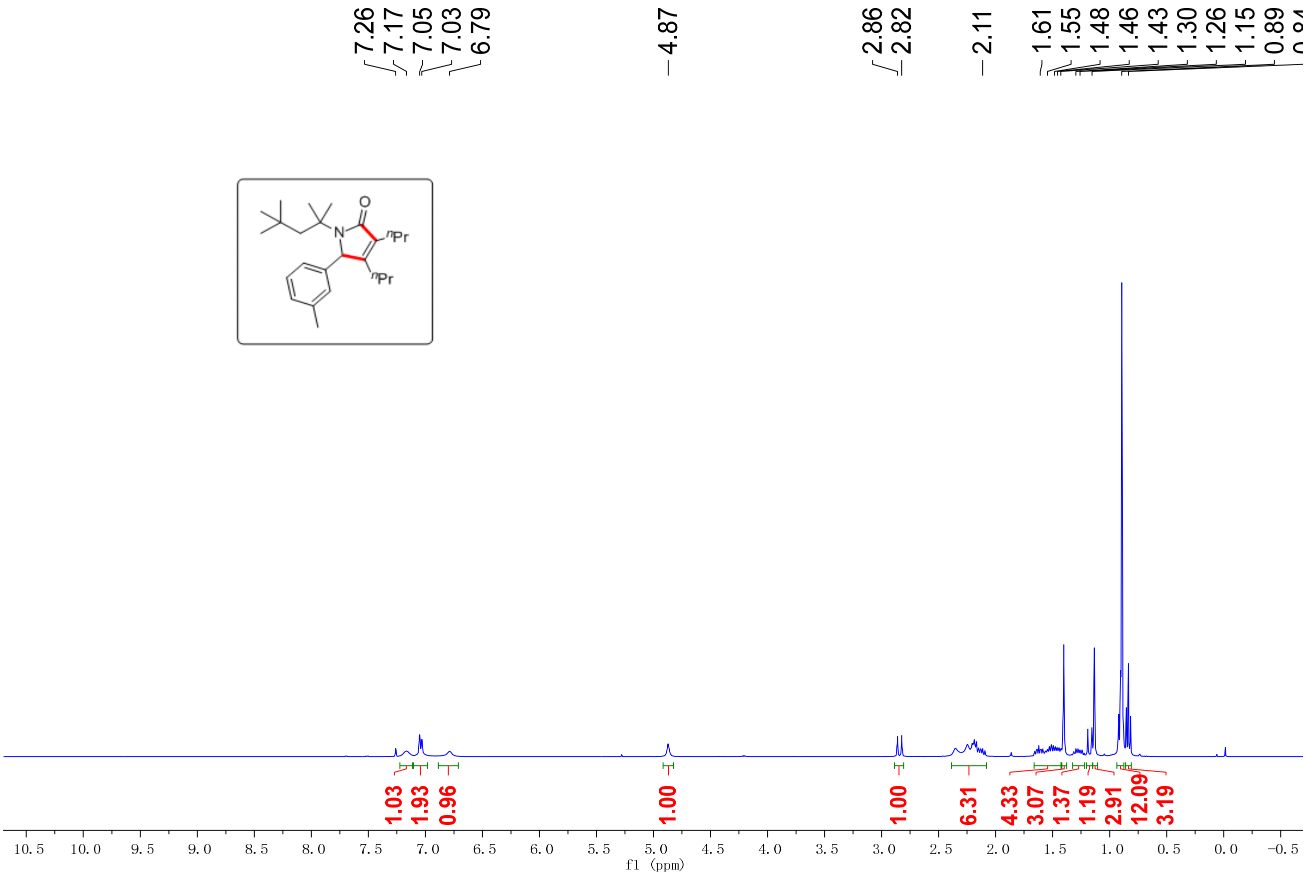

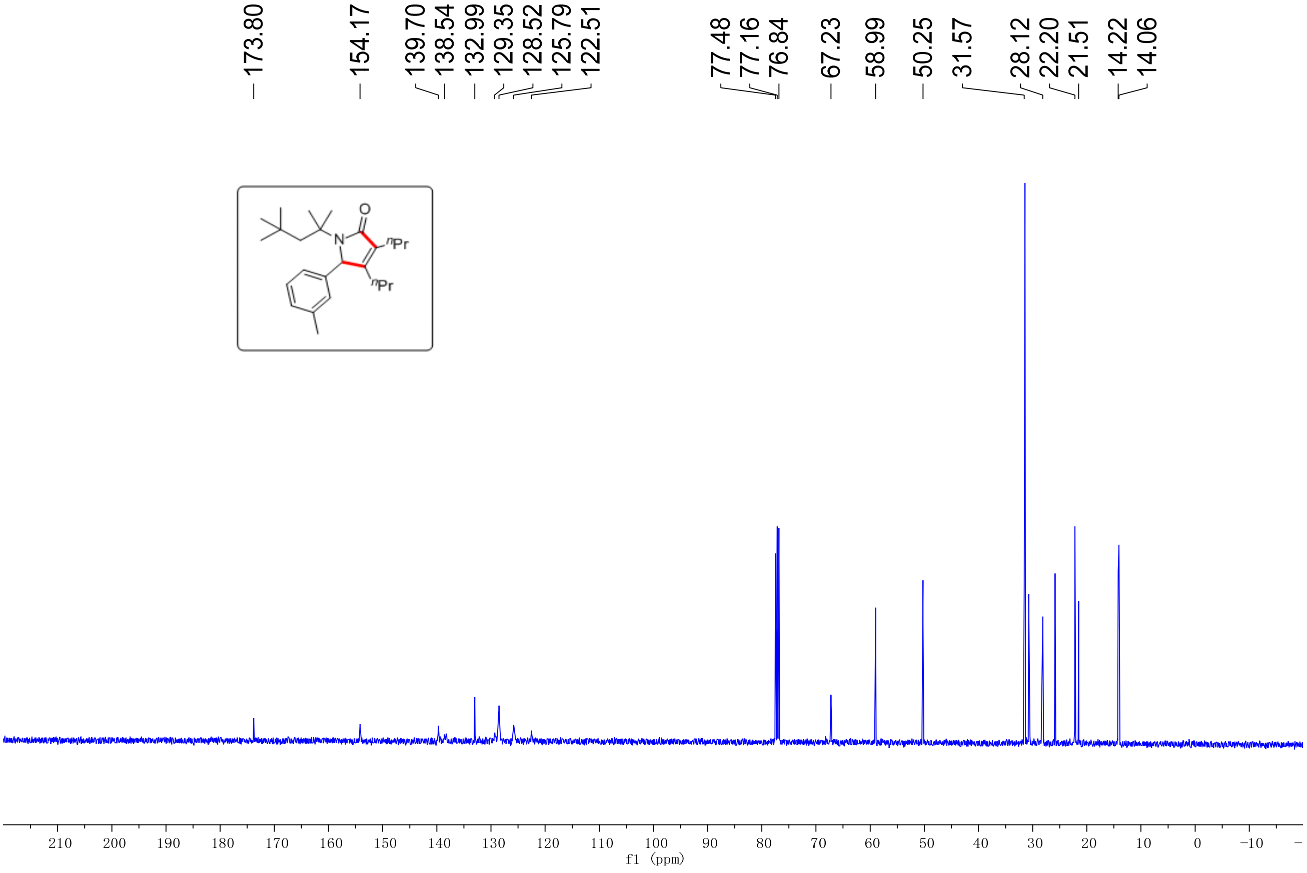

Supplementary Figure 50. ^{1}H and ^{13}C NMR spectra of compound **3c** in $CDCl_3$

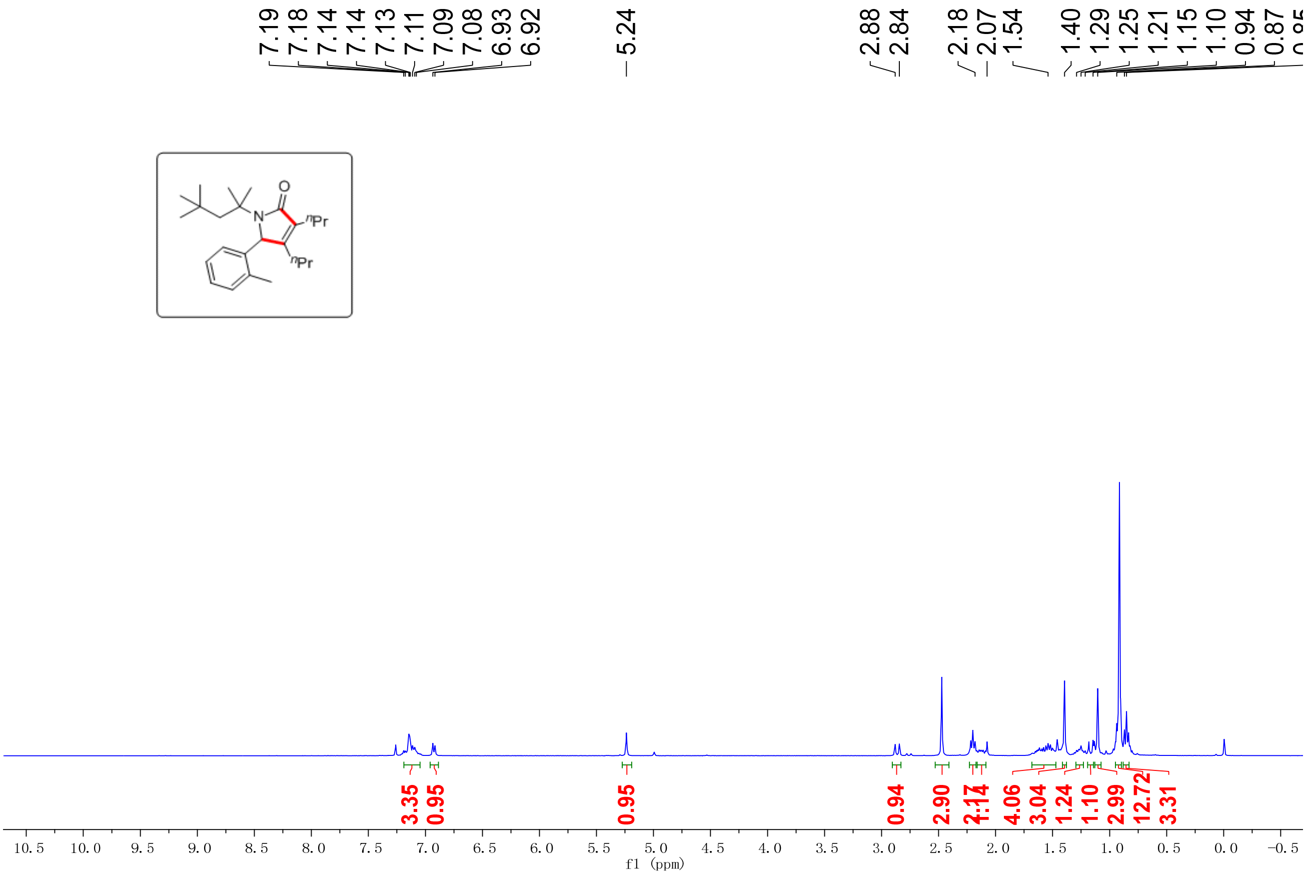

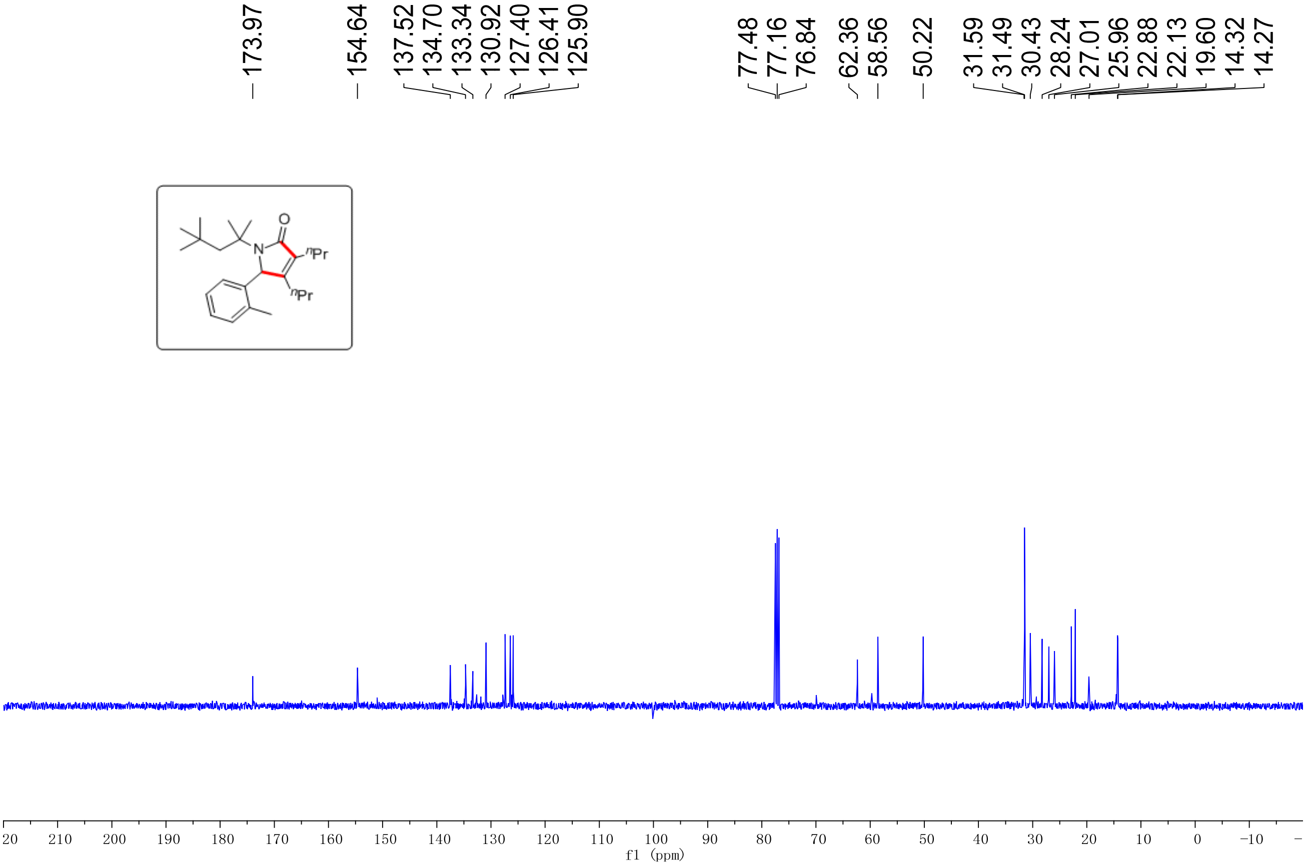

Supplementary Figure 51. ^{1}H and ^{13}C NMR spectra of compound **3d** in $CDCl_3$

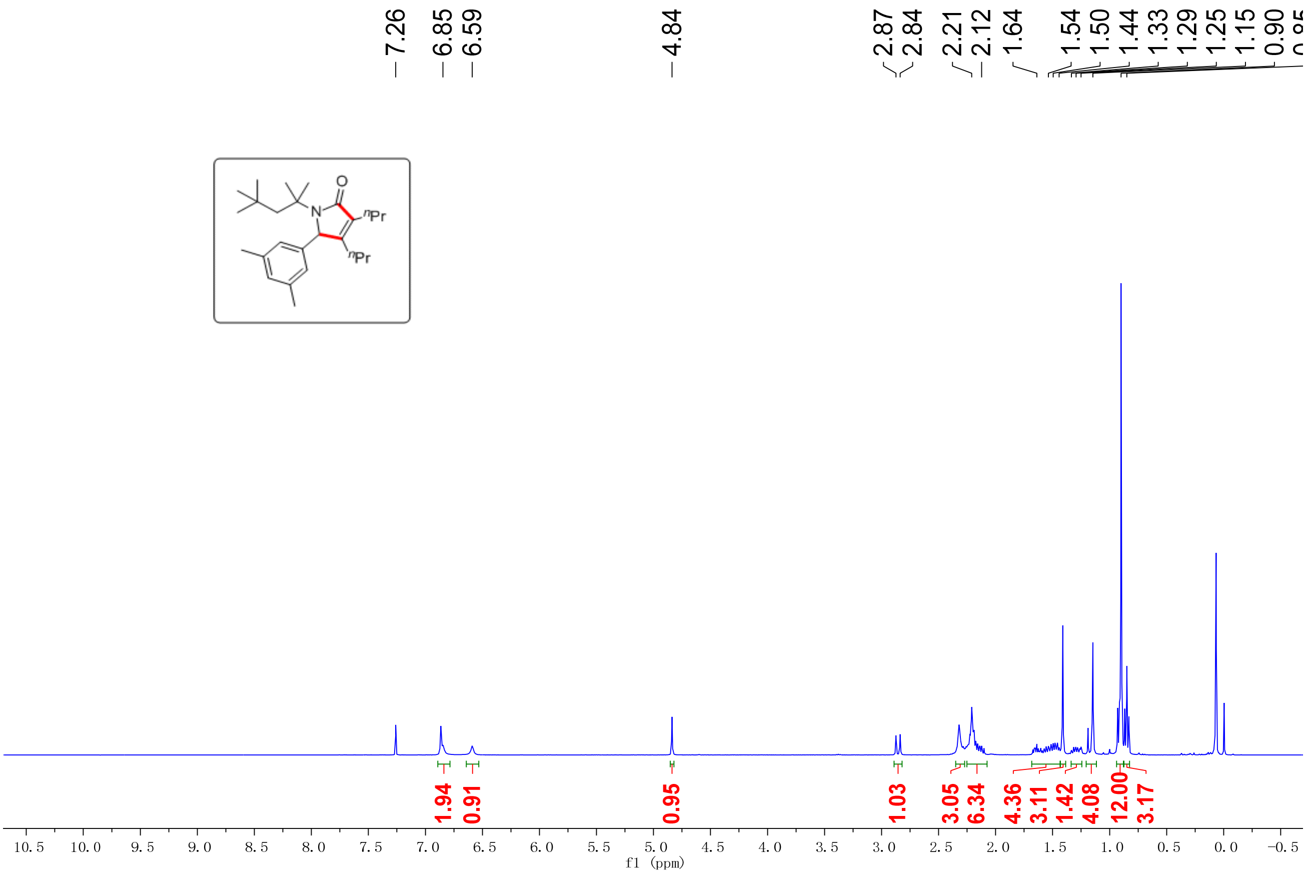

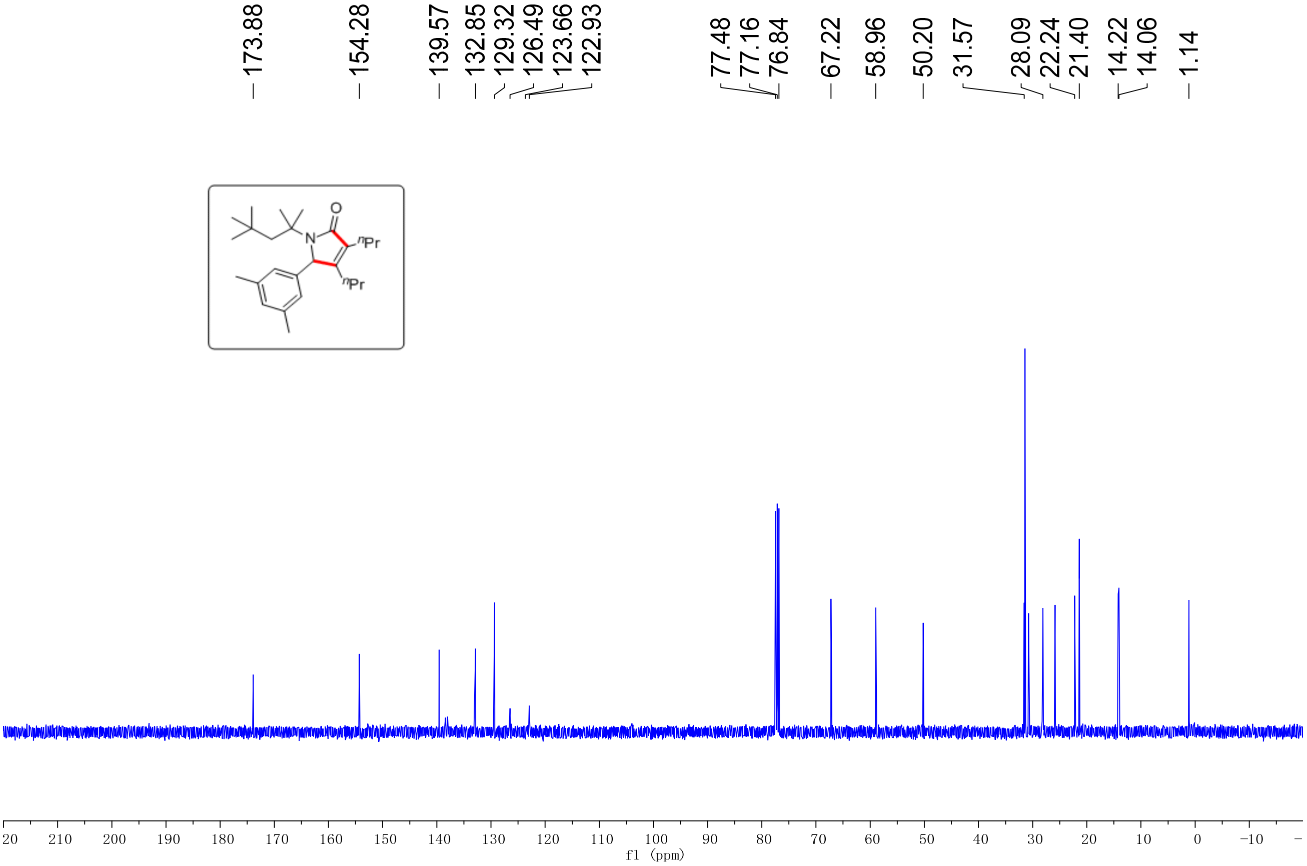

Supplementary Figure 52. ^{1}H and ^{13}C NMR spectra of compound **3e** in $CDCl_3$

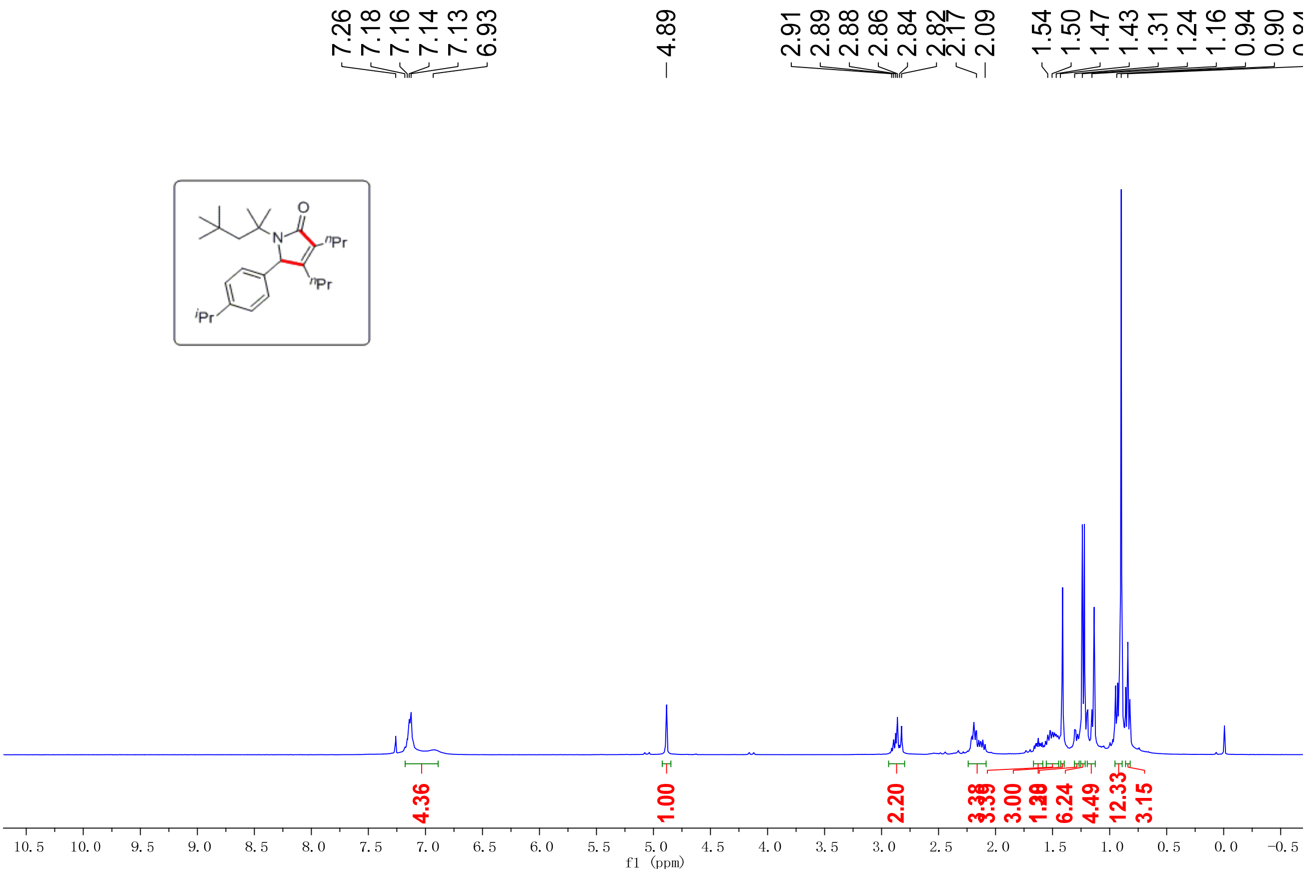

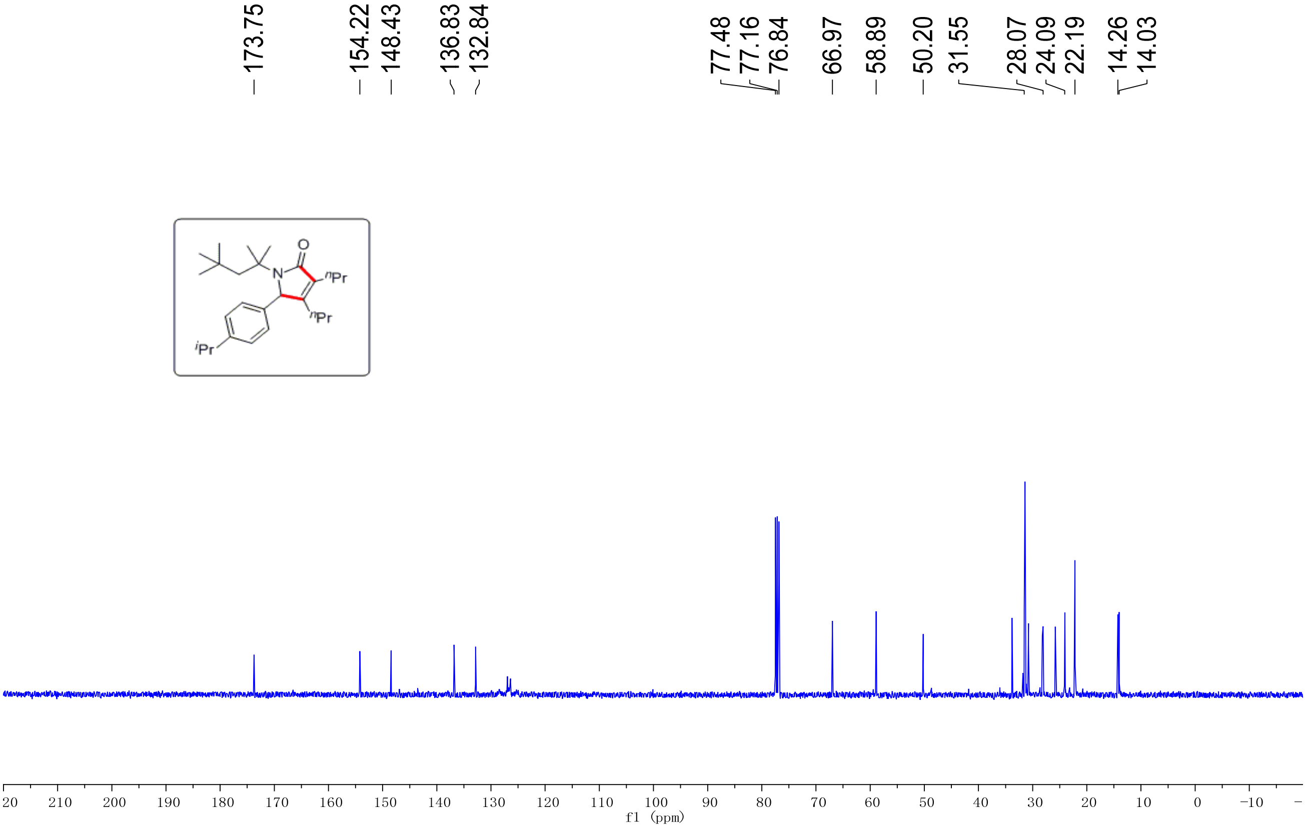

Supplementary Figure 53. ^{1}H and ^{13}C NMR spectra of compound **3f** in $CDCl_3$

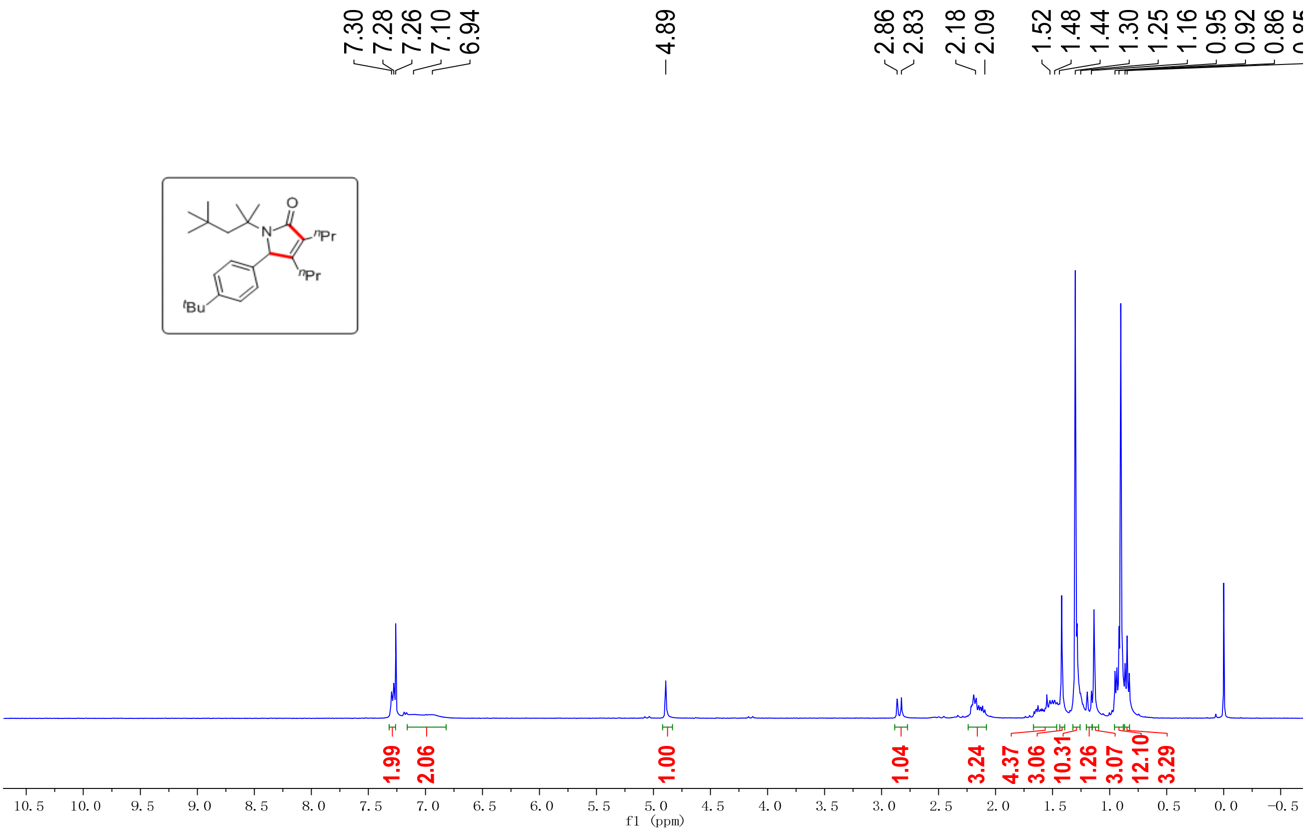

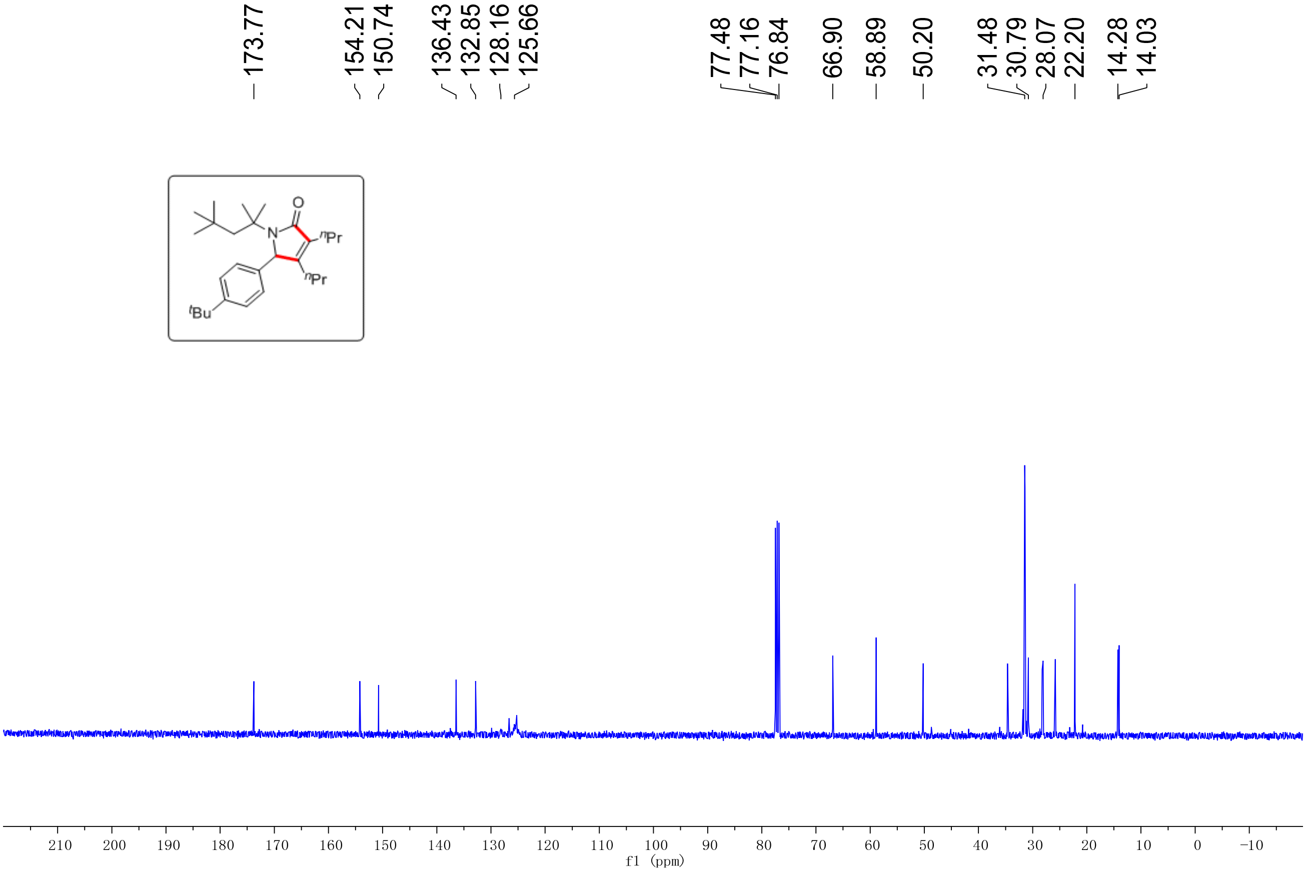

Supplementary Figure 54. ^{1}H and ^{13}C NMR spectra of compound **3g** in $CDCl_3$

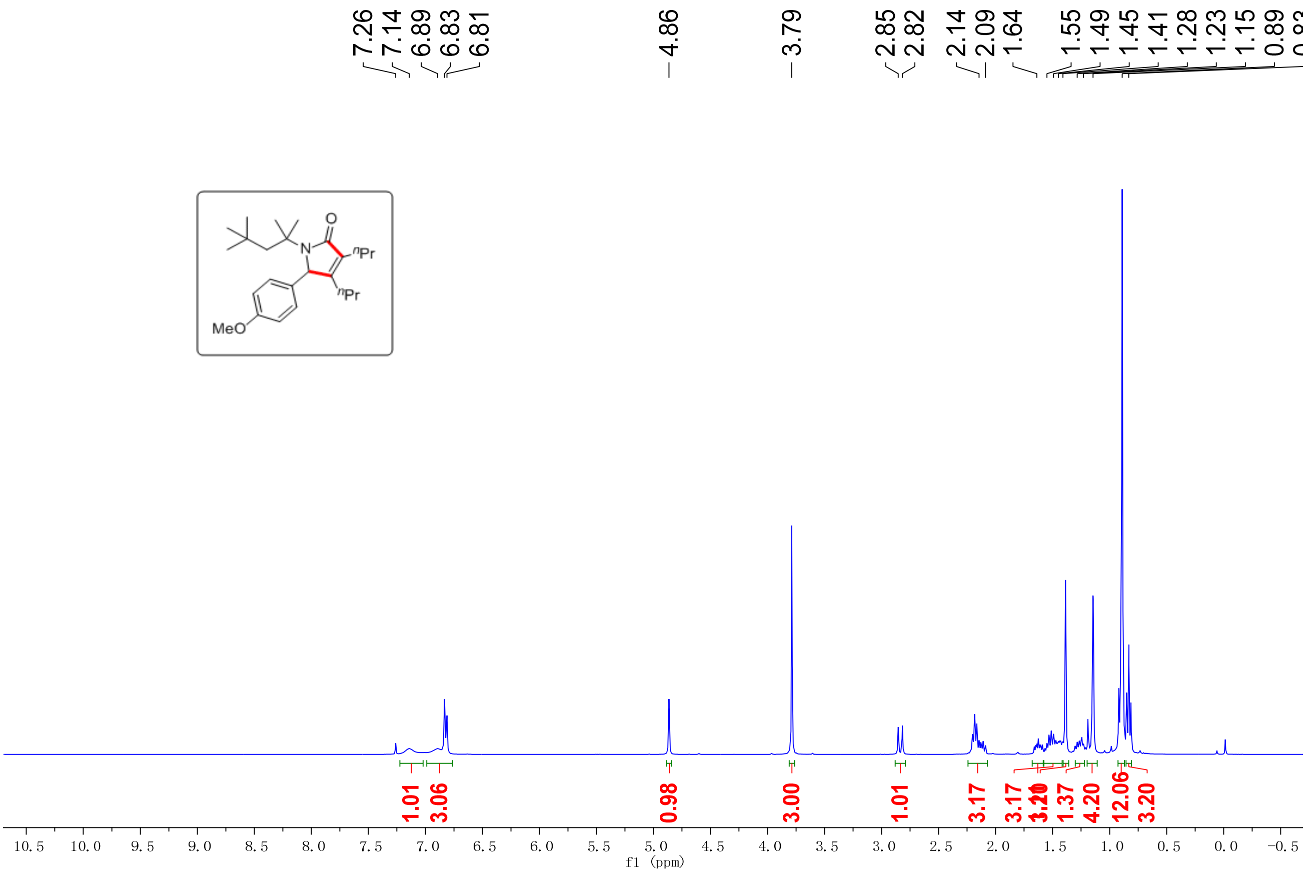

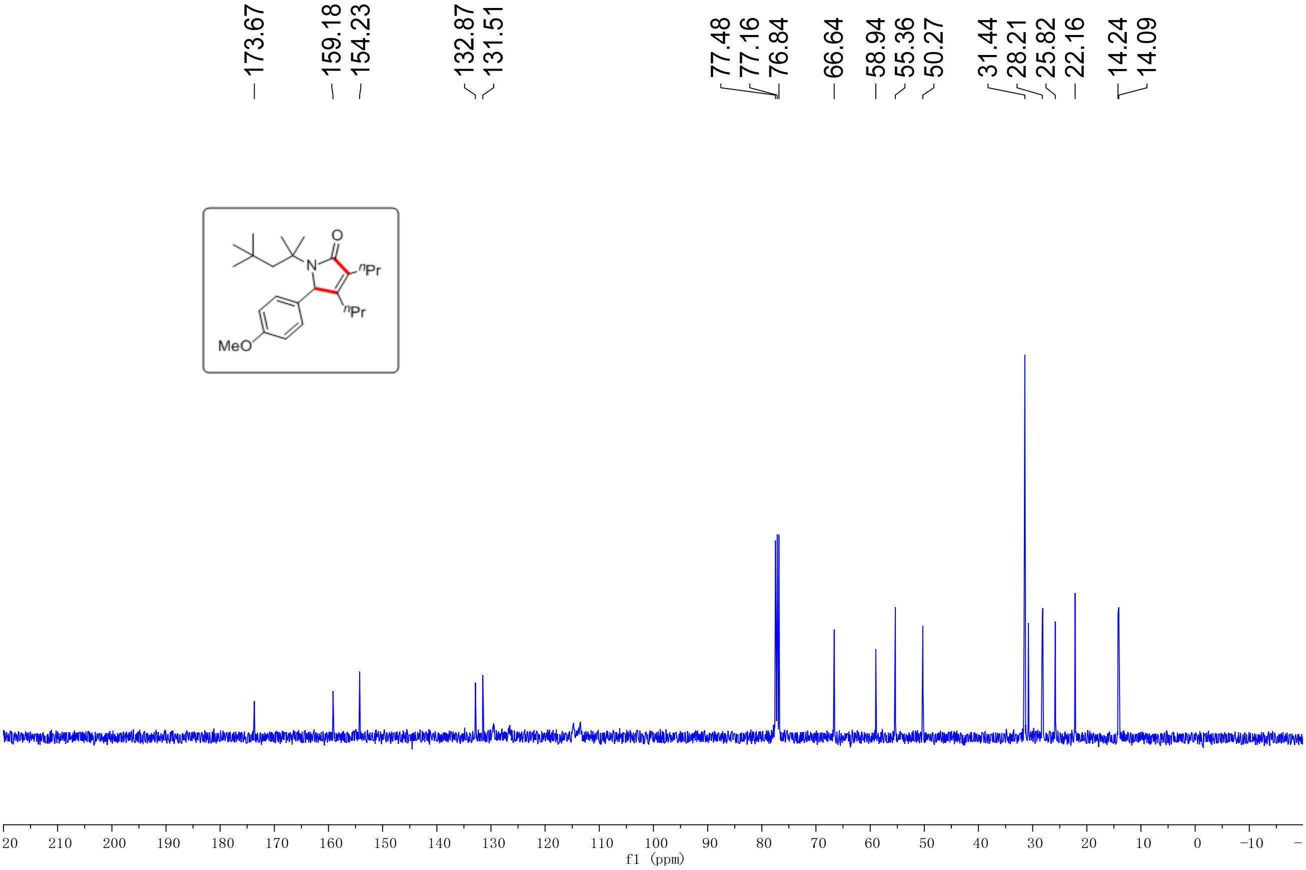

Supplementary Figure 55. ^{1}H and ^{13}C NMR spectra of compound **3h** in $CDCl_3$

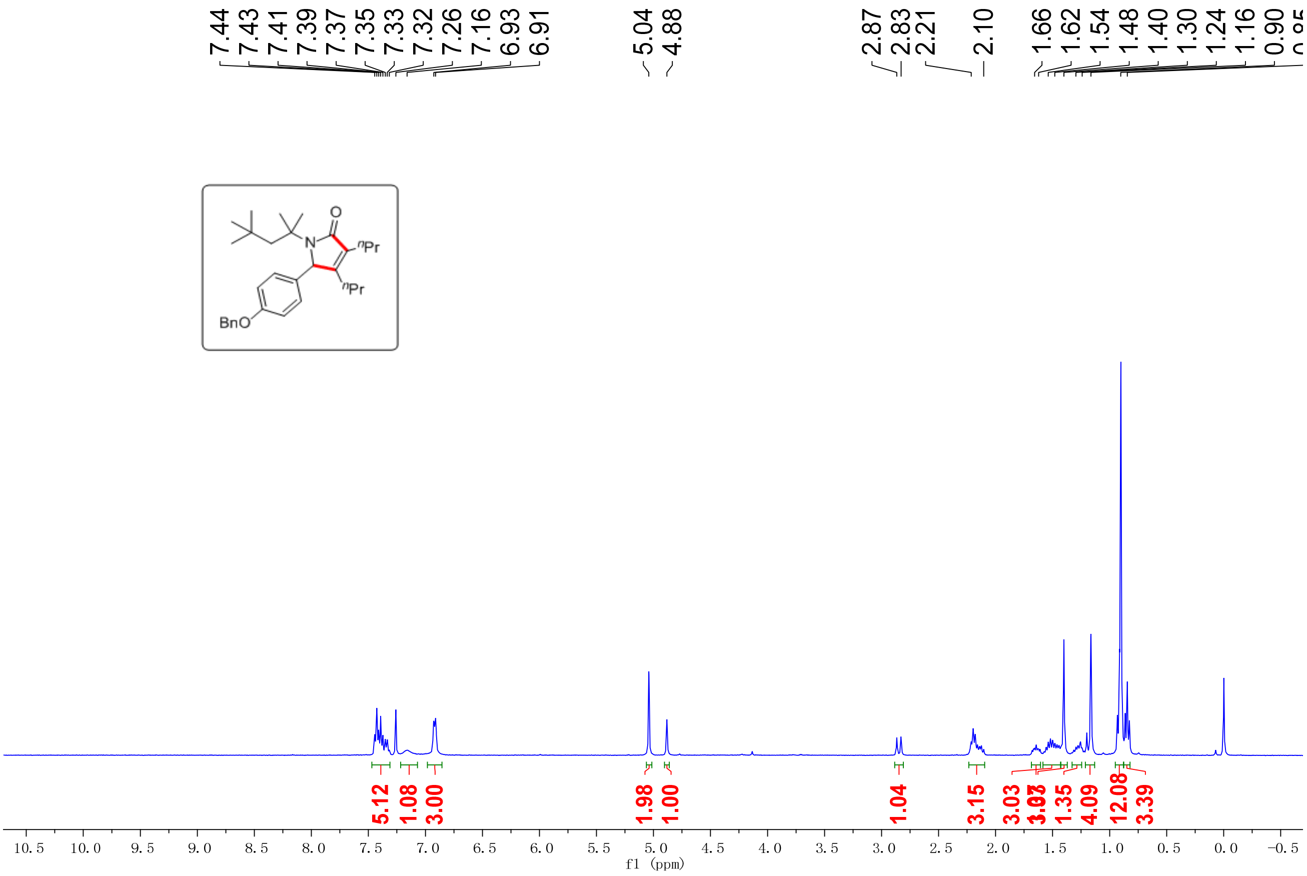

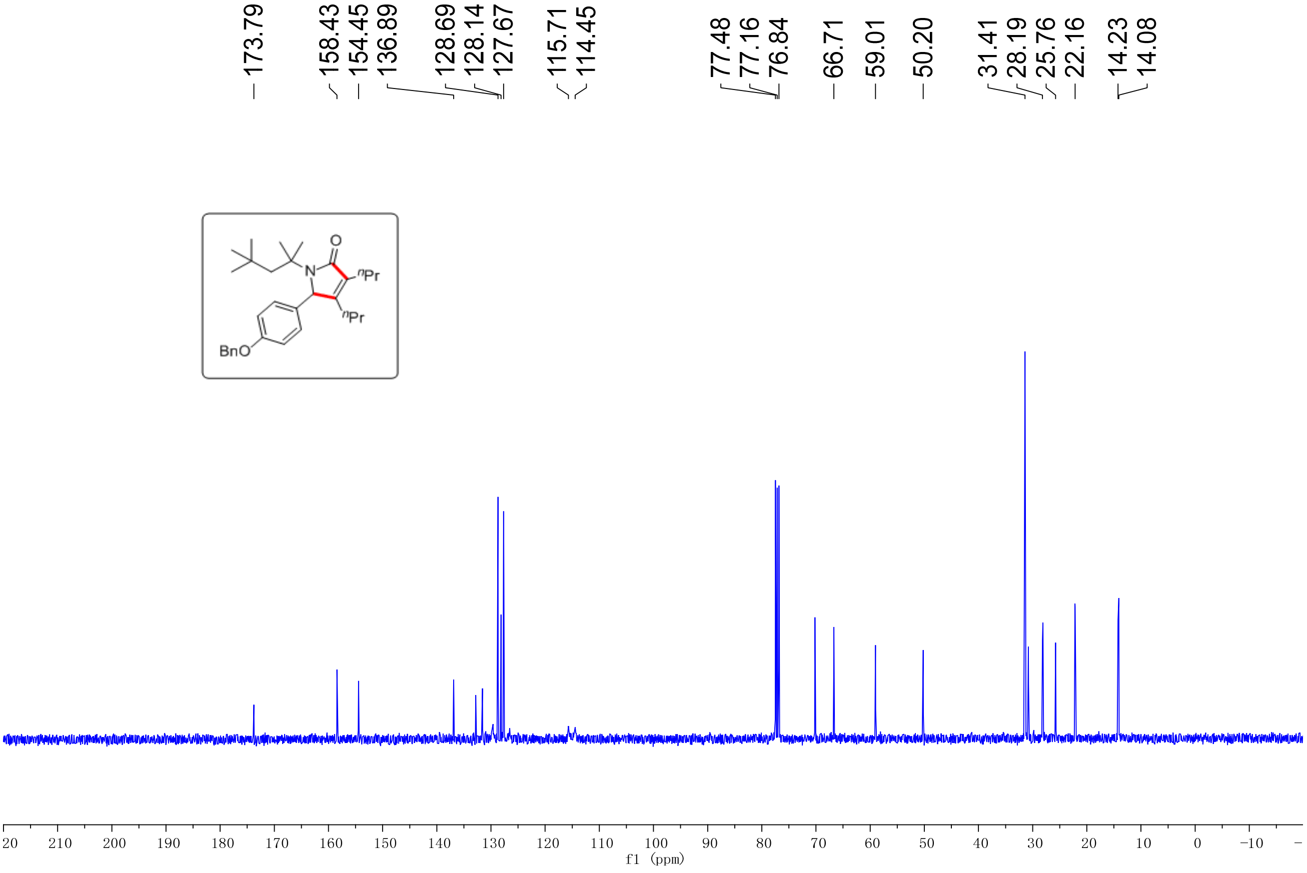

Supplementary Figure 56. ^{1}H and ^{13}C NMR spectra of compound **3i** in $CDCl_3$

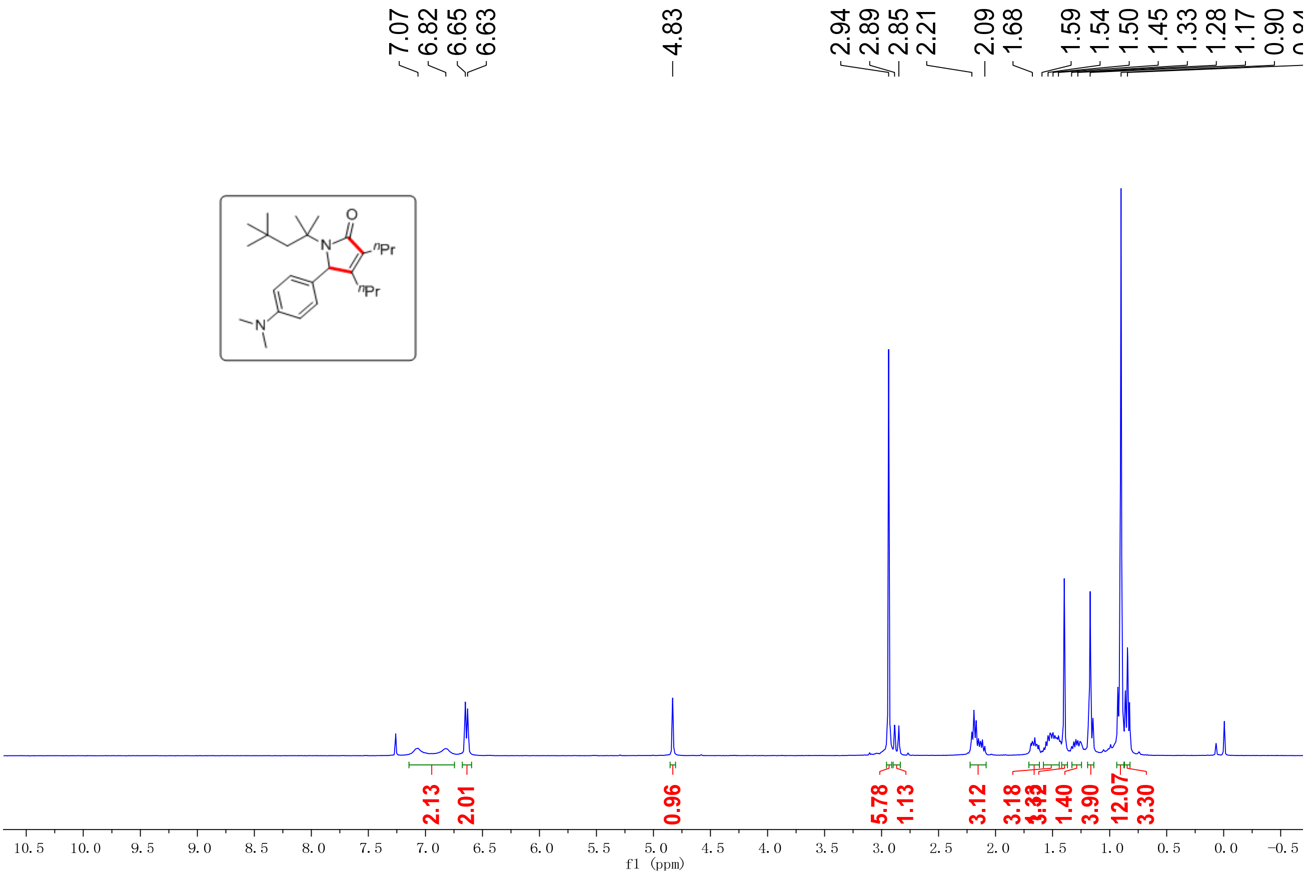

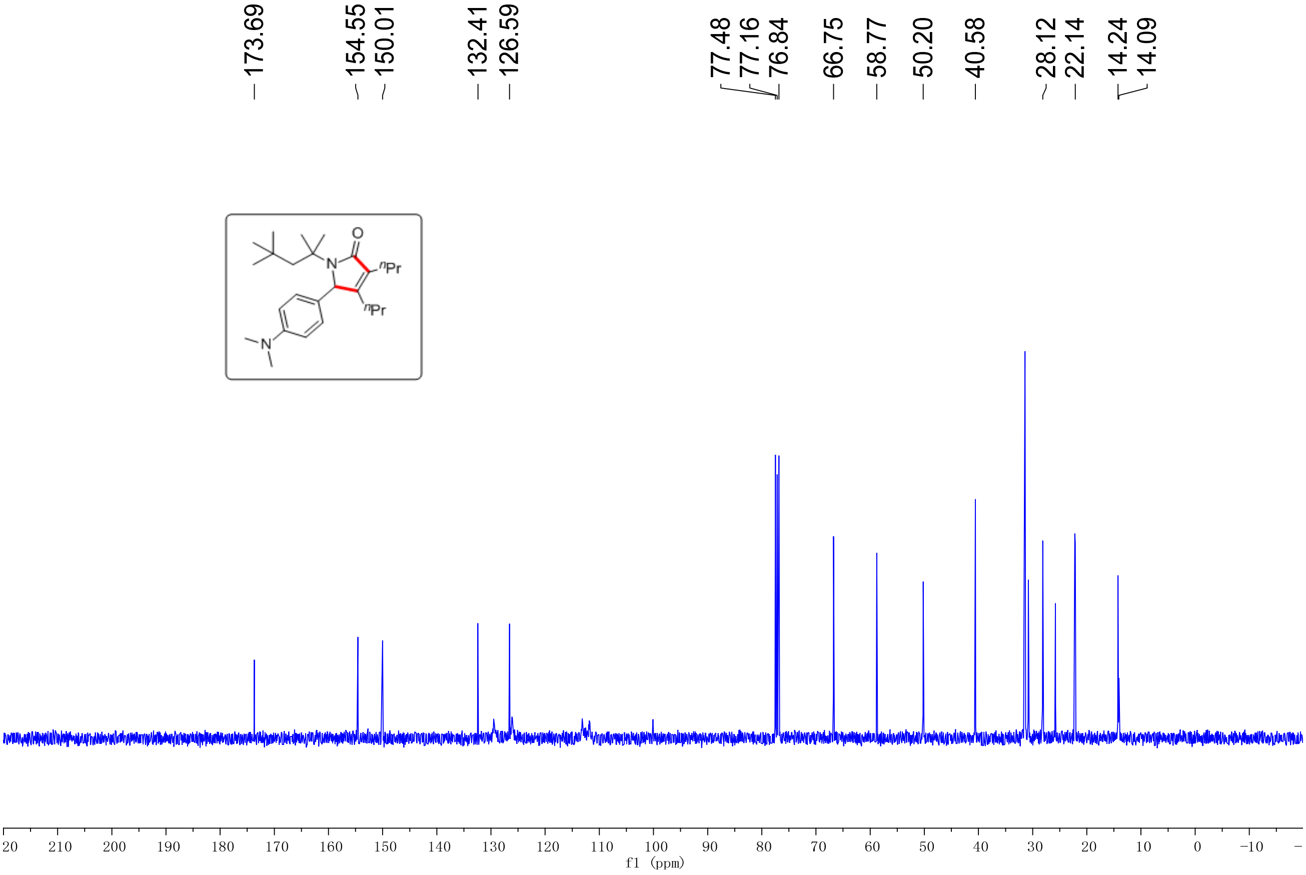

Supplementary Figure 57. ^{1}H and ^{13}C NMR spectra of compound **3j** in $CDCl_3$

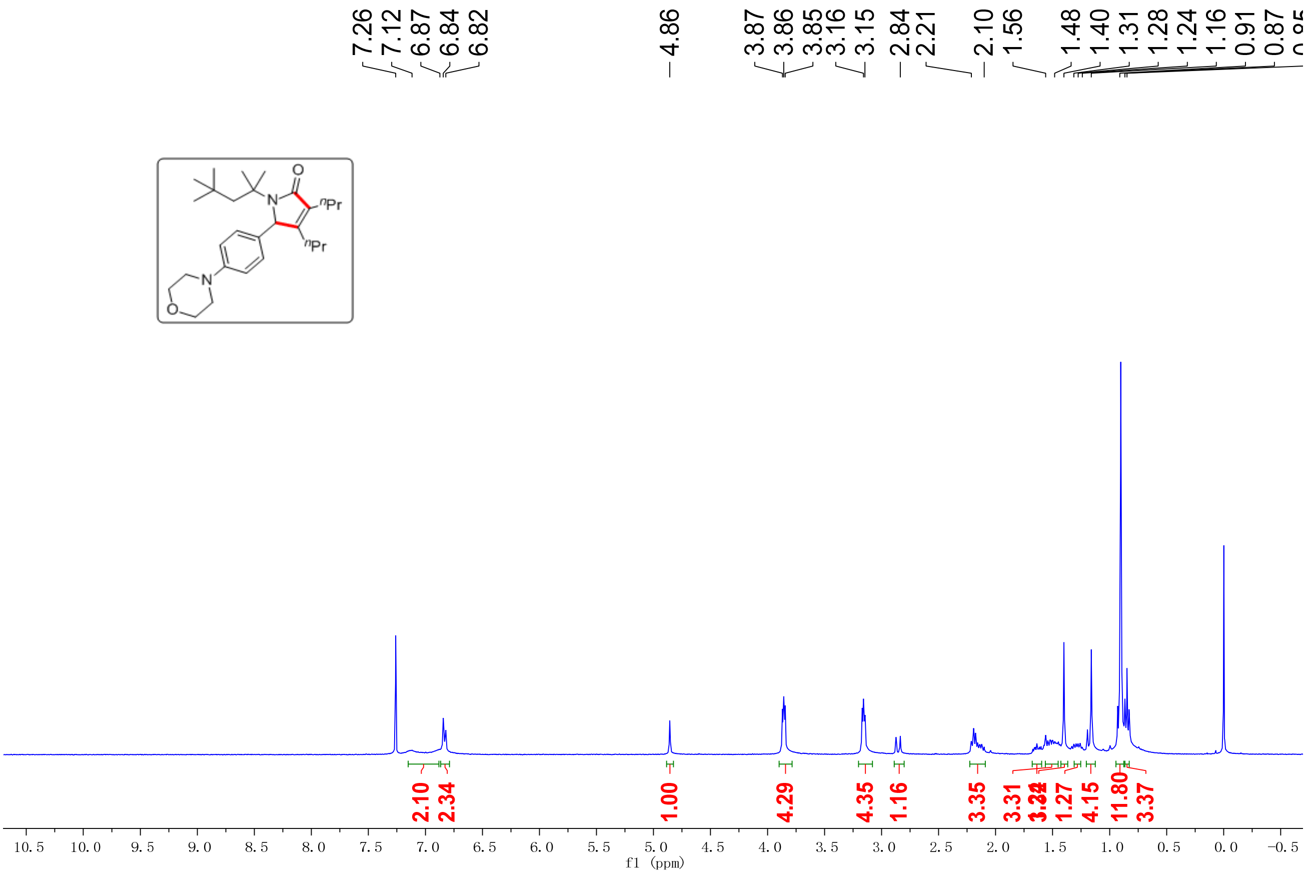

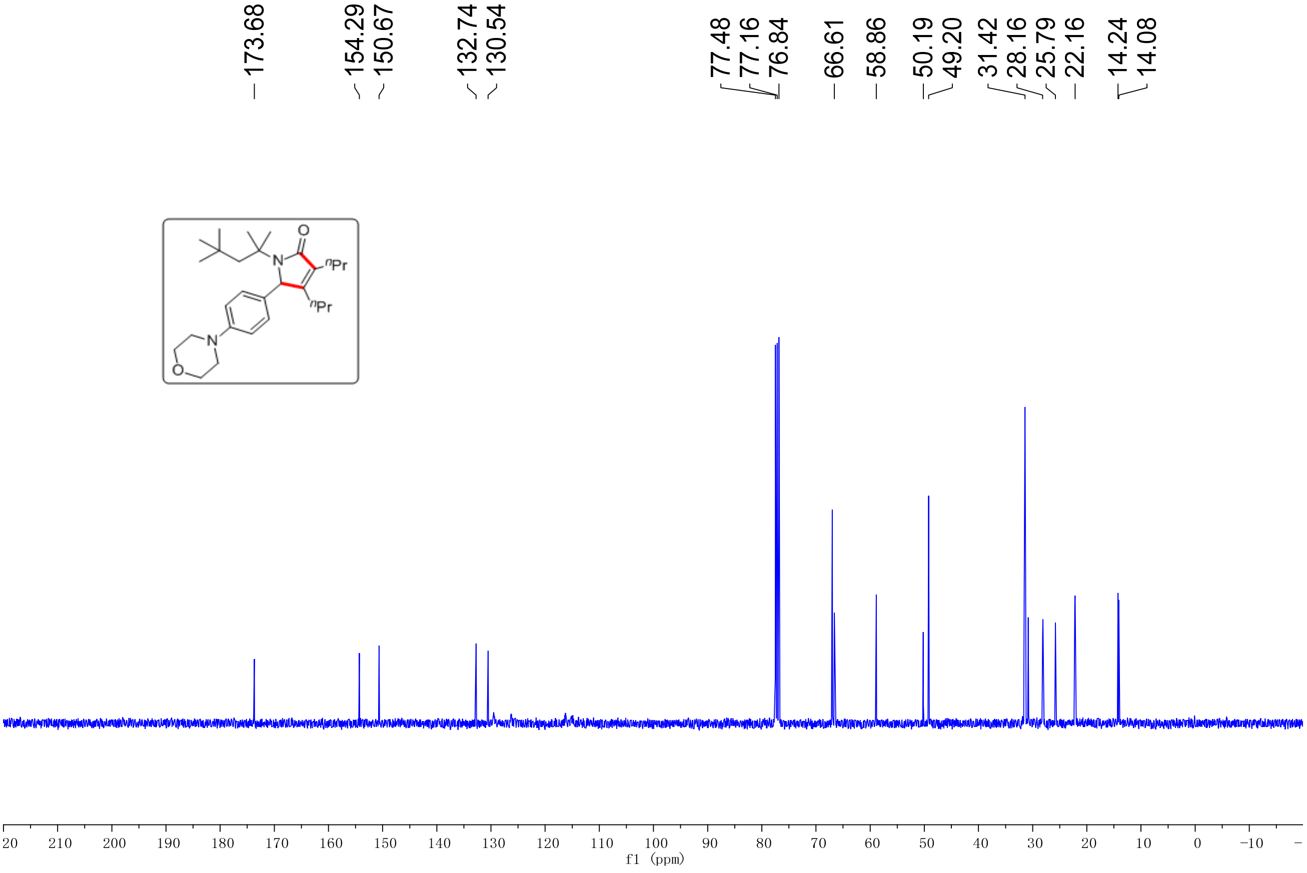

Supplementary Figure 58. ^{1}H and ^{13}C NMR spectra of compound **3k** in $CDCl_3$

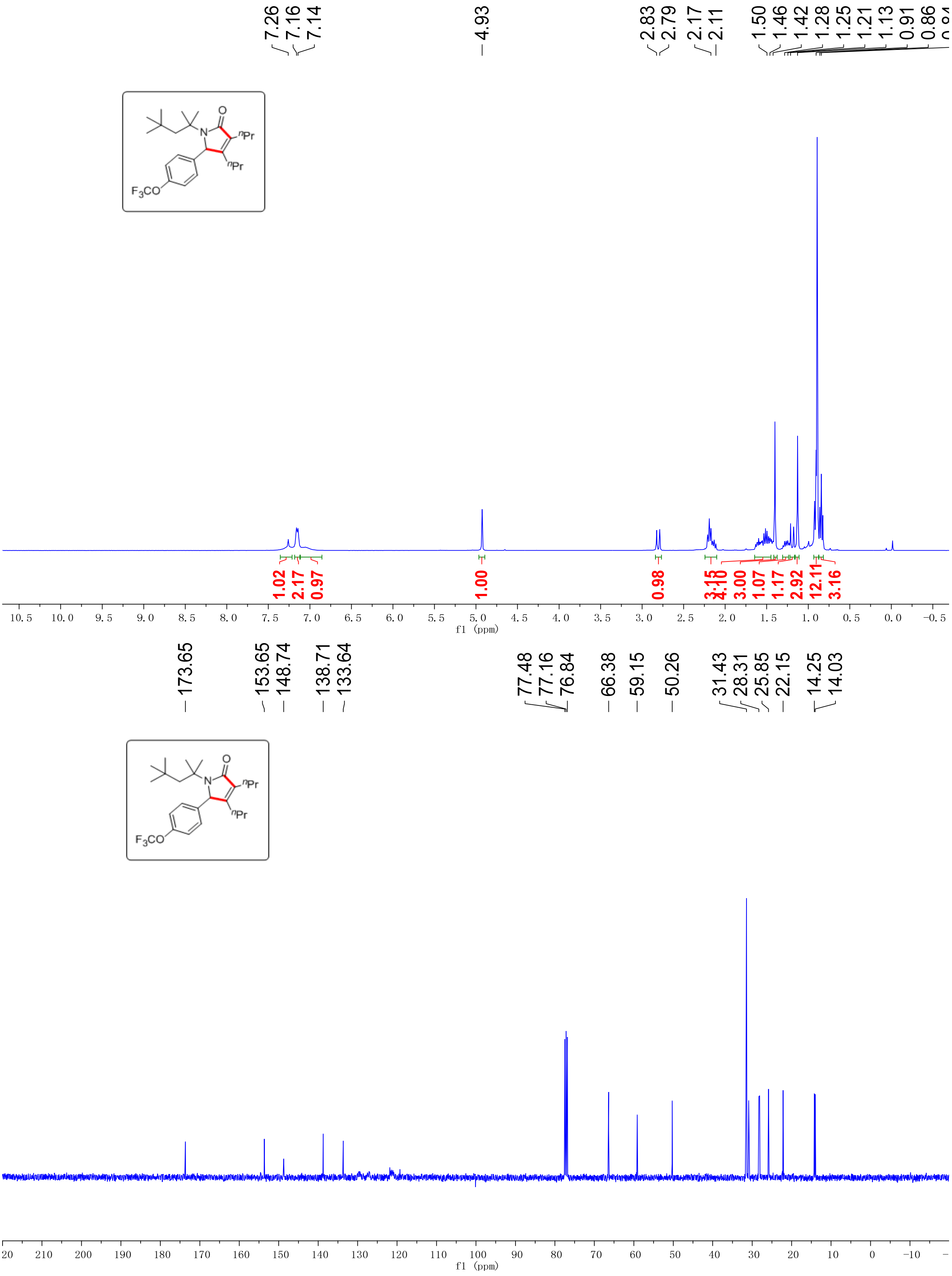

Supplementary Figure 59. ^{1}H and ^{13}C NMR spectra of compound **3l** in $CDCl_3$

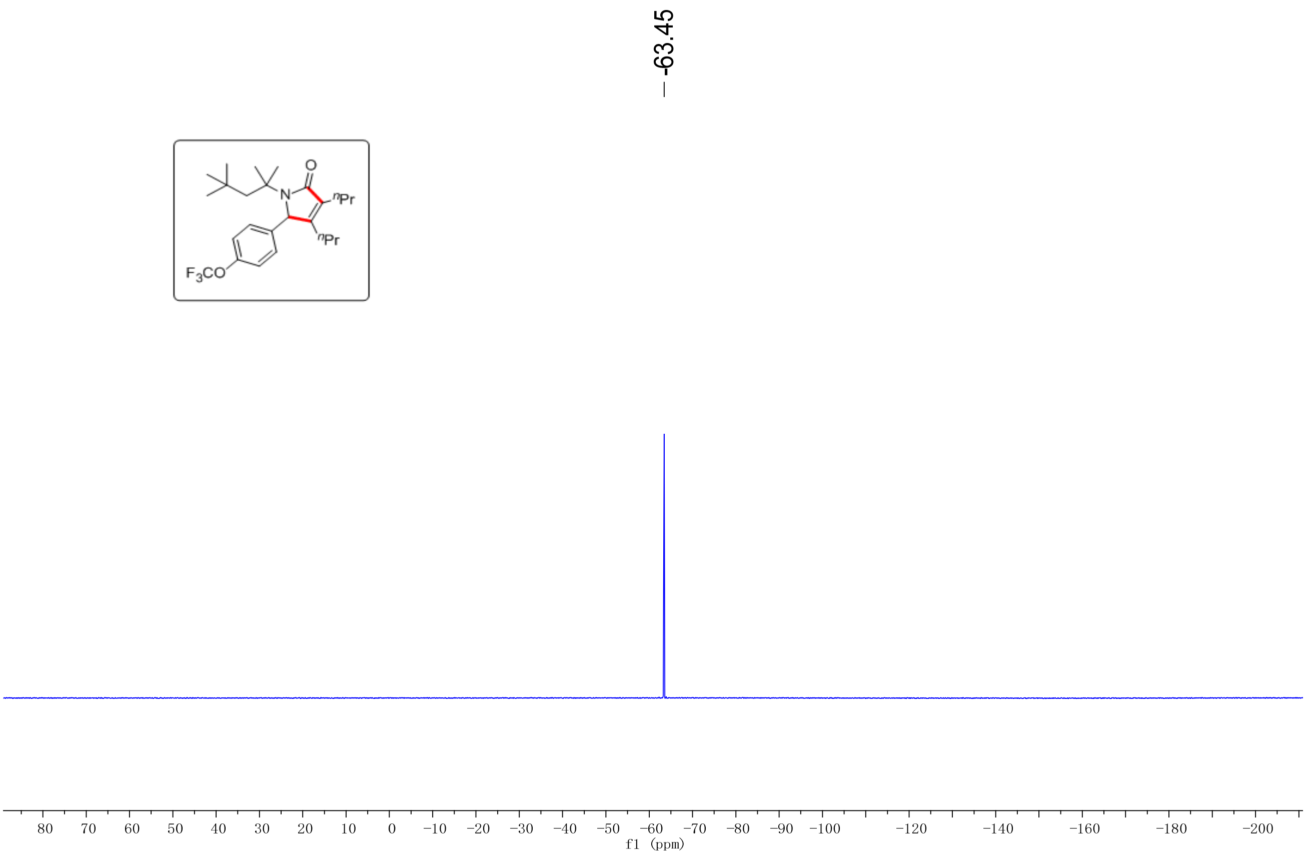

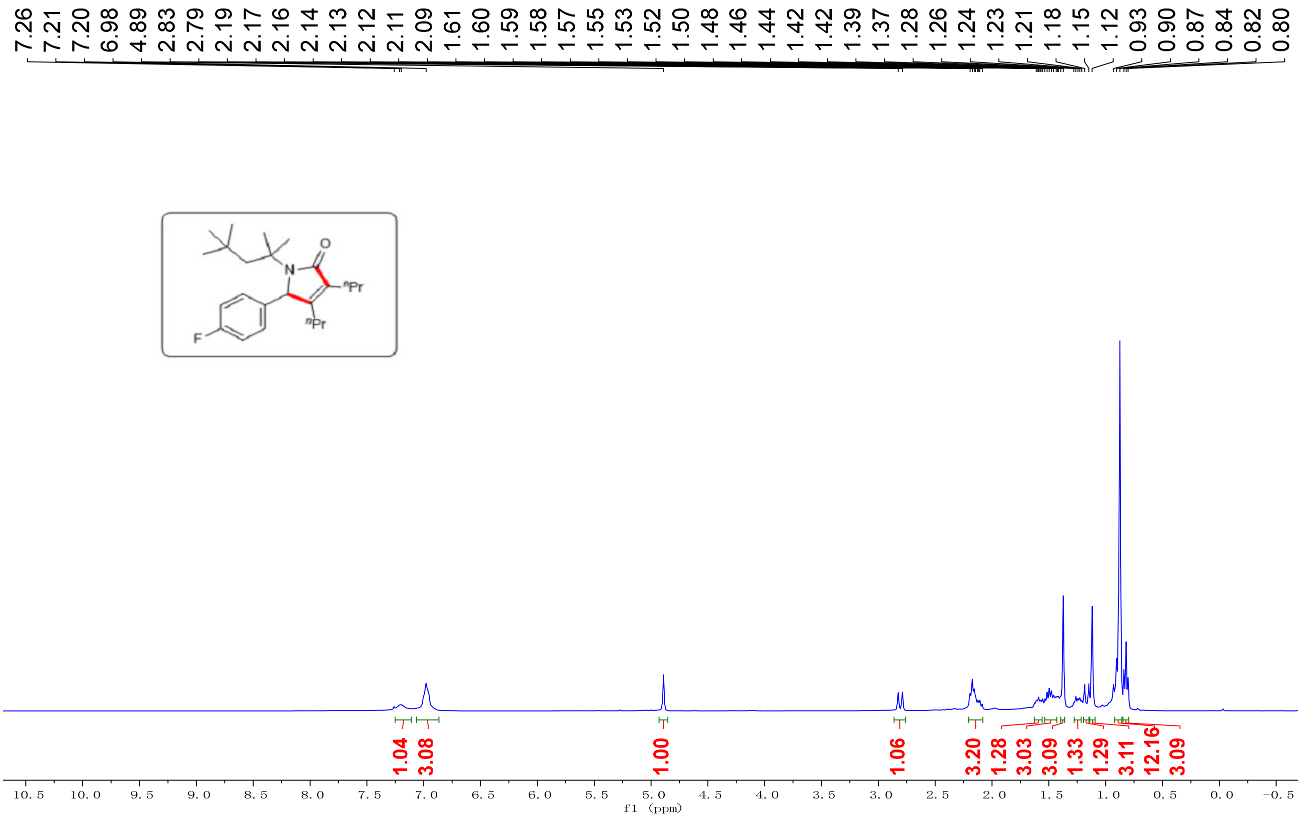

Supplementary Figure 60. ^{19}F (**3l**) and ^{1}H NMR (**3m**) spectra in $CDCl_3$

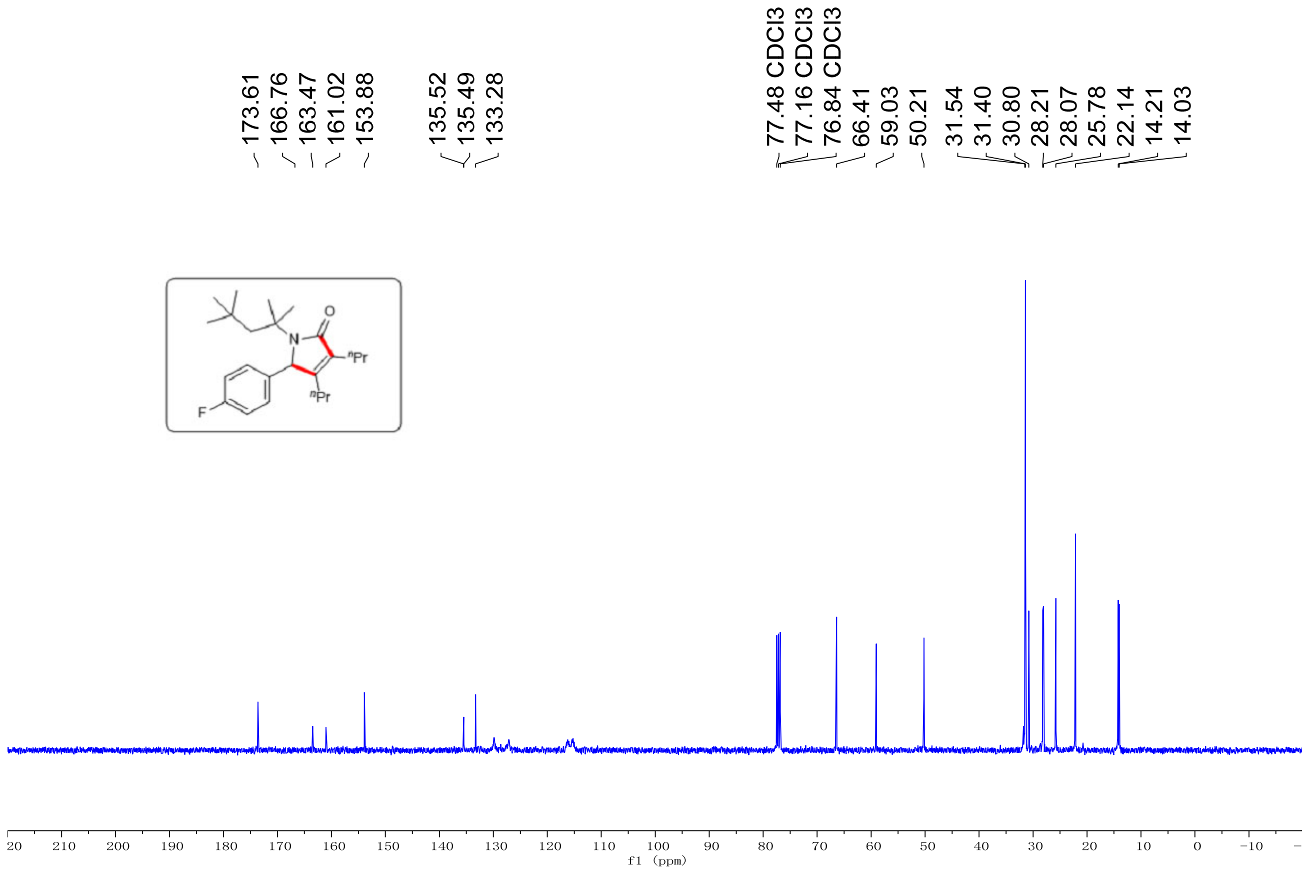

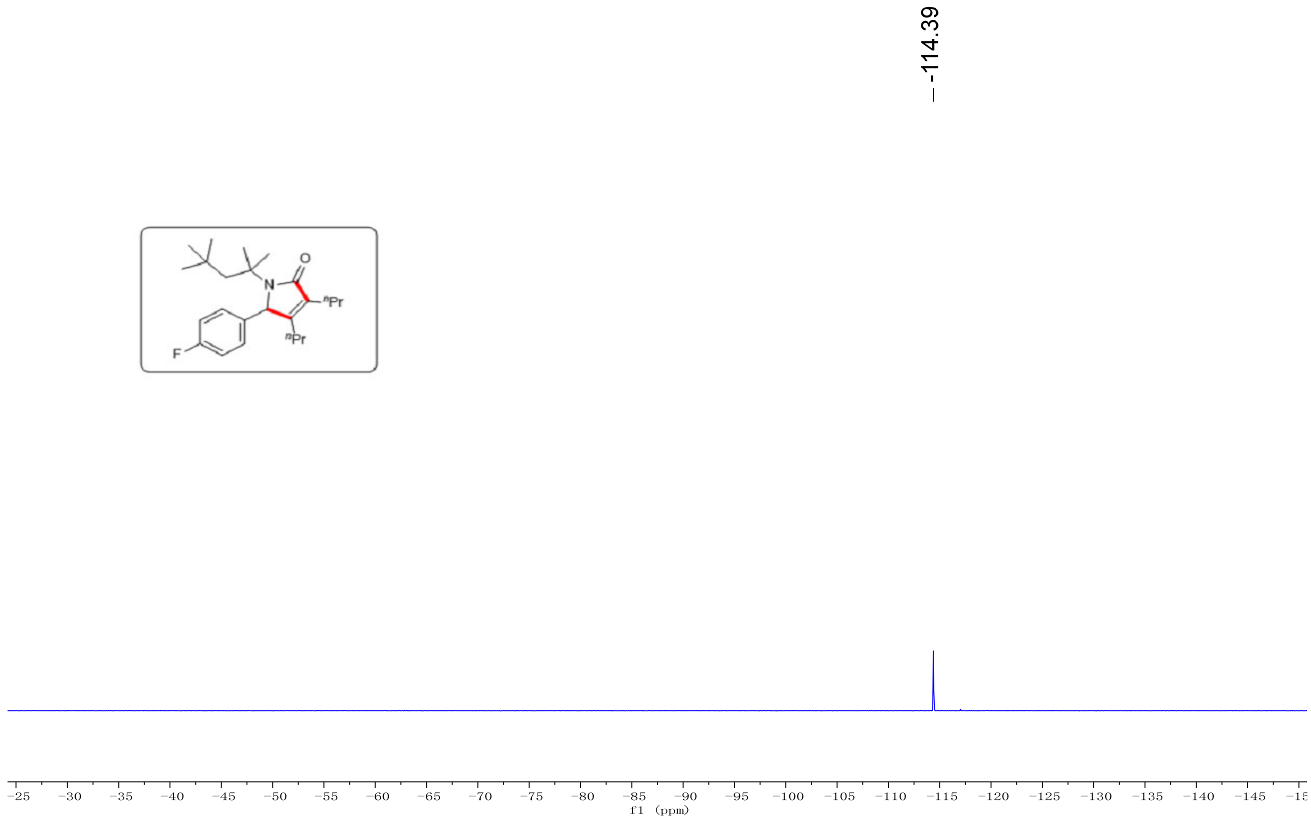

Supplementary Figure 61. ^{13}C and ^{19}F NMR spectra of compound **3m** in $CDCl_3$

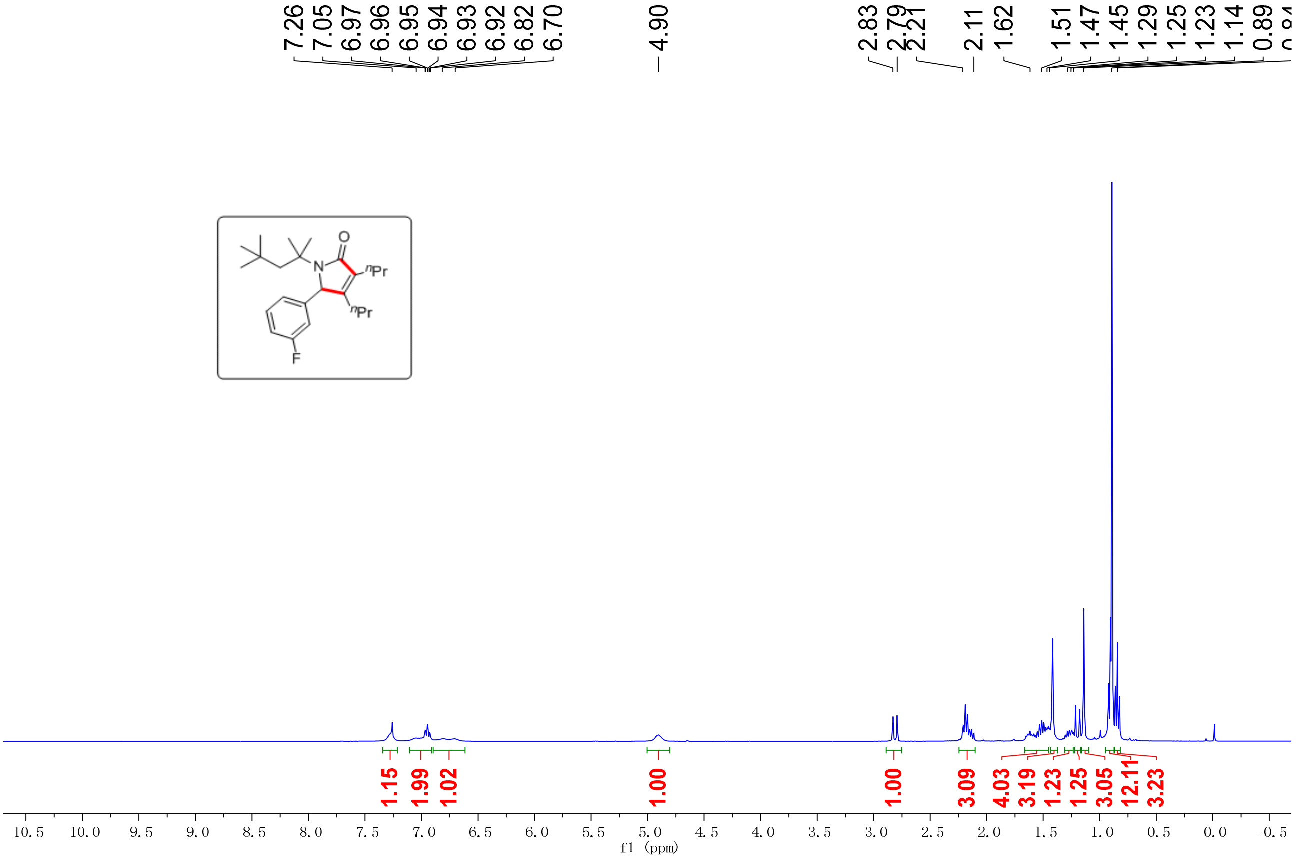

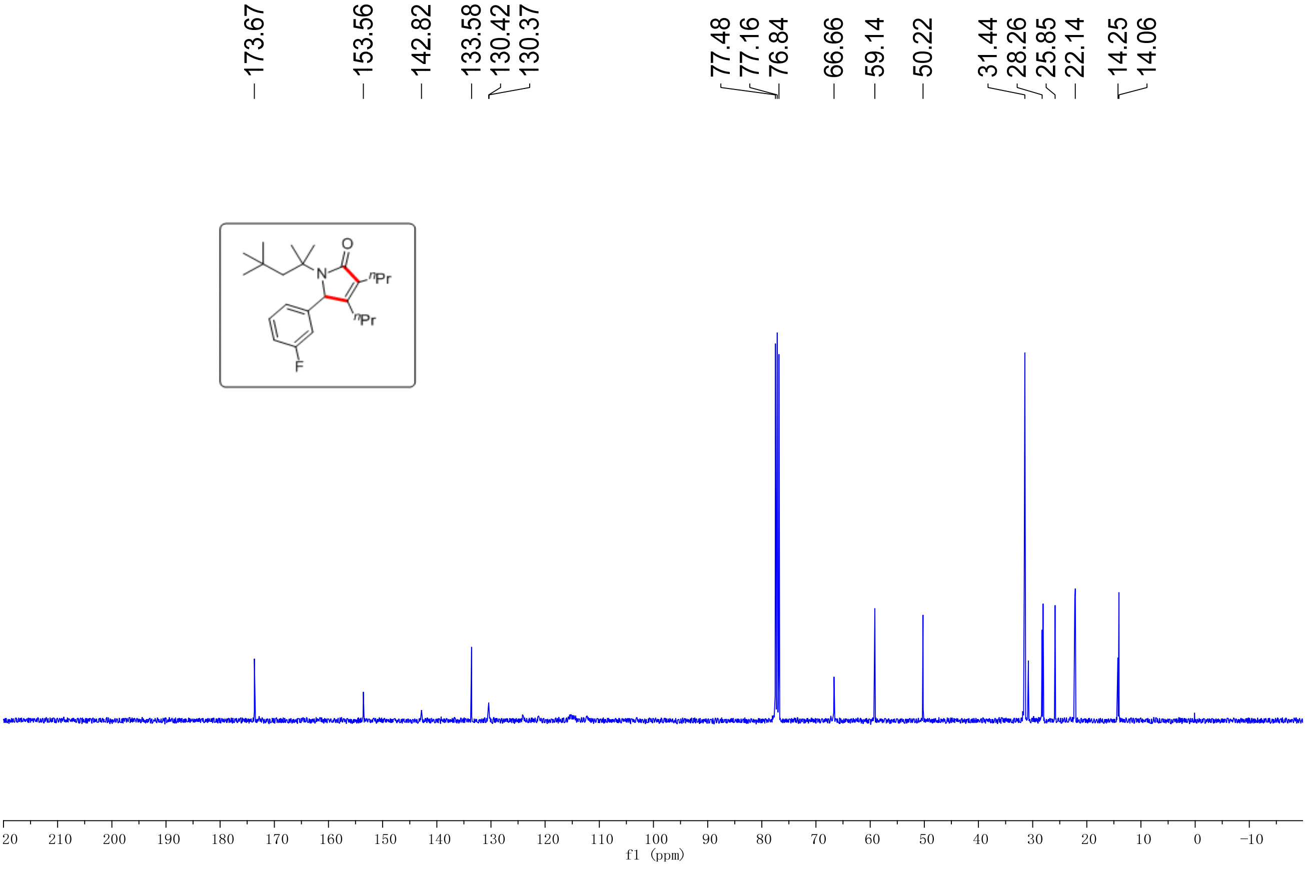

Supplementary Figure 62. ^{1}H and ^{13}C NMR spectra of compound **3n** in $CDCl_3$

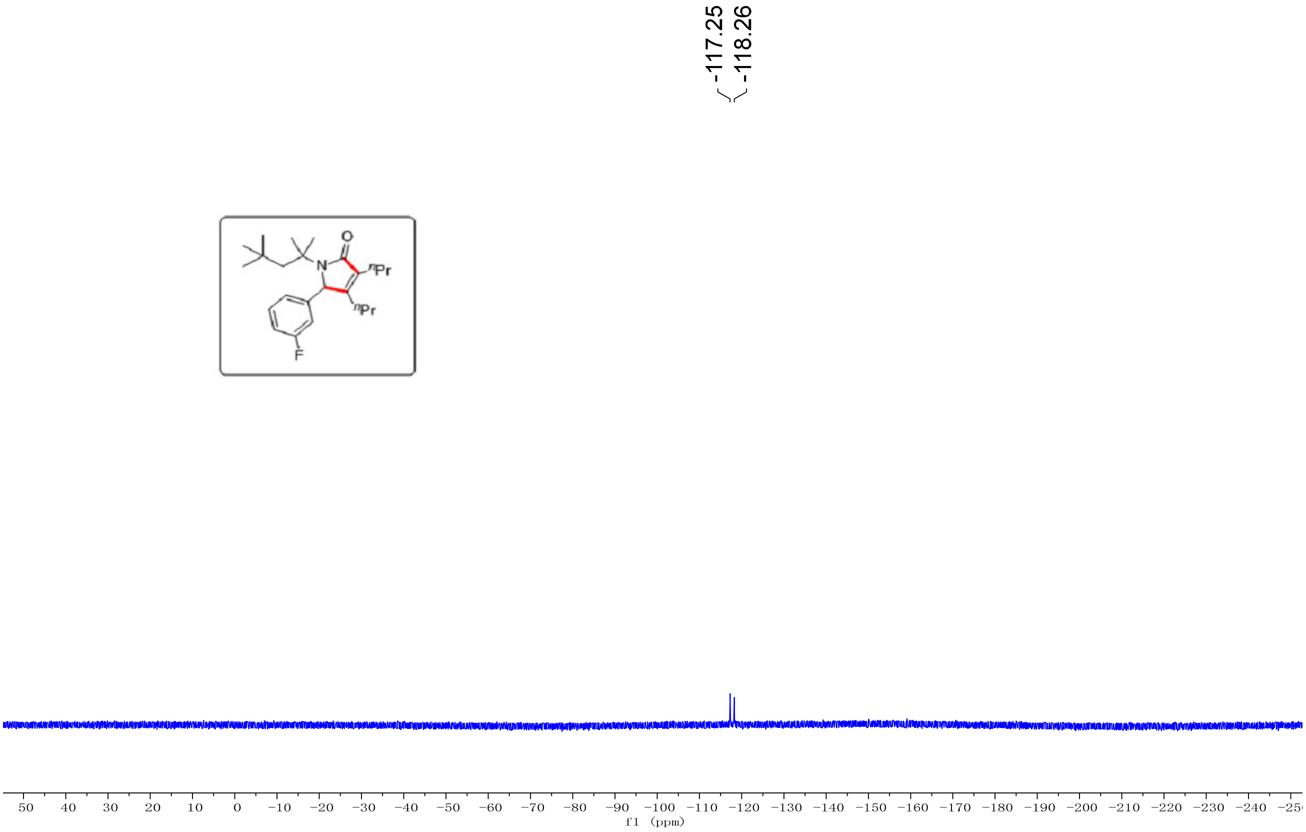

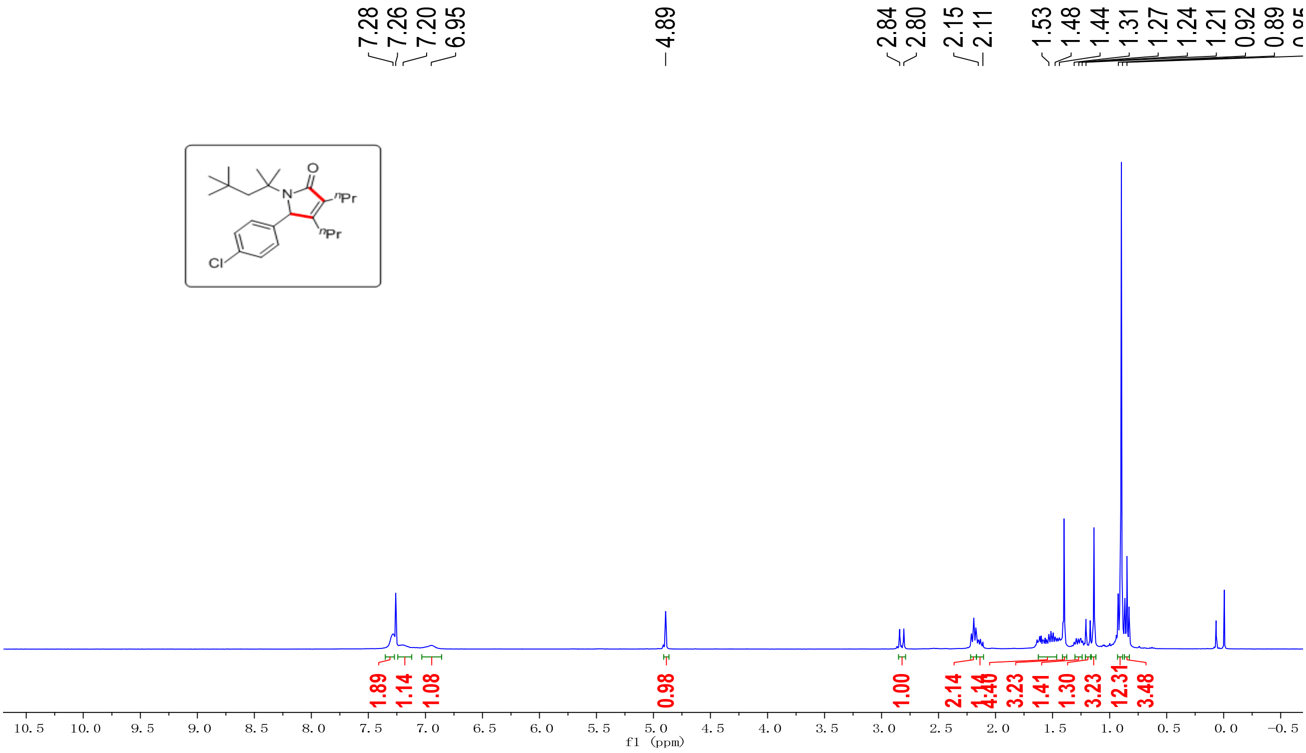

Supplementary Figure 63. ^{19}C (**3n**) and ^{1}H NMR (**3o**) spectra in $CDCl_3$

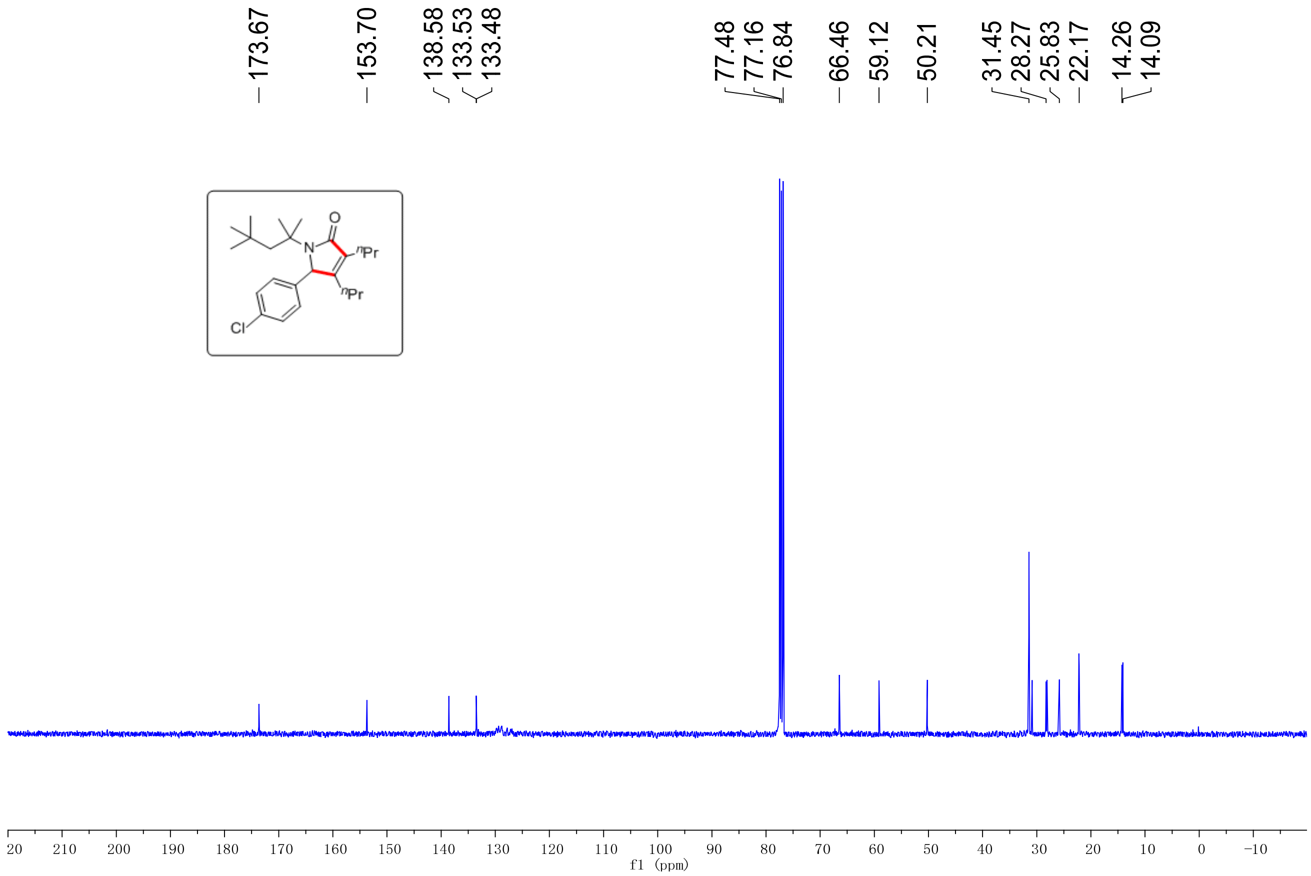

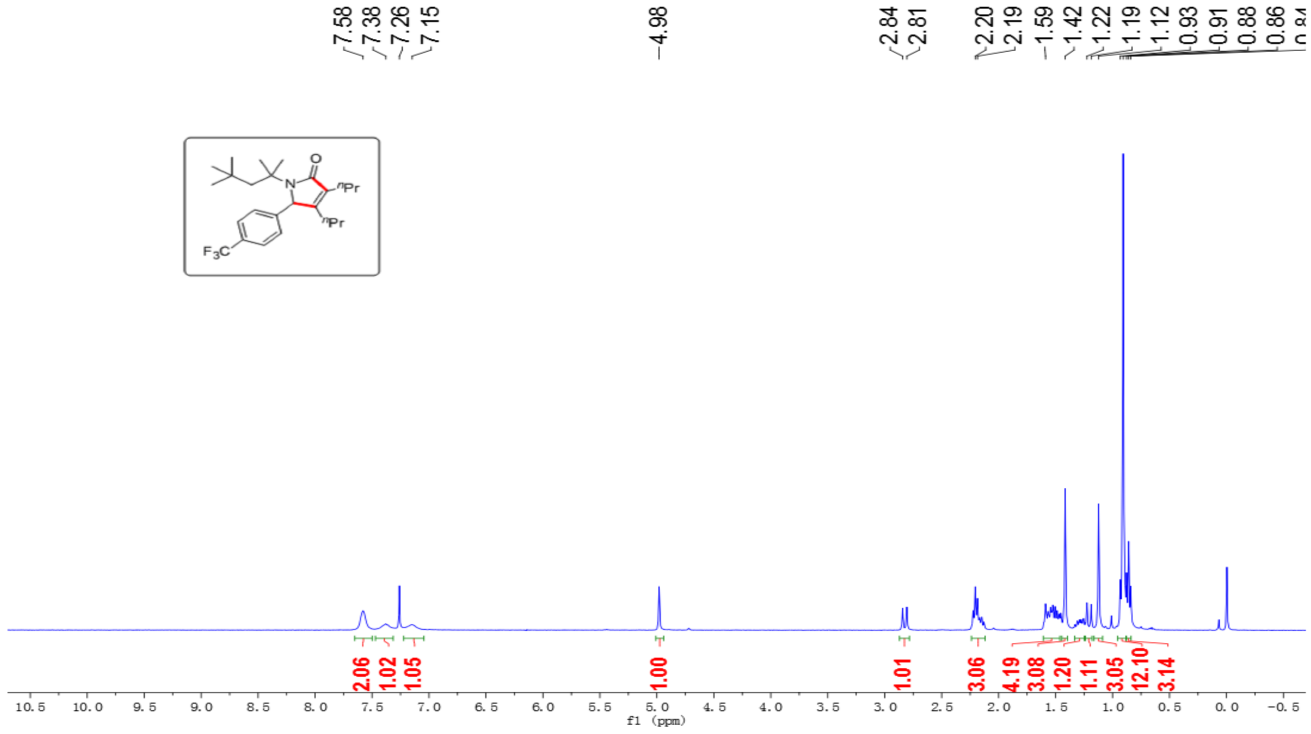

Supplementary Figure 64. ^{13}C (**3o**) and ^{1}H NMR (**3p**) spectra in $CDCl_3$

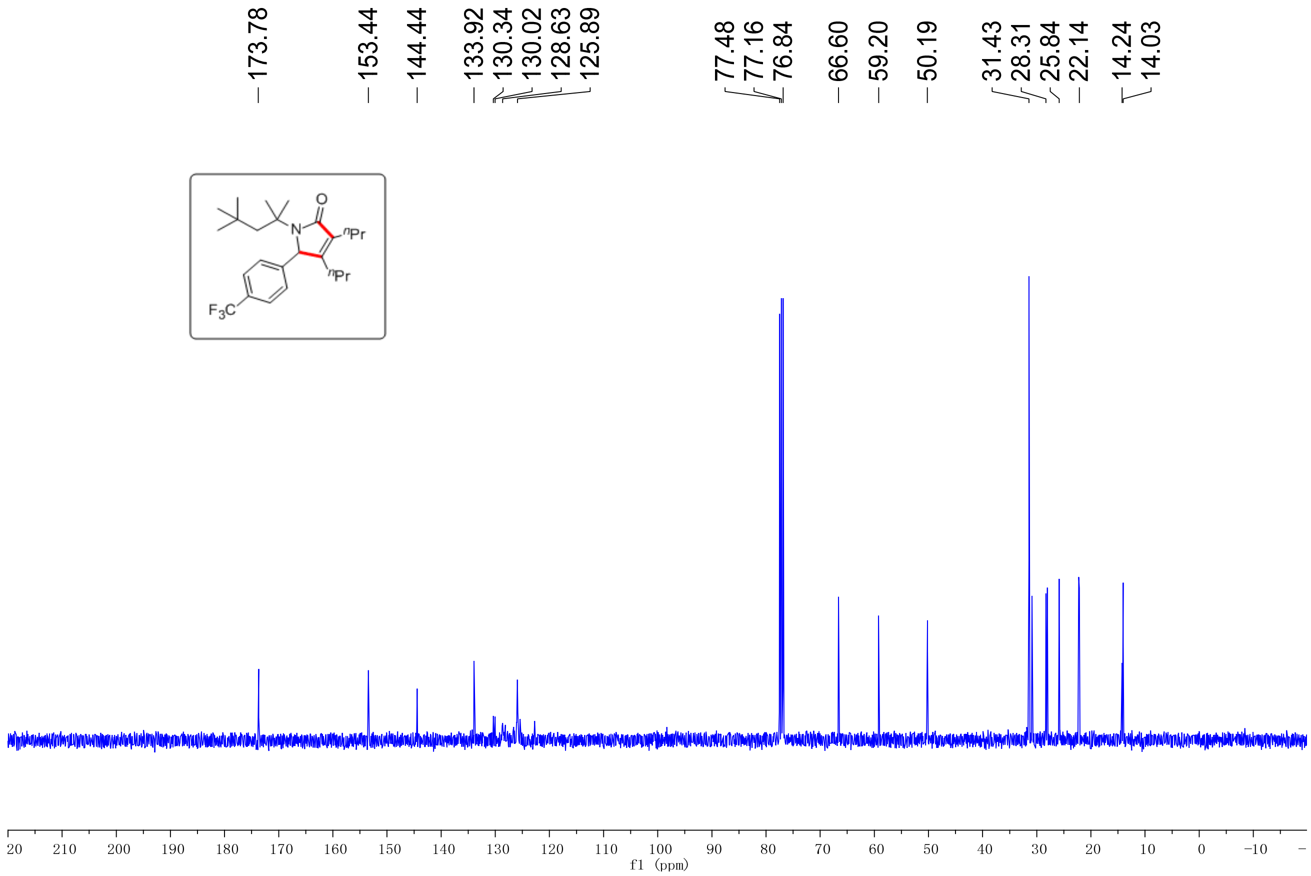

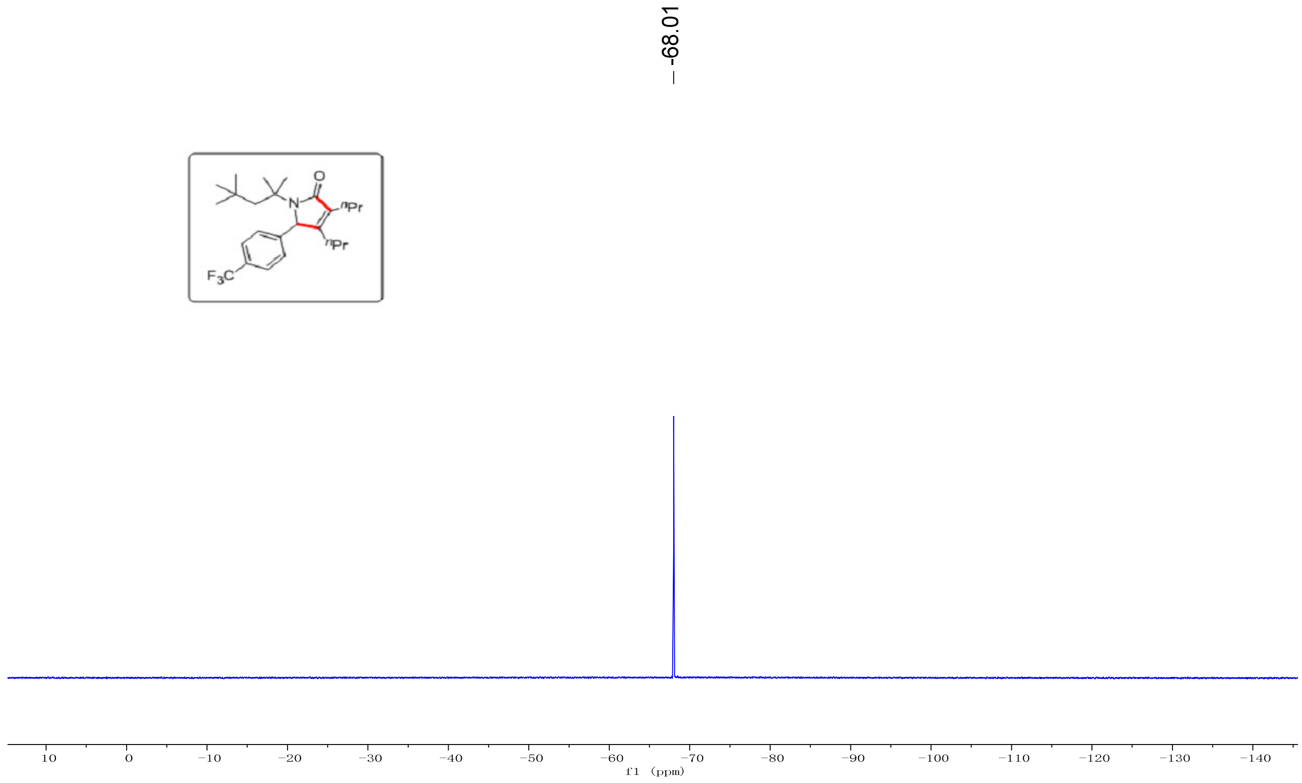

Supplementary Figure 65. ^{13}C and ^{19}F NMR spectra of compound **3p** in $CDCl_3$

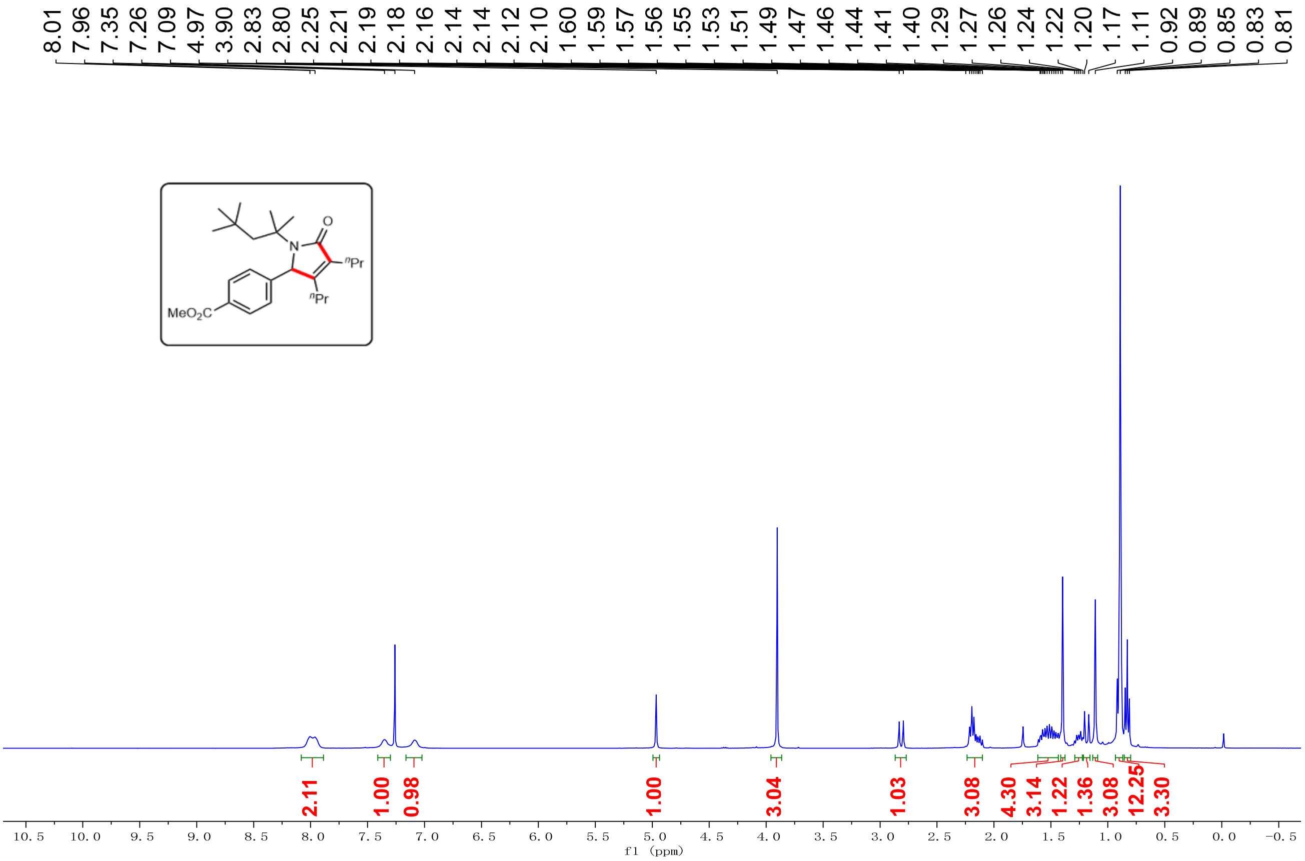

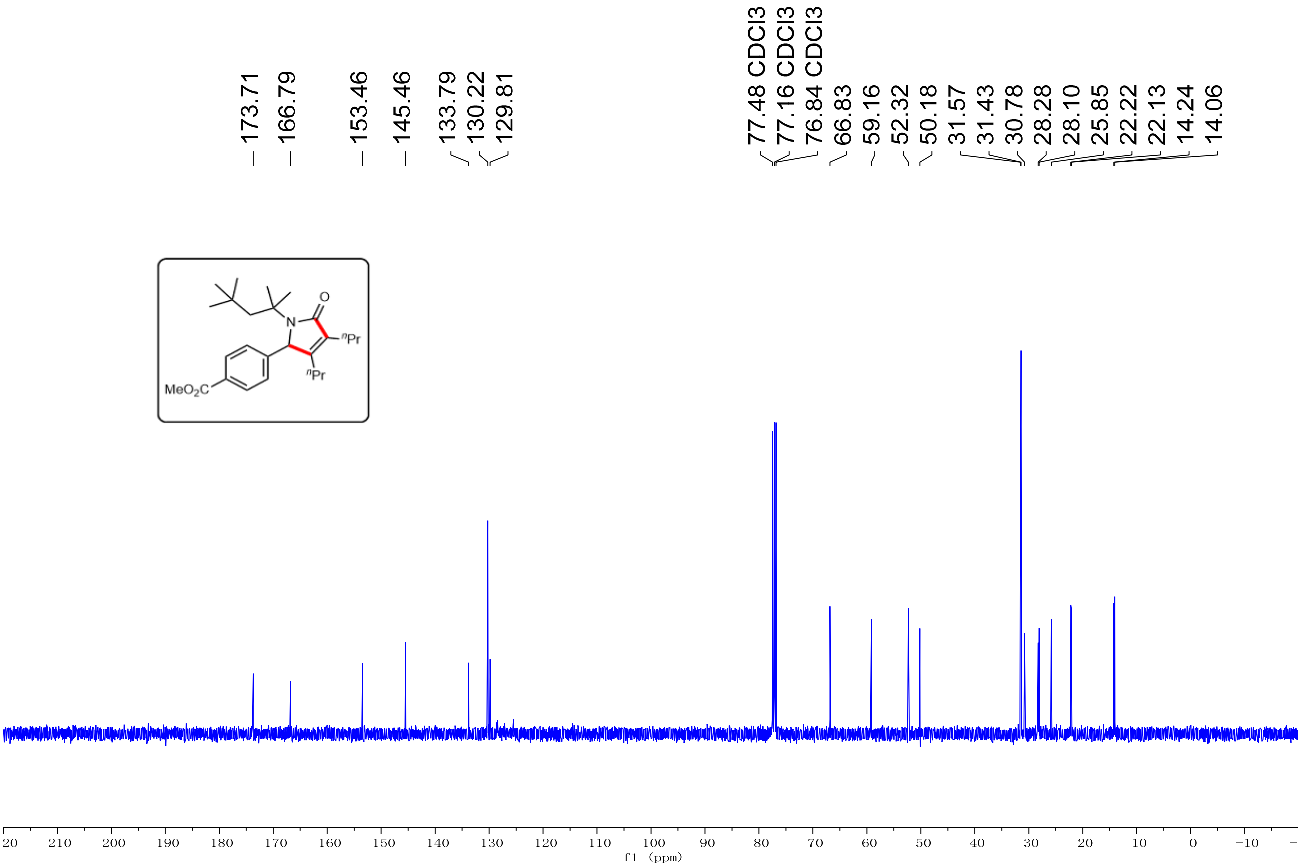

Supplementary Figure 66. ^{1}H and ^{13}C NMR spectra of compound **3q** in $CDCl_3$

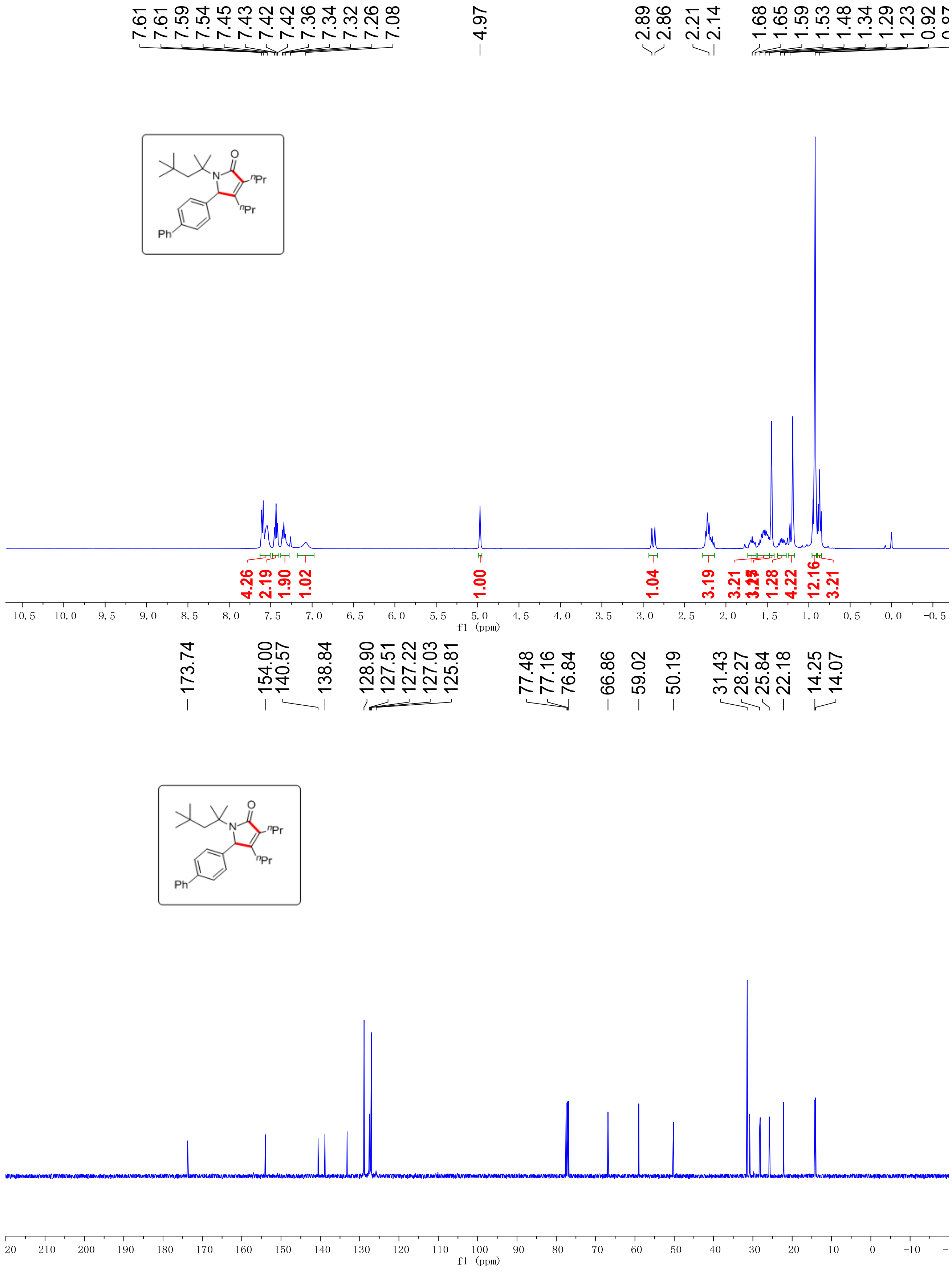

Supplementary Figure 67. ^{1}H and ^{13}C NMR spectra of compound **3r** in $CDCl_3$

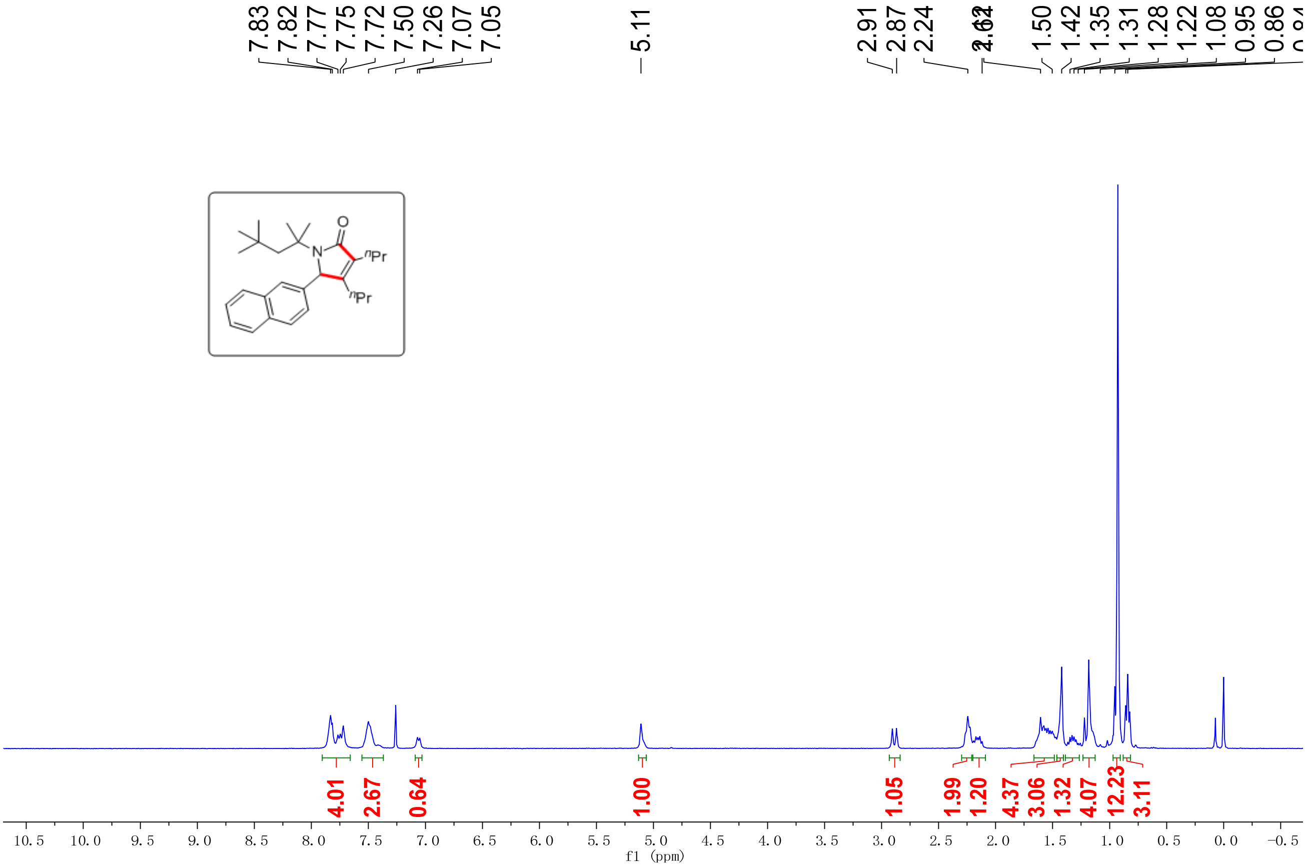

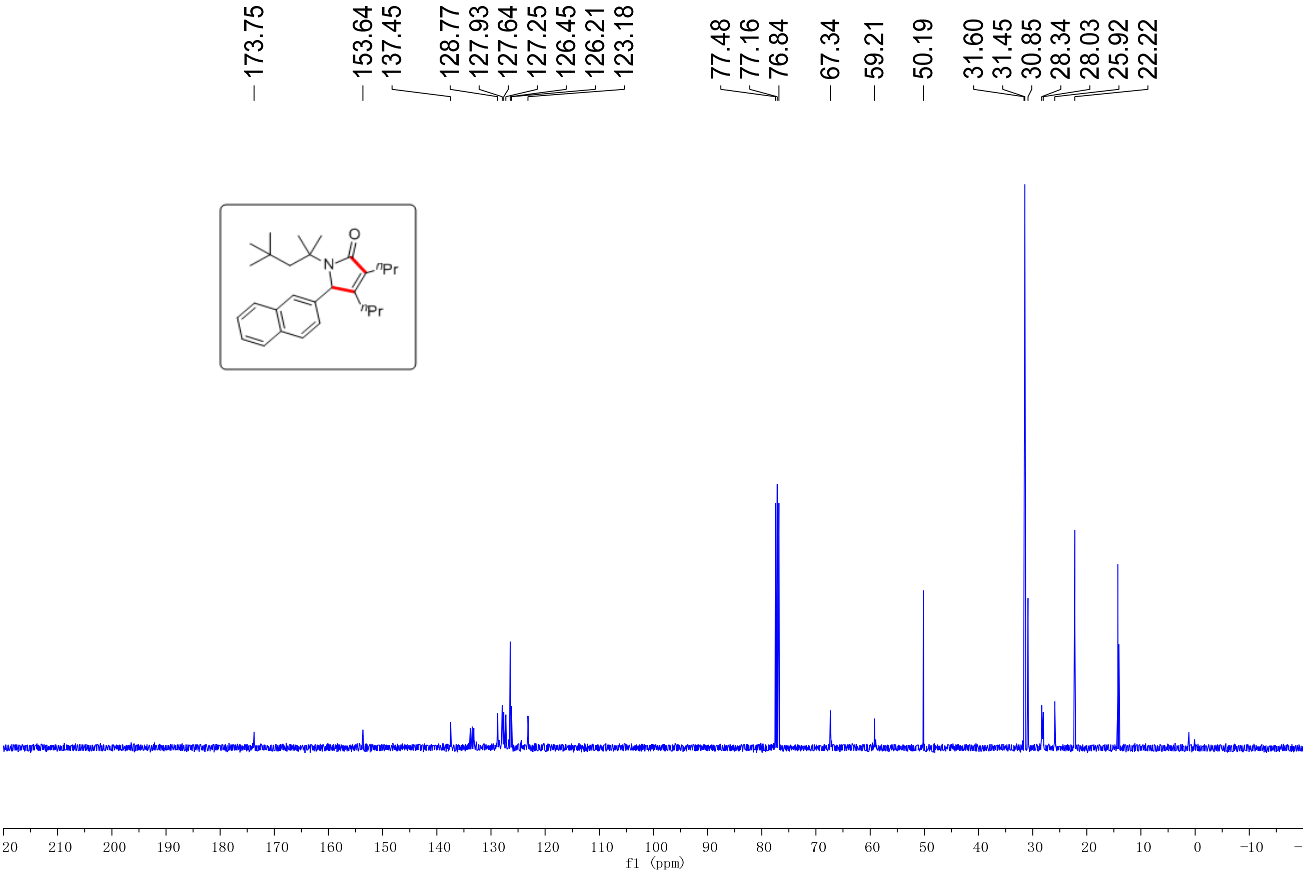

Supplementary Figure 68. ^{1}H and ^{13}C NMR spectra of compound **3s** in $CDCl_3$

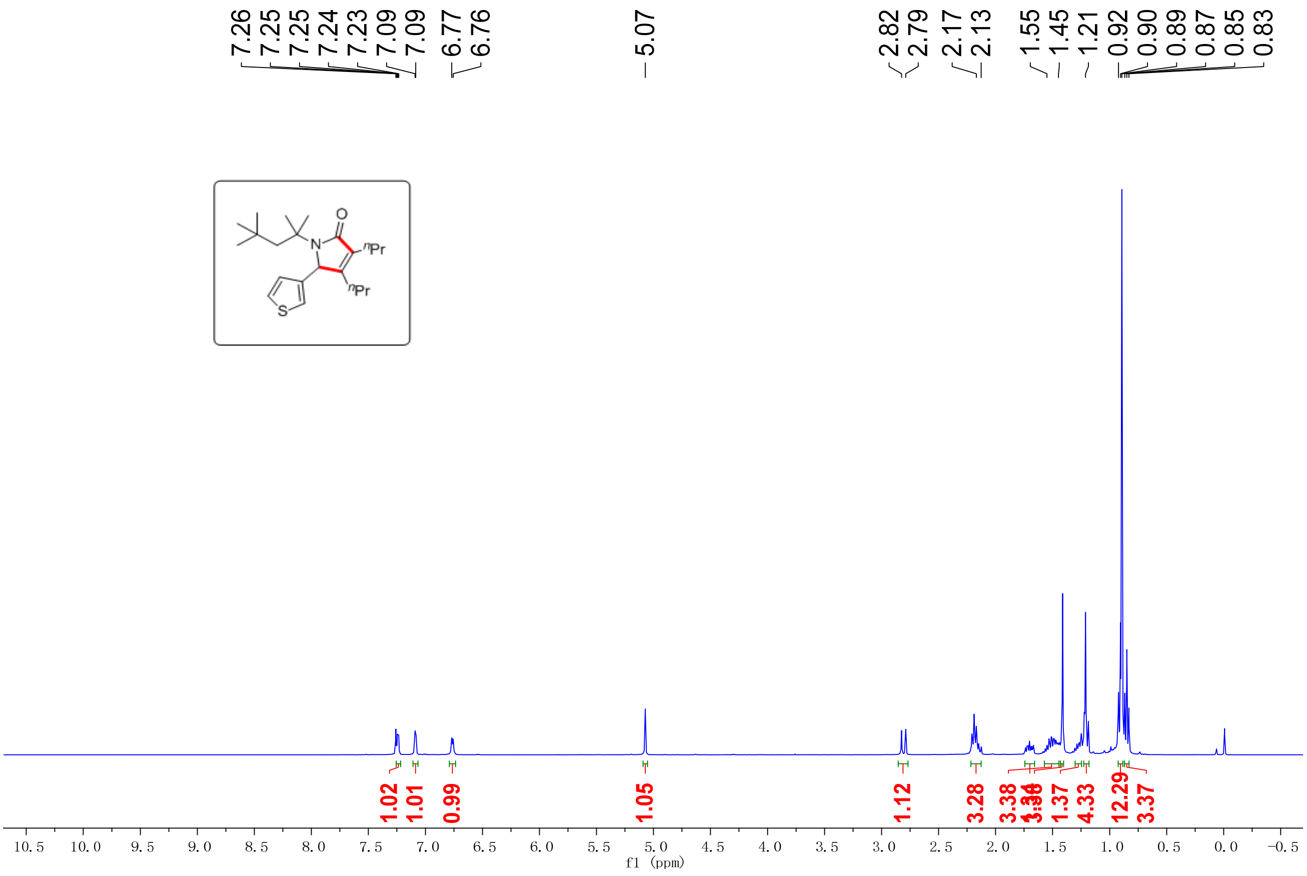

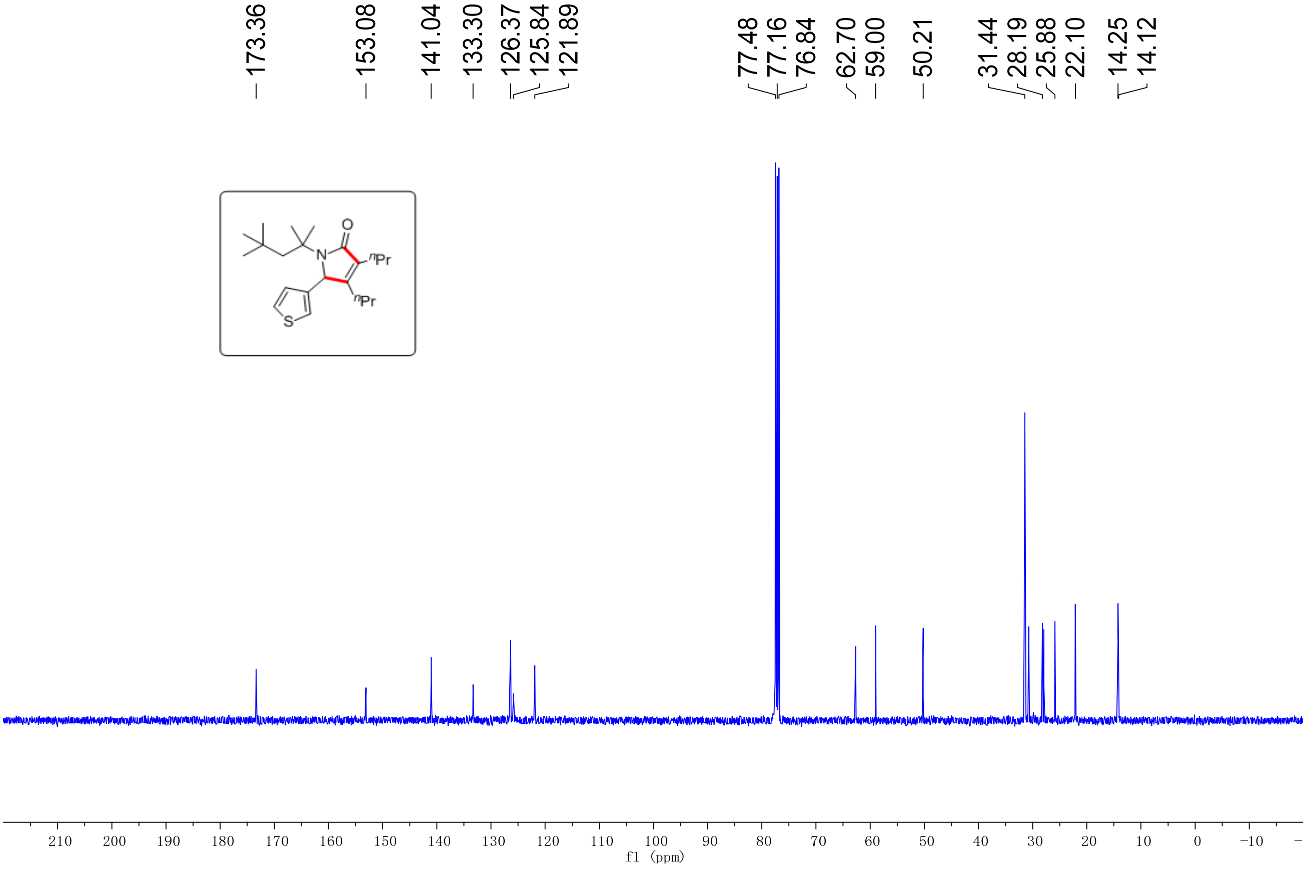

Supplementary Figure 69. ^{1}H and ^{13}C NMR spectra of compound **3t** in $CDCl_3$

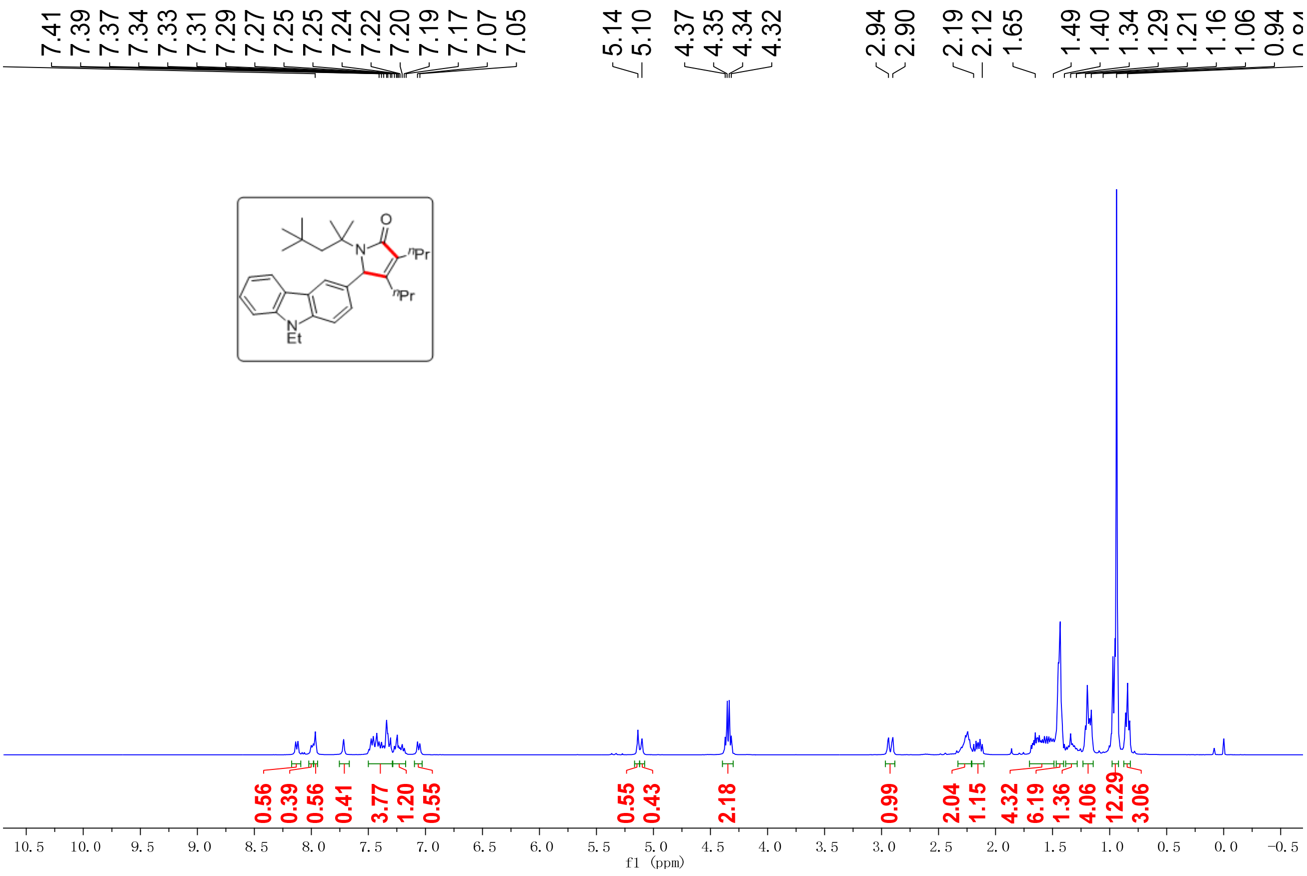

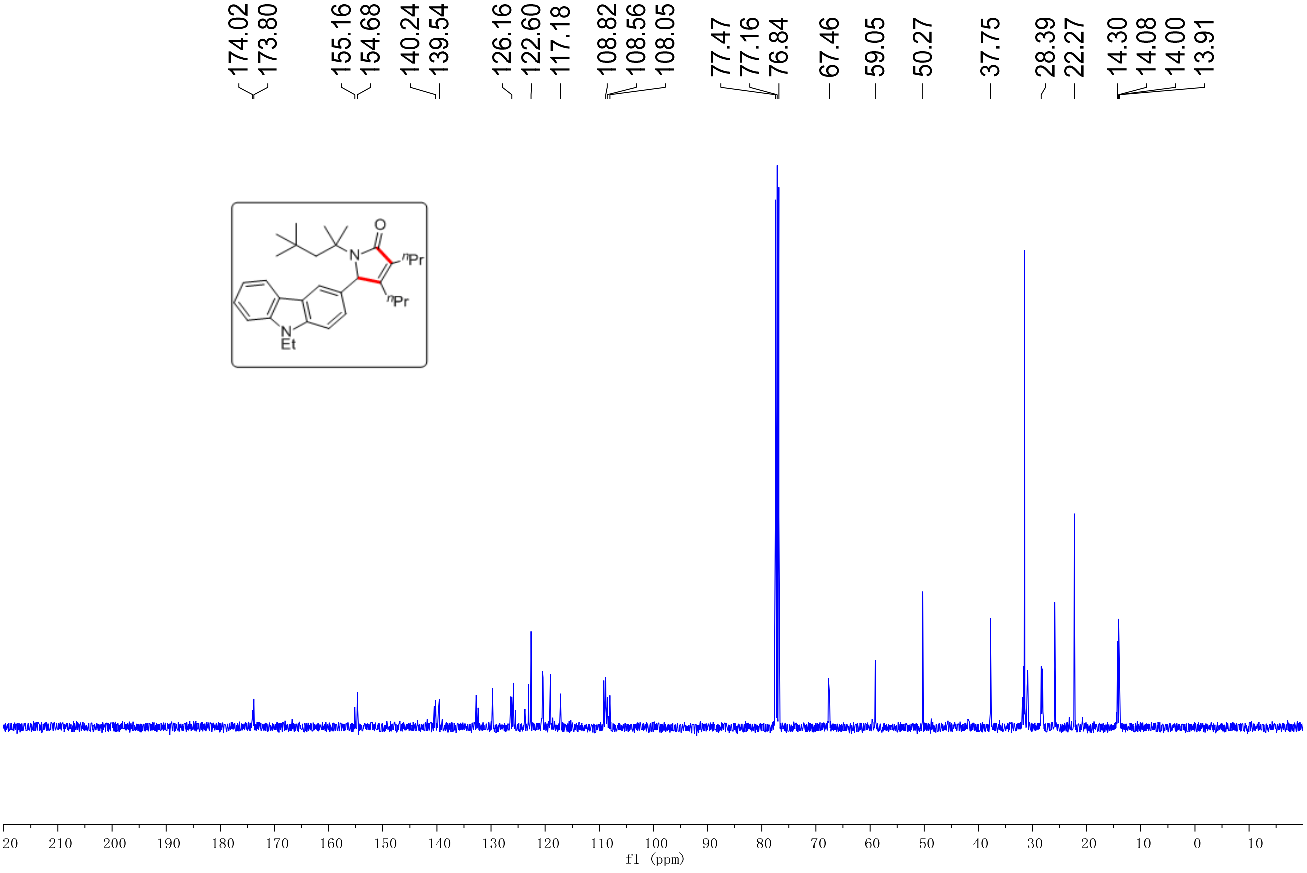

Supplementary Figure 70. ^{1}H and ^{13}C NMR spectra of compound **3u** in $CDCl_3$

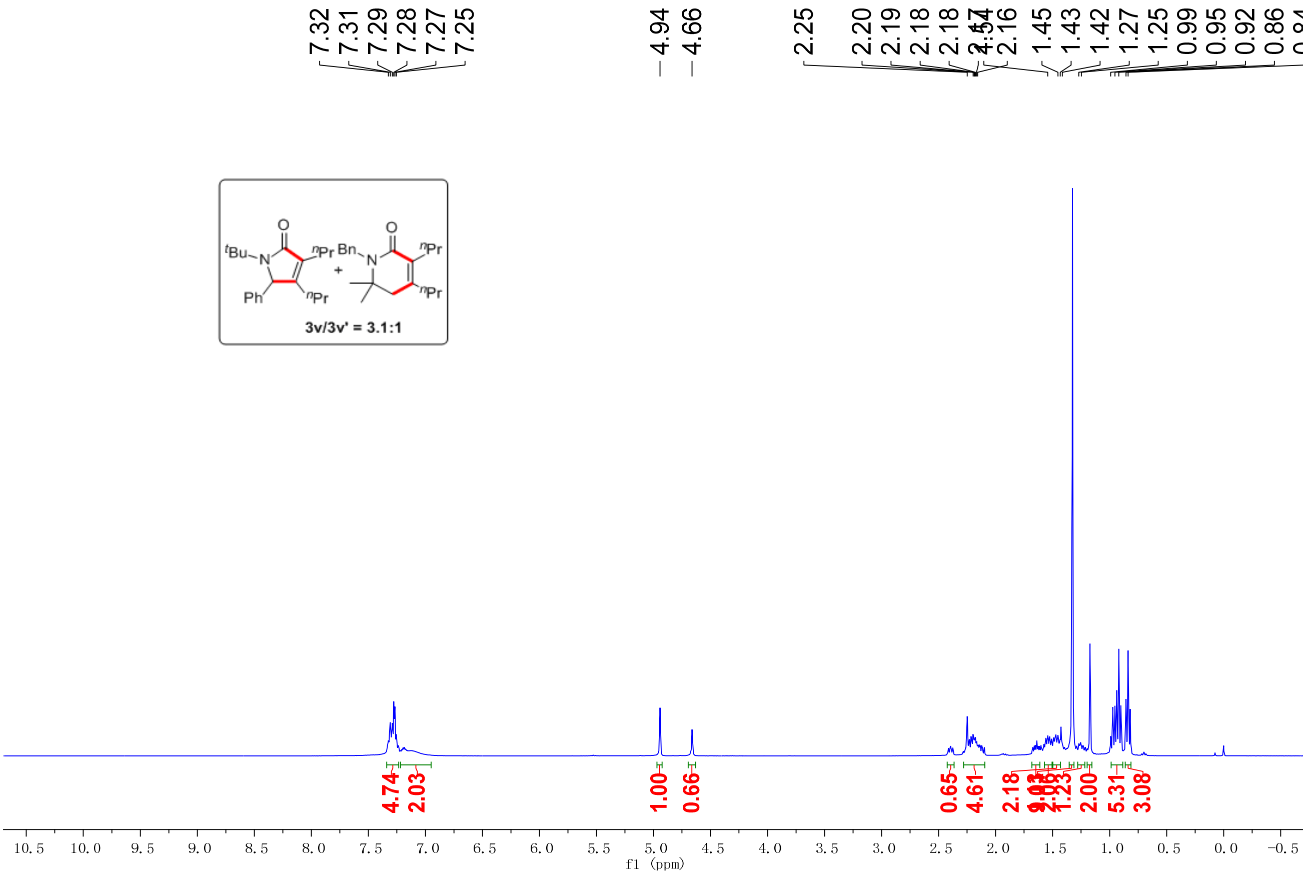

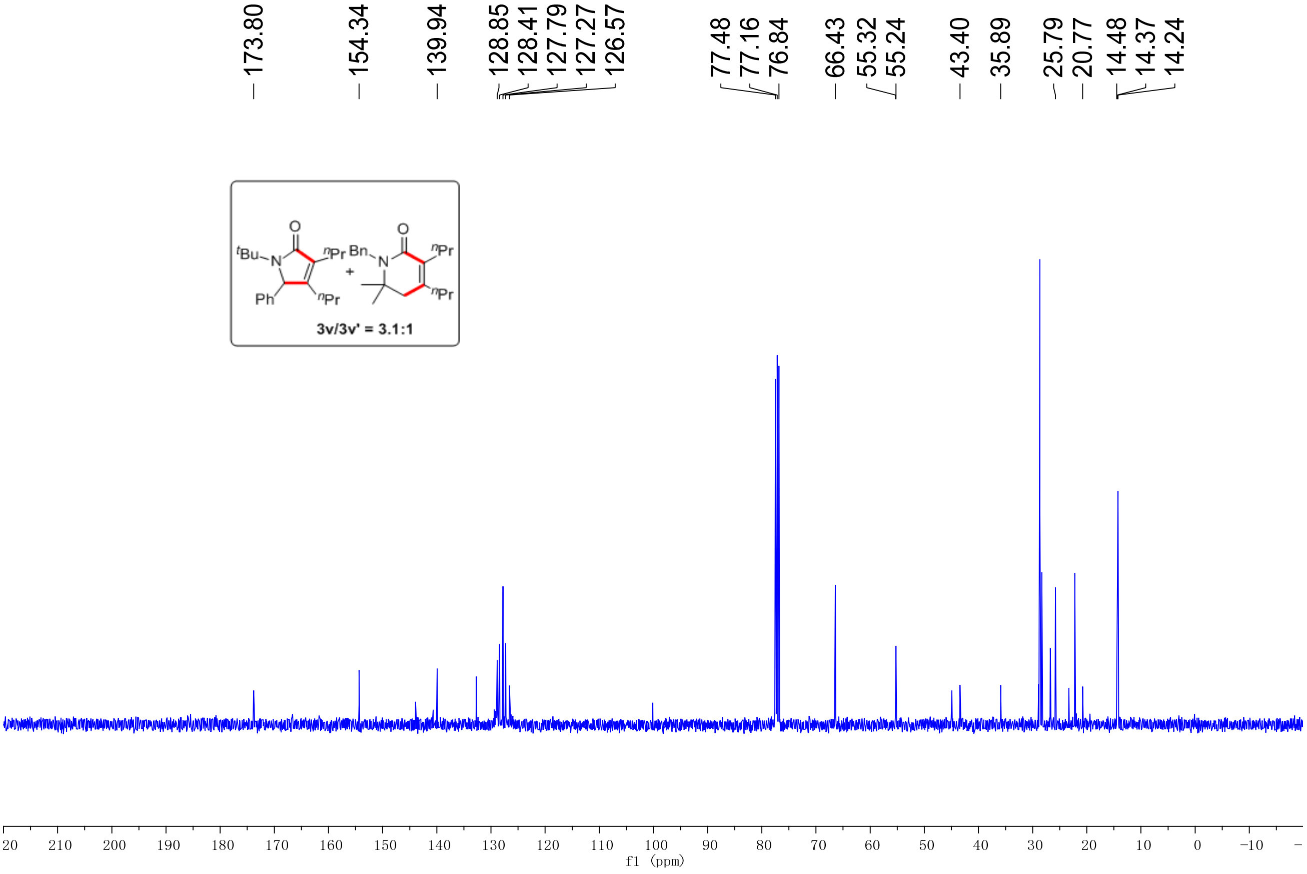

Supplementary Figure 71. ^{1}H and ^{13}C NMR spectra of compound **3v/3v′** in $CDCl_3$

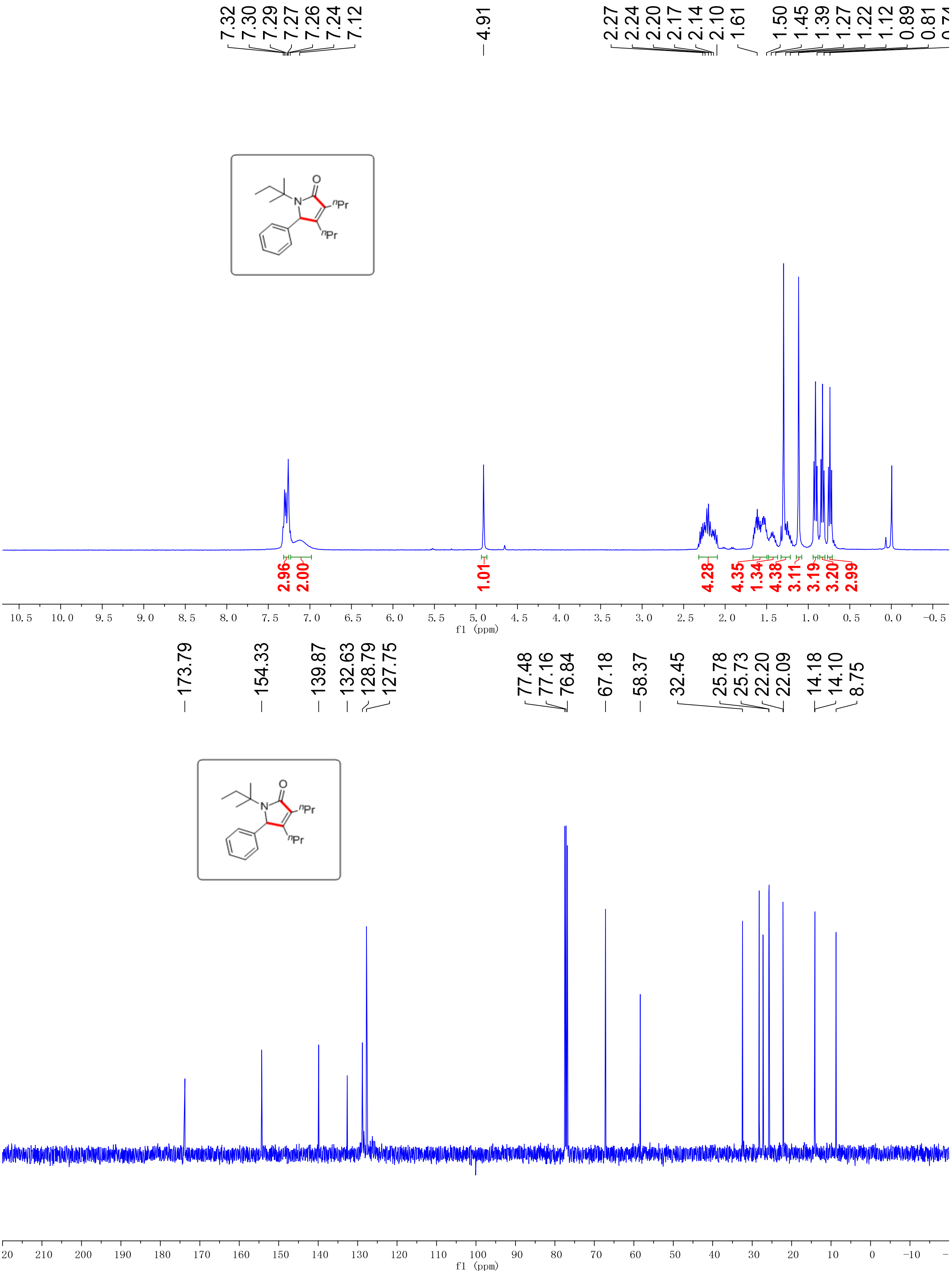

Supplementary Figure 72. ^{1}H and ^{13}C NMR spectra of compound **3w** in $CDCl_3$

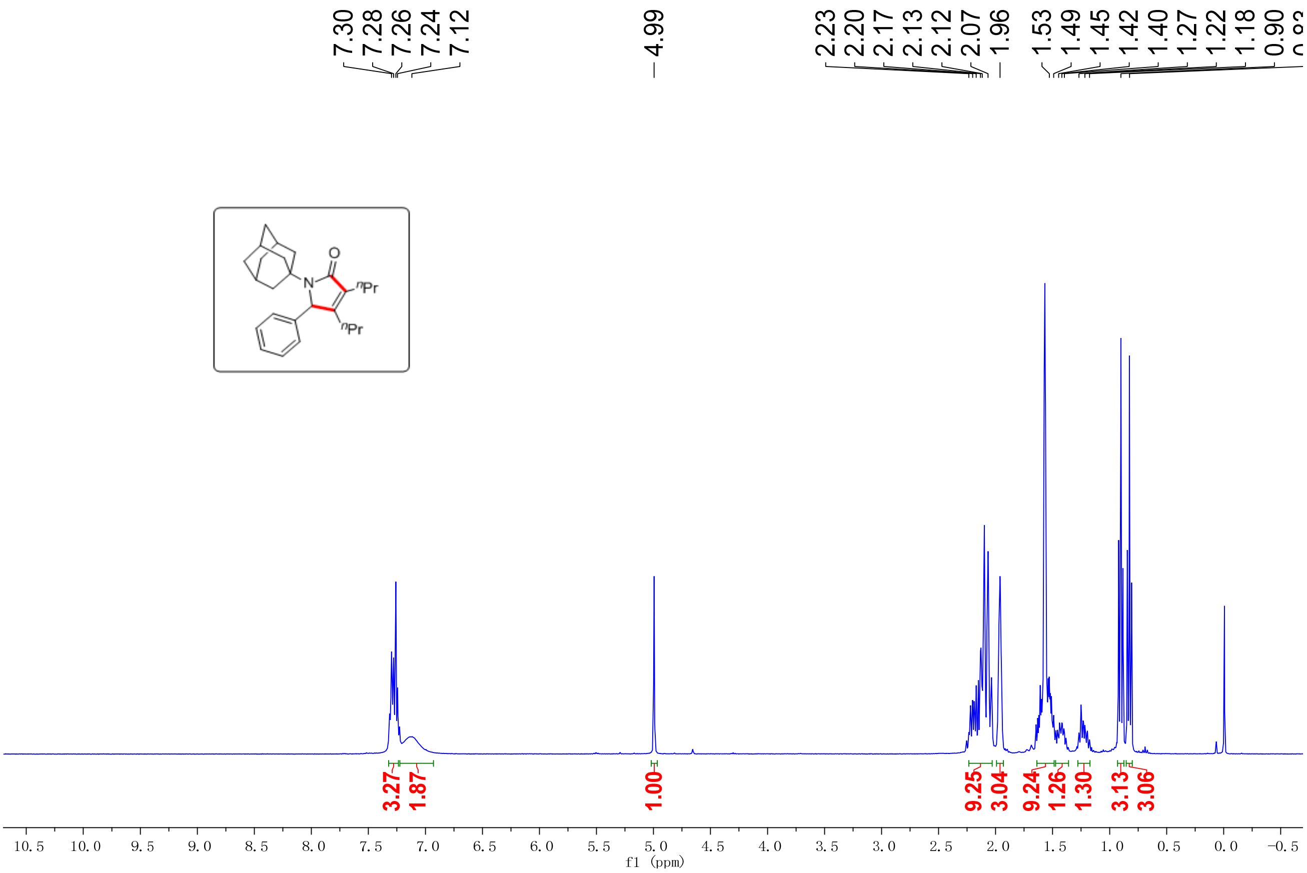

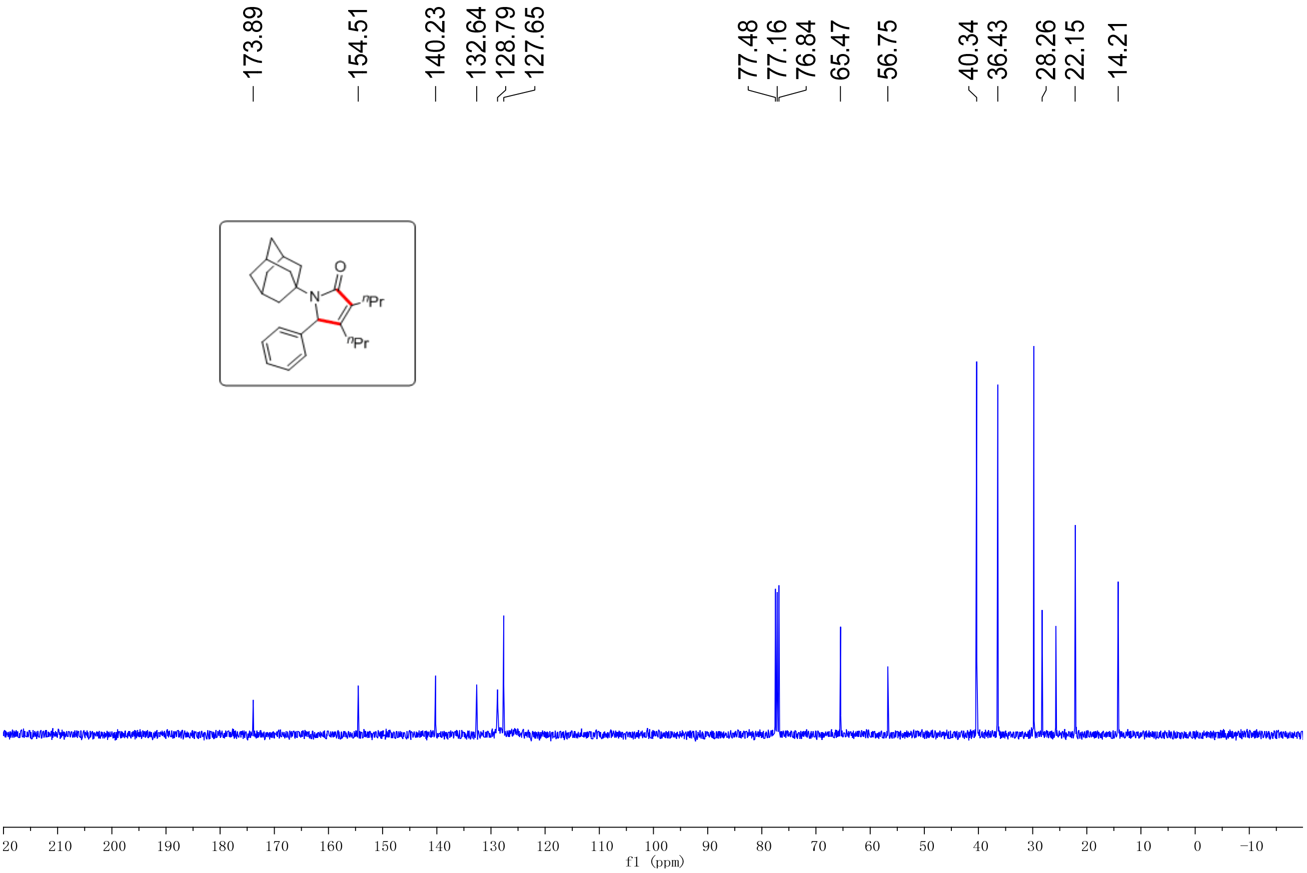

Supplementary Figure 73. ^{1}H and ^{13}C NMR spectra of compound **3x** in $CDCl_3$

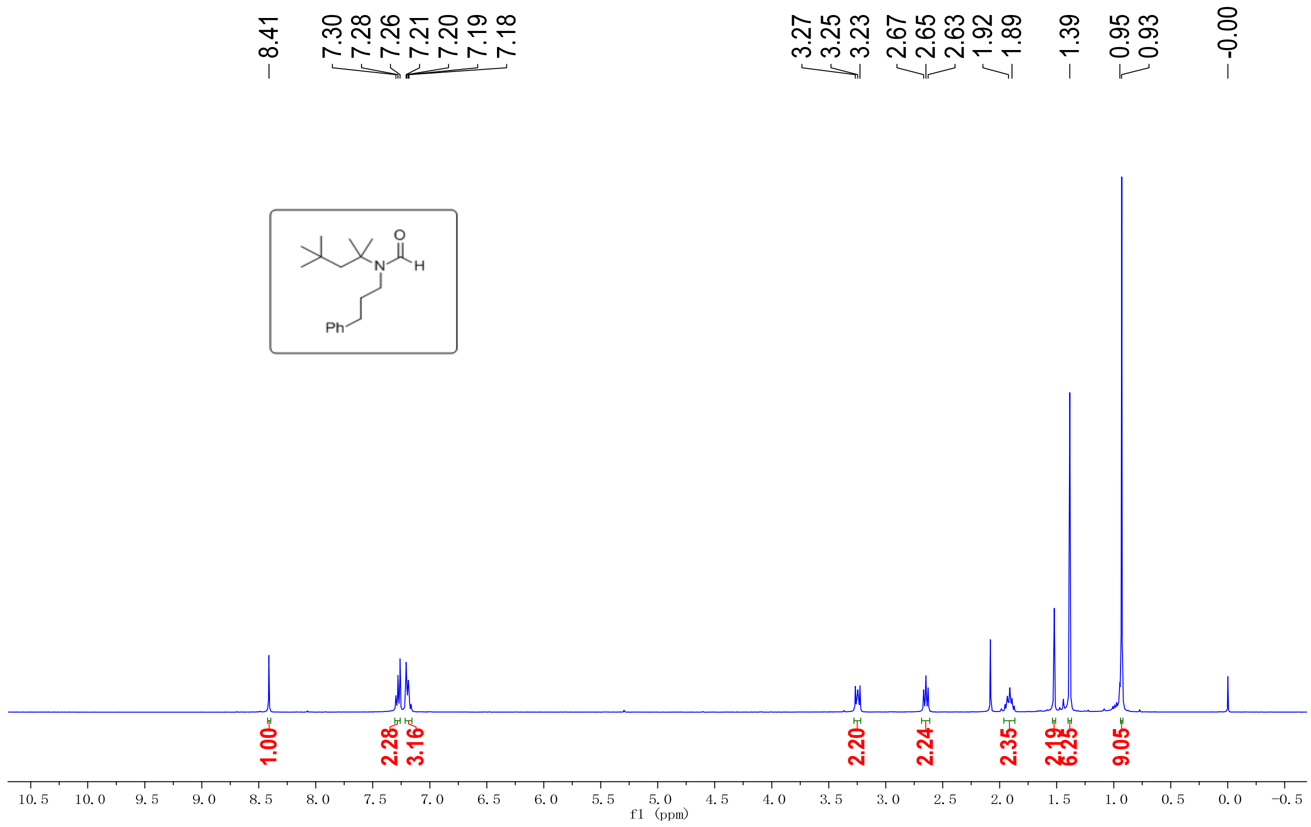

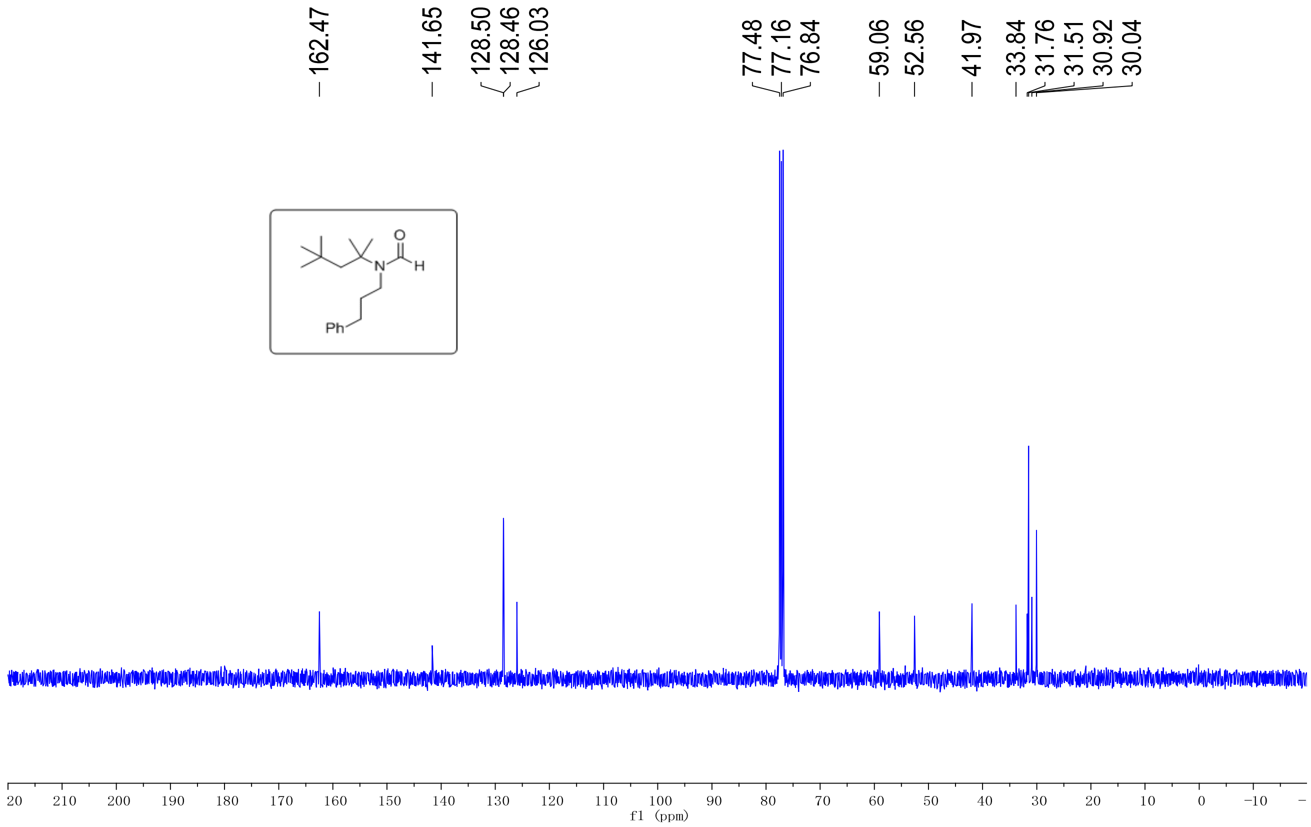

Supplementary Figure 74. ^{1}H and ^{13}C NMR spectra of compound **1y** in $CDCl_3$

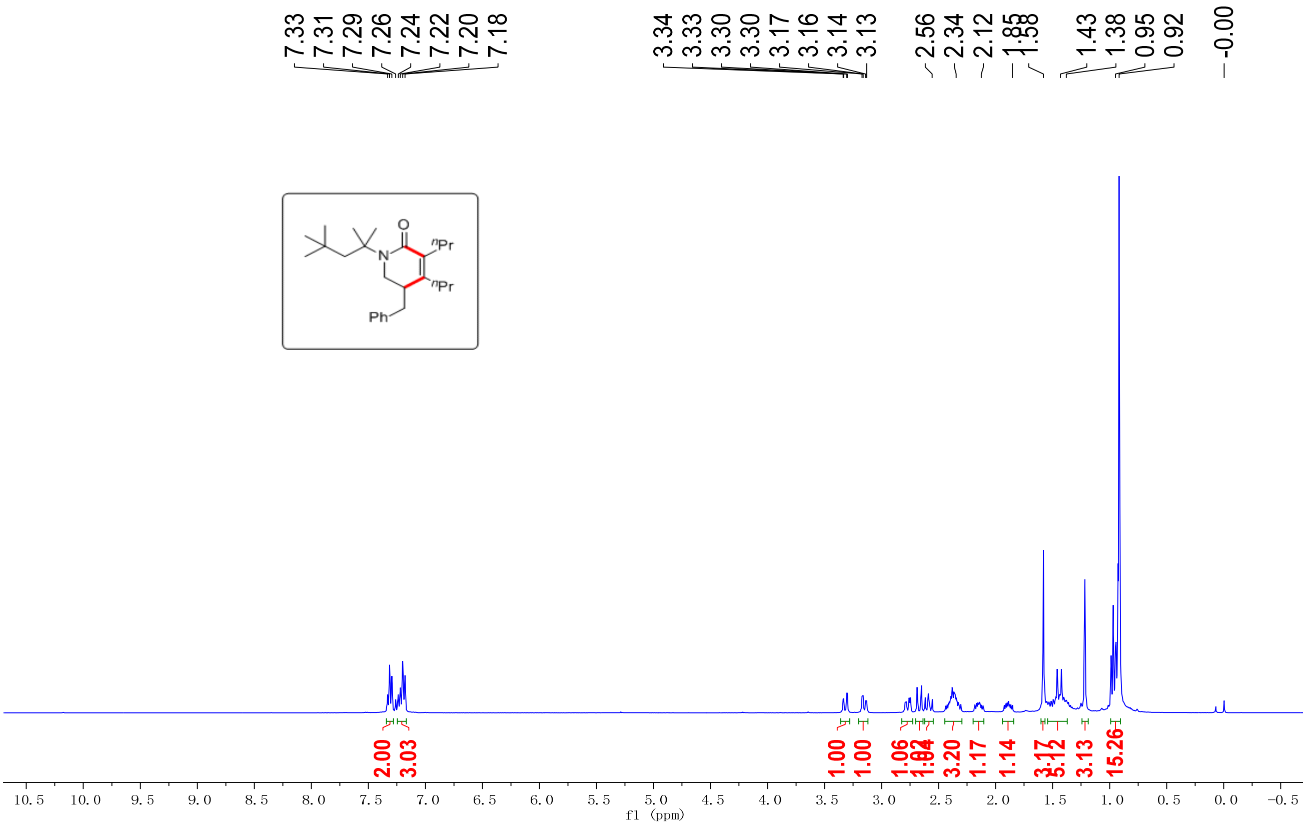

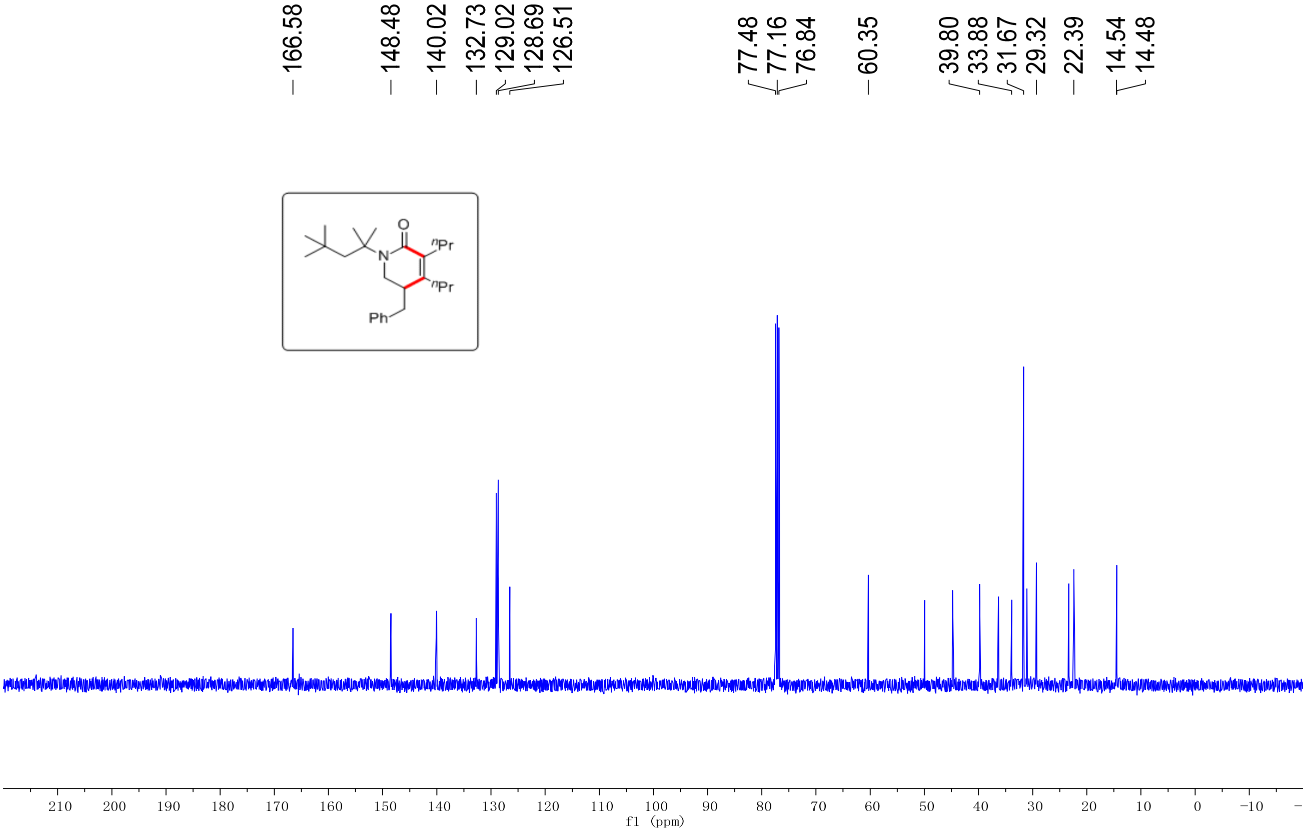

Supplementary Figure 75. ^{1}H and ^{13}C NMR spectrum of compound **3y″** in $CDCl_3$

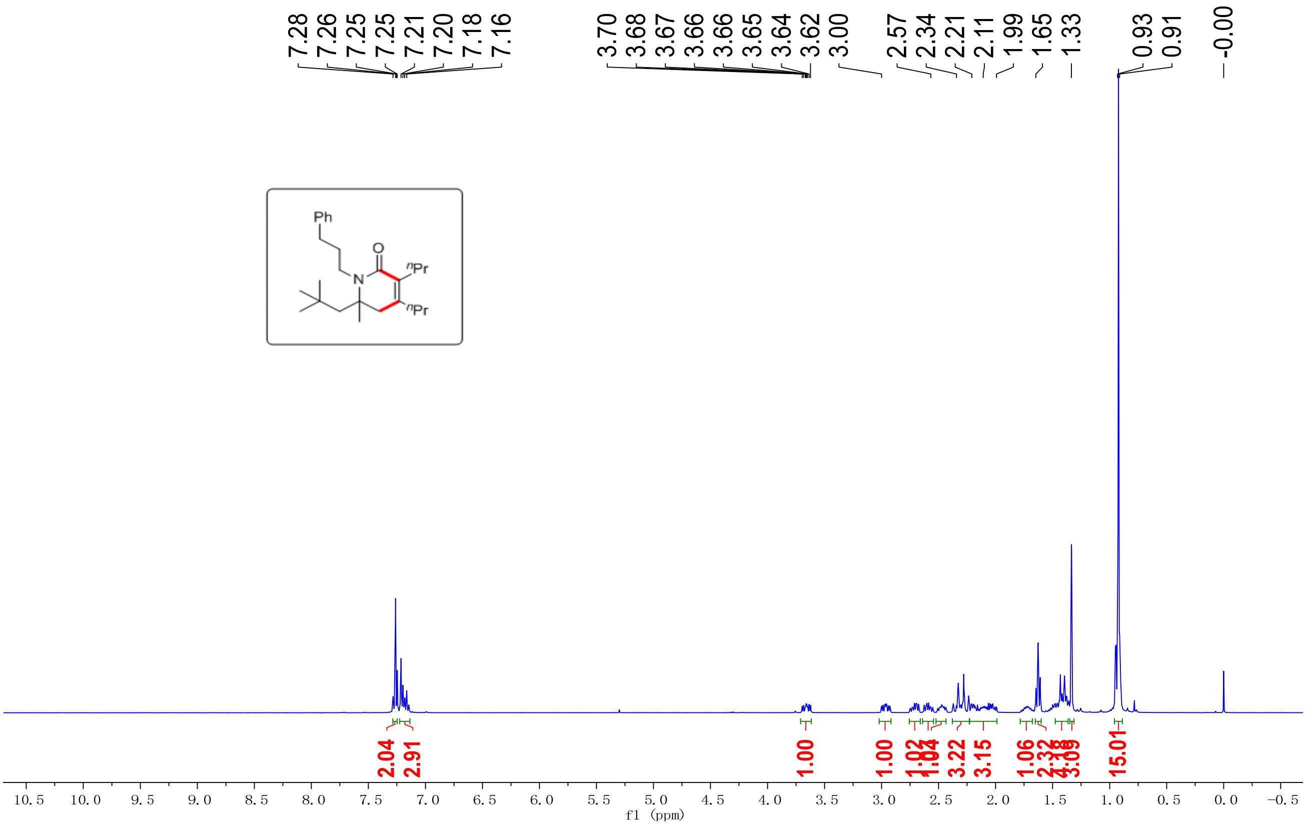

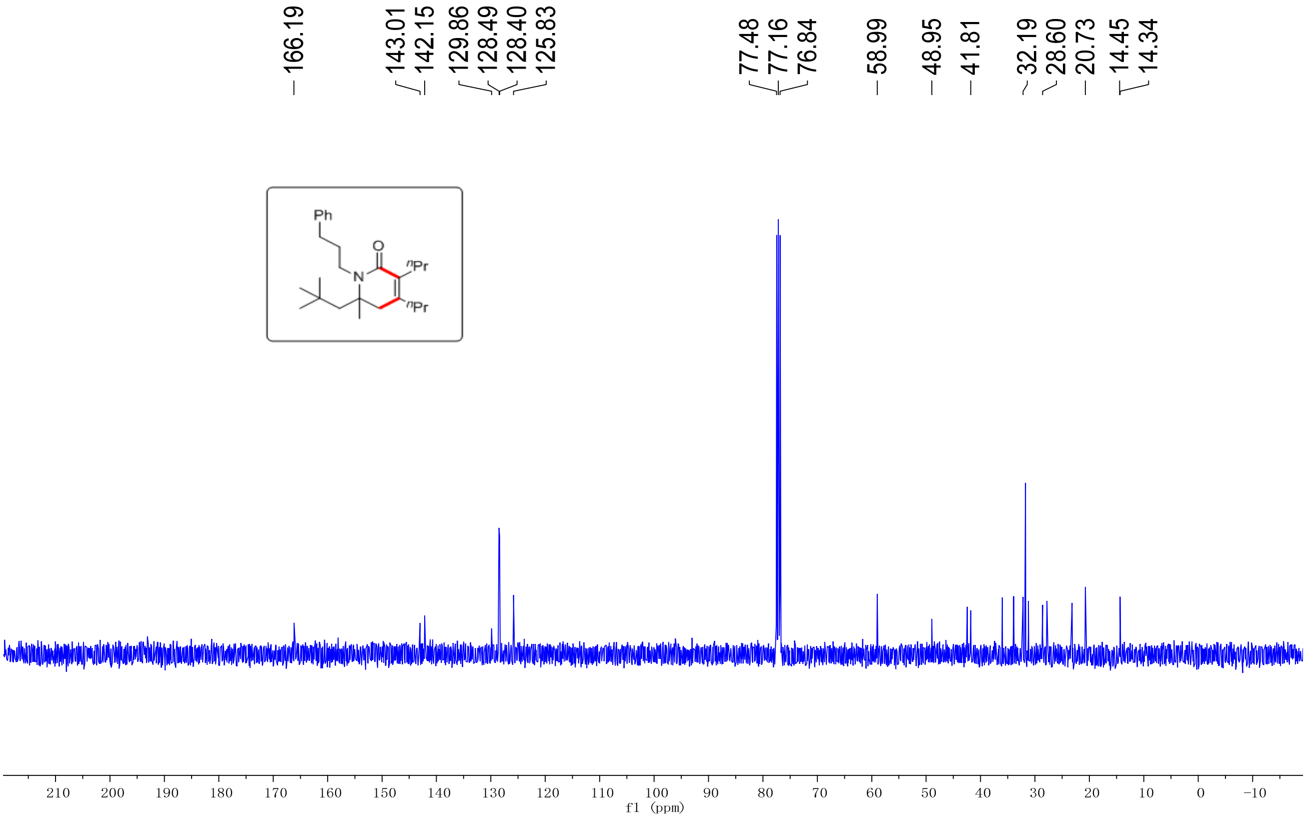

Supplementary Figure 76. ^{1}H and ^{13}C NMR spectra of compound **3y′** in $CDCl_3$

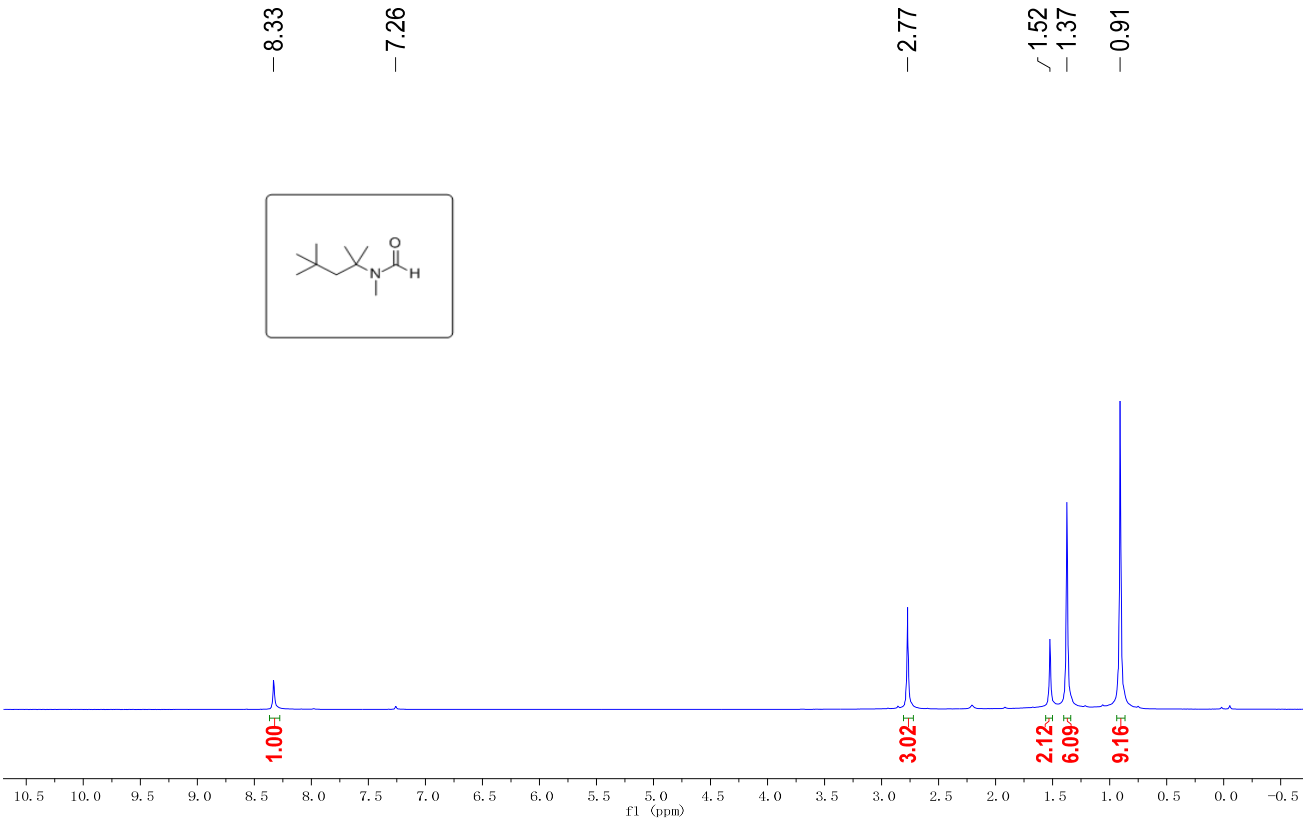

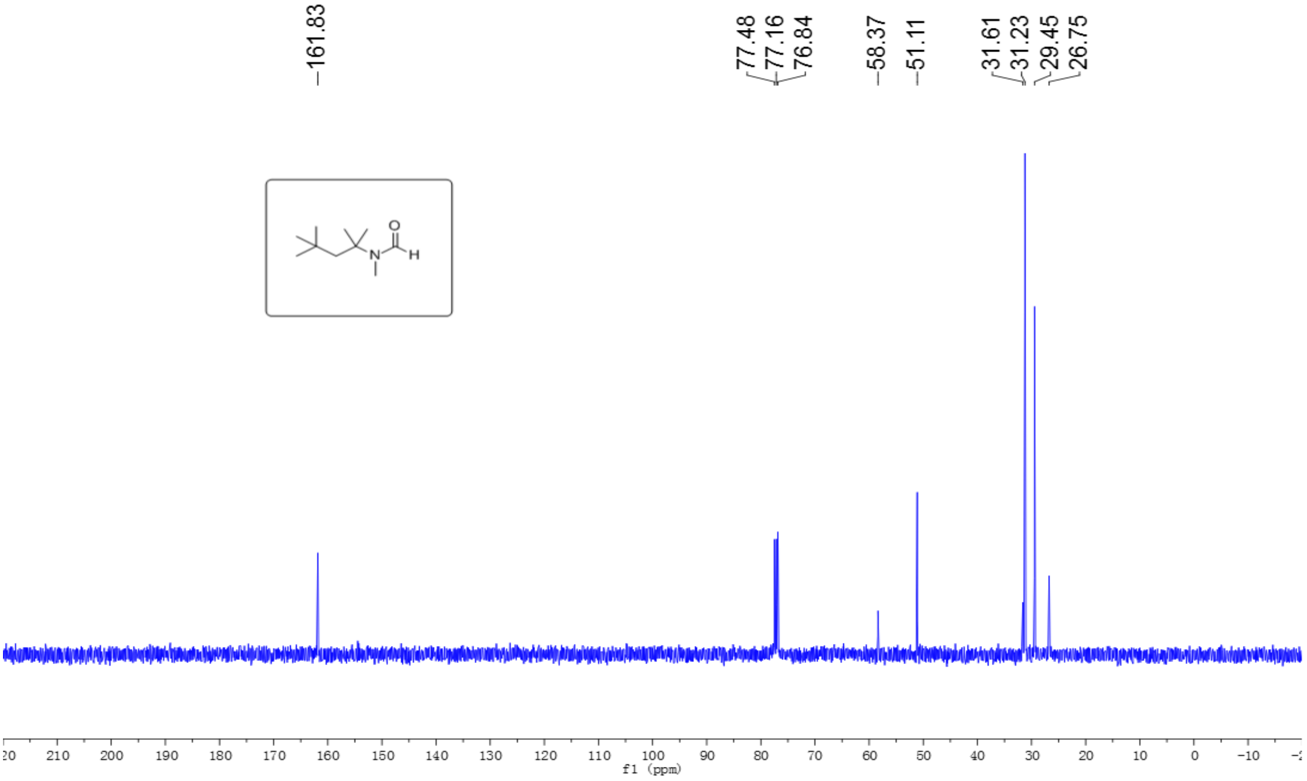

Supplementary Figure 77. ^{1}H and ^{13}C NMR spectra of compound **1z** in $CDCl_3$

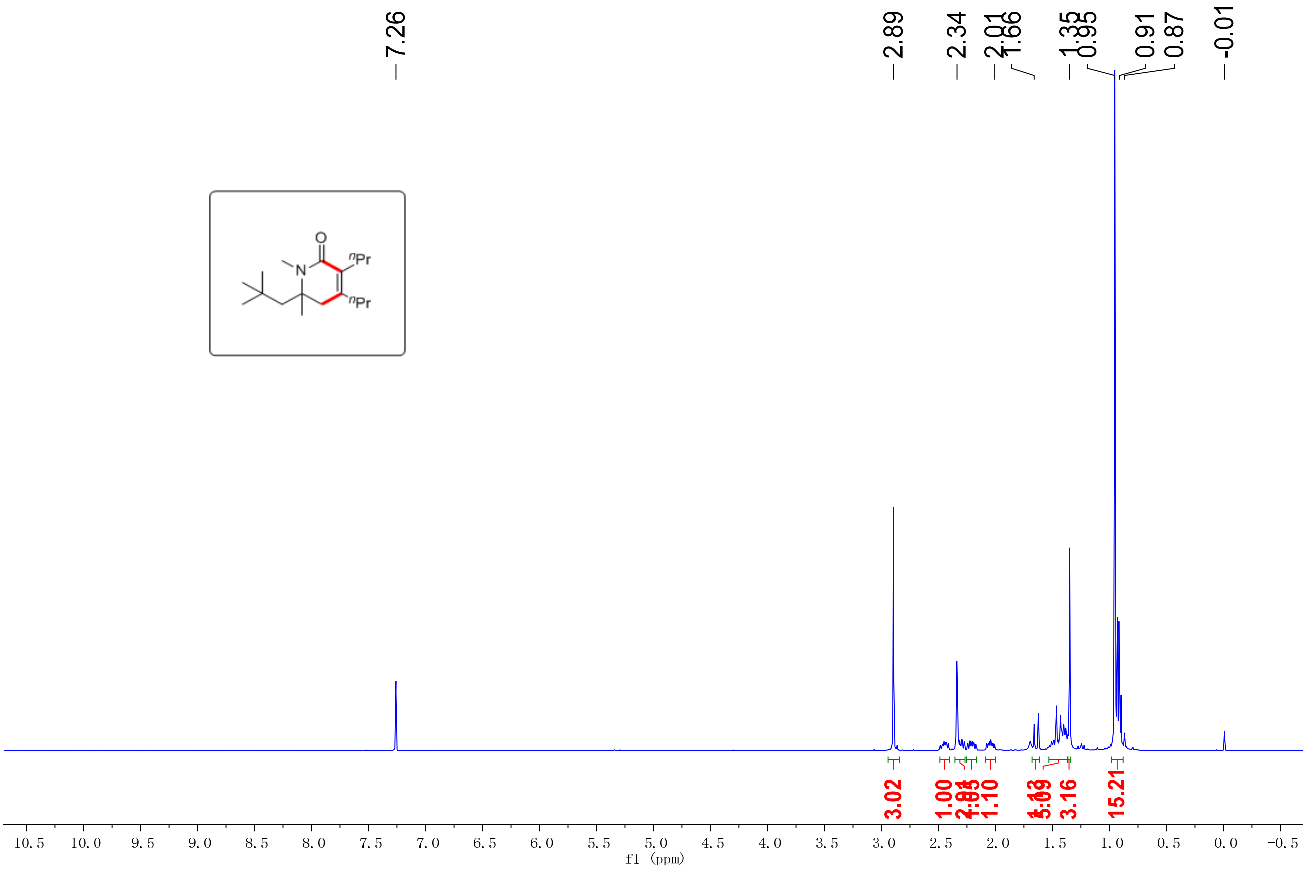

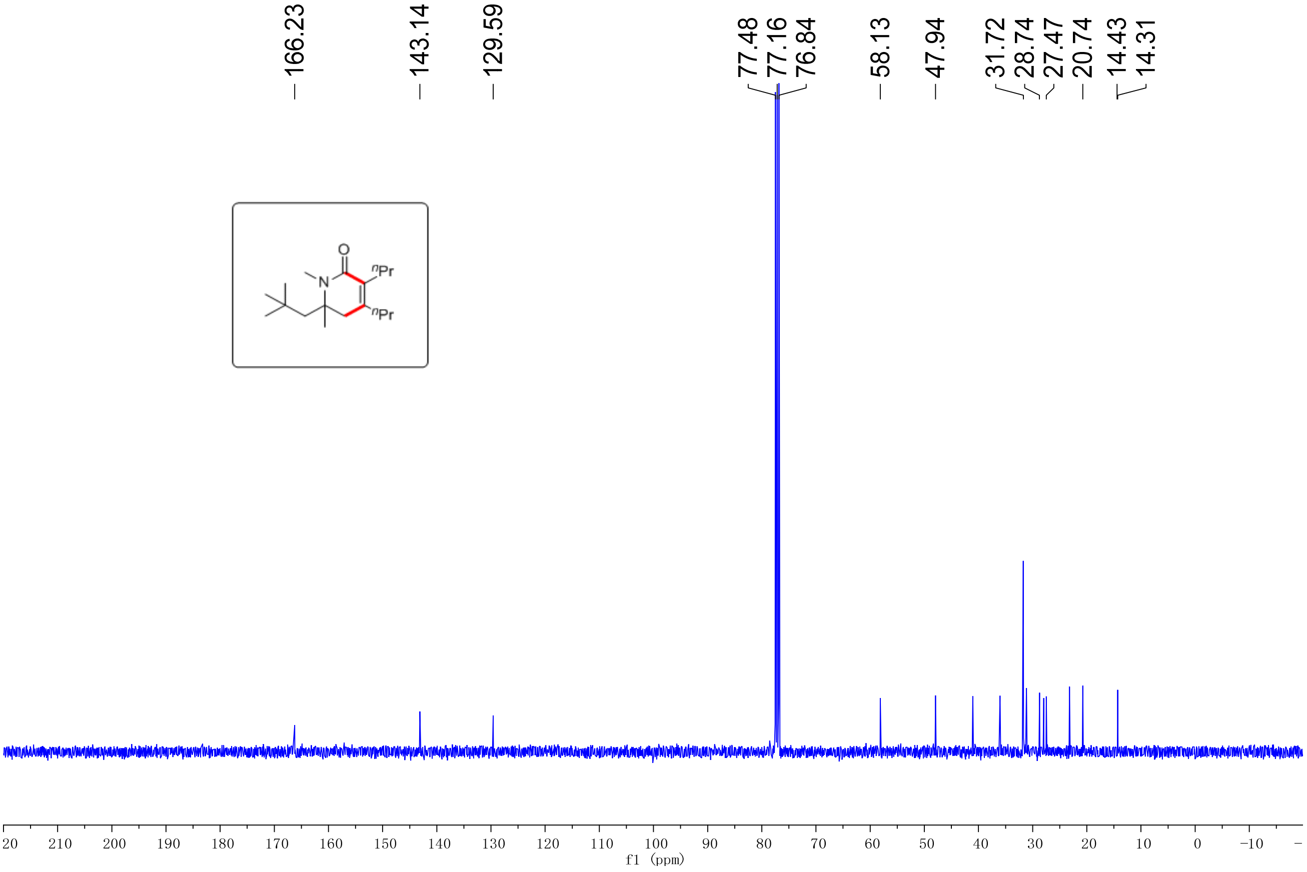

Supplementary Figure 78. ^{1}H and ^{13}C NMR spectra of compound **3z′** in $CDCl_3$

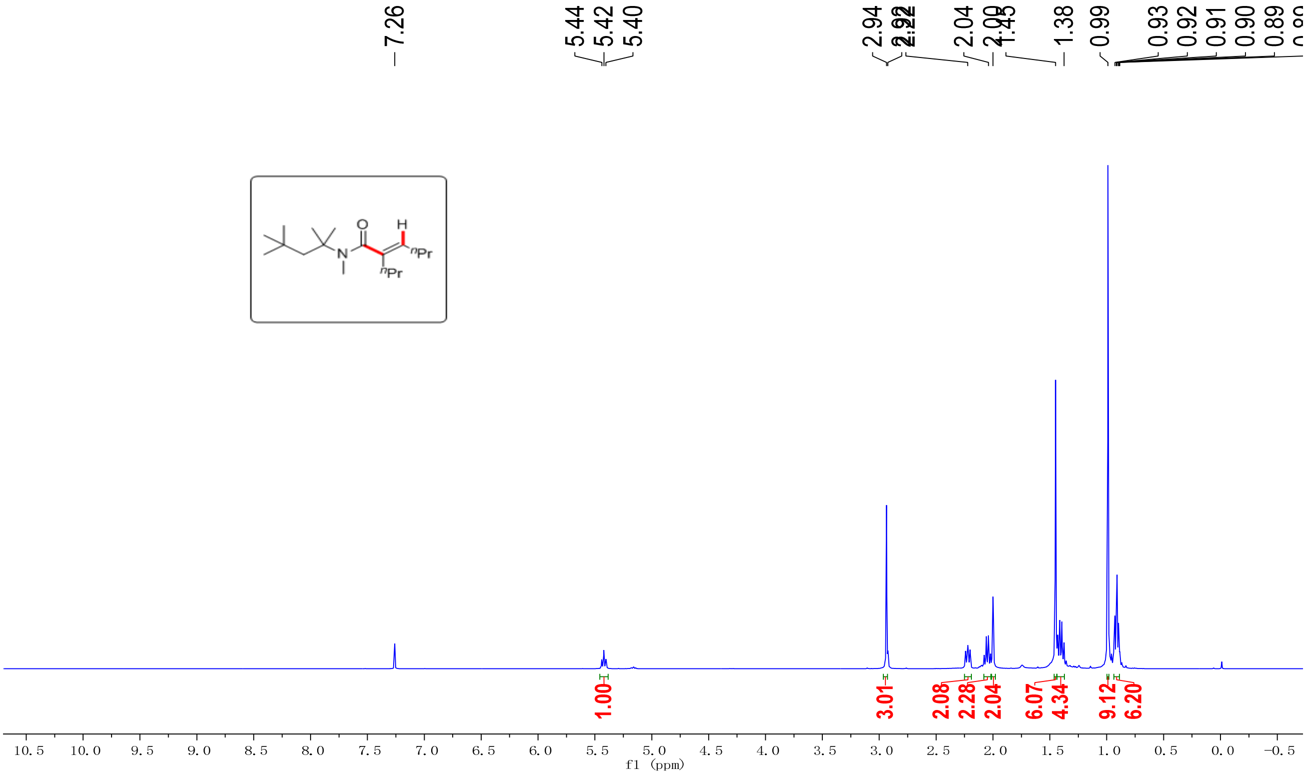

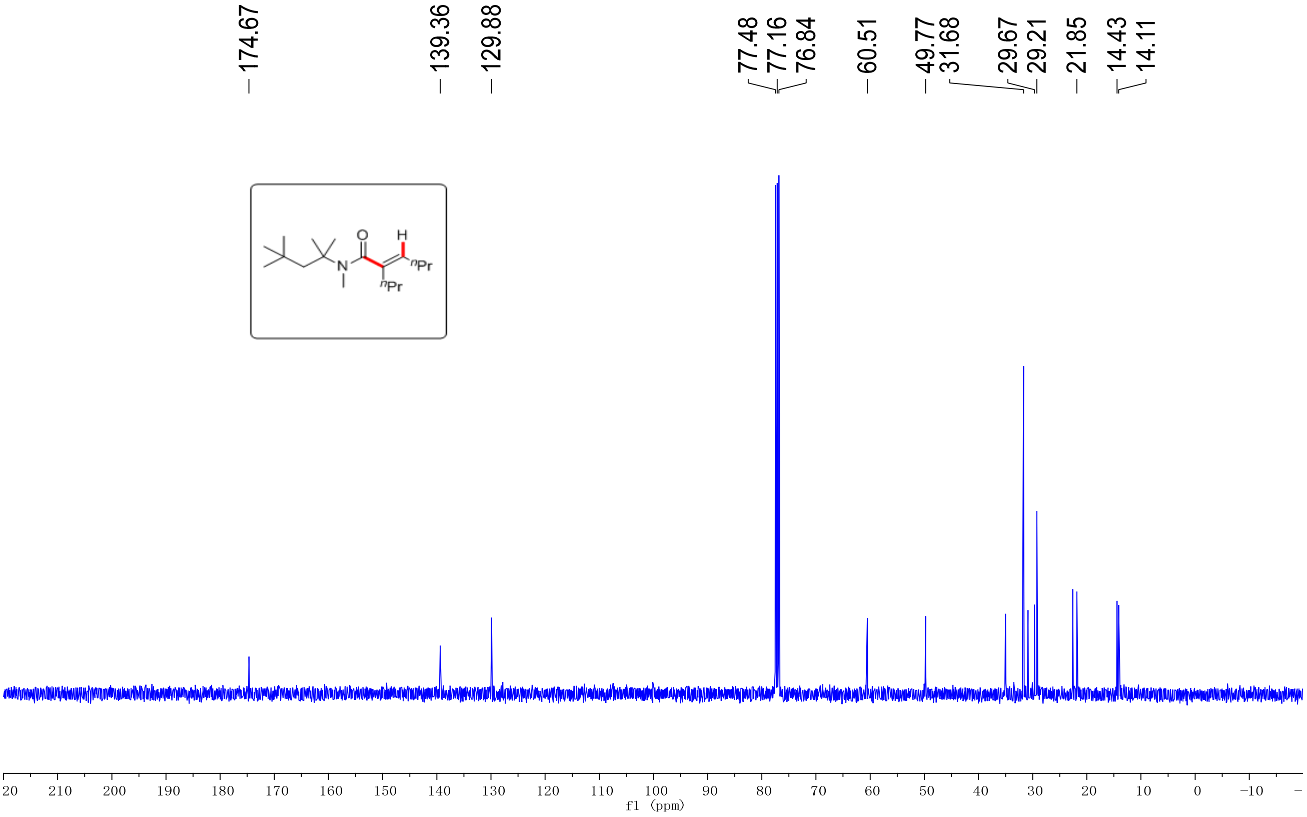

Supplementary Figure 79. ^{1}H and ^{13}C NMR spectrum of compound **3z″** in $CDCl_3$

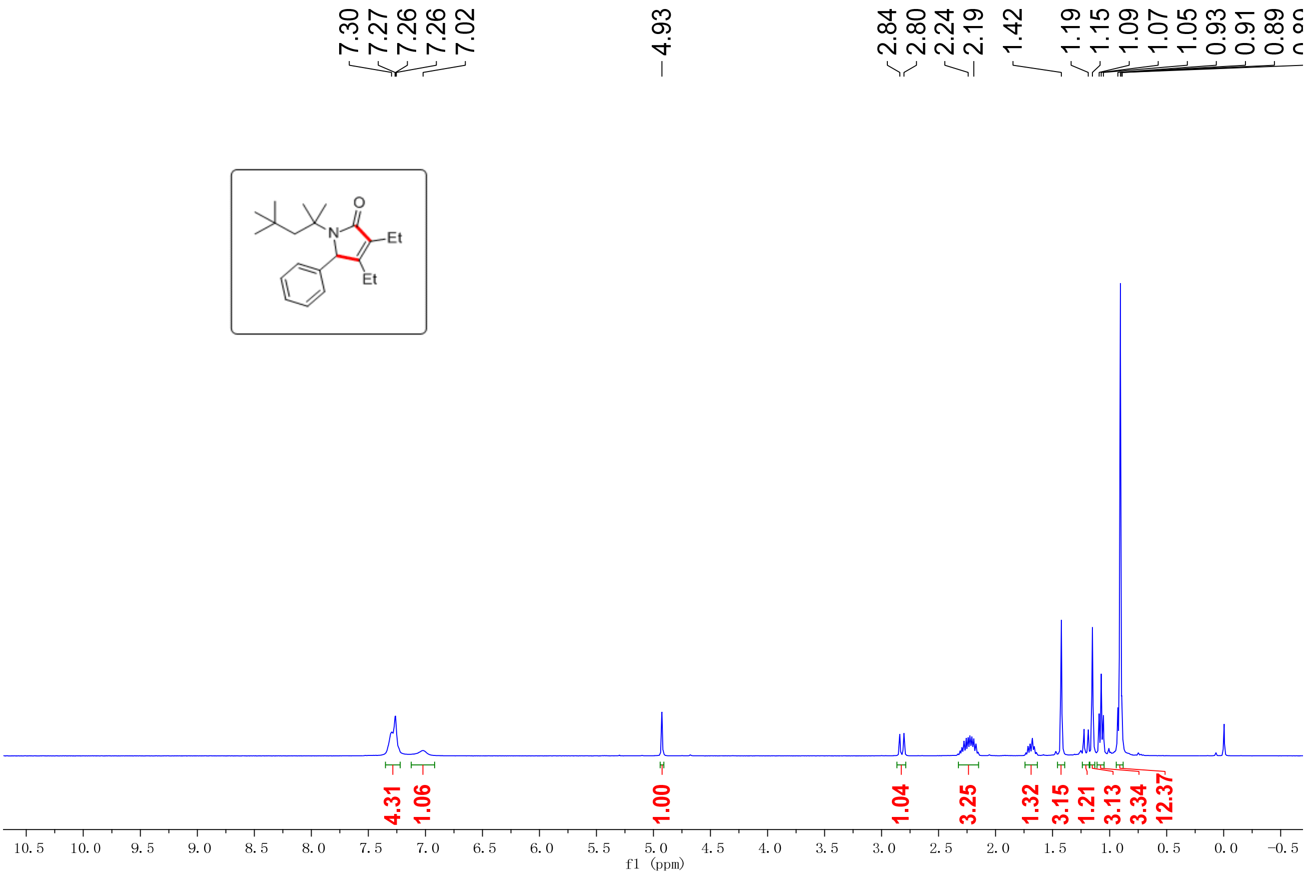

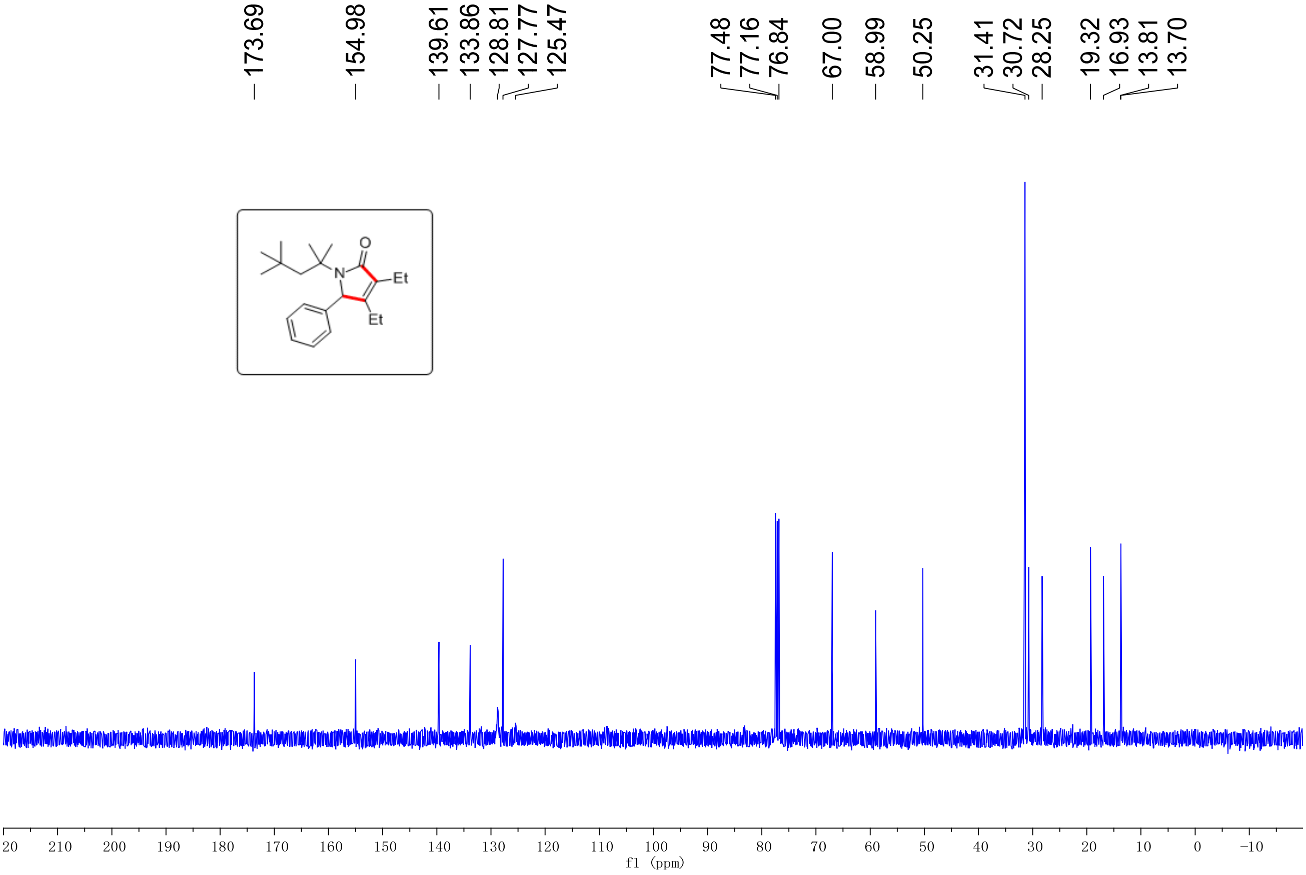

Supplementary Figure 80. ^{1}H and ^{13}C NMR spectra of compound **4a** in $CDCl_3$

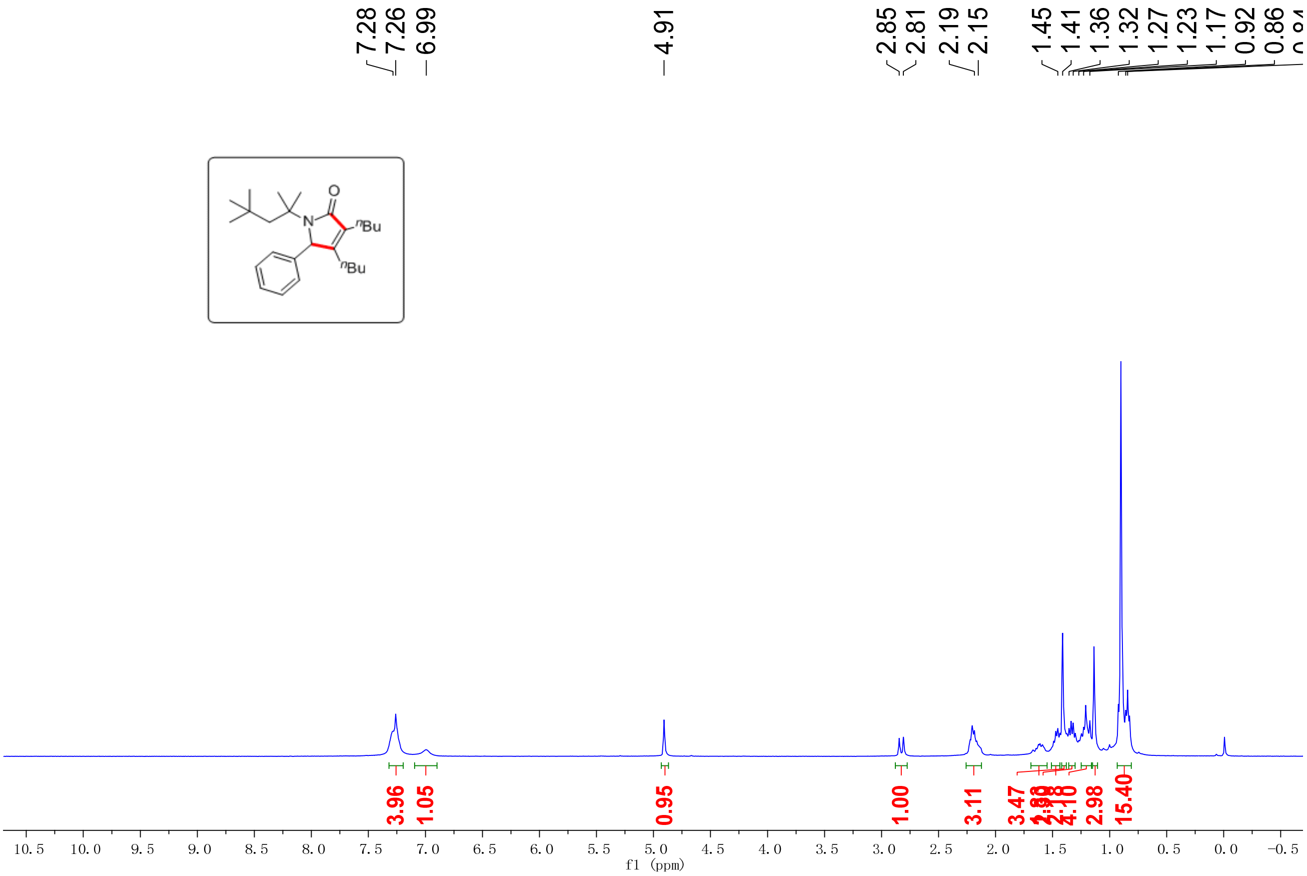

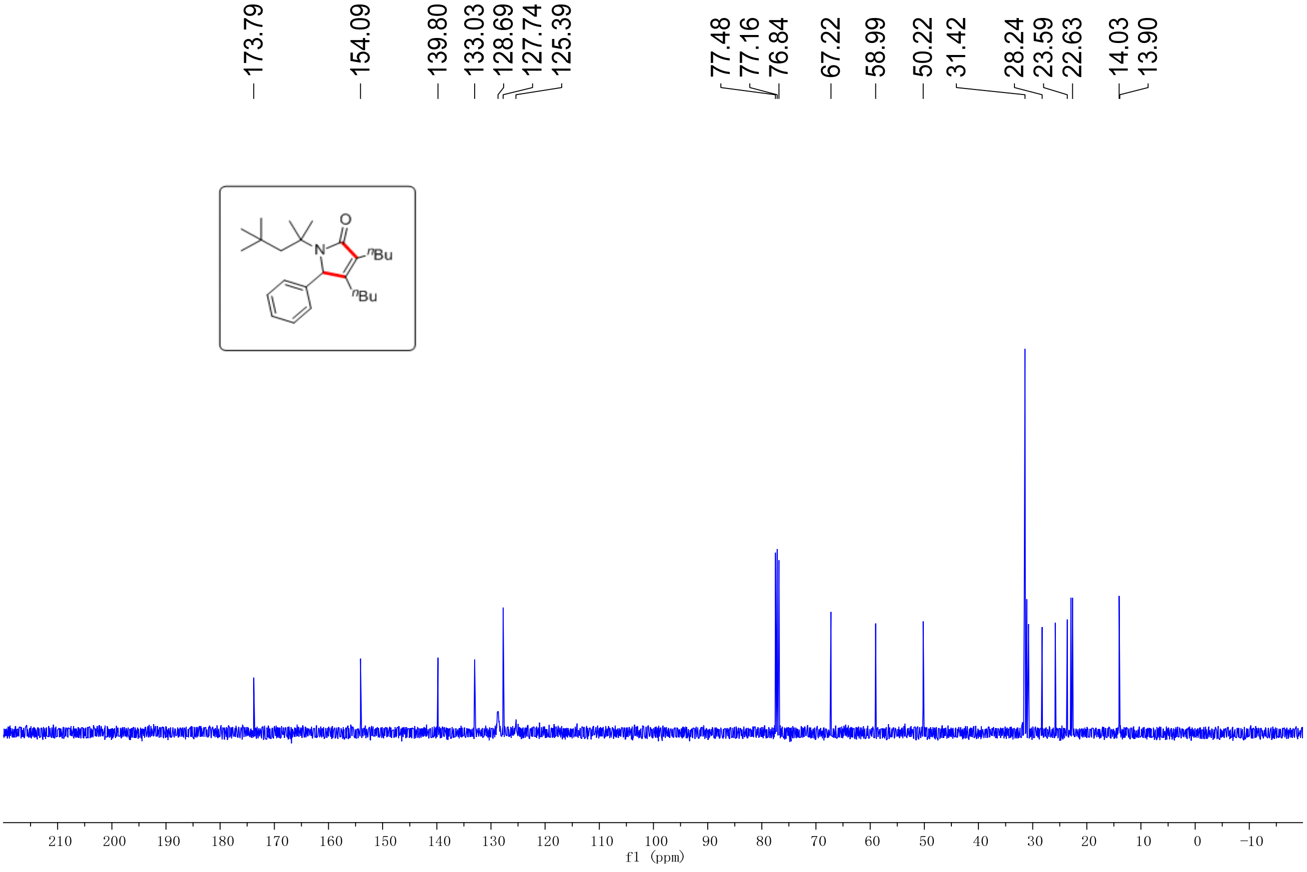

Supplementary Figure 81. ^{1}H and ^{13}C NMR spectra of compound **4b** in $CDCl_3$

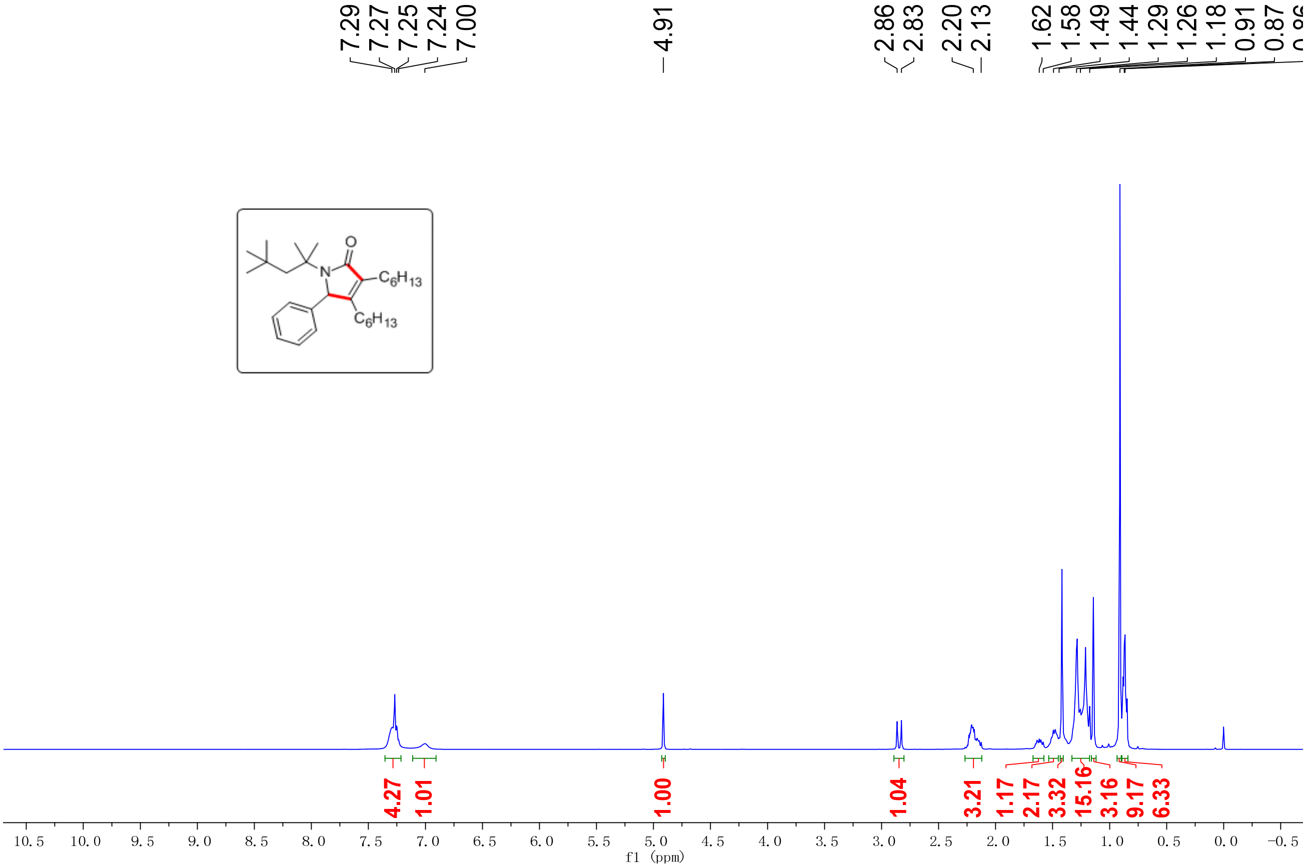

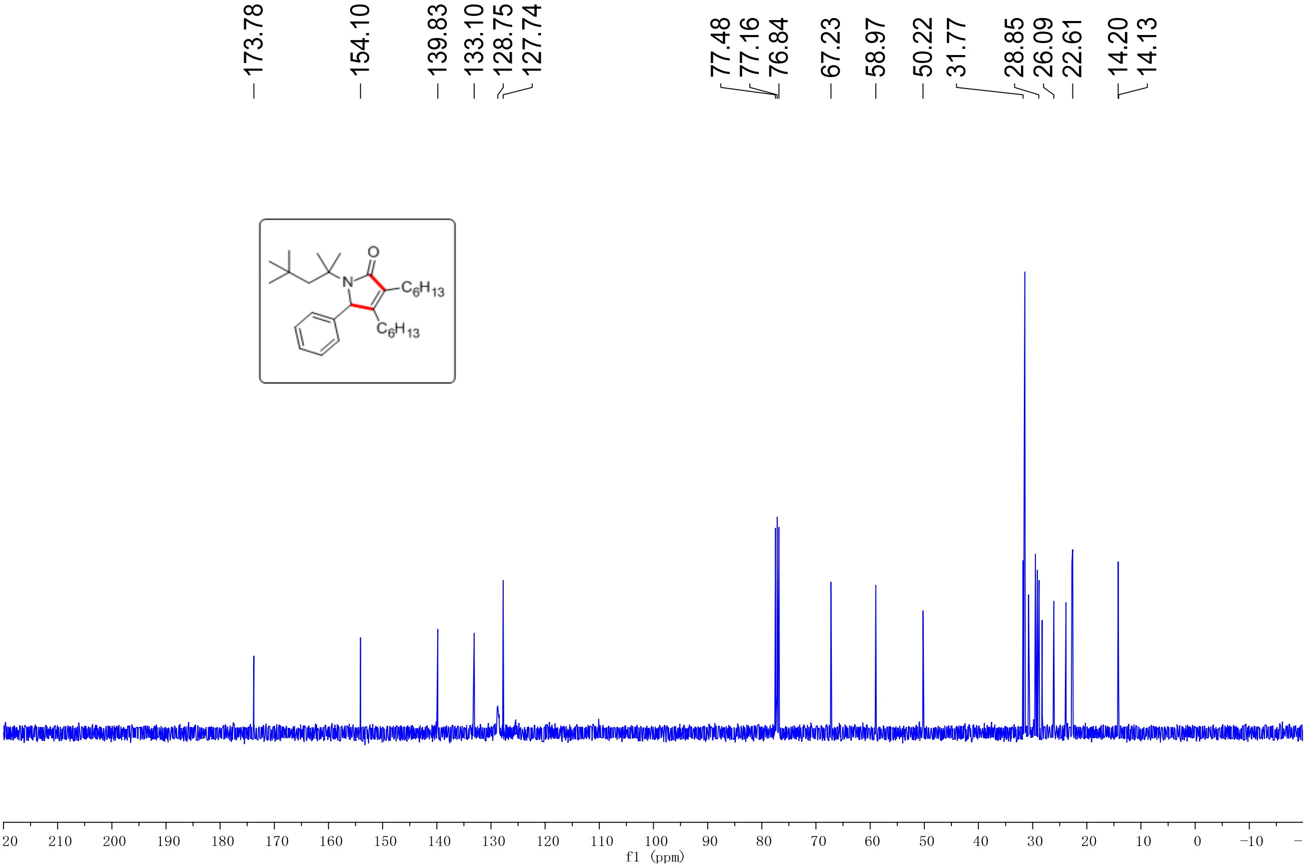

Supplementary Figure 82. ^{1}H and ^{13}C NMR spectra of compound **4c** in $CDCl_3$

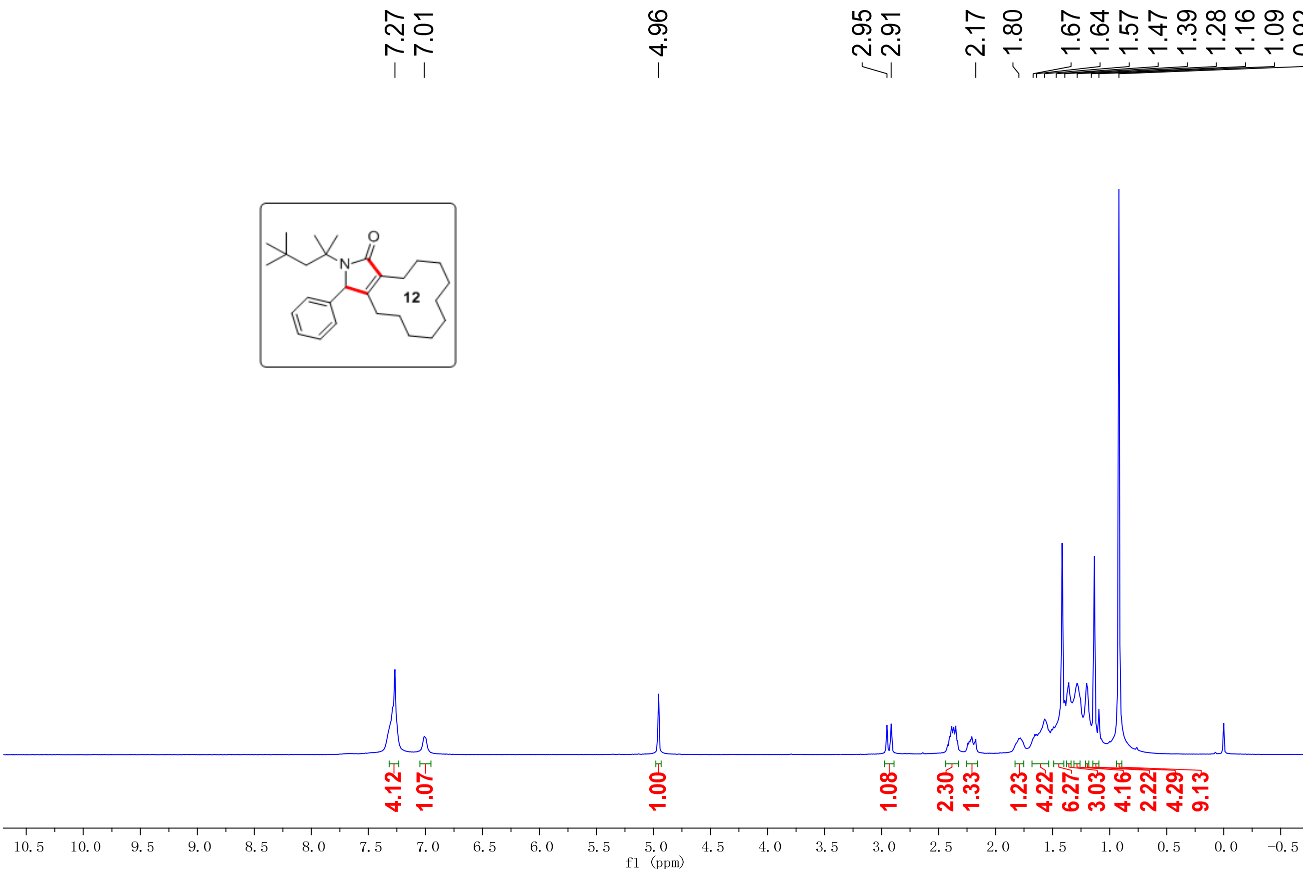

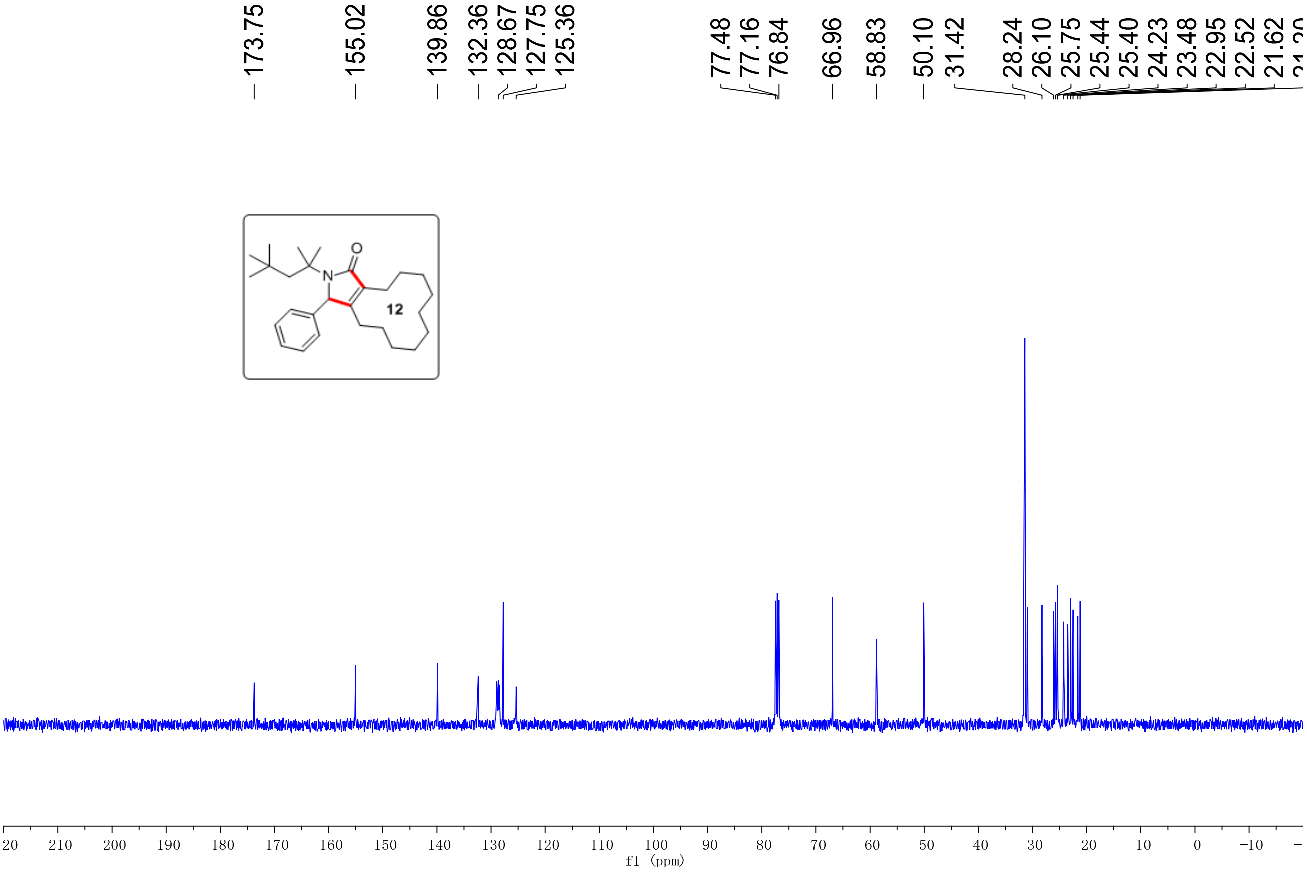

Supplementary Figure 83. ^{1}H and ^{13}C NMR spectra of compound **4d** in $CDCl_3$

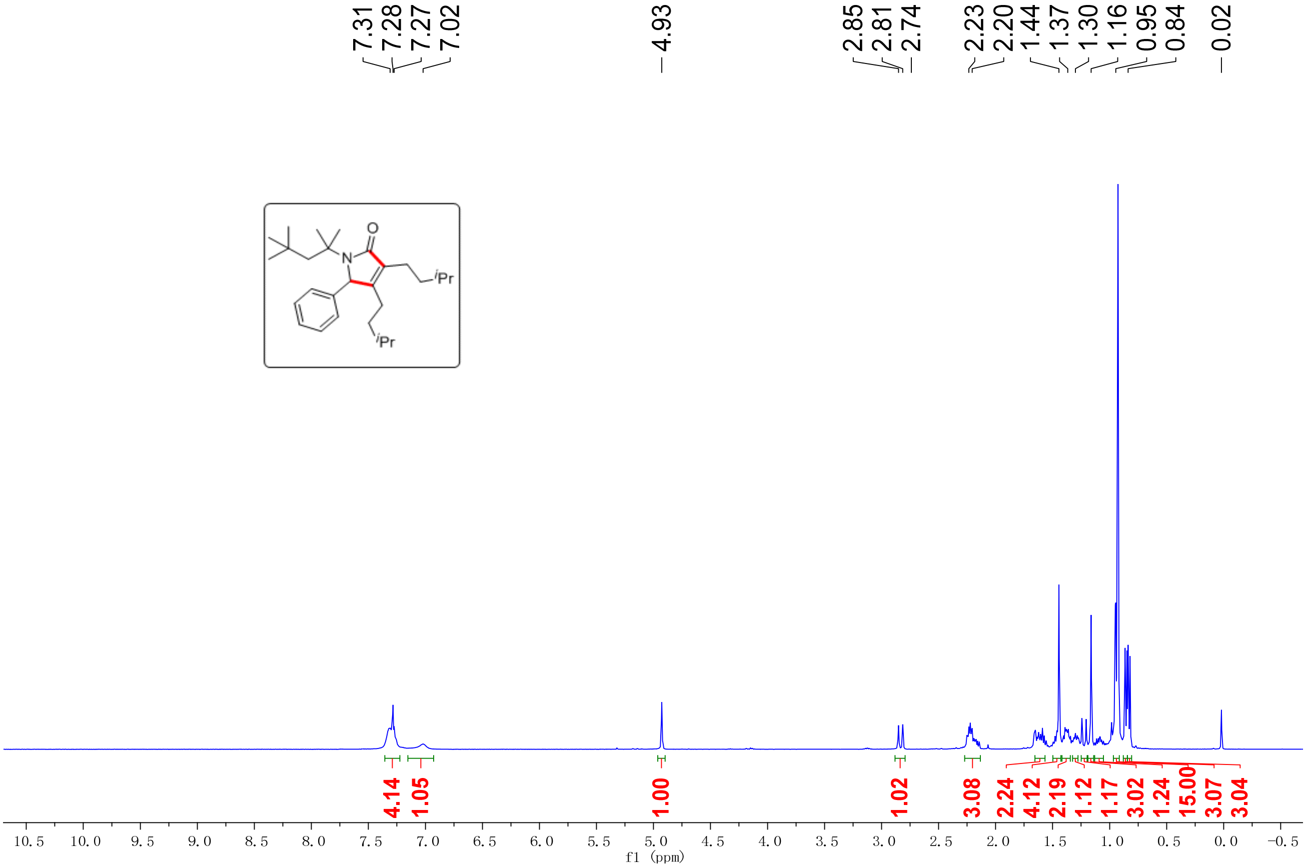

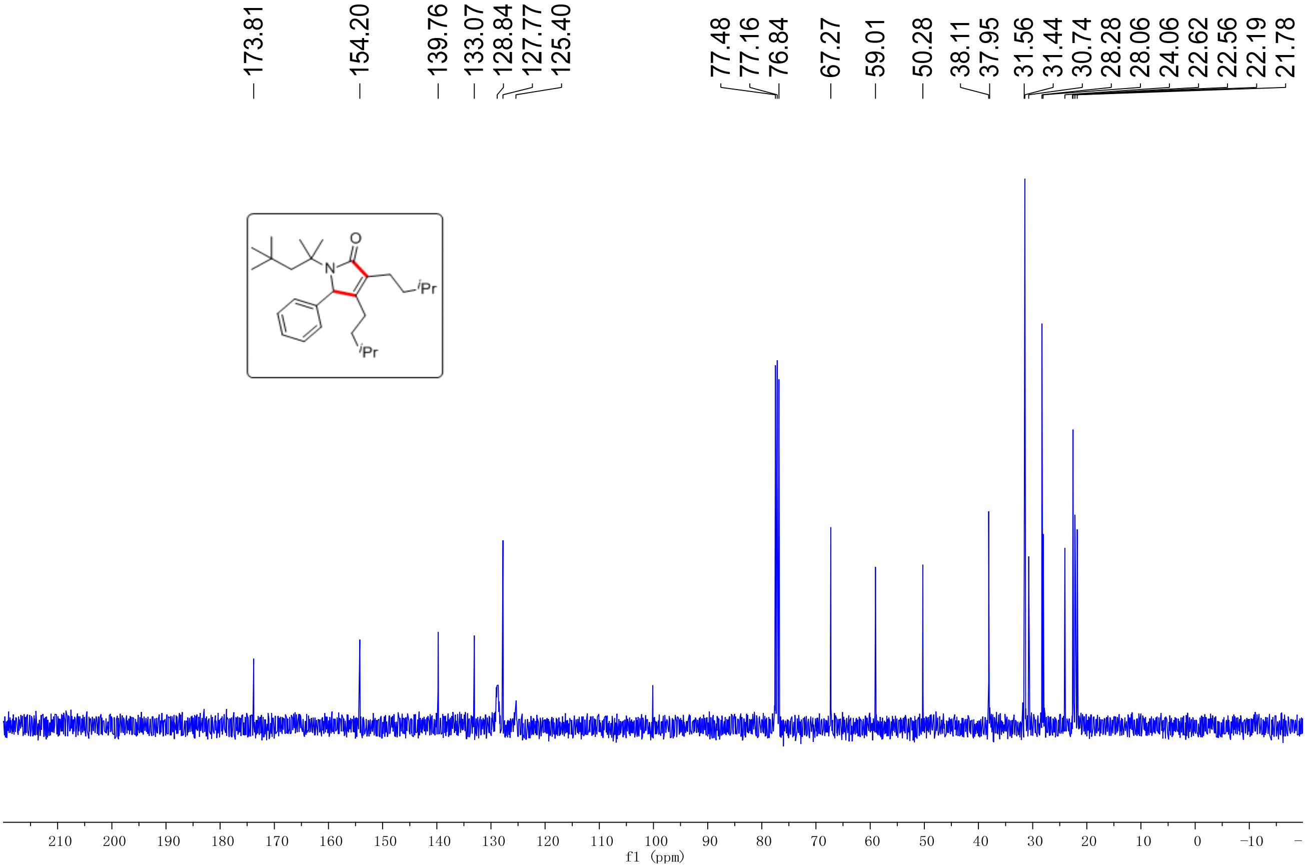

Supplementary Figure 84. ^{1}H and ^{13}C NMR spectra of compound **4e** in $CDCl_3$

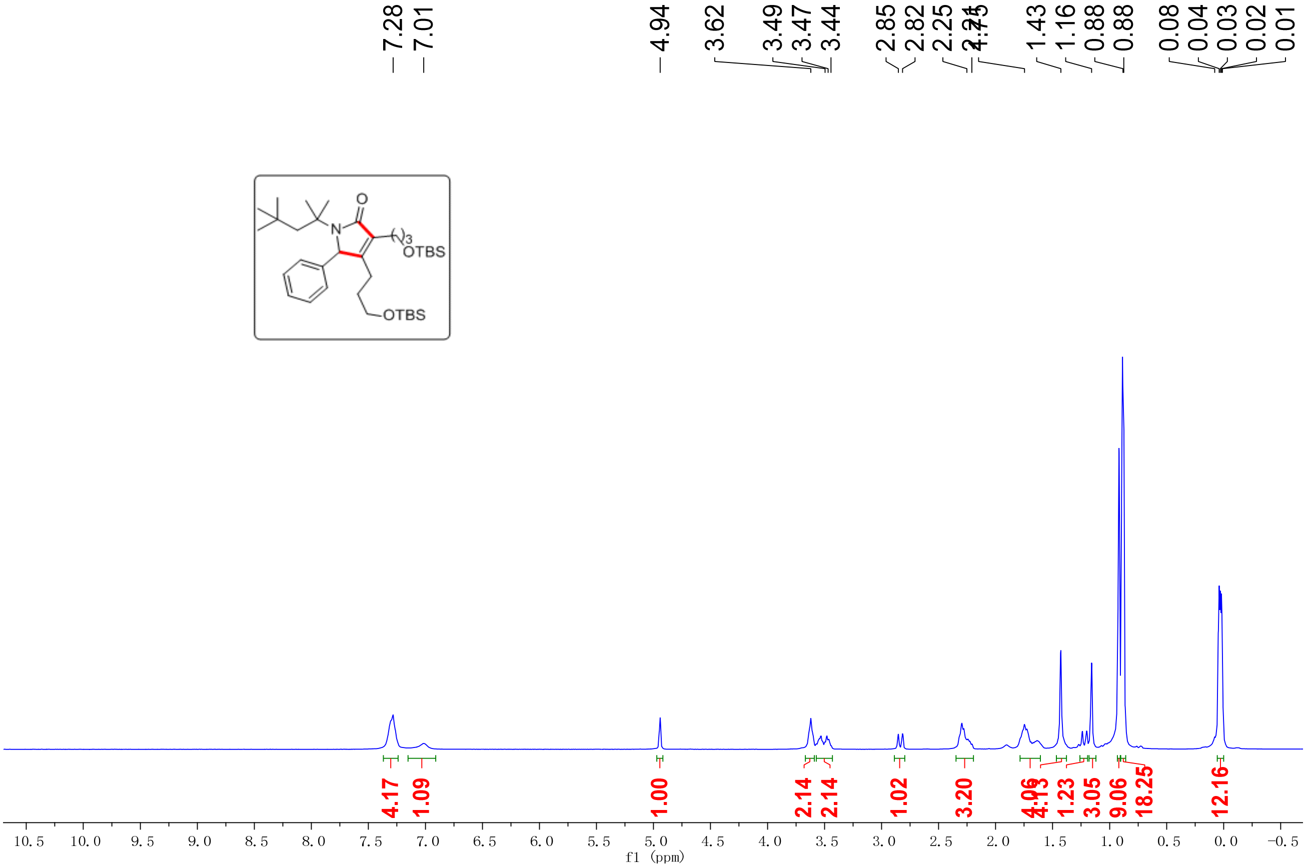

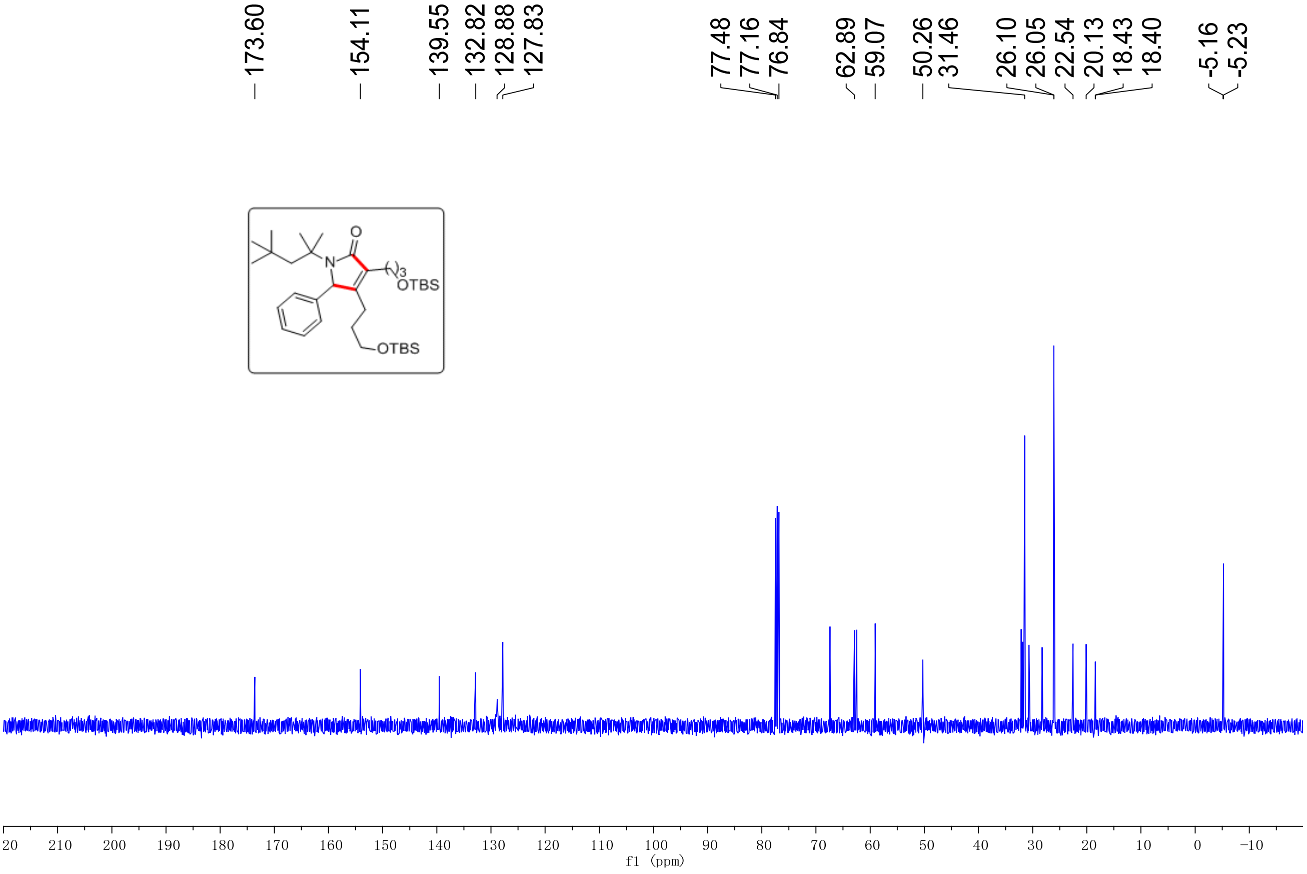

Supplementary Figure 85. ^{1}H and ^{13}C NMR spectra of compound **4f** in $CDCl_3$

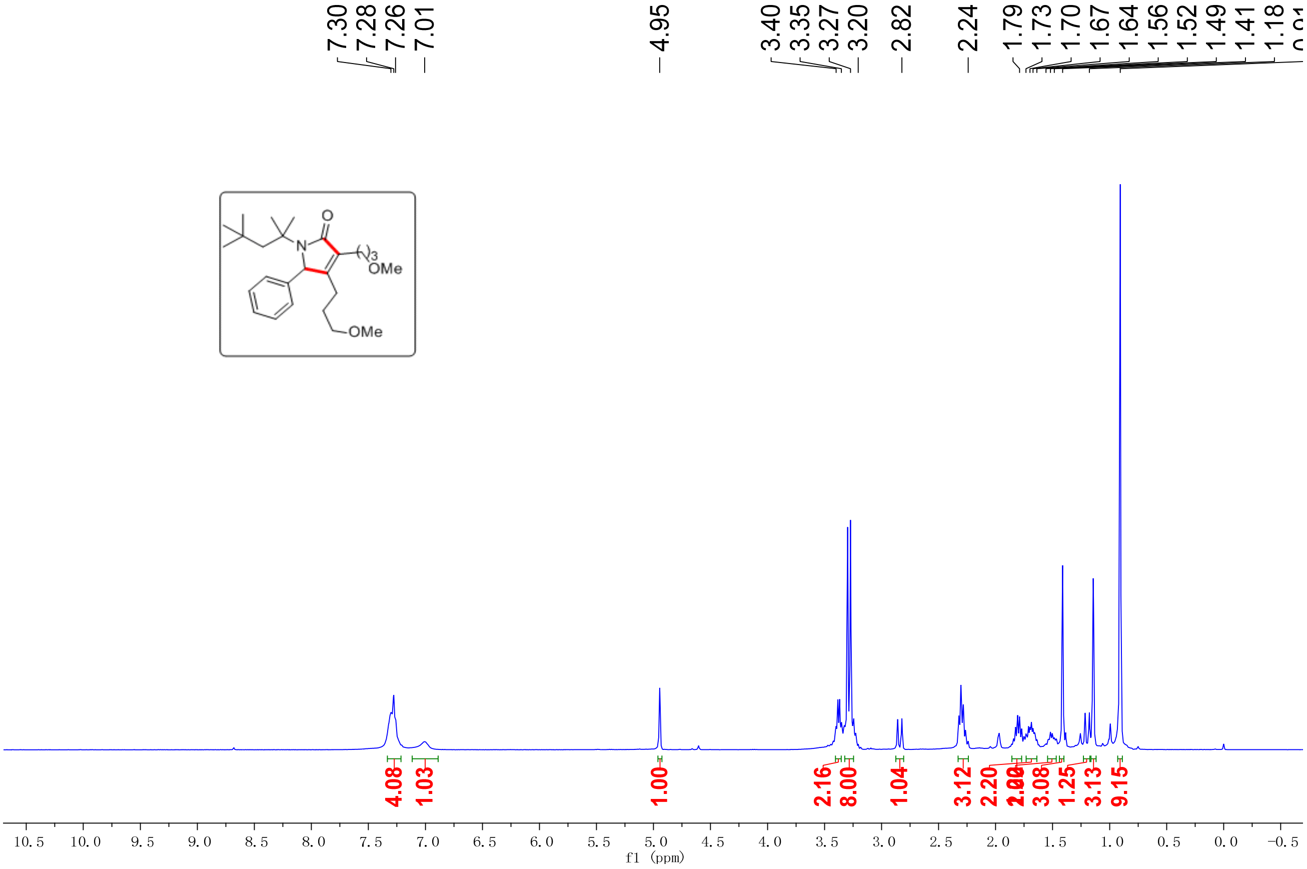

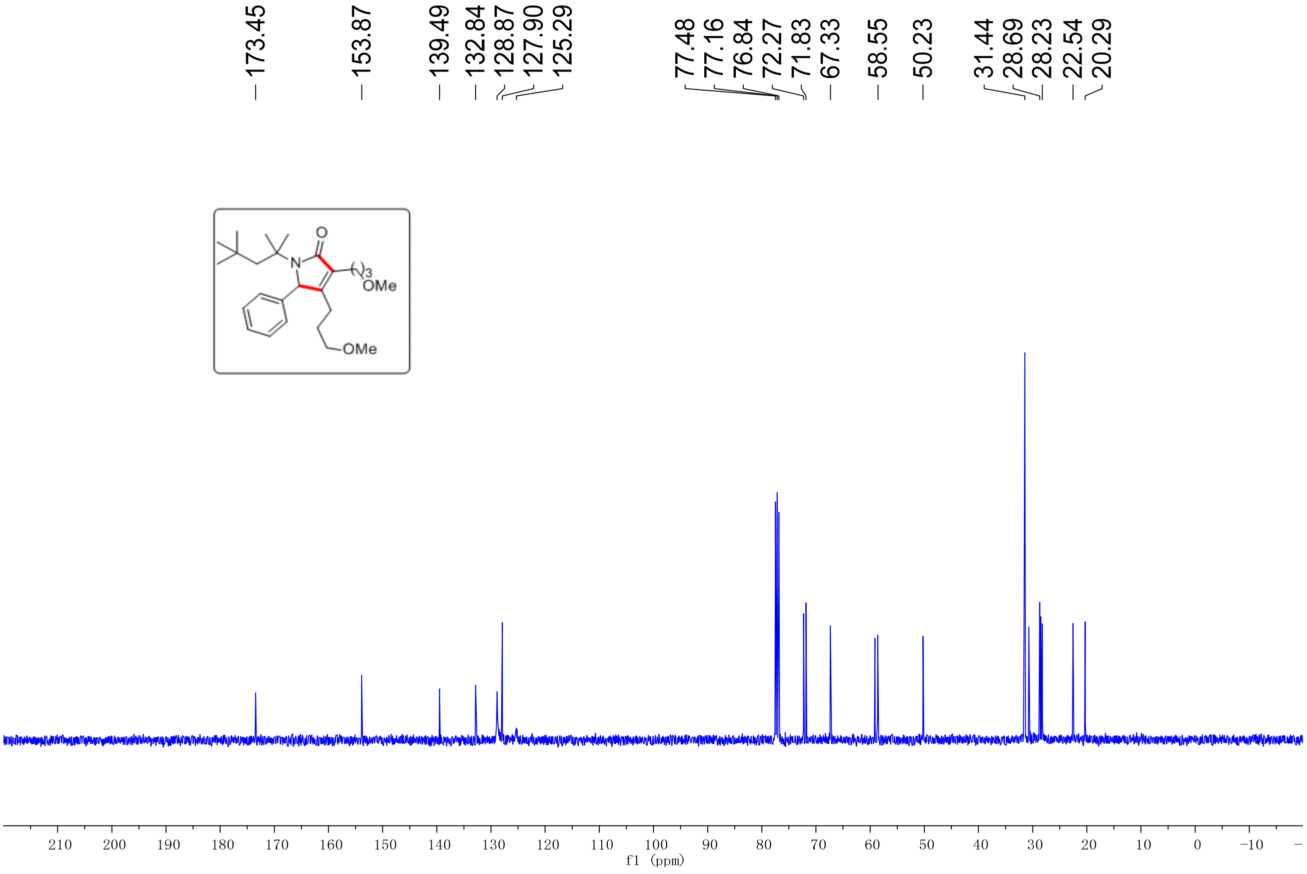

Supplementary Figure 86. ^{1}H and ^{13}C NMR spectra of compound **4g** in $CDCl_3$

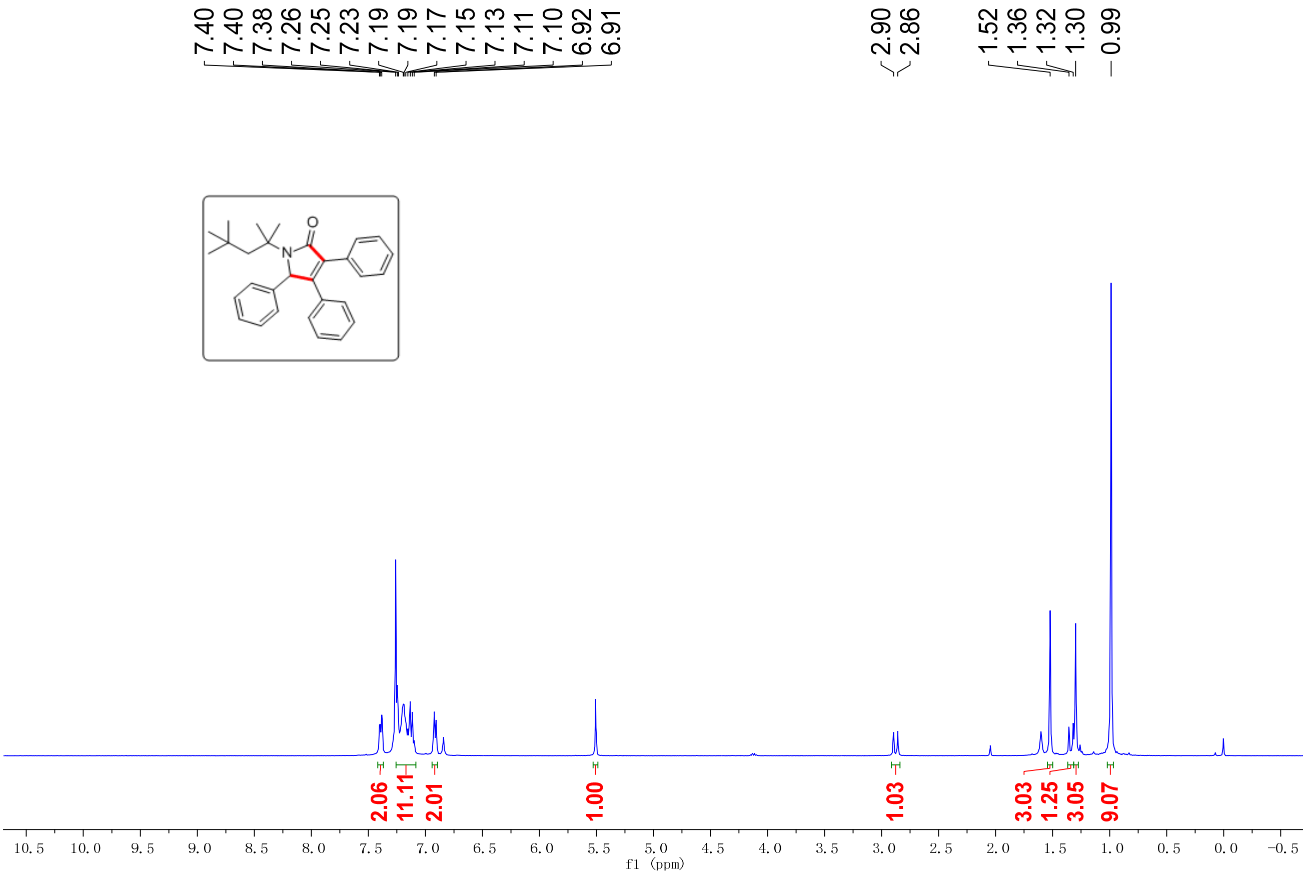

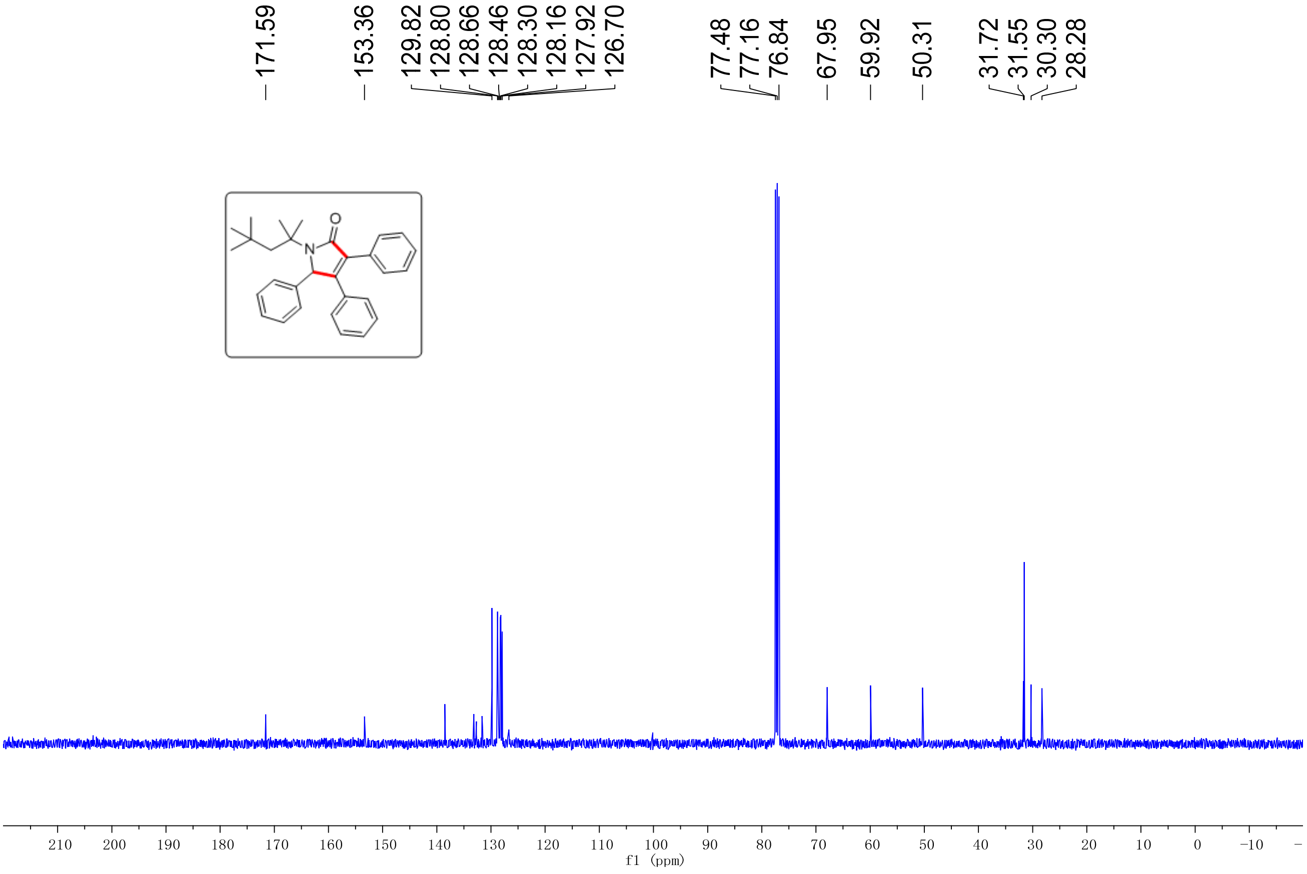

Supplementary Figure 87. ^{1}H and ^{13}C NMR spectra of compound **4h** in $CDCl_3$

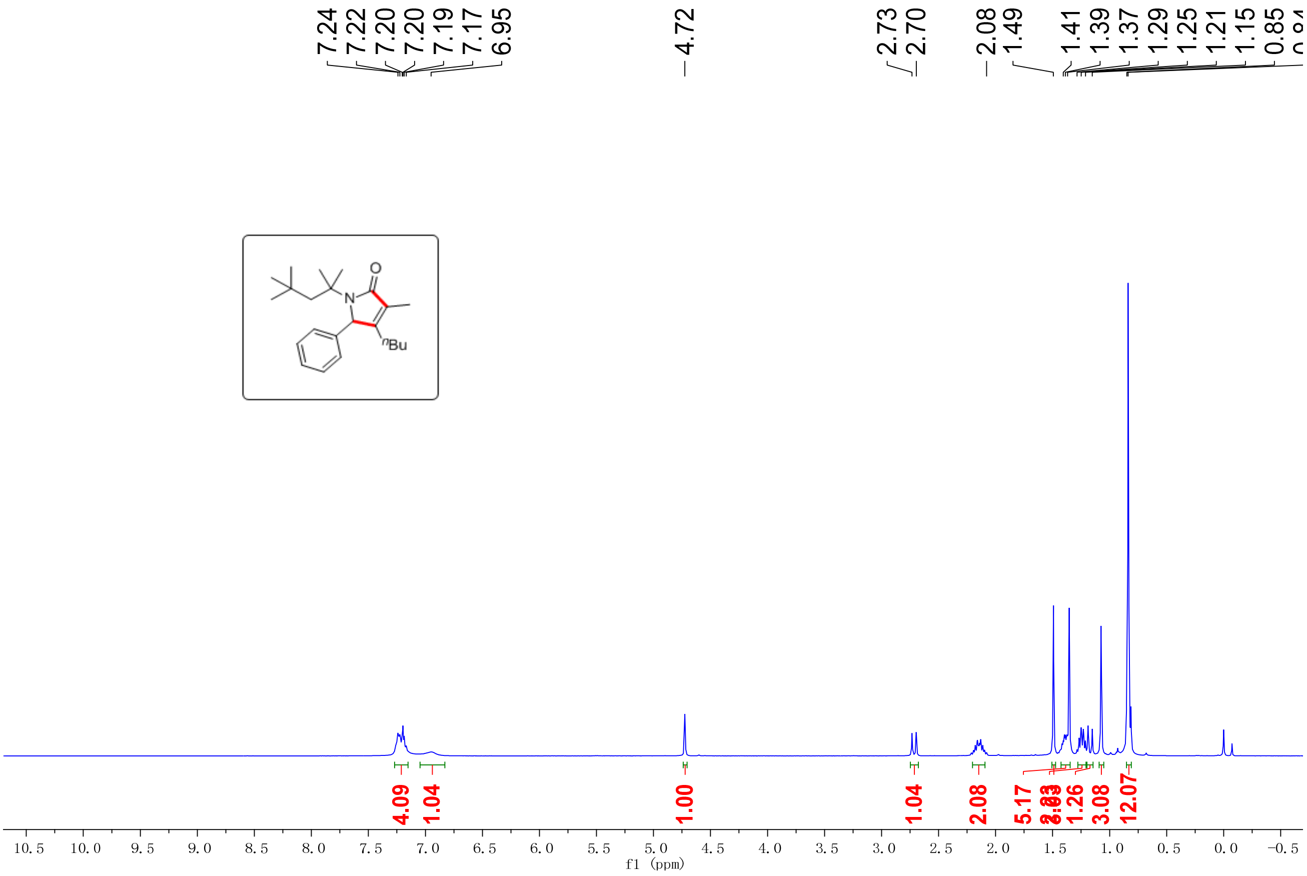

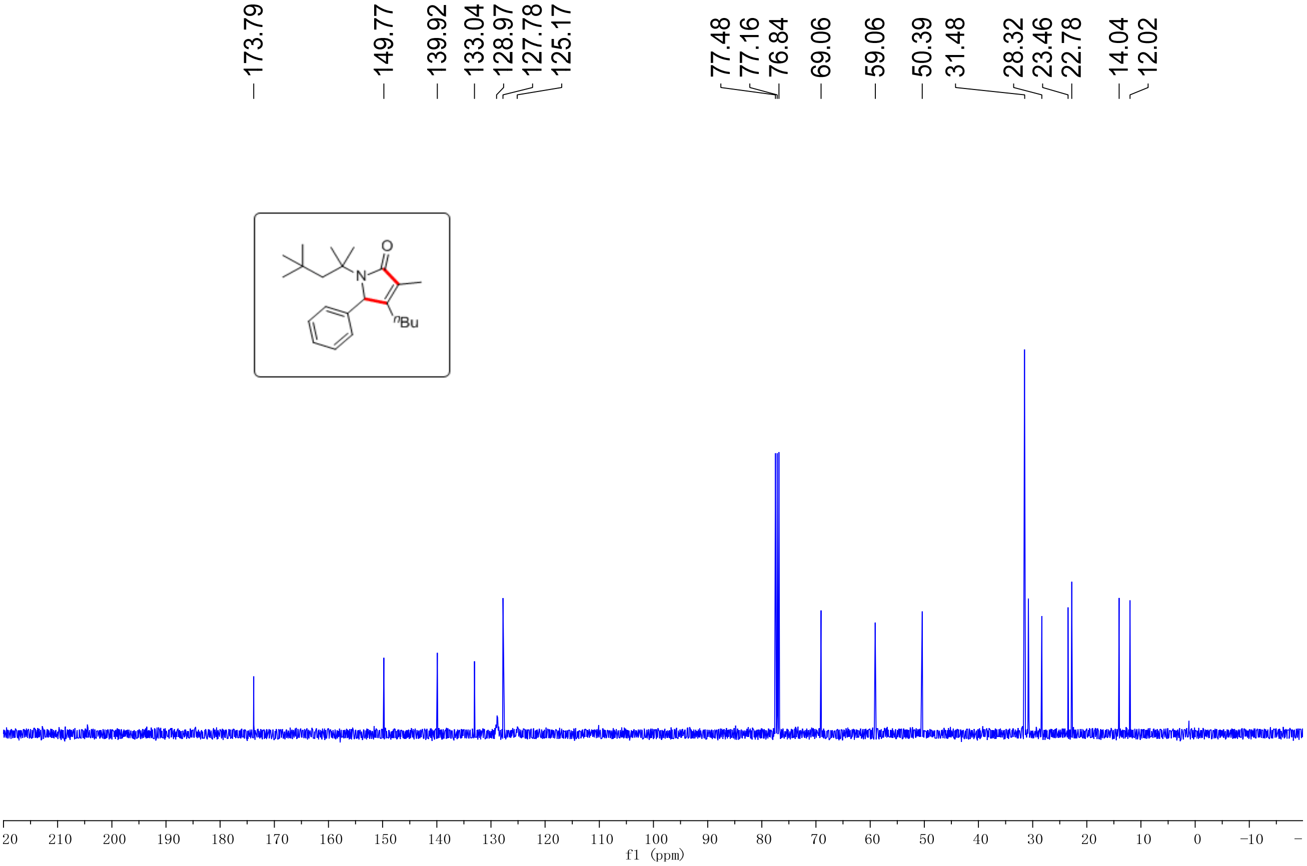

Supplementary Figure 88. ^{1}H and ^{13}C NMR spectra of compound **4i** in $CDCl_3$

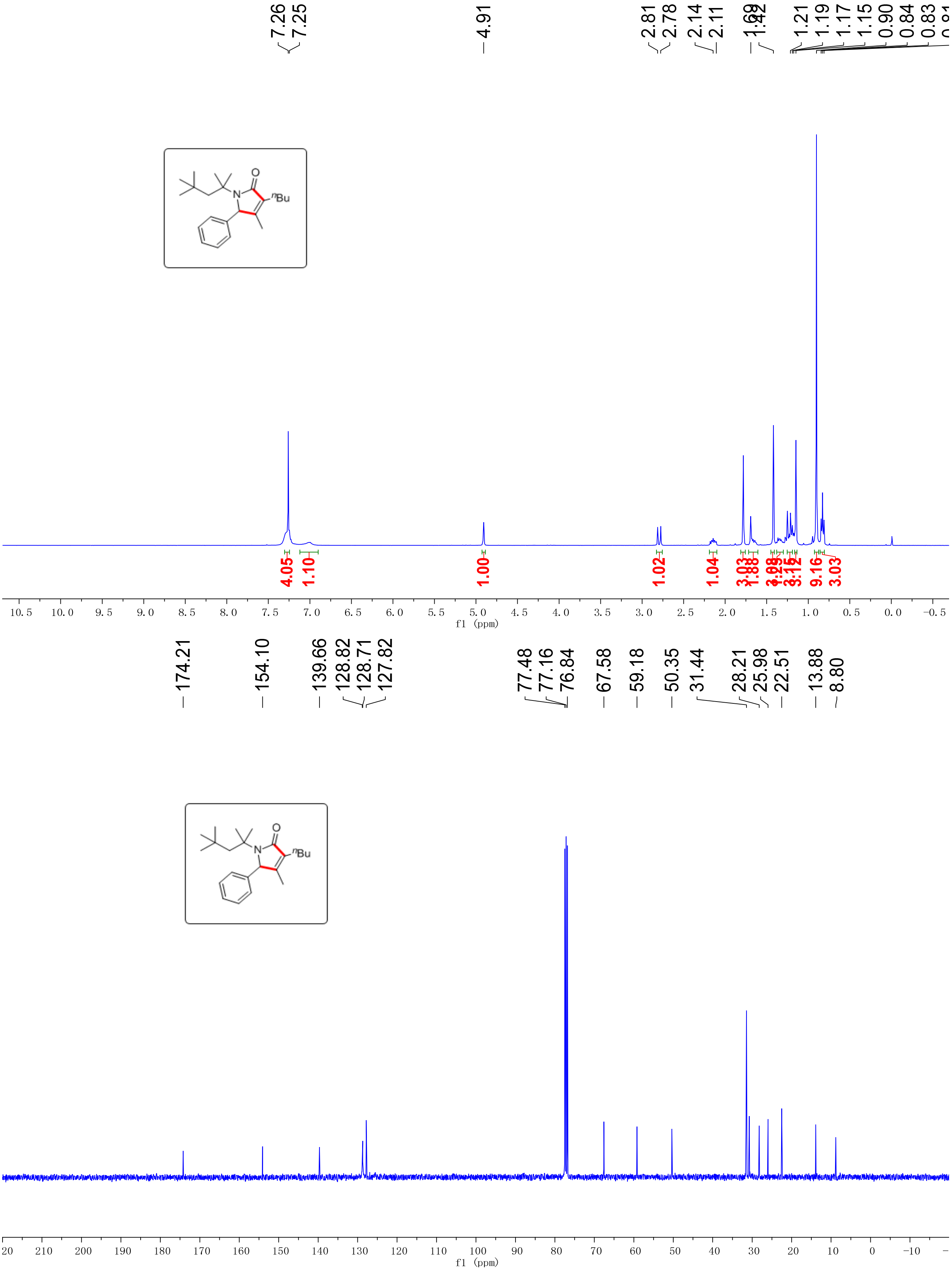

Supplementary Figure 89. ^{1}H and ^{13}C NMR spectra of compound **4i′** in $CDCl_3$

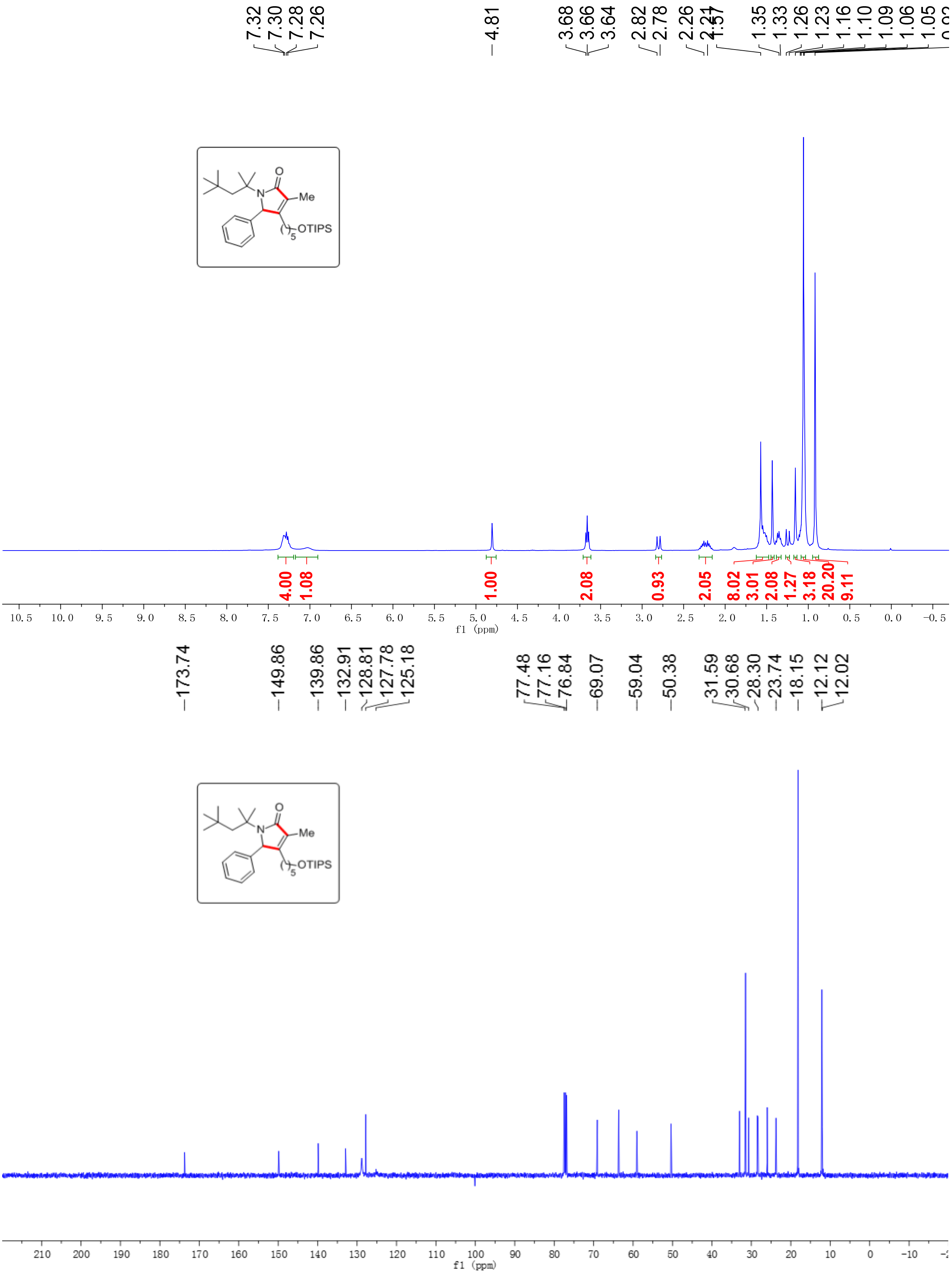

Supplementary Figure 90. ^{1}H and ^{13}C NMR spectra of compound **4j** in $CDCl_3$

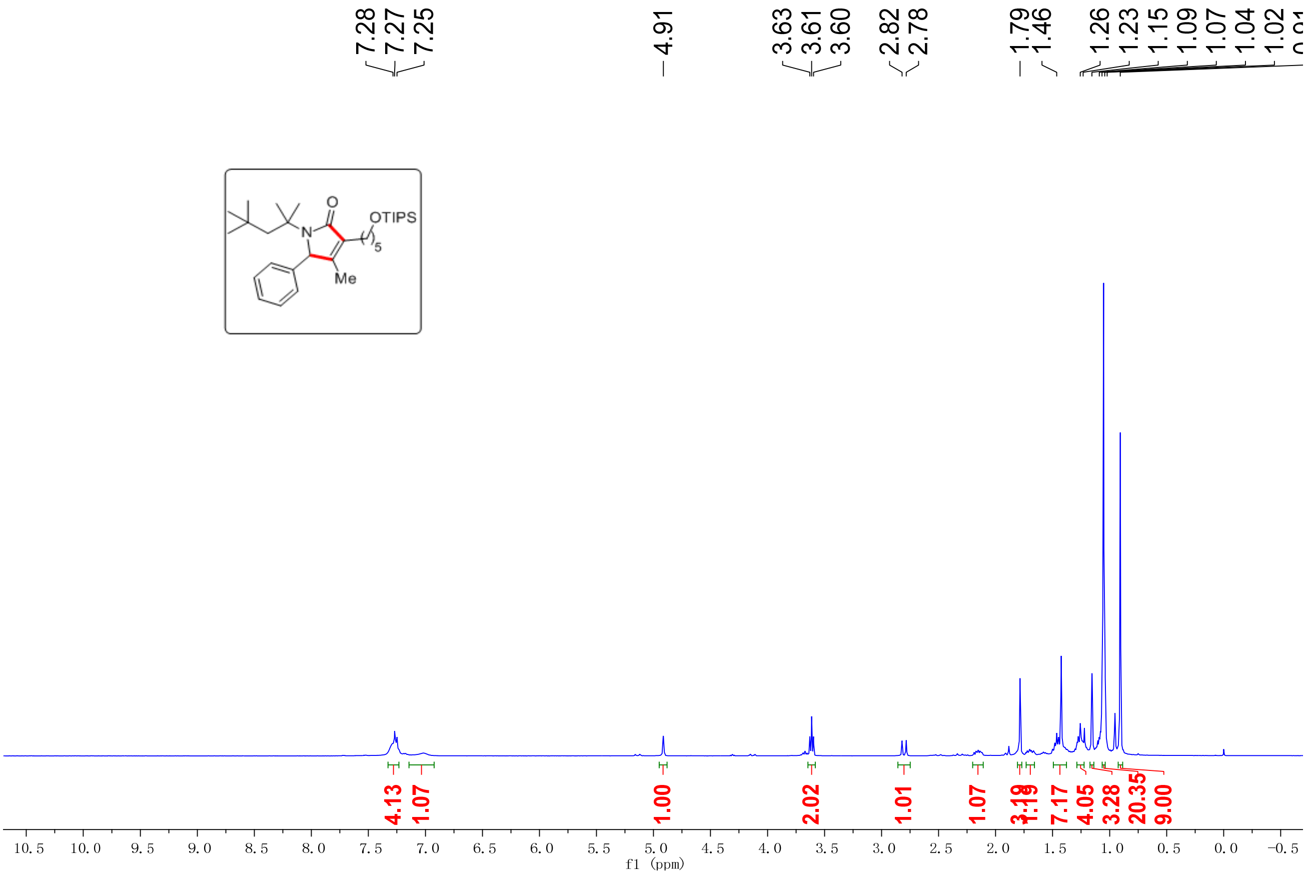

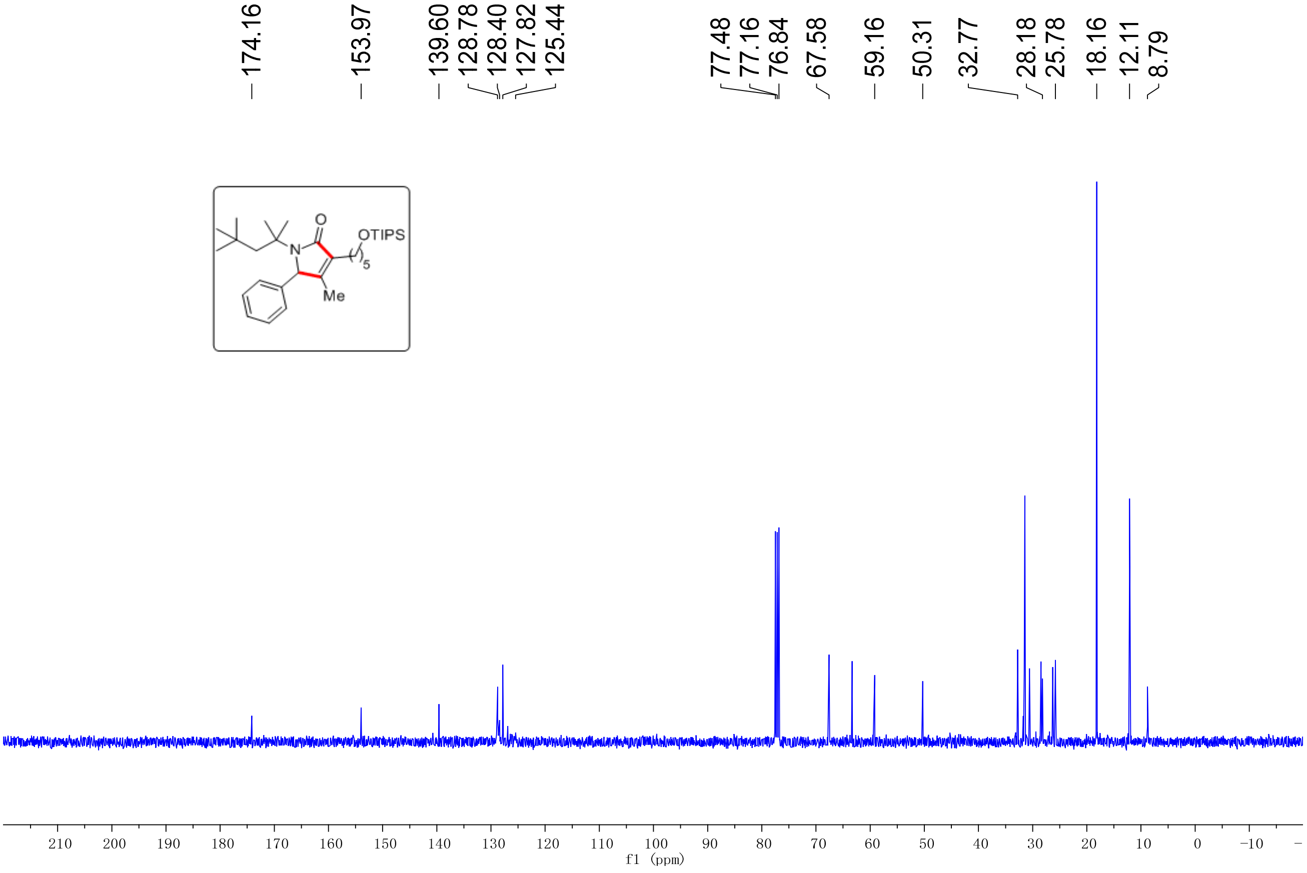

Supplementary Figure 91. ^{1}H and ^{13}C NMR spectra of compound **4j′** in $CDCl_3$

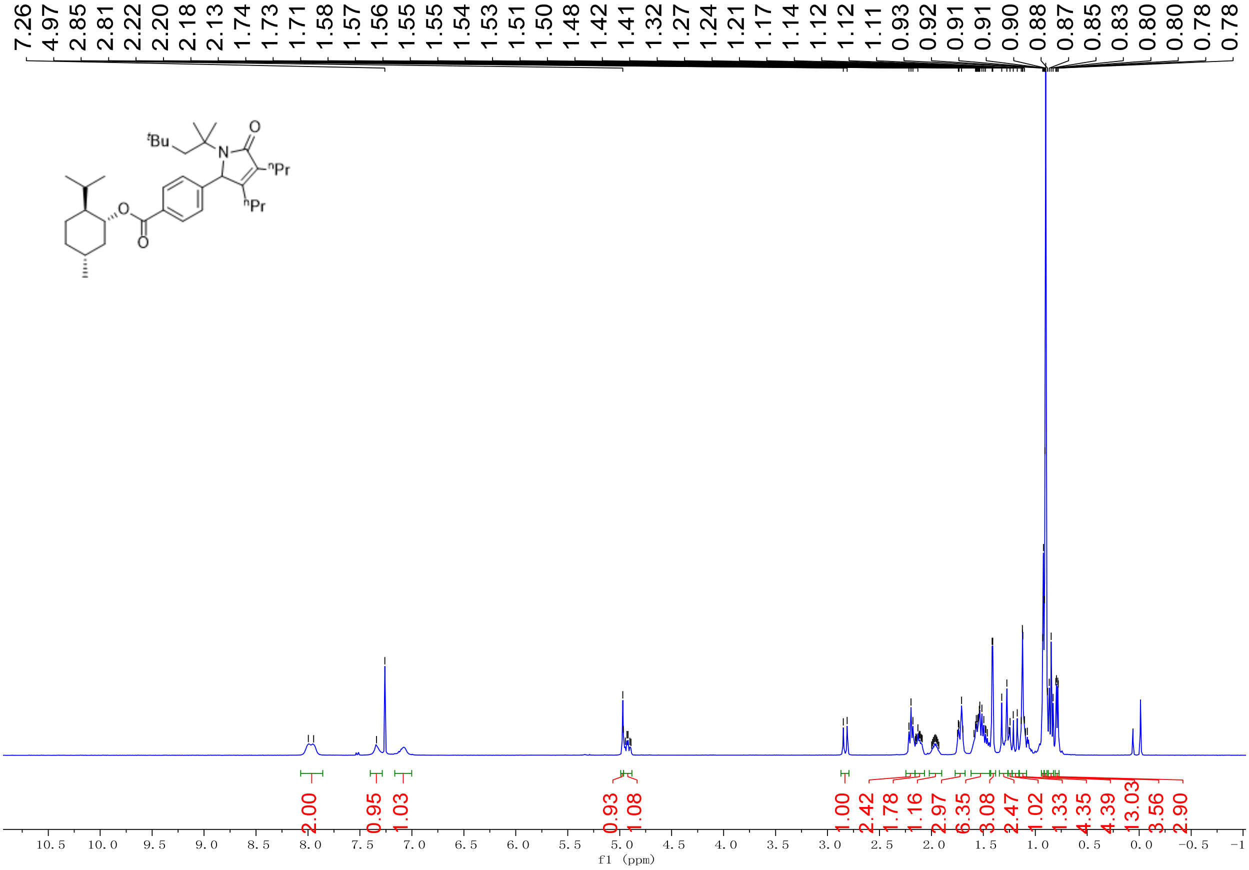

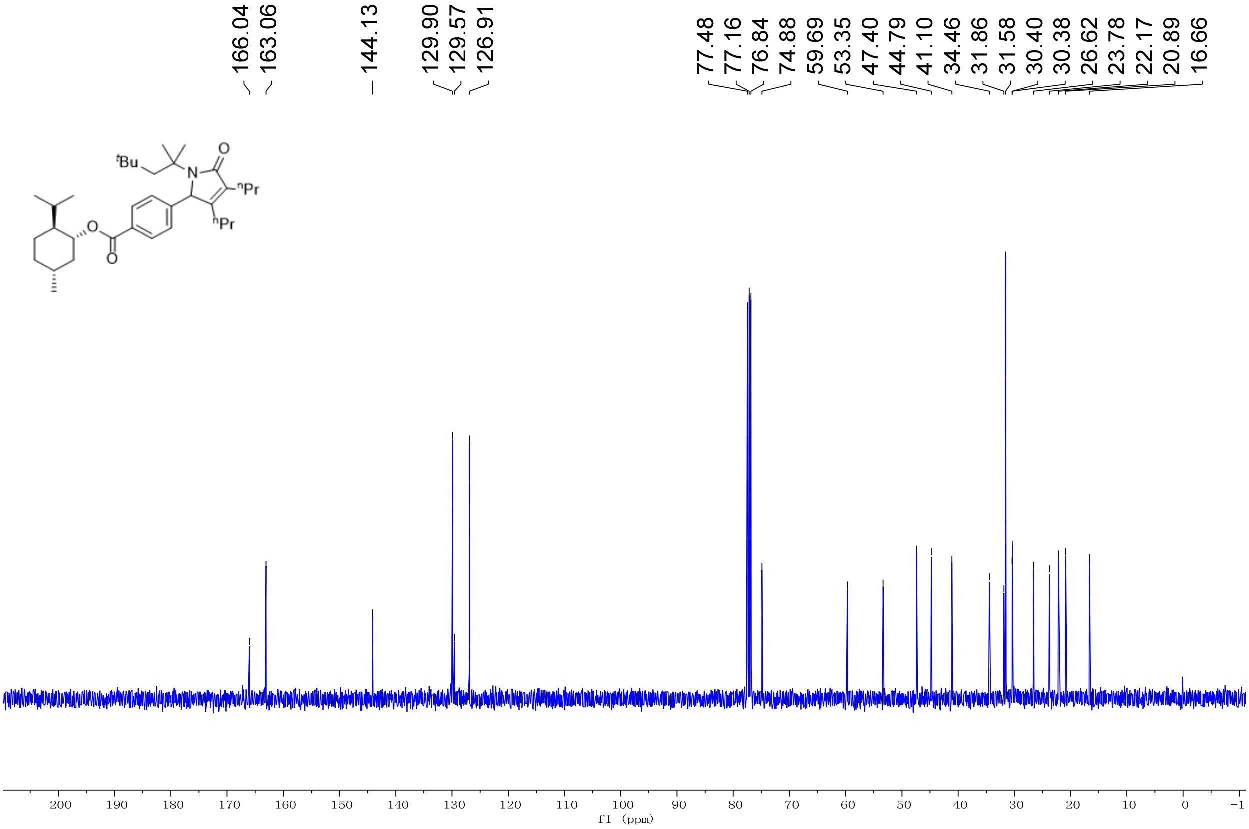

Supplementary Figure 92. ^{1}H and ^{13}C NMR spectra of compound **4k** in $CDCl_3$

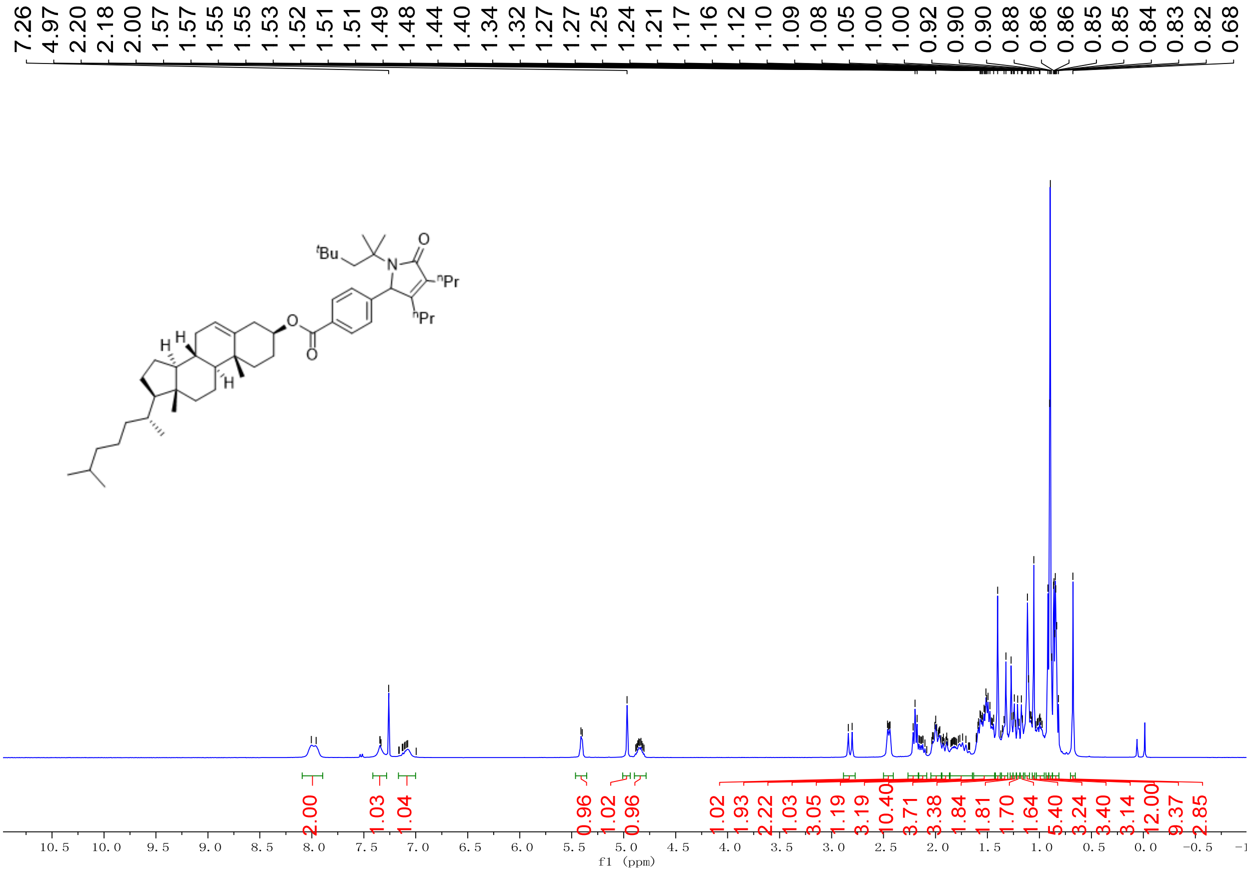

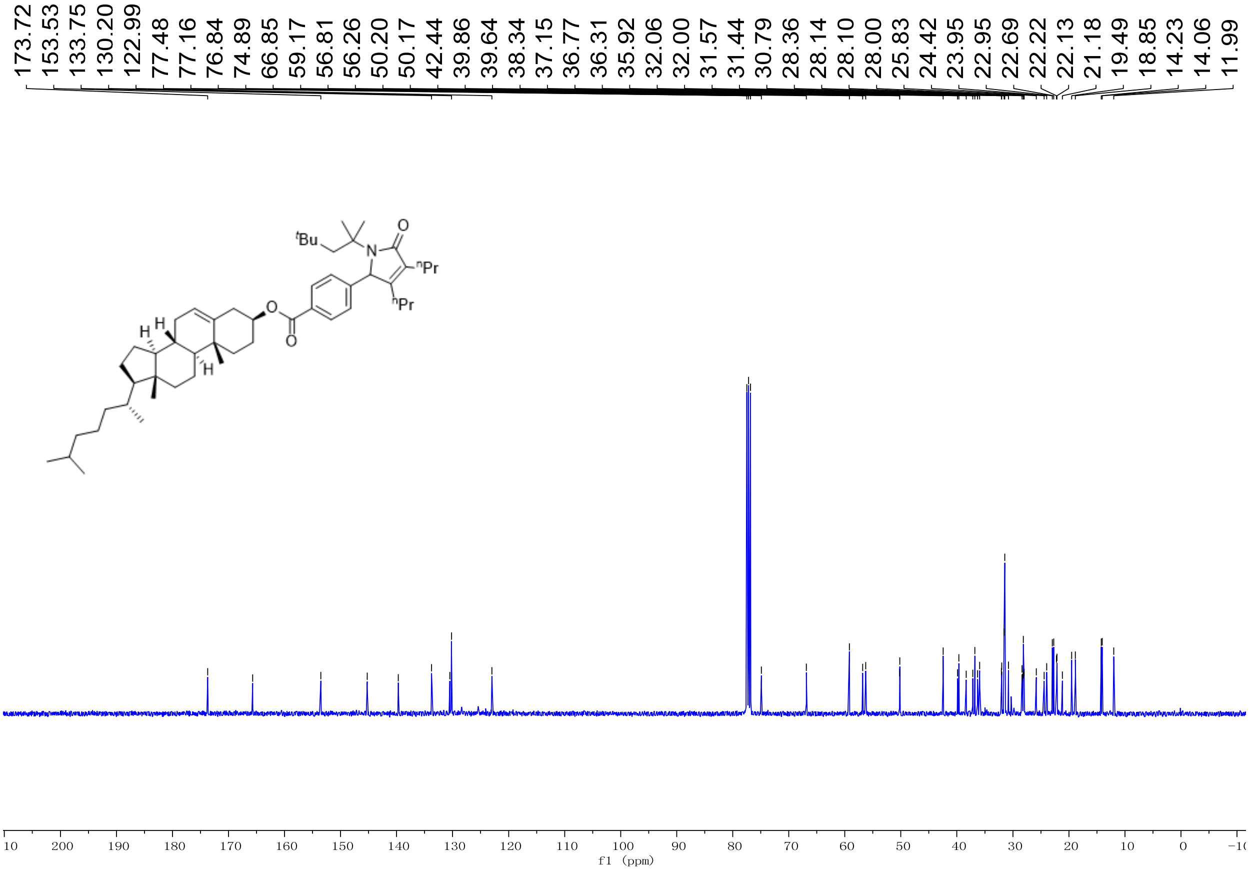

Supplementary Figure 93. ^{1}H and ^{13}C NMR spectra of compound **4l** in $CDCl_3$

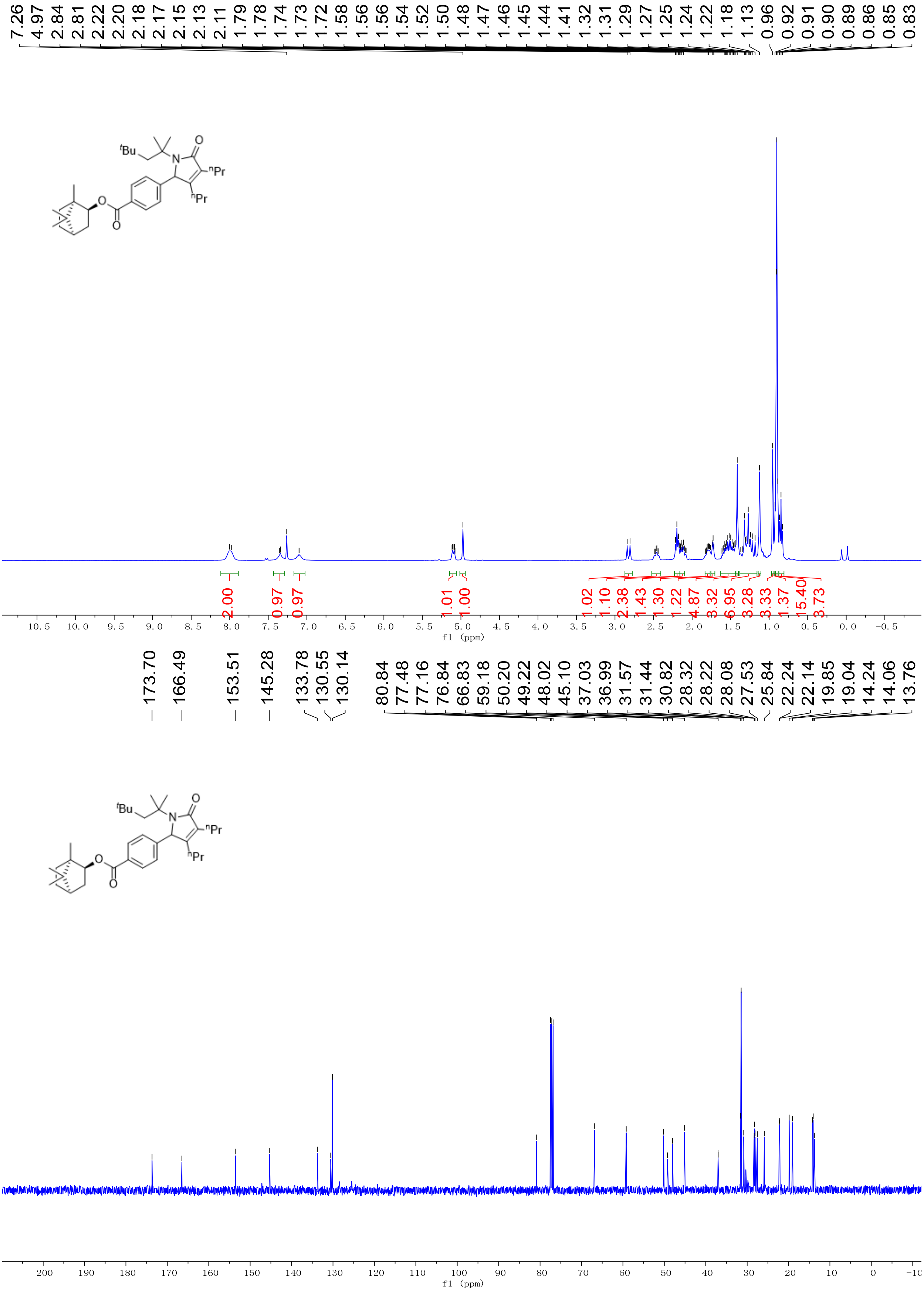

Supplementary Figure 94. ^{1}H and ^{13}C NMR spectra of compound **4m** in $CDCl_3$

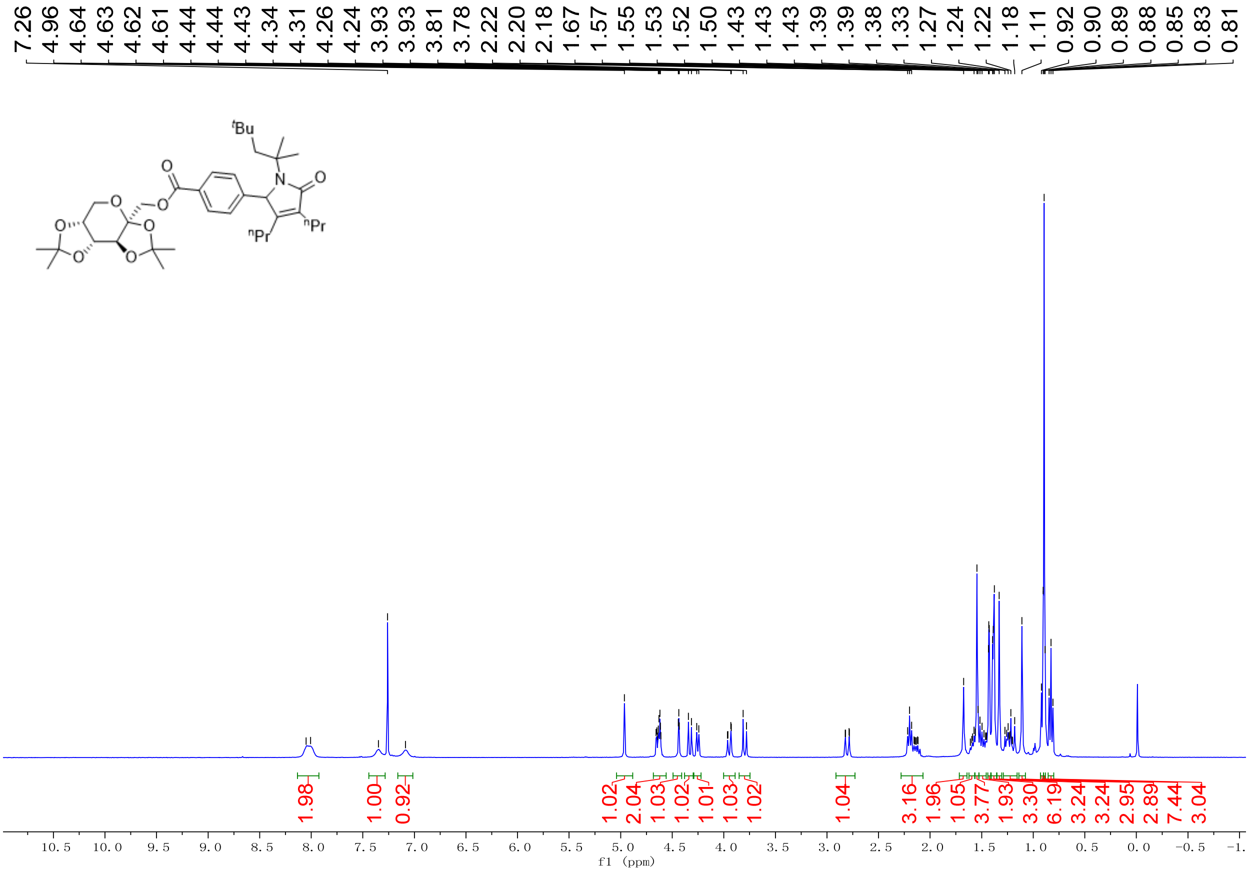

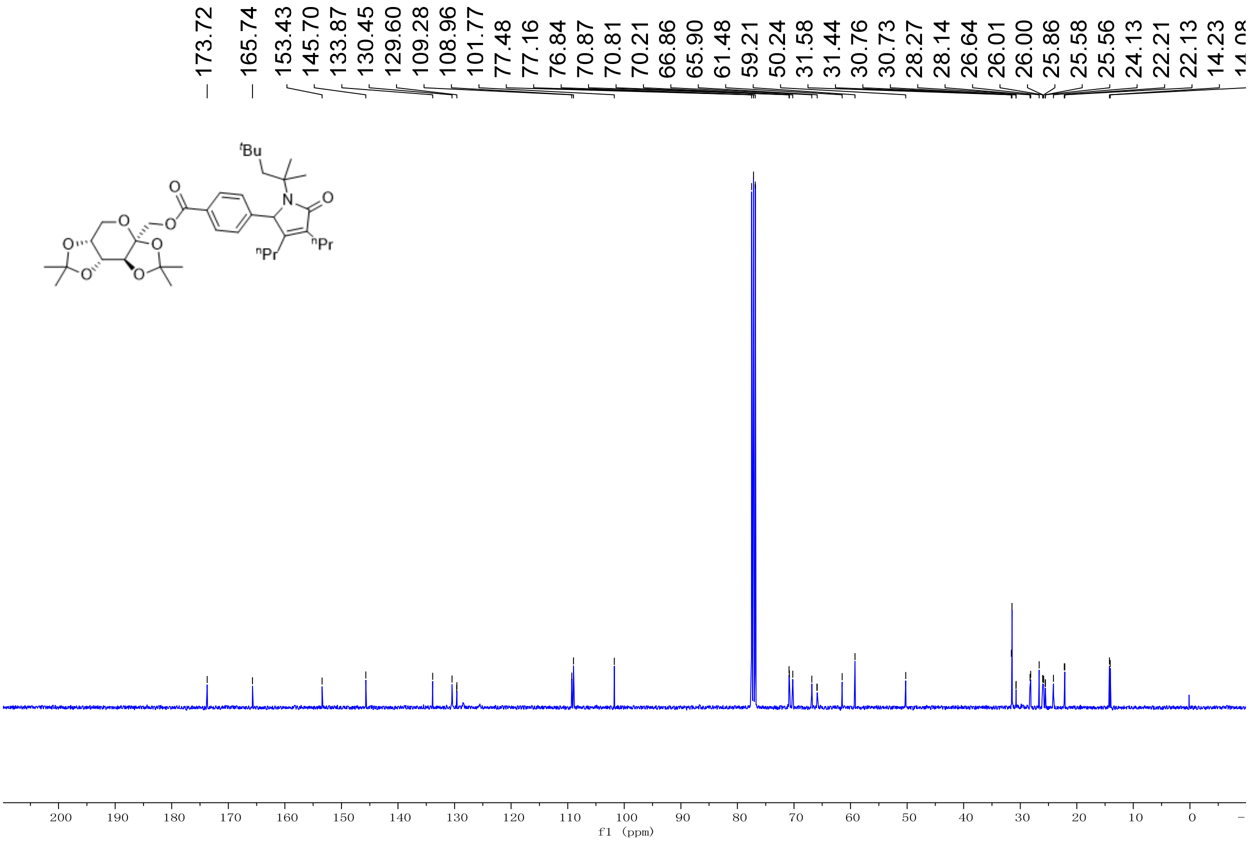

Supplementary Figure 95. ^{1}H and ^{13}C NMR spectra of compound **4n** in $CDCl_3$

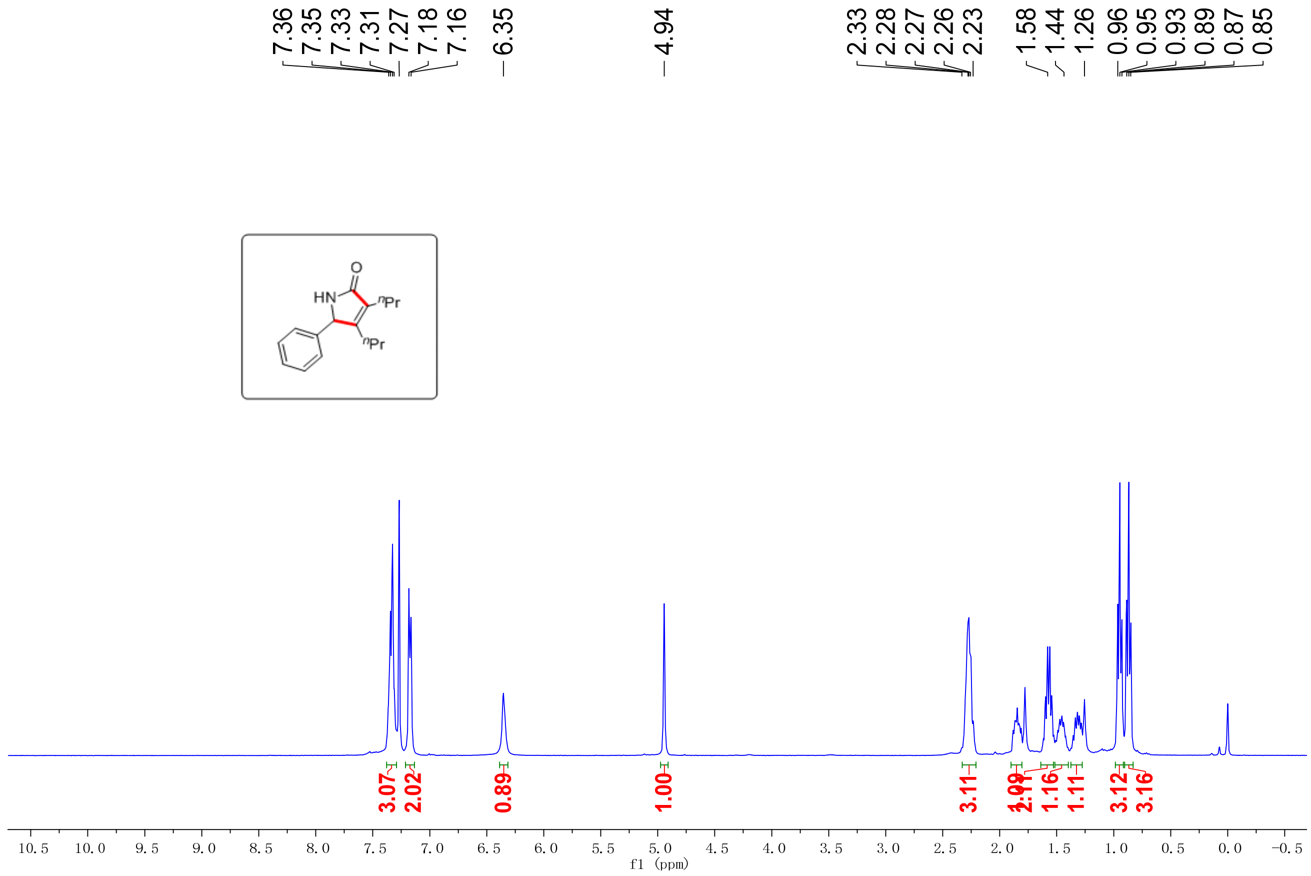

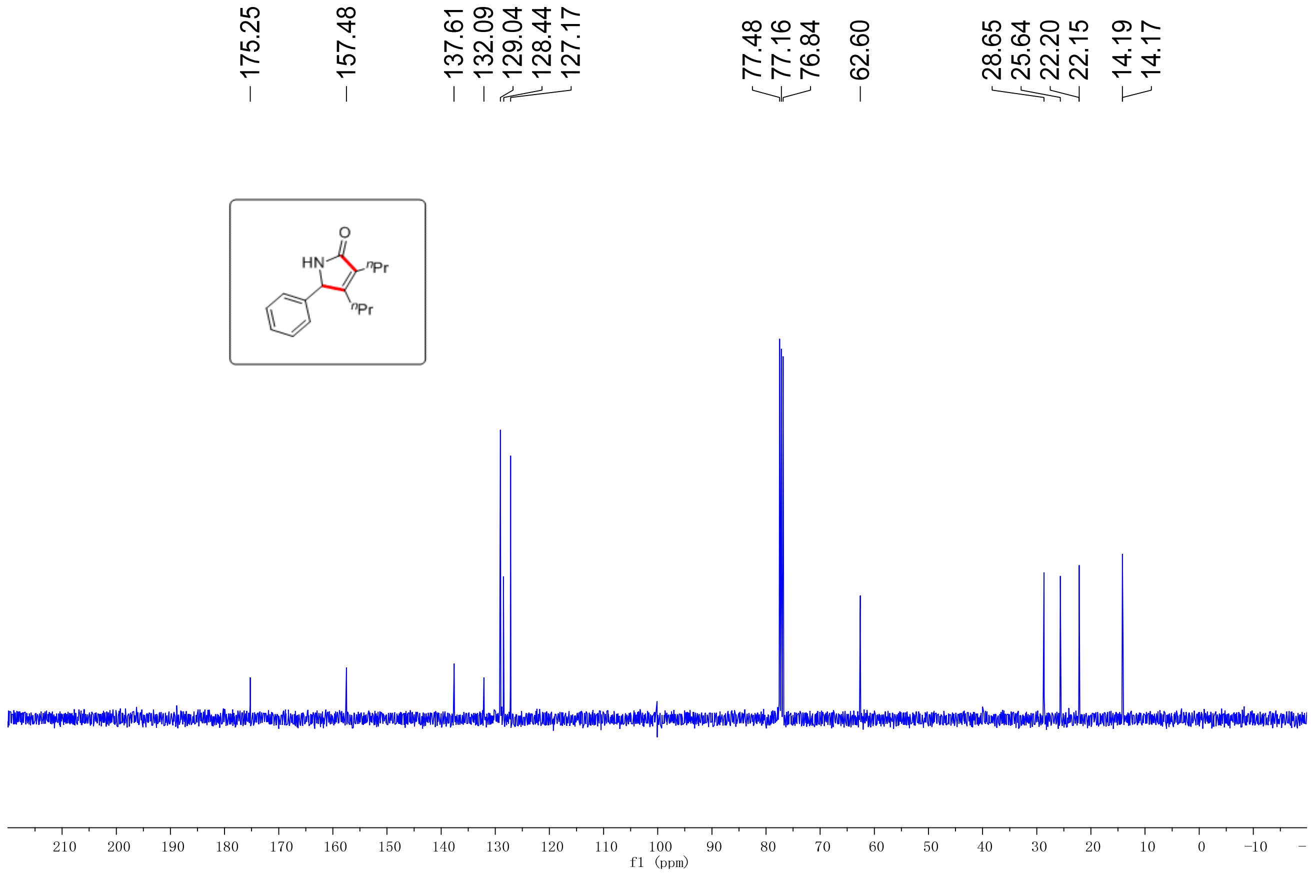

Supplementary Figure 96. ^{1}H and ^{13}C NMR spectra of compound **5** in $CDCl_3$

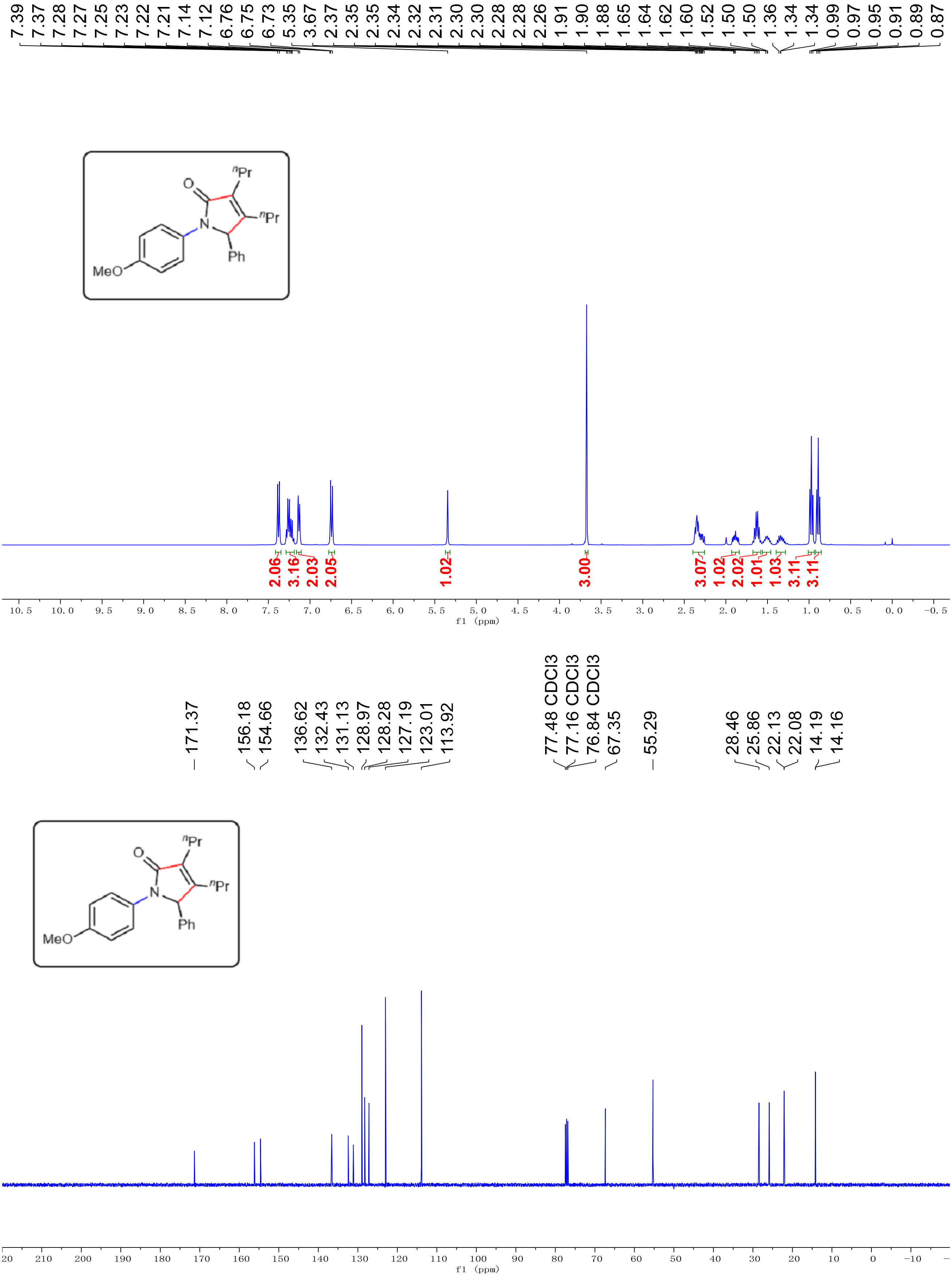

Supplementary Figure 97. ^{1}H and ^{13}C NMR spectra of compound **6** in $CDCl_3$

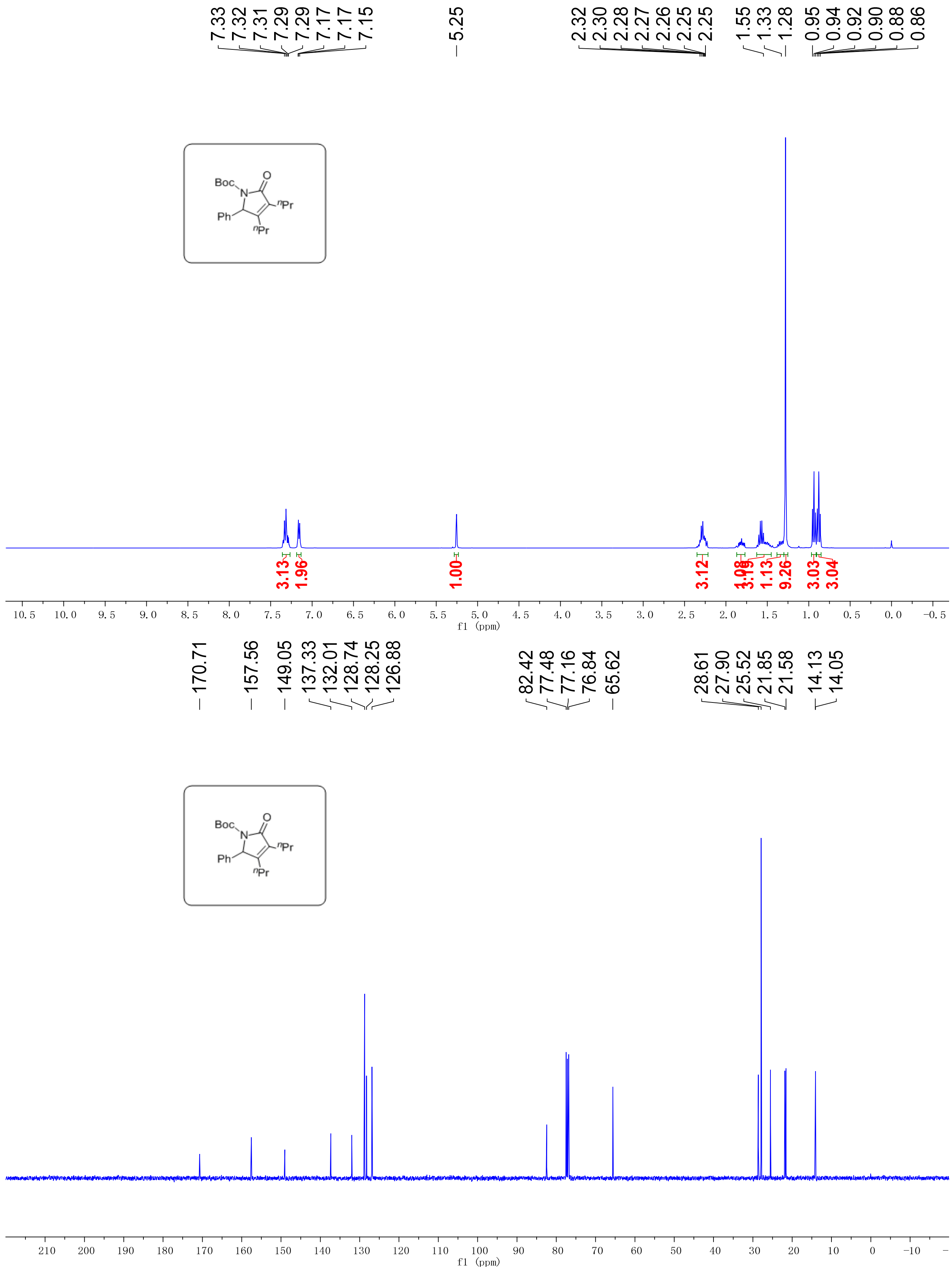

Supplementary Figure 98. ^{1}H and ^{13}C NMR spectra of compound **7** in $CDCl_3$

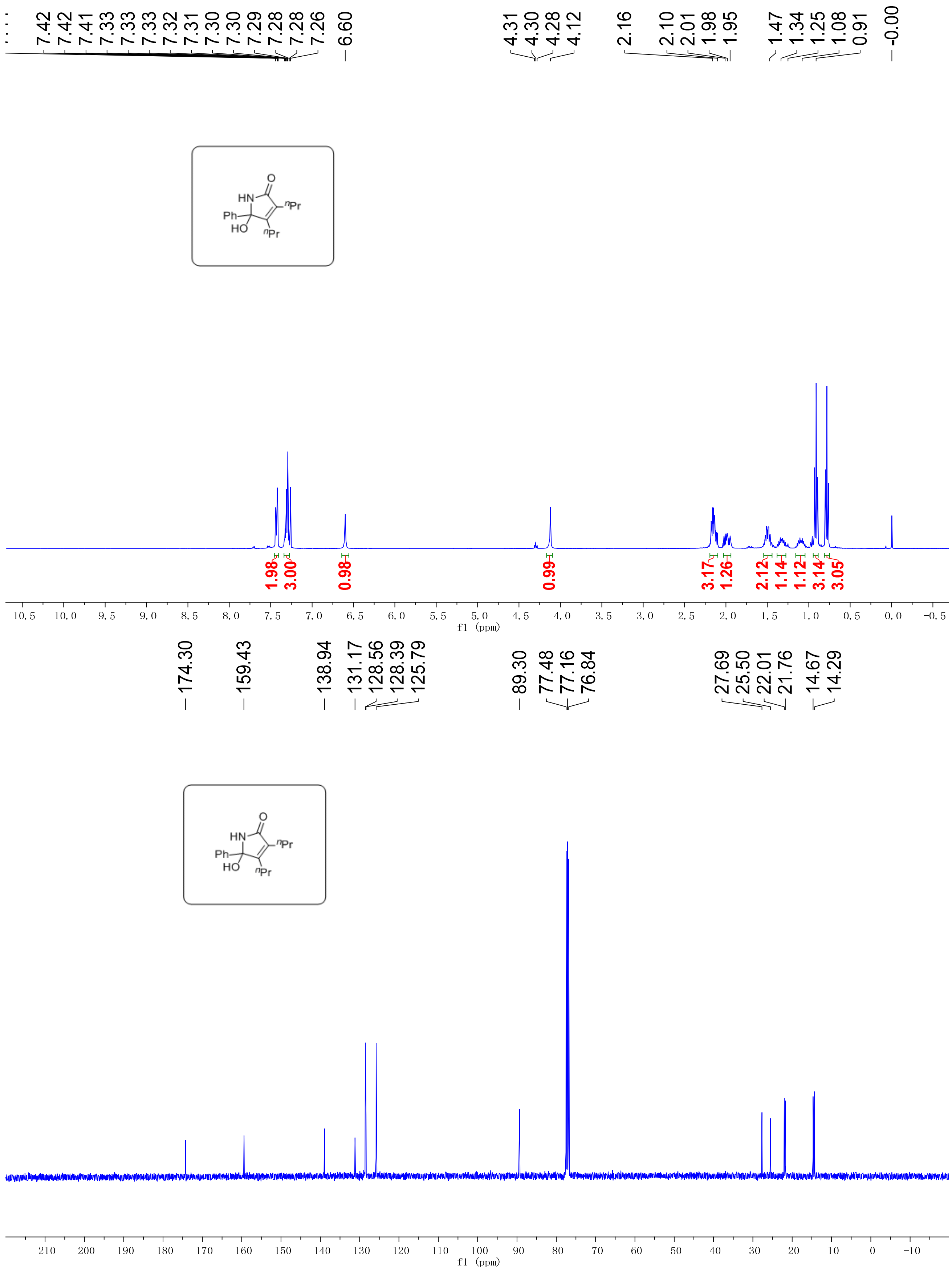

Supplementary Figure 99. ^{1}H and ^{13}C NMR spectra of compound **8** in $CDCl_3$

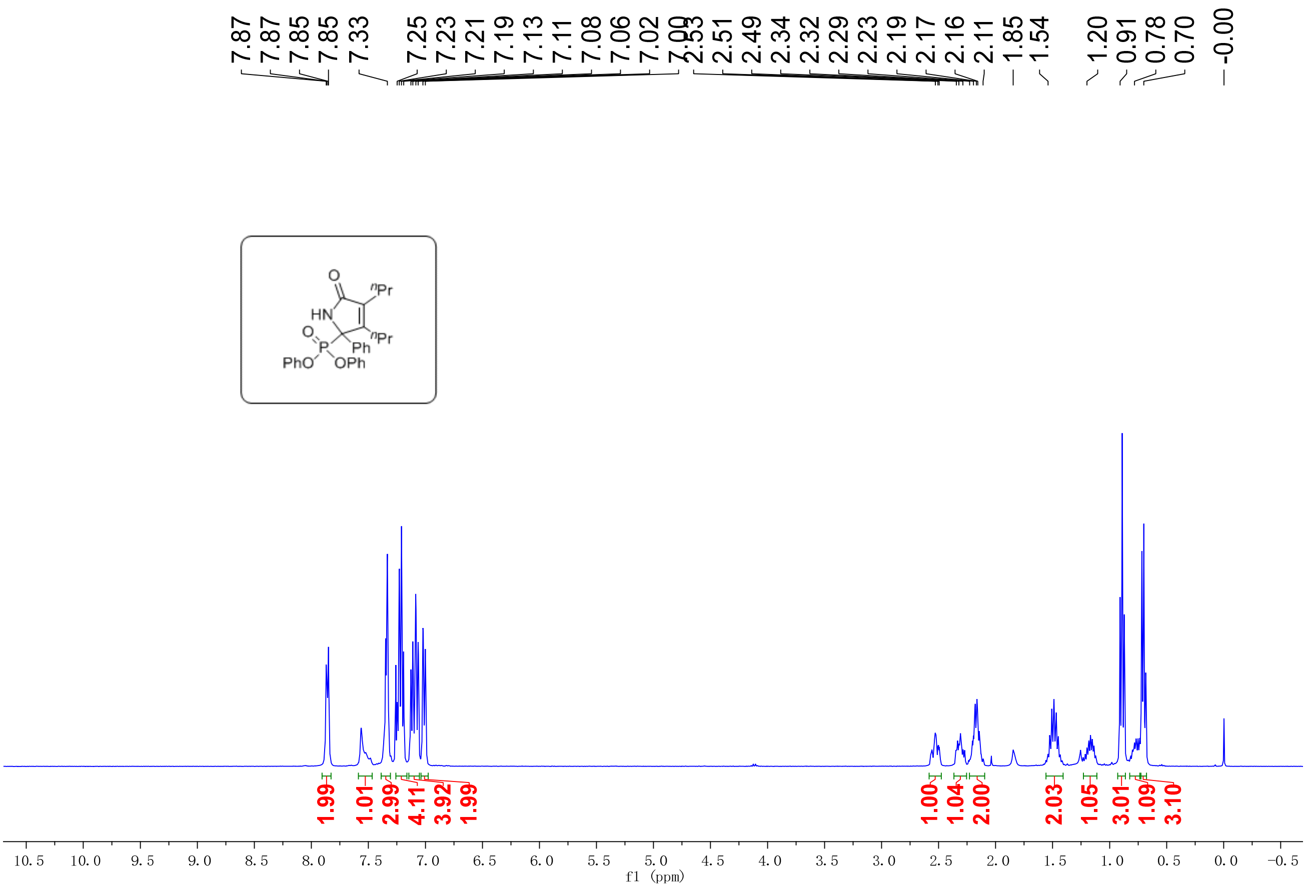

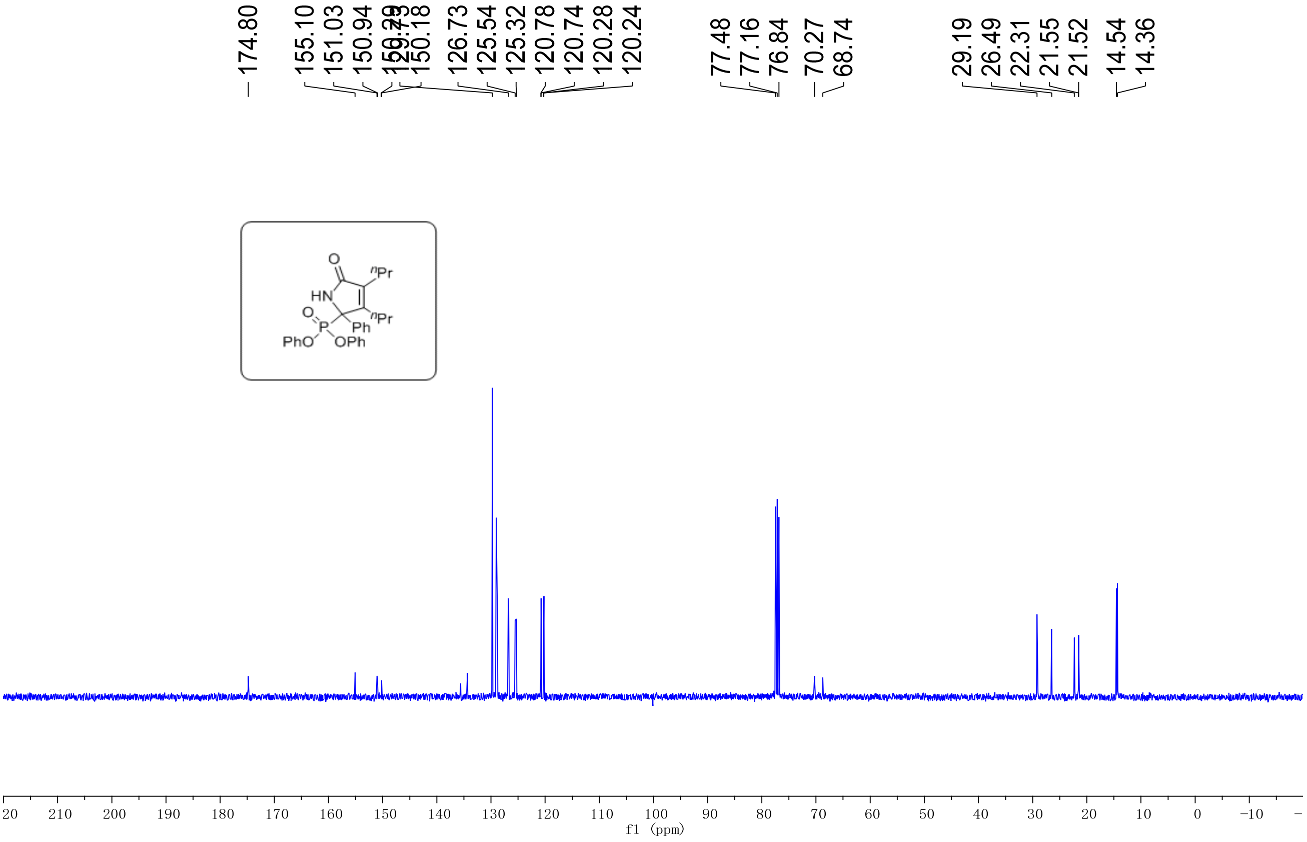

Supplementary Figure 100. ^{1}H and ^{13}C NMR spectra of compound **9** in $CDCl_3$

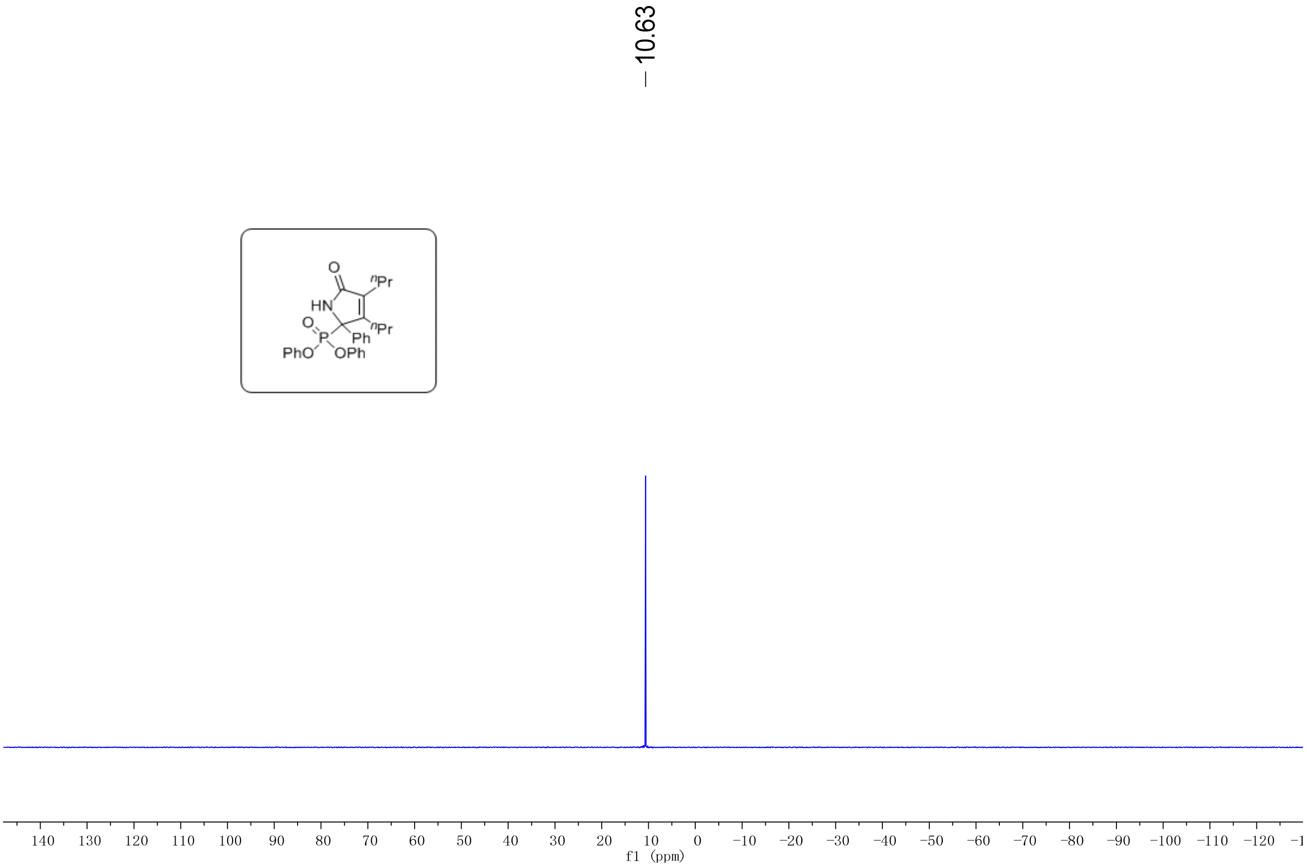

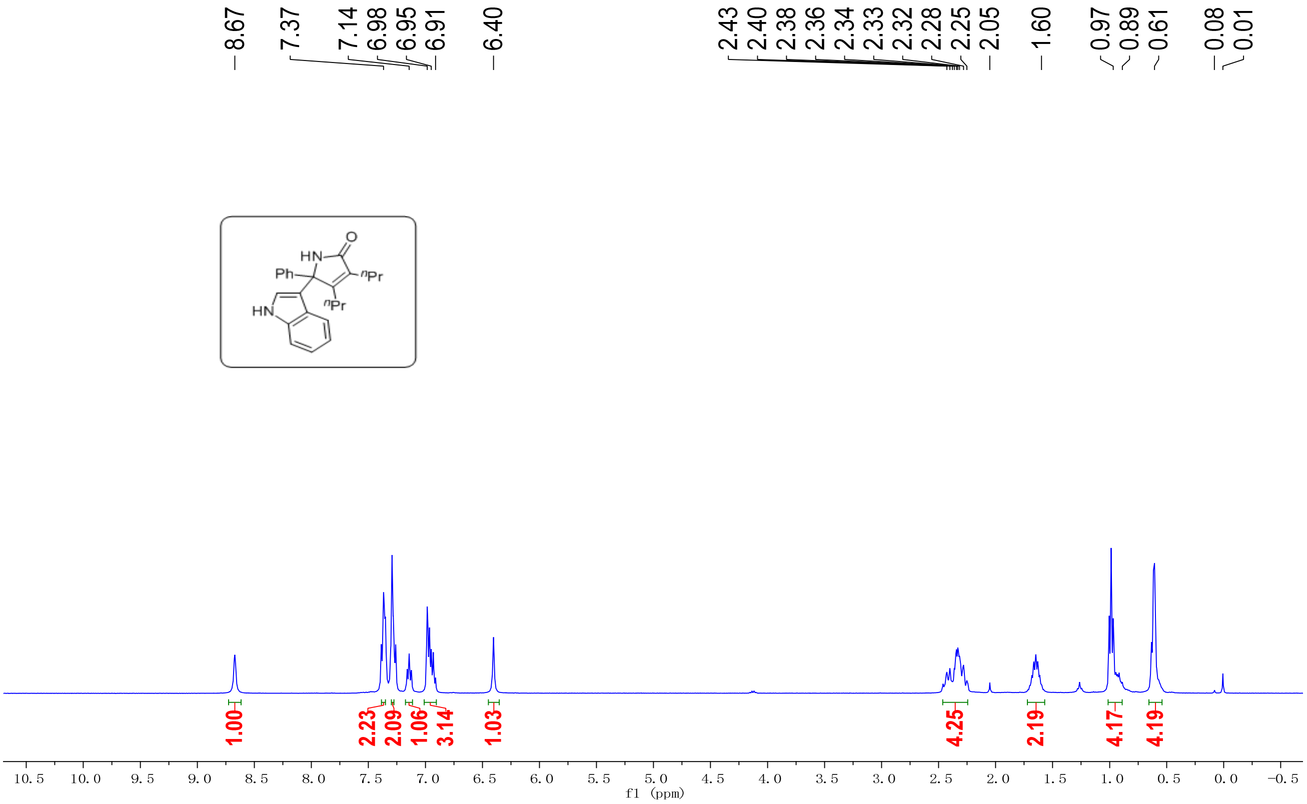

Supplementary Figure 101. ^{31}P (**9**) and ^{1}H NMR (**10**) spectra in $CDCl_3$

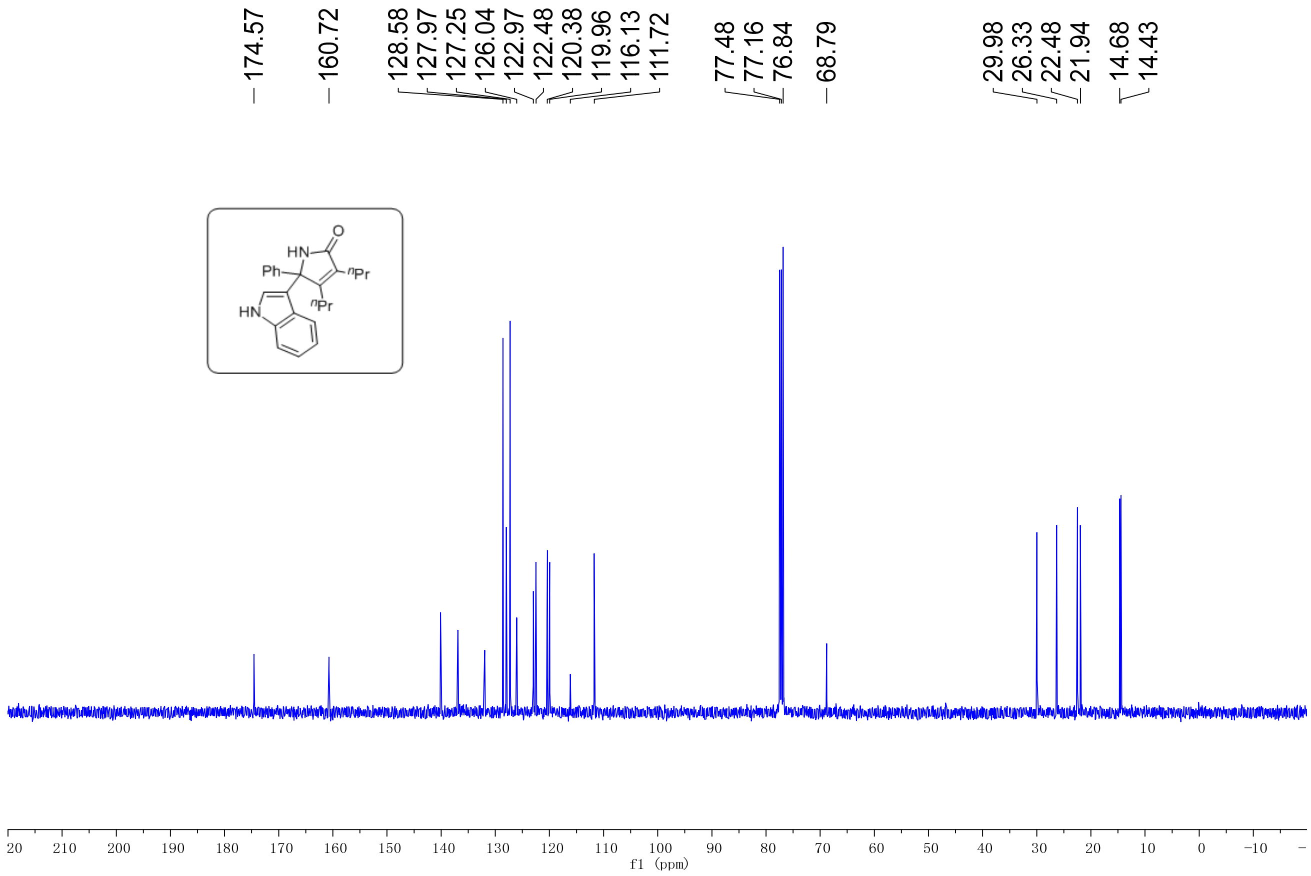

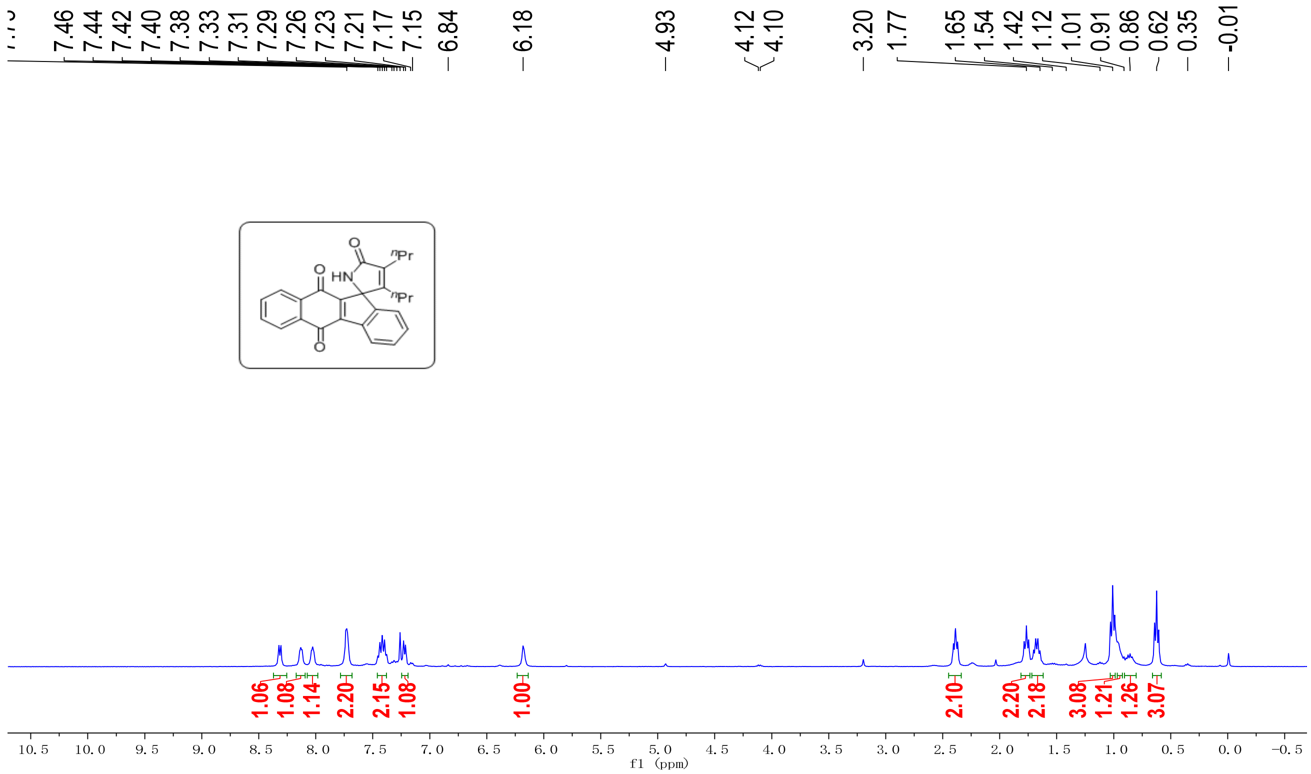

Supplementary Figure 102. ^{13}C (**10**) and ^{1}H NMR (**11**) spectrum of compound **11** in $CDCl_3$

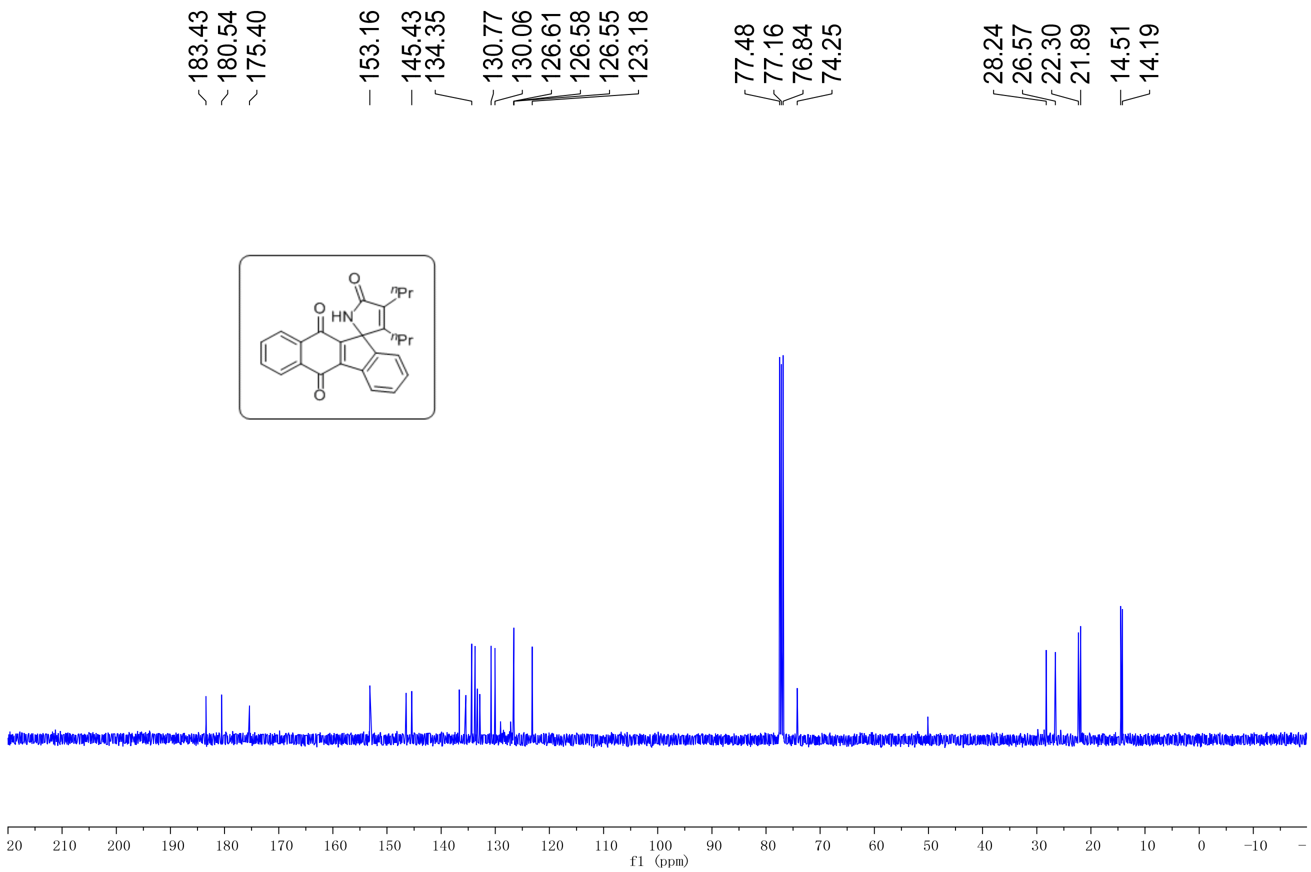

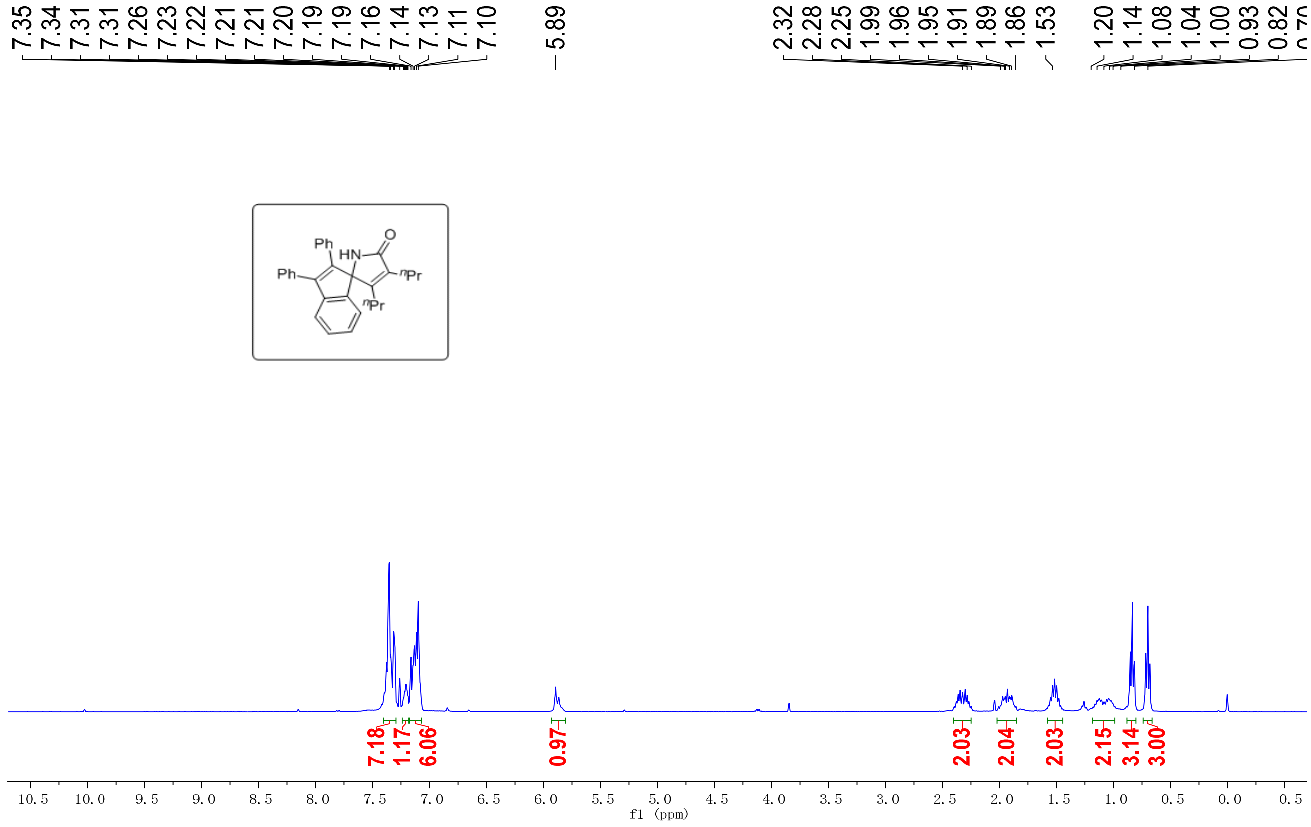

Supplementary Figure 103. ^{13}C (**11**) and ^{1}H NMR (**12**) spectra in $CDCl_3$

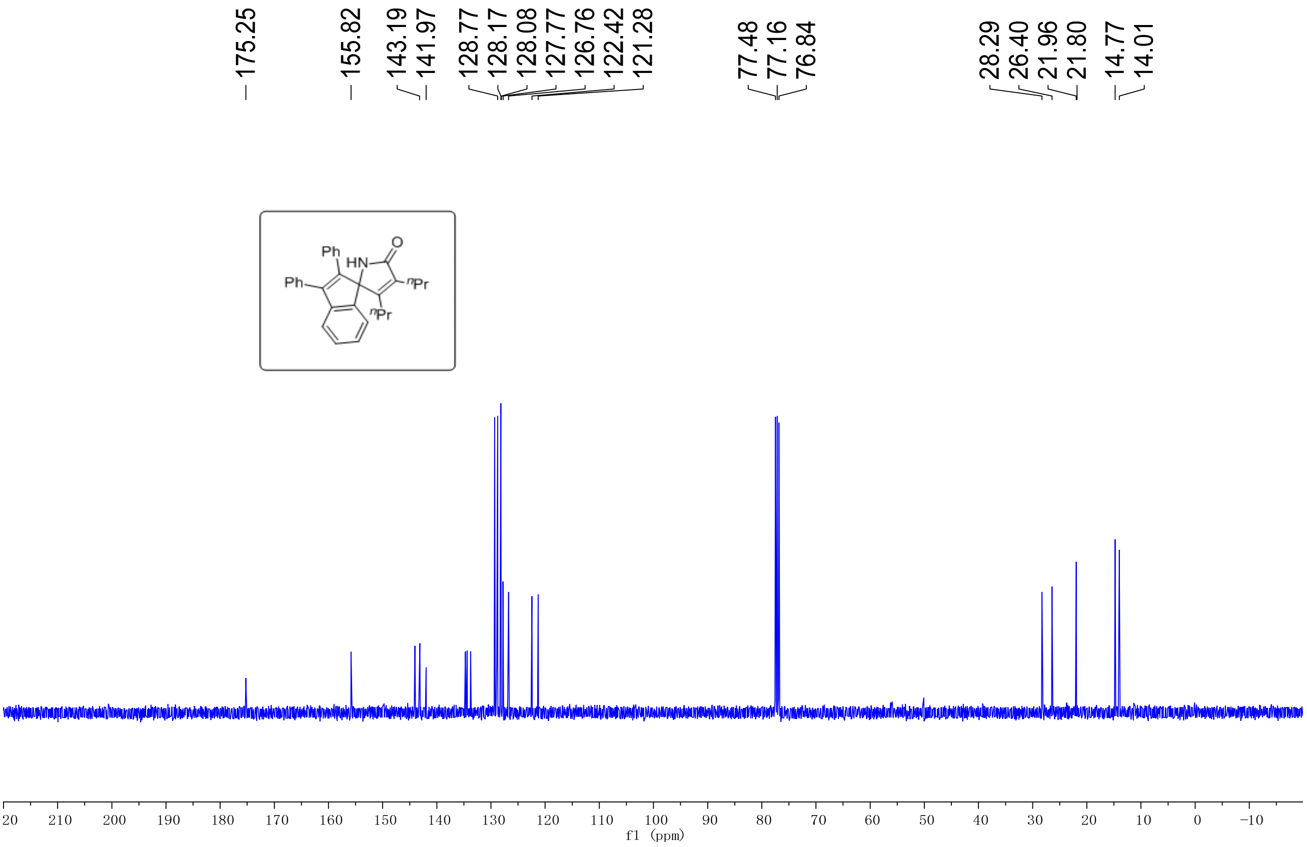

Supplementary Figure 104. ^{13}C NMR (**12**) spectrum in $CDCl_3$

Supplementary references

1 Ackermann, L. & Born, R. Modular Diamino- and Dioxophosphine Oxides and Chlorides as Ligands for Transition-Metal-Catalyzed C-C and C-N Couplings with Aryl Chlorides. *Angew. Chem. Int. Ed.* **44**, 2444−2447 (2005).

2 Donets P. A. & Cramer, N. Diaminophosphine Oxide Ligand Enabled Asymmetric Nickel-Catalyzed Hydrocarbamoylations of Alkenes. *J. Am. Chem. Soc.* **135**, 11772–11775 (2013).

3 Wang, Y.-X. et al. Enantioselective Ni–Al Bimetallic Catalyzed exo-Selective C–H Cyclization of Imidazoles with Alkenes. *J. Am. Chem. Soc.* **140**, 5360−5364 (2018).

4 Chen, H., Wang, Y.-X., Luan, Y.-X. & Ye, M. Enantioselective Twofold C−H Annulation of Formamides and Alkynes without Built-in Chelating Groups. *Angew. Chem. Int. Ed.* **59**, 9428–9432 (2020).

5 Munoz, A., Hubert, C. & Luche, J.-L. One-Pot Synthesis of Phosphonic Acid Diesters. *J. Org. Chem.* **61**, 6015–6017 (1996).

6 Cochet, T., Bellosta, V., Greiner, A., Roche, D. & Cossy, J. *N*-Formylsaccharin: A New Formylating Agent. *Synlett* **13**, 1920–1922 (2011).

7 Lee, C., Yang, W. & Parr, R. G. Development of the Colle-Salvetti Correlation-Energy Formula into a Functional of the Electron Density. *Phys. Rev. B*. **37**, 785–789 (1988).

8 Becke, A. D. Density Functional Thermochemistry. III. The Role of Exact Exchange. *J. Chem. Phys*. **98**, 5648–5652 (1993).

9 Grimme, S., Antony, J., Ehrlich, S. & Krieg, H. A Consistent and Accurate Ab Initio Parametrization of Density Functional Dispersion Correction (DFT-D) for the 94 Elements H-Pu. *J. Chem. Phys*. **132**, 154104 (2010).

10 Frisch, M. J. et al. Gaussian 09, Revision E.01; Gaussian, Inc.: Wallingford, CT, 2013.

11 For a very recent example, see: Ma, J.-B., Zhao, X., Zhang, D., Shi, S.-L. Enantio- and Regioselective Ni-Catalyzed *para*-C–H Alkylation of Pyridines with Styrenes via Intermolecular Hydroarylation. *J. Am. Chem. Soc*. **144**, 13643–13651 (2022).

12 Weigend, F. & Ahlrichs, R. Balanced Basis Sets of Split Valence, Triple Zeta Valence and Quadruple Zeta Valence Quality for H to Rn: Design and Assessment of Accuracy. *Phys. Chem. Chem. Phys*. **7**, 3297–3305 (2005).

13 Fukui, K. Formulation of the Reaction Coordinate. *J. Phys. Chem*. **74**, 4161–4163 (1970).

14 Fukui, K. The Path of Chemical Reactions-The IRC Approach. *Acc. Chem. Res*. **14**, 363–368 (1981).

15 Weigend, F. Accurate Coulomb-Fitting Basis Sets for H to Rn. Phys. *Chem. Chem. Phys*. **8**, 1057−1065 (2006).

16 Marenich, A. V., Cramer, C. J. & Truhlar, D. G. Universal Solvation Model Based on Solute Electron Density and on a Continuum Model of the Solvent Defined by the Bulk Dielectric Constant and Atomic Surface Tensions. *J. Phys. Chem. B*. **113**, 6378−6396 (2009).
