# Supplementary material for: Ni-catalyzed benzylic β-C(sp3)–H bond activation of formamides
Source: Nat Commun. 2022 Dec 22;13:7892. doi: 10.1038/s41467-022-35541-6 (PMC9780214; doi:10.1038/s41467-022-35541-6)
Supplement: Supplementary file 2 — Description of Additional Supplementary Files [file 41467_2022_35541_MOESM2_ESM.docx]

**Supplementary Data 1. Calculated energies of the stationary points.** Thermal corrections to Gibbs energies (TCGs) and single-point energies (SPEs). ^*^Computed at the B3LYP-D3/def2TZVPP level. ^†^Standard state at 1 atm and 298.15 K was used.
