# Supplementary material for: Ni-catalyzed benzylic β-C(sp3)–H bond activation of formamides
Source: Nat Commun. 2022 Dec 22;13:7892. doi: 10.1038/s41467-022-35541-6 (PMC9780214; doi:10.1038/s41467-022-35541-6)
Supplement: Supplementary file 3 — Supplementary Data 1 [file 41467_2022_35541_MOESM3_ESM.pdf]

Supplementary Data 1. Calculated energies of the stationary points. Thermal corrections to Gibbs energies (TCGs) and single-point energies (SPEs). *Computed at the B3LYP-D3/def2TZVPP level. †Standard state at 1 atm and 298.15 K was used.

**Stationary point**	**SPEs (a.u.)***	**TCGs (a.u.)*,†**
**1a**	-755.1391108	0.334969
**2a**	-313.3978724	0.160483
**IM0**	-2830.253197	0.394141
**olefin**	-314.6460811	0.183827
**3a**	-1067.402785	0.502962
**3a'**	-1067.396747	0.505307
**TS1**	-3585.455275	0.752051
**IM1**	-3585.467467	0.75228
**IM2**	-3585.464951	0.753225
**TS2**	-3898.893372	0.936444
**TS3**	-3898.891306	0.932108
**IM3**	-3898.894148	0.938803
**IM4**	-3898.924717	0.940696
**IM5**	-3898.944227	0.94162
**TS-iso**	-3898.897071	0.940198
**TS6**	-3898.896053	0.941444
**TS7**	-3898.902259	0.93607
**IM4'**	-3898.927449	0.945376
**IM9**	-3898.938535	0.942934
**TS3'**	-4212.301859	1.133667
**TS4**	-3897.667019	0.920577
**TS5**	-3897.699034	0.922185
**IM6**	-3897.698742	0.91541
**IM7**	-3897.713135	0.925201
**IM8**	-3897.734639	0.925681
**TS8**	-3897.677738	0.920362
**TS9**	-3897.696335	0.928427
**IM10**	-3897.69446	0.917673
**IM11**	-3897.7048	0.9261
**IM12**	-3897.72557	0.927107

Cartesian coordinates of the stationary points

1a

C	0.95499900	-1.70455200	-1.53521900
H	1.93127000	-1.91677700	-1.05521500
O	0.85283100	-1.57738100	-2.74340400
N	-0.07921900	-1.59980300	-0.65056700
C	0.08186100	-1.84703800	0.81570200

C	-0.13616900	-3.37742100	1.00942100
C	1.48770200	-1.40085700	1.26119900
C	-0.92743300	-0.96989900	1.57158600
H	-1.01604000	-3.65312300	0.40538600
H	0.72133600	-3.85841900	0.50863100
H	1.71058000	-0.39875500	0.86562600
H	1.53235900	-1.34583900	2.35474400
H	2.28009000	-2.09017600	0.93927900
H	-0.75122200	-1.04777400	2.65226200
H	-0.80648000	0.08266100	1.27975000
H	-1.96914000	-1.25481200	1.38149100
C	-1.41919600	-1.48113600	-1.23103600
H	-1.34249100	-1.87325900	-2.25592900
H	-2.11559800	-2.12774400	-0.67772800
C	-1.96025000	-0.06766900	-1.28882600
C	-1.19562500	0.95893400	-1.86388400
C	-3.23463800	0.23522100	-0.79503200
C	-1.69379300	2.26142100	-1.92624500
H	-0.21425900	0.72272000	-2.27959300
C	-3.73760200	1.53785300	-0.86058800
H	-3.84091300	-0.55719200	-0.34622000
C	-2.96522700	2.55660900	-1.42263600
H	-1.08835500	3.05132400	-2.37784400
H	-4.73322000	1.75714500	-0.46689700
H	-3.35293200	3.57701900	-1.47175200
C	-0.32641500	-4.07060800	2.38768000
C	0.75312200	-3.70017800	3.41710100
C	-1.71920600	-3.79642700	2.98581300
C	-0.22640200	-5.58507800	2.11153200
H	1.76635700	-3.84521600	3.01093600
H	0.66594400	-2.65697200	3.75380400
H	0.65722100	-4.33802600	4.31047100
H	-2.51480400	-4.02702900	2.25876300
H	-1.88366900	-4.43190800	3.87082100
H	-1.84562900	-2.75399000	3.30494700
H	-0.40149500	-6.16392100	3.03225500
H	-0.97249400	-5.90383900	1.36579000
H	0.76994200	-5.85506200	1.72649100

2a

C	-0.00861800	1.01330200	-0.24894700
C	1.20429600	1.00343200	-0.26120400
C	2.66650600	1.02342700	-0.24024400
C	3.25447100	1.97614800	0.81625900

H	3.04487400	1.30948700	-1.23882300
H	3.04584500	0.00090800	-0.05921300
C	4.78111300	1.97534700	0.81928900
H	2.87399900	2.99404500	0.62747900
H	2.87109400	1.68583700	1.80886300
H	5.18209900	2.66274300	1.58037700
H	5.18052700	0.97003300	1.03386500
H	5.18390800	2.28804700	-0.15857200
C	-1.47082900	0.99340100	-0.26984600
C	-2.05890500	0.04096900	-1.32657000
H	-1.85010000	2.01599200	-0.45058700
H	-1.84917600	0.70708200	0.72866400
C	-3.58554800	0.04152400	-1.32921400
H	-1.67582900	0.33173800	-2.31915800
H	-1.67821900	-0.97693900	-1.13829200
H	-3.98661600	-0.64563400	-2.09047300
H	-3.98803400	-0.27163600	-0.35137200
H	-3.98519200	1.04685400	-1.54328400

3a

C	-5.25782200	3.30833900	2.14538100
O	-5.72391500	4.12220800	2.92942200
N	-4.28570000	2.35664000	2.41023100
C	-3.88054200	1.98029500	3.79798900
C	-3.34885800	3.25167000	4.48539900
C	-5.17170900	1.41037200	4.45906200
C	-2.73732700	0.96075700	3.74160000
H	-2.55560700	3.69241900	3.86219600
H	-4.14357300	3.99593600	4.60511900
H	-2.91604100	3.01641300	5.46509400
H	-5.60477700	0.70513900	3.72800400
H	-5.87247200	2.25642300	4.50740700
H	-2.39142800	0.75359700	4.76206300
H	-3.03699100	0.00504800	3.28944600
H	-1.88453100	1.35780500	3.17662300
C	-4.08896300	1.49731600	1.25107200
H	-4.39699100	0.45862200	1.47505900
C	-2.66703700	1.47568700	0.71004200
C	-2.11976900	0.29652800	0.19671600
C	-1.92358400	2.66105300	0.63951600
C	-0.84803000	0.29736600	-0.38537400
H	-2.69105600	-0.63395500	0.25679200
C	-0.65247500	2.66423400	0.06558700
H	-2.35397400	3.58077700	1.04119300

C	-0.11112500	1.48157000	-0.45251400
H	-0.43062400	-0.63158100	-0.78135400
H	-0.08015700	3.59388400	0.01808400
H	0.88445500	1.48432600	-0.90238000
C	-5.66932900	3.13961800	0.72140400
C	-5.02949400	2.07511900	0.20509300
C	-6.64439100	4.08352100	0.09930300
C	-6.02996700	5.46537500	-0.18300500
H	-7.48979800	4.21714100	0.79581700
H	-7.04955200	3.65670600	-0.83322600
C	-7.03467400	6.44892300	-0.77751100
H	-5.62690900	5.86263100	0.76258700
H	-5.17016300	5.34415700	-0.86495400
H	-6.57350900	7.43058200	-0.96948400
H	-7.44251500	6.07823300	-1.73313100
H	-7.88551200	6.60707800	-0.09452500
C	-5.10010000	1.52144800	-1.18516200
C	-4.25293000	2.30035700	-2.20874700
H	-6.15121100	1.52153900	-1.52142000
H	-4.77488600	0.46734800	-1.18140200
C	-4.32687000	1.69826500	-3.60928200
H	-4.59672900	3.34827800	-2.22567600
H	-3.20709600	2.32755500	-1.86593200
H	-3.72499200	2.27662500	-4.32759700
H	-3.94916900	0.66230600	-3.61593400
H	-5.36410200	1.67456300	-3.98349400
C	-5.20899100	0.68833900	5.83546400
C	-4.64207300	-0.74184500	5.76377900
C	-4.49672000	1.46512300	6.95422500
C	-6.70282600	0.57872600	6.20640600
H	-5.11823800	-1.31506400	4.95153600
H	-3.55729600	-0.76047400	5.59712300
H	-4.83713100	-1.27720200	6.70730600
H	-4.84390500	2.50869000	7.00268200
H	-4.70150800	0.99580400	7.93029200
H	-3.40507500	1.47518000	6.82156600
H	-6.83408900	0.02864100	7.15220100
H	-7.15603300	1.57554700	6.32623800
H	-7.26814900	0.04532700	5.42490000

3a′

C	-4.46248100	4.82403400	2.11281300
O	-4.12345300	5.77371300	2.81863500
N	-4.01707000	3.54582900	2.37064300

C	-4.29771700	2.40681200	1.44591400
C	-4.20837300	1.09822900	2.27696700
C	-5.71142200	2.60452200	0.88897700
C	-3.25442800	2.45194200	0.31338200
H	-3.14944400	0.95877600	2.53791800
H	-4.72278300	1.29338800	3.22868200
H	-3.41724800	1.66081600	-0.42817700
H	-3.30046100	3.42013200	-0.20510000
H	-2.23868700	2.32903300	0.71891700
C	-2.95857800	3.42957800	3.37681300
H	-2.16704000	2.76530700	3.00207000
H	-2.52867900	4.43506400	3.48573800
C	-3.43165300	2.95557000	4.73514300
C	-2.80691700	1.88457000	5.38500800
C	-4.50655600	3.59735000	5.36865300
C	-3.24936300	1.44995100	6.63794000
H	-1.96989500	1.37441000	4.89947900
C	-4.95301900	3.16274200	6.61774800
H	-4.97860800	4.45030800	4.87719700
C	-4.32848200	2.08572200	7.25622400
H	-2.75276000	0.60902400	7.12860700
H	-5.79118500	3.67189900	7.10033000
H	-4.68062900	1.74600400	8.23327700
C	-5.37326500	5.04227200	0.95021000
C	-5.98878100	3.99046300	0.37549200
H	-5.88002400	1.87255800	0.08686900
H	-6.45002200	2.37529700	1.67717100
C	-5.61134800	6.48543100	0.58469600
C	-6.70434900	7.14934200	1.43741600
H	-5.87450500	6.56849800	-0.48179100
H	-4.67319500	7.04249300	0.72953300
C	-6.91565700	8.62020700	1.08720000
H	-7.64924400	6.59117000	1.31196600
H	-6.41862200	7.04996100	2.49604300
H	-7.70203300	9.07579300	1.70976000
H	-5.99038400	9.19961200	1.24083300
H	-7.21135000	8.74456200	0.03143100
C	-6.96849000	4.10013000	-0.76200800
C	-6.30040500	3.86834200	-2.12869500
H	-7.46569300	5.08144400	-0.76290300
H	-7.76534100	3.34719900	-0.62300500
C	-7.29090800	3.89565500	-3.29034900
H	-5.52308200	4.63779400	-2.27414000
H	-5.76642500	2.90197500	-2.11456800

H	-6.78521000	3.73789000	-4.25587200
H	-8.05695100	3.11011200	-3.18058400
H	-7.81676800	4.86331200	-3.34219300
C	-4.71555400	-0.28943200	1.78434100
C	-6.25155400	-0.41183400	1.81436300
C	-4.20442500	-0.68561200	0.39016400
C	-4.15557200	-1.30265700	2.80604900
H	-6.65810400	-0.07704800	2.78195800
H	-6.74215800	0.16725900	1.02178400
H	-6.54707000	-1.46437600	1.67577000
H	-3.10838600	-0.60328400	0.32061000
H	-4.47450300	-1.73190600	0.17397000
H	-4.64441700	-0.06669700	-0.40585100
H	-4.50630100	-2.32217800	2.58063900
H	-3.05386600	-1.31314500	2.79257600
H	-4.47473400	-1.05063900	3.82988000

olefin

H	-5.25257500	4.72587000	-1.11186700
H	-3.22994300	5.45946900	-0.16082700
C	-3.16834200	4.83662000	-1.06246200
C	-4.32125500	4.41861300	-1.60462400
C	-4.49822000	3.52784200	-2.80183300
C	-5.00765300	2.12514900	-2.43043000
H	-3.55578800	3.43024100	-3.36431800
H	-5.22383400	3.99197900	-3.49555900
C	-5.24590600	1.23288300	-3.64698200
H	-4.27682200	1.65197000	-1.75162900
H	-5.94100100	2.22069200	-1.84740000
H	-5.60286900	0.23305400	-3.35334200
H	-5.99886300	1.67182400	-4.32275900
H	-4.31963400	1.09886500	-4.23049600
C	-1.77386600	4.51488500	-1.52000400
C	-1.03327500	3.57265900	-0.55628400
H	-1.19443400	5.45291800	-1.60709700
H	-1.78265900	4.06565500	-2.52607300
C	0.40272800	3.28221200	-0.98790600
H	-1.03778600	4.01302000	0.45679100
H	-1.60220500	2.62992700	-0.47640700
H	0.90821000	2.60238300	-0.28389800
H	0.43180700	2.81228600	-1.98523300
H	0.99825700	4.20875100	-1.04405700

IM0

C	-1.47486100	3.85033800	0.69740000
C	-2.72291400	3.05536000	0.33535400
H	-1.36281300	3.91187800	1.79816500
H	-1.54725500	4.87334500	0.30554900
H	-3.54420000	3.29179600	1.02893300
H	-3.05323300	3.32524100	-0.68782000
N	-2.33812900	1.65203200	0.42933300
N	-0.37762900	3.11890500	0.06759900
C	-3.29812100	0.60627100	-0.00086100
C	-4.68479700	0.92842100	0.57293300
H	-5.11386500	1.84338900	0.13802400
H	-5.37708600	0.10245200	0.35187800
H	-4.63059700	1.05157900	1.66545000
C	-3.36124700	0.53503000	-1.53852400
H	-2.36126900	0.27311300	-1.92485000
H	-4.07718800	-0.23008300	-1.87675900
H	-3.65934600	1.50004300	-1.97685300
C	-2.83253400	-0.74619900	0.55190000
H	-1.86075200	-1.01326100	0.11251600
H	-2.71345400	-0.70575800	1.64339900
H	-3.55327500	-1.53592800	0.29251600
C	0.98652900	3.70291900	0.03688100
C	1.66495200	3.62046500	1.41715400
H	1.78176600	2.57723200	1.73529000
H	2.65673000	4.09862700	1.39011600
H	1.06126100	4.13766300	2.17909500
C	0.89284300	5.17293200	-0.40159800
H	1.90380000	5.56623100	-0.58437300
H	0.31226000	5.26504200	-1.33262700
H	0.42925300	5.80857800	0.36725100
C	1.81515700	2.95075400	-1.01464800
H	1.37870600	3.09264400	-2.01545100
H	2.85121500	3.32036400	-1.02226200
H	1.83634700	1.86966500	-0.81529700
P	-0.65454800	1.44320300	0.23507400
O	-0.04547000	0.89498600	1.61737600
Al	0.95514700	-0.53091400	1.00427400
Ni	0.12030900	0.13898100	-1.14663300
C	0.30272100	-2.27137300	1.69753400
H	-0.52807200	-2.66182700	1.08387100
H	1.11737600	-3.01326100	1.59707000
C	2.91375600	-0.21117500	0.83485700
H	3.43783500	-0.69589400	1.68025000
H	3.13569900	0.86633000	0.92201000

C	3.46967900	-0.75066000	-0.49384000
H	2.95445900	-0.28941500	-1.35902500
H	4.55037600	-0.56647300	-0.63095100
H	3.31633300	-1.84028400	-0.58650800
C	-0.14512100	-2.17624100	3.16681200
H	-0.50981300	-3.13932800	3.56782600
H	0.67807400	-1.84793700	3.82534600
H	-0.95684100	-1.44042700	3.29273100

IM1

C	-3.31680100	5.82846600	-1.59383600
C	-4.73673000	5.27114000	-1.60385500
H	-3.26497200	6.71938800	-0.93862400
H	-3.00861900	6.13333000	-2.60394400
H	-5.46529700	6.09248500	-1.50772500
H	-4.94386900	4.75305900	-2.55935100
N	-4.81819700	4.34557100	-0.47912200
N	-2.47732700	4.73656500	-1.09639000
C	-6.10605500	4.12870400	0.20557600
C	-6.47606500	5.36779400	1.04364500
H	-6.55350400	6.26649300	0.41204800
H	-7.44579700	5.23002800	1.54792100
H	-5.70471100	5.54991600	1.80429000
C	-7.19053100	3.84756800	-0.84759800
H	-6.90594000	2.98221000	-1.46661200
H	-8.15005700	3.62539100	-0.35621700
H	-7.35194100	4.70789500	-1.51399700
C	-6.00918000	2.90971900	1.13268700
H	-5.76141100	2.00311600	0.56268600
H	-5.24833500	3.04184400	1.91275800
H	-6.97438900	2.75199600	1.63695300
C	-1.00887100	4.94033100	-1.07858700
C	-0.61455000	6.16733900	-0.23299400
H	-0.97749400	6.05629500	0.79765400
H	0.48017300	6.28375300	-0.21292100
H	-1.03803200	7.09450100	-0.64809000
C	-0.52497600	5.12151300	-2.52761100
H	0.57353700	5.18523900	-2.55647600
H	-0.84559000	4.26781300	-3.14422800
H	-0.91663000	6.04352200	-2.98197200
C	-0.31995400	3.69477100	-0.50531800
H	-0.59163400	2.79774000	-1.07820000
H	0.77162900	3.82558100	-0.55066700
H	-0.58605900	3.53008900	0.54824500

P	-3.30147400	3.95919700	0.19790500
O	-3.10921900	4.85231700	1.50967100
Al	-2.71499000	4.35967800	3.20861500
Ni	-2.93770300	1.81275400	0.39609300
C	-4.03316100	5.13179200	4.46433700
H	-5.01223900	4.64192100	4.29732500
H	-3.75620100	4.86638100	5.50280400
C	-0.76888100	4.53213700	3.57640500
H	-0.53105300	5.59742800	3.76200700
H	-0.18245400	4.26873400	2.67525300
C	-2.51261100	1.65850400	2.32578900
H	-1.43874900	1.83312200	2.05290200
O	-3.06962600	2.55051700	3.09899100
N	-2.77524000	0.31023400	2.60826000
C	-3.61391900	-0.11996200	3.77293800
C	-3.77617400	-1.64680000	3.74733900
C	-2.82336200	0.31227800	5.04822600
C	-5.02303400	0.48513600	3.63102100
H	-4.25600500	-1.98942900	2.81723900
H	-2.81930500	-2.17505100	3.87072800
H	-4.42353600	-1.95089000	4.57705700
H	-2.38518100	1.29295000	4.83411300
H	-1.97687300	-0.39236800	5.12195300
H	-5.69859800	0.07005100	4.39167600
H	-5.00788900	1.57353700	3.71765300
H	-5.43213300	0.22452900	2.64302600
C	-2.06404500	-0.59019300	1.71207400
H	-0.96884600	-0.52192400	1.86197600
H	-2.34542500	-1.62747200	1.91357700
C	-2.38832700	-0.23185100	0.27415300
C	-1.37086100	-0.00777800	-0.68776300
C	-3.74553700	-0.13712700	-0.14433100
C	-1.69021200	0.25753800	-2.01882200
H	-0.32553000	-0.05027400	-0.37222700
C	-4.05257100	0.13408100	-1.49506400
H	-4.54629700	-0.37089900	0.55974600
C	-3.03346600	0.32403100	-2.42682200
H	-0.89126700	0.43646500	-2.74132200
H	-5.09825500	0.19403800	-1.80363900
H	-3.27775500	0.55096300	-3.46629800
C	-4.19284400	6.65736700	4.35436200
H	-4.93823300	7.07302100	5.05715200
H	-3.24095100	7.17998300	4.55485100
H	-4.50465400	6.95855600	3.33939200

C	-0.28673300	3.68480700	4.76615600
H	-0.43003300	2.60549200	4.58087500
H	0.78335700	3.82568600	5.00578200
H	-0.85095000	3.91726100	5.68682300
C	-3.45391000	0.44318800	6.46209900
C	-4.38670300	1.66455800	6.56230400
C	-4.19769100	-0.81578900	6.93424700
C	-2.26598800	0.68558100	7.41668900
H	-3.90044500	2.56903800	6.16765600
H	-5.32463200	1.52600100	6.01053200
H	-4.65098900	1.85352300	7.61536400
H	-3.56343500	-1.71378900	6.85814000
H	-4.49439700	-0.70699900	7.99012200
H	-5.11863800	-0.99587700	6.35948900
H	-2.61749600	0.83915800	8.44952400
H	-1.57289800	-0.17147700	7.41868800
H	-1.69543700	1.57911500	7.11688700

IM2

C	-3.25279200	5.47546700	-1.77525800
C	-4.54504400	4.66228900	-1.79449700
H	-3.42155500	6.47488200	-1.32683300
H	-2.86748300	5.62129600	-2.79431100
H	-5.40909400	5.31534800	-1.98365500
H	-4.50457800	3.91063800	-2.60552200
N	-4.65743800	4.01213400	-0.48754200
N	-2.34967600	4.65380700	-0.98235300
C	-5.98214100	3.94843700	0.17166800
C	-6.43326700	5.35735700	0.60209500
H	-6.53362200	6.02999900	-0.26441400
H	-7.41188400	5.31969200	1.10638000
H	-5.69381700	5.78621000	1.29240500
C	-6.99933100	3.33401100	-0.80505000
H	-6.69630500	2.31470200	-1.08676200
H	-7.99104100	3.28002300	-0.33100600
H	-7.10553100	3.92731800	-1.72492500
C	-5.90803800	3.04608000	1.40796700
H	-5.48855400	2.06549100	1.15260900
H	-5.29496600	3.48723000	2.19915200
H	-6.91677300	2.89496300	1.81907200
C	-0.89467200	4.85330700	-0.99251600
C	-0.52083200	6.20022200	-0.34852700
H	-0.91626900	6.24843900	0.67652600
H	0.57142000	6.33833300	-0.31482300

H	-0.94919200	7.03815200	-0.92011100
C	-0.37577200	4.77759300	-2.43713600
H	0.72311700	4.83692900	-2.45119700
H	-0.68488200	3.82893800	-2.90234000
H	-0.75502000	5.60656600	-3.05225600
C	-0.24805500	3.70763200	-0.19297100
H	-0.61731600	2.72814100	-0.57098400
H	0.84290300	3.68376000	-0.31791800
H	-0.40851700	3.82200200	0.90036500
P	-3.16295600	3.93109700	0.33400400
O	-3.28943700	4.91355300	1.59341600
Al	-2.88745300	4.48589700	3.32720200
Ni	-1.77613300	2.35440000	1.03349600
C	-4.30751800	5.17566000	4.51693400
H	-5.25262700	4.63518100	4.31684300
H	-4.04747900	4.91468000	5.56129900
C	-0.97620200	4.81087400	3.75114900
H	-0.89092700	5.78097400	4.27778900
H	-0.39134700	4.94479600	2.82167900
C	-2.80446400	1.77414000	2.42780400
H	-0.74584900	1.57123600	1.79477100
O	-3.13628300	2.61713300	3.33176300
N	-3.22697900	0.49636300	2.54305800
C	-3.88660500	0.00558700	3.82191400
C	-4.24909900	-1.48105100	3.68932500
C	-2.82001200	0.18124700	4.94966800
C	-5.21230300	0.76519800	4.00219400
H	-4.93820500	-1.66061500	2.84984400
H	-3.36633000	-2.12521900	3.57229000
H	-4.76176100	-1.79765900	4.60510500
H	-2.24218400	1.08410300	4.72854900
H	-2.11801000	-0.66190400	4.83002500
H	-5.76700200	0.36316000	4.86045600
H	-5.06526100	1.83777200	4.14487700
H	-5.83322300	0.61537100	3.10659600
C	-2.69767200	-0.44745200	1.54385800
H	-1.64830400	-0.69093400	1.77998500
H	-3.27379500	-1.37453100	1.60804600
C	-2.78059300	0.05601800	0.11689400
C	-1.62748500	0.13567900	-0.68177500
C	-4.01469400	0.40465000	-0.45210900
C	-1.70312400	0.60286000	-1.99784600
H	-0.66416700	-0.15554300	-0.25907800
C	-4.08924100	0.88492000	-1.75894700

H	-4.92073300	0.32704000	0.15178600
C	-2.93146500	0.99657200	-2.53413000
H	-0.79455200	0.67050200	-2.60119700
H	-5.05408400	1.18189700	-2.17338100
H	-2.98842100	1.38181600	-3.55460600
C	-4.55061500	6.68984600	4.40930300
H	-5.32899200	7.05922300	5.10192800
H	-3.63366100	7.26599600	4.62591800
H	-4.86577000	6.97544300	3.39078100
C	-0.33084000	3.70339700	4.60173100
H	-0.32080900	2.74397700	4.05855700
H	0.71321000	3.92502800	4.88942200
H	-0.88806500	3.53200400	5.53998000
C	-3.17716100	0.28415900	6.45769100
C	-3.83434000	1.63622900	6.79392500
C	-4.05666600	-0.86666300	6.97159400
C	-1.82773500	0.22592100	7.20347500
H	-3.24363100	2.47167200	6.38865000
H	-4.85078900	1.72652200	6.39183600
H	-3.89992700	1.76467200	7.88645900
H	-3.61180500	-1.84835100	6.74152700
H	-4.16672600	-0.80023300	8.06614100
H	-5.07027200	-0.84146300	6.54457100
H	-1.97499900	0.33802300	8.28954900
H	-1.31428100	-0.73360800	7.02862200
H	-1.15731000	1.03321500	6.86807800

IM3

C	-3.62078300	6.09071200	-1.68380000
C	-4.95131600	5.35806200	-1.74712700
H	-3.71616000	6.98868000	-1.04310900
H	-3.31144800	6.42092200	-2.68554700
H	-5.78023000	6.08139800	-1.77973700
H	-5.00726900	4.74231700	-2.66777000
N	-5.00265500	4.54255700	-0.54291700
N	-2.67554100	5.11920000	-1.13709200
C	-6.19914200	3.72003100	-0.27386000
C	-7.46089800	4.57364000	-0.48382900
H	-7.60941300	4.84564300	-1.53936600
H	-8.34925600	4.00933100	-0.16213300
H	-7.40245300	5.49799800	0.11095200
C	-6.24587300	2.48725400	-1.19633400
H	-5.36499200	1.85163300	-1.02332500
H	-7.15201900	1.88911300	-1.01029700

H	-6.25200800	2.78406800	-2.25627100
C	-6.16947300	3.26078500	1.18629000
H	-5.30874000	2.60817200	1.36950400
H	-6.09911400	4.11694500	1.87133200
H	-7.07965500	2.69030000	1.42264700
C	-1.24306400	5.50637500	-1.08551700
C	-1.02955200	6.76060400	-0.21706400
H	-1.37082000	6.58279100	0.80997300
H	0.03719400	7.03145400	-0.18964700
H	-1.58129000	7.62442600	-0.61794800
C	-0.75772300	5.78064400	-2.52087600
H	0.32764900	5.96235800	-2.51987200
H	-0.96408000	4.91586300	-3.17023100
H	-1.23465300	6.66852400	-2.96125400
C	-0.40324400	4.34784500	-0.53236200
H	-0.46032600	3.47032300	-1.18831400
H	0.64767100	4.66359300	-0.45511800
H	-0.72597900	4.04146900	0.47207900
P	-3.45128400	4.21228600	0.11102700
O	-3.34220200	5.07510300	1.45223400
Al	-2.62351600	4.77403400	3.07295300
Ni	-2.64996700	2.17074300	0.31538700
C	-3.84836400	5.36879700	4.51010700
H	-3.64246700	4.78062600	5.42533300
H	-4.88985900	5.12233200	4.23153800
C	-0.72363600	5.29394000	3.27638900
H	-0.11278700	4.90514500	2.44171600
H	-0.62810200	6.39480800	3.21393600
C	-2.90266900	1.96470600	2.25558500
H	-1.93481000	1.54825600	1.53339500
O	-2.65288300	2.90802000	3.07188200
N	-3.63069500	0.89584300	2.69637700
C	-4.18729100	0.78660500	4.10627900
C	-3.08287100	1.13086200	5.12666600
C	-5.40379700	1.74934000	4.14024900
C	-4.59733500	-0.67383100	4.36095100
H	-2.12781800	0.67883800	4.82070500
H	-2.92803400	2.20623700	5.23845800
H	-3.34411000	0.71215800	6.10642600
H	-6.03988600	1.45853100	3.28973500
H	-5.00765900	2.74313600	3.89612900
H	-4.85409700	-0.78717800	5.41997200
H	-5.47503800	-0.98425400	3.77949400
H	-3.77243200	-1.36558000	4.14828000

C	-3.99683300	-0.08984200	1.68574200
H	-5.03272100	-0.41352800	1.85338900
H	-4.01174400	0.42342800	0.71005300
C	-3.08185400	-1.29158900	1.57611900
C	-3.43069800	-2.33094500	0.70245700
C	-1.87375900	-1.37665700	2.27567900
C	-2.58873000	-3.43051300	0.53074000
H	-4.36858100	-2.27171900	0.14279200
C	-1.03159400	-2.47984000	2.11252700
H	-1.59084300	-0.57256600	2.95563100
C	-1.38356600	-3.50919700	1.23696300
H	-2.87293900	-4.23015100	-0.15743800
H	-0.09266200	-2.52928300	2.66845000
H	-0.72344600	-4.36926700	1.10410500
C	-1.77876500	0.92760900	-0.90811600
C	-2.52534400	1.71101000	-1.56919300
C	-2.97857700	2.27813700	-2.85777700
H	-4.01513800	1.95755900	-3.06063000
H	-3.01301100	3.37418400	-2.75771600
C	-0.72742000	-0.11503300	-0.83051800
H	-1.16257000	-1.05266200	-0.45006400
H	-0.37585300	-0.32990300	-1.85635700
C	-2.07015300	1.89152600	-4.03196300
C	-2.52152500	2.51940500	-5.34874300
H	-2.03784800	0.79225500	-4.12478200
H	-1.03875400	2.20764300	-3.79796400
H	-1.85865700	2.23821300	-6.18215600
H	-3.54426800	2.20237300	-5.61290800
H	-2.52606000	3.62023500	-5.28111700
C	0.46969300	0.27126000	0.04667900
C	1.57039900	-0.78586400	0.03925700
H	0.11149400	0.43644600	1.07668700
H	0.87053100	1.24113900	-0.29402400
H	2.40705200	-0.50126400	0.69652100
H	1.18487500	-1.75875200	0.38375600
H	1.97795200	-0.93293800	-0.97475800
C	-6.35500600	1.92979300	5.35630300
C	-7.22376200	0.68975300	5.63485200
C	-5.62207200	2.34167300	6.64277400
C	-7.30229900	3.08165700	4.95655500
H	-7.70280500	0.32052900	4.71341500
H	-6.65342000	-0.13856400	6.07577300
H	-8.02514000	0.94345500	6.34733700
H	-4.95750400	3.20002000	6.46552600

H	-6.35203500	2.63459600	7.41460100
H	-5.02087200	1.52117300	7.05854300
H	-8.03644200	3.27737200	5.75414400
H	-6.74110500	4.01090200	4.77589900
H	-7.85894000	2.83906400	4.03700900
C	-3.74254300	6.86859400	4.83103200
H	-4.41676100	7.19221000	5.64509800
H	-2.71805400	7.14504400	5.13526800
H	-3.98574900	7.48851600	3.95038300
C	-0.13944300	4.80367200	4.61295000
H	-0.70422100	5.20299700	5.47373600
H	-0.18444800	3.70291400	4.69263100
H	0.91693000	5.09221600	4.76376100

IM4

C	-3.98379300	6.03432900	-2.01095500
C	-5.28664200	5.24338900	-2.05906000
H	-4.09441000	6.89921700	-1.32932700
H	-3.72530800	6.42047600	-3.00747100
H	-6.14262000	5.92606000	-1.94713700
H	-5.39809700	4.72784500	-3.03003900
N	-5.23042200	4.28165500	-0.96084200
N	-2.96755000	5.09228900	-1.54003800
C	-6.46892100	3.92917700	-0.23272600
C	-6.94067900	5.12756000	0.61327600
H	-7.15613400	6.00298500	-0.01885900
H	-7.86150100	4.88189900	1.16541200
H	-6.15887200	5.40652900	1.33315100
C	-7.54886000	3.52724300	-1.25075500
H	-7.21204400	2.67000900	-1.85343100
H	-8.47389500	3.24136800	-0.72728200
H	-7.79920100	4.35055800	-1.93574500
C	-6.21613700	2.73465200	0.69548400
H	-5.83244300	1.87085700	0.13443900
H	-5.50027300	2.98131600	1.48801700
H	-7.15777900	2.44449600	1.18432100
C	-1.56283100	5.57487100	-1.45185600
C	-1.45331400	6.81440000	-0.54308300
H	-1.82322800	6.58599100	0.46470100
H	-0.40428100	7.13834400	-0.46621200
H	-2.03190000	7.66176400	-0.94078100
C	-1.07769200	5.92119500	-2.86975200
H	-0.01949700	6.22234500	-2.84156000
H	-1.17371700	5.04979100	-3.53490900

H	-1.64259300	6.75716800	-3.30820100
C	-0.65413900	4.46724900	-0.90113400
H	-0.65941700	3.58264700	-1.55019900
H	0.37582900	4.84809400	-0.83628500
H	-0.94789700	4.15726200	0.11139800
P	-3.66024400	4.08497400	-0.33941100
O	-3.58498000	4.86280300	1.04437700
Al	-2.96788800	4.51713900	2.70987600
Ni	-2.91322600	2.10644500	-0.18943000
C	-4.39486000	4.99238100	3.99958900
H	-4.15302200	4.57995300	4.99792300
H	-5.33095300	4.48600600	3.69632800
C	-1.13959600	5.19808800	3.03428000
H	-1.13769000	6.30296600	2.96396000
H	-0.45938400	4.85928700	2.23068700
C	-3.11273000	1.79695100	1.74388300
H	-1.64615100	0.69531800	-0.53367100
O	-2.89370400	2.67350900	2.65190900
N	-3.52919300	0.58177100	2.18820100
C	-3.84561300	0.27610400	3.64537900
C	-2.65040700	0.68244300	4.53137300
C	-5.15821800	1.06175900	3.92007000
C	-4.02307100	-1.24054900	3.82160800
H	-1.71078300	0.38920700	4.03982300
H	-2.60962700	1.75594400	4.72723400
H	-2.70139500	0.14937600	5.49022200
H	-5.87466400	0.73845500	3.14483700
H	-4.92917900	2.11149200	3.70092900
H	-4.15170800	-1.45802900	4.88754900
H	-4.90037700	-1.64164800	3.29750600
H	-3.13356800	-1.78573800	3.48127000
C	-3.95601400	-0.36745600	1.16769200
H	-4.97443500	-0.72223700	1.38503500
H	-4.04412000	0.19973200	0.22295700
C	-3.03584000	-1.54189700	0.92600800
C	-3.55991700	-2.75544800	0.46266300
C	-1.64927000	-1.42285100	1.08736000
C	-2.71756900	-3.82560500	0.15305800
H	-4.64156300	-2.86212200	0.34106300
C	-0.80344000	-2.48944400	0.77381600
H	-1.23462500	-0.49026500	1.47452700
C	-1.33412400	-3.69364500	0.30231700
H	-3.14240700	-4.76536600	-0.20751100
H	0.27615500	-2.38023300	0.90199000

H	-0.67282400	-4.52803700	0.05817300
C	-2.59463000	1.84831200	-2.00830100
C	-1.81371000	0.82943900	-1.64974000
C	-1.16153200	-0.26497900	-2.45770900
H	-0.82338100	0.16072000	-3.41869700
H	-0.26012200	-0.62615800	-1.93533200
C	-3.03334200	2.29652100	-3.35475800
H	-2.91793000	3.39105300	-3.41334000
H	-2.39884700	1.85311600	-4.14606000
C	-4.64767100	6.50469700	4.11258200
H	-4.93404800	6.93881700	3.13839200
H	-5.44989100	6.76533700	4.82707900
H	-3.74129200	7.04316100	4.44018900
C	-0.57775500	4.76921000	4.40084300
H	0.43797200	5.15726800	4.59999800
H	-1.21933900	5.11625600	5.22983700
H	-0.52142800	3.66962000	4.48751200
C	-5.92994600	1.05599400	5.26792000
C	-7.08431300	2.06283600	5.07346600
H	-7.70845400	2.12218300	5.97933400
H	-6.69810700	3.07104100	4.85957500
H	-7.73485600	1.76427800	4.23487500
C	-6.55263500	-0.30865500	5.61133600
H	-7.10303800	-0.72577700	4.75234600
H	-5.80478100	-1.04886900	5.92576900
H	-7.26672900	-0.19982800	6.44363200
C	-5.07029500	1.54100600	6.44627600
H	-4.29478500	0.81304200	6.72077600
H	-4.57551900	2.49560200	6.21152600
H	-5.70195600	1.69866000	7.33548300
C	-4.50638900	1.94534300	-3.62076400
C	-5.00054300	2.43826700	-4.97808200
H	-5.11273800	2.37936500	-2.81048900
H	-4.63336800	0.85124900	-3.54675800
H	-6.06383200	2.19622300	-5.13457700
H	-4.89003300	3.53197200	-5.06711900
H	-4.42858100	1.98398100	-5.80430900
C	-2.09216900	-1.45636000	-2.72900600
C	-1.39406900	-2.60369500	-3.45400300
H	-2.95692800	-1.10182300	-3.31625300
H	-2.49771100	-1.81708900	-1.77310600
H	-2.08512000	-3.44138700	-3.63875600
H	-0.98832900	-2.28139300	-4.42768000
H	-0.55426600	-2.99278800	-2.85510500

IM4'

C	-2.95706500	5.27207700	-1.61162800
C	-4.33348700	4.65528700	-1.85755900
H	-3.07244500	6.30922500	-1.24209500
H	-2.37843000	5.30807800	-2.54680800
H	-5.03230000	5.43205100	-2.20390400
H	-4.27745700	3.87355900	-2.63786700
N	-4.76061300	4.10010100	-0.58397700
N	-2.29697600	4.41744700	-0.62512500
C	-6.18281500	4.05121000	-0.19031900
C	-6.60936000	5.43582300	0.33220900
H	-6.45856100	6.20525700	-0.44154200
H	-7.67373700	5.44751900	0.61685300
H	-6.00404800	5.70696000	1.20788800
C	-7.04411700	3.65113800	-1.40022100
H	-6.72217100	2.68199400	-1.80767400
H	-8.09860400	3.56806400	-1.09660800
H	-6.99218800	4.39649100	-2.20679700
C	-6.39319000	3.00306300	0.91425100
H	-6.09704100	2.00117600	0.57186900
H	-5.82204600	3.24445300	1.81883400
H	-7.45630700	2.97078600	1.19490700
C	-0.95391600	4.81588500	-0.12834100
C	-0.99499900	6.18203200	0.58506100
H	-1.68119600	6.15428400	1.44000000
H	0.00869800	6.45047400	0.94900000
H	-1.32248500	6.98073200	-0.09768000
C	0.01261500	4.89562100	-1.32216200
H	1.03115900	5.11102000	-0.96559000
H	0.03353600	3.94628100	-1.87759500
H	-0.26562800	5.69850100	-2.02060500
C	-0.43207200	3.75066600	0.84789700
H	-0.36371400	2.76790400	0.36525200
H	0.57182000	4.03414500	1.19630700
H	-1.06901300	3.65924500	1.73759700
P	-3.45526000	3.71423300	0.41803000
O	-3.58815200	4.63511000	1.70126200
Al	-3.53067200	4.47654700	3.49487000
Ni	-3.24532600	1.63371000	0.86155500
C	-5.27117500	4.98856000	4.29590600
H	-5.31223500	4.60005800	5.33152100
H	-6.10604200	4.48754700	3.76934300
C	-1.90207700	5.27731500	4.28118000

H	-1.87331700	6.35703300	4.03778400
H	-0.99994900	4.85319300	3.80195600
C	-3.44795300	1.62894000	2.83642200
H	-5.13986500	1.28938200	-1.26741100
O	-3.37867300	2.64561400	3.61536700
N	-3.61633900	0.44773900	3.48662200
C	-3.89107300	-0.80396600	2.71827600
C	-5.43913900	-0.92226700	2.58648800
C	-3.19962200	-0.67079600	1.36470200
C	-3.26985300	-1.99933100	3.45697000
H	-5.82066600	-1.13165200	3.59450100
H	-5.79914000	0.08978800	2.34400300
H	-2.21716800	-1.79278500	3.70449700
H	-3.80963100	-2.24305900	4.38168800
C	-3.71596000	0.43011500	4.95667200
H	-3.15451100	-0.42589900	5.34721000
H	-3.21401000	1.34117100	5.30249400
C	-5.13463100	0.40105000	5.48266900
C	-5.56518000	-0.63053800	6.32547200
C	-6.04757900	1.40210700	5.11644900
C	-6.88576100	-0.67550200	6.78450600
H	-4.86139500	-1.41367100	6.62190500
C	-7.36473200	1.35952700	5.57224400
H	-5.72029900	2.21616600	4.47056400
C	-7.79086200	0.31737700	6.40370700
H	-7.20716600	-1.48996700	7.43836700
H	-8.06182900	2.14843100	5.27962900
H	-8.82407300	0.28378300	6.75748800
C	-4.17444200	1.15542300	-1.77168300
C	-3.05802200	1.37016100	-1.05236400
H	-3.12801900	-1.62989900	0.83616300
H	-3.80465300	-0.06671900	0.64843600
H	-2.17350000	-0.28924200	1.47852500
C	-4.32421200	0.73631100	-3.21218700
C	-4.98582800	-0.64099500	-3.36906400
H	-3.35325500	0.73289300	-3.73185900
H	-4.94987200	1.48243300	-3.74043300
C	-5.21278100	-1.04095500	-4.82553700
H	-4.35658600	-1.39495200	-2.86399700
H	-5.94804300	-0.64347300	-2.82682100
H	-5.68396200	-2.03349000	-4.90754600
H	-5.86687800	-0.31701600	-5.33984700
H	-4.26142000	-1.07551800	-5.38229000
C	-1.67042900	1.27502800	-1.63480300

C	-0.71080900	0.33027500	-0.90035000
H	-1.24183100	2.28990800	-1.62243900
H	-1.70237300	0.97488100	-2.69685800
C	0.68722800	0.31384300	-1.51423100
H	-0.63595800	0.62959900	0.16022100
H	-1.13284400	-0.69013000	-0.89767200
H	1.36315000	-0.37106500	-0.97795900
H	0.65495700	-0.00421600	-2.56949100
H	1.14014500	1.31903200	-1.48917900
C	-5.51718500	6.50687800	4.31146500
H	-6.46945900	6.79218800	4.79487300
H	-4.71399500	7.04023700	4.84916200
H	-5.54138400	6.92645100	3.29027400
C	-1.81465600	5.09405800	5.80577500
H	-0.91074900	5.54773200	6.25199300
H	-2.68344700	5.54295500	6.31811100
H	-1.80541600	4.02544000	6.08538500
H	-3.29541500	-2.89063300	2.82066200
C	-6.16065000	-1.89630400	1.61360300
C	-6.07399500	-1.43751900	0.14673300
H	-6.37662500	-0.38418900	0.04222400
H	-5.06927700	-1.53449100	-0.28240200
H	-6.74938100	-2.04023500	-0.48123200
C	-7.64625000	-1.84974900	2.02873100
H	-8.25962100	-2.47770200	1.36312600
H	-7.78323100	-2.20868400	3.06118100
H	-8.03719200	-0.82087600	1.98031900
C	-5.67694700	-3.35046100	1.72411000
H	-4.65870900	-3.48241700	1.32816600
H	-5.68783500	-3.70291700	2.76749500
H	-6.33644100	-4.01309600	1.14096800

IM5

C	-2.32714700	5.06312100	-2.22996300
C	-3.76814700	4.83007400	-2.67773700
H	-2.24446700	6.03806200	-1.71312800
H	-1.64778000	5.08079300	-3.09522900
H	-4.23594500	5.78945600	-2.94265400
H	-3.79770300	4.19030300	-3.57875600
N	-4.46616500	4.18863000	-1.56149900
N	-2.00843900	3.94719200	-1.33946400
C	-5.83900500	4.63707200	-1.21735600
C	-5.82572800	6.09456800	-0.71543300
H	-5.44564200	6.78089500	-1.48819700

H	-6.84261000	6.42531500	-0.45255200
H	-5.18484700	6.18134200	0.17061600
C	-6.73052900	4.52403700	-2.46499100
H	-6.76729900	3.48844800	-2.83135800
H	-7.75663700	4.84078900	-2.22419800
H	-6.37221600	5.16502200	-3.28417700
C	-6.42077000	3.73473900	-0.11780800
H	-6.42861400	2.68180900	-0.42671500
H	-5.85848200	3.82031300	0.82012800
H	-7.45629000	4.03841400	0.09501300
C	-0.62898400	3.84757100	-0.79788500
C	-0.22705100	5.13513400	-0.05431000
H	-0.94998800	5.36162400	0.73937400
H	0.76921300	5.02023700	0.39943200
H	-0.18159200	5.99622000	-0.73814700
C	0.34624300	3.59349000	-1.96073200
H	1.37445600	3.50892400	-1.57754100
H	0.09836500	2.65935400	-2.48663100
H	0.33670000	4.41493900	-2.69209900
C	-0.54165300	2.65604600	0.16488900
H	-0.84387000	1.71932600	-0.32395000
H	0.49591400	2.53678200	0.51010800
H	-1.16727100	2.80292000	1.05584800
P	-3.38454200	3.58455500	-0.37454200
O	-3.38574600	4.64160600	0.82020200
Al	-3.49514400	4.65525300	2.61857500
Ni	-3.67944400	1.45241000	0.29276200
C	-5.13235100	5.54582900	3.28303700
H	-5.27965500	5.21906400	4.33164100
H	-6.02190000	5.17067800	2.74284800
C	-1.77283800	5.12111700	3.47714400
H	-1.45621500	6.13448800	3.16376800
H	-0.97986800	4.44569400	3.10271300
C	-3.72165000	1.81612400	2.14395700
H	-2.39342400	-0.15888100	-1.40596000
O	-3.72566800	2.83630700	2.90343300
N	-3.77907400	0.58266100	2.61950800
C	-3.91588300	0.14896900	4.04826900
C	-2.68571500	0.66257800	4.81151300
C	-5.28508400	0.72825300	4.51918400
C	-3.92083800	-1.38349600	4.06887900
H	-1.77392100	0.26729700	4.34012100
H	-2.63435000	1.75743700	4.80500600
H	-2.70016000	0.31355600	5.85285400

H	-6.05385300	0.09841500	4.03953100
H	-5.38767700	1.72429000	4.07032000
H	-4.00121800	-1.74016800	5.10253300
H	-4.77065600	-1.79298700	3.50402900
H	-2.99533500	-1.78919700	3.63816700
C	-3.80517400	-0.22273700	1.39505500
H	-4.74816900	-0.77697700	1.28658000
H	-4.66201100	-0.67755800	-0.92346400
C	-2.60924300	-1.04067900	1.10134800
C	-2.72477800	-2.23000500	0.35638000
C	-1.31175100	-0.60720400	1.44546600
C	-1.59513300	-2.93798200	-0.05801200
H	-3.71986500	-2.60419100	0.10077000
C	-0.18423400	-1.31979900	1.04129300
H	-1.19352100	0.31134800	2.02245900
C	-0.31582600	-2.48570700	0.27749000
H	-1.71578400	-3.85514200	-0.64031200
H	0.80917600	-0.95793100	1.31877800
H	0.56909200	-3.03949000	-0.04393300
C	-4.46440400	0.18109400	-1.56671900
C	-3.16738500	0.46035200	-1.87377800
C	-2.70958300	1.33520200	-3.00165300
H	-3.56247200	1.87030500	-3.44466000
H	-2.04020600	2.11646600	-2.61906400
C	-5.65698800	0.78731300	-2.25086200
H	-6.51633000	0.78116400	-1.56010600
H	-5.45142400	1.84174000	-2.48246700
C	-5.10179200	7.08201900	3.23000300
H	-5.01455600	7.45174500	2.19319500
H	-6.00404300	7.55431500	3.65989900
H	-4.23551700	7.48858900	3.78058100
C	-1.82691100	5.05362500	5.01314700
H	-0.86611100	5.30525600	5.49832600
H	-2.58247300	5.74645800	5.42235800
H	-2.10711300	4.04559400	5.36667900
C	-5.67306600	0.90194700	6.01280200
C	-7.17588100	1.24926800	6.01497300
H	-7.53278500	1.43975700	7.03976000
H	-7.37287800	2.15262700	5.41563100
H	-7.77674600	0.42668800	5.59432800
C	-5.47253600	-0.36697200	6.85579100
H	-5.99989900	-1.22922900	6.41696500
H	-4.41043500	-0.63358900	6.96062200
H	-5.87104700	-0.21514400	7.87217500

C	-4.92388500	2.08312600	6.66013700
H	-3.85592300	1.87918500	6.80634800
H	-5.00885700	2.99019300	6.04109000
H	-5.35338100	2.30785500	7.64984500
C	-6.04726700	0.03350800	-3.53371000
C	-7.26357000	0.64232600	-4.22814600
H	-6.24241800	-1.02527600	-3.28908300
H	-5.18461200	0.02952700	-4.22161300
H	-7.53457300	0.08003300	-5.13545700
H	-8.14262700	0.64906700	-3.56268900
H	-7.06962300	1.68550000	-4.52710000
C	-1.98408700	0.52859700	-4.08888900
C	-1.50686900	1.41382100	-5.23861200
H	-2.65041000	-0.26357400	-4.47330100
H	-1.12274700	0.00498200	-3.63722100
H	-0.98348600	0.82957200	-6.01157900
H	-2.35440900	1.92703000	-5.72273700
H	-0.81418700	2.19211500	-4.87848800

IM6

C	-2.84322600	5.47680900	-1.60280100
C	-4.20102100	5.07929100	-2.16473500
H	-2.93633200	6.41806900	-1.02856200
H	-2.11379400	5.63973000	-2.40943100
H	-4.76099100	5.97227600	-2.48326100
H	-4.07815900	4.43057500	-3.05433100
N	-4.87892500	4.38927700	-1.07898400
N	-2.44133300	4.35330800	-0.75365300
C	-6.34881600	4.27567600	-1.07164100
C	-6.97224700	5.67784300	-0.93760900
H	-6.72859500	6.32027900	-1.79733000
H	-8.06974600	5.60965400	-0.87835700
H	-6.59967400	6.16320500	-0.02372500
C	-6.81827800	3.60533500	-2.37503200
H	-6.33855900	2.62264300	-2.48997600
H	-7.91141000	3.47280000	-2.37503500
H	-6.55914100	4.21339000	-3.25545200
C	-6.80552900	3.43921300	0.13205900
H	-6.32720700	2.45081700	0.13775900
H	-6.56299100	3.93731000	1.07826100
H	-7.89475800	3.29371200	0.09146200
C	-1.10251900	4.40810500	-0.11166100
C	-0.94626800	5.67354200	0.75216500
H	-1.74949200	5.73067900	1.49778000

H	0.02070900	5.66094600	1.27820500
H	-0.97643400	6.58667500	0.13836000
C	-0.02757000	4.38965200	-1.21061700
H	0.97482900	4.39971800	-0.75654200
H	-0.11812100	3.48219400	-1.82476500
H	-0.09457500	5.26536400	-1.87278500
C	-0.90174400	3.16418700	0.76352800
H	-1.08283500	2.24339000	0.19149900
H	0.13445200	3.13727100	1.13187400
H	-1.55986500	3.16530400	1.64209400
P	-3.81868300	3.73306200	0.08167100
O	-4.02237800	4.57692700	1.41515000
Al	-4.16905700	4.24259200	3.18385700
Ni	-3.83234900	1.49833800	0.40064500
C	-6.03537800	4.44863300	3.81627200
H	-6.05057300	4.25516700	4.90660600
H	-6.65806100	3.64314900	3.38055100
C	-2.69707700	5.08823100	4.20097700
H	-2.75171500	6.18780700	4.08321600
H	-1.73008500	4.80096000	3.74603000
C	-3.70877100	1.54686000	2.28964200
O	-3.81556400	2.41248600	3.21563300
N	-3.53481300	0.25454700	2.52898700
C	-3.59128200	-0.47306200	3.83878900
C	-2.54987500	0.16044400	4.77680100
C	-5.06117300	-0.27930700	4.31325200
C	-3.21605000	-1.93494700	3.58285900
H	-1.56968800	0.18205000	4.27717700
H	-2.82150500	1.18919300	5.04175900
H	-2.44307300	-0.42678700	5.69690300
H	-5.69686100	-0.51178500	3.44130200
H	-5.17662500	0.80142300	4.48293700
H	-3.17545300	-2.47581200	4.53678800
H	-3.94069700	-2.44352200	2.93225800
H	-2.22608600	-2.00537500	3.11315200
C	-3.59553500	-0.32087800	1.17928000
H	-4.46485600	-0.98055300	1.05034500
C	-2.34370600	-0.90767200	0.65576000
C	-2.38601300	-1.90809900	-0.33295300
C	-1.07650700	-0.45062700	1.07080000
C	-1.21680000	-2.42969100	-0.88587700
H	-3.35616000	-2.28324400	-0.66609900
C	0.09340200	-0.96949000	0.51608400
H	-1.01636400	0.32017400	1.84047800

C	0.03299100	-1.96079900	-0.46945800
H	-1.28105800	-3.20976300	-1.64895700
H	1.06257000	-0.59593400	0.85672100
H	0.94951100	-2.36731400	-0.90288300
C	-3.84510300	1.14591900	-1.76795300
C	-4.85783000	0.66657400	-1.25533400
C	-2.75276500	1.59303000	-2.64060900
C	-1.53950400	0.64959900	-2.63125700
H	-2.44218700	2.60381200	-2.33263900
H	-3.15579700	1.67620300	-3.66669500
C	-0.47327900	1.06304800	-3.64086300
H	-1.11377200	0.62131100	-1.61630200
H	-1.87599600	-0.37722700	-2.83826300
H	0.39191000	0.38352800	-3.60007400
H	-0.86594500	1.04653800	-4.67129400
H	-0.10566300	2.08352600	-3.44652500
C	-6.14183800	-0.01134900	-1.01243600
C	-6.24221800	-1.38673600	-1.69072800
H	-6.95052200	0.64292200	-1.38258000
H	-6.31740000	-0.11334700	0.06996500
C	-7.59698700	-2.04754400	-1.45132700
H	-6.05778700	-1.26614400	-2.77119300
H	-5.43231200	-2.03126000	-1.31272900
H	-7.65504900	-3.03183200	-1.94082700
H	-7.78265500	-2.19689700	-0.37484600
H	-8.41935000	-1.42793000	-1.84538400
C	-5.69143900	-1.01382700	5.52784600
C	-5.99539800	-2.49231600	5.22355100
C	-4.84244800	-0.91861500	6.80517700
C	-7.03526400	-0.30268500	5.78973100
H	-6.58326900	-2.59192800	4.29643800
H	-5.08633900	-3.09747700	5.11142400
H	-6.58539800	-2.93630400	6.04175900
H	-4.56381900	0.12316500	7.02719700
H	-5.41037100	-1.30601600	7.66655900
H	-3.91825000	-1.51057500	6.73292500
H	-7.58284000	-0.78778600	6.61364500
H	-6.87973700	0.75401500	6.05892400
H	-7.67920500	-0.32817400	4.89553000
C	-6.67948400	5.81485600	3.53121200
H	-6.73726400	6.01812200	2.44749500
H	-7.70696800	5.90837000	3.92851200
H	-6.09223500	6.64009100	3.97055900
C	-2.68507600	4.73077500	5.69675600

H	-2.58648500	3.64160200	5.85175300
H	-1.85833900	5.20701200	6.25516300
H	-3.62240800	5.03420600	6.19458500

IM7

C	-1.19517300	6.00140000	0.60493900
C	-2.20904300	6.19091900	-0.51057600
H	-1.29795500	6.81610600	1.34667500
H	-0.16899700	6.02840700	0.21151800
H	-2.29014300	7.25453500	-0.78492100
H	-1.88252200	5.63169800	-1.41133100
N	-3.46241900	5.67923400	0.02798000
N	-1.50247100	4.68956700	1.17785500
C	-4.61875700	5.51355300	-0.87469100
C	-4.76294400	6.75984500	-1.75983200
H	-3.91883700	6.87492900	-2.45547600
H	-5.68210100	6.68872000	-2.36007600
H	-4.82610500	7.66305800	-1.13490400
C	-4.43801200	4.27741200	-1.77516900
H	-4.36872000	3.32329800	-1.20257500
H	-5.28129700	4.14842400	-2.46785800
H	-3.51004100	4.33429000	-2.36288500
C	-5.88947700	5.36652700	-0.02764400
H	-5.85479600	4.46099000	0.60057200
H	-6.00602600	6.22481200	0.64724200
H	-6.77849600	5.28490800	-0.67058700
C	-0.58747000	4.16205100	2.22327800
C	-0.44519500	5.16236600	3.38559400
H	-1.43427300	5.40185900	3.80168500
H	0.17867900	4.73331700	4.18472900
H	0.03299800	6.09917000	3.06112500
C	0.78089000	3.88530400	1.57916100
H	1.47041900	3.46098100	2.32474500
H	0.67360000	3.16454900	0.75481600
H	1.24820600	4.80064400	1.18650400
C	-1.13198600	2.83927200	2.77214300
H	-1.30306600	2.10867300	1.97167900
H	-0.40161200	2.41047800	3.47379500
H	-2.06657000	2.98467400	3.32621300
P	-3.20018600	4.47146800	1.23220500
O	-3.75764900	5.02550800	2.61554900
Al	-4.85936800	4.17634800	3.78702000
Ni	-4.32848400	2.64199700	0.62152900
C	-6.77389100	4.57435600	3.49445500

H	-7.08563400	4.20747700	2.49845600
H	-7.35897700	3.96683600	4.21224700
C	-4.08283800	4.37035500	5.59439400
H	-3.93688400	5.45510100	5.76450100
H	-3.05922600	3.94854600	5.60046900
C	-4.28070800	1.75267000	2.22673600
O	-4.62095700	2.38531600	3.27915900
N	-3.86167700	0.47901500	2.32994200
C	-3.86644000	-0.26845300	3.66153700
C	-3.16924700	0.56416700	4.75123100
C	-5.36911700	-0.57198500	3.91712100
C	-3.01763700	-1.54742500	3.52086400
H	-2.18448200	0.88931800	4.38897500
H	-3.73319200	1.44569400	5.05654900
H	-3.00261500	-0.06863900	5.63302300
H	-5.68269900	-1.23801000	3.09443200
H	-5.90880300	0.37238700	3.76044000
H	-2.80126800	-1.95114900	4.51566800
H	-3.51495900	-2.34716500	2.95656900
H	-2.05639300	-1.31923600	3.04035300
C	-3.81683600	-0.32808000	1.08198300
H	-3.93032800	-1.36440700	1.40092000
C	-2.46333500	-0.26868100	0.36813200
C	-1.67490400	-1.42483100	0.27841300
C	-1.97796200	0.90406900	-0.22374400
C	-0.43772000	-1.40878100	-0.37268200
H	-2.03329900	-2.35593700	0.72437300
C	-0.73975100	0.93271400	-0.86430700
H	-2.57533200	1.82027100	-0.17268900
C	0.03822400	-0.22667800	-0.94347600
H	0.15617500	-2.32422100	-0.43030300
H	-0.38554700	1.86790000	-1.30475200
H	1.00657400	-0.20885000	-1.44910700
C	-5.42398700	1.22743500	0.01622000
C	-5.01369500	-0.04166100	0.17969700
C	-7.15102000	6.05578100	3.65068800
H	-8.22841500	6.25441700	3.50282000
H	-6.89209000	6.43342300	4.65542600
H	-6.60513700	6.69110900	2.93145900
C	-4.89996800	3.77766800	6.75273300
H	-5.01932700	2.68599000	6.65069400
H	-4.44674000	3.95548900	7.74513500
H	-5.91949400	4.19932200	6.78708500
C	-6.65629600	1.61948400	-0.77522700

C	-6.52625200	1.44774600	-2.29900600
H	-6.91564800	2.67086800	-0.56708700
H	-7.52896600	1.03742000	-0.42349900
C	-7.65330500	2.13081900	-3.07068000
H	-5.55121600	1.84149600	-2.62921300
H	-6.50459100	0.37517900	-2.54711300
H	-7.55821500	1.97408600	-4.15695900
H	-8.63890600	1.74499300	-2.76168400
H	-7.65974700	3.21901600	-2.88815500
C	-5.64996500	-1.26292100	-0.44382700
C	-4.86261000	-1.86700400	-1.61949500
H	-6.66676100	-1.01668000	-0.78806300
H	-5.77542500	-2.04482700	0.33022300
C	-5.57277700	-3.05991400	-2.25479200
H	-4.69325300	-1.07970000	-2.37324000
H	-3.85883400	-2.16764800	-1.27849100
H	-4.99208200	-3.47987300	-3.09126300
H	-5.73309900	-3.86703800	-1.52028800
H	-6.56327500	-2.77232000	-2.64576000
C	-5.92279800	-1.17426100	5.23710800
C	-5.21410900	-2.46553700	5.67539000
C	-5.89019600	-0.15437000	6.38922000
C	-7.40033400	-1.51089900	4.94820000
H	-5.16852400	-3.20143500	4.85687200
H	-4.18846600	-2.27676900	6.02332200
H	-5.75912600	-2.93138300	6.51236300
H	-6.36092500	0.79496000	6.09063400
H	-6.44306600	-0.54481400	7.25897200
H	-4.86941000	0.06993600	6.72383100
H	-7.89863500	-1.89637900	5.85187300
H	-7.95151000	-0.61758600	4.61343300
H	-7.48888100	-2.27589700	4.15974700

IM8

C	-1.11905700	5.58742100	0.17506300
C	-1.96511300	5.53585500	-1.08095300
H	-1.30329500	6.54214200	0.70404300
H	-0.04872800	5.54178900	-0.07603900
H	-1.98454800	6.52166100	-1.56787500
H	-1.53203200	4.81380400	-1.80595000
N	-3.28843200	5.14943900	-0.62087800
N	-1.52209000	4.43027400	0.96960000
C	-4.39023400	5.08537900	-1.60425300
C	-4.39266100	6.36224100	-2.45902100

H	-3.52458200	6.41756900	-3.13240000
H	-5.29648500	6.38268200	-3.08635800
H	-4.39707800	7.25573700	-1.81614200
C	-4.23930800	3.85191500	-2.51288600
H	-4.23404800	2.94130300	-1.89120100
H	-5.06685700	3.77177400	-3.23487300
H	-3.29548200	3.88356200	-3.07867700
C	-5.71278200	5.02441600	-0.83169600
H	-5.75914900	4.12674800	-0.20727600
H	-5.80893600	5.89415200	-0.16746500
H	-6.56448800	5.00106600	-1.52707500
C	-0.90340000	4.31289800	2.31459000
C	-1.30945800	5.48893700	3.22676100
H	-2.39931500	5.52835800	3.33985000
H	-0.85435000	5.37536600	4.22279600
H	-0.96840400	6.45158600	2.81756600
C	0.62744200	4.28697400	2.16057100
H	1.09638400	4.06181100	3.13056200
H	0.92761800	3.51009500	1.44001200
H	1.02903700	5.25242100	1.81996100
C	-1.32546400	2.99585700	2.96915600
H	-0.90968000	2.13485700	2.42999200
H	-0.95261100	2.95969900	4.00265000
H	-2.41378400	2.88653400	3.01483100
P	-3.17656800	3.98653200	0.66153600
O	-4.07801600	4.53130100	1.86118600
Al	-5.22278000	3.98433800	3.14525300
Ni	-3.66133200	1.88275000	0.16986400
C	-7.07230800	4.62273200	2.82400000
H	-7.29916400	4.65920100	1.74326500
H	-7.78488300	3.88742500	3.24542100
C	-4.49804000	4.30403300	4.96223200
H	-4.42470700	5.39451100	5.13582300
H	-3.45861800	3.93527200	5.03353800
C	-4.85150400	1.22237500	2.15680300
O	-5.15406300	2.10243800	3.02217200
N	-4.08339600	0.13036300	2.45392300
C	-3.88597200	-0.40184200	3.84569300
C	-3.08246700	0.62377400	4.65602000
C	-5.32686100	-0.64102500	4.38908200
C	-3.05813800	-1.68892300	3.77066100
H	-2.12148900	0.80241000	4.15755900
H	-3.61307700	1.57583300	4.74667300
H	-2.87226200	0.24217500	5.66122700

H	-5.86899100	-1.17656900	3.59027900
H	-5.78969200	0.35321700	4.45137500
H	-2.82546500	-2.02108100	4.78989300
H	-3.58485900	-2.50840100	3.26342100
H	-2.10561400	-1.51209000	3.25483800
C	-3.89120800	-0.67066900	1.23790800
H	-4.35185500	-1.66707600	1.36619300
C	-2.44851600	-0.83589800	0.79040400
C	-1.92703800	-2.07177100	0.40403000
C	-1.67028300	0.31798100	0.59045400
C	-0.65055100	-2.15730900	-0.16794800
H	-2.52287000	-2.97719800	0.54555000
C	-0.40075100	0.23930200	0.02129700
H	-2.03588300	1.29499400	0.95566200
C	0.11352300	-1.00546200	-0.36369000
H	-0.25498400	-3.13182600	-0.46424300
H	0.18353600	1.15180400	-0.11927400
H	1.10696200	-1.07464600	-0.81264200
C	-5.35835700	1.17549500	0.78072300
C	-4.62785900	0.12320400	0.15305800
C	-7.34047900	6.00783700	3.43879700
H	-8.37765800	6.35865000	3.28722200
H	-7.15678100	6.01233200	4.52692700
H	-6.67746200	6.77811900	3.00705800
C	-5.34989900	3.66557100	6.07228100
H	-5.41613700	2.56955400	5.95271800
H	-4.95906100	3.85159100	7.08959700
H	-6.38764700	4.04117500	6.05620700
C	-6.72831100	1.74650900	0.50776400
C	-7.17167500	1.85244200	-0.95121700
H	-6.79678500	2.73773000	0.97741000
H	-7.46302800	1.12456200	1.05588800
C	-8.51874700	2.55839400	-1.08803200
H	-6.40591000	2.39323800	-1.52388100
H	-7.23645100	0.85014600	-1.40106800
H	-8.82954400	2.63692500	-2.14175600
H	-9.31033300	2.01627400	-0.54474300
H	-8.47662300	3.57718600	-0.67070200
C	-4.91362500	-0.62535600	-1.13140600
C	-4.41041300	0.04864700	-2.41196200
H	-5.99423600	-0.83316900	-1.22700600
H	-4.43035300	-1.61494900	-1.06508000
C	-4.65441600	-0.79119200	-3.66313400
H	-4.89793000	1.02774900	-2.52519200

H	-3.33109100	0.25348800	-2.30173500
H	-4.28451200	-0.28532700	-4.56874200
H	-4.14743300	-1.76824500	-3.59455900
H	-5.73030200	-0.98771600	-3.80614900
C	-5.65842700	-1.38102800	5.71556400
C	-5.45108400	-2.90333700	5.61232300
C	-4.88831300	-0.84101500	6.93122800
C	-7.16231600	-1.12916700	5.95463900
H	-5.96569100	-3.31475100	4.72863000
H	-4.39172000	-3.18449700	5.54752600
H	-5.86618300	-3.40402300	6.50197900
H	-4.96641900	0.25469900	7.00462400
H	-5.30042800	-1.27050100	7.85861200
H	-3.82141100	-1.10669300	6.89893000
H	-7.51022900	-1.65739800	6.85664100
H	-7.36807000	-0.05540600	6.08882200
H	-7.76526000	-1.48356600	5.10278300

IM9

C	-2.01529600	5.04976300	-1.31172800
C	-3.29957100	4.79375500	-2.09115300
H	-2.07352900	6.03524100	-0.81216500
H	-1.14362000	5.06138400	-1.98297700
H	-3.68406500	5.73863700	-2.50197900
H	-3.10790200	4.12067100	-2.95052200
N	-4.25021600	4.19179600	-1.15954900
N	-1.91630600	3.95468500	-0.34565800
C	-5.65706500	4.66774800	-1.14747400
C	-5.74112200	6.11102100	-0.61190200
H	-5.15772800	6.79948400	-1.24358700
H	-6.78362800	6.46618600	-0.61074800
H	-5.34426400	6.16037100	0.40842200
C	-6.23149700	4.63234600	-2.57484000
H	-6.20305400	3.62183600	-3.00039600
H	-7.27944100	4.96761400	-2.55811800
H	-5.68543300	5.30466700	-3.25291400
C	-6.49662500	3.73842300	-0.25695700
H	-6.43598600	2.69695100	-0.60228500
H	-6.17135900	3.77753000	0.79018600
H	-7.55113400	4.05043400	-0.28207800
C	-0.71713700	3.92930000	0.53368800
C	-0.54543600	5.26390200	1.28658400
H	-1.45821000	5.51642400	1.84031300
H	0.29007300	5.19463200	1.99992700

H	-0.31948700	6.08972000	0.59540900
C	0.53218100	3.67016000	-0.32688500
H	1.42859100	3.64590000	0.31118000
H	0.46005700	2.70860400	-0.85465300
H	0.68659500	4.46001300	-1.07647600
C	-0.84110800	2.79300900	1.55990900
H	-1.03585500	1.82571600	1.07378000
H	0.09998500	2.70784800	2.12290900
H	-1.64046300	2.98221200	2.28756800
P	-3.48750800	3.58774000	0.26221200
O	-3.80923000	4.67159300	1.39393300
Al	-4.25253400	4.57663700	3.13576500
Ni	-3.98388500	1.39576500	0.82610800
C	-6.16990200	4.93755400	3.49130300
H	-6.37283400	4.59181800	4.52373100
H	-6.81146700	4.30372700	2.84953500
C	-2.93250900	5.45491900	4.31729300
H	-2.82799300	6.52192100	4.04156300
H	-1.93163300	5.01581600	4.14569000
C	-4.03301200	1.66402100	2.71267300
H	-5.03301300	-0.24927900	-0.96921300
O	-4.00957100	2.76410200	3.35825800
N	-4.03088800	0.55867400	3.47832200
C	-4.06593600	-0.73595200	2.75817500
C	-4.96439400	-1.70839300	3.56960200
C	-4.62354800	-0.34872800	1.39505000
C	-2.61484100	-1.23592500	2.63640500
H	-4.42405400	-1.96639700	4.49564000
H	-5.84360500	-1.13161700	3.89220900
H	-1.99641800	-0.44934800	2.17867400
H	-2.18710400	-1.48016400	3.62160000
C	-3.87332400	0.64140100	4.93016100
H	-3.25811100	-0.20606100	5.26270700
H	-3.31001700	1.56241700	5.13828100
C	-5.17564800	0.66554300	5.70466000
C	-5.31562400	-0.09728400	6.87032900
C	-6.25249500	1.45916600	5.28276500
C	-6.50460400	-0.07105200	7.60480400
H	-4.48648000	-0.72766900	7.20446500
C	-7.44135300	1.48575500	6.01388300
H	-6.15887600	2.06261700	4.38038900
C	-7.57288200	0.72082800	7.17717500
H	-6.59751400	-0.67627600	8.50997800
H	-8.26782100	2.11367500	5.67239700

H	-8.50474900	0.74188600	7.74729300
C	-4.35468500	0.47427900	-1.42458100
C	-3.03390100	0.40450100	-1.10272100
H	-4.41375200	-1.14592600	0.66643500
H	-5.71801400	-0.22294900	1.45988000
H	-2.71778700	-0.37337200	-0.40038300
C	-4.93147500	1.28939500	-2.54376900
C	-4.81408500	0.59548300	-3.91130300
H	-4.45067300	2.27457100	-2.57513900
H	-5.99675800	1.47690300	-2.33789300
C	-5.44295500	1.41633900	-5.03541600
H	-3.74936900	0.41383700	-4.13295500
H	-5.28657700	-0.40118600	-3.86159300
H	-5.34232500	0.91225300	-6.00939500
H	-6.51745300	1.58507800	-4.85529300
H	-4.96476400	2.40626800	-5.11795000
C	-1.92091400	1.08689800	-1.83995300
C	-1.10851700	0.08995900	-2.68314100
H	-1.25064500	1.58123900	-1.12673600
H	-2.30887100	1.89179800	-2.48023200
C	0.02704400	0.76333600	-3.45095500
H	-0.70030900	-0.69535000	-2.02231100
H	-1.78119400	-0.43001100	-3.38659400
H	0.60075600	0.03511200	-4.04524200
H	-0.36053100	1.52968300	-4.14249200
H	0.72983800	1.26529500	-2.76625100
C	-6.59770100	6.40844000	3.36086600
H	-7.65506300	6.58371300	3.63182600
H	-5.99013300	7.06380600	4.00933000
H	-6.46805300	6.78028800	2.33026400
C	-3.29725400	5.34088100	5.80746000
H	-2.55717300	5.81409300	6.47851900
H	-4.27186900	5.81185800	6.02303700
H	-3.38666100	4.28636900	6.12437600
H	-2.54491400	-2.13409800	2.01190900
C	-5.50614200	-3.04446700	2.98732500
C	-6.62752800	-2.82426500	1.95514900
H	-7.40805100	-2.15641600	2.35342400
H	-6.25400100	-2.38698700	1.02069900
H	-7.10394400	-3.78636300	1.70551100
C	-6.11705100	-3.79824000	4.18708600
H	-6.58674800	-4.74036800	3.86209000
H	-5.34757800	-4.04434100	4.93657100
H	-6.88700300	-3.18897700	4.68698500

C	-4.41844800	-3.93301000	2.36405100
H	-4.02095700	-3.50327600	1.43286200
H	-3.57736500	-4.09087900	3.05754900
H	-4.83558000	-4.92221300	2.11464100

IM10

C	-3.53399600	5.85574500	-1.20638800
C	-4.97032600	5.36384100	-1.29011200
H	-3.47471600	6.71582900	-0.51274500
H	-3.17721900	6.18968200	-2.19167600
H	-5.66069700	6.22012900	-1.25911800
H	-5.14398700	4.83403300	-2.24902200
N	-5.14915100	4.48897500	-0.14097700
N	-2.76008700	4.71033400	-0.72856800
C	-6.48424400	3.92703200	0.15338300
C	-7.54646400	5.03154500	0.01942200
H	-7.66424100	5.37414100	-1.01908300
H	-8.52285800	4.64734000	0.35041600
H	-7.28276400	5.89611300	0.64742600
C	-6.81402200	2.77563000	-0.81476400
H	-6.06647500	1.97629600	-0.71636500
H	-7.80736100	2.34750300	-0.60664100
H	-6.80817800	3.12220100	-1.85973900
C	-6.52031700	3.41725400	1.60029400
H	-5.81489400	2.59060000	1.74745700
H	-6.25749800	4.21952900	2.30375600
H	-7.52350900	3.04026700	1.84780400
C	-1.28673900	4.87580000	-0.62687500
C	-0.90602000	6.06567300	0.27515300
H	-1.31404700	5.92936800	1.28472700
H	0.18910700	6.15242800	0.34628900
H	-1.28677000	7.01671300	-0.12672100
C	-0.72408600	5.09256500	-2.04269400
H	0.37501000	5.14024900	-2.00906800
H	-1.01602600	4.26301100	-2.70501600
H	-1.07688000	6.03374200	-2.48965300
C	-0.66030800	3.59529900	-0.06229900
H	-0.86357100	2.73790900	-0.71527500
H	0.42909900	3.72488800	0.01767400
H	-1.03506900	3.36409600	0.94437000
P	-3.66756400	3.88031200	0.48658500
O	-3.40674300	4.66835400	1.84602600
Al	-2.94523800	4.16091100	3.50952100
Ni	-3.64391200	1.54893400	0.65378300

C	-4.18877500	4.91994100	4.85169700
H	-4.18286400	4.28561100	5.75861700
H	-5.22982500	4.87447100	4.47870000
C	-1.00710500	4.34528600	3.87405000
H	-0.73238200	5.41753000	3.84176600
H	-0.41570800	3.87468800	3.06633700
C	-3.74302100	1.47359900	2.56120700
O	-3.28894300	2.34545700	3.37567200
N	-4.27725100	0.37252800	3.12521200
C	-4.67102000	-0.72828100	2.21447200
C	-6.12137400	-1.13660200	2.59490900
C	-4.56683100	-0.12613000	0.81703000
C	-3.65476700	-1.86993500	2.39288000
H	-6.09958100	-1.50633800	3.63142800
H	-6.69697700	-0.19817300	2.63416400
H	-3.76277200	-2.62184100	1.60232400
H	-2.63399700	-1.46642400	2.34386800
H	-3.77474200	-2.38602500	3.35696100
C	-4.34111400	0.21463100	4.58144800
H	-4.14631100	-0.83708900	4.82992900
H	-3.52085100	0.81238100	5.00228300
C	-5.65637700	0.65156100	5.19405900
C	-6.33337500	-0.17660000	6.09752200
C	-6.21501900	1.89536200	4.87004300
C	-7.54788100	0.22332800	6.66263900
H	-5.91029800	-1.15161700	6.35648400
C	-7.42830000	2.29700500	5.43003400
H	-5.69240200	2.55631700	4.18191900
C	-8.10141500	1.46118700	6.32678700
H	-8.06502700	-0.43738500	7.36280500
H	-7.84403500	3.27295700	5.16828200
H	-9.05202900	1.77501600	6.76461600
C	-3.12174100	1.38163100	-1.38957900
C	-2.43194000	0.59792400	-0.71766500
H	-4.29887300	-0.88206700	0.06813400
H	-5.53094400	0.31274100	0.50980700
C	-3.84674300	6.36981400	5.23465200
H	-4.52339700	6.79331500	5.99956400
H	-2.82143000	6.44988000	5.63599600
H	-3.89369000	7.04213600	4.35980900
C	-0.59105400	3.75116100	5.23046100
H	-0.81180100	2.67012100	5.28173600
H	0.48597700	3.86787700	5.45158800
H	-1.14046400	4.22157700	6.06475700

C	-6.97000000	-2.16112600	1.79454700
C	-7.30069500	-1.69595900	0.36585300
C	-6.32817500	-3.55746500	1.73321600
C	-8.29729300	-2.27962200	2.57341500
H	-7.73518300	-0.68336800	0.36445000
H	-6.41571100	-1.68384400	-0.28387900
H	-8.03847100	-2.37646200	-0.08984500
H	-6.00119700	-3.89540700	2.72949700
H	-7.05557600	-4.29253100	1.35268100
H	-5.45886800	-3.58574600	1.06151200
H	-8.98678200	-2.97611800	2.07027400
H	-8.12734000	-2.64980000	3.59706700
H	-8.79910200	-1.30220900	2.65370900
C	-3.59265500	2.13114400	-2.56352300
C	-2.80471300	1.79912800	-3.83882100
H	-3.49115600	3.20335400	-2.33772700
H	-4.66824800	1.94123400	-2.71818000
C	-3.26379700	2.63918800	-5.02814100
H	-1.73238100	1.97275200	-3.64519400
H	-2.90604600	0.72419500	-4.06540000
H	-2.69344100	2.39793500	-5.93858800
H	-4.33140400	2.47167900	-5.24742800
H	-3.13348500	3.71517300	-4.82529100
C	-1.47003600	-0.42138100	-0.26991500
C	-0.59079100	-0.00060000	0.91755100
H	-0.83043600	-0.68057200	-1.13343800
H	-2.02066100	-1.34051400	-0.00844200
C	0.34867400	-1.11549800	1.36749000
H	-0.01320000	0.89528900	0.64134000
H	-1.24334800	0.30789500	1.74985400
H	0.97487500	-0.79234700	2.21312100
H	-0.21438900	-2.00733500	1.68984400
H	1.02222000	-1.42828100	0.55236700

IM11

C	-1.09390400	4.56074000	-1.43154500
C	-2.38311700	4.69468400	-2.22096300
H	-0.71558100	5.56563100	-1.16335500
H	-0.32283600	4.05617000	-2.03082000
H	-2.30809700	5.52031800	-2.94508100
H	-2.57072900	3.76043800	-2.79075600
N	-3.41164200	4.95030400	-1.22122500
N	-1.44452000	3.76853300	-0.25219800
C	-4.83161800	4.96851500	-1.63476600

C	-4.95581800	5.59496900	-3.03153200
H	-4.50627200	4.96632100	-3.81421900
H	-6.01868200	5.72956700	-3.28059400
H	-4.47178400	6.58232600	-3.05374900
C	-5.41970500	3.53898800	-1.67874900
H	-5.76815800	3.16465700	-0.68159900
H	-6.35598700	3.50565400	-2.25766300
H	-4.72020900	2.81988300	-2.12706600
C	-5.61917700	5.83177100	-0.64050700
H	-5.53567100	5.43943100	0.38109500
H	-5.22359500	6.85689700	-0.63131500
H	-6.68760200	5.85614300	-0.90423100
C	-0.34613800	3.42542900	0.69064300
C	0.31111600	4.69478900	1.26549600
H	-0.44052400	5.31508100	1.77195100
H	1.09377600	4.42433500	1.99108500
H	0.78607600	5.29730600	0.47661300
C	0.69730700	2.58185900	-0.06138800
H	1.47287300	2.23369300	0.63731200
H	0.22024000	1.70249700	-0.52117600
H	1.20549900	3.15360200	-0.85180100
C	-0.89312300	2.57922200	1.84556000
H	-1.40120200	1.67973900	1.47688300
H	-0.06193700	2.26431600	2.49329800
H	-1.59451700	3.14081000	2.47194600
P	-3.03323000	4.16183600	0.27061800
O	-2.94132600	5.24826000	1.43067400
Al	-3.63189400	5.17076200	3.11832300
Ni	-4.71531300	2.81890400	0.76529400
C	-5.31325800	6.19879700	3.30772100
H	-5.97430400	6.05433500	2.43463100
H	-5.87999700	5.78507500	4.16421400
C	-2.21730700	5.48811400	4.46251100
H	-1.32787200	4.87371600	4.22745000
H	-1.86883700	6.53606000	4.38920400
C	-4.15007400	2.39572700	2.43668700
O	-3.99659700	3.33425000	3.29757600
N	-3.83202200	1.13885100	2.80293400
C	-4.15279200	-0.02025600	1.88471900
C	-3.34694200	-1.23711200	2.42007800
C	-5.67434100	-0.29221900	1.94612000
C	-3.72639000	0.26952900	0.43948900
H	-2.32753100	-0.87659300	2.62989900
H	-3.78881400	-1.49989900	3.38911600

H	-3.88790400	-0.63370500	-0.15754700
H	-4.34282100	1.04896100	-0.03387300
H	-2.67061200	0.55735200	0.36204100
C	-3.55051500	0.91497000	4.23770000
H	-2.76822600	0.16032400	4.35577900
H	-3.14794000	1.86320700	4.61127600
C	-4.78125500	0.53296000	5.03432800
C	-4.85105400	-0.69163100	5.71101800
C	-5.88336600	1.40175700	5.09677300
C	-5.99937300	-1.05571100	6.42135500
H	-3.99532900	-1.37203800	5.68455400
C	-7.03096100	1.03866800	5.80162400
H	-5.83793700	2.36316100	4.58617300
C	-7.09678000	-0.19309000	6.46162400
H	-6.03543800	-2.01574900	6.94215400
H	-7.88187600	1.72319000	5.83475500
H	-7.99849300	-0.47567100	7.01018700
C	-6.34648600	2.13360600	1.53608000
C	-6.64568500	0.87393200	1.92323100
H	-5.92775000	-0.97794800	1.12397700
H	-5.88238100	-0.86078200	2.86704000
C	-5.07684500	7.70318600	3.52349100
H	-6.01010100	8.27617800	3.67368500
H	-4.44093600	7.89013800	4.40612400
H	-4.55758500	8.15851700	2.66182000
C	-2.68351700	5.19801900	5.89910400
H	-3.01723800	4.15132400	6.01363900
H	-1.90035300	5.36983600	6.66014900
H	-3.54458300	5.82962500	6.17990200
C	-3.16392900	-2.58488300	1.65590500
C	-4.46211600	-3.16253000	1.06873100
C	-2.08616800	-2.50550000	0.55624100
C	-2.64847500	-3.57628700	2.72228800
H	-5.25390900	-3.22981400	1.83004800
H	-4.84736700	-2.56503000	0.23004200
H	-4.27850300	-4.17916100	0.68522100
H	-1.15580600	-2.06316300	0.94746600
H	-1.84607900	-3.51674800	0.19052500
H	-2.39798000	-1.91200300	-0.31230000
H	-2.42473200	-4.55617100	2.27196600
H	-1.72508300	-3.20551300	3.19624200
H	-3.39634600	-3.73106300	3.51647400
C	-7.32942200	3.27939300	1.71850200
C	-8.39084700	3.42545300	0.61904500

H	-6.77397200	4.22767500	1.78692600
H	-7.84451300	3.19182600	2.69298000
C	-9.27292600	4.65719900	0.81358400
H	-7.89757400	3.47601200	-0.36518500
H	-9.01455300	2.51694400	0.58808000
H	-10.02879400	4.74968700	0.01722700
H	-9.80603700	4.61536500	1.77777100
H	-8.66994100	5.58028600	0.81628300
C	-8.02833400	0.45633200	2.38884800
C	-8.79801600	-0.38988100	1.36128800
H	-8.63599800	1.33750300	2.64165000
H	-7.93383000	-0.12550600	3.32329400
C	-10.20399000	-0.75736200	1.83087300
H	-8.85145000	0.16609000	0.40906900
H	-8.23182100	-1.31151700	1.14065900
H	-10.73606200	-1.37121200	1.08661500
H	-10.17190400	-1.32712900	2.77456800
H	-10.80984700	0.14580600	2.01422100

IM12

C	-1.71617600	4.33159500	-1.68706900
C	-3.04906800	4.52529700	-2.38199500
H	-1.25141600	5.31948900	-1.50397400
H	-1.03096300	3.74752500	-2.31965500
H	-2.97090300	5.32688500	-3.12908100
H	-3.34209700	3.59751000	-2.92095300
N	-3.97104200	4.88267800	-1.31613100
N	-2.01140600	3.62873800	-0.44266400
C	-5.35146800	5.29730800	-1.65849100
C	-5.35404800	6.09616700	-2.97136300
H	-5.11524200	5.47217300	-3.84531300
H	-6.35731600	6.51839300	-3.13125500
H	-4.63780400	6.93045800	-2.92527300
C	-6.27161800	4.07237000	-1.83160100
H	-6.31040600	3.47864400	-0.90165200
H	-7.30179900	4.36899600	-2.08270200
H	-5.89765900	3.42035800	-2.63714100
C	-5.84581600	6.23398800	-0.54620500
H	-5.72645500	5.78784600	0.44524800
H	-5.24762700	7.15652200	-0.54783100
H	-6.90564100	6.49342200	-0.69008900
C	-0.87761900	3.46765300	0.50362200
C	-0.44840800	4.81957900	1.10950100
H	-1.26840300	5.26419000	1.68515000

H	0.41770900	4.68210100	1.77554800
H	-0.15119700	5.53234200	0.32531900
C	0.31289200	2.84149100	-0.24365900
H	1.10228500	2.57637900	0.47595100
H	0.00013000	1.92688400	-0.77123200
H	0.75677500	3.53255000	-0.97496900
C	-1.28555900	2.50572200	1.61853300
H	-1.46250900	1.50403200	1.21257800
H	-0.49182400	2.44350000	2.37718300
H	-2.19558100	2.84106500	2.12443300
P	-3.64292000	3.90706000	0.08731800
O	-3.62734700	4.86381000	1.35986500
Al	-4.15924900	4.84363000	3.09858300
Ni	-5.06212500	2.21927900	0.41177100
C	-5.62647200	6.13390700	3.40041000
H	-6.46368000	5.95959200	2.70345300
H	-6.04813700	5.94898900	4.40768300
C	-2.59430300	4.98055900	4.30805500
H	-1.80098600	4.27057500	4.00922400
H	-2.13313800	5.98021000	4.19240500
C	-5.09402300	2.03107600	2.75661300
O	-4.77762000	3.10464800	3.39238900
N	-4.36828500	0.90366600	3.03322800
C	-4.47820300	-0.34857000	2.21011300
C	-4.28337300	-1.54391000	3.17846400
C	-5.86429200	-0.40471600	1.56118600
C	-3.42162100	-0.26489700	1.09602000
H	-3.38791300	-1.35422200	3.78093600
H	-5.12200900	-1.48897300	3.89030800
H	-3.57515000	-1.04924700	0.34417300
H	-3.50716600	0.71732600	0.59092700
H	-2.39771900	-0.35583200	1.48075200
C	-3.22520900	1.04424100	3.94032900
H	-2.37554800	0.47229300	3.54636700
H	-2.92607000	2.09705900	3.94034300
C	-3.51060800	0.63254500	5.37012700
C	-2.55949300	-0.09290200	6.09789600
C	-4.70480400	1.00512300	6.00148300
C	-2.79388000	-0.44567900	7.42956800
H	-1.62389100	-0.38969200	5.61456000
C	-4.94324600	0.65150000	7.33097800
H	-5.44592900	1.58475800	5.44960200
C	-3.98975200	-0.07603300	8.04971400
H	-2.04172200	-1.01418500	7.98202800

H	-5.87801300	0.95130600	7.81097900
H	-4.17805200	-0.35244300	9.08980600
C	-6.28987000	2.02143900	1.91904500
C	-6.41502600	0.90925700	1.04038300
H	-5.82833200	-1.14638600	0.75618000
H	-6.59477400	-0.79056300	2.29685300
C	-5.17200900	7.59920100	3.29320400
H	-5.97883100	8.32485000	3.50403000
H	-4.35054000	7.82155700	3.99618200
H	-4.79084500	7.82879700	2.28277600
C	-2.96298100	4.74898400	5.78353100
H	-3.39819000	3.74686000	5.94009000
H	-2.10170400	4.83481300	6.47113600
H	-3.71956400	5.47495700	6.12919400
C	-4.13727200	-3.02179600	2.71547700
C	-5.24490600	-3.51323200	1.77003900
C	-2.76523600	-3.28930500	2.06934200
C	-4.21313200	-3.85467600	4.01298100
H	-6.24709500	-3.29464100	2.16894800
H	-5.16945200	-3.06328000	0.76986400
H	-5.16752800	-4.60464400	1.63956100
H	-1.94638400	-2.92139200	2.70854200
H	-2.61682700	-4.37200800	1.92910100
H	-2.66305200	-2.81731600	1.08361500
H	-4.05312300	-4.92404600	3.80243900
H	-3.44826100	-3.53262500	4.73761300
H	-5.19648700	-3.74683000	4.49751800
C	-7.42329900	2.95657900	2.31982300
C	-7.92315500	4.00595000	1.32791900
H	-7.11826500	3.48184800	3.23628800
H	-8.27305300	2.30725600	2.60325500
C	-9.06150300	4.84758500	1.89827000
H	-7.08405800	4.65350800	1.05615600
H	-8.25137100	3.53285200	0.39013300
H	-9.39433500	5.61272300	1.17913200
H	-9.93471500	4.22307900	2.15171200
H	-8.74625800	5.36797900	2.81682000
C	-7.62834800	0.71901000	0.15248900
C	-7.25253900	0.30925400	-1.27827900
H	-8.22753900	1.63769700	0.10452300
H	-8.29234000	-0.05608500	0.58524100
C	-8.46125700	0.11096000	-2.18914900
H	-6.59195100	1.08631100	-1.70224800
H	-6.64958500	-0.61525800	-1.25340200

H	-8.15981800	-0.17533800	-3.20907100
H	-9.12637400	-0.67877400	-1.80193800
H	-9.05775000	1.03543600	-2.26358900

TS1

C	-3.44307600	5.77609300	-1.63224000
C	-4.80620100	5.08416000	-1.65468200
H	-3.48440900	6.70570100	-1.03061500
H	-3.12538700	6.04462800	-2.64928400
H	-5.61315100	5.83223800	-1.62836200
H	-4.92112900	4.50133500	-2.58766100
N	-4.86047200	4.20463300	-0.48244000
N	-2.55955900	4.77742800	-1.04498000
C	-6.15025000	4.03679000	0.21938600
C	-6.55278000	5.33448000	0.94745100
H	-6.64464300	6.17559200	0.24252000
H	-7.52560500	5.21647800	1.45052900
H	-5.79268200	5.59539300	1.69571300
C	-7.22525500	3.64399500	-0.80832900
H	-6.91879500	2.73918900	-1.35624600
H	-8.17919200	3.43712400	-0.29954300
H	-7.40990700	4.44314800	-1.54154100
C	-6.03269700	2.90342200	1.24423200
H	-5.79817300	1.94969100	0.75100900
H	-5.26063700	3.10433800	1.99623400
H	-6.98638400	2.78406900	1.77854700
C	-1.09665100	4.85390600	-1.17106400
C	-0.53435200	6.05380300	-0.38795300
H	-0.82279600	5.98684700	0.67097400
H	0.56451700	6.09543300	-0.45359800
H	-0.93201000	6.99943700	-0.78808300
C	-0.71844100	4.95658600	-2.65758800
H	0.37518100	4.91787100	-2.77463400
H	-1.16331100	4.12232300	-3.22156100
H	-1.06023500	5.90180800	-3.10374600
C	-0.49283300	3.54569800	-0.62531500
H	-0.98998400	2.67093600	-1.08431200
H	0.57847500	3.46760200	-0.85760800
H	-0.54503900	3.51452500	0.48862600
P	-3.33738100	4.01578000	0.27505000
O	-3.36905600	4.96749000	1.57028600
Al	-2.87499800	4.49432600	3.26006200
Ni	-2.09177000	2.31954800	0.82383500
C	-4.19916100	5.21210100	4.53870800

H	-5.17156300	4.71733100	4.34789800
H	-3.92102600	4.90592300	5.56575200
C	-0.92765200	4.72506400	3.57364100
H	-0.73913000	5.76368500	3.90732800
H	-0.36528000	4.62594600	2.62607100
C	-2.79319500	1.76929800	2.41346300
H	-1.39204700	1.74435700	2.12587500
O	-3.16026300	2.63426600	3.29059600
N	-3.23417900	0.47440400	2.54745100
C	-3.91691800	0.02087800	3.82218000
C	-4.30132200	-1.46220700	3.69752200
C	-2.87023400	0.20577200	4.96558000
C	-5.23600700	0.79805400	3.97423100
H	-4.94280100	-1.63821800	2.82023900
H	-3.42853400	-2.12788000	3.64227800
H	-4.87422900	-1.75427500	4.58491400
H	-2.31219300	1.12553000	4.75980900
H	-2.14897400	-0.62195100	4.84868900
H	-5.81854200	0.39432700	4.81332200
H	-5.07780300	1.86657100	4.13245100
H	-5.83201000	0.66547000	3.05966800
C	-2.55705300	-0.50306900	1.68684300
H	-1.57114600	-0.77161500	2.11190500
H	-3.15532300	-1.41998000	1.66619900
C	-2.35635600	-0.05773800	0.24996700
C	-1.15465200	-0.35511000	-0.42461100
C	-3.40287600	0.54202900	-0.48369000
C	-1.00518500	-0.07220700	-1.78239700
H	-0.33033700	-0.80801800	0.13219300
C	-3.24696800	0.83266600	-1.84639900
H	-4.35197300	0.74396600	0.01176900
C	-2.05174800	0.52768000	-2.49763500
H	-0.06433100	-0.30945300	-2.28413000
H	-4.06450800	1.31748000	-2.38369200
H	-1.92883500	0.76040600	-3.55779500
C	-4.37961600	6.73781000	4.48628000
H	-5.12871500	7.11660200	5.20577700
H	-3.43446900	7.26575400	4.70426800
H	-4.69934700	7.07134000	3.48380800
C	-0.35607700	3.74051900	4.60772800
H	-0.43926900	2.69730200	4.25622500
H	0.71063000	3.91447300	4.84009000
H	-0.90212500	3.79095800	5.56665100
C	-3.24483600	0.29033900	6.47081800

C	-3.93069300	1.62749300	6.80926500
C	-4.11021800	-0.87931900	6.96506700
C	-1.90252300	0.25066600	7.23045100
H	-3.35774900	2.47730300	6.40794900
H	-4.94774800	1.69578200	6.40445600
H	-4.00257400	1.75081800	7.90197900
H	-3.65172400	-1.85194700	6.72369500
H	-4.22652100	-0.82886400	8.05980600
H	-5.12180600	-0.86080300	6.53298900
H	-2.06289400	0.35147000	8.31577500
H	-1.36990900	-0.69813500	7.05389000
H	-1.24347500	1.07297300	6.90879300

TS2

C	-3.63690700	6.07398000	-1.67887600
C	-4.96081300	5.32991300	-1.74362500
H	-3.74118200	6.97186000	-1.03956000
H	-3.32914100	6.40560000	-2.68064900
H	-5.79551800	6.04627600	-1.77785700
H	-5.01063200	4.71278900	-2.66362900
N	-5.00732600	4.51507300	-0.53846500
N	-2.68298700	5.11232400	-1.12965700
C	-6.19806500	3.68280500	-0.27064600
C	-7.46634600	4.52211400	-0.49745700
H	-7.60922500	4.78469600	-1.55611800
H	-8.35118900	3.95084800	-0.17854900
H	-7.42261800	5.45123600	0.09113700
C	-6.22560000	2.44297900	-1.18450900
H	-5.33755500	1.81933200	-1.00467600
H	-7.12448400	1.83433900	-0.99765800
H	-6.23130000	2.73251000	-2.24650200
C	-6.17552400	3.23708800	1.19410800
H	-5.31459100	2.58924100	1.39028800
H	-6.11239000	4.10053500	1.87073400
H	-7.08510500	2.66585100	1.43098100
C	-1.25521300	5.51761300	-1.07001100
C	-1.06029200	6.76652000	-0.18972500
H	-1.40069700	6.57460200	0.83488000
H	0.00268600	7.05116200	-0.15817900
H	-1.62290300	7.62662200	-0.58341200
C	-0.76897100	5.81193900	-2.50106300
H	0.31431600	6.00533200	-2.49416100
H	-0.96307400	4.95196000	-3.16045300
H	-1.25400200	6.69919200	-2.93376800

C	-0.40297300	4.36448000	-0.52507700
H	-0.44045900	3.49483400	-1.19265100
H	0.64226900	4.69479300	-0.43399100
H	-0.72959700	4.03885800	0.47182900
P	-3.45380700	4.19312500	0.11228700
O	-3.34163600	5.04489900	1.45779800
Al	-2.61494700	4.74028900	3.07759500
Ni	-2.63760300	2.15500600	0.36336300
C	-3.82609900	5.39845100	4.49614800
H	-3.53075200	4.95091900	5.46468400
H	-4.85385600	5.03768200	4.30586900
C	-0.70209900	5.21720100	3.26509300
H	-0.10437400	4.78743800	2.44128500
H	-0.57450600	6.31268000	3.17220300
C	-2.96718500	1.96390400	2.25273200
H	-1.87721200	1.45213600	1.45023600
O	-2.69462300	2.87431900	3.09795700
N	-3.68856100	0.88672700	2.67953900
C	-4.23001400	0.74511100	4.09480700
C	-3.10579800	1.02330500	5.11181800
C	-5.42120200	1.73725100	4.17298800
C	-4.68142100	-0.71024900	4.30751200
H	-2.17726800	0.52402300	4.79753000
H	-2.89471000	2.08828300	5.23014900
H	-3.38555100	0.60974900	6.08920500
H	-6.10315300	1.44730700	3.35786400
H	-5.01703400	2.71871600	3.89607500
H	-4.91661800	-0.85629800	5.36721300
H	-5.58330800	-0.96931000	3.73756700
H	-3.88633400	-1.42103300	4.04897200
C	-4.06484800	-0.07660900	1.65036400
H	-5.09616900	-0.41112100	1.82388700
H	-4.09559200	0.45933800	0.68769600
C	-3.14737400	-1.27247700	1.49782600
C	-3.47843700	-2.26381800	0.56357400
C	-1.95967500	-1.40251600	2.22469700
C	-2.64129000	-3.36210700	0.36225200
H	-4.39874700	-2.16755600	-0.01964100
C	-1.12281200	-2.50505900	2.03245000
H	-1.68832400	-0.63175400	2.94624000
C	-1.45841200	-3.48783300	1.09876900
H	-2.91163800	-4.12373100	-0.37303400
H	-0.20020300	-2.58988300	2.61104200
H	-0.80225000	-4.34711700	0.94315800

C	-1.71494400	0.95015500	-0.88367700
C	-2.45819000	1.73216800	-1.54359000
C	-2.91425100	2.30050300	-2.82999400
H	-3.95231100	1.98094500	-3.02686200
H	-2.94813600	3.39613700	-2.72887800
C	-0.66281500	-0.08758700	-0.78321700
H	-1.10393300	-1.02644800	-0.41418500
H	-0.29387400	-0.29769600	-1.80377000
C	-2.01263900	1.91208300	-4.00859300
C	-2.47421400	2.53588100	-5.32379900
H	-1.97919000	0.81265600	-4.09903500
H	-0.98023100	2.23052400	-3.78238900
H	-1.81666200	2.25372400	-6.16103700
H	-3.49816300	2.21620600	-5.57996400
H	-2.48040200	3.63686700	-5.25903300
C	0.51823700	0.29761100	0.11532500
C	1.61780400	-0.76072000	0.12548300
H	0.14110400	0.46187500	1.13860900
H	0.92543900	1.26814000	-0.21592800
H	2.44260000	-0.47780800	0.79816900
H	1.22541100	-1.73385400	0.46167200
H	2.04357600	-0.90705100	-0.88115100
C	-6.30826800	1.96329300	5.42900800
C	-7.12133900	0.72249100	5.83689400
C	-5.51452700	2.47805600	6.64141500
C	-7.30938300	3.06368400	5.01541600
H	-7.63848200	0.27709100	4.97161200
H	-6.49976500	-0.05648700	6.29879900
H	-7.88869700	1.00177500	6.57673300
H	-4.88479800	3.33740600	6.36870600
H	-6.20730000	2.80579300	7.43332100
H	-4.86817900	1.70311300	7.07545600
H	-7.99389500	3.30036900	5.84531100
H	-6.78589700	3.99007900	4.73382700
H	-7.91892900	2.74560400	4.15410500
C	-3.83525500	6.93104800	4.62036500
H	-4.50118900	7.30369600	5.42019200
H	-2.82655900	7.32494000	4.83567600
H	-4.16474800	7.40803400	3.68084800
C	-0.12876500	4.74786500	4.61389700
H	-0.67738500	5.19003500	5.46400300
H	-0.20764600	3.65182700	4.72600200
H	0.93677400	5.00732200	4.75263200

TS3

C	-2.83898700	5.61390500	-2.28547300
C	-4.31936700	5.28738900	-2.46416600
H	-2.72532200	6.59752000	-1.78991300
H	-2.32628600	5.66289900	-3.25736000
H	-4.88936600	6.21014600	-2.65099200
H	-4.46831700	4.61860900	-3.33293300
N	-4.75215700	4.64000000	-1.22708300
N	-2.30505900	4.52482300	-1.47070400
C	-6.10496700	4.89167800	-0.68347400
C	-6.18015400	6.30791700	-0.08292400
H	-5.95340100	7.07052100	-0.84466500
H	-7.18743900	6.51788000	0.31036000
H	-5.45324100	6.41064700	0.73276600
C	-7.13692400	4.74196700	-1.81357300
H	-7.06420900	3.74824600	-2.27873000
H	-8.15416000	4.86373300	-1.41185800
H	-7.00138600	5.49859700	-2.59993300
C	-6.42899400	3.85554100	0.40363200
H	-6.37467200	2.83321100	0.00394100
H	-5.74798500	3.93226700	1.26045600
H	-7.44814000	4.02488600	0.78132500
C	-0.84294800	4.43424000	-1.25377700
C	-0.29165300	5.76388600	-0.70703300
H	-0.80676200	6.03038600	0.22758400
H	0.78632000	5.67760900	-0.50073200
H	-0.42610000	6.58710400	-1.42517100
C	-0.17705800	4.07638600	-2.59266700
H	0.90938100	3.95463100	-2.46272100
H	-0.59717700	3.13488100	-2.97693900
H	-0.33176700	4.85921600	-3.35040400
C	-0.52836700	3.32014500	-0.24697500
H	-0.92626500	2.35656800	-0.58692100
H	0.56143500	3.21917900	-0.13850800
H	-0.93800900	3.54559000	0.74680700
P	-3.44641200	4.06808400	-0.29736000
O	-3.31425800	5.02053100	0.97184000
Al	-3.24748100	4.86009900	2.77324700
Ni	-3.43452800	1.98283300	0.25068200
C	-4.68271800	5.90412000	3.64255500
H	-4.73127500	5.60166800	4.70693100
H	-5.66083800	5.60711900	3.21815700
C	-1.37882700	5.01061400	3.41127400
H	-0.99856000	6.02701000	3.19090400

H	-0.73934200	4.33554600	2.80987600
C	-3.57459900	2.14742000	2.11751500
H	-1.54758400	0.57084000	-1.50447200
O	-3.71704300	3.05221300	3.00197500
N	-3.70736900	0.84421100	2.40246700
C	-4.10488100	0.22044800	3.71664400
C	-3.07556300	0.66915600	4.76864700
C	-5.54434500	0.75270000	3.97012900
C	-4.03940900	-1.30271500	3.57563300
H	-2.06182100	0.42017300	4.42170600
H	-3.12577100	1.75116200	4.93841300
H	-3.23914700	0.15320800	5.72229000
H	-6.09630100	0.59576900	3.02729500
H	-5.44467500	1.84211100	4.07605600
H	-4.20419500	-1.76235300	4.55796100
H	-4.80326000	-1.69621400	2.89049700
H	-3.05014500	-1.62476000	3.22179800
C	-3.40339700	0.09064300	1.17473200
H	-4.14530900	-0.70262600	1.01406000
H	-3.65232800	0.52818000	-0.17236600
C	-2.01197900	-0.45799400	1.06947000
C	-1.78702600	-1.61545300	0.30907600
C	-0.90598200	0.21979100	1.60755800
C	-0.49090200	-2.08509200	0.08664600
H	-2.63847600	-2.14666100	-0.12383000
C	0.38876500	-0.25154300	1.39001100
H	-1.05960300	1.13211400	2.18573000
C	0.60330700	-1.40278900	0.62420100
H	-0.33635000	-2.98630500	-0.51140900
H	1.23737900	0.28957800	1.81511300
H	1.61860100	-1.76611000	0.44982400
C	-3.55053000	1.20035600	-1.58898300
C	-2.42860500	0.69762100	-2.14615800
C	-2.19301600	0.25214400	-3.56280800
H	-3.05581700	0.48343200	-4.20674700
H	-1.33892200	0.82163600	-3.97701800
C	-4.86709800	1.34347800	-2.30914100
H	-5.19209100	2.39489800	-2.24207800
H	-4.77240000	1.11010300	-3.38378700
C	-4.52049900	7.43024700	3.55087700
H	-4.52119100	7.77674700	2.50248000
H	-5.32051300	7.98830700	4.07096700
H	-3.56315500	7.76298500	3.98867500
C	-1.18678200	4.70241000	4.90540400

H	-0.13785700	4.79427800	5.24224700
H	-1.78510500	5.37988700	5.53925200
H	-1.51123300	3.67602900	5.15187500
C	-6.46722300	0.25561100	5.11696300
C	-7.65964500	1.23467300	5.13665400
H	-8.39970700	0.93658300	5.89634200
H	-7.32883800	2.25975700	5.36688000
H	-8.16938000	1.25861700	4.15975500
C	-7.02606900	-1.15579100	4.85754400
H	-7.48117200	-1.22376200	3.85602100
H	-6.25810100	-1.93680700	4.93299000
H	-7.80809000	-1.39655400	5.59578000
C	-5.79518900	0.29230300	6.49875900
H	-5.01858300	-0.47956100	6.60251200
H	-5.33147600	1.27126900	6.69567800
H	-6.54227900	0.10851800	7.28773000
C	-5.96098100	0.44679500	-1.70726400
C	-7.30779800	0.56953800	-2.41521700
H	-6.08314200	0.69324700	-0.63695200
H	-5.61652000	-0.60253300	-1.73560800
H	-8.06381800	-0.09674000	-1.97032200
H	-7.69584600	1.59916700	-2.35585300
H	-7.22110400	0.31195900	-3.48378400
C	-1.86757300	-1.24708400	-3.65855300
C	-1.53923200	-1.70214600	-5.07880500
H	-2.72612500	-1.82043300	-3.26639900
H	-1.02313600	-1.47488300	-2.98440800
H	-1.31585300	-2.78015700	-5.11940700
H	-2.38130800	-1.50944100	-5.76441500
H	-0.66276800	-1.16369900	-5.47638900

TS3’

C	-3.45407100	5.53980400	-2.09860300
C	-4.80136700	4.82457000	-2.10085200
H	-3.58247200	6.57937300	-1.73856500
H	-3.04123600	5.58473800	-3.11738700
H	-5.60181400	5.54023200	-2.33975600
H	-4.82028400	4.02852300	-2.86902800
N	-4.97537600	4.26997600	-0.76753200
N	-2.58490600	4.76363400	-1.21809900
C	-6.29214500	4.27158400	-0.10005400
C	-6.58085300	5.67329100	0.46994300
H	-6.58524300	6.42559000	-0.33500100
H	-7.56288900	5.70868700	0.96846900

H	-5.80326600	5.95014000	1.19415100
C	-7.39042000	3.86824600	-1.09528800
H	-7.19053300	2.87152000	-1.50867200
H	-8.36327600	3.84410900	-0.58123900
H	-7.47951200	4.57755100	-1.93077500
C	-6.28760200	3.24013700	1.03264900
H	-6.04457400	2.23957600	0.64616900
H	-5.56494600	3.49918000	1.81259200
H	-7.27857700	3.20201200	1.50836100
C	-1.20754100	5.26921700	-0.98354900
C	-1.22863900	6.64385200	-0.28421800
H	-1.74446900	6.57321700	0.68214000
H	-0.20130100	7.00130600	-0.11350700
H	-1.74350100	7.40018900	-0.89578900
C	-0.48498800	5.38788000	-2.33639500
H	0.56558900	5.67372200	-2.17567800
H	-0.50852600	4.42896000	-2.87426200
H	-0.93755900	6.15706000	-2.97921900
C	-0.42301400	4.28118100	-0.11033600
H	-0.36631800	3.28986800	-0.57528800
H	0.60249500	4.65336600	0.03113100
H	-0.86724100	4.17112200	0.88655400
P	-3.49474600	3.98752200	0.01538000
O	-3.45245000	4.92435700	1.31090100
Al	-3.14223000	4.66385200	3.06296400
Ni	-2.65964000	1.98971100	0.34423200
C	-4.67797400	5.34980900	4.10742900
H	-4.60126100	5.01880200	5.15857700
H	-5.62705800	4.92059000	3.73613400
C	-1.34735800	5.23238000	3.67750000
H	-1.28550800	6.33389000	3.58751100
H	-0.55037900	4.85490400	3.01088600
C	-3.12576300	1.81060500	2.28769500
H	-5.01366500	0.88699700	-0.96436300
O	-3.19404100	2.79643100	3.08955200
N	-3.84257100	0.69241200	2.60303000
C	-4.61170300	0.55618400	3.91828600
C	-5.24473800	-0.84434900	3.98945400
C	-3.57202800	0.69119700	5.07088100
C	-5.75667000	1.57655900	3.93827700
H	-5.69312100	-0.96871700	4.98026800
H	-6.05240100	-0.97583900	3.25638300
H	-4.50901600	-1.64704600	3.85378800
H	-2.85130600	1.45974100	4.78261200

H	-3.02282200	-0.26330100	5.09309300
H	-6.39023300	1.41303400	4.82026000
H	-5.40000300	2.60869200	3.95065200
H	-6.38475300	1.43202300	3.04883100
C	-3.98420900	-0.32992700	1.56367500
H	-5.02401500	-0.68003400	1.56446700
H	-3.85809200	0.15614300	0.58928900
C	-3.04429800	-1.51645800	1.60435900
C	-3.08844600	-2.41412000	0.52838800
C	-2.11000700	-1.73418300	2.62128400
C	-2.22315100	-3.50785800	0.47381300
H	-3.79413700	-2.23665100	-0.28799400
C	-1.24159400	-2.82781800	2.57097200
H	-2.04277000	-1.02765900	3.44713400
C	-1.29486500	-3.72032600	1.49748500
H	-2.26793800	-4.19308300	-0.37618200
H	-0.51456600	-2.97670800	3.37315800
H	-0.61336200	-4.57302900	1.45485300
C	-4.29142900	1.02933700	-1.78065200
C	-3.07558800	1.54159600	-1.50817500
C	-1.23718400	0.73437300	0.63221200
C	-1.39596400	1.31311000	1.78698000
C	-4.76480600	6.88605600	4.06439600
H	-5.60426400	7.29237600	4.65734200
H	-3.84396100	7.35497000	4.45298700
H	-4.89432100	7.25760600	3.03306000
C	-1.05258900	4.82433200	5.13130500
H	-1.07857700	3.72780400	5.26120300
H	-0.06412800	5.16234900	5.49215400
H	-1.80375800	5.23753700	5.82642200
C	-3.94349600	1.03718900	6.54250100
C	-4.33235400	2.51797100	6.69946300
C	-5.03604900	0.14625000	7.15342100
C	-2.64410000	0.81469500	7.34520200
H	-3.56048600	3.17348700	6.26955000
H	-5.28494600	2.76360700	6.21504300
H	-4.43067000	2.77179900	7.76721600
H	-4.78410600	-0.92263400	7.06531900
H	-5.14776000	0.37265800	8.22600600
H	-6.01895900	0.30810200	6.68676900
H	-2.78513400	1.08884200	8.40259000
H	-2.32518900	-0.23966100	7.30864000
H	-1.82273300	1.43138100	6.94556800
C	-0.25442800	-0.16744900	-0.02009800

C	0.87082500	0.58245600	-0.75146900
H	-0.78249200	-0.81467100	-0.73739000
H	0.18589200	-0.84621600	0.72981600
C	1.82184700	-0.36070300	-1.48244000
H	0.42899300	1.29468500	-1.46227300
H	1.42814700	1.19358600	-0.02285400
H	2.62947700	0.19239200	-1.98710700
H	2.28803500	-1.07748100	-0.78620800
H	1.28789100	-0.94486900	-2.24965400
C	-0.59898800	1.60045100	3.02473100
C	0.79093200	0.96253100	3.01754300
H	-1.14065000	1.25621900	3.91875400
H	-0.51153100	2.69173200	3.14186400
C	1.55909200	1.28294300	4.29866600
H	0.69324700	-0.12904200	2.89836600
H	1.35424300	1.32006400	2.14097700
H	2.56448000	0.83385100	4.29012700
H	1.67568300	2.37110800	4.43003100
H	1.02884300	0.90166900	5.18728900
C	-2.07851900	1.80873500	-2.61962200
C	-1.50241900	0.56587700	-3.31561800
H	-1.24508200	2.41172200	-2.23577200
H	-2.55422400	2.44990200	-3.38643800
C	-0.41908600	0.91399700	-4.33474500
H	-1.09797600	-0.12109600	-2.55948000
H	-2.31058400	0.00769700	-3.81254400
H	-0.01647300	0.01458100	-4.82782100
H	-0.81164600	1.57954600	-5.12191100
H	0.42626600	1.43752100	-3.85742800
C	-4.83109700	0.61778400	-3.13306900
C	-6.28258300	1.04132300	-3.37696300
H	-4.76941200	-0.48407100	-3.24014700
H	-4.20435200	1.02776500	-3.94106400
C	-6.84385300	0.53341800	-4.70350400
H	-6.91304100	0.68041900	-2.54407500
H	-6.34218900	2.14175400	-3.34523400
H	-7.88604500	0.85628400	-4.85660400
H	-6.25061200	0.90629500	-5.55531100
H	-6.82404800	-0.56843100	-4.74922300

TS4

C	-2.92133500	5.38014200	-1.73843900
C	-4.35894300	4.97138300	-2.04836200
H	-2.91038900	6.30673300	-1.13275500

H	-2.36367300	5.57527400	-2.66516400
H	-4.99048000	5.86738100	-2.14986200
H	-4.40794400	4.42150500	-3.00750300
N	-4.80348900	4.12981400	-0.94029400
N	-2.34472000	4.24661200	-1.01550600
C	-6.22026900	4.17042700	-0.51505400
C	-6.52660400	5.50968100	0.18269100
H	-6.33502700	6.36196800	-0.48783600
H	-7.58261600	5.56086900	0.49169800
H	-5.89135700	5.62296400	1.07113200
C	-7.11547100	3.99609600	-1.75354200
H	-6.89316900	3.04575200	-2.26275900
H	-8.17462500	3.98800300	-1.45473300
H	-6.98439300	4.81315100	-2.47822800
C	-6.52273400	3.01823300	0.45498100
H	-6.30305900	2.04265900	-0.00095700
H	-5.95144600	3.10398400	1.38797200
H	-7.58916800	3.04411200	0.72458700
C	-0.89966900	4.27486900	-0.67690500
C	-0.56084600	5.48433000	0.21513600
H	-1.15146200	5.45825700	1.14063900
H	0.50765200	5.47728900	0.48047100
H	-0.76863000	6.43541600	-0.29884300
C	-0.08929900	4.34146400	-1.98211700
H	0.98675900	4.29150300	-1.75689900
H	-0.34606300	3.49757400	-2.63979000
H	-0.26325400	5.27755900	-2.53330800
C	-0.52365300	2.98146800	0.05419000
H	-0.77724900	2.09455300	-0.54319200
H	0.55765800	2.96759800	0.25123700
H	-1.03080000	2.89825200	1.02401300
P	-3.51940300	3.61535600	0.05496200
O	-3.56717300	4.51360600	1.37015200
Al	-3.57622600	4.23569300	3.15817000
Ni	-3.46335100	1.52786500	0.51546300
C	-5.00196800	5.30582500	4.00625200
H	-5.05062700	5.05549700	5.08372700
H	-5.97614000	4.97932700	3.59334400
C	-1.71522500	4.21753800	3.84239200
H	-1.29930700	5.24317600	3.82282100
H	-1.09923500	3.64973000	3.11851100
C	-3.76183900	1.58830200	2.38518400
O	-4.12858800	2.43150700	3.26623200
N	-3.82033000	0.25841700	2.60050600

C	-4.41196500	-0.46512500	3.77945600
C	-3.73906000	0.07407500	5.05368600
C	-5.93123700	-0.14443400	3.68947600
C	-4.08846200	-1.95775000	3.65564600
H	-2.64542000	0.00262400	4.95689100
H	-3.99907500	1.12325200	5.23008200
H	-4.03401100	-0.51856300	5.92789500
H	-6.22107300	-0.34087500	2.64335400
H	-6.00884200	0.94562600	3.81497800
H	-4.36508900	-2.46875800	4.58658700
H	-4.63341400	-2.44763000	2.83659400
H	-3.00906200	-2.11082300	3.50455200
C	-3.29567400	-0.38286900	1.39739400
H	-3.62259500	-1.41700400	1.35568900
C	-1.79374500	-0.32689900	1.24765000
C	-1.18836100	-0.87193800	0.10009400
C	-0.96440900	0.22862400	2.23704000
C	0.19786700	-0.85706900	-0.05282200
H	-1.81608200	-1.28825500	-0.68785300
C	0.42212500	0.24358200	2.08191600
H	-1.40667300	0.66837500	3.12977700
C	1.01136700	-0.29539000	0.93572900
H	0.64477900	-1.27858800	-0.95644800
H	1.04312100	0.68866800	2.86275600
H	2.09637200	-0.27383600	0.81069100
C	-3.91899100	0.76015900	-1.14929500
C	-4.27988100	-0.20571000	-0.35519600
C	-4.14781100	1.15373100	-2.56299500
C	-2.84172500	1.52264300	-3.28111800
H	-4.80487300	2.03970900	-2.56107000
H	-4.67735900	0.35647500	-3.11952900
C	-3.06031600	1.98237800	-4.71949600
H	-2.35502200	2.31814100	-2.69620500
H	-2.15967400	0.65464300	-3.25958600
H	-2.10861000	2.24775900	-5.20671600
H	-3.54094800	1.19912000	-5.32954100
H	-3.71173700	2.87200500	-4.75603200
C	-5.20365800	-1.37977400	-0.37368700
C	-4.54151700	-2.73767700	-0.65501300
H	-5.95910200	-1.18507700	-1.15530500
H	-5.76238600	-1.43231200	0.57837200
C	-5.53798000	-3.89427400	-0.62062800
H	-4.04420100	-2.68911100	-1.63851400
H	-3.73636800	-2.92519200	0.07677900

H	-5.04862000	-4.85600600	-0.83913300
H	-6.01479700	-3.97857700	0.37032100
H	-6.34198000	-3.75108700	-1.36111300
C	-7.01928700	-0.79845200	4.58226900
C	-7.27308600	-2.27213400	4.21516400
C	-6.72065900	-0.68986100	6.08594600
C	-8.31426300	-0.01161100	4.29088200
H	-7.44352200	-2.38791100	3.13216200
H	-6.43917800	-2.92788100	4.49804000
H	-8.17029100	-2.64534000	4.73486700
H	-6.47165000	0.34346100	6.37291000
H	-7.60237700	-0.99898300	6.67022700
H	-5.88655400	-1.33843400	6.39110500
H	-9.16424000	-0.43122400	4.85223400
H	-8.20764500	1.04704600	4.57560000
H	-8.56700800	-0.04656300	3.21862200
C	-4.85299000	6.82675900	3.83702900
H	-4.85259500	7.11768100	2.77199800
H	-5.65999500	7.40306800	4.32555600
H	-3.90060100	7.19127900	4.26049700
C	-1.53693200	3.61631200	5.24537300
H	-1.88987500	2.57123000	5.28402700
H	-0.48720600	3.61272100	5.59268400
H	-2.11993800	4.16901600	6.00274600

TS5

C	-1.15975700	6.03491600	0.65204300
C	-2.03554200	6.13237400	-0.58468800
H	-1.42237400	6.84075800	1.36415000
H	-0.09821500	6.14719700	0.38927500
H	-2.17254400	7.18420900	-0.88174200
H	-1.54679000	5.60369600	-1.42882900
N	-3.29792200	5.51007800	-0.20845200
N	-1.42726200	4.70388100	1.19681400
C	-4.29820800	5.23498700	-1.26146000
C	-4.36430500	6.42193000	-2.23403300
H	-3.43603300	6.54108800	-2.81154400
H	-5.18464600	6.26933200	-2.95117300
H	-4.55645600	7.35530000	-1.68400900
C	-3.93380100	3.95916600	-2.05060700
H	-3.92081500	3.05419000	-1.40394000
H	-4.65190600	3.76066700	-2.86026400
H	-2.92798800	4.03531800	-2.49044800
C	-5.67116000	5.08174200	-0.59730200

H	-5.67983700	4.22073100	0.08939000
H	-5.91273700	5.97365300	-0.00444200
H	-6.45762600	4.91906300	-1.34817000
C	-0.60883900	4.25377700	2.34797500
C	-0.73306600	5.22592800	3.53695900
H	-1.78646400	5.33301800	3.83124900
H	-0.15847100	4.85312200	4.39912100
H	-0.34124900	6.22340100	3.28553200
C	0.85741800	4.14502700	1.89806000
H	1.47124600	3.72616100	2.70999100
H	0.93818600	3.48382600	1.02160000
H	1.28650000	5.12378800	1.63697500
C	-1.08092900	2.86608600	2.78478000
H	-1.01434600	2.13756400	1.96624800
H	-0.45624200	2.50808100	3.61620400
H	-2.11740300	2.89522600	3.13877800
P	-3.10502000	4.36061400	1.06830000
O	-3.82656000	4.92549100	2.37693600
Al	-5.12943600	4.19090800	3.41418200
Ni	-4.11564900	2.49692400	0.57217200
C	-6.93123300	4.78521200	2.84253400
H	-7.17327600	4.40955400	1.83148000
H	-7.68537800	4.32115100	3.50722400
C	-4.64292400	4.43322000	5.31655900
H	-4.56636600	5.52051200	5.51104500
H	-3.62180600	4.04302600	5.48993800
C	-4.48221900	1.65010200	2.14466700
O	-4.98873900	2.34157500	3.09631300
N	-3.80294800	0.48445300	2.42225100
C	-3.76176300	-0.13710600	3.80399500
C	-3.29141400	0.89567600	4.83955100
C	-5.20927900	-0.64120300	4.06276100
C	-2.69530600	-1.24553400	3.78247200
H	-2.38471300	1.39477300	4.48111700
H	-4.04430800	1.65636400	5.05111700
H	-3.03693700	0.38611700	5.77601600
H	-5.51260700	-1.21958700	3.17312400
H	-5.83557600	0.26295300	4.05584800
H	-2.47849800	-1.56544500	4.80907600
H	-2.99444500	-2.13952900	3.22080200
H	-1.76386200	-0.85915900	3.34451500
C	-3.79407700	-0.44448700	1.28086800
H	-4.02914300	-1.44269800	1.67238100
C	-2.46669100	-0.56889500	0.54429900

C	-1.88843600	-1.82685400	0.34188800
C	-1.83277300	0.55671500	0.00335300
C	-0.70229600	-1.96041800	-0.38748600
H	-2.37029000	-2.71520900	0.75927000
C	-0.64501100	0.43208300	-0.71497000
H	-2.28105600	1.54852500	0.15067900
C	-0.07533000	-0.83056500	-0.91663400
H	-0.26579800	-2.95089300	-0.53703500
H	-0.16372800	1.32588100	-1.11968500
H	0.85344400	-0.93145300	-1.48310200
C	-5.48593400	1.13641000	0.56685900
C	-4.96673600	-0.09515700	0.37838400
C	-7.08824800	6.31430100	2.87396200
H	-8.09387200	6.66280800	2.57414900
H	-6.89968500	6.71657700	3.88479600
H	-6.36098000	6.80709800	2.20524600
C	-5.62108100	3.80427200	6.32168800
H	-5.69733700	2.71026100	6.18792400
H	-5.33958900	3.97482300	7.37711600
H	-6.64256400	4.20254200	6.19470500
C	-6.87171600	1.59164100	0.18289500
C	-7.09663600	1.87891500	-1.30910800
H	-7.11518600	2.49518400	0.76449500
H	-7.60071200	0.82717700	0.51130700
C	-8.45401100	2.52147700	-1.58283000
H	-6.29281200	2.53326400	-1.67517900
H	-7.00276800	0.94243900	-1.88250900
H	-8.60285400	2.71510000	-2.65696300
H	-9.27840500	1.87355800	-1.24200800
H	-8.55082900	3.48119900	-1.04944600
C	-5.48364800	-1.15264000	-0.55421700
C	-4.82767900	-1.09718300	-1.94836100
H	-6.57451800	-1.03526900	-0.66812000
H	-5.31854100	-2.15707900	-0.12505300
C	-5.37995500	-2.15475400	-2.90002000
H	-4.98114500	-0.08937500	-2.36808300
H	-3.73817400	-1.21723000	-1.84076100
H	-4.89915400	-2.09580700	-3.88899600
H	-5.21243000	-3.17165900	-2.50779800
H	-6.46619200	-2.03319200	-3.04727700
C	-5.64386800	-1.48800300	5.29029100
C	-5.04410100	-2.90510300	5.27807700
C	-5.33453500	-0.81129500	6.63586400
C	-7.17631200	-1.62318800	5.16823600

H	-5.21039400	-3.40069200	4.30735000
H	-3.96439400	-2.90632800	5.47758400
H	-5.51964700	-3.52655900	6.05411100
H	-5.67499500	0.23557700	6.64719200
H	-5.84835600	-1.34270900	7.45317200
H	-4.26071000	-0.81919200	6.86796000
H	-7.58308600	-2.23504500	5.98912100
H	-7.66567600	-0.63699000	5.20538200
H	-7.45966600	-2.10277200	4.21701400

TS6

C	-3.67679700	6.41796300	-1.72413800
C	-4.98370700	5.63589200	-1.74097000
H	-3.75850200	7.28396600	-1.03816800
H	-3.44034900	6.79985500	-2.72723600
H	-5.84342300	6.32324200	-1.70981500
H	-5.05412100	5.04644700	-2.67749200
N	-4.94403400	4.77896500	-0.56171600
N	-2.68139200	5.44754100	-1.27784100
C	-6.02420600	3.79465700	-0.34163300
C	-7.38335900	4.47382500	-0.56555900
H	-7.53452200	4.76418900	-1.61578500
H	-8.19456000	3.78110800	-0.29516300
H	-7.47061700	5.37314200	0.06296200
C	-5.87280500	2.59415200	-1.29639600
H	-4.90008100	2.10608600	-1.13049100
H	-6.66897600	1.85013700	-1.13417800
H	-5.91831600	2.91266900	-2.34930800
C	-5.95631400	3.31063500	1.11220700
H	-5.01777700	2.77808300	1.30706900
H	-6.01583400	4.15728300	1.81067100
H	-6.78437200	2.61562600	1.31975700
C	-1.24583600	5.79738400	-1.33257900
C	-0.92514600	6.99749300	-0.42236600
H	-1.21463500	6.77903900	0.61354500
H	0.15088300	7.23035900	-0.44650500
H	-1.46934900	7.89672900	-0.74936800
C	-0.86991200	6.12142800	-2.78808100
H	0.21397700	6.29343700	-2.86955300
H	-1.14476300	5.28608100	-3.45041100
H	-1.37160700	7.03073000	-3.15001700
C	-0.41583100	4.58377100	-0.88876900
H	-0.66912200	3.69436400	-1.48990200
H	0.65679900	4.78825800	-1.02206600

H	-0.56798500	4.35031300	0.17590000
P	-3.34593000	4.48520100	-0.02081100
O	-3.18449700	5.25607000	1.36923400
Al	-2.40880800	4.81345200	2.94589100
Ni	-2.26551900	2.64586200	0.26529700
C	-3.62342700	5.41278700	4.39073200
H	-3.34935200	4.96504100	5.36268200
H	-4.65654300	5.07372600	4.18876400
C	-0.54067800	5.46440300	3.10257400
H	-0.54506000	6.56839100	3.02691900
H	0.09267200	5.12672200	2.26156000
C	-2.52812300	2.04069000	2.00179200
H	-1.81129500	0.30646200	-0.40939400
O	-2.29620500	2.94070300	2.89814200
N	-3.32680300	0.98061600	2.37032000
C	-3.80944300	0.77841300	3.79676500
C	-4.62102600	-0.52611500	3.88273900
C	-2.52746100	0.62897800	4.67320100
C	-4.75552000	1.92195100	4.18580700
H	-5.54682000	-0.48000700	3.29224500
H	-4.04515200	-1.40353100	3.56525900
H	-4.91578400	-0.67997000	4.92640900
H	-1.77348100	1.31122600	4.27231900
H	-2.15661500	-0.39140000	4.48518400
H	-5.18984400	1.73620100	5.17732100
H	-4.25862000	2.89247500	4.20109700
H	-5.58679500	1.96795700	3.46838800
C	-3.84257200	0.15547500	1.29088700
H	-4.93705100	0.07877800	1.37393000
H	-3.67971000	0.73435600	0.36512300
C	-3.25453900	-1.23100000	1.11705700
C	-3.89283600	-2.12672700	0.24889400
C	-2.07045500	-1.63375100	1.74613600
C	-3.35829000	-3.39461500	0.00733900
H	-4.82121000	-1.82659800	-0.24655300
C	-1.53561700	-2.90234800	1.51190100
H	-1.56209500	-0.94180200	2.41484000
C	-2.17495600	-3.78745600	0.63881200
H	-3.87027500	-4.07955600	-0.67300400
H	-0.61095900	-3.19865500	2.01334700
H	-1.75564400	-4.77926900	0.45422000
C	-0.88033500	0.66090100	0.04739600
C	-0.92266400	1.53234300	1.08337700
C	0.28232000	1.99793700	1.85196400

C	0.83905800	0.93786600	2.81463400
H	0.01925200	2.89740000	2.42173900
H	1.07645900	2.29985600	1.14495400
C	2.04347400	1.43662000	3.60894100
H	0.04093000	0.63303400	3.50830400
H	1.10744000	0.03164800	2.24507800
H	2.42110400	0.66433500	4.29760000
H	2.87225600	1.72517400	2.94107800
H	1.78164200	2.32341300	4.20789800
C	0.34688600	0.09836600	-0.61460000
C	0.39427500	0.42247800	-2.11495400
H	0.34045500	-1.00061100	-0.48555100
H	1.26268300	0.46674700	-0.12701100
C	1.61005300	-0.17526400	-2.81919100
H	-0.53270800	0.05732400	-2.59205000
H	0.38649800	1.51943200	-2.24140900
H	1.61833400	0.07215700	-3.89232000
H	2.54929800	0.20185800	-2.38173900
H	1.62186500	-1.27399800	-2.72776800
C	-2.49278200	0.85334400	6.21094000
C	-1.09304100	0.38003100	6.65729900
H	-0.95167700	0.54009800	7.73792100
H	-0.94862000	-0.69307400	6.45158200
H	-0.30063000	0.93445400	6.12889200
C	-2.61473400	2.34409300	6.57596500
H	-1.89210300	2.94922900	6.00795600
H	-3.61384800	2.74912100	6.37691500
H	-2.40691900	2.48937600	7.64851000
C	-3.53917500	0.04104500	6.99030600
H	-4.56630200	0.37822300	6.78643000
H	-3.47637600	-1.03313700	6.75304500
H	-3.37256000	0.15228600	8.07408800
C	-3.61150300	6.94752500	4.51088300
H	-4.28972400	7.33003900	5.29566000
H	-2.60281600	7.32791900	4.74874400
H	-3.91353800	7.42732800	3.56373700
C	0.10252700	5.05067000	4.43717000
H	1.14002700	5.41150200	4.56234600
H	-0.47540500	5.43269400	5.29628100
H	0.13230000	3.95238200	4.55136600

TS7

C	-1.96837200	5.31003100	-1.23484900
C	-3.30472600	5.09924500	-1.93847400

H	-1.94525000	6.31027400	-0.76121300
H	-1.13567400	5.25290200	-1.95068100
H	-3.65817200	6.04780700	-2.37199800
H	-3.19903500	4.37175400	-2.76674700
N	-4.21884100	4.61299800	-0.91754400
N	-1.87900400	4.23539100	-0.24494700
C	-5.65623900	4.93965400	-0.96705800
C	-5.86011800	6.41576000	-0.57726700
H	-5.32525000	7.08556800	-1.26903600
H	-6.92646300	6.69124800	-0.60340700
H	-5.47248400	6.58830000	0.43630400
C	-6.18756500	4.68041200	-2.38603900
H	-5.99703100	3.63904200	-2.67969200
H	-7.27198600	4.86490900	-2.42525500
H	-5.71378900	5.33990700	-3.12831400
C	-6.43083100	4.04613400	0.01327200
H	-6.31120600	2.98619300	-0.24817700
H	-6.09821300	4.19620800	1.04817400
H	-7.50151000	4.29481000	-0.02728500
C	-0.63431500	4.11267200	0.55319300
C	-0.36027800	5.39679600	1.35878500
H	-1.21398700	5.63501600	2.00624800
H	0.53461200	5.27293300	1.98814300
H	-0.18062800	6.25561300	0.69430300
C	0.54024800	3.83576600	-0.40028100
H	1.46462200	3.68196300	0.17667800
H	0.34980800	2.93300700	-0.99898900
H	0.72197800	4.67408100	-1.08841100
C	-0.75337300	2.92497300	1.51969600
H	-1.03386200	2.00566900	0.98461600
H	0.21621000	2.75567700	2.01083100
H	-1.49126100	3.10855800	2.31123800
P	-3.43298100	3.92880700	0.41993400
O	-3.67934600	4.92217200	1.63975600
Al	-4.05521500	4.70814900	3.39259100
Ni	-3.90899800	1.81087000	0.81774000
C	-5.96519800	5.04022900	3.80988900
H	-6.12745300	4.74631200	4.86537200
H	-6.60476300	4.35171000	3.22384600
C	-2.68172600	5.53553600	4.54825700
H	-2.58530000	6.60966200	4.29817100
H	-1.69289200	5.09810900	4.31252400
C	-3.91398200	1.87705300	2.73604200
H	-5.88942900	1.07225700	-1.32866100

O	-3.82376000	2.87597000	3.52967300
N	-3.94812900	0.67079200	3.33949200
C	-4.01119600	-0.53833000	2.47887000
C	-4.91483800	-1.57900000	3.20010300
C	-4.61295300	-0.08635100	1.15036100
C	-2.57206200	-1.04679000	2.27900200
H	-4.35491800	-1.93370800	4.07904400
H	-5.77469000	-1.02512500	3.60328400
H	-1.94010800	-0.22159200	1.92104500
H	-2.14685900	-1.42098200	3.22274900
C	-3.75993800	0.55533400	4.78934300
H	-3.15377400	-0.33669600	4.99784200
H	-3.17626700	1.43347400	5.09926300
C	-5.04567600	0.50339600	5.58872700
C	-5.21033100	-0.44931700	6.60143500
C	-6.08426100	1.41322700	5.34030800
C	-6.38757600	-0.49897900	7.35336000
H	-4.41028500	-1.16835900	6.79993200
C	-7.26143900	1.36431700	6.08904100
H	-5.96845300	2.16747800	4.56221300
C	-7.41870600	0.40792400	7.09739400
H	-6.50027100	-1.25190300	8.13738400
H	-8.05817500	2.08357400	5.88454700
H	-8.34140100	0.37045000	7.68141600
C	-4.94845300	1.17866200	-1.88552400
C	-3.81077400	1.34841000	-1.18198100
H	-4.61173300	-0.94026200	0.45704700
H	-5.66623100	0.21081400	1.27051000
H	-4.00854400	0.51226300	0.00782500
C	-5.09002200	1.05570000	-3.38069300
C	-5.05117900	-0.40486600	-3.86252300
H	-4.30064700	1.62566400	-3.89729200
H	-6.04855900	1.50510500	-3.69454400
C	-5.24572000	-0.54411900	-5.37084700
H	-4.08643300	-0.84895800	-3.56194500
H	-5.82663800	-0.98141000	-3.32757400
H	-5.21021200	-1.59798600	-5.68927700
H	-6.21811000	-0.13131400	-5.68721200
H	-4.46222500	-0.00067100	-5.92491400
C	-2.43911500	1.33633800	-1.80942300
C	-1.64833500	0.06629700	-1.45624000
H	-1.87283600	2.21734400	-1.47113200
H	-2.51034400	1.41151200	-2.90835900
C	-0.28050500	0.00416300	-2.13146200

H	-1.52255100	0.01010000	-0.36157000
H	-2.24465500	-0.82068000	-1.73530000
H	0.26507800	-0.91407900	-1.86215200
H	-0.37747500	0.02683100	-3.22957000
H	0.34655900	0.86219000	-1.83976900
C	-6.44174900	6.48727700	3.60661700
H	-7.49449700	6.64963900	3.90249600
H	-5.83323300	7.19858300	4.19210800
H	-6.35637900	6.79759300	2.55117000
C	-2.97021900	5.37926200	6.05094200
H	-2.19749200	5.83578800	6.69629100
H	-3.93309800	5.84202200	6.32845100
H	-3.04138800	4.31619600	6.34246100
H	-2.52349900	-1.85840900	1.54380900
C	-5.49427800	-2.84471100	2.50546600
C	-6.68600800	-2.51796500	1.58436500
H	-7.42805600	-1.89252900	2.10565200
H	-6.38746100	-1.99344400	0.66781600
H	-7.18994800	-3.44840800	1.27735100
C	-6.03011600	-3.72691100	3.65330800
H	-6.52664400	-4.62719600	3.25775500
H	-5.21449400	-4.05419200	4.31805000
H	-6.76150200	-3.17733700	4.26727300
C	-4.45188300	-3.65741300	1.72137200
H	-4.09901400	-3.12601200	0.82494000
H	-3.57557100	-3.90015600	2.34270700
H	-4.89304200	-4.60827300	1.38161200

TS8

C	-3.29600300	5.85197100	-1.22865500
C	-4.75148900	5.45817700	-1.45262200
H	-3.24612100	6.76057000	-0.59852700
H	-2.80101700	6.07541700	-2.18545600
H	-5.37116000	6.36006700	-1.57053500
H	-4.84973300	4.86073500	-2.38062500
N	-5.13568000	4.70191000	-0.26937200
N	-2.68146800	4.69622400	-0.57867600
C	-6.52737100	4.23637400	-0.09624000
C	-7.48724300	5.41070900	-0.35213300
H	-7.47757200	5.73243300	-1.40417300
H	-8.51802100	5.11019100	-0.11073200
H	-7.21764400	6.27020300	0.28026600
C	-6.84948100	3.08567500	-1.06855400
H	-6.17441300	2.23607700	-0.89364600

H	-7.88850700	2.74141400	-0.94564200
H	-6.72438400	3.40593800	-2.11468400
C	-6.72917700	3.77301500	1.35549200
H	-6.06432400	2.93424800	1.60789600
H	-6.51620200	4.59016000	2.05810500
H	-7.76614300	3.43591300	1.50181000
C	-1.22216100	4.72358600	-0.30821100
C	-0.83222400	5.95231800	0.53431900
H	-1.39473700	5.96552500	1.47651900
H	0.24332200	5.93165500	0.76800100
H	-1.03584900	6.89016300	-0.00473900
C	-0.47408000	4.75321600	-1.65259700
H	0.61290300	4.74660100	-1.47983200
H	-0.73183500	3.87371200	-2.26184900
H	-0.70556300	5.65733300	-2.23517200
C	-0.80915500	3.44431100	0.43423700
H	-1.07978900	2.54716900	-0.13843800
H	0.28062100	3.44663600	0.58398800
H	-1.27409200	3.37374300	1.42674800
P	-3.79520800	4.00898100	0.53269600
O	-3.63543000	4.80558900	1.89498400
Al	-3.30211800	4.39545800	3.61624900
Ni	-3.80882800	1.82450900	0.71527200
C	-4.81940700	4.98183600	4.74758000
H	-4.76508000	4.49472200	5.73923900
H	-5.77329600	4.63321500	4.30791900
C	-1.48366100	4.91612500	4.20132900
H	-1.36238100	6.01003000	4.08037100
H	-0.72324500	4.46843900	3.53428900
C	-3.70097300	1.68021500	2.63106400
O	-3.32271400	2.54926700	3.49106800
N	-4.03807700	0.47889200	3.15444100
C	-4.46225800	-0.61186700	2.24994300
C	-5.85886300	-1.10056100	2.75227300
C	-4.60094100	-0.03439800	0.84052800
C	-3.40749400	-1.72864100	2.31412600
H	-5.68146200	-1.60448100	3.71444100
H	-6.43064400	-0.19533500	3.00170100
H	-3.59151300	-2.50491300	1.56392200
H	-2.40614600	-1.31141600	2.14760500
H	-3.41409600	-2.22292000	3.29579100
C	-3.94800500	0.24276700	4.59874500
H	-3.62611100	-0.79198600	4.77328800
H	-3.15392400	0.90306200	4.97436700

C	-5.22592300	0.51333700	5.36826900
C	-5.61897300	-0.34628600	6.40146700
C	-6.02399500	1.62815900	5.07855600
C	-6.78509200	-0.10089300	7.13238500
H	-5.00855800	-1.22407400	6.63382300
C	-7.18871600	1.87633500	5.80600600
H	-5.73117600	2.31018000	4.28234200
C	-7.57556200	1.01185800	6.83478000
H	-7.07861800	-0.78454700	7.93282700
H	-7.79116100	2.75691800	5.57076300
H	-8.48824200	1.20594900	7.40323100
C	-3.51217300	1.48052400	-1.14008300
C	-3.24059900	0.34913200	-0.56577400
H	-4.82900400	-0.84463900	0.14513500
H	-5.48141400	0.63135100	0.76922100
C	-4.86591400	6.50940900	4.92085400
H	-5.70450800	6.85615000	5.55269000
H	-3.93933000	6.89116300	5.38456500
H	-4.96394400	7.02294300	3.94806900
C	-1.18637600	4.52633800	5.65918700
H	-1.25823800	3.43420200	5.80797500
H	-0.17856900	4.82835900	5.99927900
H	-1.90929900	4.98441300	6.35665800
C	-6.81400000	-2.01750300	1.93498100
C	-7.55762500	-1.25399800	0.82072000
C	-6.12824300	-3.25489700	1.33542900
C	-7.87441700	-2.49617200	2.94923900
H	-8.01284500	-0.32965200	1.21100500
H	-6.90817900	-0.97854800	-0.02001300
H	-8.36865900	-1.87871200	0.41336900
H	-5.57855200	-3.82447900	2.10089000
H	-6.88171900	-3.92791800	0.89571700
H	-5.42325500	-2.99125700	0.53278800
H	-8.64357700	-3.10958500	2.45389500
H	-7.41672600	-3.10335000	3.74642500
H	-8.37721600	-1.64161300	3.42922600
C	-3.34445000	2.14864700	-2.45377600
C	-2.47178100	1.40077700	-3.46828800
H	-2.94022800	3.15547700	-2.27447800
H	-4.35027500	2.31629400	-2.88304200
C	-2.31760300	2.17062900	-4.77849500
H	-1.47758600	1.22154100	-3.02237300
H	-2.90278500	0.40436700	-3.66735900
H	-1.68712900	1.62725700	-5.49978000

H	-3.29666400	2.34582000	-5.25479600
H	-1.85575100	3.15684900	-4.60559800
C	-2.35456100	-0.83973700	-0.73743700
C	-0.95437800	-0.62213800	-0.14158400
H	-2.25590700	-1.03166900	-1.81920300
H	-2.80364100	-1.74702100	-0.30739300
C	-0.08627800	-1.87596200	-0.19589600
H	-0.46822000	0.20533300	-0.68462100
H	-1.05451600	-0.26976000	0.89862100
H	0.91907600	-1.68838200	0.21204900
H	-0.53639100	-2.69675100	0.38727500
H	0.03428800	-2.23536700	-1.23135400

TS9

C	-1.10861200	4.60711100	-1.41936300
C	-2.37105800	4.73487800	-2.25431300
H	-0.76506500	5.61260200	-1.10809900
H	-0.30307800	4.13924300	-2.00351400
H	-2.28789800	5.58287400	-2.95160500
H	-2.51354700	3.81349400	-2.85816000
N	-3.44269700	4.93593000	-1.28844300
N	-1.48573000	3.77134700	-0.28087200
C	-4.84650800	4.93867500	-1.75393300
C	-4.93204900	5.62034300	-3.12753900
H	-4.43747800	5.03492400	-3.91667900
H	-5.98697400	5.74336200	-3.41406200
H	-4.46952200	6.61764200	-3.08984500
C	-5.40896500	3.50111400	-1.87868500
H	-5.74546900	3.07199700	-0.89885100
H	-6.33916600	3.48469300	-2.46863300
H	-4.68990900	2.82119900	-2.35686600
C	-5.68689300	5.74529100	-0.75506400
H	-5.65214300	5.29155000	0.24358400
H	-5.29357300	6.76737800	-0.66708600
H	-6.74042100	5.78621500	-1.07112100
C	-0.42722200	3.42095400	0.70089100
C	0.17141800	4.68190500	1.35363700
H	-0.61567800	5.26245400	1.85263300
H	0.93186200	4.40170000	2.09903100
H	0.66080700	5.32711500	0.60806800
C	0.67361600	2.63031200	-0.02588700
H	1.41968300	2.27071500	0.69881100
H	0.24136900	1.76080900	-0.54499700
H	1.20799100	3.24551700	-0.76492600

C	-1.01605600	2.52048500	1.79298000
H	-1.45666300	1.61057400	1.36753600
H	-0.22163800	2.22515800	2.49411400
H	-1.79029000	3.03113100	2.37622700
P	-3.10326600	4.11136100	0.19609900
O	-3.06567700	5.17263600	1.38339700
Al	-3.82265400	5.10569100	3.04105400
Ni	-4.71010200	2.71249800	0.57047300
C	-5.37560300	6.33119500	3.17358300
H	-5.97289400	6.30129100	2.24409000
H	-6.05757300	5.96624700	3.96564300
C	-2.43691400	5.25198500	4.44863500
H	-1.61517100	4.54026700	4.24331500
H	-1.96685200	6.25293200	4.40116500
C	-4.47838100	2.34793200	2.32648500
O	-4.40395400	3.33593000	3.16258400
N	-3.89485200	1.16064700	2.71477000
C	-4.17523100	-0.05259200	1.87913200
C	-3.31395100	-1.21096500	2.44673800
C	-5.68815600	-0.35869500	1.98495300
C	-3.80775800	0.18576300	0.40638500
H	-2.30211700	-0.80633800	2.60386900
H	-3.71466100	-1.43685200	3.44154100
H	-3.94744600	-0.74648800	-0.15136700
H	-4.48040700	0.91361400	-0.08341300
H	-2.77053600	0.51747700	0.28167200
C	-3.55280400	1.04011200	4.14453300
H	-2.68696900	0.38171800	4.26533900
H	-3.24211000	2.04164400	4.46088000
C	-4.70153200	0.56580600	5.01636800
C	-4.63490300	-0.65583700	5.70070700
C	-5.86964300	1.33833300	5.13582300
C	-5.71462100	-1.11678800	6.45999100
H	-3.72334200	-1.25676600	5.64215700
C	-6.95146200	0.87681500	5.88705100
H	-5.91826500	2.30674200	4.63914900
C	-6.88292300	-0.35589400	6.54467000
H	-5.64173900	-2.07196300	6.98600900
H	-7.85408500	1.48798400	5.96454900
H	-7.73190800	-0.71505800	7.13135300
C	-6.22687500	2.09467000	1.71434800
C	-6.62279900	0.82491100	1.99030600
H	-5.97565000	-1.02592800	1.15868200
H	-5.88541200	-0.93105700	2.90610100

C	-4.97355600	7.78620900	3.46878200
H	-5.83759700	8.46966500	3.56170000
H	-4.40096500	7.86549700	4.40906500
H	-4.32643100	8.19604300	2.67348000
C	-2.99105300	5.00890800	5.86253400
H	-3.44435300	4.00577500	5.95573300
H	-2.22608100	5.08820800	6.65693600
H	-3.78722900	5.73140700	6.11422100
C	-3.11792700	-2.58790200	1.74205200
C	-4.41939400	-3.21579500	1.21745600
C	-2.07199700	-2.53383500	0.61093300
C	-2.55331500	-3.51920000	2.83680500
H	-5.18763800	-3.26759800	2.00386300
H	-4.83995100	-2.66033700	0.36691500
H	-4.22650400	-4.24344700	0.86998500
H	-1.13999700	-2.06043500	0.95958900
H	-1.82329900	-3.55420600	0.27755200
H	-2.41698500	-1.97933400	-0.27075600
H	-2.31756300	-4.51292300	2.42437200
H	-1.62776600	-3.10689400	3.27047900
H	-3.27776900	-3.65480900	3.65581300
C	-7.14167800	3.28440700	1.93397800
C	-8.19166400	3.52347700	0.84193900
H	-6.53125900	4.19062000	2.03842500
H	-7.65984800	3.18954700	2.90627800
C	-8.99289100	4.80232500	1.07650600
H	-7.69393200	3.57344200	-0.13924100
H	-8.87423000	2.66009400	0.78179400
H	-9.73750300	4.96876900	0.28178400
H	-9.53175900	4.76307700	2.03759500
H	-8.33107300	5.68259400	1.11191400
C	-8.03623700	0.46441500	2.38242800
C	-8.81640600	-0.25012800	1.26471400
H	-8.59434300	1.36111500	2.68710600
H	-8.00122100	-0.19472500	3.26809600
C	-10.26264100	-0.54622800	1.65436600
H	-8.79093900	0.37466800	0.35549000
H	-8.30349800	-1.19086700	1.00041000
H	-10.80202600	-1.06652300	0.84722300
H	-10.31062200	-1.18117300	2.55450500
H	-10.81106500	0.38376700	1.87854600

TS-iso

C	-2.69300900	5.91831100	-1.74709400

C	-4.04229000	5.33944800	-2.16332200
H	-2.84149800	6.83823000	-1.14971000
H	-2.09202900	6.18015200	-2.62917000
H	-4.76932100	6.15171200	-2.30946100
H	-3.95546200	4.79436900	-3.11982700
N	-4.46682900	4.44367700	-1.08789100
N	-2.03899900	4.86395900	-0.97000400
C	-5.90542000	4.36510600	-0.73771500
C	-6.37096300	5.69048000	-0.10479300
H	-6.26970700	6.53126400	-0.80891600
H	-7.43116600	5.63118100	0.18701800
H	-5.77351100	5.91458400	0.78765100
C	-6.71850700	4.07724300	-2.00996400
H	-6.41070200	3.12518400	-2.46211100
H	-7.78827400	4.01284100	-1.76028500
H	-6.60295500	4.87028200	-2.76346800
C	-6.13700500	3.21732800	0.24959100
H	-5.76334700	2.26556300	-0.14880200
H	-5.65648100	3.40232000	1.21693500
H	-7.21272200	3.10580100	0.44145900
C	-0.63894900	5.07710500	-0.52747400
C	-0.52041500	6.34136800	0.34465900
H	-1.16991900	6.26048200	1.22755500
H	0.51818700	6.47668100	0.68386400
H	-0.80680100	7.24625700	-0.21241300
C	0.25116300	5.20252900	-1.77486500
H	1.30813500	5.27997900	-1.47803300
H	0.12998600	4.31794700	-2.41820300
H	0.01309000	6.09875600	-2.36672600
C	-0.16009300	3.86497100	0.27886000
H	-0.24440900	2.94207900	-0.31167100
H	0.89271600	4.00721700	0.56424400
H	-0.73798000	3.74706500	1.20555400
P	-3.20703800	4.08127000	-0.00942600
O	-3.45355900	4.94696100	1.30532700
Al	-3.71794500	4.68277700	3.06817200
Ni	-2.81257600	2.05488000	0.46654000
C	-5.45326900	5.37613600	3.69792200
H	-5.61983900	4.99621300	4.72467100
H	-6.27334000	4.94140000	3.09670200
C	-2.04673100	5.04750700	4.06334400
H	-1.21084000	4.56505500	3.51928900
H	-1.82561300	6.13093900	4.00954500
C	-3.23468300	2.08680700	2.23902000

H	-1.57379900	-0.04109300	-0.02581600
O	-3.84899100	2.77221100	3.09568800
N	-3.11276400	0.67886600	2.43399500
C	-1.82127800	0.19134400	3.05900000
C	-1.96274500	0.26796700	4.58836700
C	-1.58784300	-1.23508300	2.49161100
C	-0.68799400	1.13685300	2.63488800
H	-2.26783900	1.28328700	4.88131600
H	-2.70692400	-0.43888700	4.97798700
H	-1.00358100	0.05035800	5.07760400
H	-1.30277300	-1.09448400	1.43987300
H	-2.55962300	-1.74871700	2.45422300
H	0.26132100	0.79463800	3.06009400
H	-0.57400800	1.17180600	1.54316500
H	-0.86155800	2.16007900	2.99571900
C	-4.35836400	0.05780200	2.91756800
H	-4.12494700	-0.96580400	3.24120500
H	-4.74619100	0.59422800	3.79775600
C	-5.41549600	-0.00055200	1.83767200
C	-6.74568300	0.32564900	2.12726600
C	-5.08663400	-0.41738800	0.53898800
C	-7.73492400	0.22258100	1.14547800
H	-7.00730400	0.67230400	3.13054300
C	-6.07234700	-0.51448600	-0.44456300
H	-4.05103400	-0.64807800	0.28847500
C	-7.40065100	-0.19872500	-0.14384100
H	-8.76775900	0.48578100	1.38626900
H	-5.79680100	-0.83089400	-1.45254000
H	-8.17080200	-0.27053600	-0.91551600
C	-1.72719600	0.26828300	-1.07091300
C	-2.43591600	1.39308000	-1.29520800
C	-2.78243200	1.88066800	-2.67136700
H	-2.75231700	2.97933700	-2.69400700
H	-2.04667100	1.53970100	-3.42399500
C	-1.06354000	-0.67405400	-2.04515200
H	-1.54964300	-1.66734800	-1.98237900
H	-1.20891100	-0.32821000	-3.08122100
C	-4.17783400	1.40070200	-3.09259200
C	-4.63991700	1.96023700	-4.43532600
H	-4.89048200	1.67480100	-2.29963000
H	-4.17623400	0.29705000	-3.12581500
H	-5.64215000	1.59159000	-4.70745100
H	-4.68808600	3.06095900	-4.41207400
H	-3.94795300	1.67840600	-5.24645200

C	0.43526000	-0.84766200	-1.76210900
C	1.11932600	-1.84375900	-2.69587800
H	0.56709000	-1.17152900	-0.71290500
H	0.92641100	0.13868200	-1.83395600
H	2.19258100	-1.94476000	-2.46861500
H	0.66386500	-2.84483900	-2.61291700
H	1.02905800	-1.52869000	-3.74879400
C	-0.59151600	-2.26245400	3.09979600
C	0.82173800	-1.70005300	3.32054700
C	-1.11363400	-2.86905600	4.41520700
C	-0.49302900	-3.40026700	2.06215000
H	1.22146900	-1.24300000	2.40161400
H	0.84947100	-0.94500300	4.11988400
H	1.50791900	-2.51005100	3.61570900
H	-2.13937500	-3.25379600	4.29393700
H	-0.47696500	-3.71389200	4.72377200
H	-1.11866400	-2.14655500	5.24123300
H	0.16309000	-4.20707000	2.42499000
H	-1.48333000	-3.83730600	1.85563400
H	-0.08292900	-3.03288500	1.10747200
C	-5.56488800	6.91016000	3.69657100
H	-6.53301200	7.27676200	4.08309600
H	-4.77754700	7.37538700	4.31498500
H	-5.45039700	7.32510500	2.68002600
C	-2.04460800	4.59371200	5.53118200
H	-2.84329900	5.08952100	6.10935600
H	-2.22534800	3.50794000	5.62002800
H	-1.09396300	4.80281800	6.05460300
